# Direct Carboboration of Aryl Alkenes with Stable Organoborons Through Ziegler‐Type Addition

**DOI:** 10.1002/advs.202511395

**Published:** 2025-08-30

**Authors:** Mo Yang, Shengda Chen, Daojing Li, Liuzhou Gao, Guoqiang Wang, Shuhua Li

**Affiliations:** ^1^ State Key Laboratory of Coordination Chemistry Key Laboratory of Mesoscopic Chemistry of Ministry of Education School of Chemistry and Chemical Engineering Nanjing University Nanjing 210093 P. R. China; ^2^ School of Chemistry and Chemical Engineering Yangzhou University Yangzhou 225009 P. R. China

**Keywords:** boron, carboboration, carbometalation, density functional calculations, Ziegler addition

## Abstract

Carbometallation reaction represents a classic strategy for the bifunctionalization of unsaturated hydrocarbons, yet conventional implementations predominantly rely on either highly reactive organometallic reagents or transition metal catalysts. Notably, the direct employment of metalloid organoboron reagents for such addition processes remains elusive except when specific substrate combinations are employed. Herein, a base‐catalyzed carboboration reaction of aryl alkenes with bench‐stable benzylic boronates is reported. This anionic‐based strategy is not only applicable to the carboboration of substituted styrenes but also to vinyl cyclopropanes and vinyl cyclobutane, affording the corresponding 1,5‐ and 1,6‐carboboration‐related products. Combined experimental and computational investigations reveal a mechanism that involves the Ziegler‐type carbometallation process. The synthetic utility is further highlighted by the facile one‐pot derivatization of the resulting benzylic boronates. Furthermore, a modified catalytic system offers an alternative to the anionic polymerization of styrene.

## Graphical abstract

The direct carboboration of vinyl arenes with benzylic boronates has been achieved using KO*^t^* Bu as the catalyst. Vinylcyclopropanes and vinylcyclobutane also participate in this reaction, yielding the corresponding 1,5‐ and 1,6‐carboboration products. Both experimental and computational studies support a carbanion‐related mechanism for this transformation.

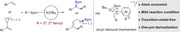

## 1 Introduction

**Scheme 1:**
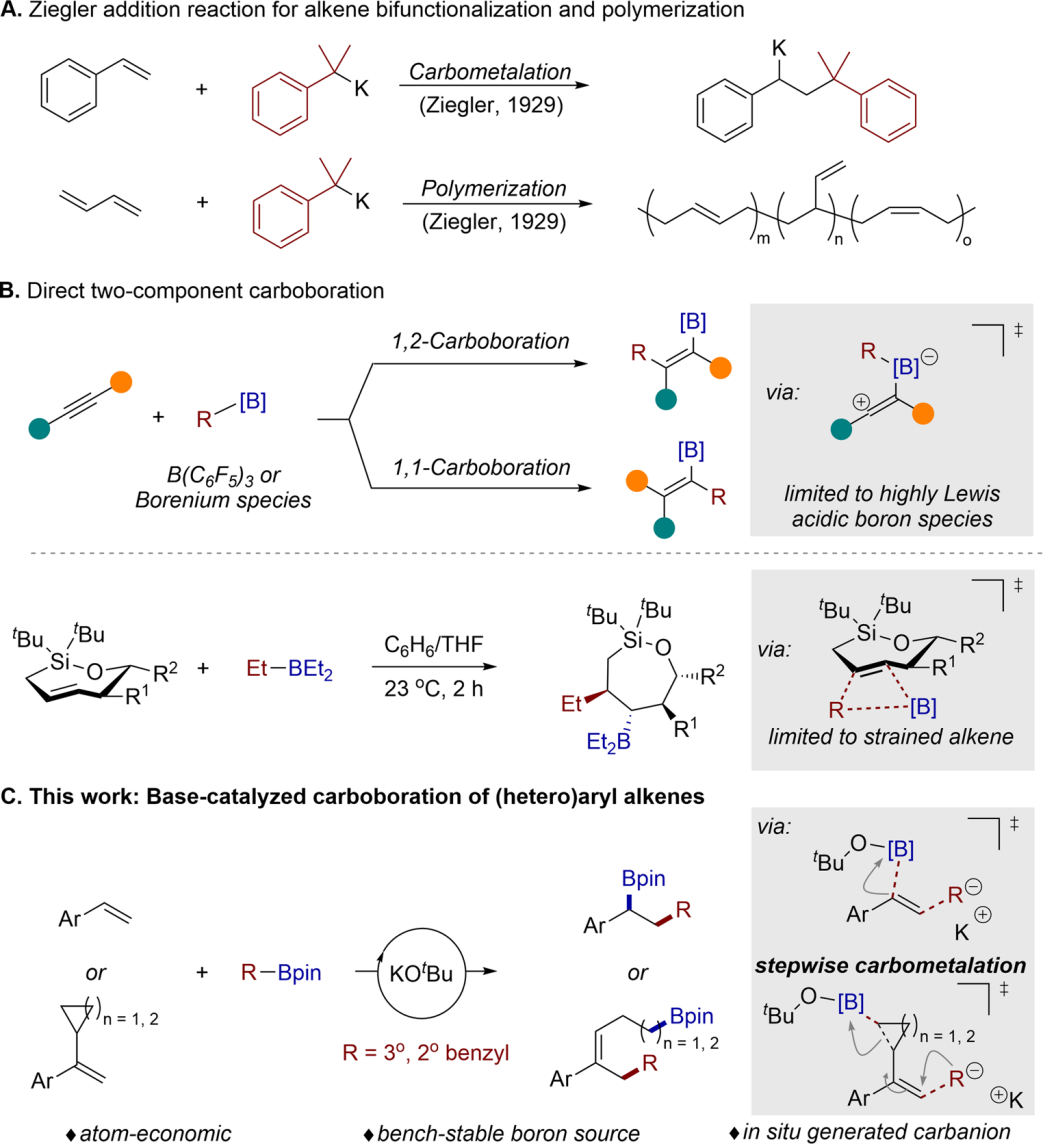
The direct carbometallation and carboboration of unsaturated C─C bonds (previous studies and this work).

Since the discovery of the pioneering Ziegler addition,^[^ ^1^ ^]^ carbometalation reactions through the addition of carbon‐metal bonds to carbon‐carbon unsaturated bonds have become one of the fundamental reactions in synthetic chemistry (**Scheme** **1A**); for example, classical reactions such as bifunctionalization of alkenes^[^ ^2^ ^]^ and anionic polymerization^[^ ^3^ ^]^ (e.g., the Ziegler‐Natta reaction) are regularly practiced both in laboratories and industrial processes. However, these processes typically rely on reactive organometallic reagents (such as organolithiums, organoaluminiums, etc.) or transition‐metal catalysts. For stable organoboron reagents (which are classified as metalloid reagents), although the resulting carbon‐boration products offer ample opportunities for further synthetic transformations, there are only a few examples of direct addition reactions between organoboron compounds and C─C unsaturated bonds. Typically, superacidic boranes, such as borenium ions, B(C_6_F_5_)_3_, are required. For instance, the groups of Sawamura,^[^ ^4^ ^]^ Ingelson,^[^ ^5^ ^]^ Thomas^[^ ^6^ ^]^ et al. have realized 1,1‐ or 1,2‐carboboration of alkynes using Lewis acidic boranes (Wrackmeyer reaction, Scheme 1B, up).^[^ ^7^ ^]^ Woerpel and co‐workers designed highly strained *trans*‐cycloalkenes to achieve direct carboboration with Et_3_B (Scheme 1B, bottom).^[^ ^8^ ^]^ These reactions generally proceed through either stepwise (involves functional group migration) or concerted addition mechanisms,^[^ ^9^ ^]^ limited to specialized boron reagents or highly reactive substrates.

Inspired by the unique reactivity of tetracoordinated boranes,^[^ ^10^ ^]^ we entertained the idea of employing bases to activate the C─B bond to form carbanion species for a Ziegler‐type addition reaction. The boryl group would then rebound to the resulting adduct, ultimately leading to the formation of the carboboration product. Because the carbanion species is in situ formed,^[^ ^11^ ^]^ this strategy would avoid the issue of handling sensitive organometallics and also be complementary to the preparation of carboboration products via either transition‐metal‐catalyzed, photo‐, or base‐mediated three‐component processes.^[^ ^12^ ^]^ Here, we disclose the direct carboboration reaction of aryl alkenes, utilizing benzylic boronates as both carbon nucleophiles and boron electrophiles with KO*^t^* Bu as the catalyst (Scheme 1C). This strategy also applies to remote ring‐opening 1,5‐ and 1,6‐carboboration of vinylcyclopropanes and vinylcyclobutane. Control experiments and density functional theory (DFT) calculations reveal a Ziegler‐type addition process involving benzylic potassium species. Anionic polymerization of electron‐rich styrene could also be achieved using benzylic boronate/KO*^t^* Bu as the initiator under slightly modified conditions.

## 2 Results and Discussion

Our group and Ito's group have recently shown that silyl boronate can undergo anionic‐type silylborylation reaction (e.g., using PhMe_2_SiBpin) with aromatic alkenes in the presence of an alkoxide base.^[^ ^13^ ^]^ We doubt whether benzylic boronates, as the congeners of silylboronates, could also be amenable to carboboration reaction via a carbanion addition pathway. We commenced our study by using *p*‐methoxystyrene **1a** and tertiary benzylic boronate **2a** as model substrates with an alkali‐metal alkoxide as the catalyst (see Table S1, Supporting Information for details). After some trials, we found that utilizing KO*^t^* Bu (30 mol%) as the catalyst results in the carbaboration product **3a** in 98% NMR yield and an 84% isolated yield at 60 °C (**Table** **1**, entry 1). The choice of base and solvent was critical to the reaction outcome.^[^ ^14^ ^]^ Changing KO*^t^* Bu with other alkoxide bases or KHMDS resulted in no or low product yield (entries 2–4), probably due to different solvation, aggregation, basicity, and steric hindrance of the anionic component of the bases (see Supporting Information for more discussion).^[^ ^15^ ^]^ Using other solvents, such as 1,4‐dioxane and toluene, led to lower yields (entries 5 and 6). The current conditions were ineffective for electron‐deficient substrate *p*‐fluoro styrene, likely due to an undesired oligomerization reaction (entry 7, 18%).^[^ ^16^ ^]^ It was found that performing the reaction under neat conditions with increased boronate equivalents could result in a satisfying yield (78%, entry 8).

**Table 1:** Optimization of carboboration of aryl alkenes with benzylic boronates.^a)^

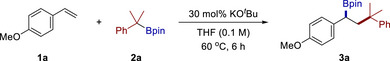		
Entry	Change from standard conditions	Yield [%] ^b)^
1	none	98 (84)
2	KOMe instead of KO*^t^* Bu	n.d.
3	NaO*^t^* Bu instead of KO*^t^* Bu	n.d.
4	KHMDS instead of KO*^t^* Bu	8
5	1,4‐dioxane instead of THF	44
6	toluene instead of THF	9
7	*p*‐fluorostyrene instead of **1a**	18
8	*p*‐fluorostyrene instead of **1a** neat, 3.0 equiv. **2a**	78

^a)^ Reactions were carried out with **1a** (0.1 mmol), **2a** (1.2 equiv.), and KO*^t^* Bu (30 mol%) in THF (1.0 mL) at 60 °C for 6 h;

^b)^ Yields determined by ^1^H NMR using Bn_2_O as internal standard; n.d. = not detected; isolated yields in parentheses.

**Scheme 2:**
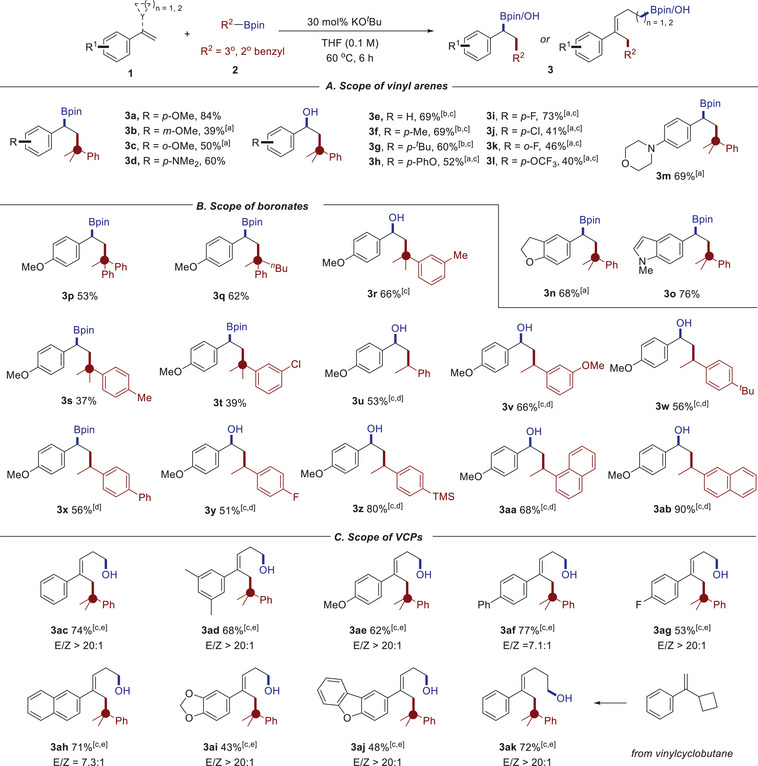
Substrate scope. Reaction conditions: alkene **1** (0.1 mmol), benzylic boronate **2** (1.2 equiv.), KO*^t^* Bu (30 mol%), THF (1.0 mL), 60 °C, 6 h. a) Neat and with 3.0 equiv. **2a**. b) With 2.0 equiv. **2a**. c) One‐pot sequential carboboration/C─B oxidation was conducted due to the instability of some carboboronate products on silica gel or the challenging purification of these nonpolar compounds (with 3.0 equiv. NaBO_3_•4H_2_O, see Supporting Information for details). d) At 80 °C. e) At 40 °C.

With the optimized reaction conditions in hand, we initially explored the substrate scope of aryl alkenes with 2‐phenylpropan‐2‐yl boronate **2a** as the substrate (**Scheme** **2A**), and a series of corresponding carboboration products with an all‐carbon quaternary center were obtained. Additionally, previously reported transition‐metal‐catalyzed carboboration reactions were applicable to only 1° benzylhalides, possibly due to the potential β‐H elimination of 3° and 2° benzylic‐[Ni] species.^[^ ^17^ ^]^ Due to the instability of some carboboration products on silica gel or the challenging purification of some nonpolar products, one‐pot sequential carboboration/C─B oxidation was conducted for some products. It was found that both the substituent position and electronic effect exert significant influences on the optimal conditions and reaction yields. Taking methoxy‐substituted styrenes as an example, the *para*‐methoxy substrate (**3a**, 84% yield) exhibited superior performance compared to its *meta*‐ and *ortho*‐counterparts (17% and 26%, respectively; see Table S2, Supporting Information for details) in the presence of THF. Inspired by the case of *p*‐fluoro styrene, solvent‐free conditions enabled the successful formation of corresponding carboboration products with moderate yield (**3b**, 39%; **3c**, 50%). These outcomes might be attributed to two factors: 1) The electron‐donating conjugative effect of *para*‐electron‐rich groups enhances the charge density on the benzylic atom of the addition intermediate (*p*‐MeO), thereby improving nucleophilicity toward boron species and reducing the barrier height for C─B bond formation; 2) the coordination effect between tetrahydrofuran (THF) solvent and potassium cations may preferentially facilitate an anionic polymerization pathway, consequently competing with and suppressing the desired C─B bond formation. Similarly, phenyl alkene containing an electron‐donating group (N, N‐dimethylaniline) is well tolerated using THF as the solvent, affording the corresponding carboboration products in moderate yield (**3d**). Styrene and its alkyl‐substituted derivatives are also compatible with the reaction conditions using THF as solvent (**1e**→**3e**, **1f**→**3f**, **1g**→**3 g**). However, for styrenes decorated with substituents such as *p*‐phenoxy (**1** **h**), halides (**1i**‐**k**), and trifluoromethoxy (**1l**) with positive Hammett substituent constants (with σ_p_ or σ_m_ values ranging from 0.06 to 0.34), modified reaction condition is required. For these substrates, the optimized solvent‐free protocol employing 3.0 equivalents of boronate proved effective for yielding the corresponding bifunctionalized products (**3h**‐**3l**). Besides, substrates containing heterocycles (e.g., *p*‐morpholino styrene **1m**, 5‐vinyl‐2,3‐dihydrobenzofuran **1n**, and 1‐methyl‐5‐vinyl‐1*H*‐indole **1o**) are also compatible with this protocol, affording the corresponding carboboration products **3m**‐**3o** in good yields.

Then, the scope of benzylic boronates was investigated (Scheme 2B). Phenyl (**2p**), butyl (**2q**) groups instead of the methyl group of **2a,** and the *m*‐methylphenyl (**2r**) instead of the phenyl group of **2a** were well tolerated, producing the highly congested all‐carbon sp^3^ carbon centers with different substitution modes (**3p**‐**3r**). Replacement of the phenyl group in **2a** with *p*‐methylphenyl (**2s**) or *m*‐chlorophenyl (**2t**) substituents was also compatible, although the product yields were lower. This protocol is also applicable to the secondary benzylic boronates. It was found that the reaction outcome seems to be less sensitive to substituents attached to the benzyl ring. A series of secondary benzylic boronates (e.g., containing fluorine, silyl, and naphthyl substituents) is well tolerated, affording the related carboboration or carbohydroxylation products in good to excellent yields (**3u**‐**3ab**). The lower yield of some substrates (such as **3s**, **3t**, and **3u)** might be attributed to several reasons: 1) the possible decomposition of carboboration products during separation; 2) the intrinsic reactivity difference of the substrates toward the carboboration reaction (see Supporting Information for more discussion).

Furthermore, we extended this protocol to the ring‐opening 1,5‐carboboration of vinylcyclopropanes (VCPs) derivatives, which are versatile C3 or C5 synthons and have widely engaged a diverse array of ring‐opening bond reorganization chemistry through either transition‐metal catalysis or carbocation‐based skeleton reorganization.^[^ ^18^ ^]^ Based on the current carbanion addition mechanistic scenario, a two‐stage carboboration followed by C─B hydroxylation affords a wide range of 1,5‐carbohydroxylation products with excellent *E*/*Z* selectivity up to > 20:1 (Scheme 2C, see Figure S1, Supporting Information for the structural determination of *E* isomer). Similar ring‐opening borofunctionalization processes of VCPs are mainly achieved through transition‐metal catalysis.^[^ ^19^ ^]^ To the best of our knowledge, this is the first case of transition‐metal‐free catalytic carboboration of VCPs. Parent VCP **1ac** affords the 1,5‐carbohydroxylation product **3ac** in 74% yield. Substrates with electron‐donating groups, such as 3,5‐dimethyl (**1ad**), *p*‐methoxy (**1ae**), and *p*‐phenyl (**1af**), yielded the corresponding ring‐opening products **3ad**‐**3af** in satisfying yields. The fluorine atom is well tolerated in the reaction (**1ag→3ag**). Products with other (hetero)aryl rings, such as 2‐naphthyl (**1ah**), benzo[*d*][1,3]dioxole (**1ai**), dibenzo[*b,d*]furan (**1aj**), were also obtained under similar conditions. Besides, the vinyl cyclobutane substrate with lower strain energy could also undergo the addition/ring‐opening reaction to generate the 1,6‐carbofuctionalization product **3ak** with a good yield. We have also explored the ring‐opening/carboboration reaction of cyclopentyl or cyclohexyl‐derived substrates, but no corresponding product was obtained. Further DFT calculations reveal that the formation of these products is thermodynamically unfavorable (see Supporting Information for more details).

**Scheme 3:**
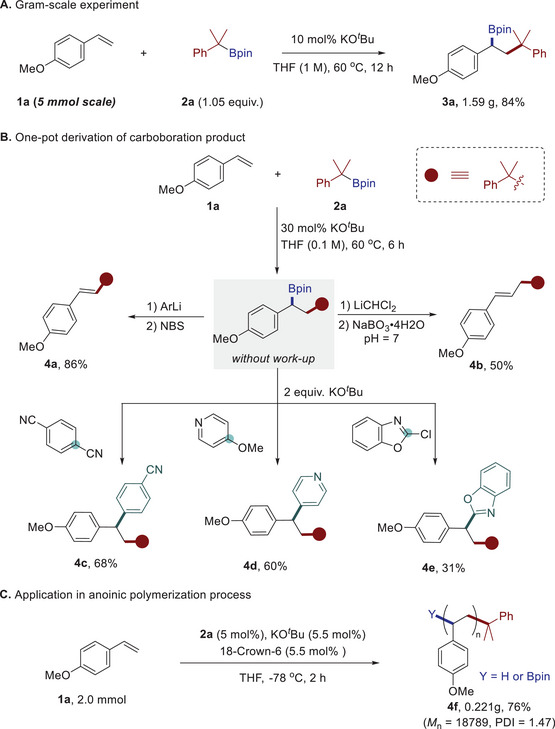
Gram‐scale synthesis and synthetic application. See the Supporting Information for reaction condition details.

To further exhibit the synthetic value of this method, a gram‐scale reaction conducted with *p*‐methoxy styrene **1a** and boronate **2a** affords the related carboboration product **3a** in 84% isolated yield with a lower catalyst loading (10 mol%) (**Scheme** **3** **A**). The boryl group in the adduct could easily undergo downstream diversifications through one‐pot operations (Scheme 3B). For instance, treating the carboboration reaction mixture with [3,5‐bis(trifluoromethyl)phenyl]lithium (ArLi) and N‐bromosuccinimide (NBS) provides an internal alkene product **4a** in *E*‐configuration, probably proceeding through a sequential C─B bromination/elimination process. The successive addition of LiCH_2_Cl reagent and NaBO_3_•4H_2_O resulted in the homologation product **4b** with an elongated carbon chain. The carboboration product **3a** could also undergo nucleophilic aromatic substitution reactions (S_N_Ar) with different types (hetero)aryl electrophiles,^[^ ^13a^ ^]^ such as (hetero)aryl cyanide, ether, and chloride, affording the formal alkene carboarylation product in 31% to 68% yields without using any transition‐metal catalysts or photoirradiation.^[^ ^20^ ^]^ The carbanion addition scenario could be further expanded to the polymerization of *p*‐methoxy styrene^[^ ^21^ ^]^ (Scheme 3C). Using KO*^t^* Bu (5.5 mol%) and boronate **2a** (5.0 mol%) as the initiator and 18‐crown‐6 as additive (5.5 mol%), the reaction could afford the poly (*p*‐methoxy styrene) in 76% yield with the number‐average molecular weight (*M*n) of 18.8 kDa and the polymer dispersity index (PDI) of 1.47. It should be noted that previously reported anionic polymerization of alkoxy‐substituted styrenes usually requires *^n^* BuLi as the initiator, which may lead to undesired *ortho*‐metalation side reaction.

**Scheme 4:**
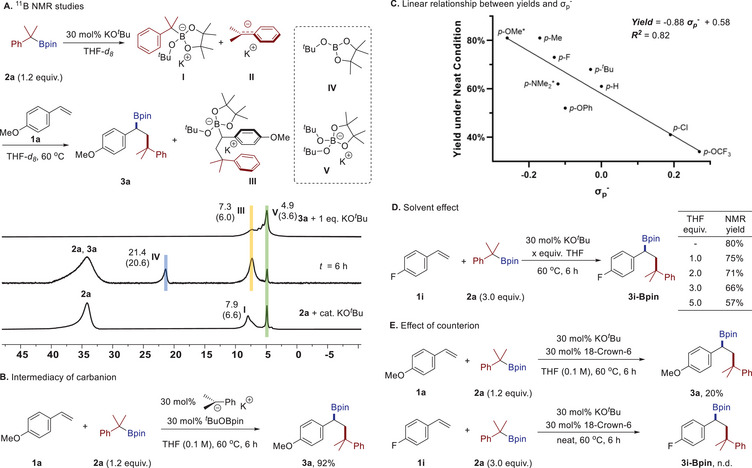
Mechanistic studies. Chemical shifts are shown in ppm, and those in parentheses were computed with the gauge‐independent atomic orbital (GIAO) method at B97‐2/pcSseg‐2 level of theory. *Isolated yield after C─B bond oxidation.

Then, several control experiments were conducted to elucidate the mechanism of this carboboration reaction. Potential reaction intermediates involved in this carboboration were listed in **Scheme** **4A**, based on ^11^B NMR monitoring. Introduction of a catalytic amount of KO*^t^* Bu (30 mol%) into the solution of benzylic boronate **2a** (in THF*‐d_8_*) leads to the formation of two distinct ^11^B signals at upfield (δ = 7.9 and 4.9 ppm). One signal at δ = 7.9 ppm might be assigned to the KO*^t^* Bu‐**2a** ate complex **I**, and the other signal at δ = 4.9 could be assigned to the Lewis adduct **V** based on previous works^[^ ^13^, ^22^ ^]^ and gauge‐independent atomic orbital (GIAO) calculation (computed at B97‐2/pcSseg2 level of theory).^[^ ^23^ ^]^ **V** is formed through the complexation of KO*^t^* Bu and *^t^* BuOBpin fragment **IV** (formed through C─B bond heterolysis). Coupled with the characteristic signals of **II** detected in the ^1^H NMR spectrum (see Figure S3, Supporting Information for details), these observations confirm the generation of carbanionic species even under sub‐stochiometric basic conditions.^[^ ^11^ ^]^ Subsequent introduction of styrene **1a** (at 60 °C) to the mixture produced two signals (δ = 7.3 and 21.4 ppm), tentatively assigned to borate adduct **III** and a free deborylative fragment *^t^* BuOBpin **IV**. The detection of adduct **V** in the mixture of product **3a** and an equimolar amount of base suggests a dynamic equilibration between the carboboration product and its deborylated counterpart via base‐mediated C─B heterolysis. Replacing the KO*^t^* Bu with the independently synthesized cumyl potassium as the catalyst also yielded the carboboration product **3a** in 43% yield. With the addition of *^t^* BuOBpin, a higher yield of 92% would be achieved, further supporting the proposal of cumyl potassium as an important intermediate (Scheme 4B, see Supporting Information for more details). Furthermore, a good linear regression (R^2^ = 0.82) was observed between the *para*‐substituent constants (σ_p_^−^)^[^ ^24^ ^]^ and reaction yields under neat conditions (see Figure S13, Supporting Information).^[^ ^25^ ^]^ This negative correlation not only contains information about a carbanion‐involved mechanism but also reveals that the σ_p_^−^ constant might be an effective descriptor for discriminating between the competing carboboration and anionic polymerization pathways.

Given the pronounced solvent effect for substrates bearing electron‐withdrawing groups (c.f. Table 1, entry 7), the influence of THF stoichiometry on the reaction outcome was then investigated using *p*‐fluorostyrene as the model substrate. As shown in Scheme 4D, incremental addition of THF (0 → 5.0 equiv.) induced progressive attenuation of carboboration yields (80% → 57%), suggesting competitive THF‐K^+^ coordination that potentially facilitates oligomerization pathways. This mechanistic hypothesis was further verified through cation‐chelation experiments (Scheme 4E): introduction of 18‐crown‐6 (30 mol%) to the standard reaction mixture dramatically suppressed carboboration product formation for *p*‐methoxystyrene (92% → 20%) and completely inhibited the reaction for *p*‐fluorostyrene. These outcomes reveal the critical role of potassium cation complexation in the reaction mechanism.^[^ ^26^ ^]^

**Figure 1:**
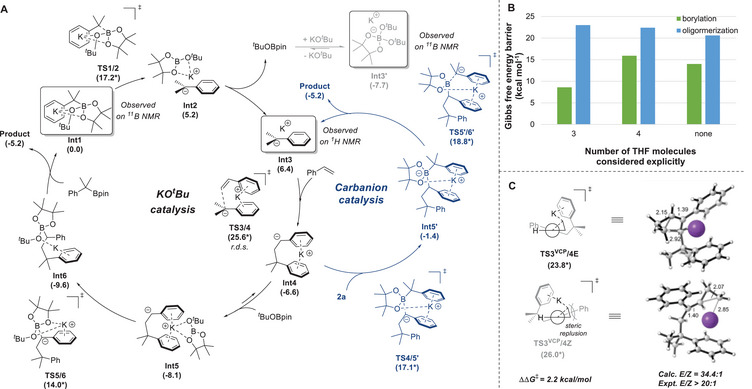
Computational investigations. A) Calculated Gibbs free energy profile of reaction pathways for carboboration of styrene [at (SMD, THF) M06‐2X/def2‐TZVPP//def2‐SVP level of theory]. B) Free energy barriers for competing C─B bond formation and oligomerization steps using different solvation models. C) Anionic ring‐opening step for vinylcyclopropane substrate **1ac** with Newman projections and 3D illustrations of *E*/*Z* selectivity‐determining transition states. For each reaction step, the Gibbs free reaction energies and barriers (labeled with an asterisk) are listed in parentheses in kcal mol^−1^. Selected distances were labeled in Å. Color code: white: hydrogen; grey: carbon; purple: potassium.

Based on the aforementioned control experiments, we performed DFT calculations at (SMD, THF) M06‐2X/def2‐TZVPP//def2‐SVP level of theory^[^ ^27^ ^]^ to disclose the detailed mechanism for KO*^t^* Bu‐catalyzed carboboration of styrene **1e** and benzylic boronate **2a**. The fate of the carbometallation intermediate formed through the addition of benzylic potassium and styrene was systematically investigated using the combined molecular dynamics and coordinate driving methods^[^ ^28^ ^]^ developed by our group. In addition to the desired carboboration pathway, the competing oligomerization process was also identified (see Figure S16, Supporting Information for details). As shown in **Figure** **1**A, the reaction initiates with the deborylation of the KO*^t^* Bu benzylic boronate **2a** complex **Int1** through **TS1/2** with an activation barrier of 17.2 kcal mol^−1^. This low energy barrier indicated a facile carbanion generation process even at room temperature. Although the formation of cumyl potassium **Int3** is endergonic, the complexation of the *^t^* BuOBpin fragment with another KO*^t^* Bu molecule provides the thermodynamic driving force for the deborylation process (**Int3**→**Int3’**). This result was in accordance with the observation of tetracoordinated boronate species **V** in ^11^B NMR studies (c.f. Scheme 4A). The in situ formed cumyl potassium could then undergo carbometalation reaction with styrene^[^ ^1b^ ^]^ through **TS3/4**, forming a more stable carbanion species **Int4**. Then the boryl group of *^t^* BuOBpin rebounds to **Int4** via **TS4/5** to generate the base‐ligated product **Int6**. **Int6** undergoes ligand exchange with substrate **2a** to liberate the carboboration product **3e** and form another ate complex **Int1**. The whole carboboration process is exergonic by 5.2 kcal mol^−1^, and the carbometallation step with an activation barrier of 25.6 kcal mol^−1^ is the rate‐limiting step. Such a barrier height is consistent with the mild reaction temperature required (60 °C).

In addition to binding with *^t^* BuOBpin (**TS5/6**), carbometallation species **Int4** can also react with boronate **2a** through **TS4/5′** to form ate complex **Int5’**. As shown in Figure 1A (blue line), the complexation of **Int4** with **2a** requires a higher barrier compared with **TS5/6** (∆G^‡^ = 17.1 versus 14.0 kcal mol^−1^), and this C─B bond formation pathway is also kinetically accessible.

Because the potassium ion can associate with THF molecules in the solution phase, the influence of solvent on the competition between C─B bond formation and oligomerization reactions (through **Int5**, product selectivity determining step) was then computationally explored through explicit incorporation of 3 or 4 THF molecules in the computational models (see Figure S17, Supporting Information for details). As shown in Figure 1B, with three THF molecules coordinated with the potassium cation, the barrier difference between the carboboration and oligomerization is much larger than that with the implicit solvation model (∆∆G^‡^ = 10.9 versus 6.2 kcal mol^−1^). When the number of explicitly considered THF molecules increases to 4, the activation energy barrier difference becomes closer to the implicit solvation model (∆∆G^‡^ = 5.0 versus 6.2 kcal mol^−1^). These computational results can rationalize the experimental observation that increasing the THF equivalents diminishes the reaction yield when *p*‐fluorostyrene is used as the substrate, as reduced cation solvation is favorable for the desired carboboration pathway relative to oligomerization side reactions, especially for electron‐deficient substrates.

It is worth noting that VCPs (including phenyl VCP) have frequently been utilized as probes for the qualitative identification of free radical reactions (through ring‐opening of cyclopropylmethyl radical).^[^ ^29^ ^]^ We therefore computationally investigate whether the current carbanion mechanistic scenario is applicable to the ring‐opening/1,5‐carboboration reaction of phenyl VCP. Gibbs free energy surface calculations along both the carbanion pathway and the radical pathway were conducted (see Figures S18 and S19, Supporting Information for details). Our computations reveal that the key ring‐opening process through a carbanion intermediate requires an activation barrier of only 23.8 kcal mol^−1^ for the formation of the *E*‐product, which is kinetically feasible under the experimental conditions (Figure 1C). The activation barrier difference between the formation of *E* and *Z* isomers is 2.2 kcal mol^−1^, possibly due to steric repulsion between cyclopropyl and aryl groups (Figure 1C). Such a barrier difference is consistent with the experimentally observed selectivity (Expt. *E*/*Z* > 20:1). Previous studies by Stoltz,^[^ ^30^ ^]^ Jeon,^[^ ^31^ ^]^ and Shibata^[^ ^32^ ^]^ demonstrated that the combination of alkoxide bases with hydrosilanes or silylboronates can lead to radical mechanisms through a single electron transfer (SET) process. In particular, the ring‐opening reaction of the phenyl VCP substrate has been adopted in some cases as evidence for the involvement of a radical mechanism. However, our computational investigations reveal that no thermodynamically feasible SET pathway could be located, despite the lower activation barrier for the radical ring‐opening process. Electron paramagnetic resonance (EPR) spectroscopic analysis on the reaction does not detect any radical signals with or without the styrene substrate (see Figure S14, Supporting Information for details). Therefore, a radical‐based addition mechanism is less likely for the current process.

## 3 Conclusion

In summary, we reported a base‐catalyzed, two‐component carboboration reaction of (hetero)aryl alkenes utilizing bench‐stable benzylic boronates as the boron source. This reaction features mild, transition‐metal‐free conditions and its ability to simultaneously introduce a quaternary or tertiary carbon center along with a modifiable boryl group. Control experiments combined with DFT calculations suggest a Ziegler‐type mechanism involving sequential carbometallation and boryl group rebound. The coordination environment of the potassium cation plays a crucial role in achieving selectivity over oligomerization side reactions. Additionally, we have disclosed a 1,5‐ and 1,6‐carboboration reaction of vinylcyclopropanes and vinylcyclobutane under similar conditions, which proceed via an anionic addition/ring‐opening process rather than a radical pathway. Further investigations into this process are currently underway in our laboratory.

## Conflict of Interest

The authors declare no conflict of interest.

## Supporting information



Supporting Information

## Data Availability

The data that support the findings of this study are available in the supplementary material of this article.
