# Supplementary material for: Direct Carboboration of Aryl Alkenes with Stable Organoborons Through Ziegler‐Type Addition
Source: Adv Sci (Weinh). 2025 Aug 30;12(43):e11395. doi: 10.1002/advs.202511395 (PMC12631861; doi:10.1002/advs.202511395)
Supplement: Supplementary file 1 — Supporting Information [file ADVS-12-e11395-s001.pdf]

Supporting Information

Direct Carboboration of Aryl Alkenes with Stable Organoborons through Ziegler-Type Addition

Mo Yang[a], Shengda Chen[a], Daojing Li[a], Liuzhou Gao[b], Guoqiang Wang*[a] and Shuhua Li*[a]

[a] State Key Laboratory of Coordination Chemistry, Key Laboratory of Mesoscopic Chemistry of Ministry of Education, School of Chemistry and Chemical Engineering, Nanjing University, 210023, Nanjing, P. R. China

[b] School of Chemistry and Chemical Engineering, Yangzhou University, 225009, Yangzhou, P. R. China

Correspondence to: wangguoqiang710@nju.edu.cn; shuhua@nju.edu.cn

Table of Contents

1. General Information **3**

3. Experimental Details for the Carboboration of Alkenes **19**

3.2 Reaction condition optimization 19

3.3 General procedure for base-catalyzed carboboration of alkenes 20

3.4 Characterization data of carboboration/carbohydroxylation products 21

3.5 Unsuccessful examples and low yield discussion 48

4. Synthetic Applications **51**

4.1 Gram-scale experiment 51

4.2 Product transformations 51

4.3 Polymerization process 57

5. Experimental Details for Mechanistic Investigations **58**

5.1 NMR studies of reaction intermediates 58

5.2 Intermediacy of cumyl potassium 63

5.3 Linear regression analysis on experimental yields 64

5.4 Influence of THF addition on reaction yield 70

5.5 Influence of crown ether addition on reaction yield 71

5.6 Electron paramagnetic resonance (EPR) experiments 72

6. Computational Investigations **73**

6.1 Computational details 73

6.2 KO^{t}Bu-catalyzed carboboration of alkene with implicit solvation model 75

6.3 KO^{t}Bu-catalyzed carboboration of alkene with explicit solvent molecules 76

6.4 KO^{t}Bu-catalyzed carboboration of vinylcyclopropanes 77

6.5 Effects of anion parts in base catalysts 79

6.6 Possibility of SET processes 80

7. References **81**

8. NMR Spectra **83**

9. Energies and Cartesian Coordinates of the Optimized Structures **134**

1. General Information

All reactions were carried out with standard Schlenk techniques under argon or in an argon-filled glove box. KO^{t}Bu was purchased from J&K (1 M solution in THF) and Sigma-Aldrich (solid). Chemicals purchased from Sigma-Aldrich, J&K, Bide Pharmtech, and Alfa Aesar Chemical Companies were used as received. Anhydrous solvents, such as 1,4-dioxane, ethyl acetate (EtOAc), cyclohexane, acetonitrile (MeCN), and dimethyl sulfoxide (DMSO) were purchased from J&K and used as received (water < 30 ppm, J&K Seal). THF and toluene were distilled from sodium/benzophenone under an argon atmosphere before use. THF-d_8 was dried by the addition of Na/K alloy. Analytical thin-layer chromatography (TLC) was performed on silica gel 60 F_{254} aluminum sheets from Qingdao Haiyang Chemical Co., Ltd. Flash chromatography was performed on silica gel (200 – 300 mesh, Qingdao Haiyang Chemical Co., Ltd). ^{1}H, ^{11}B, ^{13}C, and ^{19}F NMR spectra were recorded in $CDCl_3$ on a Bruker AVANCE Avance III 400 instrument. Chemical shifts are reported in parts per million (ppm) and are referenced to the residual solvent resonance as the internal standard ($CDCl_3$: 7.26 ppm for ^{1}H NMR and 77.16 ppm for ^{13}C{^{1}H} NMR; THF-d_8: 3.58 ppm for ^{1}H NMR and 67.21 ppm for ^{13}C{^{1}H} NMR). Data are reported as follows: chemical shift (δ ppm), multiplicity (s = singlet, d = doublet, t = triplet, q = quartet, m = multiplet), coupling constants (Hz), and integration. High-resolution mass spectrometry was conducted using Thermo Scientific™ Q Exactive™ UHMR combined Quadrupole Orbitrap™ mass spectrometer equipped with an ESI ionization source. Electron paramagnetic resonance (EPR) spectroscopy was recorded using the Bruker BioSpin EMX PLUS(PPMS) system. Gel permeation chromatography (GPC) analyses were conducted on an Agilent 1260 Infinity II Multidetector GPC/SEC System with THF as eluent. The system was calibrated against linear polystyrene standards in THF.

2. Preparation of Substrates

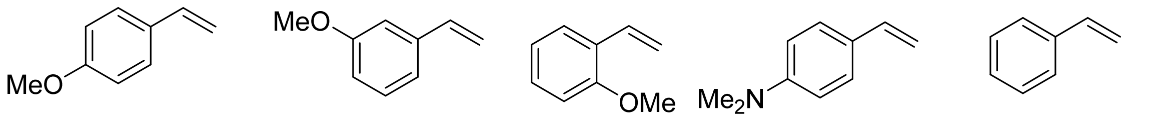

1a **1b** **1c** **1d** **1e**

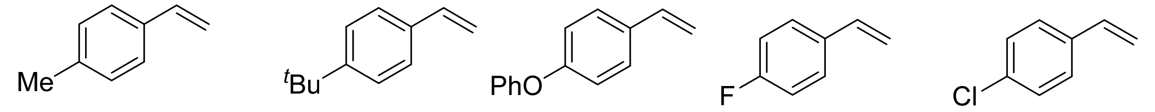

1f **1g** **1h** **1i** **1j**

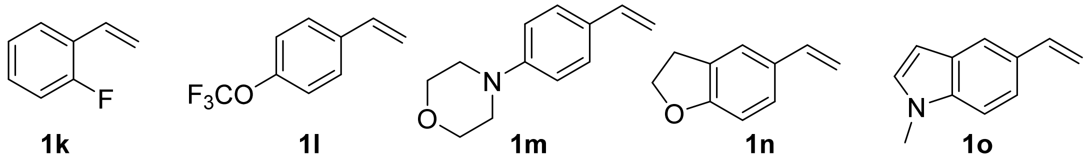

1k **1l** **1m** **1n** **1o**

Scheme S1. Listed aryl alkene substrates. Alkenes **1a-1k** were purchased from Sigma-Aldrich, J&K, and Bide Pharmtech and used as received.

General Procedure 1 (GP1): Synthesis of alkenes **1m-1p**:

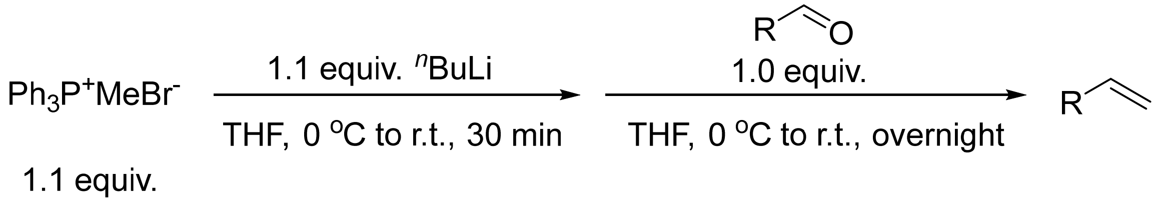

In an oven-dried flask, ^{n}BuLi (1.05 mL, 2.4 M in heptane, 1.1 equiv.) was added dropwise to a suspension of methyltriphenylphosphonium bromide (0.98 g, 1.1 equiv.) in anhydrous THF (2.5 mL) at 0 °C. The resulting reaction mixture was then stirred at room temperature for 30 min. Subsequently, an aldehyde (2.5 mmol, 1.0 equiv.) in anhydrous THF (2.5 mL) was added dropwise at 0 °C, and the reaction mixture was stirred at room temperature for 18 h. The reaction was quenched with saturated aqueous NH_4Cl solution (5 mL) and extracted with petroleum ether (3 × 5 mL). The combined organic layers were dried over Na_2SO_4. After filtration, the organic phase was concentrated under reduced pressure. The crude product was purified by silica gel flash column chromatography to afford the corresponding alkenes.

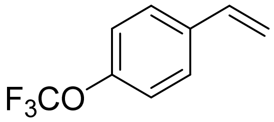

1l

1-(trifluoromethoxy)-4-vinylbenzene (1l)[1]

Prepared according to **GP1** from 4-(trifluoromethoxy)benzaldehyde on a 5 mmol scale. Purified by flash column chromatography (PE) to afford the product as a colorless oil (0.310 g, 33% yield). The spectral data matched those reported in the literature.

^{1}H NMR (400 MHz, $CDCl_3$) δ 7.44 – 7.40 (m, 2H), 7.17 (d, *J* = 8.1 Hz, 2H), 6.70 (dd, *J* = 17.6, 10.9 Hz, 1H), 5.73 (d, *J* = 17.6 Hz, 1H), 5.29 (d, *J* = 10.9 Hz, 1H) ppm.

^{19}F NMR (376 MHz, $CDCl_3$) δ -57.88 ppm.

^{13}C{^{1}H} NMR (101 MHz, $CDCl_3$) δ 148.8, 136.5, 135.6, 127.6, 121.2, 120.6 (q, *J* = 257.0 Hz), 115.0 ppm.

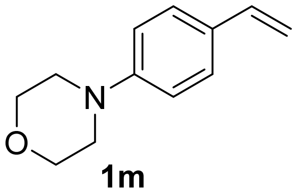

1m

4-(4-vinylphenyl)morpholine (1m)[2]

Prepared according to **GP1** from 4-morpholinobenzaldehyde on 10 mmol scale. Purified by flash column chromatography (pure PE) to afford the product as pale yellow solid (0.985 g, 52%). The spectral data matched those reported in the literature.

^{1}H NMR (400 MHz, $CDCl_3$) δ 7.38 – 7.30 (m, 2H), 6.90 – 6.82 (m, 2H), 6.64 (dd, *J* = 17.6, 10.9 Hz, 1H), 5.60 (dd, *J* = 17.6, 0.9 Hz, 1H), 5.10 (dd, *J* = 10.9, 0.9 Hz, 1H), 3.90 – 3.83 (m, 4H), 3.20 – 3.13 (m, 4H) ppm.

^{13}C{^{1}H} NMR (101 MHz, $CDCl_3$) δ 151.0, 136.4, 129.7, 127.3, 115.6, 111.2, 67.0, 49.3 ppm.

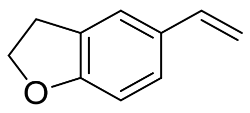

1n

5-vinyl-2,3-dihydrobenzofuran (1n)[3]

Prepared according to **GP1** from 2,3-dihydrobenzofuran-5-carbaldehyde on 5 mmol scale. Purified by flash column chromatography (pure PE) to afford the product as yellow oil (0.482 g, 66% yield). The spectral data matched those reported in the literature.

^{1}H NMR (400 MHz, $CDCl_3$) δ 7.32 – 7.27 (m, 1H), 7.15 (dd, *J* = 8.2, 1.9 Hz, 1H), 6.74 (d, *J* = 8.2 Hz, 1H), 6.65 (dd, , 5.58 (dd, *J* = 17.6, 1.0 Hz, 1H), 5.09 (dd, *J* = 10.9, 1.0 Hz, 1H), 4.58 (t, *J* = 8.7 Hz, 2H), 3.20 (t, *J* = 8.7 Hz, 2H) ppm.

^{13}C{^{1}H} NMR (101 MHz, $CDCl_3$) δ 160.1, 136.7, 130.7, 127.5, 126.9, 122.5, 111.0, 109.3, 71.6, 29.7 ppm.

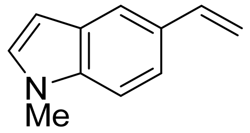

1o

1-methyl-5-vinyl-1*H*-indole (1o)[4]

Prepared according to **GP1** from 1-methyl-1*H*-indole-5-carbaldehyde on 5 mmol scale. Purified by flash column chromatography (pure PE) to afford the product as yellow oil (0.496 g, 63% yield). The spectral data matched those reported in the literature.

^{1}H NMR (400 MHz, $CDCl_3$) δ 7.64 (d, *J* = 5.3 Hz, 1H), 7.38 (t, *J* = 6.6 Hz, 1H), 7.32 – 7.24 (m, 1H), 7.04 (d, *J* = 3.0 Hz, 1H), 6.85 (ddd, *J* = 17.8, 10.9, 7.2 Hz, 1H), 6.48 (t, *J* = 4.2 Hz, 1H), 5.79 – 5.63 (m, 1H), 5.22 – 5.07 (m, 1H), 3.79 (s, 3H) ppm.

^{13}C{^{1}H} NMR (101 MHz, $CDCl_3$) δ 138.1, 136.7, 129.4, 128.7, 119.9, 119.5, 110.9, 109.4, 101.4, 33.0 ppm.

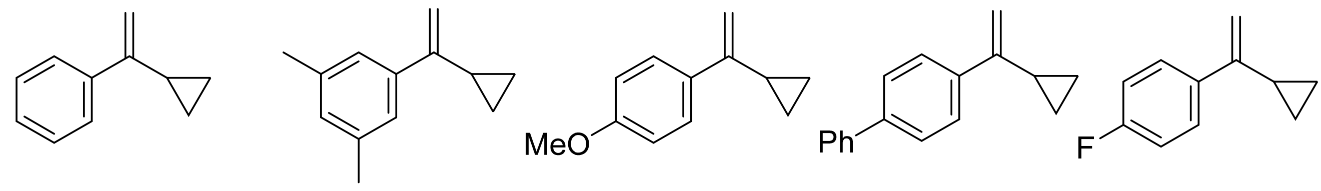

1ac **1ad** **1ae** **1af** **1ag**

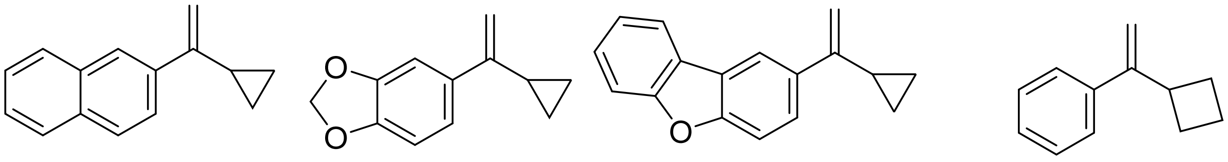

1ah **1ai** **1aj** **1ak**

Scheme S2. Listed vinylcyclopropane substrates.

General Procedure 2 (GP2): synthesis of vinylcyclopropanes **1ac**-**1ak**:

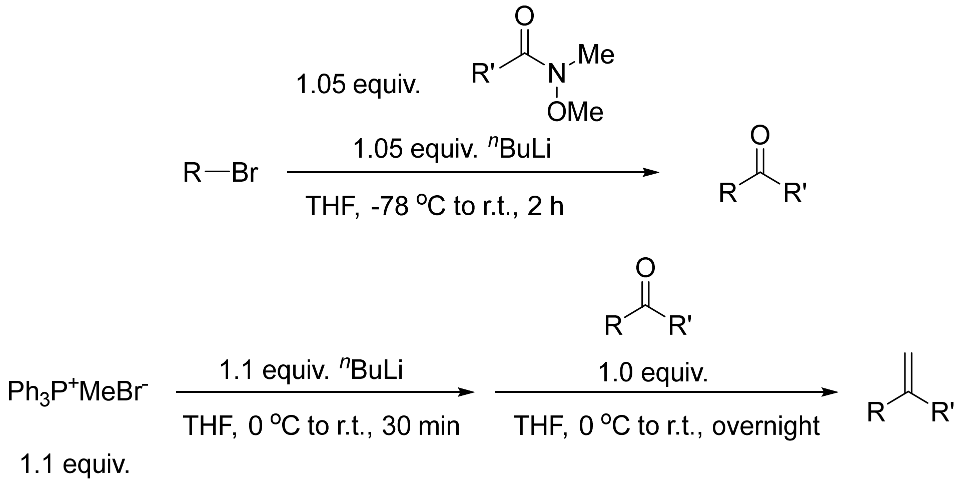

To a solution of the corresponding (hetero)aryl bromide (5 mmol, 1.0 equiv) in anhydrous THF (10 mL) maintained at -78 °C under nitrogen atmosphere, ^{n}BuLi (2.2 mL, 2.4 M in heptane, 1.05 equiv) was added dropwise. The resulting mixture was stirred at -78 °C for 30 min, after which a solution of Weinreb amide (678 mg, 5.25 mmol, 1.05 equiv) in anhydrous THF (1 mL) was transferred via cannula. The reaction vessel was then allowed to warm to ambient temperature (25 °C) over 2 h with continuous stirring. The reaction was carefully quenched by slow addition of saturated aqueous NH_4Cl solution (10 mL) at 0 °C. The organic phase was separated, and the aqueous layer was extracted with EtOAc (3 × 10 mL). The aqueous phases were combined and extracted with EtOAc (3 × 10 mL). The combined organic phase was dried over anhydrous Na_2SO_4, filtered through a Celite pad, and concentrated in vacuo using a rotary evaporator. The residue was purified by flash column chromatography on silica gel to afford the corresponding ketone.

Then, in an oven-dried flask, ^{n}BuLi (1.05 mL, 2.4 M in heptane, 1.1 equiv.) was added dropwise to a suspension of methyltriphenylphosphonium bromide (0.98 g, 1.1 equiv.) in anhydrous THF (2.5 mL) at 0

°C. The resulting reaction mixture was then stirred at room temperature for 30 min. Subsequently, the obtained ketone (2.5 mmol, 1.0 equiv.) in anhydrous THF (2.5 mL) was added dropwise at 0 °C, and the reaction mixture was stirred at room temperature for 18 h. The reaction was quenched with saturated aqueous NH_4Cl solution (5 mL) and extracted with petroleum ether (3 × 5 mL). The combined organic layers were dried over Na_2SO_4. After filtration, the organic phase was concentrated under reduced pressure. The crude product was purified by silica gel flash column chromatography to afford the corresponding vinylcyclopropane.

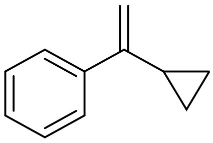

1ac

(1-cyclopropylvinyl)benzene (1ac)[5]

Prepared according to **GP2** from cyclopropyl(phenyl)methanone on 5 mmol scale (2nd step). Purified by flash column chromatography (pure PE) to afford the product as colorless oil (0.558 g, 78% yield). The spectral data matched those reported in the literature.

^{1}H NMR (400 MHz, $CDCl_3$) δ 7.66 – 7.57 (m, 2H), 7.40 – 7.32 (m, 2H), 7.29 (dt, *J* = 7.2, 2.5 Hz, 1H), 5.39 – 5.23 (m, 1H), 4.95 (dd, *J* = 2.5, 1.2 Hz, 1H), 1.66 (tt, *J* = 9.0, 4.6 Hz, 1H), 0.89 – 0.80 (m, 2H), 0.69 – 0.54 (m, 2H) ppm.

^{13}C{^{1}H} NMR (101 MHz, $CDCl_3$) δ 149.5, 141.8, 128.3, 127.6, 126.3, 109.1, 15.8, 6.8 ppm.

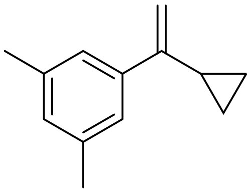

1ad

1-(1-cyclopropylvinyl)-3,5-dimethylbenzene (1ad)[5]

Prepared according to **GP2** from 1-bromo-3,5-dimethylbenzene on 2 mmol scale (2nd step). Purified by flash column chromatography (pure PE) to afford the product as yellow oil(0.206 g, 60% yield). The spectral data matched those reported in the literature.

^{1}H NMR (400 MHz, $CDCl_3$) δ 7.20 (s, 2H), 6.93 (s, 1H), 5.22 (d, *J* = 0.9 Hz, 1H), 4.91 – 4.85 (m, 1H), 2.33 (s, 6H), 1.69 – 1.57 (m, 1H), 0.87 – 0.78 (m, 2H), 0.62 – 0.54 (m, 2H) ppm.

^{13}C{^{1}H} NMR (101 MHz, CDCl$_3$) δ 149.8, 141.9, 137.7, 129.2, 124.2, 108.7, 21.5, 15.8, 6.9 ppm.

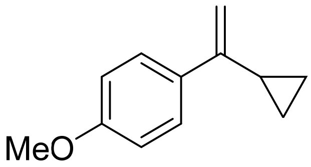

1ae

1-(1-cyclopropylvinyl)-4-methoxybenzene (1ae)[5]

Prepared according to **GP2** from cyclopropyl(4-methoxyphenyl)methanone on 5 mmol scale (2nd step). Purified by flash column chromatography (PE/EtOAc = 50:1) to afford the product as colorless oil (0.765 g, 88% yield). The spectral data matched those reported in the literature.

^{1}H NMR (400 MHz, CDCl$_3$) δ 7.59 – 7.50 (m, 2H), 6.92 – 6.84 (m, 2H), 5.30 – 5.07 (m, 1H), 4.86 (t, *J* = 1.2 Hz, 1H), 3.82 (s, 3H), 1.68 – 1.57 (m, 1H), 0.88 – 0.75 (m, 2H), 0.62 – 0.54 (m, 2H) ppm.

^{13}C{^{1}H} NMR (101 MHz, CDCl$_3$) δ 159.3, 148.7, 134.3, 127.3, 113.6, 107.6, 55.4, 15.8, 6.7 ppm.

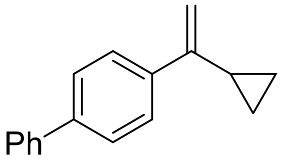

1af

4-(1-cyclopropylvinyl)-1,1'-biphenyl (1af)[5]

Prepared according to **GP2** from 4-bromo-1,1'-biphenyl on 2 mmol scale (2nd step). Purified by flash column chromatography (pure PE) to afford the product as white solid (0.314 g, 73% yield). The spectral data matched those reported in the literature.

^{1}H NMR (400 MHz, CDCl$_3$) δ 7.70 (d, *J* = 8.3 Hz, 2H), 7.66 – 7.56 (m, 4H), 7.45 (t, *J* = 7.6 Hz, 2H), 7.35 (t, *J* = 7.4 Hz, 1H), 5.36 (s, 1H), 4.98 (s, 1H), 1.70 (ddd, *J* = 13.6, 8.2, 5.4 Hz, 1H), 0.92 – 0.81 (m, 2H), 0.68 – 0.59 (m, 2H) ppm.

^{13}C{^{1}H} NMR (101 MHz, CDCl$_3$) δ 149.0, 141.0, 140.7, 140.4, 128.9, 127.4, 127.2, 127.0, 126.6, 109.3, 15.7, 6.8 ppm.

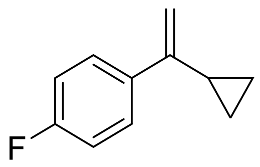

1ag

1-(1-cyclopropylvinyl)-4-fluorobenzene (1ag)[5]

Prepared according to **GP2** from cyclopropyl(4-fluorophenyl)methanone on 2.5 mmol scale (2nd step). Purified by flash column chromatography (pure PE) to afford the product as colorless oil (0.183 g, 23% yield, low yield due to volatility). The spectral data matched those reported in the literature.

^{1}H NMR (400 MHz, $CDCl_3$) δ 7.61 – 7.51 (m, 2H), 7.07 – 6.95 (m, 2H), 5.22 (s, 1H), 4.92 (s, 1H), 1.66 – 1.56 (m, 1H), 0.88 – 0.79 (m, 2H), 0.62 – 0.54 (m, 2H) ppm.

^{19}F NMR (376 MHz, $CDCl_3$) δ -115.42 ppm.

^{13}C{^{1}H} NMR (101 MHz, $CDCl_3$) δ 162.5 (d, *J* = 245.6 Hz), 148.5, 137.8 (d, *J* = 3.2 Hz), 127.8 (d, *J* = 7.9 Hz), 115.0 (d, *J* = 21.3 Hz), 109.1, 15.9, 6.8 ppm.

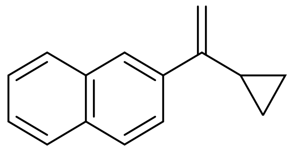

1ah

2-(1-cyclopropylvinyl)naphthalene (1ah)[5]

Prepared according to **GP2** from 2-bromonaphthalene on 2 mmol scale (2nd step). Purified by flash column chromatography (pure PE) to afford the product as colorless oil (0.314 g, 81% yield). The spectral data matched those reported in the literature.

^{1}H NMR (400 MHz, $CDCl_3$) δ 8.08 (s, 1H), 7.89 – 7.83 (m, 2H), 7.81 (d, *J* = 8.7 Hz, 1H), 7.73 (dd, *J* = 8.6, 1.7 Hz, 1H), 7.53 – 7.41 (m, 2H), 5.44 (s, 1H), 5.06 (s, 1H), 1.79 (ddd, *J* = 13.6, 8.4, 5.5 Hz, 1H), 0.96 – 0.84 (m, 2H), 0.71 – 0.63 (m, 2H) ppm.

^{13}C{^{1}H} NMR (101 MHz, $CDCl_3$) δ 149.3, 139.0, 133.5, 133.0, 128.4, 127.7, 127.7, 126.2, 125.9, 124.9, 124.7, 109.8, 15.8, 6.9 ppm.

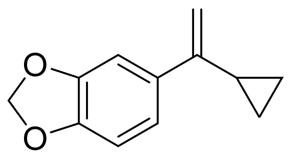

1ai

5-(1-cyclopropylvinyl)benzo[*d*][1,3]dioxole (1ai)[6]

Prepared according to **GP2** from 5-bromobenzo[*d*][1,3]dioxole on 2 mmol scale (2nd step). Purified by flash column chromatography (PE/EtOAc = 50:1) to afford the product as colorless oil (0.239 g, 64% yield). The spectral data matched those reported in the literature.

^{1}H NMR (400 MHz, $CDCl_3$) δ 7.16 – 7.08 (m, 2H), 6.79 (d, *J* = 7.9 Hz, 1H), 5.96 (s, 2H), 5.18 (s, 1H), 4.86 (d, *J* = 1.2 Hz, 1H), 1.65 – 1.54 (m, 1H), 0.87 – 0.78 (m, 2H), 0.58 (qd, *J* = 4.2, 2.1 Hz, 2H) ppm.

^{13}C{^{1}H} NMR (101 MHz, $CDCl_3$) δ 148.9, 147.7, 147.1, 136.2, 119.7, 108.2, 108.0, 106.8, 101.1, 15.9, 6.8 ppm.

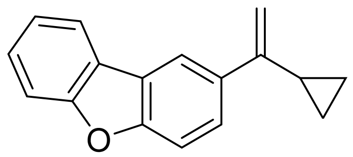

1ah

2-(1-cyclopropylvinyl)dibenzo[*b,d*]furan (1aj)[6]

Prepared according to **GP2** from 2-bromodibenzo[*b,d*]furan on 2 mmol scale (2nd step). Purified by flash column chromatography (PE/EtOAc = 50:1) to afford the product as colorless oil (0.326 g, 70% yield). The spectral data matched those reported in the literature.

^{1}H NMR (400 MHz, $CDCl_3$) δ 8.17 (d, *J* = 1.9 Hz, 1H), 7.99 (dd, *J* = 7.7, 1.3 Hz, 1H), 7.72 (dd, *J* = 8.6, 1.9 Hz, 1H), 7.58 (d, *J* = 8.2 Hz, 1H), 7.53 (d, *J* = 8.6 Hz, 1H), 7.46 (ddd, *J* = 8.4, 7.2, 1.4 Hz, 1H), 7.36 (td, *J* = 7.5, 1.0 Hz, 1H), 5.34 (d, *J* = 1.0 Hz, 1H), 5.03 – 4.97 (m, 1H), 1.76 (ttd, *J* = 8.3, 5.4, 1.2 Hz, 1H), 0.98 – 0.87 (m, 2H), 0.72 – 0.64 (m, 2H) ppm.

^{13}C{^{1}H} NMR (101 MHz, $CDCl_3$) δ 156.7, 155.9, 149.5, 137.0, 127.3, 125.8, 124.5, 124.2, 122.8, 120.8, 118.3, 111.8, 111.2, 109.0, 16.3, 7.0 ppm.

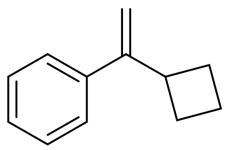

1ak

(1-cyclobutylvinyl)benzene (1ak)[5]

Prepared according to **GP2** from cyclobutyl(phenyl)methanone on 5 mmol scale (2nd step). Purified by flash column chromatography (pure PE) to afford the product as colorless oil (0.730 g, 92% yield). The spectral data matched those reported in the literature.

^{1}H NMR (400 MHz, $CDCl_3$) δ 7.38 (dd, J = 8.4, 1.3 Hz, 2H), 7.35 – 7.29 (m, 2H), 7.29 – 7.22 (m, 1H), 5.35 (t, J = 1.2 Hz, 1H), 5.07 – 5.02 (m, 1H), 3.54 – 3.40 (m, 1H), 2.29 – 2.14 (m, 2H), 2.07 – 1.88 (m, 3H), 1.87 – 1.71 (m, 1H) ppm.

^{13}C{^{1}H} NMR (101 MHz, $CDCl_3$) δ 152.2, 140.9, 128.3, 127.4, 126.2, 109.9, 39.7, 28.6, 17.9 ppm.

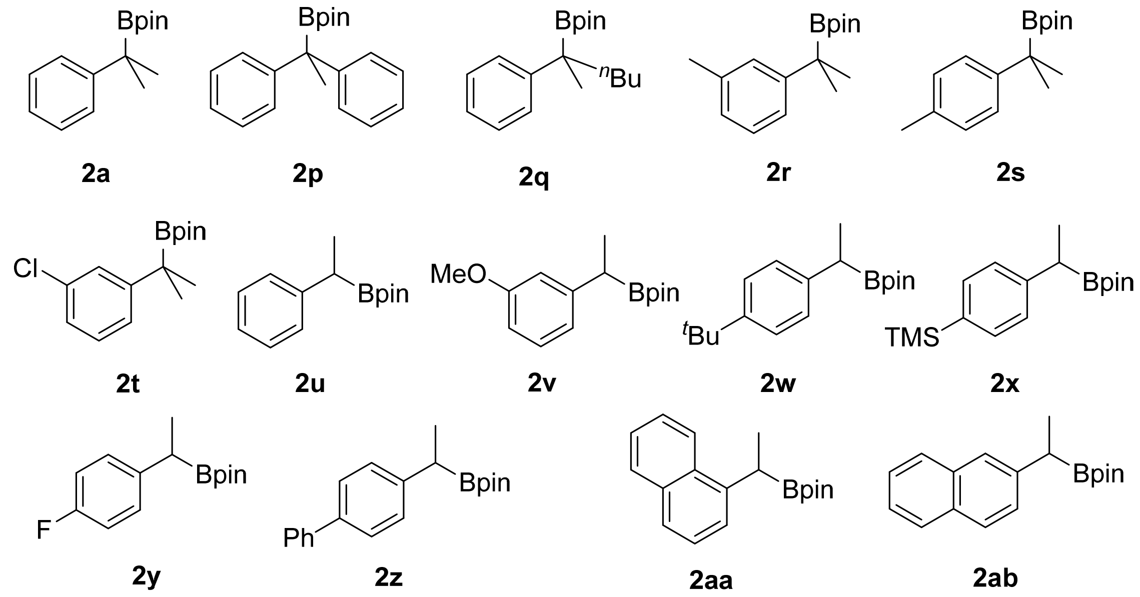

Scheme S3. Listed benzylic boronate substrates.

Benzylic boronates **2q-2t** were synthesized according to previous reports[7].

General Procedure 3 (GP3): Synthesis of boronates **2a**, **2p**, **2u-2ab**, modified from previous report without further optimization**[8]**:

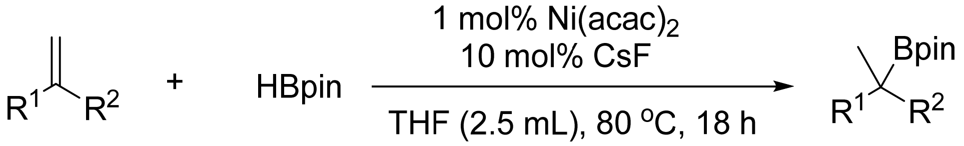

In an argon-filled glovebox, aryl alkene (5 mmol), $Ni(acac)_2$ (13 mg, 0.01 equiv.), CsF (76 mg, 0.1 equiv.), and anhydrous THF (5 mL) were added to an oven-dried vial equipped with a magnetic stir bar. To this suspension, pinacolborane (HBpin, 0.87 mL, 1.2 equiv) was introduced via a syringe. The reaction mixture was stirred at 80 °C for 18 h. Upon completion, the cooled mixture was diluted with ethyl acetate (15 mL) and deliberately oxidized by exposure to atmospheric oxygen. The resulting solution was filtered with a short plug of silica gel and Celite. Then the solution was concentrated in *vacco* and the residue was purified by chromatography on silica gel to afford the product.

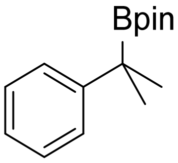

2a

4,4,5,5-tetramethyl-2-(2-phenylpropan-2-yl)-1,3,2-dioxaborolane (2a)[8]

Prepared according to **GP3** from prop-1-en-2-ylbenzene on 10 mmol scale. Purified by flash column chromatography (PE/EtOAc = 50:1) to afford the product as colorless oil (1.32 g, 53% yield). The spectral data matched those reported in the literature.

^{1}H NMR (400 MHz, $CDCl_3$) δ 7.31 – 7.18 (m, 4H), 7.12 – 7.07 (m, 1H), 1.31 (s, 6H), 1.16 (s, 12H) ppm.

^{11}B NMR (128 MHz, $CDCl_3$) δ 34.22 ppm.

^{13}C{^{1}H} NMR (101 MHz, $CDCl_3$) δ 148.8, 128.2, 126.4, 125.1, 83.4, 25.7, 24.6. (The carbon attached to boron was not observed due to quadrupolar relaxation.)

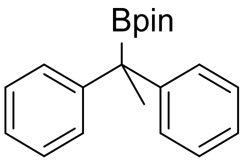

2p

2-(1,1-diphenylethyl)-4,4,5,5-tetramethyl-1,3,2-dioxaborolane (2p)[8]

Prepared according to **GP3** from ethene-1,1-diyldibenzene on 10 mmol scale. Purified by flash column chromatography (PE/EtOAc = 50:1) to afford the product as white solid (1.65 g, 53% yield). The spectral data matched those reported in the literature.

^{1}H NMR (400 MHz, $CDCl_3$) δ 7.29 – 7.20 (m, 8H), 7.19 – 7.13 (m, 2H), 1.68 (s, 3H), 1.20 (s, 12H) ppm.

^{11}B NMR (128 MHz, $CDCl_3$) δ 34.13 ppm.

^{13}C{^{1}H} NMR (101 MHz, $CDCl_3$) δ 147.8, 128.7, 128.1, 125.5, 83.9, 25.9, 24.6 ppm. (The carbon attached to boron was not observed due to quadrupolar relaxation.)

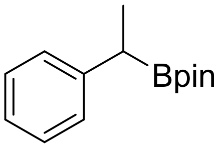

2u

4,4,5,5-tetramethyl-2-(1-phenylethyl)-1,3,2-dioxaborolane (2u)[8]

Prepared according to **GP3** from styrene on 5 mmol scale. Purified by flash column chromatography (PE/EtOAc = 50:1) to afford the product as colorless oil (0.92 g, 79% yield). The spectral data matched those reported in the literature.

^{1}H NMR (400 MHz, $CDCl_3$) δ 7.30 – 7.19 (m, 4H), 7.17 – 7.09 (m, 1H), 2.44 (q, *J* = 7.5 Hz, 1H), 1.33 (d, *J* = 7.5 Hz, 3H), 1.21 (s, 6H), 1.20 (s, 6H) ppm.

^{11}B NMR (128 MHz, $CDCl_3$) δ 33.88 ppm.

^{13}C{^{1}H} NMR (101 MHz, $CDCl_3$) δ 145.1, 128.4, 127.9, 125.2, 83.4, 24.8, 24.7, 17.2 ppm. (The carbon attached to boron was not observed due to quadrupolar relaxation.)

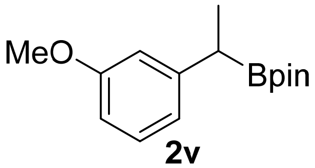

2v

2-(1-(3-methoxyphenyl)ethyl)-4,4,5,5-tetramethyl-1,3,2-dioxaborolane (2v)[8]

Prepared according to **GP3** from 1-methoxy-3-vinylbenzene on 2.5 mmol scale. Purified by flash column chromatography (PE/EtOAc = 50:1) to afford the product as colorless oil (0.50 g, 76% yield). The spectral data matched those reported in the literature.

^{1}H NMR (400 MHz, $CDCl_3$) δ 7.17 (t, *J* = 7.9 Hz, 1H), 6.86 – 6.75 (m, 2H), 6.72 – 6.65 (m, 1H), 3.79 (s, 3H), 2.41 (q, *J* = 7.5 Hz, 1H), 1.32 (d, *J* = 7.5 Hz, 3H), 1.21 (s, 6H), 1.20 (s, 6H) ppm.

^{11}B NMR (128 MHz, $CDCl_3$) δ 33.79 ppm.

^{13}C{^{1}H} NMR (101 MHz, $CDCl_3$) δ 159.7, 146.8, 129.3, 120.5, 113.6, 110.7, 83.4, 55.2, 24.8, 24.7, 17.1 ppm. (The carbon attached to boron was not observed due to quadrupolar relaxation.)

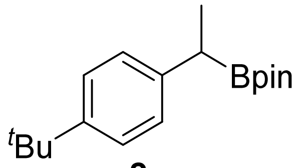

2w

2-(1-(4-(tert-butyl)phenyl)ethyl)-4,4,5,5-tetramethyl-1,3,2-dioxaborolane (2w)[8]

Prepared according to **GP3** from 1-(*tert*-butyl)-4-vinylbenzene on 2.5 mmol scale. Purified by flash column chromatography (PE/EtOAc = 50:1) to afford the product as colorless oil (0.58 g, 81% yield). The spectral data matched those reported in the literature.

^{1}H NMR (400 MHz, $CDCl_3$) δ 7.31 – 7.22 (m, 2H), 7.19 – 7.09 (m, 2H), 2.40 (q, *J* = 7.5 Hz, 1H), 1.34 – 1.27 (m, 12H), 1.22 (s, 6H), 1.21 (s, 6H) ppm.

^{11}B NMR (128 MHz, $CDCl_3$) δ 33.96 ppm.

^{13}C{^{1}H} NMR (101 MHz, $CDCl_3$) δ 147.8, 141.9, 127.5, 125.3, 83.4, 34.4, 31.6, 24.8, 24.8, 17.4 ppm. (The carbon attached to boron was not observed due to quadrupolar relaxation.)

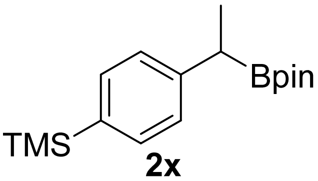

2x

trimethyl(4-(1-(4,4,5,5-tetramethyl-1,3,2-dioxaborolan-2-yl)ethyl)phenyl)silane (2x)[8]

Prepared according to **GP3** from trimethyl(4-vinylphenyl)silane on 2.5 mmol scale. Purified by flash column chromatography (PE/EtOAc = 50:1) to afford the product as colorless oil (0.25 g, 33% yield). The spectral data matched those reported in the literature.

^{1}H NMR (400 MHz, $CDCl_3$) δ 7.49 – 7.35 (m, 2H), 7.21 (d, *J* = 7.9 Hz, 2H), 2.42 (q, *J* = 7.5 Hz, 1H), 1.33 (d, *J* = 7.5 Hz, 3H), 1.22 (s, 6H), 1.21 (s, 6H), 0.24 (s, 9H) ppm.

^{11}B NMR (128 MHz, $CDCl_3$) δ 33.72 ppm.

^{13}C{^{1}H} NMR (101 MHz, $CDCl_3$) δ 145.8, 136.4, 133.6, 127.4, 83.4, 24.8, 24.8, 17.3, -0.9 ppm. (The carbon attached to boron was not observed due to quadrupolar relaxation.)

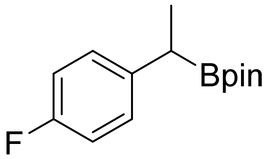

2y

2-(1-(4-fluorophenyl)ethyl)-4,4,5,5-tetramethyl-1,3,2-dioxaborolane (2y)[8]

Prepared according to **GP3** from 1-fluoro-4-vinylbenzene on 2.5 mmol scale. Purified by flash column chromatography (PE/EtOAc = 50:1) to afford the product as pale yellow oil (0.40 g, 64% yield). The spectral data matched those reported in the literature.

^{1}H NMR (400 MHz, $CDCl_3$) δ 7.20 – 7.11 (m, 2H), 6.99 – 6.88 (m, 2H), 2.41 (q, *J* = 7.6 Hz, 1H), 1.31 (d, *J* = 7.6 Hz, 3H), 1.21 (s, 6H), 1.20 (s, 6H) ppm.

^{11}B NMR (128 MHz, $CDCl_3$) δ 33.55 ppm.

^{13}C{^{1}H} NMR (101 MHz, $CDCl_3$) δ 160.9 (d, *J* = 242.5 Hz), 140.5 (d, *J* = 2.1 Hz), 129.0 (d, *J* = 7.7 Hz), 115.0 (d, *J* = 20.9 Hz), 83.4, 24.6, 24.6, 17.2 ppm. (The carbon attached to boron was not observed due to quadrupolar relaxation.)

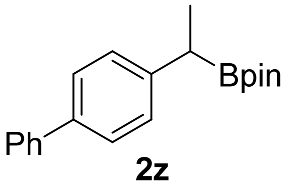

2z

2-(1-([1,1'-biphenyl]-4-yl)ethyl)-4,4,5,5-tetramethyl-1,3,2-dioxaborolane (2z)[8]

Prepared according to **GP3** from 4-vinyl-1,1'-biphenyl on 2.5 mmol scale. Purified by flash column chromatography (PE/EtOAc = 50:1) to afford the product as white solid (0.44 g, 57% yield). The spectral data matched those reported in the literature.

^{1}H NMR (400 MHz, $CDCl_3$) δ 7.62 – 7.56 (m, 2H), 7.53 – 7.47 (m, 2H), 7.46 – 7.38 (m, 2H), 7.34 – 7.27 (m, 3H), 2.48 (q, *J* = 7.5 Hz, 1H), 1.37 (d, *J* = 7.5 Hz, 3H), 1.23 (s, 6H), 1.22 (s, 6H) ppm.

^{11}B NMR (128 MHz, $CDCl_3$) δ 33.87 ppm.

^{13}C{^{1}H} NMR (101 MHz, $CDCl_3$) δ 144.3, 141.4, 138.1, 128.8, 128.3, 127.2, 127.1, 127.0, 83.5, 24.8, 24.8, 17.2 ppm. (The carbon attached to boron was not observed due to quadrupolar relaxation.)

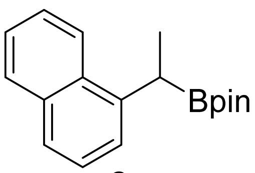

2aa

4,4,5,5-tetramethyl-2-(1-(naphthalen-1-yl)ethyl)-1,3,2-dioxaborolane (2aa)[8]

Prepared according to **GP3** from 1-vinylnaphthalene on 2.5 mmol scale. Purified by flash column chromatography (PE/EtOAc = 50:1) to afford the product as pale yellow oil (0.51 g, 72% yield). The spectral data matched those reported in the literature.

^{1}H NMR (400 MHz, $CDCl_3$) δ 8.17 – 8.06 (m, 1H), 7.89 – 7.78 (m, 1H), 7.67 (dd, *J* = 7.6, 1.9 Hz, 1H), 7.51 – 7.36 (m, 4H), 3.12 (q, *J* = 7.4 Hz, 1H), 1.50 (d, *J* = 7.4 Hz, 3H), 1.21 (s, 6H), 1.20 (s, 6H) ppm.

^{11}B NMR (128 MHz, $CDCl_3$) δ 34.35 ppm.

^{13}C{^{1}H} NMR (101 MHz, $CDCl_3$) δ 141.6, 134.1, 132.2, 128.9, 126.0, 125.5, 125.3, 124.4, 124.2, 83.6, 24.8, 24.7, 16.6 ppm (with one peak overlap in downfield). (The carbon attached to boron was not observed due to quadrupolar relaxation.)

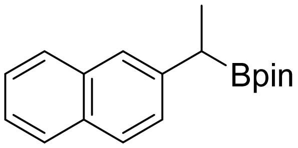

2ab

4,4,5,5-tetramethyl-2-(1-(naphthalen-2-yl)ethyl)-1,3,2-dioxaborolane (2ab)[8]

Prepared according to **GP3** from 1-methoxy-3-vinylbenzene on 2.5 mmol scale. Purified by flash column chromatography (PE/EtOAc = 50:1) to afford the product as colorless oil (0.35 g, 50% yield). The spectral data matched those reported in the literature.

^{1}H NMR (400 MHz, $CDCl_3$) δ 7.95 – 7.71 (m, 3H), 7.67 – 7.62 (m, 1H), 7.46 – 7.34 (m, 3H), 2.61 (q, *J* = 7.5 Hz, 1H), 1.42 (d, *J* = 7.4 Hz, 3H), 1.21 (s, 6H), 1.20 (s, 6H) ppm.

^{11}B NMR (128 MHz, $CDCl_3$) δ 33.87 ppm.

^{13}C{^{1}H} NMR (101 MHz, $CDCl_3$) δ 142.7, 134.0, 131.9, 127.8, 127.7, 127.6, 127.4, 125.8, 125.4, 124.9, 83.5, 24.8, 24.8, 17.0 ppm. (The carbon attached to boron was not observed due to quadrupolar relaxation.)

3. Experimental Details for the Carboboration of Alkenes

3.2 Reaction condition optimization

General procedure: In an argon-filled glovebox, 4,4,5,5-tetramethyl-2-(2-phenylpropan-2-yl)-1,3,2-dioxaborolane **2a** (1.2 equiv., 29.5 mg), base, and solvent (1.0 mL) were added to an oven-dried reaction vial equipped with a magnetic stir bar. Then *para*-methoxystyrene **1a** (0.1 mmol, 13.4 mg) was added to the reaction mixture. The reaction vial was capped, removed from the glovebox, and stirred at varied temperatures. After the indicated time, the reaction mixture was quenched with saturated NH_4Cl (aqueous). The organic phase was extracted with EtOAc three times. Then the combined solution was concentrated in *vacuo* and the internal standard was added. The yield was determined by ^{1}H NMR analysis based on the integration of the target product and internal standard. The results are tabulated in Table S1.

Table S1. Optimization of Reaction Conditions.[a]

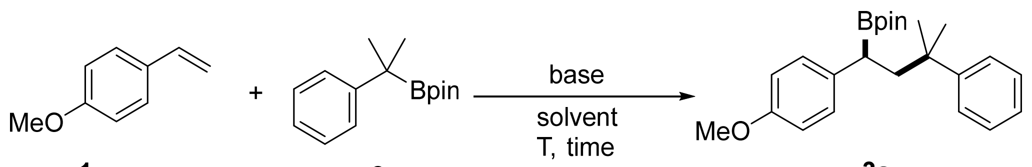

1a **2a** **3a**

Entry	Base (equiv.)	Solvent	T (°C)	Time (h)	Yield/%[b]
1	KO^{t}Bu (0.2)	THF	80	6	82
2	KO^{t}Bu (0.3)	THF	80	6	77
3	KO^{t}Bu (0.4)	THF	80	6	67
4	KO^{t}Bu (0.2)	THF	60	6	88
**5**	**KO^{t}Bu (0.3)**	**THF**	**60**	**6**	**98 (84)**
6	KOtBu (0.3)	THF	40	6	52
7	KOtBu (0.3)	THF	r.t.	6	64
8	KO^{t}Bu (0.3)	THF	60	12	67
9	KO^{t}Bu (0.3)	THF	60	3	59
10	KO^{t}Bu (0.3)	1,4-Dioxane	60	6	44
11	KO^{t}Bu (0.3)	MeCN	60	6	n.d.
12	KO^{t}Bu (0.3)	DMAc	60	6	n.d.
13	KO^{t}Bu (0.3)	Toluene	60	6	9

14	KOMe (0.3)	THF	60	6	n.d.
15	NaO^{t}Bu (0.3)	THF	60	6	n.d.
16	KHMDS (0.3)	THF	60	6	8
17	LDA (0.3)	THF	60	6	n.d.
18	PhLi (0.3)	THF	60	6	n.d.

[a] Reaction conditions: alkene **1a** (0.1 mmol), benzylic boronate **2a** (1.2 equiv.), with indicated catalyst, solvent, stirred under indicated temperature and time. [b] Yield determined by ^{1}H NMR using Bn_2O as internal standard; n.d. = not detected; isolated yields in parentheses.

Based on our mechanistic investigations, the lower yield under decreased temperature (40 °C) was attributed to the incomplete reaction with a slower reaction rate. The inferior yield under increased temperature (80 °C) might be attributed to the existence of side reactions, such as oligomerization and unfavorable entropy effect for multimolecular reactions under elevated reaction temperature. The reaction showed an inferior yield at room temperature, so the reaction was further optimized at 60 °C for better efficiency and also to avoid possible side reactions under prolonged reaction time.

3.3 General procedure for base-catalyzed carboboration of alkenes

General Procedure A (in THF solution): In an argon-filled glovebox, benzyl boronate **2** (0.12 mmol, 1.2 equiv.), KO^{t}Bu (30 mol%, 30 μL, 1 M in THF) and anhydrous THF (1.0 mL) were added to an oven-dried reaction vial containing a magnetic stir bar. The alkene **1** (0.1 mmol) was subsequently added to the reaction mixture. The reaction vial was sealed, removed from the glovebox, and stirred at the indicated temperature for 6 hours (unless other noted). After the reaction finished, saturated NH_4Cl aqueous solution (2 mL) was added to the reaction mixture, and the organic phase was separated. The aqueous layer was extracted with EtOAc (3×2 mL). Then, the organic phase was combined and dried over anhydrous Na_2SO_4. After filtration, the solvent was removed under reduced pressure. The crude material was purified by flash column chromatography on silica gel or preparative TLC to afford the corresponding carboboration products.

General Procedure B (neat): In an argon-filled glovebox, alkene **1** (0.1 mmol), benzyl boronate **2** (0.3 mmol, 3.0 equiv.), KO^{t}Bu (30 mol%, 3.3 mg) were added to an oven-dried reaction vial containing a magnetic stir bar. The reaction vial was sealed, removed from the glovebox, and stirred at varied temperatures for 6 h. After the reaction finished, saturated NH_4Cl aqueous solution (2 mL) was added to the reaction mixture, and the organic phase was separated. The aqueous layer was extracted with EtOAc (3×2 mL). Then, the organic layers were combined and dried over anhydrous Na_2SO_4. After filtration, the solvent was removed under reduced pressure. The crude material was purified by flash column chromatography on silica gel or preparative TLC to afford the corresponding carboboration products.

General Procedure for one-pot C–B oxidation: After the carboboration reaction was finished, 1 mL water (for **General Procedure A**) or 2 mL THF/water solution (v/v = 1:1) (for **General Procedure B**) was added to the reaction mixture. Then $NaBO_3 \cdot 4H_2O$ (3.0 equiv. according to benzyl boronates) was added, and the reaction mixture was stirred for 3 h at room temperature. After the reaction finished, the reaction mixture was diluted with EtOAc (2 mL), and the organic phase was separated. The aqueous layer was extracted with EtOAc (3×2 mL). Then, the organic layers were combined, dried over anhydrous Na_2SO_4. After filtration, the solvent was removed under reduced pressure. The crude material was purified by flash column chromatography on silica gel or preparative TLC to afford the corresponding oxidation products.

3.4 Characterization data of carboboration/carbohydroxylation products

2-(1-(4-methoxyphenyl)-3-methyl-3-phenylbutyl)-4,4,5,5-tetramethyl-1,3,2-dioxaborolane (3a)

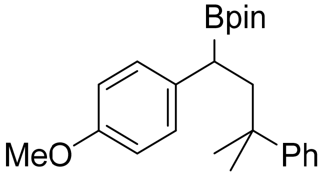

3a
$C_{24}H_{33}BO_3$
M = 380.34 g/mol

Prepared form 1-methoxy-4-vinylbenzene **1a** (13.4 mg, 0.1 mmol) and 4,4,5,5-tetramethyl-2-(2-phenylpropan-2-yl)-1,3,2-dioxaborolane **2a** (29.5 mg, 1.2 equiv.) according to General Procedure A. Purified by flash column chromatography (PE/EtOAc = 50:1) to afford the product as colorless oil (32.0 mg, 84% yield).

^{1}H NMR (400 MHz, $CDCl_3$) δ 7.35 – 7.22 (m, 4H), 7.18 – 7.09 (m, 1H), 7.02 – 6.94 (m, 2H), 6.77 – 6.69 (m, 2H), 3.74 (s, 3H), 2.36 (dd, *J* = 13.5, 9.3 Hz, 1H), 2.08 (dd, *J* = 9.3, 3.5 Hz, 1H), 1.92 (dd, *J* = 13.5, 3.5 Hz, 1H), 1.31 (s, 3H), 1.25 (s, 3H), 1.09 (s, 12H) ppm.

^{11}B NMR (128 MHz, $CDCl_3$) δ 34.47 ppm.

^{13}C{^{1}H} NMR (101 MHz, $CDCl_3$) δ 157.3, 149.4, 136.7, 129.1, 128.2, 126.2, 125.6, 113.8, 83.3, 55.3, 47.2, 38.9, 30.1, 28.4, 27.5, 24.8, 24.5 ppm.

HRMS (ESI): calculated for $C_{24}H_{33}BO_3Na^+$ $[M+Na]^+$ 403.2415, found 403.2398.

2-(1-(3-methoxyphenyl)-3-methyl-3-phenylbutyl)-4,4,5,5-tetramethyl-1,3,2-dioxaborolane (3b)

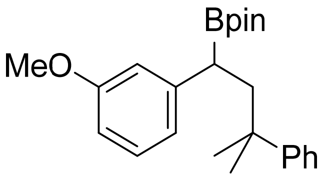

3b

$C_{24}H_{33}BO_3$

M = 380.34 g/mol

Prepared form 1-methoxy-3-vinylbenzene **1b** (13.4 mg, 0.1 mmol) and 4,4,5,5-tetramethyl-2-(2-phenylpropan-2-yl)-1,3,2-dioxaborolane **2a** (73.8 mg, 3.0 equiv.) according to General Procedure B. Purified by flash column chromatography (PE/EtOAc = 50:1) to afford the product as colorless oil (15.0 mg, 39% yield).

^{1}H NMR (400 MHz, $CDCl_3$) δ 7.33 (dd, *J* = 8.4, 1.2 Hz, 2H), 7.30 – 7.24 (m, 2H), 7.19 – 7.13 (m, 1H), 7.10 (t, *J* = 7.8 Hz, 1H), 6.68 (d, *J* = 7.6 Hz, 1H), 6.65 – 6.60 (m, 2H), 3.74 (s, 3H), 2.39 (dd, *J* = 13.5, 9.2 Hz, 1H), 2.12 (dd, *J* = 9.2, 3.4 Hz, 1H), 1.97 (dd, *J* = 13.5, 3.4 Hz, 1H), 1.33 (s, 3H), 1.27 (s, 3H), 1.12 (s, 6H), 1.12 (s, 6H) ppm.

^{11}B NMR (128 MHz, $CDCl_3$) δ 33.82 ppm.

^{13}C{^{1}H} NMR (101 MHz, $CDCl_3$) δ 159.6, 149.3, 146.4, 129.2, 128.2, 126.2, 125.6, 120.8, 113.7, 110.9, 83.4, 55.2, 47.0, 38.9, 30.1, 28.4, 24.8, 24.5 ppm. (The carbon attached to boron was not observed due to quadrupolar relaxation.)

HRMS (ESI): characterized after oxidation following the general procedure; calculated for $C_{18}H_{23}O_2^+$ $[M+H]^+$ 271.1693, found 271.1682.

2-(1-(2-methoxyphenyl)-3-methyl-3-phenylbutyl)-4,4,5,5-tetramethyl-1,3,2-dioxaborolane (3c)

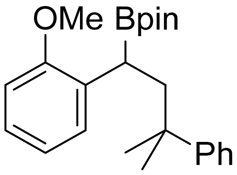

3c

$C_{24}H_{33}BO_3$

M = 380.34 g/mol

Prepared form 1-methoxy-2-vinylbenzene **1c** (13.4 mg, 0.1 mmol) and 4,4,5,5-tetramethyl-2-(2-phenylpropan-2-yl)-1,3,2-dioxaborolane **2a** (73.8 mg, 3.0 equiv.) according to General Procedure B. Purified by flash column chromatography (PE/EtOAc = 50:1) to afford the product as colorless oil (19.0 mg, 50% yield) ppm.

^{1}H NMR (400 MHz, $CDCl_3$) δ 7.32 (d, *J* = 7.5 Hz, 2H), 7.24 (t, *J* = 5.8 Hz, 2H), 7.13 (t, *J* = 7.2 Hz, 1H), 7.09 – 7.01 (m, 1H), 6.91 (dd, *J* = 7.5, 1.5 Hz, 1H), 6.82 – 6.68 (m, 2H), 3.71 (s, 3H), 2.45 – 2.33 (m, 1H), 1.87 (dd, *J* = 12.3, 3.5 Hz, 1H), 1.30 (s, 3H), 1.23 (s, 3H), 1.16 (s, 6H), 1.13 (s, 6H) ppm.

^{11}B NMR (128 MHz, $CDCl_3$) δ 33.85 ppm.

^{13}C{^{1}H} NMR (101 MHz, $CDCl_3$) δ 156.8, 149.6, 133.9, 130.1, 127.9, 126.4, 126.1, 125.3, 120.4, 110.2, 83.1, 55.2, 45.8, 39.1, 29.5, 28.9, 24.8, 24.7 ppm. (The carbon attached to boron was not observed due to quadrupolar relaxation..)

HRMS (ESI): calculated for $C_{24}H_{34}BO_3^+$ $[M+H]^+$ 381.2596, found 381.2602.

***N*,*N*-dimethyl-4-(3-methyl-3-phenyl-1-(4,4,5,5-tetramethyl-1,3,2-dioxaborolan-2-yl)butyl)aniline (3d)**

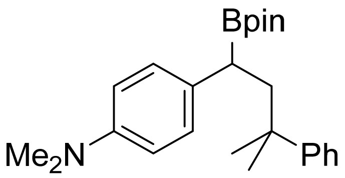

3d
$C_{25}H_{36}BNO_2$
M = 393.38 g/mol

Prepared form *N*,*N*-dimethyl-4-vinylaniline **1d** (14.7 mg, 0.1 mmol) and 4,4,5,5-tetramethyl-2-(2-phenylpropan-2-yl)-1,3,2-dioxaborolane **2a** (29.5 mg, 1.2 equiv.) according to General Procedure A. Purified by flash column chromatography (PE/EtOAc = 2:1) to afford the product as white solid (23.6 mg, 60% yield, m.p. = 62.3 – 66.3 ^{o}C).

^{1}H NMR (400 MHz, $CDCl_3$) δ 7.27 (d, *J* = 7.8 Hz, 2H), 7.20 (t, *J* = 7.7 Hz, 2H), 7.08 (t, *J* = 7.2 Hz, 1H), 6.89 (d, *J* = 8.6 Hz, 2H), 6.56 (d, *J* = 8.6 Hz, 2H), 2.81 (s, 6H), 2.30 (dd, *J* = 13.4, 9.7 Hz, 1H), 1.98 (dd, *J* = 9.6, 2.9 Hz, 1H), 1.86 (dd, *J* = 13.5, 3.1 Hz, 1H), 1.26 (s, 3H), 1.20 (s, 3H), 1.04 (s, 12H) ppm.

^{11}B NMR (128 MHz, $CDCl_3$) δ 34.13 ppm. *The ^{11}B NMR spectrum revealed the presence of impurities, which were attributed to the decomposition of the product following its isolation (at δ 22.54 ppm)*

$^{13}C\{^1H\}$ NMR (101 MHz, $CDCl_3$) δ 149.6, 148.6, 132.9, 128.9, 128.1, 126.2, 125.5, 113.3, 83.2, 47.4, 41.1, 38.9, 30.1, 28.4, 27.2, 24.8, 24.6 ppm.

HRMS (ESI): calculated for $C_{25}H_{37}BNO_2^+$ $[M+H]^+$ 394.2912, found 394.2894.

3-methyl-1,3-diphenylbutan-1-ol (3e)

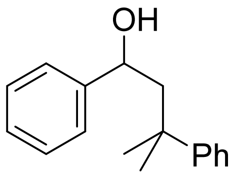

3e

$C_{17}H_{20}O$

M = 240.35 g/mol

Prepared from styrene **1e** (10.4 mg, 0.1 mmol) and 4,4,5,5-tetramethyl-2-(2-phenylpropan-2-yl)-1,3,2-dioxaborolane **2a** (49.2 mg, 2.0 equiv.) according to General Procedure A followed by C–B bond oxidation. Purified by thin layer chromatography (PE/EtOAc = 10:1) to afford the product as colorless oil (16.5 mg, 69% yield). The spectral data matched those reported in the literature.[9]

^1H NMR (400 MHz, $CDCl_3$) δ 7.48 – 7.41 (m, 2H), 7.40 – 7.34 (m, 2H), 7.32 – 7.27 (m, 2H), 7.27 – 7.18 (m, 4H), 4.58 (dd, *J* = 8.7, 3.0 Hz, 1H), 2.20 (dd, *J* = 14.6, 8.7 Hz, 1H), 2.03 (dd, *J* = 14.6, 3.0 Hz, 1H), 1.60 (brs, 1H), 1.46 (s, 3H), 1.40 (s, 3H) ppm.

$^{13}C\{^1H\}$ NMR (101 MHz, $CDCl_3$) δ 148.8, 146.0, 128.5, 128.5, 127.4, 126.2, 126.1, 125.7, 72.5, 54.1, 37.7, 30.1, 29.3 ppm.

HRMS (ESI): calculated for $C_{17}H_{19}O^-$ $[M-H]^-$ 239.1441, found 239.1440.

3-methyl-3-phenyl-1-(p-tolyl)butan-1-ol (3f)

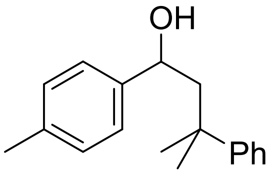

3f

$C_{18}H_{22}O$

M = 254.37 g/mol

Prepared from 1-methyl-4-vinylbenzene **1f** (10.4 mg, 0.1 mmol) and 4,4,5,5-tetramethyl-2-(2-phenylpropan-2-yl)-1,3,2-dioxaborolane **2a** (49.2 mg, 2.0 equiv.) according to General Procedure A followed by C–B bond oxidation. Purified by thin layer chromatography (PE/EtOAc = 10:1) to afford the product as colorless oil (20.7 mg, 69% yield). The spectral data matched those reported in the literature.[9]

^{1}H NMR (400 MHz, $CDCl_3$) δ 7.46 – 7.41 (m, 2H), 7.41 – 7.34 (m, 2H), 7.28 – 7.19 (m, 1H), 7.12 (s, 4H), 4.55 (dd, *J* = 8.5, 3.2 Hz, 1H), 2.34 (s, 3H), 2.20 (dd, *J* = 14.6, 8.5 Hz, 1H), 2.02 (dd, *J* = 14.6, 3.2 Hz, 1H), 1.46 (s, 3H), 1.39 (s, 3H) ppm.

^{13}C{^{1}H} NMR (101 MHz, $CDCl_3$) δ 148.9, 143.1, 137.0, 129.2, 128.5, 126.2, 126.0, 125.7, 72.3, 54.0, 37.7, 30.1, 29.3, 21.2 ppm.

HRMS (ESI): calculated for $C_{18}H_{21}O^-$ [M-H]$^-$ 253.1598, found 253.1600.

1-(4-(*tert*-butyl)phenyl)-3-methyl-3-phenylbutan-1-ol (3g)

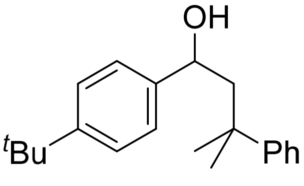

3g

$C_{21}H_{28}O$

M = 296.45 g/mol

Prepared from 1-(*tert*-butyl)-4-vinylbenzene **1g** (16.0 mg, 0.1 mmol) and 4,4,5,5-tetramethyl-2-(2-phenylpropan-2-yl)-1,3,2-dioxaborolane **2a** (49.2 mg, 2.0 equiv.) according to General Procedure A with One-pot Oxidation. Purified by thin layer chromatography (PE/EtOAc = 10:1) to afford the product as yellow oil (20.2 mg, 68% yield).

^{1}H NMR (400 MHz, $CDCl_3$) δ 7.43 (d, *J* = 7.6 Hz, 2H), 7.39 – 7.29 (m, 4H), 7.25 – 7.20 (m, 1H), 7.15 (d, *J* = 8.3 Hz, 2H), 4.56 (dd, *J* = 8.6, 2.9 Hz, 1H), 2.20 (dd, *J* = 14.6, 8.6 Hz, 1H), 2.04 (dd, *J* = 14.6, 2.9 Hz, 1H), 1.47 (s, 3H), 1.40 (s, 3H), 1.32 (s, 9H) ppm.

^{13}C{^{1}H} NMR (101 MHz, $CDCl_3$) δ 150.3, 148.9, 143.0, 128.5, 126.2, 126.0, 125.5, 125.4, 72.3, 53.9, 37.7, 34.6, 31.5, 29.9, 29.5 ppm.

HRMS (ESI): calculated for $C_{21}H_{28}ONa^+$ [M+Na]$^+$ 319.2032, found 319.2023.

3-methyl-1-(4-phenoxyphenyl)-3-phenylbutan-1-ol (3h)

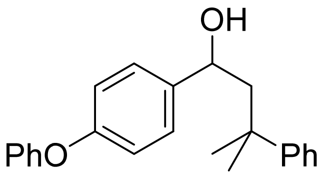

3h
$C_{23}H_{24}O_2$
M = 332.44 g/mol

Prepared from 1-phenoxy-4-vinylbenzene **1h** (19.6 mg, 0.1 mmol) and 4,4,5,5-tetramethyl-2-(2-phenylpropan-2-yl)-1,3,2-dioxaborolane **2a** (73.8 mg, 3.0 equiv.) according to General Procedure B followed by C–B bond oxidation. Purified by flash column chromatography (PE/EtOAc = 50:1) to afford the product as colorless oil (17.4 mg, 52% yield).

^{1}H NMR (400 MHz, $CDCl_3$) δ 7.42 (dd, *J* = 8.4, 1.2 Hz, 2H), 7.39 – 7.28 (m, 4H), 7.25 – 7.20 (m, 1H), 7.20 – 7.14 (m, 2H), 7.10 (t, *J* = 7.4 Hz, 1H), 6.99 (dd, *J* = 8.6, 1.0 Hz, 2H), 6.96 – 6.88 (m, 2H), 4.57 (dt, *J* = 8.2, 2.7 Hz, 1H), 2.21 (dd, *J* = 14.6, 8.4 Hz, 1H), 2.03 (dd, *J* = 14.6, 3.3 Hz, 1H), 1.46 (s, 3H), 1.43 (d, *J* = 2.7 Hz, 1H), 1.40 (s, 3H) ppm.

^{13}C{^{1}H} NMR (101 MHz, $CDCl_3$) δ 157.5, 156.4, 148.7, 140.9, 129.8, 128.6, 127.2, 126.1, 126.1, 123.3, 118.9, 118.9, 72.0, 54.1, 37.6, 30.1, 29.3 ppm.

HRMS (ESI): calculated for $C_{23}H_{25}O_3^-$ $[M+OH]^-$ 349.1809, found 349.1822.

1-(4-fluorophenyl)-3-methyl-3-phenylbutan-1-ol (3i)

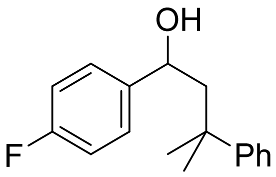

3i
$C_{17}H_{19}FO$
M = 258.34 g/mol

Prepared from 1-fluoro-4-vinylbenzene **1i** (12.2 mg, 0.1 mmol) and 4,4,5,5-tetramethyl-2-(2-phenylpropan-2-yl)-1,3,2-dioxaborolane **2a** (73.8 mg, 3.0 equiv.) according to General Procedure B with One-pot Oxidation. Purified by thin layer chromatography (PE/EtOAc = 10:1) to afford the product as pale yellow oil (19.0 mg, 73% yield).

^{1}**H NMR** (400 MHz, $CDCl_3$) δ 7.45 – 7.39 (m, 2H), 7.38 – 7.31 (m, 2H), 7.29 – 7.18 (m, 1H), 7.15 (ddd, *J* = 8.4, 5.3, 2.5 Hz, 2H), 7.01 – 6.92 (m, 2H), 4.56 (dd, *J* = 8.5, 3.2 Hz, 1H), 2.17 (dd, *J* = 14.6, 8.5 Hz, 1H), 1.98 (dd, *J* = 14.6, 3.2 Hz, 1H), 1.58 (brs, 1H), 1.45 (s, 3H), 1.38 (s, 3H) ppm.

^{13}**C{^{1}H} NMR** (101 MHz, $CDCl_3$) δ 162.0 (d, *J* = 245.0 Hz), 148.4, 141.5, 128.5, 127.2 (d, *J* = 8.0 Hz), 126.0 (d, *J* = 4.2 Hz), 115.1 (d, *J* = 21.3 Hz), 71.7, 54.1, 37.5, 30.1, 29.1 ppm (with one peak overlap in downfield).

^{19}**F NMR** (376 MHz, $CDCl_3$) δ -115.59 ppm.

HRMS (ESI): calculated for $C_{17}H_{20}FO_2^-$ $[M+OH]^-$ 275.1453, found 275.1450.

1-(4-chlorophenyl)-3-methyl-3-phenylbutan-1-ol (3j)

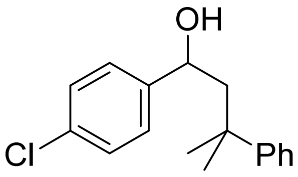

3j

$C_{17}H_{19}ClO$

M = 274.79 g/mol

Prepared from 1-chloro-4-vinylbenzene **1j** (13.8 mg, 0.1 mmol) and 4,4,5,5-tetramethyl-2-(2-phenylpropan-2-yl)-1,3,2-dioxaborolane **2a** (73.8 mg, 3.0 equiv.) according to General Procedure B with One-pot Oxidation. Purified by thin layer chromatography (PE/EtOAc = 10:1) to afford the product as pale yellow oil (11.3 mg, 41% yield). The spectral data matched those reported in the literature.[9]

^{1}**H NMR** (400 MHz, $CDCl_3$) δ 7.44 – 7.39 (m, 2H), 7.35 (t, *J* = 7.7 Hz, 2H), 7.28 – 7.22 (m, 3H), 7.12 (d, *J* = 8.4 Hz, 2H), 4.55 (dt, *J* = 8.8, 2.7 Hz, 1H), 2.15 (dd, *J* = 14.6, 8.6 Hz, 1H), 1.96 (dd, *J* = 14.6, 3.0 Hz, 1H), 1.56 (s, 1H), 1.45 (s, 3H), 1.39 (s, 3H) ppm.

^{13}**C{^{1}H} NMR** (101 MHz, $CDCl_3$) δ 148.5, 144.4, 132.9, 128.6, 128.6, 127.2, 126.2, 126.1, 71.9, 54.2, 37.6, 30.2, 29.2 ppm.

HRMS (ESI): calculated for $C_{17}H_{19}Cl_2O^-$ $[M+Cl]^-$ 309.0818, found 309.0825.

1-(2-fluorophenyl)-3-methyl-3-phenylbutan-1-ol (3k)

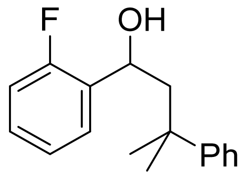

3k
$C_{17}H_{19}FO$
M = 258.34 g/mol

Prepared from 1-fluoro-2-vinylbenzene **1k** (12.2 mg, 0.1 mmol) and 4,4,5,5-tetramethyl-2-(2-phenylpropan-2-yl)-1,3,2-dioxaborolane **2a** (73.8 mg, 3.0 equiv.) according to General Procedure B with One-pot Oxidation. Purified by thin layer chromatography (PE/EtOAc = 10:1) to afford the product as colorless oil (12.0 mg, 46% yield).

^{1}H NMR (400 MHz, $CDCl_3$) δ 7.47 – 7.41 (m, 2H), 7.35 (t, *J* = 7.7 Hz, 3H), 7.26 – 7.14 (m, 2H), 7.08 (t, *J* = 7.0 Hz, 1H), 7.00 – 6.91 (m, 1H), 4.95 (d, *J* = 8.8 Hz, 1H), 2.17 (dd, *J* = 14.6, 8.8 Hz, 1H), 2.05 (dd, *J* = 14.6, 2.3 Hz, 1H), 1.57 (brs, 1H), 1.45 (s, 6H) ppm.

^{13}C{^{1}H} NMR (101 MHz, $CDCl_3$) δ 159.5 (d, *J* = 245.5 Hz), 148.6, 132.7 (d, *J* = 13.0 Hz), 128.6 (d, *J* = 8.9 Hz), 128.6, 127.3 (d, *J* = 4.4 Hz), 126.2, 126.1, 124.3 (d, *J* = 3.1 Hz), 115.3 (d, *J* = 21.7 Hz), 66.7, 53.0, 37.7, 30.3, 28.8 ppm.

^{19}F NMR (376 MHz, $CDCl_3$) δ -119.27 ppm.

HRMS (ESI): calculated for $C_{17}H_{20}FO_2^-$ $[M+OH]^-$ 275.1453, found 275.1457.

3-methyl-3-phenyl-1-(4-(trifluoromethoxy)phenyl)butan-1-ol (3l)

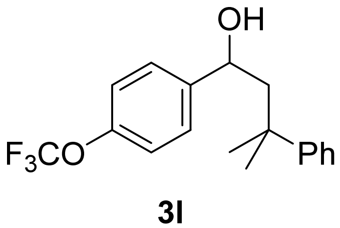

3l
$C_{18}H_{19}F_3O_2$
M = 324.34 g/mol

Prepared from 1-(trifluoromethoxy)-4-vinylbenzene **1l** (18.8 mg, 0.1 mmol) and 4,4,5,5-tetramethyl-2-(2-phenylpropan-2-yl)-1,3,2-dioxaborolane **2a** (73.8 mg, 3.0 equiv.) according to General Procedure B with

One-pot Oxidation. Purified by thin layer chromatography (PE/EtOAc = 10:1) to afford the product as colorless oil (13.1 mg, 40% yield).

^{1}H NMR (400 MHz, $CDCl_3$) δ 7.41 (dd, *J* = 8.4, 1.2 Hz, 2H), 7.38 – 7.32 (m, 2H), 7.25 – 7.18 (m, 3H), 7.11 (d, *J* = 8.1 Hz, 2H), 4.60 (d, *J* = 8.6 Hz, 1H), 2.16 (dd, *J* = 14.6, 8.6 Hz, 1H), 1.99 (dd, *J* = 14.6, 3.1 Hz, 1H), 1.57 (brs, 1H), 1.46 (s, 3H), 1.40 (s, 3H) ppm.

^{13}C{^{1}H} NMR (101 MHz, $CDCl_3$) δ 148.4, 148.3 (q, *J* = 1.9 Hz), 144.6, 128.6, 127.1, 126.2, 126.1, 121.0, 120.6 (q, *J* = 256.8 Hz), 71.8, 54.3, 37.6, 30.1, 29.3 ppm.

^{19}F NMR (376 MHz, $CDCl_3$) δ -57.89 ppm.

HRMS (ESI): calculated for $C_{18}H_{20}F_3O_3^-$ $[M+OH]^-$ 341.1370, found 341.1364.

4-(4-(3-methyl-3-phenyl-1-(4,4,5,5-tetramethyl-1,3,2-dioxaborolan-2-yl)butyl)phenyl)morpholine (3m)

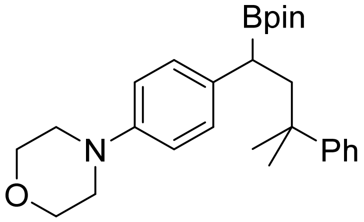

3m
$C_{27}H_{38}BNO_3$
M = 435.41 g/mol

Prepared from 4-(4-vinylphenyl)morpholine **1m** (18.9 mg, 0.1 mmol) and 4,4,5,5-tetramethyl-2-(2-phenylpropan-2-yl)-1,3,2-dioxaborolane **2a** (29.5 mg, 1.2 equiv.) according to General Procedure A. Purified by flash column chromatography (PE/EtOAc = 10:1) to afford the product as colorless oil (29.9 mg, 69% yield).

^{1}H NMR (400 MHz, $CDCl_3$) δ 7.33 (dd, *J* = 8.4, 1.5 Hz, 2H), 7.27 (t, *J* = 7.7 Hz, 2H), 7.19 – 7.10 (m, 1H), 7.03 – 6.95 (m, 2H), 6.81 – 6.73 (m, 2H), 3.88 – 3.81 (m, 4H), 3.10 (dd, *J* = 5.9, 3.8 Hz, 4H), 2.37 (dd, *J* = 13.5, 9.4 Hz, 1H), 2.08 (dd, *J* = 9.4, 3.4 Hz, 1H), 1.93 (dd, *J* = 13.5, 3.4 Hz, 1H), 1.33 (s, 3H), 1.26 (s, 3H), 1.11 (s, 6H), 1.10 (s, 6H) ppm.

^{11}B NMR (128 MHz, $CDCl_3$) δ 33.99 ppm.

^{13}C{^{1}H} NMR (101 MHz, $CDCl_3$) δ 149.5, 148.8, 136.4, 128.9, 128.1, 126.2, 125.5, 116.1, 83.3, 67.1, 49.9, 47.1, 38.9, 30.1, 28.4, 27.4, 24.8, 24.5 ppm.

HRMS (ESI): calculated for $C_{27}H_{37}BNO_3^-$ [M-H]$^-$ 434.2872, found 434.2854.

2-(1-(2,3-dihydrobenzofuran-5-yl)-3-methyl-3-phenylbutyl)-4,4,5,5-tetramethyl-1,3,2-dioxaborolane (3n)

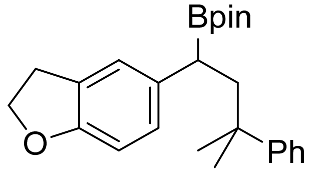

3n
$C_{25}H_{33}BO_3$
M = 392.35 g/mol

Prepared from 5-vinyl-2,3-dihydrobenzofuran **1n**(14.6 mg, 0.1 mmol) and 4,4,5,5-tetramethyl-2-(2-phenylpropan-2-yl)-1,3,2-dioxaborolane **2a** (29.5 mg, 1.2 equiv.) according to General Procedure A. Purified by flash column chromatography (PE/EtOAc = 20:1) to afford the product as colorless oil (27.0 mg, 68% yield).

^{1}H NMR (400 MHz, $CDCl_3$) δ 7.35 – 7.29 (m, 2H), 7.30 – 7.22 (m, 2H), 7.18 – 7.10 (m, 1H), 6.86 (s, 1H), 6.81 (d, *J* = 8.2 Hz, 1H), 6.60 (d, *J* = 8.2 Hz, 1H), 4.49 (t, *J* = 8.6 Hz, 2H), 3.10 (t, *J* = 8.6 Hz, 2H), 2.35 (dd, *J* = 13.4, 9.3 Hz, 1H), 2.07 (dd, *J* = 9.3, 3.5Hz, 1H), 1.92 (dd, *J* = 13.4, 3.5 Hz, 1H), 1.32 (s, 3H), 1.26 (s, 3H), 1.11 (s, 6H), 1.10 (s, 6H) ppm.

^{11}B NMR (128 MHz, $CDCl_3$) δ 33.50 ppm.

^{13}C{^{1}H} NMR (101 MHz, $CDCl_3$) δ 157.8, 149.5, 136.6, 128.1, 127.6, 126.9, 126.2, 125.5, 124.8, 109.0, 83.3, 71.1, 47.5, 38.9, 30.2, 30.0, 28.3, 27.7, 24.8, 24.5 ppm.

HRMS (ESI): calculated for $C_{25}H_{34}BO_3^+$ [M+H]$^+$ 393.2596, found 393.2588.

1-methyl-5-(3-methyl-3-phenyl-1-(4,4,5,5-tetramethyl-1,3,2-dioxaborolan-2-yl)butyl)-1H-indole (3o)

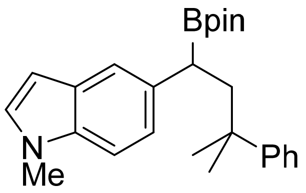

3o
$C_{26}H_{34}BNO_2$
M = 403.37 g/mol

Prepared from 1-methyl-5-vinyl-1*H*-indole **1o** (15.7 mg, 0.1 mmol) and 4,4,5,5-tetramethyl-2-(2-phenylpropan-2-yl)-1,3,2-dioxaborolane **2a** (29.5 mg, 1.2 equiv.) according to General Procedure A. Purified by flash column chromatography (PE/EtOAc = 20:1) to afford the product as white solid (30.7 mg, 76% yield).

^{1}H NMR (400 MHz, $CDCl_3$) δ 7.42 – 7.34 (m, 3H), 7.30 (t, *J* = 7.7 Hz, 2H), 7.21 – 7.15 (m, 2H), 7.04 – 6.95 (m, 2H), 6.38 (d, *J* = 2.9 Hz, 1H), 3.75 (s, 3H), 2.49 (dd, *J* = 13.5, 9.5 Hz, 1H), 2.25 (dd, *J* = 9.5, 3.1 Hz, 1H), 2.04 (dd, *J* = 13.5, 3.1 Hz, 1H), 1.37 (s, 3H), 1.30 (s, 3H), 1.12 (s, 12H) ppm.

^{11}B NMR (128 MHz, $CDCl_3$) δ 34.23 ppm.

^{13}C{^{1}H} NMR (101 MHz, $CDCl_3$) δ 149.7, 135.5, 135.2, 128.9, 128.6, 128.1, 126.2, 125.5, 122.7, 119.9, 109.0, 100.6, 83.2, 47.8, 39.0, 32.9, 30.1, 28.5, 24.8, 24.5 ppm.

HRMS (ESI): calculated for $C_{26}H_{35}BNO_2^+$ $[M+H]^+$ 404.2755, found 404.2746.

2-(1-(4-methoxyphenyl)-3,3-diphenylbutyl)-4,4,5,5-tetramethyl-1,3,2-dioxaborolane (3p)

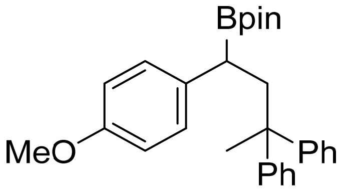

3p
$C_{29}H_{35}BO_3$
M = 442.41 g/mol

Prepared from 1-methoxy-4-vinylbenzene **1a** (13.4 mg, 0.1 mmol) and 2-(1,1-diphenylethyl)-4,4,5,5-tetramethyl-1,3,2-dioxaborolane **2p** (37.0 mg, 1.2 equiv.) according to General Procedure A at 80 ^oC. Purified by thin layer chromatography (PE/EtOAc = 50:1) to afford the product as white solid (23.6 mg, 53% yield, m.p. = 101.4 – 104.5 ^oC) ppm.

^{1}**H NMR** (400 MHz, $CDCl_3$) δ 7.28 – 7.19 (m, 8H), 7.19 – 7.13 (m, 2H), 7.05 – 6.96 (m, 2H), 6.81 – 6.73 (m, 2H), 3.77 (s, 3H), 2.92 (dd, *J* = 13.4, 9.0 Hz, 1H), 2.41 (dd, *J* = 13.4, 2.9 Hz, 1H), 2.16 (dd, *J* = 9.0, 2.9 Hz, 1H), 1.65 (s, 3H), 1.12 (s, 6H), 1.10 (s, 6H) ppm.

^{11}**B NMR** (128 MHz, $CDCl_3$) δ 33.95 ppm.

^{13}**C{^{1}H} NMR** (101 MHz, $CDCl_3$) δ 157.4, 150.3, 148.9, 136.8, 129.2, 128.1, 127.8, 127.5, 113.9, 83.4, 55.3, 47.4, 44.8, 27.6, 24.7, 24.6 ppm. (The carbon attached to boron was not observed due to quadrupolar relaxation.)

HRMS (ESI): calculated for $C_{29}H_{33}BO_3Na^+$ $[M+Na]^+$ 463.2415, found 463.2403.

2-(1-(4-methoxyphenyl)-3-methyl-3-phenylheptyl)-4,4,5,5-tetramethyl-1,3,2-dioxaborolane (3q)

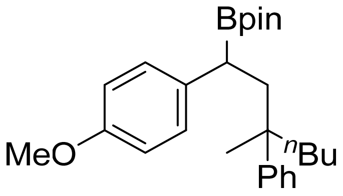

3q

$C_{27}H_{39}BO_3$

M = 422.42 g/mol

Prepared from 1-methoxy-4-vinylbenzene **1a** (13.4 mg, 0.1 mmol) and 4,4,5,5-tetramethyl-2-(2-phenylhexan-2-yl)-1,3,2-dioxaborolane **2q** (34.6 mg, 1.2 equiv.) according to General Procedure A at 80 °C. Purified by flash column chromatography (PE/EtOAc = 50:1) to afford the product as colorless oil (22.5 mg, 62% yield, d.r. = 1.2:1, determined by ^{1}H NMR).

^{1}**H NMR** (400 MHz, $CDCl_3$) major isomer: δ 7.35 – 7.24 (m, 4H), 7.21 – 7.11 (m, 1H), 7.01 – 6.92 (m, 2H), 6.80 – 6.71 (m, 2H), 3.77 (s, 3H), 2.34 (dd, *J* = 13.2, 9.9 Hz, 1H), 2.01 (m, 2H), 1.61 – 1.46 (m, 2H), 1.34 (s, 3H), 1.22 – 1.17 (m, 2H), 1.15 (s, 6H), 1.14 (s, 6H), 0.96 – 0.85 (m, 2H), 0.80 (t, *J* = 7.2 Hz, 3H); minor isomer: δ 7.35 – 7.24 (m, 4H), 7.21 – 7.11 (m, 1H), 7.08 – 7.01 (m, 2H), 6.80 – 6.71 (m, 2H), 3.77 (s, 3H), 2.45 (dd, *J* = 13.6, 8.2 Hz, 1H), 2.18 (dd, *J* = 8.2, 4.4 Hz, 1H), 1.86 (dd, *J* = 13.6, 4.4 Hz, 1H), 1.80 – 1.63 (m, 2H), 1.23 (s, 3H), 1.22 – 1.17 (m, 2H), 1.09 (s, 6H), 1.06 (s, 6H), 0.96 – 0.85 (m, 2H), 0.81 (t, *J* = 7.2 Hz, 3H) ppm.

^{11}**B NMR** (128 MHz, $CDCl_3$) major isomer: δ 34.73; minor isomer: δ 34.73 ppm.

^{13}**C{^{1}H} NMR** (101 MHz, $CDCl_3$) major isomer: δ 157.3, 147.7, 136.9, 129.1, 128.0, 126.8, 125.4, 113.8, 83.3, 55.3, 46.6, 44.0, 42.1, 26.9, 26.6, 24.7, 24.6, 23.9, 23.5, 14.2; minor isomer: δ 157.3, 148.0, 137.0,

129.2, 128.1, 126.8, 125.4, 113.8, 83.2, 55.3, 46.6, 42.6, 42.0, 26.9, 26.6, 24.8, 24.5, 23.9, 23.6, 14.2 ppm.

HRMS (ESI): calculated for $C_{27}H_{39}BO_3Na^+$ $[M+Na]^+$ 445.2884, found 445.2877.

1-(4-methoxyphenyl)-3-methyl-3-(m-tolyl)butan-1-ol (3r)

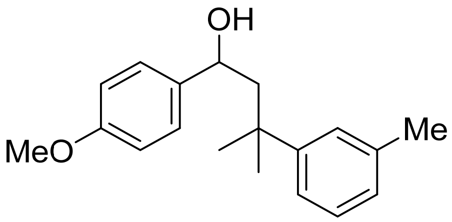

3r
$C_{19}H_{24}O_2$
M = 284.40 g/mol

Prepared from 1-methoxy-4-vinylbenzene **1a** (13.4 mg, 0.1 mmol) and 4,4,5,5-tetramethyl-2-(2-(m-tolyl)propan-2-yl)-1,3,2-dioxaborolane **2r** (34.6 mg, 1.2 equiv.) according to General Procedure A at 80 ^oC with One-pot Oxidation. Purified by thin layer chromatography (PE/EtOAc = 10:1) to afford the product as colorless oil (18.7 mg, 66% yield).

^{1}H NMR (400 MHz, $CDCl_3$) δ 7.28 – 7.18 (m, 3H), 7.17 – 7.10 (m, 2H), 7.03 (d, *J* = 6.7 Hz, 1H), 6.86 – 6.78 (m, 2H), 4.54 (dd, *J* = 8.2, 3.1 Hz, 1H), 3.78 (s, 3H), 2.37 (s, 3H), 2.18 (dd, *J* = 14.5, 8.4 Hz, 1H), 1.99 (dd, *J* = 14.5, 3.4 Hz, 1H), 1.42 (s, 3H), 1.36 (s, 3H) ppm.

^{13}C{^{1}H} NMR (101 MHz, $CDCl_3$) δ 158.9, 148.8, 138.2, 137.9, 128.4, 127.0, 126.9, 126.8, 123.2, 113.8, 72.0, 55.4, 54.0, 37.5, 30.3, 29.2, 21.8 ppm.

HRMS (ESI): calculated for $C_{19}H_{23}O_2^-$ $[M-H]^-$ 283.1704, found 283.1706.

2-(1-(4-methoxyphenyl)-3-methyl-3-(p-tolyl)butyl)-4,4,5,5-tetramethyl-1,3,2-dioxaborolane (3s)

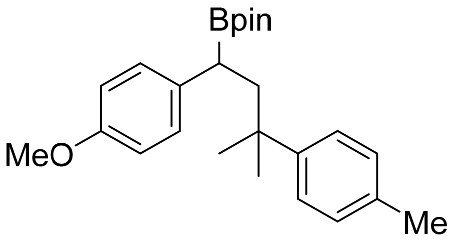

3s
$C_{25}H_{35}BO_3$
M = 394.36 g/mol

Prepared from 1-methoxy-4-vinylbenzene **1a** (13.4 mg, 0.1 mmol) and 4,4,5,5-tetramethyl-2-(2-(*p*-tolyl)propan-2-yl)-1,3,2-dioxaborolane **2s** (34.6 mg, 1.2 equiv.) according to General Procedure A at 80 °C. Purified by flash column chromatography (PE/EtOAc = 50:1) to afford the product as colorless oil (14.7 mg, 37% yield).

^{1}H NMR (400 MHz, CDCl$_3$) δ 7.26 – 7.17 (m, 2H), 7.12 – 7.05 (m, 2H), 7.04 – 6.96 (m, 2H), 6.79 – 6.70 (m, 2H), 3.76 (s, 3H), 2.35 (dd, *J* = 13.4, 9.3 Hz, 1H), 2.31 (s, 3H), 2.10 (dd, *J* = 9.3, 3.5 Hz, 1H), 1.91 (dd, *J* = 13.4, 3.5 Hz, 1H), 1.31 (s, 3H), 1.24 (s, 3H), 1.10 (s, 6H), 1.10 (s, 6H) ppm.

^{11}B NMR (128 MHz, CDCl$_3$) δ 34.26 ppm.

^{13}C{^{1}H} NMR (101 MHz, CDCl$_3$) δ 157.3, 146.4, 136.8, 134.9, 129.1, 128.8, 126.1, 113.8, 83.3, 55.3, 47.2, 38.5, 30.1, 28.6, 24.7, 24.5, 21.0 ppm. (The carbon attached to boron was not observed due to quadrupolar relaxation.)

HRMS (ESI): calculated for $C_{25}H_{36}BO_3^+$ [M+H]$^+$ 395.2752, found 395.2747.

2-(3-(3-chlorophenyl)-1-(4-methoxyphenyl)-3-methylbutyl)-4,4,5,5-tetramethyl-1,3,2-dioxaborolane (3t)

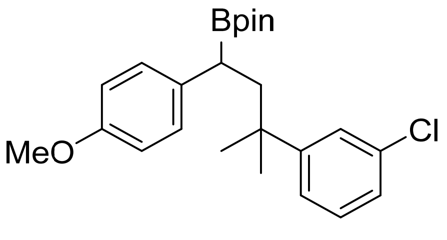

3t
$C_{24}H_{32}BClO_3$
M = 414.78 g/mol

Prepared from 1-methoxy-4-vinylbenzene **1a** (13.4 mg, 0.1 mmol) and 2-(2-(3-chlorophenyl)propan-2-yl)-4,4,5,5-tetramethyl-1,3,2-dioxaborolane **2t** (33.6 mg, 1.2 equiv.) according to General Procedure A at 80 °C with One-pot Oxidation. Purified by flash column chromatography (PE/EtOAc = 50:1) to afford the product as colorless oil (16.2 mg, 39% yield).

^{1}H NMR (400 MHz, $CDCl_3$) δ 7.31 – 7.25 (m, 1H), 7.22 – 7.17 (m, 2H), 7.17 – 7.09 (m, 1H), 7.02 – 6.94 (m, 2H), 6.78 – 6.70 (m, 2H), 3.76 (s, 3H), 2.34 (dd, *J* = 13.6, 8.9 Hz, 1H), 2.07 (dd, *J* = 8.9, 4.0 Hz, 1H), 1.92 (dd, *J* = 13.6, 4.0 Hz, 1H), 1.30 (s, 3H), 1.24 (s, 3H), 1.11 (s, 6H), 1.10 (s, 6H) ppm.

^{11}B NMR (128 MHz, $CDCl_3$) δ 34.33 ppm.

^{13}C{^{1}H} NMR (101 MHz, $CDCl_3$) δ 157.4, 151.7, 136.3, 134.1, 129.4, 129.1, 126.7, 125.7, 124.5, 113.9, 83.4, 55.3, 47.0, 39.1, 29.9, 28.4, 27.5, 24.8, 24.5 ppm.

HRMS (ESI): calculated for $C_{24}H_{33}BClO_3^+$ $[M+H]^+$ 415.2206, found 415.2201.

1-(4-methoxyphenyl)-3-phenylbutan-1-ol (3u)

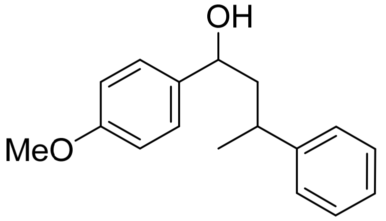

3u
$C_{17}H_{20}O_2$
M = 256.34 g/mol

Prepared from 1-methoxy-4-vinylbenzene **1a** (13.4 mg, 0.1 mmol) and 4,4,5,5-tetramethyl-2-(1-phenylethyl)-1,3,2-dioxaborolane **2u** (27.8 mg, 1.2 equiv.) according to General Procedure A at 80 °C with One-pot Oxidation. Purified by thin layer chromatography (PE/EtOAc = 10:1) to afford the product as colorless oil (13.5 mg, 53% yield, d.r. = 1.1:1, determined by ^{1}H NMR).

^{1}H NMR (400 MHz, $CDCl_3$) major isomer: δ 7.35 – 7.28 (m, 2H), 7.25 – 7.16 (m, 5H), 6.93 – 6.81 (m, 2H), 4.50 (t, *J* = 7.1 Hz, 1H), 3.81 (s, 3H), 2.75 – 2.61 (m, 1H), 2.19 (ddd, *J* = 13.6, 8.4, 7.1 Hz, 1H), 1.99 – 1.84 (m, 1H), 1.74 (brs, 1H), 1.26 (d, *J* = 6.9 Hz, 3H); minor isomer: δ 7.35 – 7.28 (m, 2H), 7.25 – 7.16 (m, 5H), 6.93 – 6.81 (m, 2H), 4.38 (dd, *J* = 9.4, 4.1 Hz, 1H), 3.79 (s, 3H), 2.99 (dqd, *J* = 9.7, 7.0, 5.2 Hz, 1H), 2.11 – 1.99 (m, 1H), 1.99 – 1.84 (m, 1H), 1.74 (brs, 1H), 1.29 (d, *J* = 7.0 Hz, 3H) ppm.

$^{13}C\{^1H\}$ NMR (101 MHz, $CDCl_3$) major isomer: δ 159.1, 146.8, 137.4, 128.6, 127.3, 127.1, 126.2, 114.0, 72.0, 55.4, 47.6, 36.8, 23.1; minor isomer: δ 159.3, 147.1, 136.8, 128.7, 127.6, 127.2, 126.2, 114.0, 72.6, 55.4, 47.2, 36.8, 22.8 ppm.

HRMS (ESI): calculated for $C_{17}H_{20}O_2Na^+$ $[M+Na]^+$ 279.1356, found 279.1344.

3-(3-methoxyphenyl)-1-(4-methoxyphenyl)butan-1-ol (3v)

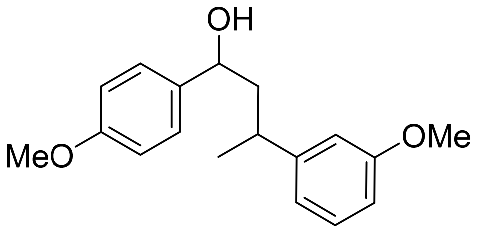

3v
$C_{18}H_{22}O_3$
M = 286.37 g/mol

Prepared from 1-methoxy-4-vinylbenzene **1a** (13.4 mg, 0.1 mmol) and 2-(1-(3-methoxyphenyl)ethyl)-4,4,5,5-tetramethyl-1,3,2-dioxaborolane **2v** (31.4 mg, 1.2 equiv.) according to General Procedure A at 80 °C with One-pot Oxidation. Purified by thin layer chromatography (PE/EtOAc = 10:1) to afford the product as colorless oil (18.9 mg, 66% yield, d.r. = 1.1:1, determined by ^1H NMR).

^1H NMR (400 MHz, $CDCl_3$) major isomer: δ 7.35 – 7.14 (m, 3H), 6.91 – 6.82 (m, 3H), 6.82 – 6.73 (m, 2H), 4.40 (dt, *J* = 9.2, 3.4 Hz, 1H), 3.81 (s, 3H), 3.79 (s, 3H), 2.97 (dqd, *J* = 9.7, 6.9, 5.1 Hz, 1H), 2.03 (ddd, *J* = 13.9, 9.4, 5.3 Hz, 1H), 1.96 – 1.86 (m, 1H), 1.77 (d, *J* = 3.4 Hz, 1H), 1.28 (d, *J* = 7.0 Hz, 3H); minor isomer: δ 7.35 – 7.14 (m, 3H), 6.91 – 6.82 (m, 3H), 6.82 – 6.73 (m, 2H), 4.51 (td, *J* = 7.0, 2.1 Hz, 1H), 3.81 (s, 3H), 3.81 (s, 3H), 2.66 (dp, *J* = 8.5, 6.9 Hz, 1H), 2.18 (ddd, *J* = 13.7, 8.5, 7.0 Hz, 1H), 1.96 – 1.86 (m, 1H), 1.73 (d, *J* = 2.9 Hz, 1H), 1.25 (d, *J* = 6.9 Hz, 3H) ppm.

$^{13}C\{^1H\}$ NMR (101 MHz, $CDCl_3$) major isomer: δ 159.9, 159.1, 148.6, 137.4, 129.6, 127.1, 119.7, 113.9, 113.3, 111.2, 72.0, 55.3, 47.5, 36.8, 23.0; minor isomer: 159.9, 159.3, 148.9, 136.8, 129.6, 127.6, 119.6, 114.0, 113.1, 111.4, 72.6, 55.4, 47.2, 36.9, 22.7 ppm.

HRMS (ESI): calculated for $C_{18}H_{23}O_4^-$ $[M+OH]^-$ 303.1602, found 303.1607.

3-(4-(tert-butyl)phenyl)-1-(4-methoxyphenyl)butan-1-ol (3w)

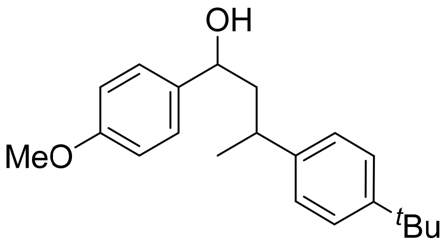

3w
$C_{21}H_{28}O_2$
M = 312.45 g/mol

Prepared from 1-methoxy-4-vinylbenzene **1a** (13.4 mg, 0.1 mmol) and 2-(1-(4-(*tert*-butyl)phenyl)ethyl)-4,4,5,5-tetramethyl-1,3,2-dioxaborolane **2w** (34.6 mg, 1.2 equiv.) according to General Procedure A at 80 °C with One-pot Oxidation. Purified by thin layer chromatography (PE/EtOAc = 10:1) to afford the product as colorless oil (17.6 mg, 56% yield, d.r. = 1:1, determined by ^{1}H NMR).

^{1}H NMR (400 MHz, $CDCl_3$) major isomer: δ 7.36 – 7.30 (m, 2H), 7.25 – 7.19 (m, 2H), 7.18 – 7.11 (m, 2H), 6.93 – 6.81 (m, 2H), 4.53 (t, *J* = 7.0 Hz, 1H), 3.81 (s, 3H), 2.69 (dt, *J* = 8.2, 6.7 Hz, 1H), 2.17 (ddd, *J* = 13.6, 8.2, 7.3 Hz, 1H), 1.99 – 1.86 (m, 1H), 1.76 (brs, 1H), 1.33 (s, 9H), 1.26 (d, *J* = 7.1 Hz, 3H); minor isomer: 7.36 – 7.30 (m, 2H), 7.25 – 7.19 (m, 2H), 7.18 – 7.11 (m, 2H), 6.93 – 6.81 (m, 2H), 4.43 (dd, *J* = 9.1, 4.3 Hz, 1H), 3.80 (s, 3H), 3.01 – 2.88 (m, 1H), 2.10 – 1.99 (m, 1H), 1.99 – 1.86 (m, 1H), 1.76 (brs, 1H), 1.33 (s, 9H), 1.28 (d, *J* = 7.0 Hz, 3H) ppm.

^{13}C{^{1}H} NMR (101 MHz, $CDCl_3$) major isomer: δ 159.2, 149.0, 144.0, 136.9, 127.6, 126.8, 125.5, 114.0, 72.6, 55.4, 47.5, 36.3, 34.5, 31.6, 22.7; minor isomer: δ 159.1, 148.9, 143.7, 137.4, 127.1, 126.9, 125.5, 113.9, 72.1, 55.4, 47.5, 36.2, 34.5, 31.6, 23.1 ppm.

HRMS (ESI): calculated for $C_{21}H_{27}O_2^-$ $[M-H]^-$ 311.2017, found 311.2031.

1-(4-methoxyphenyl)-3-(4-(trimethylsilyl)phenyl)butan-1-ol (3x)

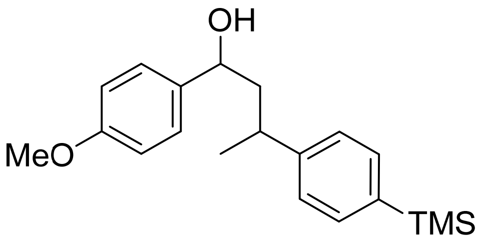

3x
$C_{20}H_{28}O_2Si$
M = 328.53 g/mol

Prepared from 1-methoxy-4-vinylbenzene **1a** (13.4 mg, 0.1 mmol) and trimethyl(4-(1-(4,4,5,5-tetramethyl-1,3,2-dioxaborolan-2-yl)ethyl)phenyl)silane **2x** (36.5 mg, 1.2 equiv.) according to General Procedure A at 80 °C with One-pot Oxidation. Purified by thin layer chromatography (PE/EtOAc = 10:1) to afford the product as colorless oil (26.4 mg, % yield, d.r. = 1:1, determined by ^{1}H NMR).

^{1}H NMR (400 MHz, $CDCl_3$) major isomer: δ 7.52 – 7.45 (m, 2H), 7.28 – 7.16 (m, 4H), 6.95 – 6.81 (m, 2H), 4.52 (t, *J* = 7.0 Hz, 1H), 3.82 (s, 3H), 2.70 (h, *J* = 7.0 Hz, 1H), 2.19 (ddd, *J* = 13.6, 8.1, 6.9 Hz, 1H), 1.99 – 1.89 (m, 1H), 1.72 (brs, 1H), 1.27 (d, *J* = 7.0 Hz, 3H), 0.28 (s, 9H); minor isomer: δ 7.52 –7.45 (m, 2H), 7.28 – 7.16 (m, 4H), 6.95 – 6.81 (m, 2H), 4.42 (dd, *J* = 9.1, 4.1 Hz, 1H), 3.80 (s, 3H), 2.98 (tdd, *J* = 6.9, 5.6, 2.6 Hz, 1H), 2.06 (ddd, *J* = 14.6, 9.2, 5.5 Hz, 1H), 1.99 – 1.89 (m, 1H), 1.76 (brs, 1H), 1.29 (d, *J* = 7.0 Hz, 3H), 0.28 (s, 9H) ppm.

^{13}C{^{1}H} NMR (101 MHz, $CDCl_3$) major isomer: δ 159.3, 147.8, 137.9, 136.8, 133.7, 127.6, 126.7, 114.0, 72.5, 55.4, 47.3, 36.7, 22.6, -0.9; minor isomer: δ 159.1, 147.4, 137.9, 137.4, 133.7, 127.1, 126.8, 113.9, 72.0, 55.4, 47.4, 36.7, 23.1, -0.9 ppm.

HRMS (ESI): calculated for $C_{20}H_{27}O_2Si^-$ [M-H]$^-$ 327.1786, found 327.1772

3-(4-fluorophenyl)-1-(4-methoxyphenyl)butan-1-ol (3y)

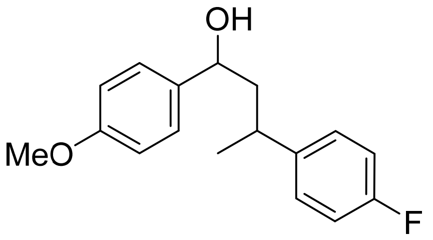

3y
$C_{17}H_{19}FO_2$
M = 274.34 g/mol

Prepared from 1-methoxy-4-vinylbenzene **1a** (13.4 mg, 0.1 mmol) and 2-(1-(4-fluorophenyl)ethyl)-4,4,5,5-tetramethyl-1,3,2-dioxaborolane **2y** (30.0 mg, 1.2 equiv.) according to General Procedure A at 80 °C with One-pot Oxidation. Purified by thin layer chromatography (PE/EtOAc = 10:1) to afford the product as colorless oil (14.0 mg, 51% yield, d.r. = 1:1, determined by ^{1}H NMR).

^{1}H NMR (400 MHz, $CDCl_3$) major isomer: δ 7.23 – 7.15 (m, 2H), 7.15 – 7.08 (m, 2H), 7.03 – 6.95 (m, 2H), 6.91 – 6.83 (m, 2H), 4.48 (t, *J* = 7.1 Hz, 1H), 3.81 (s, 3H), 2.67 (dt, *J* = 8.3, 6.7 Hz, 1H), 2.14 (ddd, *J* = 13.6, 8.3, 7.1 Hz, 1H), 1.96 – 1.79 (m, 1H), 1.73 (brs, 1H), 1.23 (d, *J* = 6.9 Hz, 2H); minor isomer: δ 7.23 – 7.15 (m, 4H), 7.03 – 6.95 (m, 2H), 6.91 – 6.83 (m, 2H), 4.34 (dd, *J* = 9.5, 4.0 Hz, 1H), 3.79 (s,

3H), 2.99 (dddd, J = 14.1, 9.8, 7.1, 5.1 Hz, 1H), 2.04 (ddd, J = 14.4, 9.5, 5.2 Hz, 1H), 1.96 – 1.79 (m, 1H), 1.73 (brs, 1H), 1.26 (d, J = 7.0 Hz, 3H) ppm.

^{19}F NMR (376 MHz, $CDCl_3$) major isomer: δ -117.36; minor isomer: δ -117.40 ppm.

^{13}C{^{1}H} NMR (101 MHz, $CDCl_3$) major isomer: δ 161.4 (d, J = 244.1 Hz), 159.4, 142.8, 136.6, 128.5 (d, J = 7.5 Hz), 127.6, 115.3 (d, J = 20.8 Hz), 114.1, 72.5, 55.4, 47.2, 36.0, 22.9; minor isomer: δ 161.4 (d, J = 244.1 Hz), 159.2, 142.5, 137.3, 128.6 (d, J = 7.7 Hz), 127.1, 115.3 (d, J = 20.8 Hz), 114.0, 72.0, 55.4, 47.7, 36.1, 23.1 ppm.

HRMS (ESI): calculated for $C_{17}H_{18}FO_2^-$ $[M-H]^-$ 273.1296, found 273.1296.

2-(3-([1,1'-biphenyl]-4-yl)-1-(4-methoxyphenyl)butyl)-4,4,5,5-tetramethyl-1,3,2-dioxaborolane (3z)

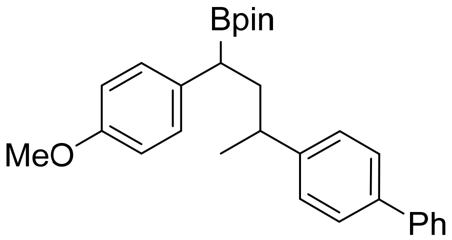

3z

$C_{29}H_{35}BO_3$

M = 442.41 g/mol

Prepared from 1-methoxy-4-vinylbenzene **1a** (13.4 mg, 0.1 mmol) and 2-(1-([1,1'-biphenyl]-4-yl)ethyl)-4,4,5,5-tetramethyl-1,3,2-dioxaborolane **2z** (29.5 mg, 1.2 equiv.) according to General Procedure A at 80 °C with One-pot Oxidation. Purified by flash column chromatography (PE/EtOAc = 50:1) to afford the product as colorless oil (24.7 mg, 56% yield, d.r. = 1:1, determined by ^{1}H NMR).

^{1}H NMR (400 MHz, $CDCl_3$) major isomer: δ 7.64 – 7.56 (m, 2H), 7.56 – 7.48 (m, 2H), 7.44 (m, 2H), 7.38 – 7.28 (m, 1H), 7.26 – 7.18 (m, 2H), 7.15 – 7.04 (m, 2H), 6.88 – 6.77 (m, 2H), 3.80 (s, 3H), 2.75 – 2.58 (m, 1H), 2.29 (t, J = 8.1 Hz, 1H), 2.23 – 2.05 (m, 1H), 2.03 – 1.91 (m, 1H), 1.25 (d, J = 6.9 Hz, 3H), 1.17 (s, 3H), 1.16 (s, 3H); minor isomer: δ 7.64 – 7.56 (m, 2H), 7.56 – 7.48 (m, 2H), 7.44 (m, 2H), 7.38 – 7.28 (m, 1H), 7.26 – 7.18 (m, 2H), 7.15 – 7.04 (m, 2H), 6.88 – 6.77 (m, 2H), 3.79 (s, 3H), 2.75 – 2.58 (m, 1H), 2.19 (dd, J = 9.2, 3.1 Hz, 0H), 2.23 – 2.05 (m, 1H), 2.03 – 1.91 (m, 1H), 1.30 (d, J = 6.9 Hz, 3H), 1.22 (s, 3H), 1.21 (s, 3H) ppm.

^{11}B NMR (128 MHz, $CDCl_3$) major isomer: δ 32.89; minor isomer: δ 32.89 ppm.

^{13}C{^{1}H} NMR (101 MHz, $CDCl_3$) major isomer: δ 157.5, 146.5, 141.4, 138.9, 135.0, 129.3, 128.8, 127.6, 127.2, 127.1, 127.0, 113.9, 83.3, 55.3, 41.5, 38.5, 29.1, 24.8, 23.1; minor isomer: δ 157.5, 147.1, 141.4,

138.9, 135.1, 129.6, 128.8, 127.9, 127.2, 127.1, 127.0, 113.9, 83.4, 55.3, 40.9, 38.2, 29.1, 24.7, 21.7 ppm.

HRMS (ESI): calculated for $C_{29}H_{36}BO_3^+$ $[M+H]^+$ 443.2752 found 443.2744.

1-(4-methoxyphenyl)-3-(naphthalen-1-yl)butan-1-ol (3aa)

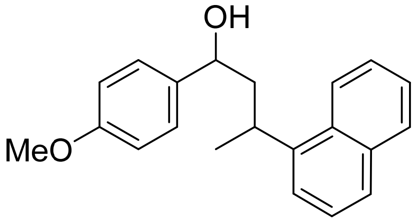

3aa
$C_{21}H_{22}O_2$
M = 306.40 g/mol

Prepared from 1-methoxy-4-vinylbenzene **1a** (13.4 mg, 0.1 mmol) and 4,4,5,5-tetramethyl-2-(1-(naphthalen-1-yl)ethyl)-1,3,2-dioxaborolane **2aa** (33.8 mg, 1.2 equiv.) according to General Procedure A at 80 °C with One-pot Oxidation. Purified by thin layer chromatography (PE/EtOAc = 10:1) to afford the product as colorless oil (20.7 mg, 68% yield, d.r. = 1.4:1 determined by ^{1}H NMR).

^{1}H NMR (400 MHz, $CDCl_3$) major isomer: δ 8.12 – 8.02 (m, 1H), 7.96 – 7.82 (m, 1H), 7.77 – 7.69 (m, 1H), 7.54 – 7.40 (m, 4H), 7.25 – 7.21 (m, 2H), 6.91 – 6.86 (m, 2H), 4.50 (dd, *J* = 7.9, 5.8 Hz, 1H), 3.91 – 3.80 (m, 1H), 3.81 (s, 3H), 2.27 – 2.11 (m, 1H), 1.98 (ddd, *J* = 13.7, 7.9, 5.8 Hz, 1H), 1.82 (brs, 1H), 1.43 (d, *J* = 6.8 Hz, 3H); minor isomer: δ 8.12 – 8.02 (m, 1H), 7.96 – 7.82 (m, 1H), 7.77 – 7.69 (m, 1H), 7.54 – 7.40 (m, 4H), 7.19 – 7.13 (m, 2H), 6.85 – 6.81 (m, 2H), 4.70 (dd, *J* = 8.1, 6.5 Hz, 1H), 3.80 (s, 3H), 3.78 – 3.69 (m, 1H), 2.40 (ddd, *J* = 13.7, 8.1, 6.5 Hz, 1H), 2.27 – 2.11 (m, 1H), 1.86 (brs, 1H), 1.42 (d, *J* = 6.8 Hz, 3H) ppm.

^{13}C{^{1}H} NMR (101 MHz, $CDCl_3$) major isomer: δ 159.2, 143.3, 137.3, 134.1, 132.0, 129.0, 127.2, 126.6, 125.9, 125.8, 125.5, 123.4, 122.9, 114.0, 72.3, 67.2, 55.4, 47.5, 30.4, 24.5, 22.8; minor isomer: δ 159.2, 143.4, 137.1, 134.1, 131.5, 129.0, 127.4, 126.6, 125.9, 125.8, 125.5, 123.5, 122.7, 114.0, 72.5, 67.2, 55.4, 47.1, 30.4, 24.5, 21.8 ppm.

HRMS (ESI): calculated for $C_{21}H_{21}O_2^-$ $[M-H]^-$ 305.1547, found 305.1557.

1-(4-methoxyphenyl)-3-(naphthalen-2-yl)butan-1-ol (3ab)

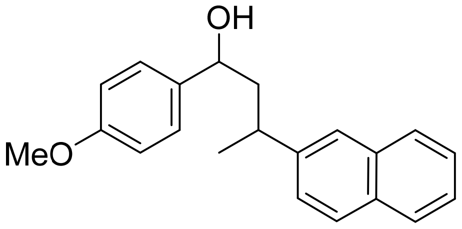

3ab
$C_{21}H_{22}O_2$
M = 306.40 g/mol

Prepared from 1-methoxy-4-vinylbenzene **1a** (13.4 mg, 0.1 mmol) and 4,4,5,5-tetramethyl-2-(1-(naphthalen-2-yl)ethyl)-1,3,2-dioxaborolane **2ab** (33.8 mg, 1.2 equiv.) according to General Procedure A at 80 ^{o}C with One-pot Oxidation. Purified by thin layer chromatography (PE/EtOAc = 10:1) to afford the product as colorless oil (27.5 mg, 90% yield, d.r. = 1:1 determined by ^{1}H NMR).

^{1}H NMR (400 MHz, $CDCl_3$) major isomer: δ 7.88 – 7.77 (m, 3H), 7.60 (s, 1H), 7.51 – 7.42 (m, 2H), 7.42 – 7.34 (m, 1H), 7.26 – 7.11 (m, 2H), 6.93 – 6.80 (m, 2H), 4.53 (t, *J* = 7.0 Hz, 1H), 3.82 (s, 3H), 2.86 (h, *J* = 6.9 Hz, 1H), 2.32 (ddd, *J* = 13.7, 8.4, 7.1 Hz, 1H), 2.08 – 1.96 (m, 1H), 1.75 (brs, 1H), 1.35 (d, *J* = 6.9 Hz, 3H); minor isomer: δ 7.88 – 7.77 (m, 3H), 7.69 (s, 1H), 7.51 – 7.42 (m, 2H), 7.42 – 7.34 (m, 1H), 7.26 – 7.11 (m, 2H), 6.93 – 6.80 (m, 2H), 4.40 (dd, *J* = 9.3, 3.5 Hz, 1H), 3.78 (s, 3H), 3.26 – 3.13 (m, 1H), 2.14 (ddd, *J* = 14.5, 9.5, 5.2 Hz, 1H), 2.08 – 1.96 (m, 1H), 1.79 (brs, 1H), 1.38 (d, *J* = 7.0 Hz, 3H) ppm.

^{13}C{^{1}H} NMR (101 MHz, $CDCl_3$) major isomer: δ 159.3, 144.6, 136.7, 133.8, 132.4, 128.3, 127.71, 127.68, 127.6, 126.0, 125.9, 125.40, 125.36, 114.0, 72.6, 55.4, 47.0, 36.9, 22.9; minor isomer: δ 159.1, 144.3, 137.4, 133.8, 132.4, 128.3, 127.71, 127.68, 127.1, 126.0, 125.76, 125.75, 125.4, 114.0, 72.0, 55.4, 47.5, 36.9, 23.1 ppm.

HRMS (ESI): calculated for $C_{21}H_{21}O_2^-$ [M-H]$^-$ 305.1547, found 305.1543

(E)-6-methyl-4,6-diphenylhept-3-en-1-ol (3ac)

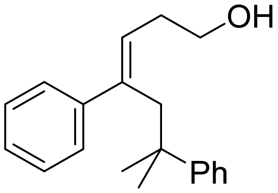

3ac
$C_{20}H_{24}O$
M = 280.41 g/mol

Prepared from (1-cyclopropylvinyl)benzene **1ac** (14.4 mg, 0.1 mmol) and 4,4,5,5-tetramethyl-2-(2-phenylpropan-2-yl)-1,3,2-dioxaborolane **2a** (29.5 mg, 1.2 equiv.) according to General Procedure A at 40 °C with One-pot Oxidation. Purified by thin layer chromatography (PE/EtOAc = 10:1) to afford the product as colorless oil (20.9 mg, 74% yield, E/Z > 20:1 determined by ^{1}H NMR).

The configuration of the product alkene was determined by ^{1}H-^{1}H NOESY analysis. As shown below, the NOE effect was observed between H atoms on *carbon a* and *carbon c*, which indicates an *E*-configuration. Besides, DFT computational analysis reveals that the formation of *E*-adduct is kinetically favorable ($\Delta\Delta G^\ddagger$ = 2.2 kcal mol^{-1}), which could also rationalize the formation of *E*-product (see **Figure S18** in Supporting Information). ^{1}H NMR spectra of alkene products also exhibit similar ^{1}H-^{1}H coupling constants. Therefore, the related products could be assigned as *E*-configuration as well.

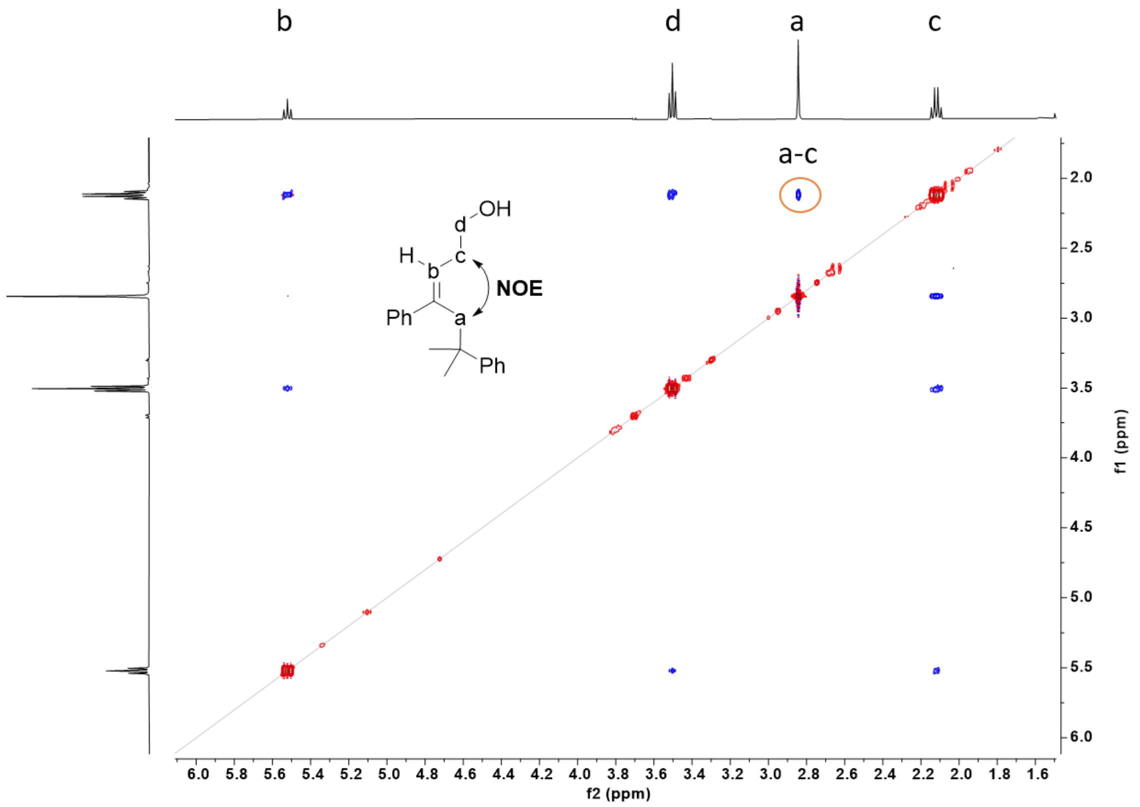

Figure S1. ^{1}H-^{1}H NOESY spectrum of **3ac**.

^{1}H NMR (400 MHz, $CDCl_3$) δ 7.35 – 7.30 (m, 2H), 7.28 – 7.17 (m, 7H), 7.17 – 7.11 (m, 1H), 5.54 (t, *J* = 7.3 Hz, 1H), 3.52 (t, *J* = 6.6 Hz, 2H), 2.86 (s, 2H), 2.14 (q, *J* = 6.6 Hz, 2H), 1.20 (s, 6H) ppm.

^{13}C{^{1}H} NMR (101 MHz, $CDCl_3$) δ 149.5, 145.6, 141.1, 128.9, 128.2, 127.9, 126.9, 126.5, 126.1, 125.7, 62.5, 44.2, 39.7, 32.8, 29.0 ppm.

HRMS (ESI): calculated for $C_{20}H_{23}O^-$ [M-H]$^-$ 279.1754, found 279.1759.

(E)-4-(3,5-dimethylphenyl)-6-methyl-6-phenylhept-3-en-1-ol (3ad)

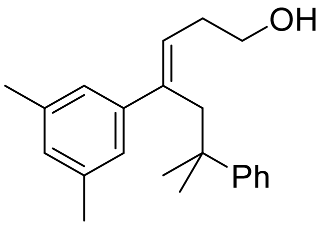

3ad
$C_{22}H_{28}O$
M = 308.46 g/mol

Prepared from 1-(1-cyclopropylvinyl)-3,5-dimethylbenzene **1ad** (17.2 mg, 0.1 mmol) and 4,4,5,5-tetramethyl-2-(2-phenylpropan-2-yl)-1,3,2-dioxaborolane **2a** (29.5 mg, 1.2 equiv.) according to General Procedure A at 40 °C with One-pot Oxidation. Purified by thin layer chromatography (PE/EtOAc = 10:1) to afford the product as colorless oil (21.0 mg, 68% yield, E/Z > 20:1 determined by ^{1}H NMR).

^{1}H NMR (400 MHz, $CDCl_3$) δ 7.36 – 7.30 (m, 2H), 7.25 (dd, *J* = 8.5, 6.9 Hz, 2H), 7.18 – 7.09 (m, 1H), 6.88 – 6.81 (m, 3H), 5.52 (t, *J* = 7.3 Hz, 1H), 3.52 (t, *J* = 6.6 Hz, 2H), 2.84 (s, 2H), 2.29 (s, 6H), 2.13 (q, *J* = 6.6 Hz, 2H), 1.21 (s, 6H) ppm.

^{13}C{^{1}H} NMR (101 MHz, $CDCl_3$) δ 149.6, 145.5, 141.4, 137.5, 128.2, 128.2, 127.8, 126.1, 125.6, 124.7, 62.5, 44.1, 39.7, 32.7, 29.0, 21.5 ppm.

HRMS (ESI): calculated for $C_{20}H_{29}O_2^-$ [M+OH]$^-$ 325.2173, found 325.2178.

(E)-4-(4-methoxyphenyl)-6-methyl-6-phenylhept-3-en-1-ol (3ae)

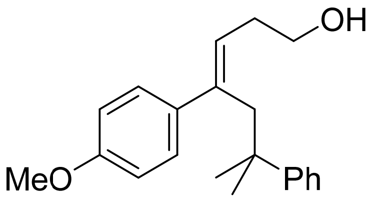

3ae
$C_{21}H_{26}O_2$
M = 310.44 g/mol

Prepared from 1-(1-cyclopropylvinyl)-4-methoxybenzene **1ae** (17.4 mg, 0.1 mmol) and 4,4,5,5-tetramethyl-2-(2-phenylpropan-2-yl)-1,3,2-dioxaborolane **2a** (29.5 mg, 1.2 equiv.) according to General Procedure A at 40 °C with One-pot Oxidation. Purified by thin layer chromatography (PE/EtOAc = 10:1) to afford the product as colorless oil (19.3 mg, 62% yield, E/Z > 20:1 determined by ^{1}H NMR).

^{1}**H NMR** (400 MHz, $CDCl_3$) δ 7.35 – 7.28 (m, 2H), 7.28 – 7.20 (m, 2H), 7.21 – 7.09 (m, 3H), 6.83 – 6.75 (m, 2H), 5.47 (t, *J* = 7.3 Hz, 1H), 3.80 (s, 3H), 3.50 (t, *J* = 6.6 Hz, 2H), 2.81 (s, 2H), 2.11 (q, *J* = 6.6 Hz, 2H), 1.19 (s, 6H) ppm.

^{13}**C{^{1}H} NMR** (101 MHz, $CDCl_3$) δ 158.5, 149.6, 140.5, 138.1, 127.9, 127.9, 127.5, 126.1, 125.6, 113.6, 62.5, 55.4, 44.2, 39.7, 32.8, 29.0 ppm.

HRMS (ESI): calculated for $C_{21}H_{26}O_2Na^+$ $[M+Na]^+$ 333.1825, found 333.1816.

(E)-4-([1,1'-biphenyl]-4-yl)-6-methyl-6-phenylhept-3-en-1-ol (3af)

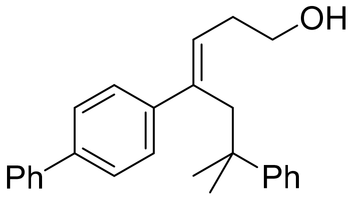

3af
$C_{26}H_{28}O$
M = 356.51 g/mol

Prepared from N,N-dimethyl-4-vinylaniline **1af** (22.0 mg, 0.1 mmol) and 4,4,5,5-tetramethyl-2-(2-phenylpropan-2-yl)-1,3,2-dioxaborolane **2a** (29.5 mg, 1.2 equiv.) according to General Procedure A at 40 °C with One-pot Oxidation. Purified by thin layer chromatography (PE/EtOAc = 10:1) to afford the product as colorless oil (27.4 mg, 77% yield, E/Z = 7.1:1 determined by ^{1}H NMR).

^{1}**H NMR** (400 MHz, $CDCl_3$) major isomer: δ 7.59 – 7.50 (m, 2H), 7.48 – 7.34 (m, 4H), 7.32 – 7.22 (m, 4H), 7.22 – 7.15 (m, 3H), 7.12 – 7.01 (m, 1H), 5.55 (t, *J* = 7.3 Hz, 1H), 3.48 (t, *J* = 6.6 Hz, 2H), 2.83 (s, 2H), 2.17 – 2.06 (m, 2H), 1.18 (s, 6H); minor isomer: δ 7.59 – 7.50 (m, 2H), 7.48 – 7.34 (m, 4H), 7.32 – 7.22 (m, 4H), 7.22 – 7.15 (m, 3H), 7.12 – 7.01 (m, 1H), 5.11 (t, *J* = 7.6 Hz, 1H), 3.42 (t, *J* = 6.3 Hz, 2H), 2.74 (s, 2H), 2.17 – 2.06 (m, 2H), 1.17 (s, 6H) ppm.

^{13}**C{^{1}H} NMR** (101 MHz, $CDCl_3$) major isomer: δ 149.4, 144.5, 141.0, 140.7, 139.3, 128.8, 127.9, 127.6, 127.2, 127.0, 126.8, 126.2, 125.6, 62.5, 44.1, 39.7, 32.8, 29.1 (with one peak overlap in downfield); minor isomer: 149.1, 144.5, 141.0, 140.5, 139.1, 129.2, 127.8, 127.6, 127.3, 126.6, 126.3, 125.5, 62.6, 42.6, 39.2, 32.5, 29.2 ppm (with two peaks overlap in downfield).

HRMS (ESI): calculated for $C_{26}H_{29}O_2^-$ $[M+OH]^-$ 373.2173, found 373.2186.

(E)-4-(4-fluorophenyl)-6-methyl-6-phenylhept-3-en-1-ol (1ag)

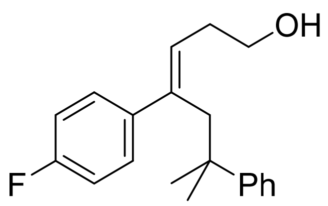

3ag
$C_{20}H_{23}FO$
M = 298.40 g/mol

Prepared from 1-(1-cyclopropylvinyl)-4-fluorobenzene **1ag** (16.2 mg, 0.1 mmol) and 4,4,5,5-tetramethyl-2-(2-phenylpropan-2-yl)-1,3,2-dioxaborolane **2a** (29.5 mg, 1.2 equiv.) according to General Procedure A at 40 ^{o}C with One-pot Oxidation. Purified by thin layer chromatography (PE/EtOAc = 10:1) to afford the product as colorless oil (16.0 mg, 53% yield, E/Z > 20:1 determined by ^{1}H NMR).

^{1}H NMR (400 MHz, $CDCl_3$) 7.31 – 7.06 (m, 7H), 6.94 – 6.83 (m, 2H), 5.48 (t, *J* = 7.2 Hz, 1H), 3.52 (t, *J* = 6.6 Hz, 2H), 2.81 (s, 2H), 2.14 (q, *J* = 6.6 Hz, 2H), 1.19 (s, 6H) ppm.

^{19}F NMR (376 MHz, $CDCl_3$) δ -116.86 ppm.

^{13}C{^{1}H} NMR (101 MHz, $CDCl_3$) δ 161.8 (d, *J* = 245.0 Hz), 149.2, 141.5 (d, *J* = 3.3 Hz), 140.2, 128.8, 128.3 (d, *J* = 7.8 Hz), 127.9, 126.1, 125.7, 114.9 (d, *J* = 21.3 Hz), 62.4, 44.4, 39.6, 32.7, 29.1 ppm.

HRMS (ESI): calculated for $C_{20}H_{23}FO^-$ $[M-H]^-$ 297.1660, found 297.1664.

(E)-6-methyl-4-(naphthalen-2-yl)-6-phenylhept-3-en-1-ol (3ah)

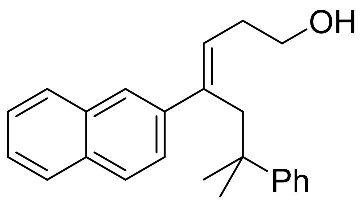

3ah
$C_{24}H_{26}O$
M = 330.47 g/mol

Prepared from 2-(1-cyclopropylvinyl)naphthalene **1ah** (19.4 mg, 0.1 mmol) and 4,4,5,5-tetramethyl-2-(2-phenylpropan-2-yl)-1,3,2-dioxaborolane **2a** (29.5 mg, 1.2 equiv.) according to General Procedure A at 40 ^{o}C with One-pot Oxidation. Purified by thin layer chromatography (PE/EtOAc = 10:1) to afford the product as colorless oil (23.4 mg, 71% yield, E/Z = 8:1 determined by ^{1}H NMR).

^{1}H NMR (400 MHz, $CDCl_3$) major isomer: δ 7.76 – 7.68 (m, 2H), 7.68 – 7.59 (m, 2H), 7.43 – 7.31 (m, 3H), 7.27 (dd, *J* = 7.2, 1.4 Hz, 2H), 7.20 – 7.12 (m, 2H), 7.07 – 6.98 (m, 1H), 5.60 (t, *J* = 7.2 Hz, 1H), 3.47 (t, *J* = 6.6 Hz, 2H), 2.89 (s, 2H), 2.16 – 2.04 (m, 2H), 1.14 (s, 6H); minor isomer: δ 7.76 – 7.68 (m, 2H), 7.68 – 7.59 (m, 2H), 7.43 – 7.31 (m, 3H), 7.27 (dd, *J* = 7.2, 1.4 Hz, 2H), 7.20 – 7.12 (m, 2H), 5.15 (t, *J* = 7.5 Hz, 1H), 3.39 (t, *J* = 6.3 Hz, 2H), 2.79 (s, 2H), 2.16 – 2.04 (m, 2H), 1.12 (s, 6H) ppm.

^{13}C{^{1}H} NMR (101 MHz, $CDCl_3$) major isomer: δ 149.5, 143.1, 141.3, 133.5, 132.4, 129.6, 128.0, 127.9, 127.7, 127.6, 126.1, 126.0, 125.7, 125.7, 125.5, 125.2, 62.4, 44.2, 39.8, 32.9, 29.1; minor isomer: 149.1, 141.0, 139.2, 133.2, 132.2, 128.8, 127.8, 127.7, 127.4, 127.4, 127.3, 126.8, 126.3, 126.0, 125.6, 62.6, 42.6, 39.3, 32.5, 29.2 ppm.

HRMS (ESI): calculated for $C_{24}H_{27}O^+$ $[M+H]^+$ 331.2056, found 331.2057.

(E)-4-(benzo[*d*][1,3]dioxol-5-yl)-6-methyl-6-phenylhept-3-en-1-ol (3ai)

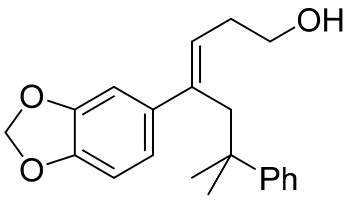

3ai
$C_{21}H_{24}O_3$
M = 324.42 g/mol

Prepared from 5-(1-cyclopropylvinyl)benzo[*d*][1,3]dioxole **1ai** (14.7 mg, 0.1 mmol) and 4,4,5,5-tetramethyl-2-(2-phenylpropan-2-yl)-1,3,2-dioxaborolane **2a** (29.5 mg, 1.2 equiv.) according to General Procedure A at 40 °C with One-pot Oxidation. Purified by thin layer chromatography (PE/EtOAc = 10:1) to afford the product as yellow oil (13.9 mg, 43% yield, E/Z > 20:1 determined by ^{1}H NMR).

^{1}H NMR (400 MHz, $CDCl_3$) δ 7.33 – 7.27 (m, 2H), 7.23 (dd, *J* = 8.6, 6.8 Hz, 2H), 7.17 – 7.07 (m, 1H), 6.75 – 6.64 (m, 3H), 5.92 (s, 2H), 5.47 (t, *J* = 7.3 Hz, 1H), 3.50 (t, *J* = 6.6 Hz, 2H), 2.77 (s, 2H), 2.11 (q, *J* = 6.6 Hz, 2H), 1.21 (s, 6H) ppm.

^{13}C{^{1}H} NMR (101 MHz, $CDCl_3$) δ 149.4, 147.4, 146.3, 140.7, 139.9, 127.9, 126.2, 125.7, 120.2, 108.0, 107.6, 101.0, 62.5, 44.5, 39.7, 32.7, 29.0 ppm (with one peak overlap in downfield).

HRMS (ESI): calculated for $C_{21}H_{25}O_4^-$ $[M+OH]^-$ 341.1758, found 341.1751.

(E)-4-(dibenzo[*b,d*]furan-2-yl)-6-methyl-6-phenylhept-3-en-1-ol (3aj)

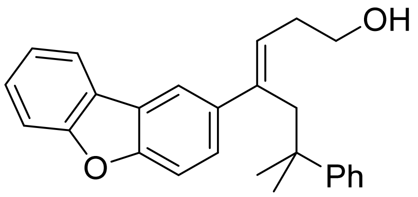

3aj
$C_{26}H_{26}O_2$
M = 370.49 g/mol

Prepared from 2-(1-cyclopropylvinyl)dibenzo[*b*,*d*]furan **1aj** (23.4 mg, 0.1 mmol) and 4,4,5,5-tetramethyl-2-(2-phenylpropan-2-yl)-1,3,2-dioxaborolane **2a** (29.5 mg, 1.2 equiv.) according to General Procedure A at 40 ^{o}C with One-pot Oxidation. Purified by thin layer chromatography (PE/EtOAc = 10:1) to afford the product as white solid (13.9 mg, 48% yield, E/Z > 20:1 determined by ^{1}H NMR, m.p. = 103.0 – 104.8 ^{o}C).

^{1}H NMR (400 MHz, $CDCl_3$) δ 7.99 – 7.92 (m, 1H), 7.76 (d, *J* = 1.8 Hz, 1H), 7.56 (dt, *J* = 8.2, 0.9 Hz, 1H), 7.50 – 7.39 (m, 2H), 7.39 – 7.28 (m, 4H), 7.25 – 7.16 (m, 2H), 7.10 – 7.02 (m, 1H), 5.61 (t, *J* = 7.2 Hz, 1H), 3.58 (t, *J* = 6.6 Hz, 2H), 2.96 (s, 2H), 2.22 (q, *J* = 6.6 Hz, 2H), 1.22 (s, 6H) ppm.

^{13}C{^{1}H} NMR (101 MHz, $CDCl_3$) δ 156.68, 155.26, 149.40, 141.11, 140.63, 128.91, 127.88, 127.16, 126.41, 126.14, 125.68, 122.74, 120.76, 118.82, 111.80, 111.05, 62.53, 44.87, 39.72, 32.88, 29.12 ppm.

HRMS (ESI): calculated for $C_{26}H_{27}O_3^-$ [M+OH]$^-$ 387.1966, found 387.1983.

(E)-7-methyl-5,7-diphenyloct-4-en-1-ol (3ak)

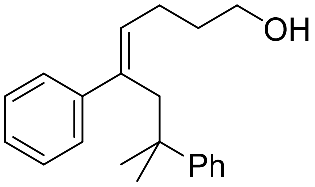

3ak
$C_{21}H_{26}O$
M = 294.44 g/mol

Prepared from (1-cyclobutylvinyl)benzene **1ak** (15.8 mg, 0.1 mmol) and 4,4,5,5-tetramethyl-2-(2-phenylpropan-2-yl)-1,3,2-dioxaborolane **2a** (29.5 mg, 1.2 equiv.) according to General Procedure A at 40 ^{o}C with One-pot Oxidation. Purified by thin layer chromatography (PE/EtOAc = 10:1) to afford the product as colorless oil (21.2 mg, 72% yield, E/Z > 20:1 determined by ^{1}H NMR).

^{1}H NMR (400 MHz, $CDCl_3$) δ 7.38 – 7.31 (m, 2H), 7.30 – 7.11 (m, 8H), 5.56 (t, *J* = 7.2 Hz, 1H), 3.60 (t, *J* = 6.5 Hz, 2H), 2.86 (s, 2H), 1.99 (q, *J* = 7.4 Hz, 2H), 1.60 – 1.47 (m, 2H), 1.21 (s, 6H) ppm.

^{13}C{^{1}H} NMR (101 MHz, $CDCl_3$) δ 149.8, 145.9, 138.9, 132.7, 128.2, 127.9, 126.9, 126.3, 126.1, 125.6, 62.7, 44.0, 39.7, 32.7, 29.0, 25.5 ppm.

HRMS (ESI): calculated for $C_{21}H_{26}ONa^+$ $[M+Na]^+$ 317.1876, found 317.1861.

3.5 Unsuccessful examples and low yield discussion

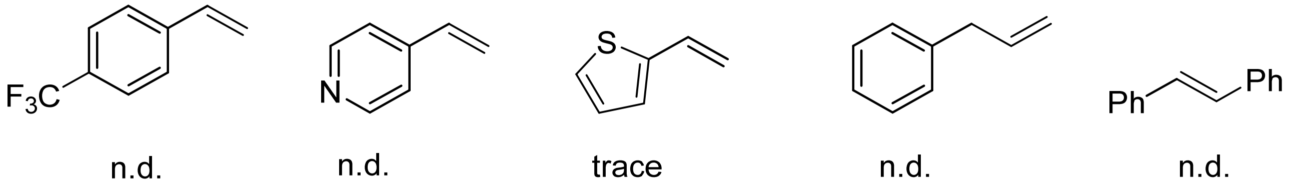

Scheme S4. Listed unsuccessful alkene substrates.

The ring-opening carboboration reaction for substrates with larger ring systems (with cyclopentyl, cyclohexyl substituent) was experimentally and computationally investigated. As shown below, our experimental studies reveal that the reaction with substrates did not afford the related carboration product. Instead, the alkene starting materials were recovered. Further DFT calculations reveal that the formation of carboboration products using cyclopentyl or cyclohexyl-derived substrates was found to be thermodynamically unfavorable.

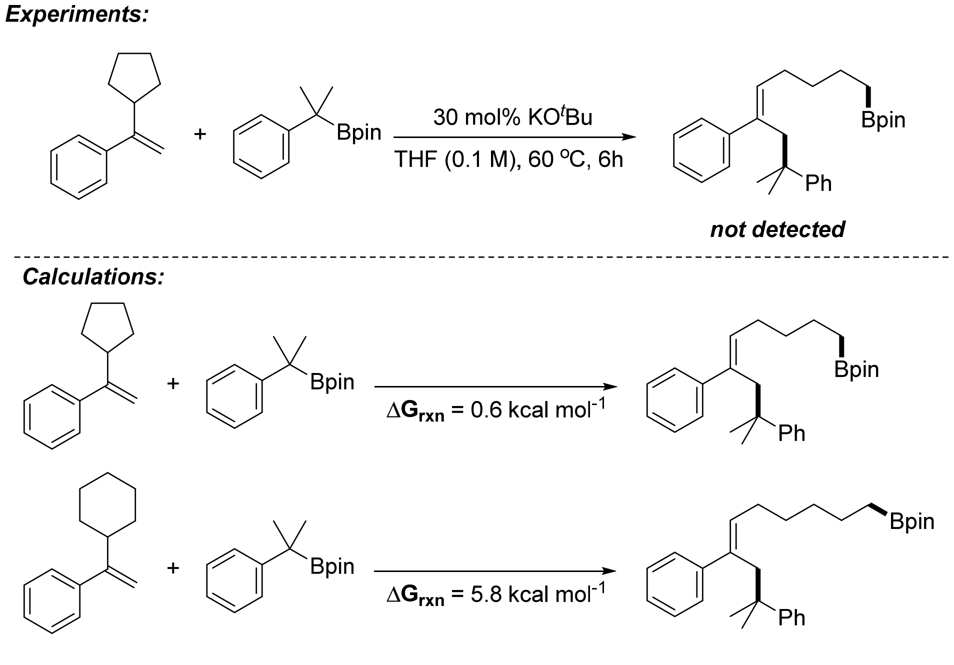

Scheme S5. Experimental and computational investigation of VCP substrates with larger ring systems.

Both aromatic ring systems in the alkene and boron reagent are found to be important, probably due to their capability to delocalize the negative charge and stabilize the corresponding carbanion intermediates, which facilitates the carboboration reaction. 1-Octene and *tert*-butylboronate were selected to be representative substrates. When subjecting these substrates to standard reaction conditions, the reaction did not produce the corresponding carboboration product. This proposal was also supported by calculation investigations, which showed that the generation of the related carbanions is kinetically unfavored. A similar phenomenon was also observed in the previous studies on the preparation of ligated benzyl potassium by Davidson et al.[10] also described a similar phenomenon.

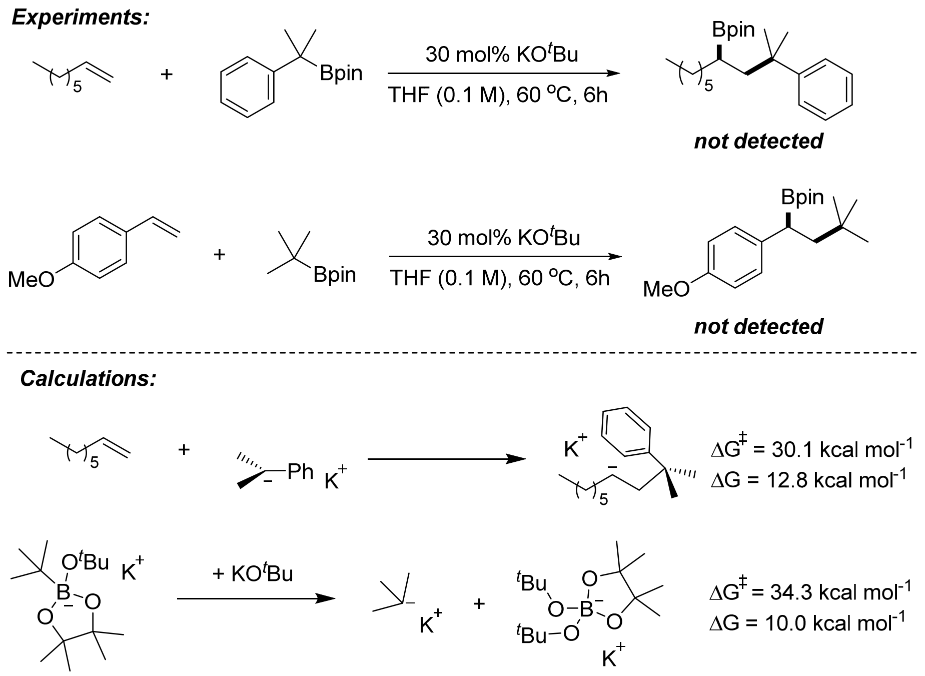

Scheme S6. Experimental and computational investigation of substrates without aromatic rings.

To investigate the possible reason for the low yield for **3s**, we have performed a one-pot carboboration/C-B oxidative hydroxylation reaction with substrate **2s**. As shown below, NMR analysis reveals the yield for carboboration product (before the addition of $NaBO_3$) and carbohydroxylation was 59% and 57%, respectively (versus 37% isolation yield for carboboration product). Therefore, the low isolated yield of **3s** may be due to the product decomposition during separation.

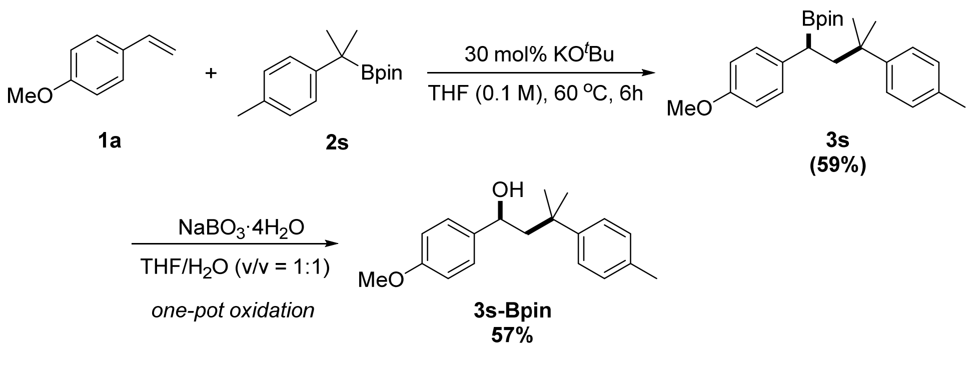

For substrates **2t** and **2u**, the low yield for addition products **3t** and **3u** was proposed to be related to the intrinsic reactivity of the corresponding carbanion intermediate **IntA** after the deborylation process, according to DFT analysis on the rate-determining step (nucleophilic addition). As shown below, compared with model substrate **2a**, the nucleophilic addition of carbanion intermediates **IntA** derived from both **2t** and **2u** toward *p*-methoxy styrene **1a** requires higher activation barriers (**IntA → IntB)**, indicating lower reactivity compared to the carbanion intermediates **IntA** derived from **2a**. These results are in qualitative consistency with the lower yields observed for substrate **2t** and **2s**.

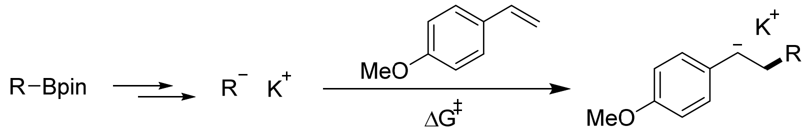

2 **IntA** **IntB**

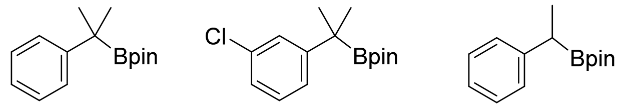

2a **2t** **2u**

Entry	R–Bpin substrate	$\Delta G^{\ddagger}$ (kcal mol^{-1})
1	**2a**	28.7
2	**2t**	30.1
3	**2u**	30.0

4. Synthetic Applications

4.1 Gram-scale experiment

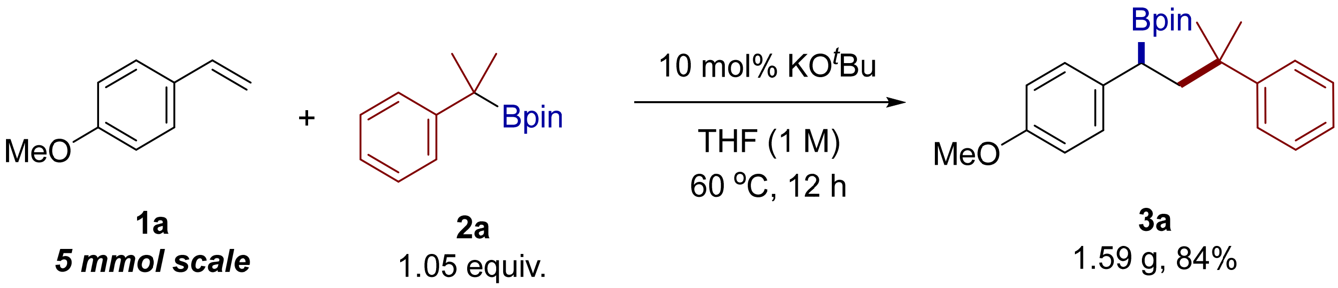

In an argon-filled glove box, an oven-dried 25 mL Schlenk-tube was sequentially charged with 4,4,5,5-tetramethyl-2-(2-phenylpropan-2-yl)-1,3,2-dioxaborolane **2a** (1.05 equiv., 1.29 g), KO^{t}Bu (56.1 mg, 0.50 mmol, 10 mol%) and anhydrous tetrahydrofuran (THF, 5 mL). Then *para*-methoxystyrene **1a** (5.0 mmol, 0.67 g) was added to the reaction mixture. The reaction tube was sealed, removed from the glovebox, and stirred at 60 °C for 12 h. After the reaction finished, saturated NH_4Cl aqueous solution (10 mL) was added to the reaction mixture, and the organic phase was separated. The aqueous layer was extracted with EtOAc (3×10 mL). Then, the organic layers were combined, dried over anhydrous Na_2SO_4, and filtered. After the removal of the solvent under reduced pressure, the crude material was purified by flash column chromatography on silica gel to afford the corresponding carboboration product **3a** as a colorless oil (1.59 g, 84%).

4.2 Product transformations

4.2.1 Bromidation/elimination reaction

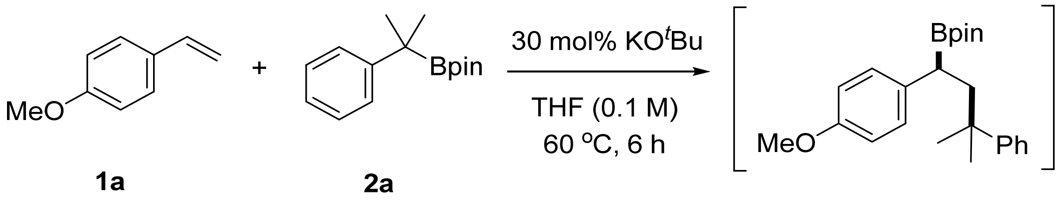

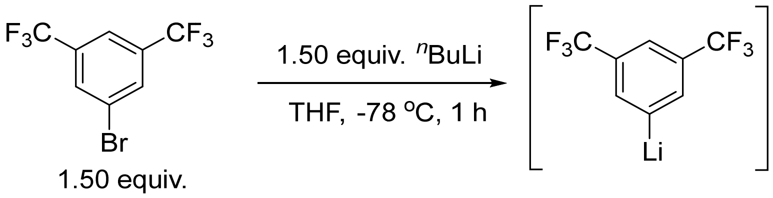

Step 1: In an argon-filled glovebox, 4,4,5,5-tetramethyl-2-(2-phenylpropan-2-yl)-1,3,2-dioxaborolane **2a** (1.2 equiv., 29.5 mg), KO^{t}Bu (30 mol%, 30 μL, 1 M in THF), and THF (1.0 mL) were added to an oven-dried reaction vial equipped with a magnetic stir bar. Then *para*-methoxystyrene **1a** (0.1 mmol, 13.4 mg)

was added to the reaction mixture. The reaction vial was capped, removed from the glovebox, and stirred at 60 °C for 6 h. The resulting reaction mixture was kept for further transformations.

Step 2: *n*-Butyllithium (2.4 M in heptane; 63 μL, 0.15 mmol, 1.50 equiv) was added dropwise to a solution of 1-bromo-3,5-bis(trifluoromethyl)benzene (43.9 mg, 0.15 mmol, 1.50 equiv) in THF (2 mL) at -78 °C. The mixture was stirred at -78 °C for 1 h.

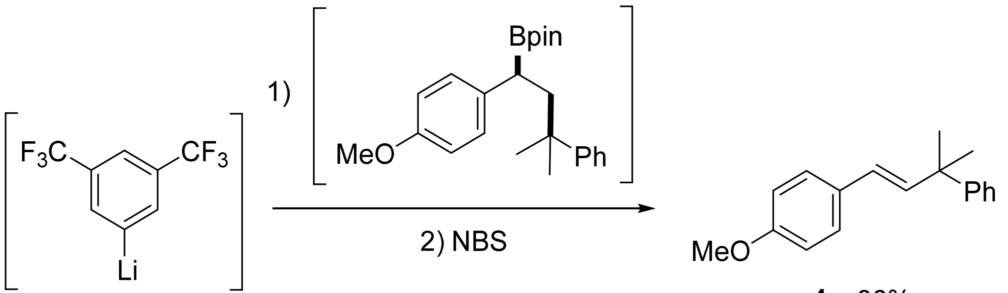

4a, 86%

Step 3: Then, the unquenched reaction mixture of **3a** was added to the aryl lithium solution at -78 °C. The mixture was stirred at -78 °C for 30 min. Next, N-bromosuccinimide (26.7 mg, 0.15 mmol, 1.50 equiv) in dry THF (1 mL) was added at -78 °C. The reaction mixture was stirred at -78 °C for 5 min, and then it was warmed to room temperature and stirred for 1 h. Na_2SO_3 (saturated aqueous solution; 10 mL) was added, and the reaction mixture was extracted with EtOAc (3 x 5 mL). The combined organic phases were dried over Na_2SO_4 and concentrated under reduced pressure. **4a** was isolated by column chromatography on silica gel (PE/EtOAc = 50:1) to afford the product as yellow oil (21.8 mg, 86% yield). The spectral data matched those reported in the literature.

(E)-1-methoxy-4-(3-methyl-3-phenylbut-1-en-1-yl)benzene (4a)[11]

^{1}H NMR (400 MHz, $CDCl_3$) δ 7.44 – 7.38 (m, 2H), 7.35 – 7.28 (m, 4H), 7.25 – 7.16 (m, 1H), 6.92 – 6.82 (m, 2H), 6.37 (d, *J* = 16.2 Hz, 1H), 6.30 (d, *J* = 16.2 Hz, 1H), 3.81 (s, 3H), 1.52 (s, 6H) ppm.

^{13}C{^{1}H} NMR (101 MHz, $CDCl_3$) δ 159.0, 149.1, 138.3, 130.7, 128.3, 127.4, 126.4, 126.0, 125.6, 114.1, 55.5, 40.8, 29.0 ppm.

HRMS (ESI): calculated for $C_{23}H_{26}NO^+$ $[M+H]^+$ 332.2009, found 332.1999.

4.2.2 Homologation reaction

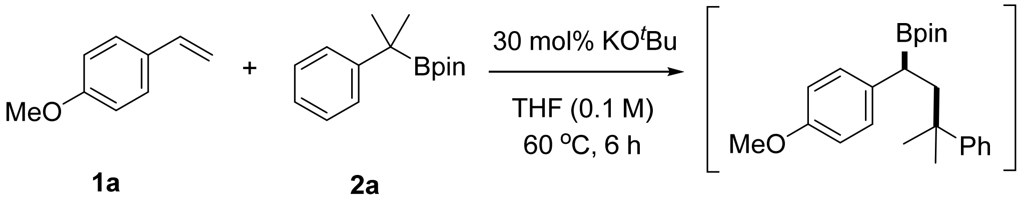

1a **2a**

Step 1: In an argon-filled glovebox, 4,4,5,5-tetramethyl-2-(2-phenylpropan-2-yl)-1,3,2-dioxaborolane **2a** (1.2 equiv., 29.5 mg), KO^{t}Bu (30 mol%, 30 µL, 1 M in THF), and THF (1.0 mL) were added to an oven-dried reaction vial equipped with a magnetic stir bar. Then *para*-methoxystyrene **1a** (0.1 mmol, 13.4 mg) was added to the reaction mixture. The reaction vial was capped, removed from the glovebox, and stirred at 60 °C for 6 h. The resulting reaction mixture was kept for further transformations.

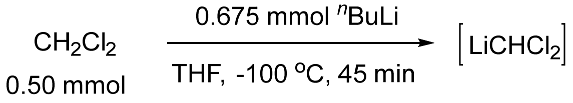

Step 2: *n*-Butyllithium (2.4 M in heptane; 0.281 mL, 0.675 mmol, 6.75 equiv) was added dropwise to a solution of CH_2Cl_2 (32.0 µL, 0.50 mmol, 5.0 equiv) in THF (5 mL) at -100 °C, and the resulting mixture was stirred for 45 min.

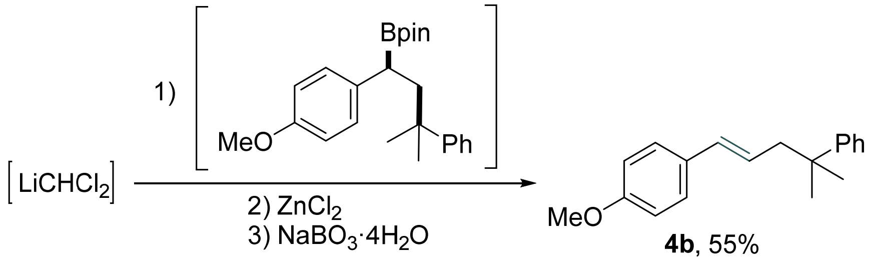

Step 3: The unquenched reaction mixture of **3a** was added to the solution of $LiCHCl_2$ at -78 °C, and the resulting mixture was stirred at -30 °C for 45 min. Next, $ZnCl_2$ (68 mg, 0.50 mmol, 5.0 equiv; the addition of $ZnCl_2$, leads to a cleaner reaction) was added, and then the mixture was stirred at room temperature for 2 h. Water (5 mL) and $NaBO_3 \cdot 4H_2O$ (77 mg, 0.50 mmol, 5.0 equiv) were added, and the suspension was stirred at room temperature for 4 h, Next, the reaction mixture was diluted with water and extracted with EtOAc (3 x 5 mL). The combined organic phases were dried over Na_2SO_4 and concentrated under reduced pressure. **4b** was isolated by column chromatography on silica gel (PE/EtOAc = 50:1) to afford the product as yellow oil (13.3 mg, 50% yield).

(E)-1-methoxy-4-(4-methyl-4-phenylpent-1-en-1-yl)benzene (4b)

^{1}H NMR (400 MHz, $CDCl_3$) δ 7.39 (dt, *J* = 8.2, 1.7 Hz, 2H), 7.36 – 7.30 (m, 2H), 7.25 – 7.16 (m, 3H), 6.85 – 6.77 (m, 2H), 6.29 (d, *J* = 15.7 Hz, 1H), 5.84 (dt, *J* = 15.7, 7.4 Hz, 1H), 3.79 (s, 3H), 2.49 (dd, *J* = 7.4, 1.2 Hz, 2H), 1.36 (s, 6H) ppm.

^{13}C{^{1}H} NMR (101 MHz, $CDCl_3$) δ 158.8, 149.5, 131.6, 128.2, 127.2, 126.0, 125.7, 125.5, 114.0, 55.4, 48.2, 28.7 ppm.

HRMS (ESI): calculated for $C_{19}H_{23}O^+$ $[M+H]^+$ 267.1743, found 267.1732.

4.2.3 Cross-coupling with terephthalonitrile

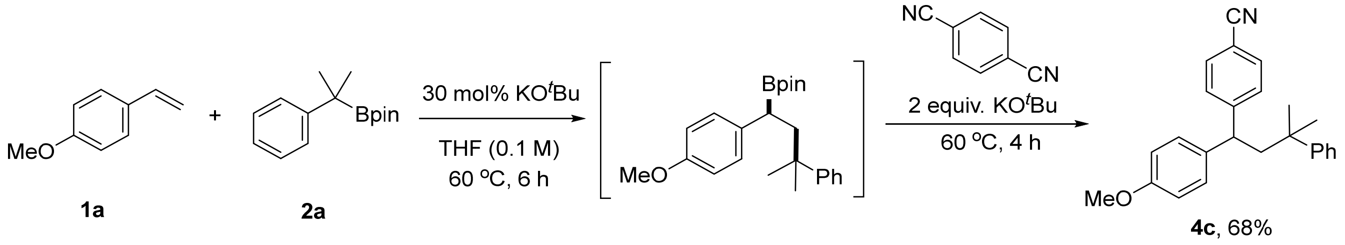

Step 1: In an argon-filled glovebox, 4,4,5,5-tetramethyl-2-(2-phenylpropan-2-yl)-1,3,2-dioxaborolane **2a** (1.2 equiv., 29.5 mg), KO^{t}Bu (30 mol%, 30 μL, 1 M in THF), and THF (1.0 mL) were added to an oven-dried reaction vial equipped with a magnetic stir bar. Then *para*-methoxystyrene **1a** (0.1 mmol, 13.4 mg) was added to the reaction mixture. The reaction vial was capped, removed from the glovebox, and stirred at 60 °C for 6 h. The resulting reaction mixture was kept for further transformations.

Step 2: In an argon-filled glovebox, KO^{t}Bu (2.0 equiv., 22.4 mg) was added to the unquenched reaction mixture of **3a**. Then terephthalonitrile (1.2 equiv., 15.4 mg) was added to the reaction mixture successively. The reaction vial was capped, removed from the glovebox, and stirred at 60 °C for 4 h. After the reaction finished, saturated NH_4Cl aqueous solution (2 mL) was added to the reaction mixture to quench the reaction, and the organic phase was separated. The aqueous layer was extracted with EtOAc (3×2 mL). Then, the organic layers were combined, dried over anhydrous Na_2SO_4, and filtered. After the removal of the solvent under reduced pressure, the crude material was purified by preparative TLC (PE/EtOAc = 10:1) to afford the corresponding product **4c** as colorless liquid (24.3 mg, 68% yield).

4-(1-(4-methoxyphenyl)-3-methyl-3-phenylbutyl)benzonitrile (4c)

^{1}H NMR (400 MHz, $CDCl_3$) δ 7.49 – 7.42 (m, 2H), 7.31 – 7.24 (m, 4H), 7.22 – 7.14 (m, 3H), 7.08 – 6.99 (m, 2H), 6.85 – 6.75 (m, 2H), 3.77 (s, 3H), 3.72 (t, *J* = 6.4 Hz, 1H), 2.49 (d, *J* = 6.4 Hz, 2H), 1.24 (s, 3H), 1.23 (s, 3H) ppm.

^{13}C{^{1}H} NMR (101 MHz, $CDCl_3$) δ 158.2, 152.3, 148.3, 136.9, 132.3, 128.7, 128.5, 128.3, 126.2, 125.9, 119.2, 114.2, 113.9, 109.5, 55.3, 50.2, 48.0, 38.8, 30.4, 29.2 ppm.

HRMS (ESI): calculated for $C_{25}H_{26}NO^+$ $[M+H]^+$ 356.2009, found 356.1996.

4.2.4 Cross-coupling with 4-methoxypyridine

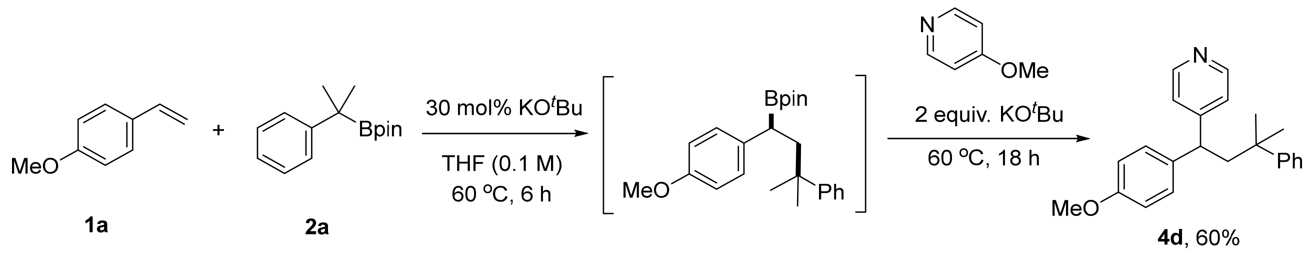

Step 1: In an argon-filled glovebox, 4,4,5,5-tetramethyl-2-(2-phenylpropan-2-yl)-1,3,2-dioxaborolane **2a** (1.2 equiv., 29.5 mg), KO^{t}Bu (30 mol%, 30 μL, 1 M in THF), and THF (1.0 mL) were added to an oven-dried reaction vial equipped with a magnetic stir bar. Then *para*-methoxystyrene **1a** (0.1 mmol, 13.4 mg) was added to the reaction mixture. The reaction vial was capped, removed from the glovebox, and stirred at 60 °C for 6 h. The resulting reaction mixture was kept for further transformations.

Step 2: In an argon-filled glovebox, KO^{t}Bu (2 equiv., 22.4 mg) was added to the unquenched reaction mixture of **3a**. Then 4-methoxypyridine (3.0 equiv., 32.7 mg) was added to the reaction mixture successively. The reaction vial was capped, removed from the glovebox, and stirred at 60 °C for 18 h. After the reaction finished, saturated NH_4Cl aqueous solution (2 mL) was added to the reaction mixture to quench the reaction, and the organic phase was separated. The aqueous layer was extracted with EtOAc (3×2 mL). Then, the organic layers were combined, dried over anhydrous Na_2SO_4, and filtered. After the removal of the solvent under reduced pressure, the crude material was purified by preparative TLC (DCM/MeOH = 30:1) to afford the corresponding product **4d** as yellow liquid (19.7 mg, 60% yield).

4-(1-(4-methoxyphenyl)-3-methyl-3-phenylbutyl)pyridine (4d)

^{1}H NMR (400 MHz, $CDCl_3$) δ 8.37 (d, *J* = 4.0 Hz, 2H), 7.34 – 7.12 (m, 4H), 7.04 – 6.95 (m, 4H), 6.81 – 6.73 (m, 3H), 3.75 (s, 3H), 3.61 (t, *J* = 6.3 Hz, 1H), 2.46 (d, *J* = 6.2 Hz, 2H), 1.22 (s, 3H), 1.21 (s, 3H) ppm.

^{13}C{^{1}H} NMR (101 MHz, $CDCl_3$) δ 158.3, 155.7, 149.8, 148.3, 136.6, 128.8, 128.3, 126.2, 125.9, 123.2, 114.1, 55.3, 49.9, 47.4, 38.9, 30.4, 29.2 ppm.

HRMS (ESI): calculated for $C_{23}H_{26}NO^+$ $[M+H]^+$ 332.2009, found 332.1999.

4.2.5 Cross-coupling with 2-chlorobenzo[*d*]oxazole

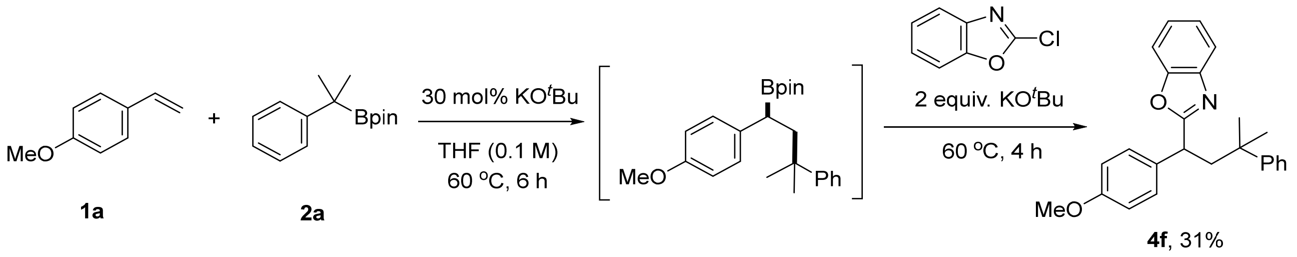

Step 1: In an argon-filled glovebox, 4,4,5,5-tetramethyl-2-(2-phenylpropan-2-yl)-1,3,2-dioxaborolane **2a** (1.2 equiv., 29.5 mg), KO^{t}Bu (30 mol%, 30 μL, 1 M in THF), and THF (1.0 mL) were added to an oven-dried reaction vial equipped with a magnetic stir bar. Then *para*-methoxystyrene **1a** (0.1 mmol, 13.4 mg) was added to the reaction mixture. The reaction vial was capped, removed from the glovebox, and stirred at 60 °C for 6 h. The resulting reaction mixture was kept for further transformations.

Step 2: In an argon-filled glovebox, KO^{t}Bu (2 equiv., 22.4 mg) was added to the unquenched reaction mixture of **3a**. Then 2-chlorobenzo[*d*]oxazole (3.0 equiv., 31.4 mg) was added to the reaction mixture successively. The reaction vial was capped, removed from the glovebox, and stirred at 60 °C for 18 h. After the reaction finished, saturated NH_4Cl aqueous solution (2 mL) was added to the reaction mixture to quench the reaction, and the organic phase was separated. The aqueous layer was extracted with EtOAc (3×2 mL). Then, the organic layers were combined, dried over anhydrous Na_2SO_4, and filtered. After the removal of the solvent under reduced pressure, the crude material was purified by preparative TLC (PE/EtOAc = 10:1) to afford the corresponding product **4e** as yellow liquid (11.4 mg, 31% yield).

2-(1-(4-methoxyphenyl)-3-methyl-3-phenylbutyl)benzo[*d*]oxazole (4e)

^{1}H NMR (400 MHz, $CDCl_3$) δ 7.65 – 7.58 (m, 1H), 7.40 – 7.33 (m, 1H), 7.33 – 7.12 (m, 8H), 7.10 – 7.01 (m, 1H), 6.82 – 6.74 (m, 2H), 4.03 (dd, *J* = 8.4, 4.7 Hz, 1H), 3.75 (s, 3H), 2.97 (dd, *J* = 14.2, 8.4 Hz, 1H), 2.37 (dd, *J* = 14.2, 4.7 Hz, 1H), 1.29 (s, 3H), 1.28 (s, 3H) ppm.

^{13}C{^{1}H} NMR (101 MHz, $CDCl_3$) δ 168.8, 158.7, 150.7, 147.8, 141.4, 133.5, 128.9, 128.2, 126.1, 125.8, 124.5, 124.1, 119.8, 114.2, 110.5, 55.4, 49.0, 42.2, 38.5, 29.3, 29.2 ppm.

HRMS (ESI): calculated for $C_{25}H_{26}NO_2^+$ [M+H]$^+$ 372.1958, found 372.1945.

4.3 Polymerization process

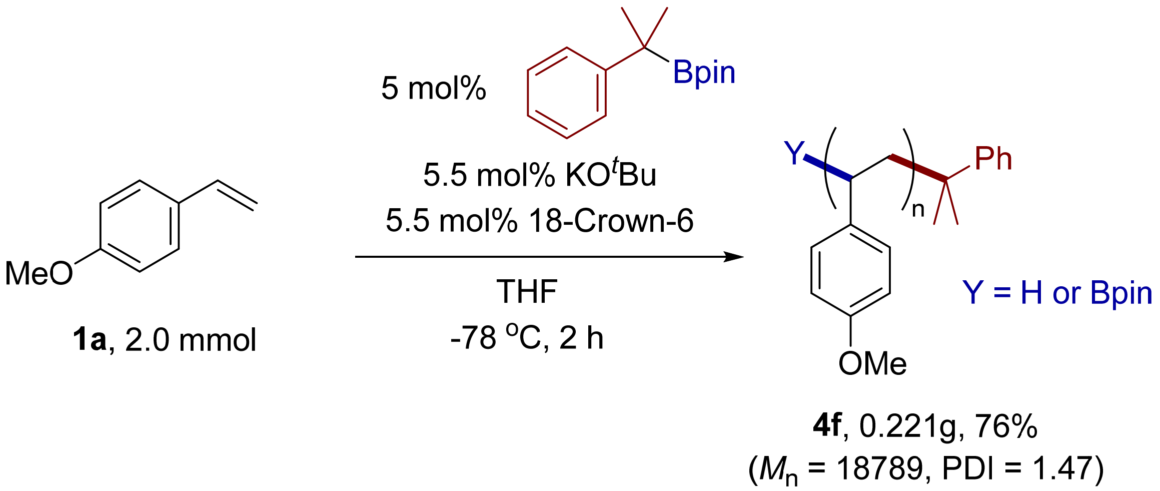

In an argon-filled glovebox, 4,4,5,5-tetramethyl-2-(2-phenylpropan-2-yl)-1,3,2-dioxaborolane **2a** (5 mol%, 0.1 mmol, 24.6 mg), KO^{t}Bu (5.5 mol%, 1 M in THF, 110 μL), 18-crown-6 (5.5 mol%, 29.0 mg) and anhydrous THF (1 mL) were added to an oven-dried reaction vial equipped with a magnetic stir bar. After the reaction mixture turned to dark reddish, the reaction vial was sealed, and removed from the glovebox. Subsequnently, a solution of *para*-methoxystyrene **1a** (2 mmol, 0.268 g) in anhydrous THF (1 mL) was added to the reaction mixture at -78 °C. The reaction was stirred at -78 °C for 2 h. After the reaction was finished, the reaction was quenched by the addition of saturated NH_4Cl aqueous solution. The organic phase was then extracted with THF (3×2 mL). The combined organic phase was dried with Na_2SO_4 and concentrated *in vaco*. The product was precipitated with excess MeOH and purified by recrystallization from MeOH. The polymer **4f** was isolated with a yield of 75%, 0.221g.

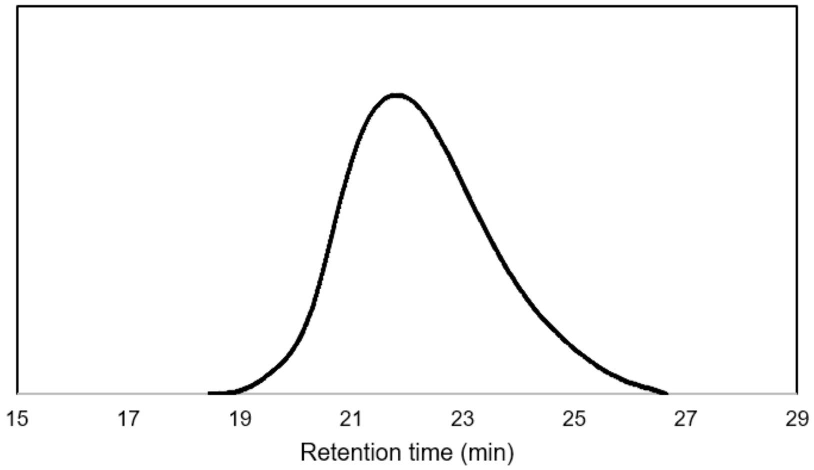

Mn	Mw	Mz	PDI
18789	27681	36404	1.47

Figure S2. GPC chromatography results of **4f**.

5. Experimental Details for Mechanistic Investigations

5.1 NMR studies of reaction intermediates

NMR analysis on the reaction mixture of 2a + KO^{t}Bu (catalytic amount). In an argon-filled glovebox, an oven-dried reaction vial equipped with a magnetic stir bar was charged with 4,4,5,5-tetramethyl-2-(2-phenylpropan-2-yl)-1,3,2-dioxaborolane **2a** (0.12 mmol, 29.5 mg), KO^{t}Bu (0.03 mmol, 3.3 mg), and deuterated tetrahydrofuran (THF-d_8, 1.0 mL). After thorough mixing, the reaction mixture was transferred to an NMR tube, sealed, and removed from the glove box. ^{1}H and ^{11}B NMR spectra of the mixture were subsequently recorded.

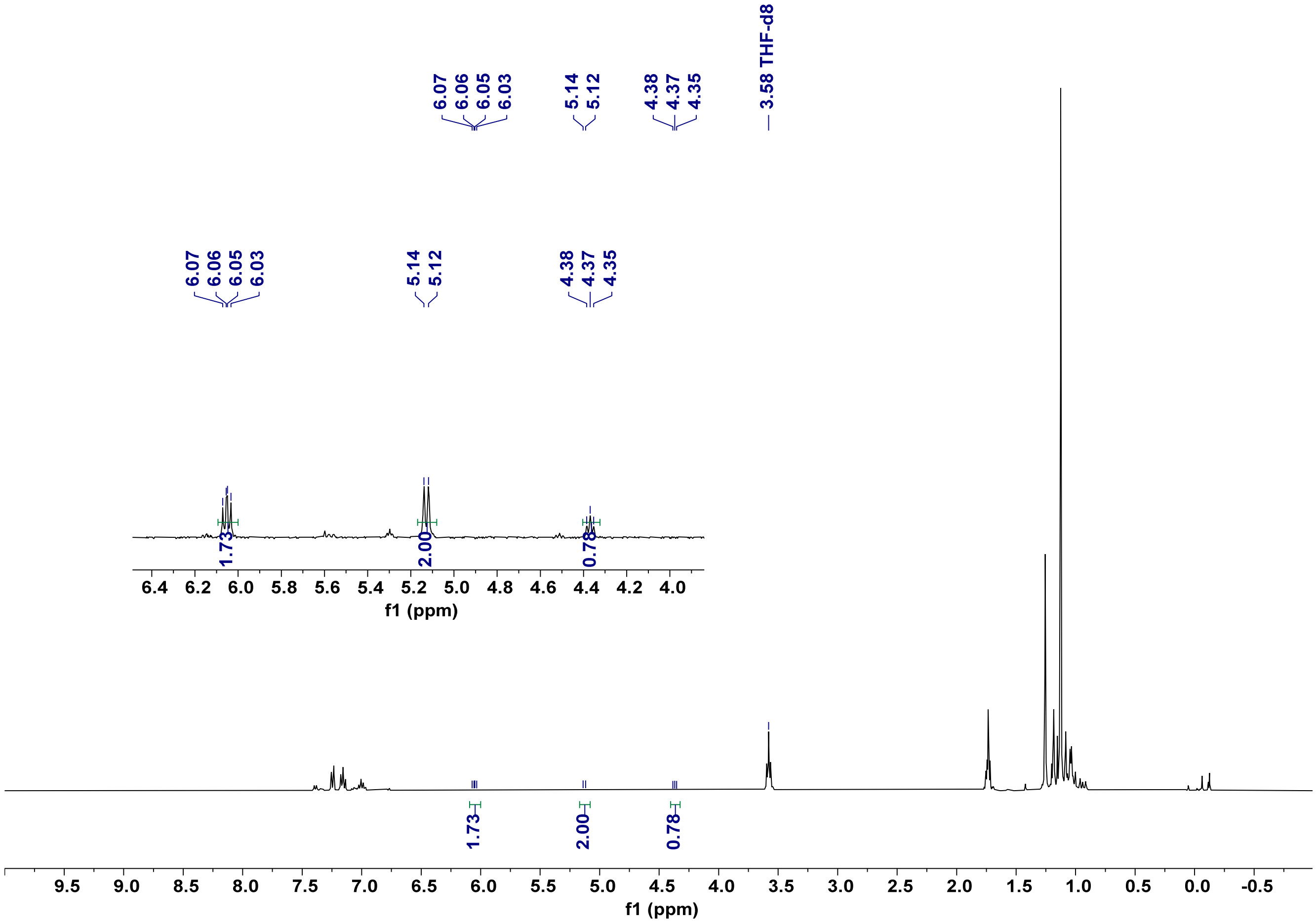

Figure S3. ^{1}H NMR spectrum (400 MHz, THF-d_8) of **2a** + cat. KO^{t}Bu.

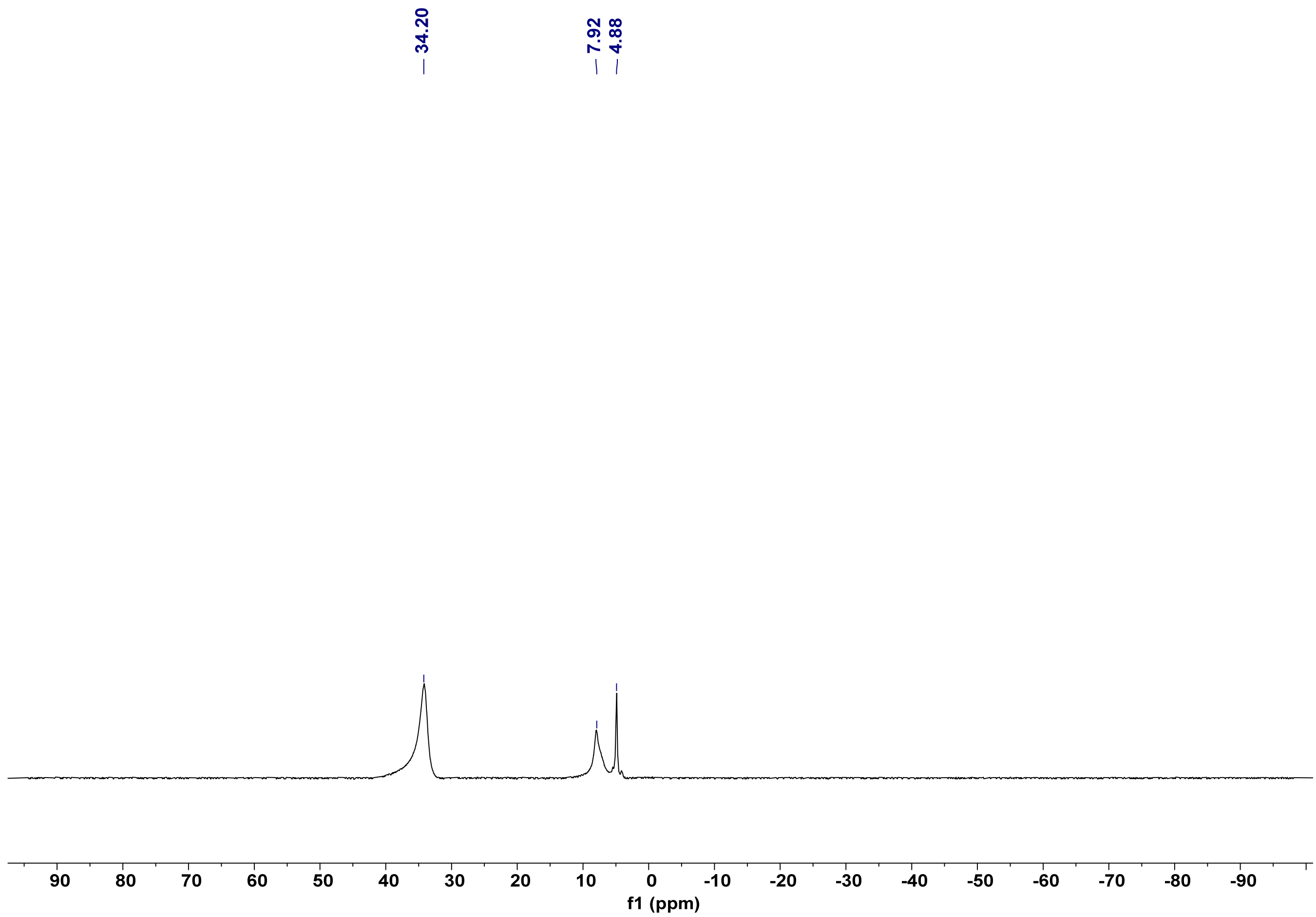

Figure S4. ^{11}B NMR spectrum (128 MHz, THF-d_8) of **2a** + cat. KO^{t}Bu.

NMR analysis on the reaction mixture at the indicated time. In an argon-filled glovebox, an oven-dried reaction vial equipped with a magnetic stir bar was charged with 4,4,5,5-tetramethyl-2-(2-phenylpropan-2-yl)-1,3,2-dioxaborolane **2a** (0.12 mmol, 29.5 mg), KO^{t}Bu (0.03 mmol, 3.3 mg), and deuterated tetrahydrofuran (THF-d_8, 1.0 mL). Then *para*-methoxystyrene **1a** (0.1 mmol, 13.4 mg) was added to the reaction mixture. The reaction vial was then sealed and stirred at 60 °C for the indicated time. Then the reaction mixture was transferred to an NMR tube, sealed, and brought out of the glove box. ^{1}H and ^{11}B NMR spectra of the mixture were subsequently recorded.

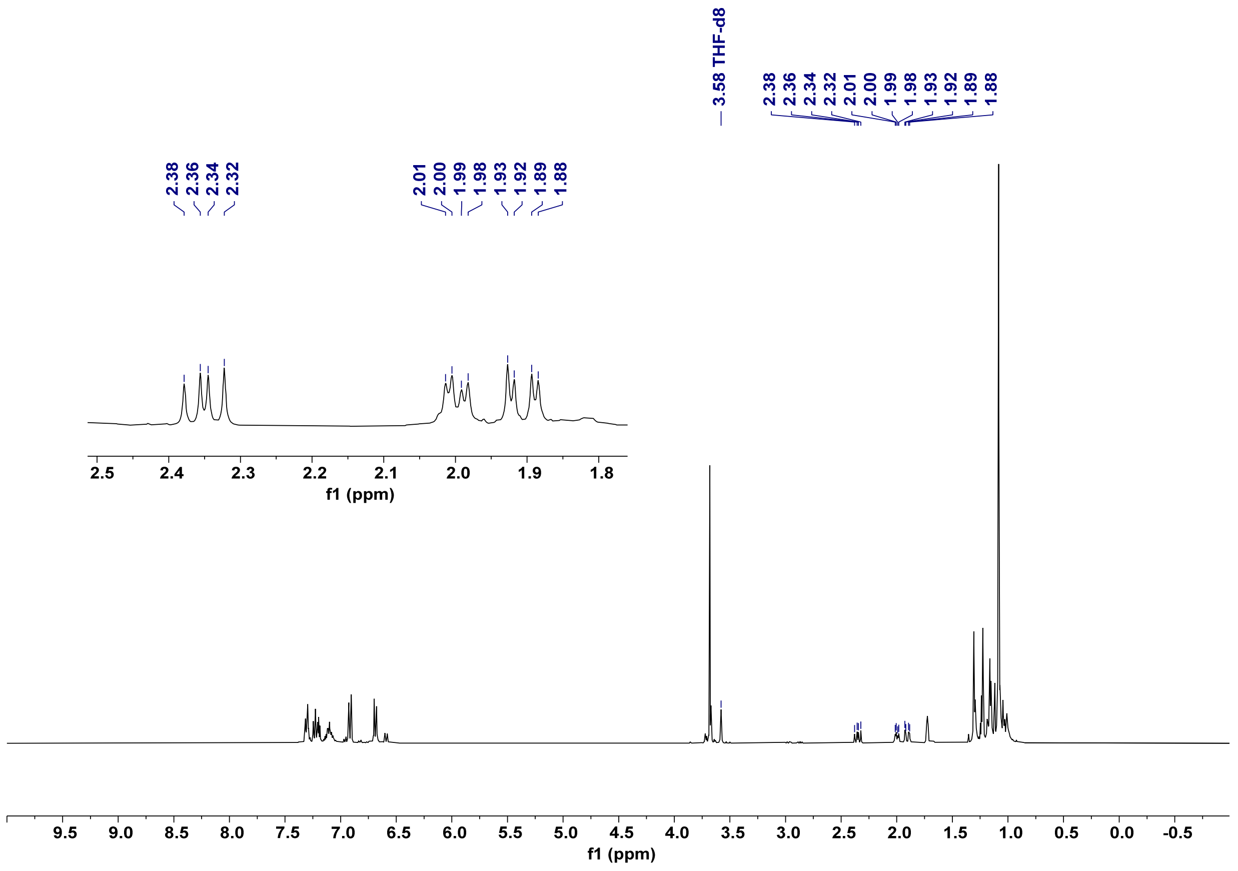

Figure S5. ^{1}H NMR spectrum (400 MHz, THF-d_8) of reaction mixture at 6 h.

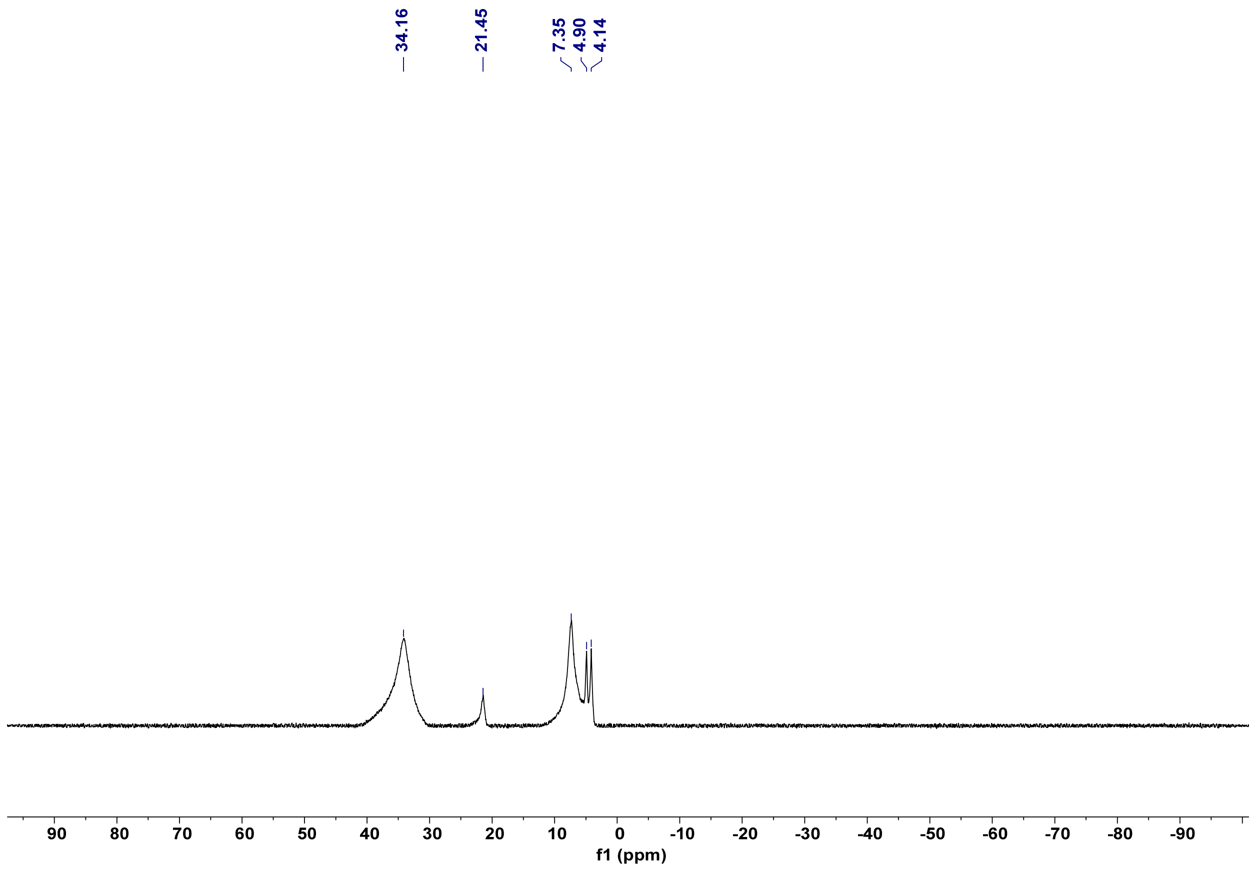

Figure S6. ^{11}B NMR spectrum (128 MHz, THF-d_8) of reaction mixture at 6 h.

NMR analysis on the reaction mixture of 3a + KO^{t}Bu (1.0 equiv.). In an argon-filled glovebox, an oven-dried reaction vial equipped with a magnetic stir bar was charged with 2-(1-(4-methoxyphenyl)-3-methyl-3-phenylbutyl)-4,4,5,5-tetramethyl-1,3,2-dioxaborolane **3a** (0.1 mmol, 38.0 mg), KO^{t}Bu (0.1 mmol, 11.2 mg), and THF-d_8 (1.0 mL). After thorough mixing, the reaction mixture was transferred to an NMR tube, sealed, and brought out of the glove box. ^{1}H and ^{11}B NMR spectra of the mixture were recorded.

In the ^{1}H NMR spectrum, no significant signals attributed to carbanion species were observed. This result can be rationalized by the inherently low thermodynamic stability of the corresponding carbanion and the rapid dynamic exchange within the borylation/deborylation equilibrium.

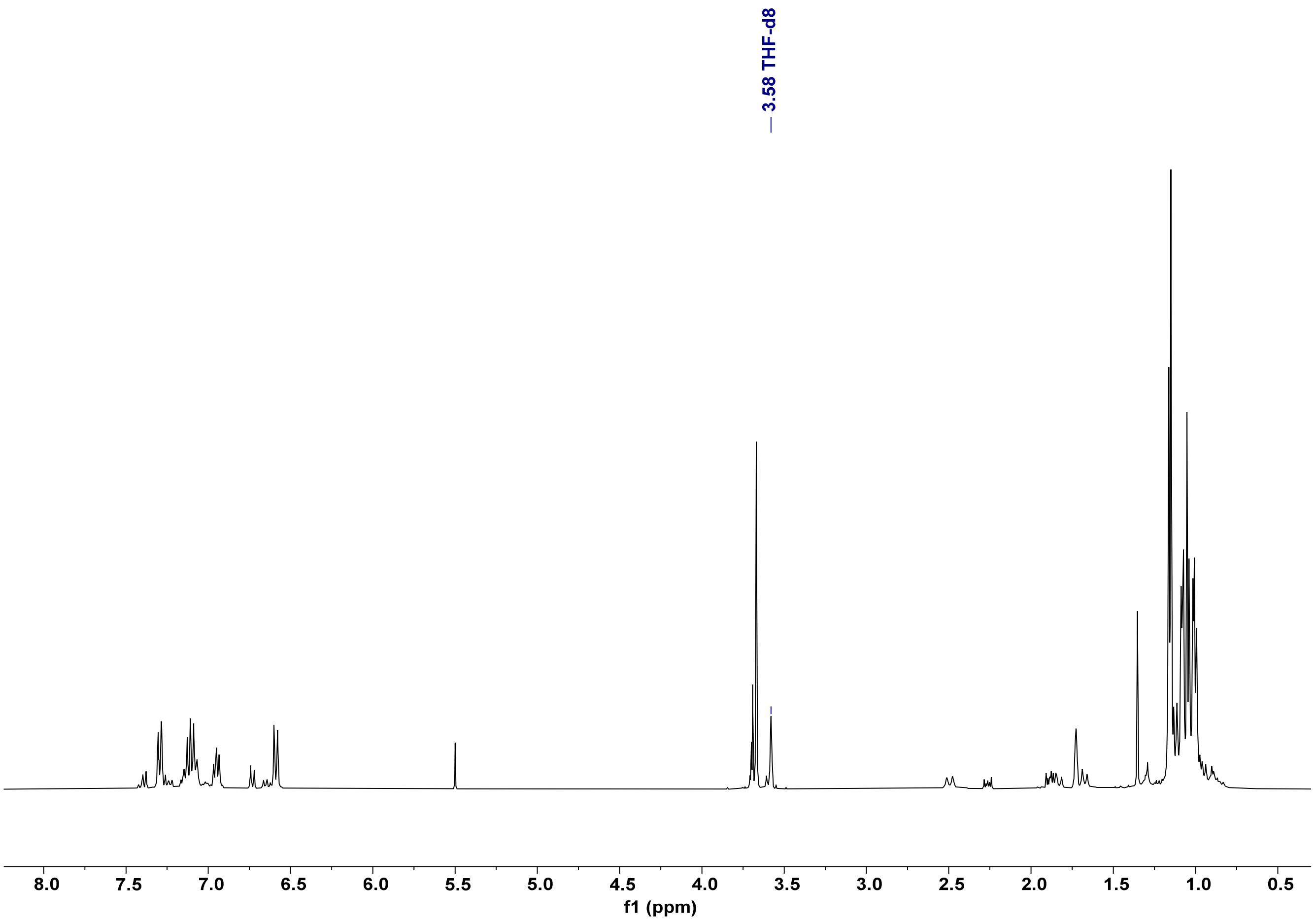

Figure S7. ^{1}H NMR spectrum (400 MHz, THF-d_8) of **3a** + 1.0 equiv. KO^{t}Bu.

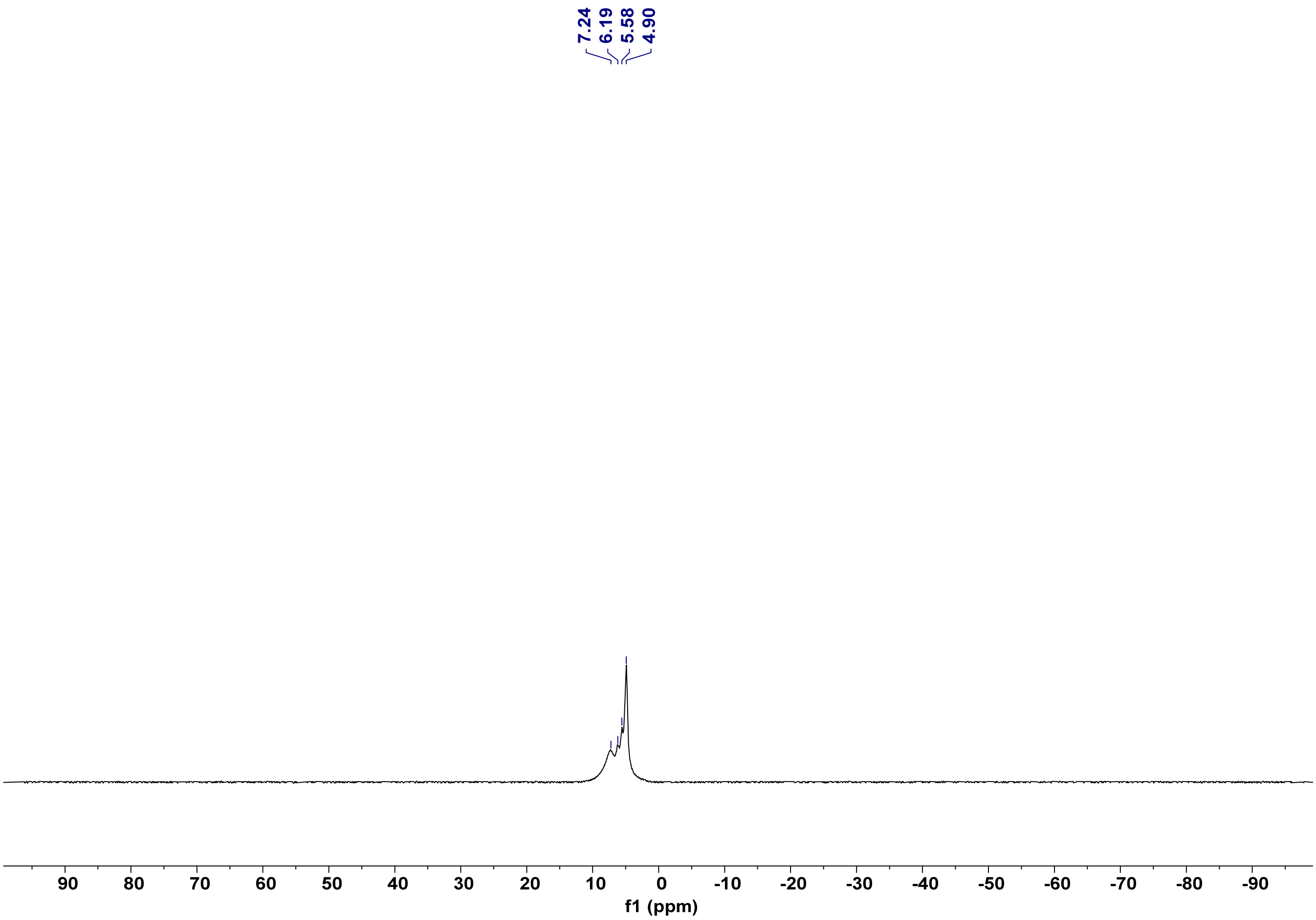

Figure S8 ^{11}B NMR spectrum (128 MHz, THF-d_8) of **3a** + 1.0 equiv. ^{t}BuOK.

To provide more substantial evidence of **Int4**, we have conducted the control experiments to probe whether the existence of **Int4** using a stoichiometric amount of KO^{t}Bu and the independently synthesized carboboration product **3a**. However, no characteristic signals (more shielded hydrogens on the aromatic ring) related to the carbanion species **Int4** could be detected according to NMR analysis. This experimental observation could be rationalized by DFT computations. As shown below, the absence of Int4 in the reaction of **3a** and KO^{t}Bu might be because the ate complex **Int6** (or **3a-KO^{t}Bu**) is predicted to be more thermodynamically favorable than the related carbanion species **Int4** or its ^{t}BuOBpin-associated form (**Int4-^{t}BuOBpin**).

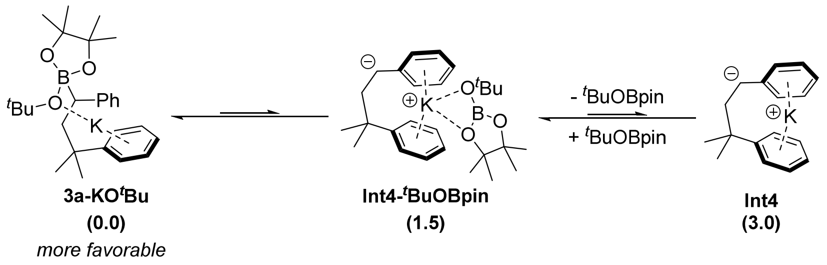

Scheme S7. Computational investigation of the equilibrium of **Int4** in the reaction mixture.

5.2 Intermediacy of cumyl potassium

Synthesis of cumyl potassium: The synthetic procedure was adapted from a previously reported method.[12] In a glovebox, a solution of *n*-butyllithium (4.2 mL, 2.4 M in hexanes, 10 mmol) was slowly added via syringe to a stirred suspension of potassium tert-butoxide (KO^{t}Bu, 1.12 g, 10 mmol) in cumene (25 mL) at 0 °C. The reaction mixture was then gradually warmed to room temperature over 30 minutes and stirred for 30 minutes at room temperature. During this time, a dark red precipitate formed, which was subsequently filtered and washed successively with dry tetrahydrofuran (THF, 20 mL) and dry hexane (20 mL). The volatiles were removed under vacuum to yield cumyl potassium ($PhMe_2CK$) as a dark red solid (0.92 g, 58% yield). **(Caution: The product can be pyrophoric if exposed to air)**

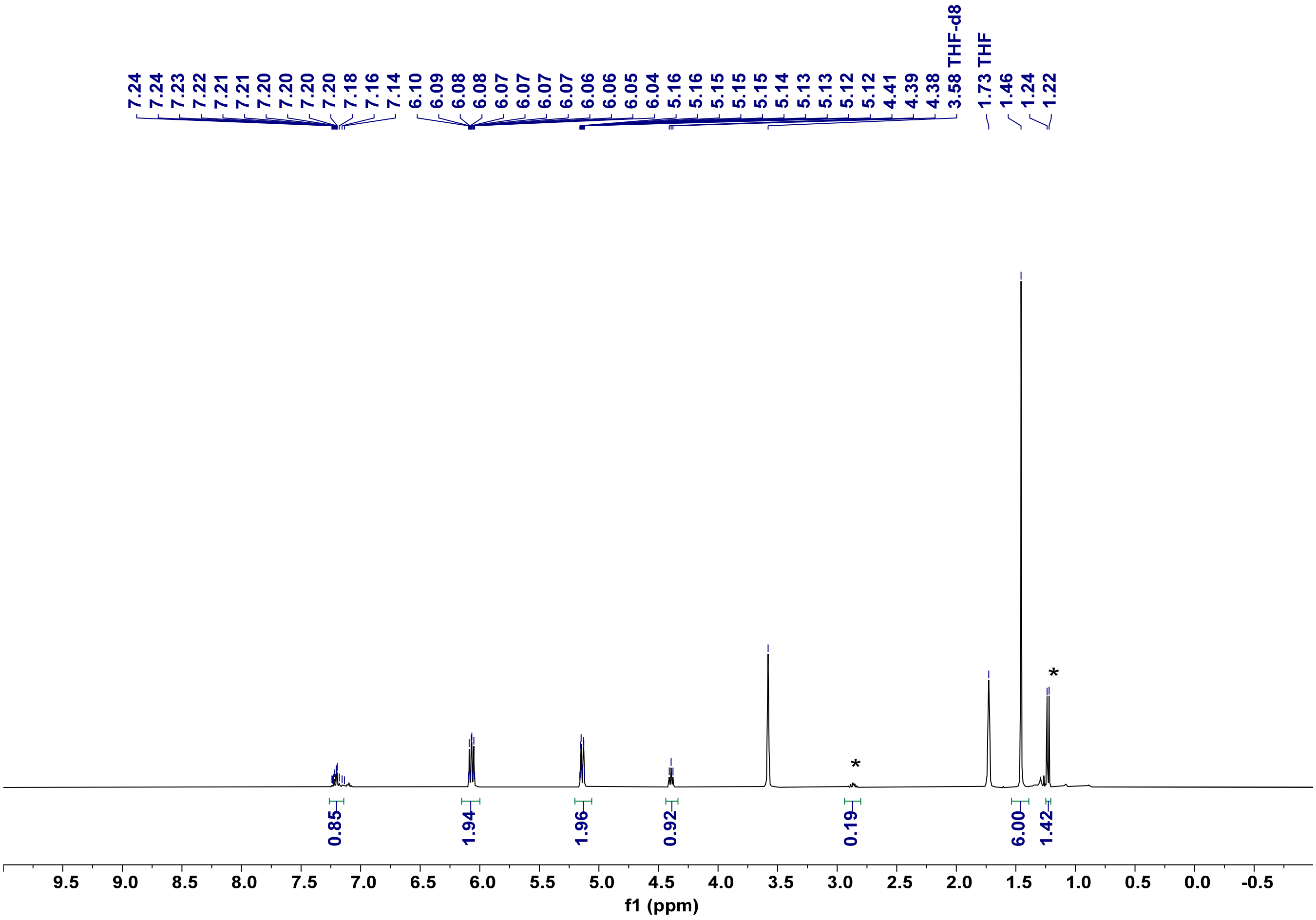

Figure S9. ^{1}H NMR spectrum (400 MHz, THF-d_8) of cumyl potassium. *Isopropylbenzene.

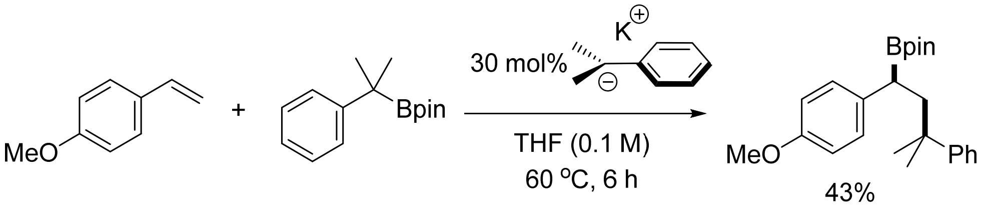

In an argon-filled glovebox, an oven-dried reaction vial equipped with a magnetic stir bar was charged with 4,4,5,5-tetramethyl-2-(2-phenylpropan-2-yl)-1,3,2-dioxaborolane **2a** (1.2 equiv., 29.5 mg), cumyl potassium ($PhMe_2CK$, 30 mol%, 4.7 mg), and anhydrous tetrahydrofuran (THF, 1 mL). *Para*-methoxystyrene **1a** (0.1 mmol, 12.2 mg) was then added to the reaction mixture. The reaction vial was sealed, removed from the glovebox, and stirred at 60 °C for 6 hours. Subsequently, the mixture was quenched with saturated ammonium chloride (NH_4Cl) aqueous solution (2 mL), and the organic phase was separated. The aqueous layer was extracted with ethyl acetate (EtOAc, 3 × 2 mL). The combined organic layers were dried over anhydrous sodium sulfate (Na_2SO_4) and filtered. After removal of the solvent under reduced pressure, the crude product was dissolved in deuterated chloroform ($CDCl_3$) with dibenzyl ether as the internal standard, and the yield of the carboboration product was determined by ^1H NMR spectroscopy.

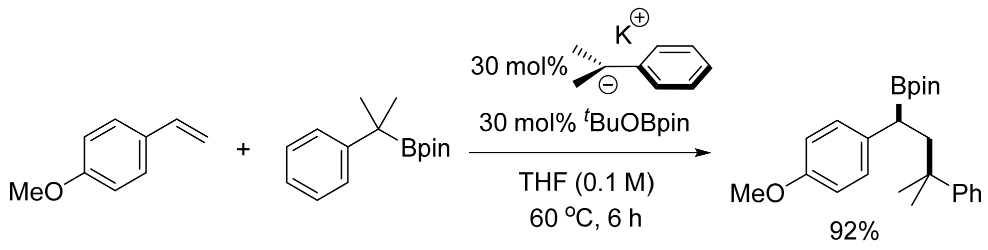

In an argon-filled glovebox, an oven-dried reaction vial equipped with a magnetic stir bar was charged with 4,4,5,5-tetramethyl-2-(2-phenylpropan-2-yl)-1,3,2-dioxaborolane **2a** (1.2 equiv., 29.5 mg), cumyl potassium ($PhMe_2CK$, 30 mol%, 4.7 mg), ^{t}BuOBpin (30 mol%, 6.0 mg), and anhydrous tetrahydrofuran (THF, 1 mL). *Para*-methoxystyrene **1a** (0.1 mmol, 12.2 mg) was then added to the reaction mixture. The reaction vial was sealed, removed from the glovebox, and stirred at 60 °C for 6 hours. Subsequently, the mixture was quenched with saturated ammonium chloride (NH_4Cl) aqueous solution (2 mL), and the organic phase was separated. The aqueous layer was extracted with ethyl acetate (EtOAc, 3 × 2 mL). The combined organic layers were dried over anhydrous sodium sulfate (Na_2SO_4) and filtered. After removal of the solvent under reduced pressure, the crude product was dissolved in deuterated chloroform ($CDCl_3$) with dibenzyl ether as the internal standard, and the yield of the carboboration product was determined by ^1H NMR spectroscopy.

5.3 Linear regression analysis on experimental yields

To gain more mechanistic information from experimental data, a tentative linear regression analysis on experimental yields was conducted, and the results are listed as follows.

Table S2. Summary of experimental data for linear regression analysis.

Substituent	σ_p	F	R	σ_I	σ_F	σ_p^+	σ_p^-	Yield A[a]	Yield B[b]
p-OCF_3	0.35	0.39	-0.04				0.27	0%	34%
p-Cl	0.23	0.42	-0.19	0.47	0.45	0.11	0.19	0%	41%
p-F	0.06	0.45	-0.39	-0.07	0.44	-0.07	-0.13	18%	73%
p-H	0	0.03	0	0	0	0	0	69%[c]	61%
p-Me	-0.17	0.01	-0.18	-0.05	0	-0.31	-0.17	69%[c]	81%
p-OPh	-0.03	0.37	-0.4	0.38		-0.5	-0.1	28%	52%
p-^{t}Bu	-0.2	-0.02	-0.18	0.5	0	-0.26	-0.03	60%[c]	68%
p-OMe	-0.27	0.29	-0.56	0.23	0.25	-0.78	-0.26	84%[d]	81%[d]
p-NMe_2	-0.83	0.15	-0.98	0.1	0.1	-1.7	-0.12	60%[d]	62%[d]
m-OMe	0.12 (σ_m)	0.29	-0.56	0.23	0.25			17%	39%[d]
o-OMe		0.29	-0.56	0.23	0.25			26%	50%[d]

[a] Isolated yield under General Procedure A (with THF as the solvent) with one-pot oxidation. [b] Isolated yield under General Procedure B (neat condition) with one-pot oxidation. [c] With 2 equiv. boronate. [d] Carboboration product isolated.

The selected substituent parameters were obtained from previous literature.[13] Selected parameters includes:σ_p and σ_m (Hammett constant), F (field inductive parameter), R (resonance parameter), σ_I (inductive parameter)[14], σ_F (field parameter)[15], σ_p^+ (nucleophilic substituent constant)[16], σ_p^- (electrophilic substituent constant)[17]. Due to the issue of data availability and better consideration of electronic effects, yields of *meta*- and *ortho*-substituted substrates are trimmed.

	σ_p	F	R	σ_I	σ_F	σ_p^+	σ_p^-	Yield A	Yield B
σ_p	1.00								
F	0.48	1.00							
R	0.82	-0.10	1.00						
σ_I	0.18	0.15	0.08	1.00					
σ_F	0.46	0.99	-0.14	0.13	1.00				
σ_p^+	0.95	0.12	0.92	0.09	0.24	1.00			
σ_p^-	0.65	0.29	0.55	0.47	0.24	0.50	1.00		
Yield A	-0.68	-0.75	-0.28	-0.30	-0.77	-0.41	-0.75	1.00	
Yield B	-0.52	-0.47	-0.30	-0.55	-0.33	-0.18	-0.90	0.79	1.00

Figure S10. Pearson correlation coefficients.

Initially, Pearson correlation analysis was conducted to examine the relationships among various parameters for preliminary screening (Figure S10). Given the observed correlations between σ_p and σ_p^+, F and σ_F, σ_p^+ and R, as well as the presence of missing values for σ_I, σ_F, and σ_p^+, the linear regression was performed using σ_p, F, R, and σ_p^- as the independent variables.

Considering the limited size of the experimental dataset, Akaike's Information Criterion (AIC) value[18] was employed to guide the stepwise regression procedure. AIC is a widely used statistical measure that effectively balances model fit with the number of parameters by penalizing over-parameterized models. The stepwise regression was implemented using the statsmodels and sklearn libraries in Python. Finally, the predictive performance and generalization ability of the final model were rigorously evaluated through leave-one-out cross-validation.[19]

5.3.1 Linear Regression Analysis of Yield A (with THF as Solvent)

```
                            OLS Regression Results
==============================================================================
Dep. Variable:                Yield A   R-squared:                       0.871
Model:                            OLS   Adj. R-squared:                  0.828
Method:                 Least Squares   F-statistic:                     20.25
Date:                Mon, 28 Apr 2025   Prob (F-statistic):            0.00215
Time:                        16:18:00   Log-Likelihood:                 7.2474
No. Observations:                   9   AIC:                            -8.495
Df Residuals:                       6   BIC:                            -7.903
Df Model:                           2
Covariance Type:            nonrobust
==============================================================================
                 coef    std err          t      P>|t|      [0.025      0.975]
------------------------------------------------------------------------------
const          0.6174      0.078      7.954      0.000       0.427       0.807
F             -0.9817      0.257     -3.820      0.009      -1.611      -0.353
σp-           -1.0732      0.286     -3.748      0.010      -1.774      -0.373
==============================================================================
Omnibus:                        2.105   Durbin-Watson:                   2.515
Prob(Omnibus):                  0.349   Jarque-Bera (JB):                0.928
Skew:                           0.773   Prob(JB):                        0.629
Kurtosis:                       2.708   Cond. No.                         7.35
==============================================================================
MSE: 0.0291          RMSE: 0.1706          MAE: 0.1343          Q²: 0.6788
==============================================================================
```

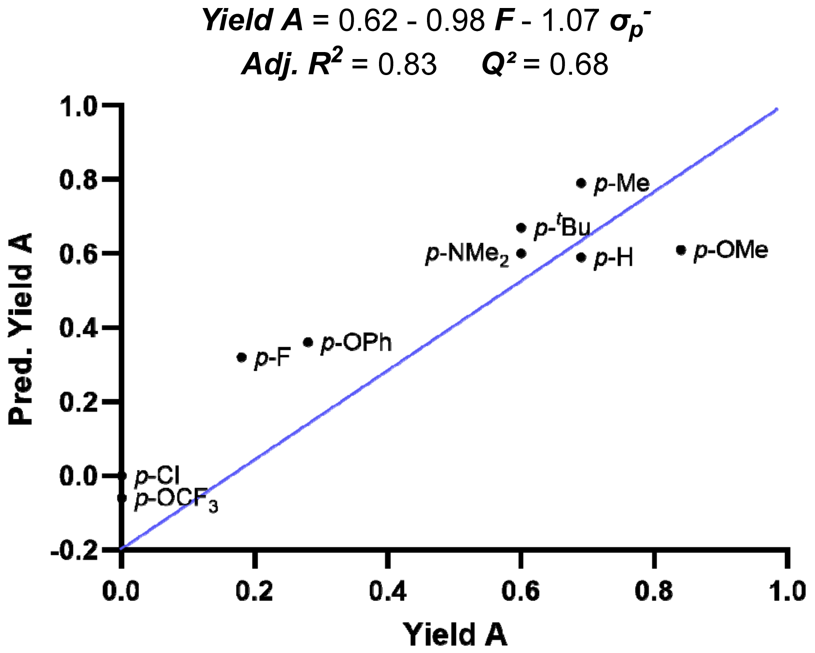

Figure S11. Predicted vs. experimental Yield A.

The reaction yields obtained under THF-solvated conditions exhibited a good correlation with substituent constant F and the Hammett parameter σ_p^-, as evidenced by an adjusted coefficient of determination (Adj. $R^2 = 0.83$). Both independent variables demonstrated high statistical significance ($p < 0.01$ for each parameter). However, leave-one-out cross-validation analysis yielded a substantially lower predictive

squared correlation coefficient (Q^2 = 0.68). This discrepancy (ΔR^2-Q^2 = 0.15) between the goodness-of-fit and predictive power suggests potential model overfitting, which consequently limits the model's generalization capability and predictive accuracy for new observations.

5.3.2 Linear regression analysis of Yield B (Solvent-Free Condition)

```
                            OLS Regression Results
==============================================================================
Dep. Variable:                Yield B   R-squared:                       0.876
Model:                            OLS   Adj. R-squared:                  0.835
Method:                 Least Squares   F-statistic:                     21.18
Date:                Mon, 28 Apr 2025   Prob (F-statistic):            0.00191
Time:                        16:25:41   Log-Likelihood:                 13.317
No. Observations:                   9   AIC:                            -20.63
Df Residuals:                       6   BIC:                            -20.04
Df Model:                           2
Covariance Type:            nonrobust
==============================================================================
                 coef    std err          t      P>|t|      [0.025      0.975]
------------------------------------------------------------------------------
const          0.6257      0.035     17.637      0.000       0.539       0.712
σp-           -1.0320      0.167     -6.175      0.001      -1.441      -0.623
R              0.1583      0.094      1.686      0.143      -0.071       0.388
==============================================================================
Omnibus:                       16.673   Durbin-Watson:                   2.514
Prob(Omnibus):                  0.000   Jarque-Bera (JB):                7.772
Skew:                          -1.889   Prob(JB):                       0.0205
Kurtosis:                       5.539   Cond. No.                         8.33
==============================================================================
MSE: 0.0056           RMSE: 0.0750            MAE: 0.0538           Q²: 0.7701
==============================================================================
```

$$\textbf{\textit{Yield B}} = 0.63 - 1.03\ \sigma_p^- - 0.16\ R$$

$$\textit{Adj. } R^2 = 0.84 \qquad Q^2 = 0.77$$

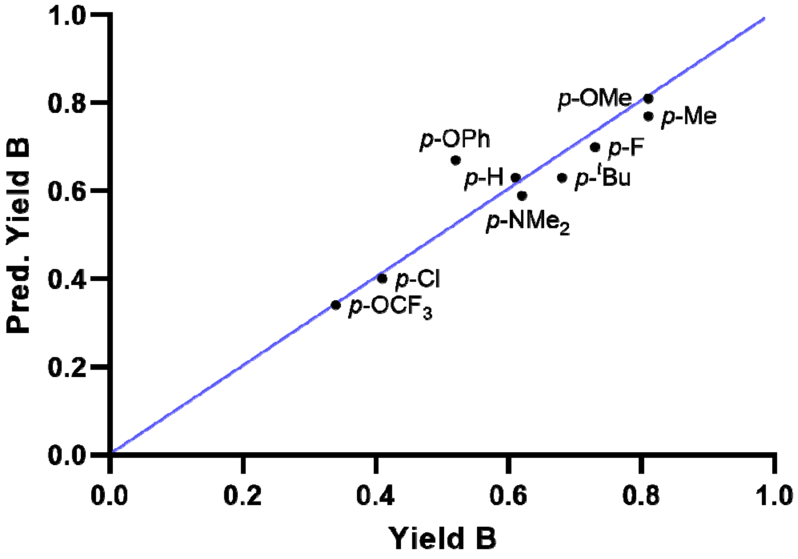

Figure S12. Predicted vs. experimental Yield B.

As shown in Figure S12, under neat (solvent-free) conditions, the reaction yields demonstrated a strong linear correlation with the Hammett parameter σ_p^- and the substituent constant R, as indicated by the coefficient of determination (Adj. R^2 = 0.84). However, statistical analysis revealed that the contribution of R was not significant (p = 0.143), suggesting its limited predictive value in the current model. In light of these findings, we subsequently performed a univariate linear regression analysis to evaluate the sole influence of σ_p^- on Yield B.

5.3.3 Single-variate linear regression analysis of Yield B

```
                            OLS Regression Results
==============================================================================
Dep. Variable:                Yield B   R-squared:                       0.817
Model:                            OLS   Adj. R-squared:                  0.791
Method:                 Least Squares   F-statistic:                     31.28
Date:                Mon, 28 Apr 2025   Prob (F-statistic):           0.000822
Time:                        20:14:15   Log-Likelihood:                 11.572
No. Observations:                   9   AIC:                            -19.14
Df Residuals:                       7   BIC:                            -18.75
Df Model:                           1
Covariance Type:            nonrobust
==============================================================================
                 coef    std err          t      P>|t|      [0.025      0.975]
------------------------------------------------------------------------------
const          0.5803      0.026     22.315      0.000       0.519       0.642
σp-           -0.8768      0.157     -5.593      0.001      -1.247      -0.506
==============================================================================
Omnibus:                        3.801   Durbin-Watson:                   1.512
Prob(Omnibus):                  0.150   Jarque-Bera (JB):                1.341
Skew:                          -0.941   Prob(JB):                        0.511
Kurtosis:                       3.176   Cond. No.                         6.21
==============================================================================
MSE: 0.0060          RMSE: 0.0775           MAE: 0.0576          Q²: 0.7543
==============================================================================
```

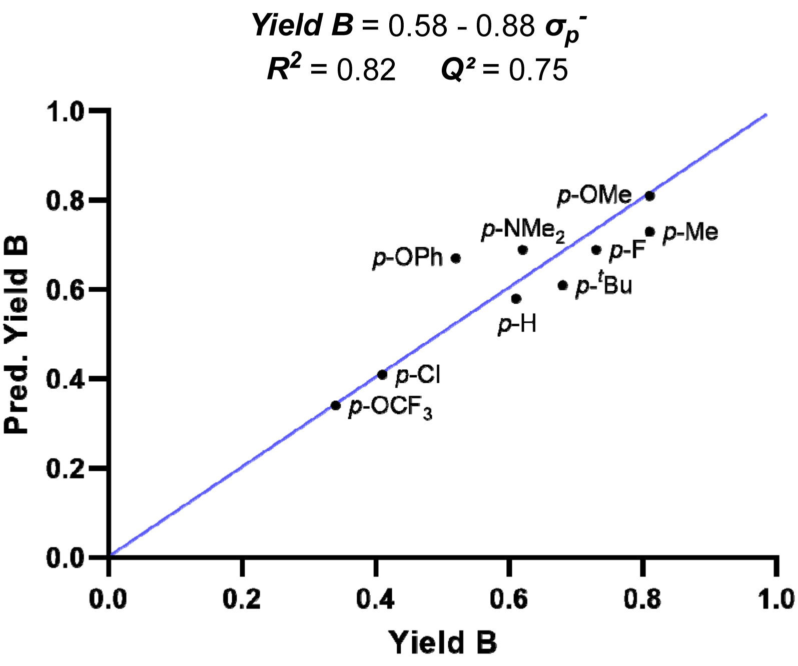

Figure S13. Predicted vs. experimental Yield B.

As shown in Figure S13, the univariate regression model maintains a statistically robust linear correlation between Yield B and the Hammett parameter σ_p^-. This relationship is further supported by leave-one-out cross-validation results (Q^2 = 0.75), demonstrating satisfactory predictive performance given the constrained sample size of the dataset.

5.4 Influence of THF addition on reaction yield

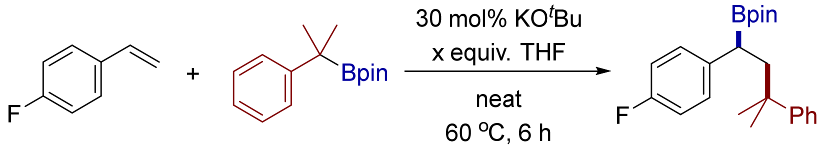

1i **2a**, 3.0 equiv. **3i-Bpin**

THF equiv.	NMR yield
-	80%
1.0	75%
2.0	71%
3.0	66%
5.0	57%

In an argon-filled glovebox, 4,4,5,5-tetramethyl-2-(2-phenylpropan-2-yl)-1,3,2-dioxaborolane **2a** (1.2 equiv., 29.5 mg), KO*t*Bu (30 mol%, 3.3 mg), and indicated equivalents of THF were added to an oven-dried reaction vial equipped with a magnetic stir bar. Subsequently, *para*-fluorostyrene **1j** (0.1 mmol, 12.2 mg) was added to the reaction mixture. The reaction vial was sealed, removed from the glovebox, and stirred at 60 °C for 6 h. Then, saturated NH_4Cl aqueous solution (2 mL) was added to the reaction mixture, and the organic phase was separated. The aqueous layer was extracted with EtOAc (3×2 mL). Then, the organic layers were combined, dried over anhydrous Na_2SO_4, and filtered. After the removal

of the solvent under reduced pressure, the crude mixture was added perfluorobenzene as the internal standard, and the yield of carboboration product was determined by ^{19}F NMR spectroscopy.

5.5 Influence of crown ether addition on reaction yield

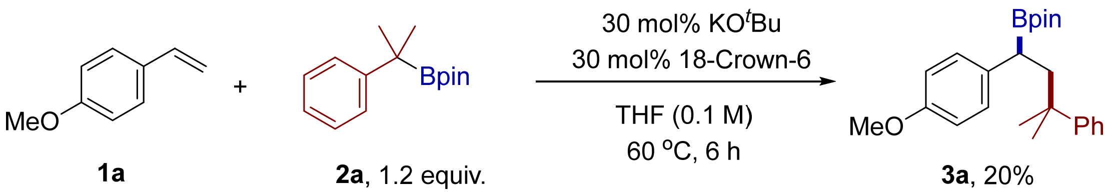

In an argon-filled glovebox, 4,4,5,5-tetramethyl-2-(2-phenylpropan-2-yl)-1,3,2-dioxaborolane **2a** (1.2 equiv., 29.5 mg), KO^{t}Bu (30 mol%, 3.3 mg), 18-crown-6 (30 mol%, 7.9 mg) and anhydrous THF (1 mL) were added to an oven-dried reaction vial equipped with a magnetic stir bar. Then *para*-methoxystyrene **1a** (0.1 mmol, 13.4 mg) was added to the reaction mixture. The reaction vial was sealed, removed from the glovebox, and stirred at 60 °C for 6 h. After the reaction was finished, saturated NH_4Cl aqueous solution (2 mL) was added to the reaction mixture to quench the reaction, and the organic phase was separated. The aqueous layer was extracted with EtOAc (3×2 mL). Then, the organic layers were combined, dried over anhydrous Na_2SO_4, and filtered. After the removal of the solvent under reduced pressure, the crude mixture was added dibenzyl ether as the internal standard, and the yield of carboboration product was determined by ^{1}H NMR spectroscopy.

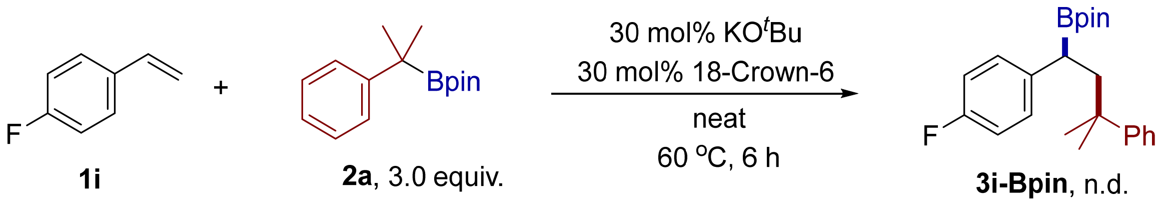

In an argon-filled glovebox, 4,4,5,5-tetramethyl-2-(2-phenylpropan-2-yl)-1,3,2-dioxaborolane **2a** (3.0 equiv., 73.8 mg), KO^{t}Bu (30 mol%, 3.3 mg), and 18-crown-6 (30 mol%, 7.9 mg) were added to an oven-dried reaction vial equipped with a magnetic stir bar. Then *para*-fluoroxystyrene **1i** (0.1 mmol, 12.2 mg) was added to the reaction mixture. The reaction vial was sealed, removed from the glovebox, and stirred at 60 °C for 6 h. After the reaction was finished, saturated NH_4Cl aqueous solution (2 mL) was added to the reaction mixture to quench the reaction, and the organic phase was separated. The aqueous layer was extracted with EtOAc (3×2 mL). Then, the organic layers were combined, dried over anhydrous Na_2SO_4, and filtered. After the removal of the solvent under reduced pressure, the crude mixture was added perfluorobenzene as the internal standard, and the yield of carboboration product was determined by ^{1}H NMR spectroscopy.

5.6 Electron paramagnetic resonance (EPR) experiments

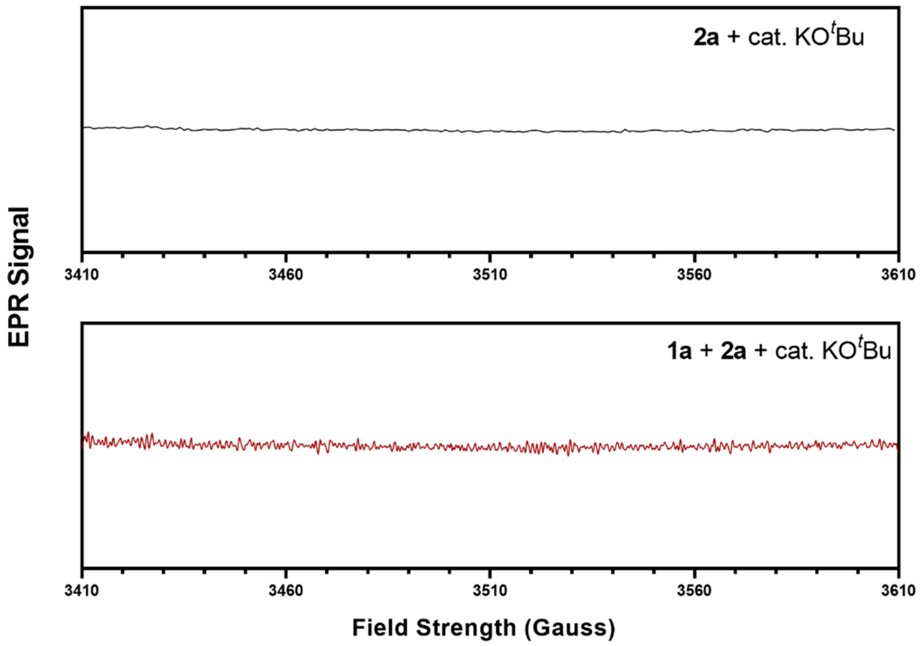

Figure S14. Electron paramagnetic resonance study of (a) catalytic amount (30 mol%) of KO^{t}Bu and **2a** and (b) reaction mixture under General Procedure B in THF at 100 K.

Electron paramagnetic resonance (EPR) spectroscopy was employed to probe for possible radical intermediates under the reaction conditions. As shown in Figure S14, no radical signals were detected in either of the following control experiments: (i) the reaction of substrate **2a** with base alone, or (ii) the combined reaction system containing **2a**, base, and alkene **1a**.

6. Computational Investigations

6.1 Computational details

All calculations were performed with the Gaussian 16 package[20]. The 3D structures of the optimized species were generated using CYLview[21]. Geometry optimizations were performed at M06-2X[22]/def2-SVP[23] level of theory in conjunction with the SMD solvation model[24] for tetrahydrofuran. Vibrational frequencies were calculated for all the geometries to provide thermal corrections to the Gibbs free energies at 298.15 K and 1.0 atm, and to confirm whether the optimized geometry corresponds to a minimum or a transition state (TS). Intrinsic reaction coordinate (IRC) calculations were performed to verify whether a TS connects the correct minimum structures. Single-point energy calculations were done with the same functional and solvation model using the def2-TZVPP[23] basis set for more accurate energies. Free energy barriers here are defined as the free energy difference between the transition state and the lowest-energy stationary point before it along the reaction pathways. NMR shifts were computed according to a previous report[25] using the Gauge-independent atomic orbital (GIAO) method at B97-2[26]/pcSseg-2[25] level of theory. Free energy barriers of single electron transfer (SET) processes were also calculated with the above computational level used for optimization and single-point energy calculations, utilizing the Marcus theory[27] for electron donor-acceptor pairs.

Due to the complexity of the reaction network, the potential reaction pathways (borylation and oligomerization) were systematically explored by the molecular dynamics and coordinate driving (MD/CD) methods[28] developed by our group. The potassium ion was initially excluded from the reaction model, and the active atoms are shown in Figure S15A. The *MaxOrder* of the CD procedure was set to 2, the *CrossedRC* protocol was employed, and the active B atom (**M3**) was set as *CrossAtoms*. The MD simulation was performed at the GFN-FF level with default parameters. The solvent effect was treated with the ALPB Model with THF in CD procedure. The *Autothr* protocol was employed to automatically determine the energy and RMSD thresholds. More detailed settings for MD/CD are shown in Table S3. The obtained transition states (Figure S15B) were further added potassium cation and refined at DFT level.

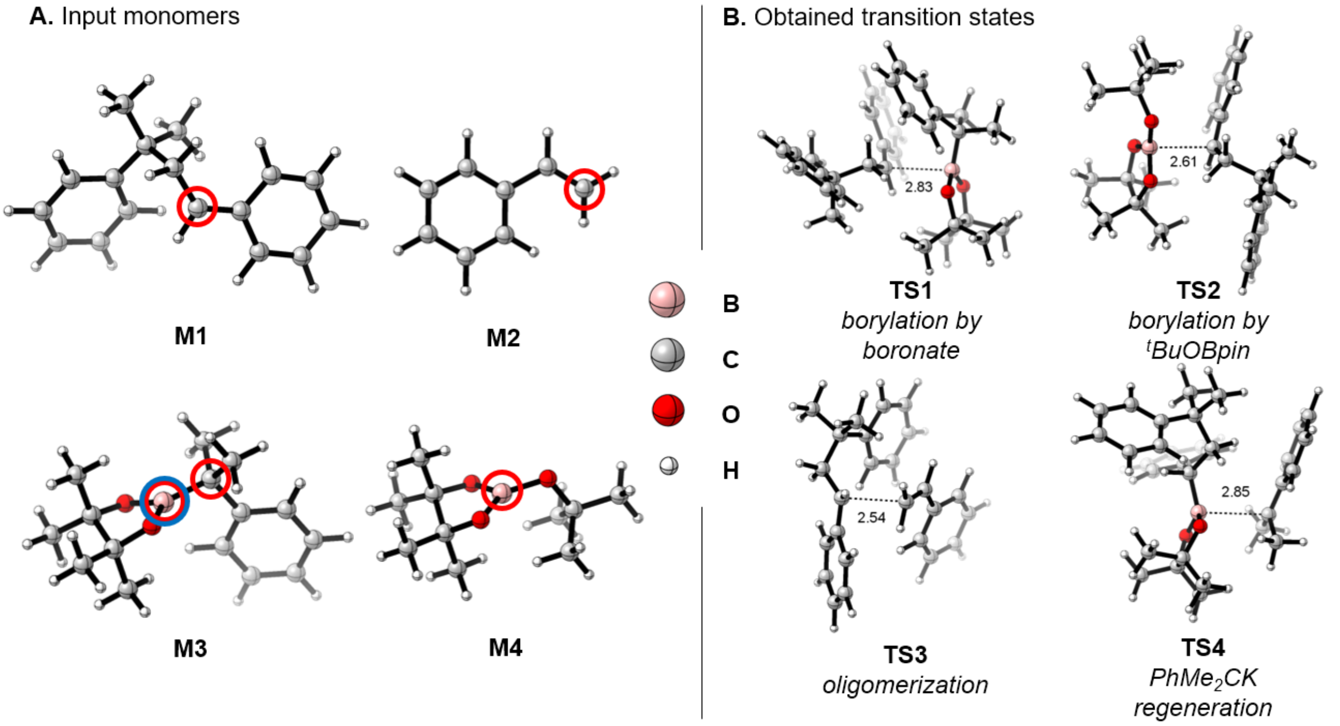

Figure S15. Illustration of input monomers and obtained transition states of MD/CD. Atoms within red circles are set as active atoms, and atoms within blue circles are set as *CrossedAtoms*. Selected distances were labeled in Å. Color code: white: hydrogen; grey: carbon; pink: boron; red: oxygen.

Table S3. Detailed input file (.yaml) of ADCR program for the carboboration reaction exploration without potassium cation.

**Keywords**			**Value**
Global	Stage1	Ecut	40.0
		GauXTBSolvent	THF
		DeltaGcut	20.0
		DeltaGmin	5.0
System	Autothr	-	True
	SmartMatch	-	False
	BaseDistance	-	2.5
	ScaleFactor	-	0.25
MD	Type	-	Metad
	Level	-	GFN-FF
	NCfms	-	15
	Sccacc	-	1.0
CD	Mode	-	Split
	MaxOrder	-	2
	MinOrder	-	1
	Break	-	0.90
	From	-	3.50
Monomer	M1	Charge	-1
		ActiveAtoms	*(See Figure S15 M1, the C atom)*
	M2	ActiveAtoms	*(See Figure S15 M2, the C atom)*

	M3	ActiveAtoms	*(See Figure S15 M3, the C, B atoms)*
		CrossedAtoms	*(See Figure S15 M3, the B atom)*
	M4	ActiveAtoms	*(See Figure S15 M4, the B atom)*

6.2 KO^{t}Bu-catalyzed carboboration of alkene with implicit solvation model

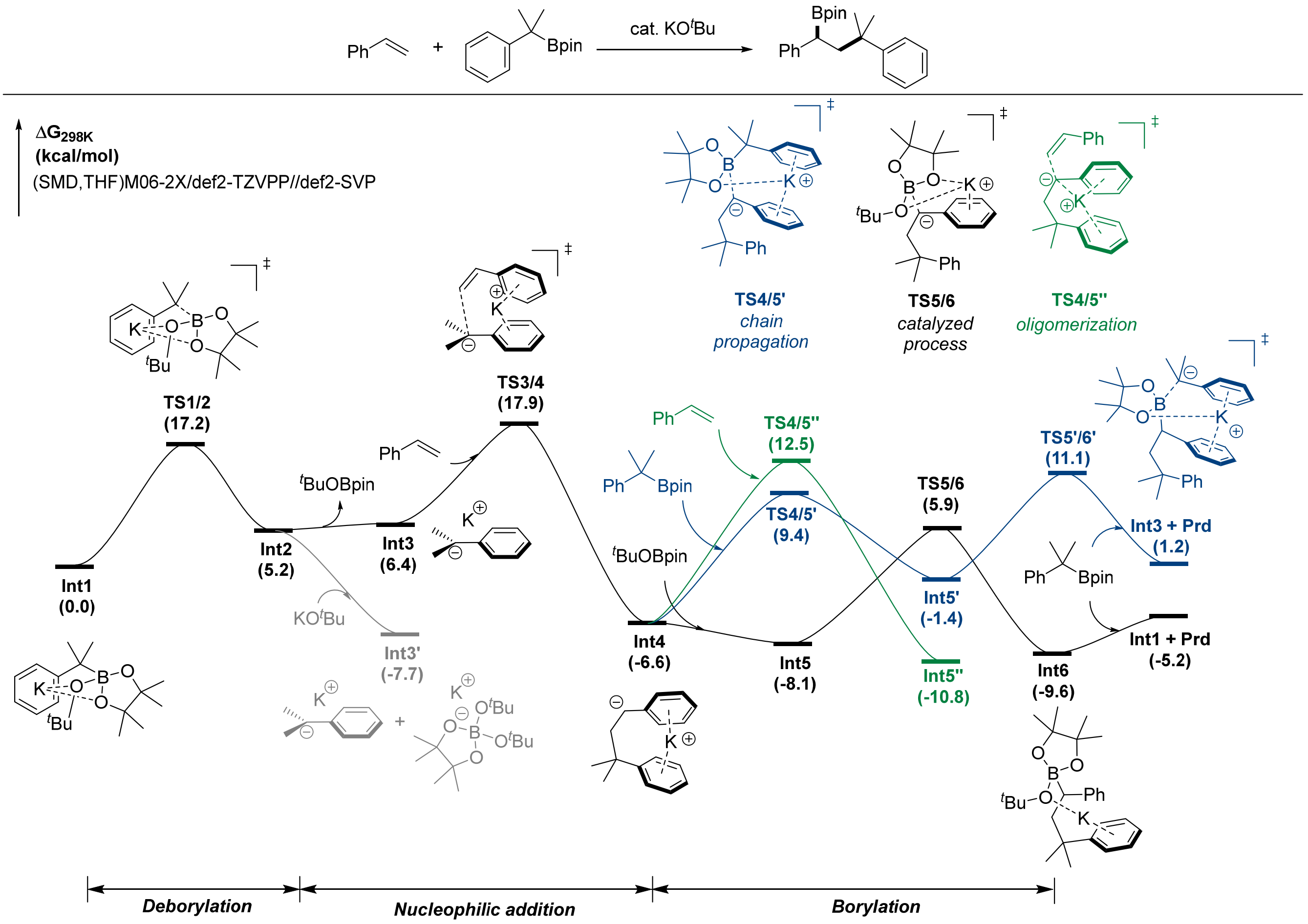

Figure S16. Gibbs free energy profile for KO^{t}Bu-catalyzed carboboration of alkene with implicit solvation model.

6.3 KO^{t}Bu-catalyzed carboboration of alkene with explicit solvent molecules

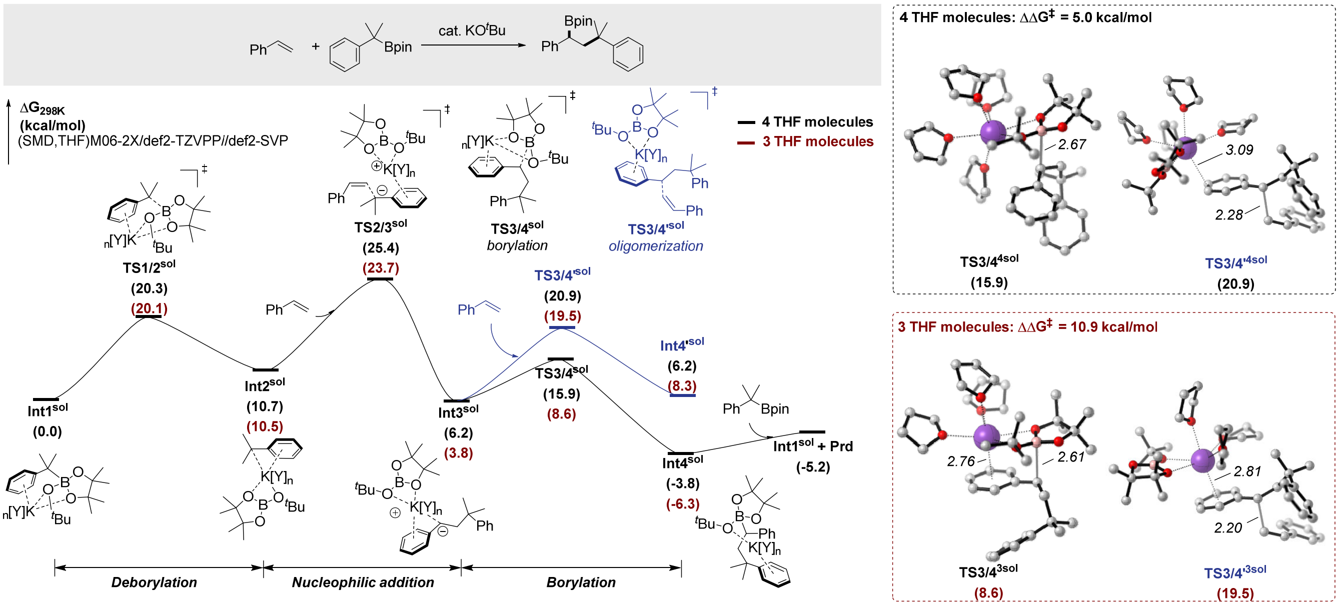

Figure S17. Gibbs free energy profile for KO^{t}Bu-catalyzed carboboration of alkene with explicit solvent molecules. H atoms are omitted in the 3D illustrations for clarity. Selected distances were labeled in Å. Color code: grey: carbon; pink: boron; red: oxygen; purple: potassium.

To better account for solvent effects, explicit THF molecules were incorporated into the computational models. Based on prior studies, potassium cations typically adopt a hexacoordinated mode in both aqueous and methanol solutions.[29] Given the potential coordinating groups in the reaction system, including the **2a**, ^{t}BuOBpin, phenyl, and vinyl moieties, two distinct coordination models were investigated: one with 3 THF molecules and another with 4 THF molecules bound to potassium. These models were manually constructed to ensure proper reactive site alignment, with only minor structural adjustments occurring after DFT optimization. In both cases, the reaction initiates via differences arising from DFT optimization for a better understanding of the effects of the coordination environment. It was found that the deborylation and nucleophilic addition steps are similar to that in implicit solvation model with only slight energy barrier difference between 3 and 4 THF models.

6.4 KO^{t}Bu-catalyzed carboboration of vinylcyclopropanes

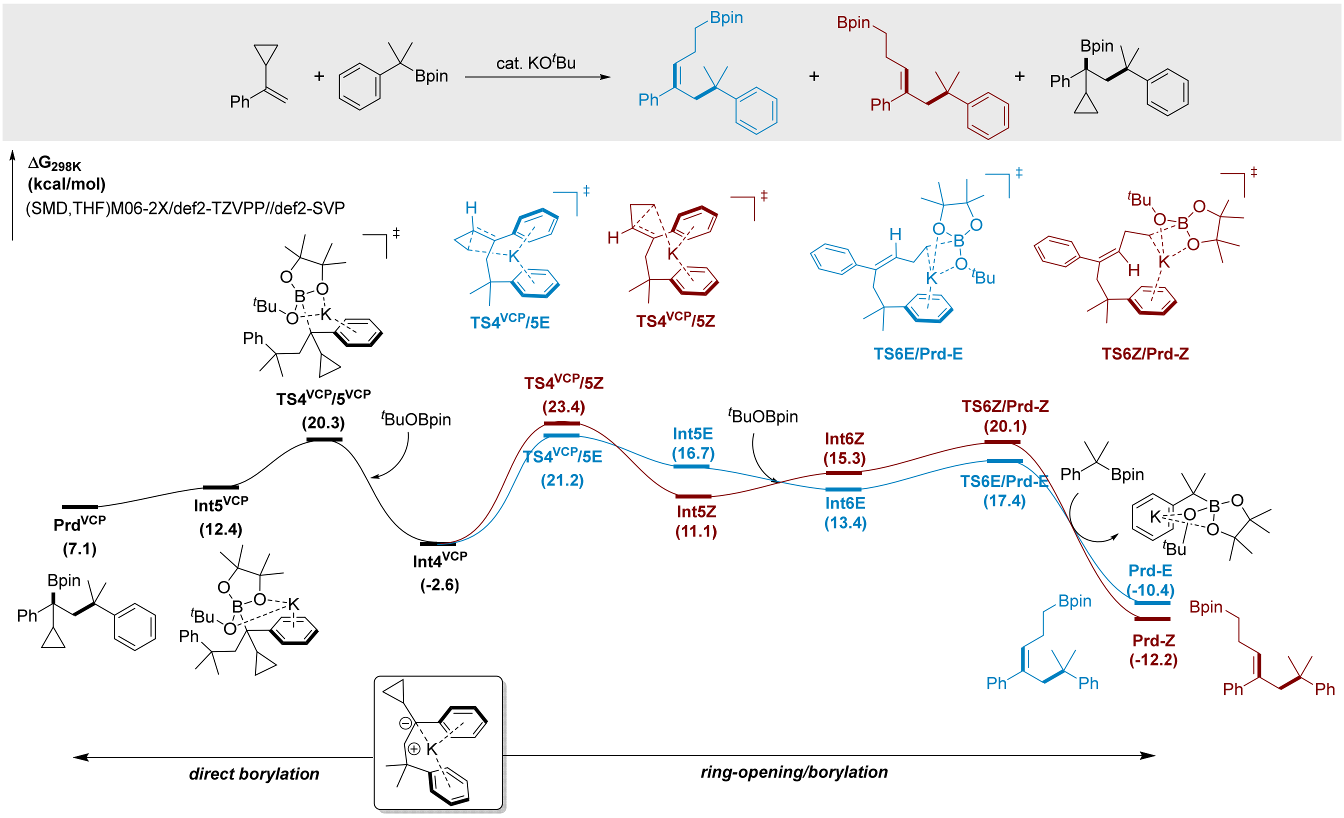

Figure S18. Gibbs free energy profile for KO^{t}Bu-catalyzed carboboration of vinylcyclopropanes (with the carbanion intermediate **Int4^{VCP}** as the start point).

Building upon the experimental evidence supporting the formation of benzylic carbanion intermediates, we conducted DFT calculations employing α-cyclopropyl styrene **1ac** as a model substrate to probe the potential anionic ring-opening pathway. The carbanion intermediate **Int4^{VCP}** was selected as the start point (as shown in **Figure S18**). The carbanion intermediate (**Int4^{VCP}**) can undergo direct borylation via transition state **TS4^{VCP}/5^{VCP}**, yielding the corresponding carboboration product (**Prd^{VCP}**). However, this pathway was found to be thermodynamically disfavored (ΔG > 0), consistent with its absence in experimental observations. Alternatively, the carbanion intermediate can undergo a ring-opening process through two distinct transition states: **TS4^{VCP}/5E** (ΔG‡ = 26.0 kcal/mol), **TS4$^{VCP/4Z}$** (ΔG‡ = 23.8 kcal/mol). The resulting ring-opened carbanion intermediates, while thermodynamically unstable, are rapidly trapped by ^{t}BuOBpin through subsequent transition states: **TS6E/Prd-E** (E-isomer pathway), **TS6Z/Prd-Z** (*Z*-isomer pathway). This sequential process ultimately affords the observed formal 1,5-carboboration products. The calculated 2.2 kcal/mol energy difference between the *E* and *Z* transition states (ΔΔG‡ = 2.2 kcal/mol) provides a quantitative explanation for the pronounced *E*-selectivity observed experimentally for most substrates. The favorable reaction pathway is summarized as follows in **Figure S19**.

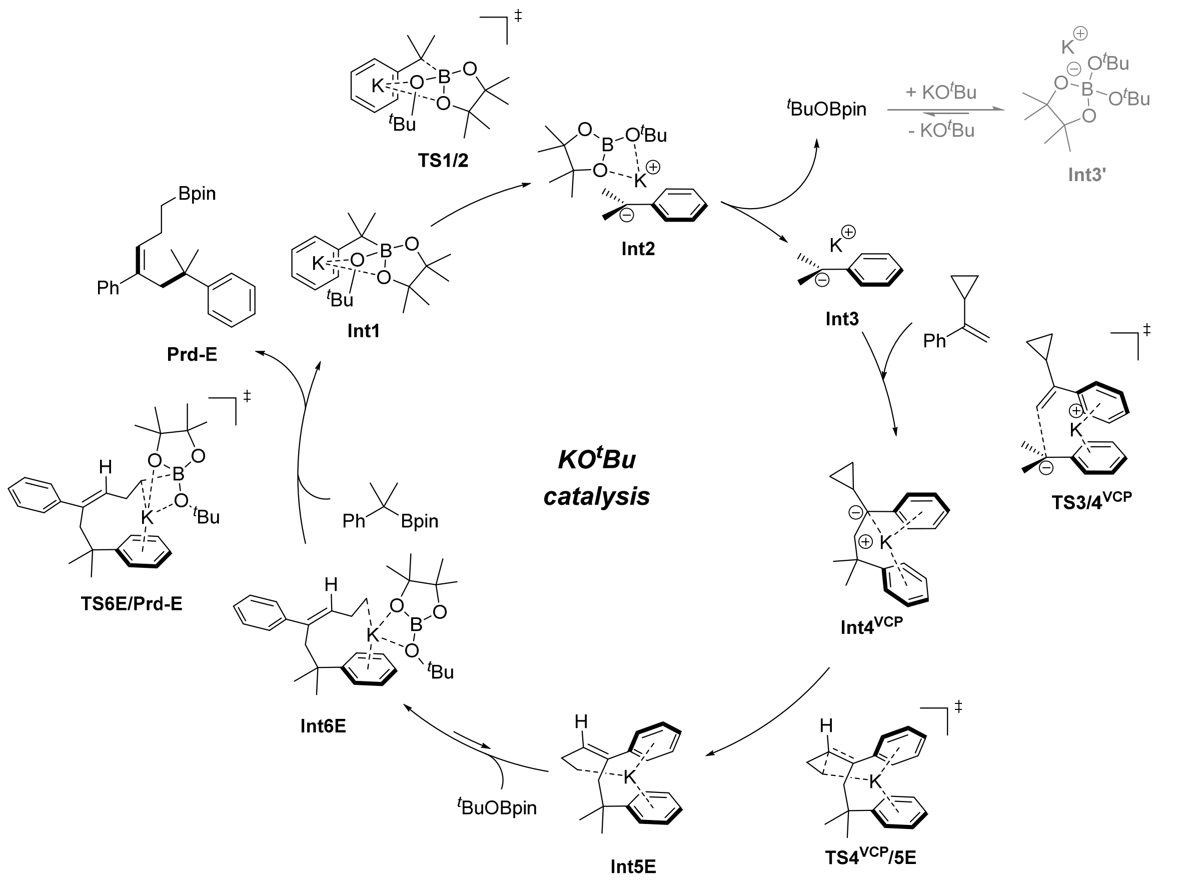

Figure S19. Proposed reaction pathway for KO^{t}Bu-catalyzed carboboration of vinylcyclopropanes.

6.5 Effects of anion parts in base catalysts

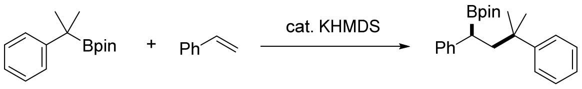

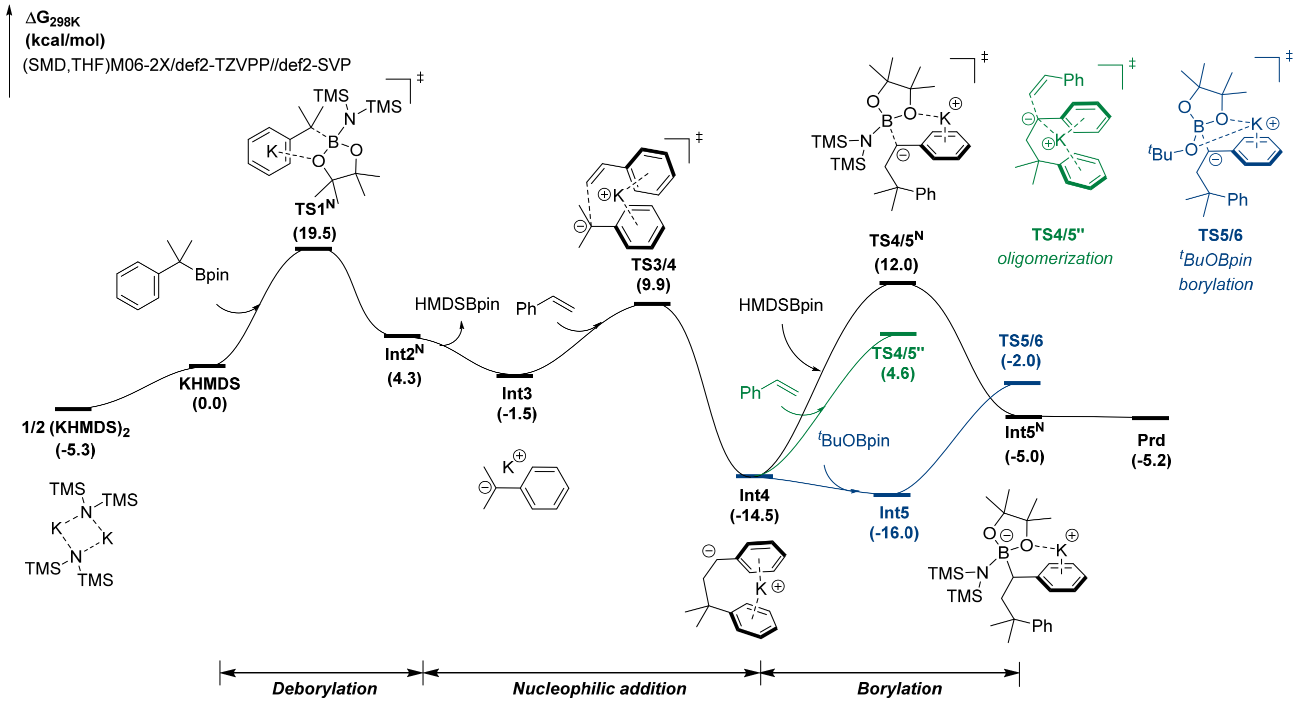

Figure S20. Gibbs free energy profile for KHMDS-catalyzed carboboration of styrene.

According to the results of reaction optimization listed in **Table S1**, the counteranion of the base is also crucial for achieving the carboboration reaction. For example, when using KHMDS as the base, the reaction only produced the carboboration product in a much lower yield (8%). To investigate the origin of the reactivity difference, DFT computations were conducted with KHMDS as the model base. As shown in **Figure S20**, in comparison with KO*t*Bu, the dimeric aggregation state of KHMDS is more thermodynamically favorable than its complexation with the benzylic boronate. This difference leads to the borylation step being the rate-determining step, and this process requires a higher energy barrier compared to the oligomerization process. The barrier height for the borylation step (**TS4/5^{N}**, $\Delta G^{\ddagger}$ = 26.5 kcal/mol) is significantly higher than that for the oligomerization (**TS4/5''**, $\Delta G^{\ddagger}$ = 19.1 kcal/mol). This difference might be attributed to the larger steric hindrance associated with the bis(trimethylsilyl)amidethe group in the base and the HMDSBpin fragment, as opposed to *t*BuOBpin. These computational results can account for the observed difference between the base of KO*t*Bu and KHMDS. Therefore, the observed reaction outcome differences of different bases are likely to be the result of the combined effects of solvation, aggregation, basicity, and steric hindrance of the anionic component of the bases.[30]

6.6 Possibility of SET processes

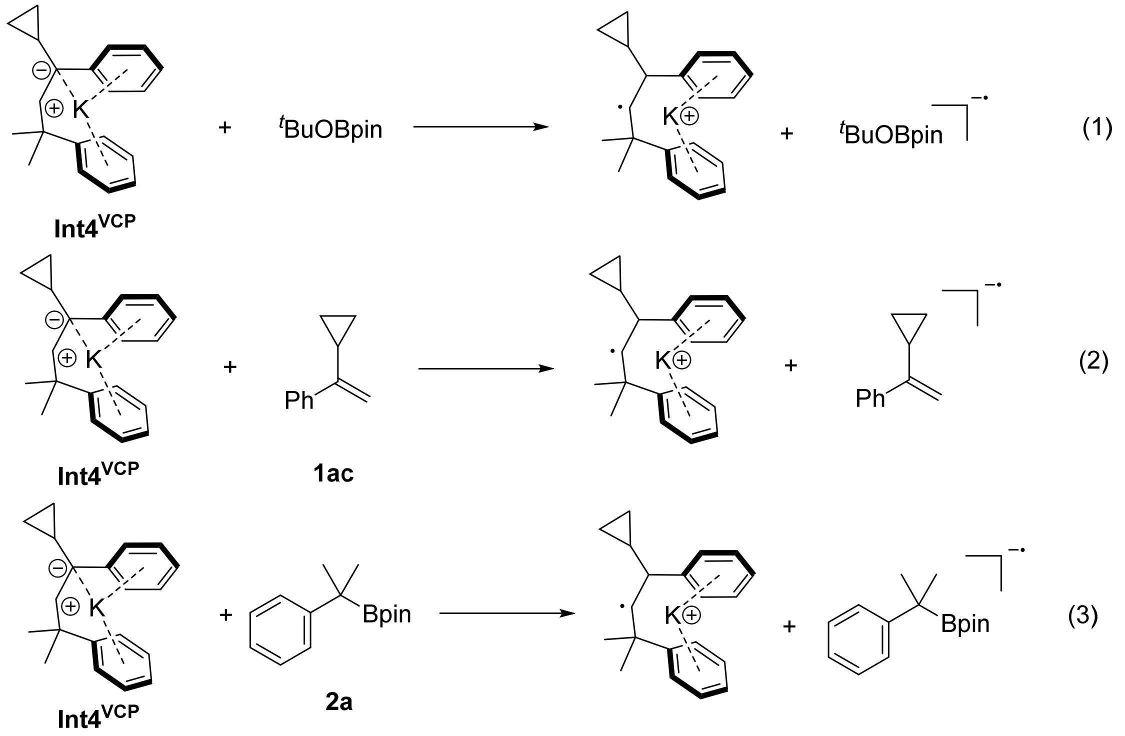

Reaction	ΔG (kcal/mol)
(1)	78.8
(2)	43.3
(3)	60.7

Figure S21. Computed reaction energies for the SET processes between the carbanion intermediate (as an electron donor) and plausible electron acceptors present.

As shown in **Figure S21**, single-electron transfer (SET) energetics were computationally evaluated between the carbanion intermediate (serving as electron donor) and plausible electron acceptors present in the reaction system. It was found that the direct single electron transfers (SET) from carbanion intermediate **Int4^{VCP}** to different species in the reaction mixture are highly endergonic, indicating that these process is less likely.

7. References

[1] M. Su, X. Huang, C. Lei, J. Jin, *Org. Lett.* **2022**, *24*, 354-358.

[2] P. Huang, Y.-X. Wang, H.-F. Yu, J.-M. Lu, *Organometallics* **2014**, *33*, 1587-1593.

[3] A. Y. Luo, Y. Bao, X.-F. Cheng, X.-S. Wang, *Synthesis* **2017**, *49*, 3962-3967.

[4] J. J. Molloy, C. P. Seath, M. J. West, C. McLaughlin, N. J. Fazakerley, A. R. Kennedy, D. J. Nelson, A. J. B. Watson, *J. Am. Chem. Soc.* **2018**, *140*, 126-130.

[5] P.-W. Long, T. He, M. Oestreich, *Org. Lett.* **2020**, *22*, 7383-7386.

[6] C. Chen, H. Wang, T. Li, D. Lu, J. Li, X. Zhang, X. Hong, Z. Lu, *Angew. Chem. Int. Ed.* **2022**, *61*, e202205619.

[7] a) J. L. Stymiest, V. Bagutski, R. M. French, V. K. Aggarwal, *Nature* **2008**, *456*, 778-782; b) V. Bagutski, R. M. French, V. K. Aggarwal, *Angew. Chem. Int. Ed.* **2010**, *49*, 5142-5145.

[8] T. Liu, C. Li, J. Bai, P. Zhang, Y. Guo, X. Wang, *Chin. J. Chem .* **2022**, *40*, 2203-2211.

[9] J. Blunt, J. Coxon, W. Robinson, H. Schuyt, *Aust. J. Chem.* **1983**, *36*, 565-579.

[10] M. G. Davidson, D. Garcia-Vivo, A. R. Kennedy, R. E. Mulvey, S. D. Robertson, *Chem. Eur. J.* **2011**, *17*, 3364-3369.

[11] X. Cai, A. Keshavarz, J. D. Omaque, B. J. Stokes, *Org. Lett.* **2017**, *19*, 2626-2629.

[12] S. Kundu, S. Sinhababu, M. M. Siddiqui, A. V. Luebben, B. Dittrich, T. Yang, G. Frenking, H. W. Roesky, *J. Am. Chem. Soc.* **2018**, *140*, 9409-9412.

[13] C. Hansch, A. Leo, R. W. Taft, *Chem. Rev.* **1991**, *91*, 165-195.

[14] R. W. Taft, Jr., I. C. Lewis, *J. Am. Chem. Soc.* **1958**, *80*, 2436-2443.

[15] R. W. Taft, R. D. Topsom, in *Progress in Physical Organic Chemistry*, **1987**, pp. 1-83.

[16] L. P. Hammett, *J. Am. Chem. Soc.* **1937**, *59*, 96-103.

[17] H. C. Brown, Y. Okamoto, *J. Am. Chem. Soc.* **1958**, *80*, 4979-4987.

[18] H. Akaike, *IEEE Transactions on Automatic Control* **1974**, *19*, 716-723.

[19] C. B. Santiago, J.-Y. Guo, M. S. Sigman, *Chem. Sci.* **2018**, *9*, 2398-2412.

[20] M. J. Frisch, G. W. Trucks, H. B. Schlegel, G. E. Scuseria, M. A. Robb, J. R. Cheeseman, G. Scalmani, V. Barone, G. A. Petersson, H. Nakatsuji, X. Li, M. Caricato, A. V. Marenich, J. Bloino, B. G. Janesko, R. Gomperts, B. Mennucci, H. P. Hratchian, J. V. Ortiz, A. F. Izmaylov, J. L. Sonnenberg, Williams, F. Ding, F. Lipparini, F. Egidi, J. Goings, B. Peng, A. Petrone, T. Henderson, D. Ranasinghe, V. G. Zakrzewski, J. Gao, N. Rega, G. Zheng, W. Liang, M. Hada, M. Ehara, K. Toyota, R. Fukuda, J. Hasegawa, M. Ishida, T. Nakajima, Y. Honda, O. Kitao, H. Nakai, T. Vreven, K. Throssell, J. A. Montgomery Jr., J. E. Peralta, F. Ogliaro, M. J. Bearpark, J. J. Heyd, E. N. Brothers, K. N. Kudin, V. N. Staroverov, T. A. Keith, R. Kobayashi, J. Normand, K. Raghavachari, A. P. Rendell, J. C. Burant, S. S. Iyengar, J. Tomasi, M. Cossi, J. M. Millam, M. Klene, C. Adamo, R. Cammi, J. W. Ochterski, R. L. Martin, K. Morokuma, O. Farkas, J. B. Foresman, D. J. Fox, Wallingford, CT, **2016**.

[21] C. Y. Legault, 1.0b ed., Université de Sherbrooke, **2009**.

[22] Y. Zhao, D. G. Truhlar, *Theor. Chem. Acc.* **2008**, *120*, 215-241.

[23] a) F. Weigend, R. Ahlrichs, *Phys. Chem. Chem. Phys.* **2005**, *7*, 3297-3305; b) F. Weigend, *Phys. Chem. Chem. Phys.* **2006**, *8*, 1057-1065.

[24] A. V. Marenich, C. J. Cramer, D. G. Truhlar, *J. Phys. Chem. B* **2009**, *113*, 6378-6396.

[25] F. Jensen, *J. Chem. Theory Comput.* **2015**, *11*, 132-138.

[26] P. J. Wilson, T. J. Bradley, D. J. Tozer, *The Journal of Chemical Physics* **2001**, *115*, 9233-9242.

[27] R. A. Marcus, *Annu. Rev. Phys. Chem.* **1964**, *15*, 155-196.

[28] a) M. Yang, L. Yang, G. Wang, Y. Zhou, D. Xie, S. Li, *J. Chem. Theory Comput.* **2018**, *14*, 5787-5796; b) G. Li, Z. Li, L. Gao, S. Chen, G. Wang, S. Li, *Phys. Chem. Chem. Phys.* **2023**, *25*, 23696-23707.

[29] P. R. Smirnov, *Russ. J. Gen. Chem.* **2020**, *90*, 1693-1702.

[30] K. J. Msayib, C. I. F. Watt, *Chem. Soc. Rev.* **1992**, *21*, 237-243.

8. NMR Spectra

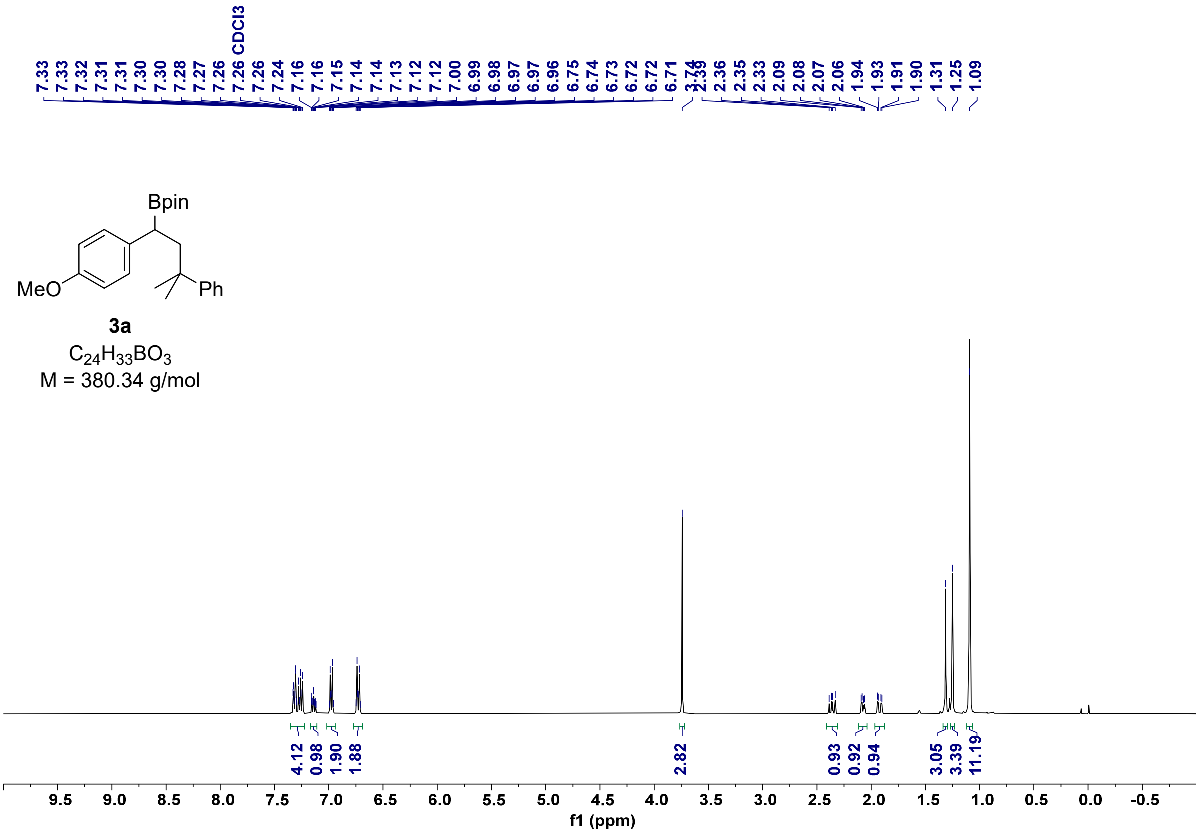

^{1}H NMR spectrum (400 MHz, $CDCl_3$) of compound **3a**.

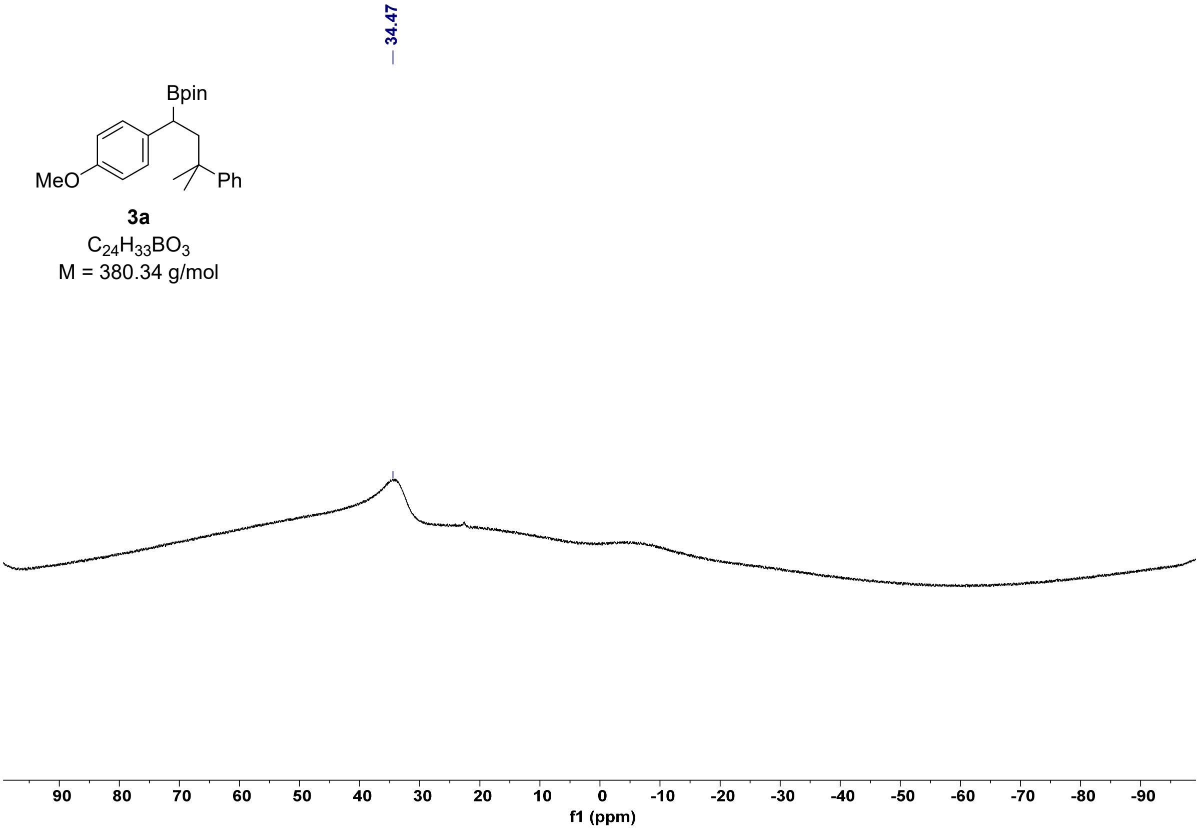

^{11}B NMR spectrum (400 MHz, $CDCl_3$) of compound **3a**.

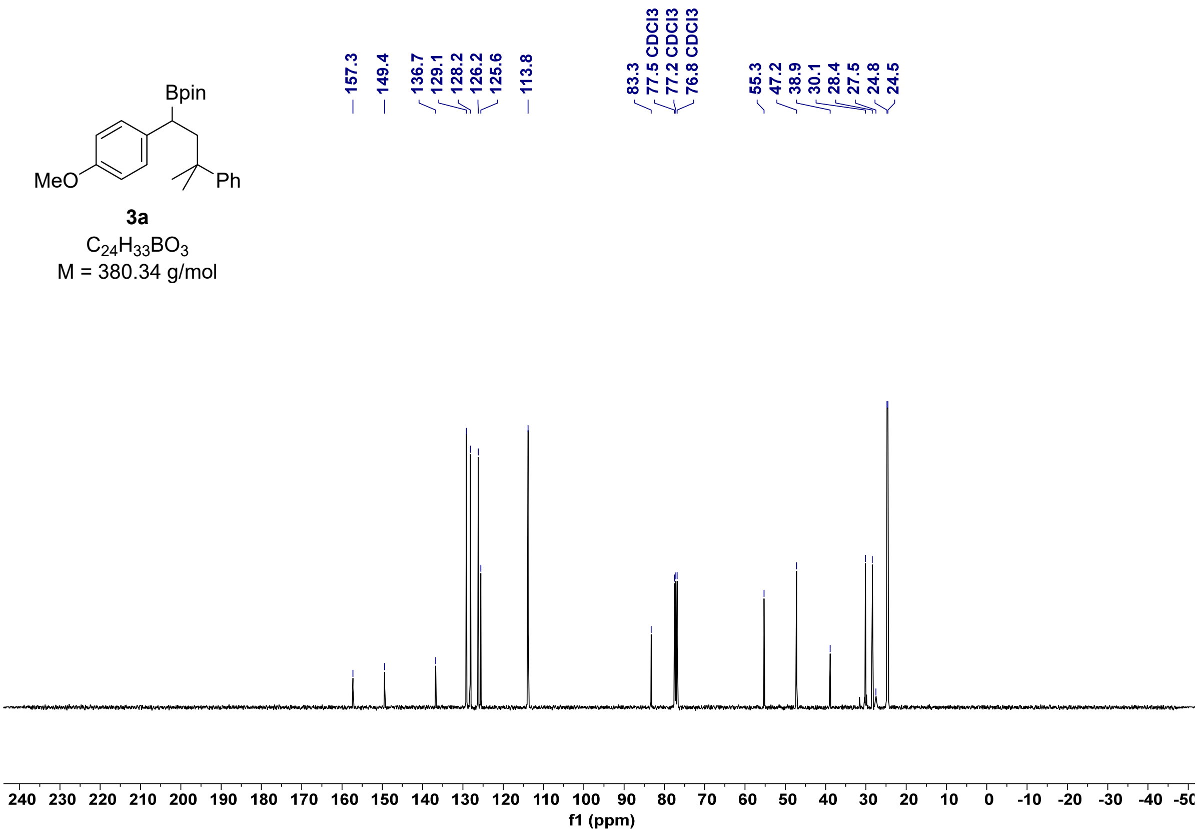

$^{13}C\{^1H\}$ NMR spectrum (100 MHz, $CDCl_3$) of compound **3a**.

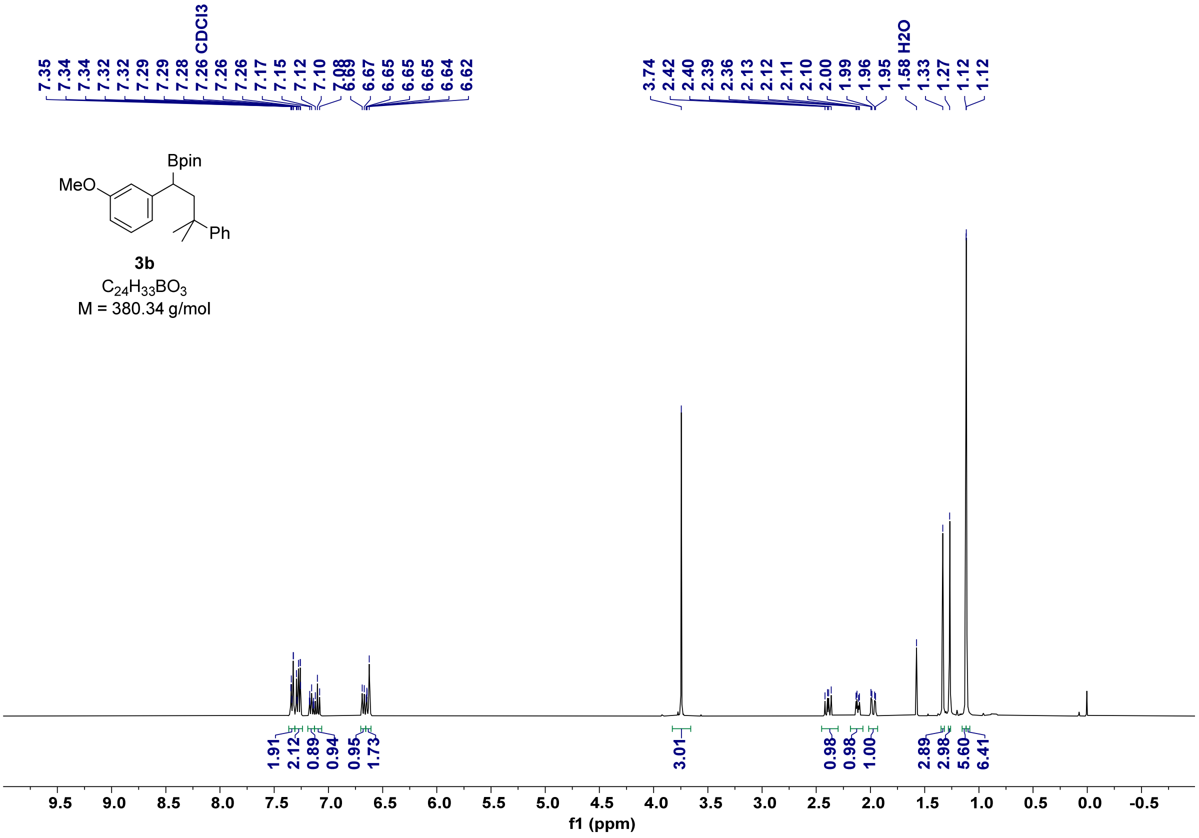

^1H NMR spectrum (400 MHz, $CDCl_3$) of compound **3b**.

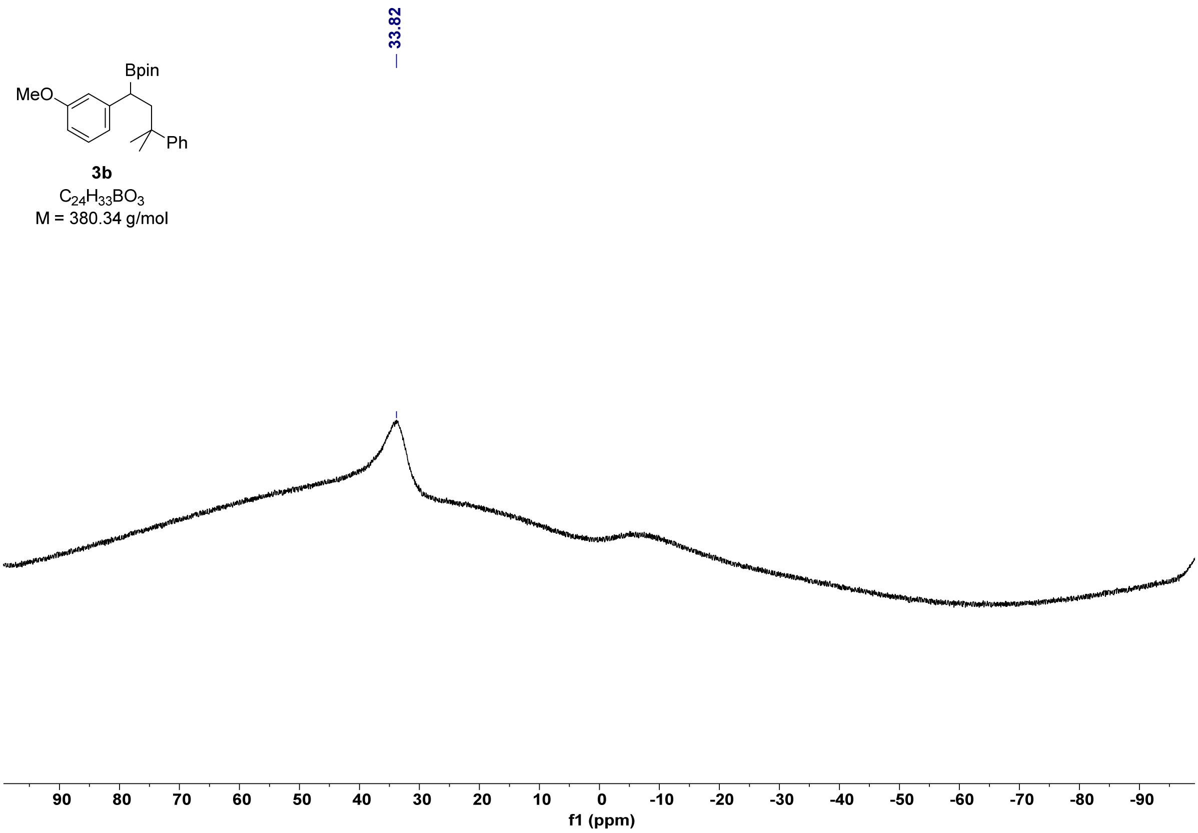

^{11}B NMR spectrum (400 MHz, $CDCl_3$) of compound **3b**.

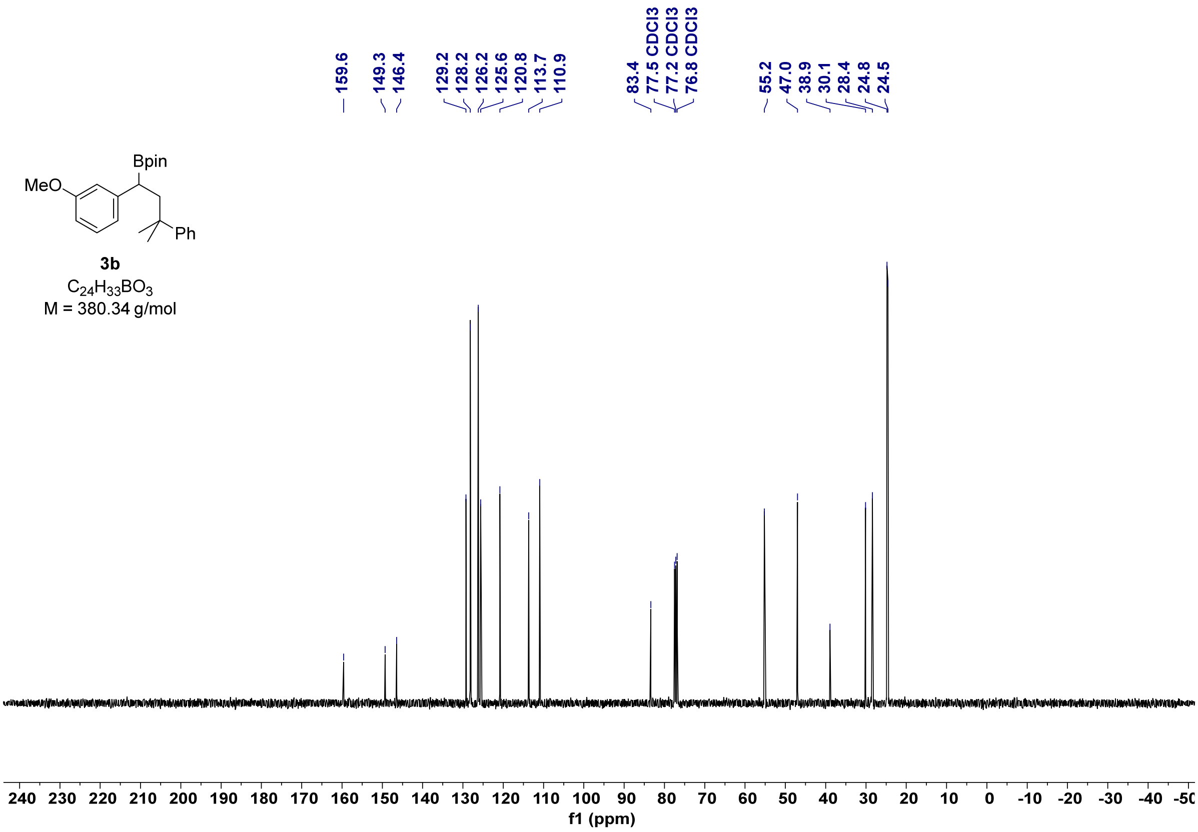

^{13}C{^{1}H} NMR spectrum (100 MHz, $CDCl_3$) of compound **3b**.

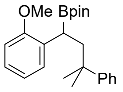

3c
$C_{24}H_{33}BO_3$
M = 380.34 g/mol

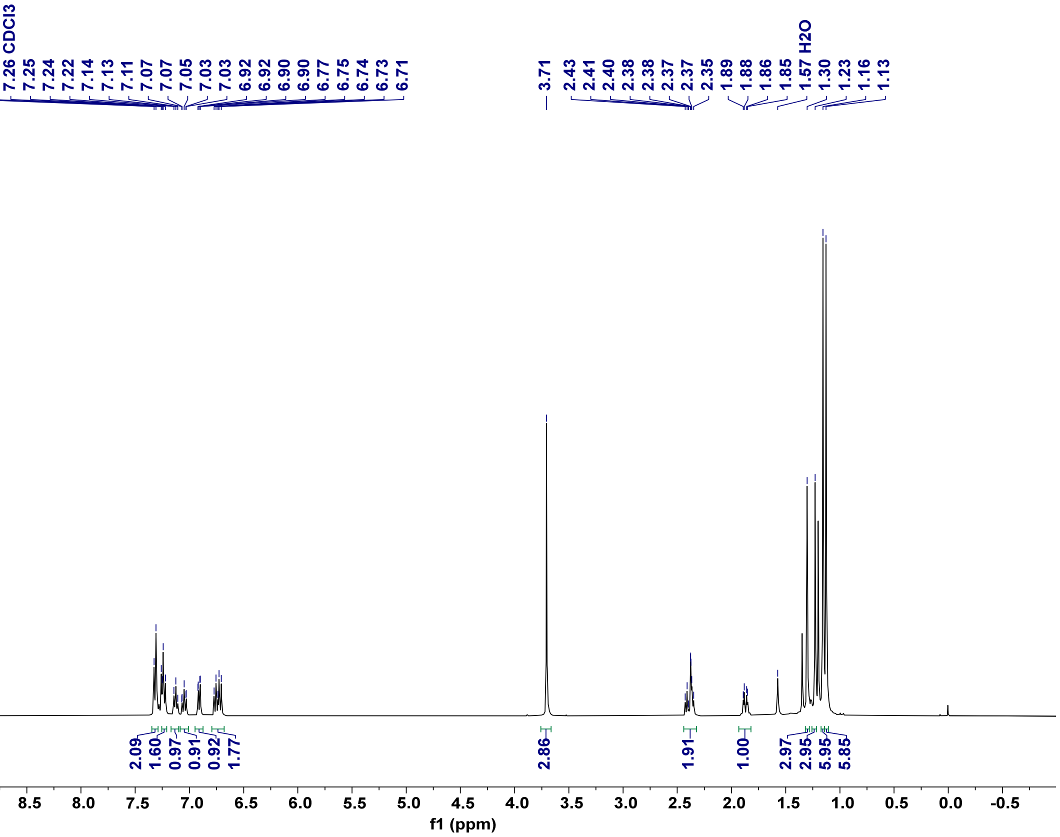

^{1}H NMR spectrum (400 MHz, $CDCl_3$) of compound **3c**.

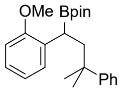

3c
$C_{24}H_{33}BO_3$
M = 380.34 g/mol

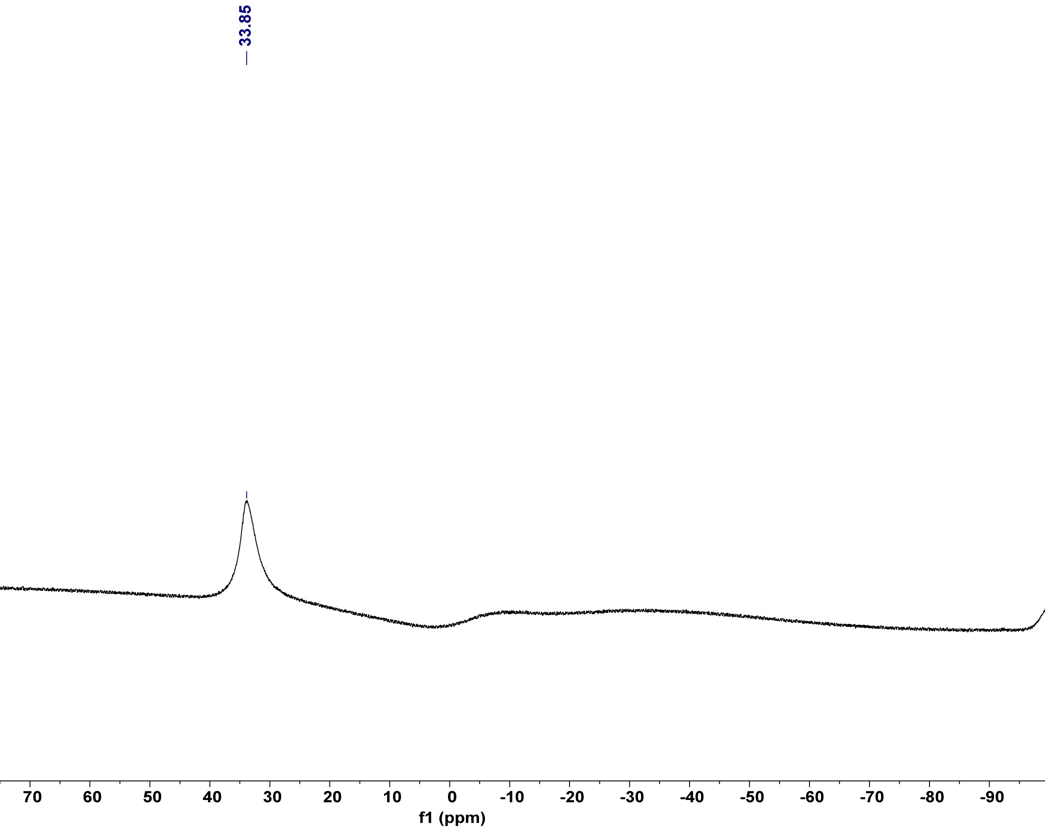

^{11}B NMR spectrum (128 MHz, $CDCl_3$) of compound **3c**.

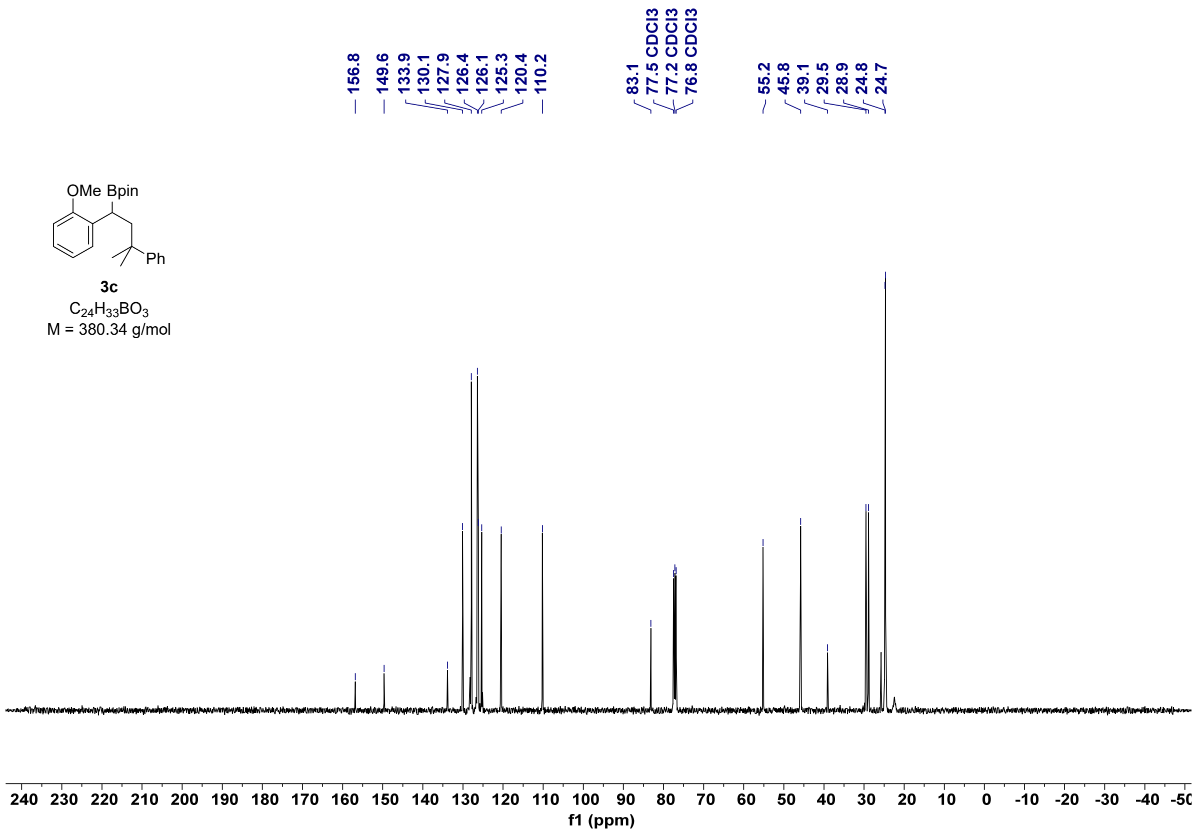

$^{13}C\{^1H\}$ NMR spectrum (100 MHz, $CDCl_3$) of compound **3c**.

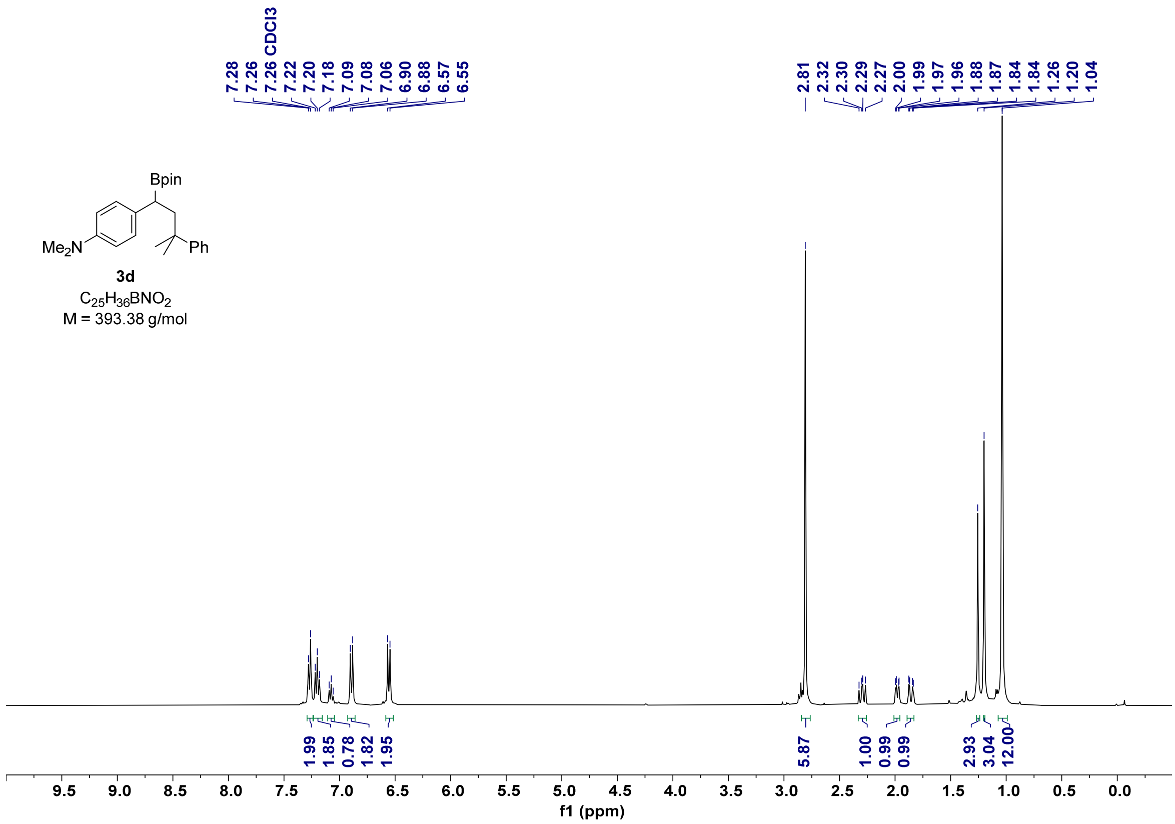

^1H NMR spectrum (400 MHz, $CDCl_3$) of compound **3d**.

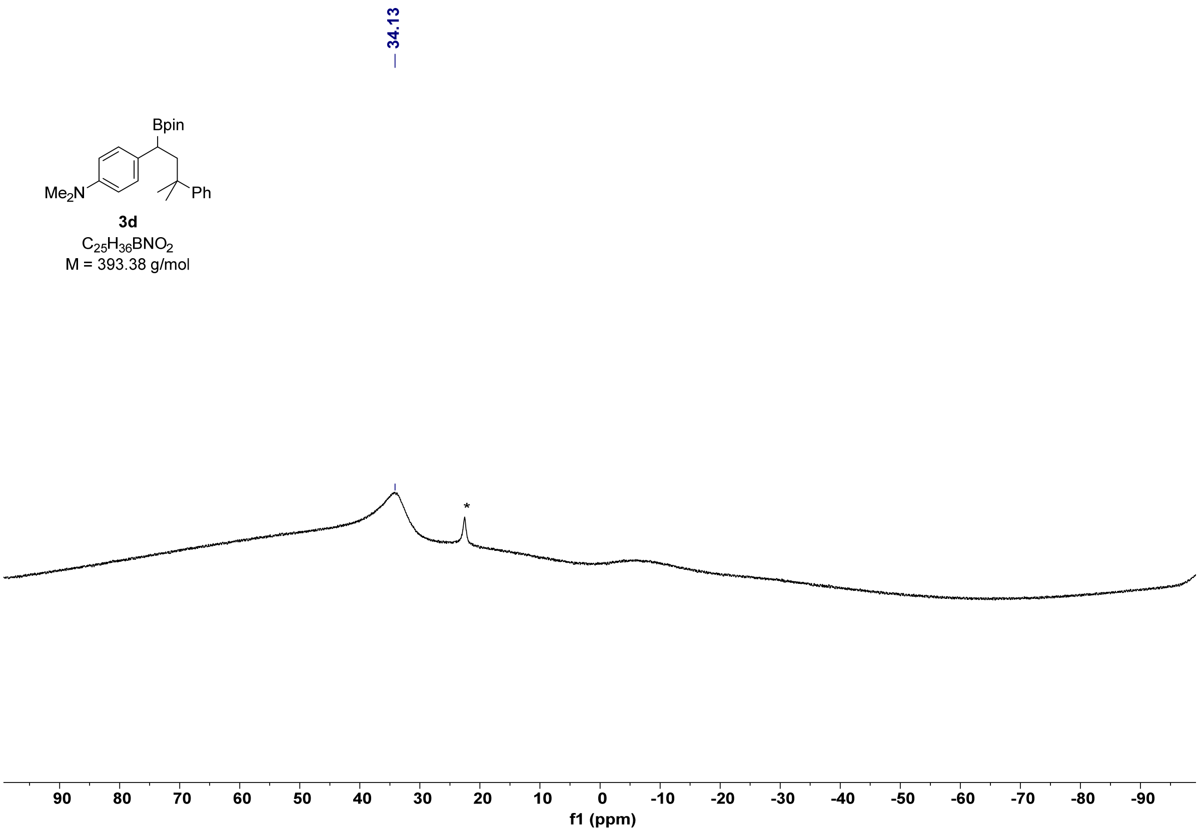

^{11}**B NMR** spectrum (128 MHz, $CDCl_3$) of compound **3d**. * Impurity due to decomposition during purification.

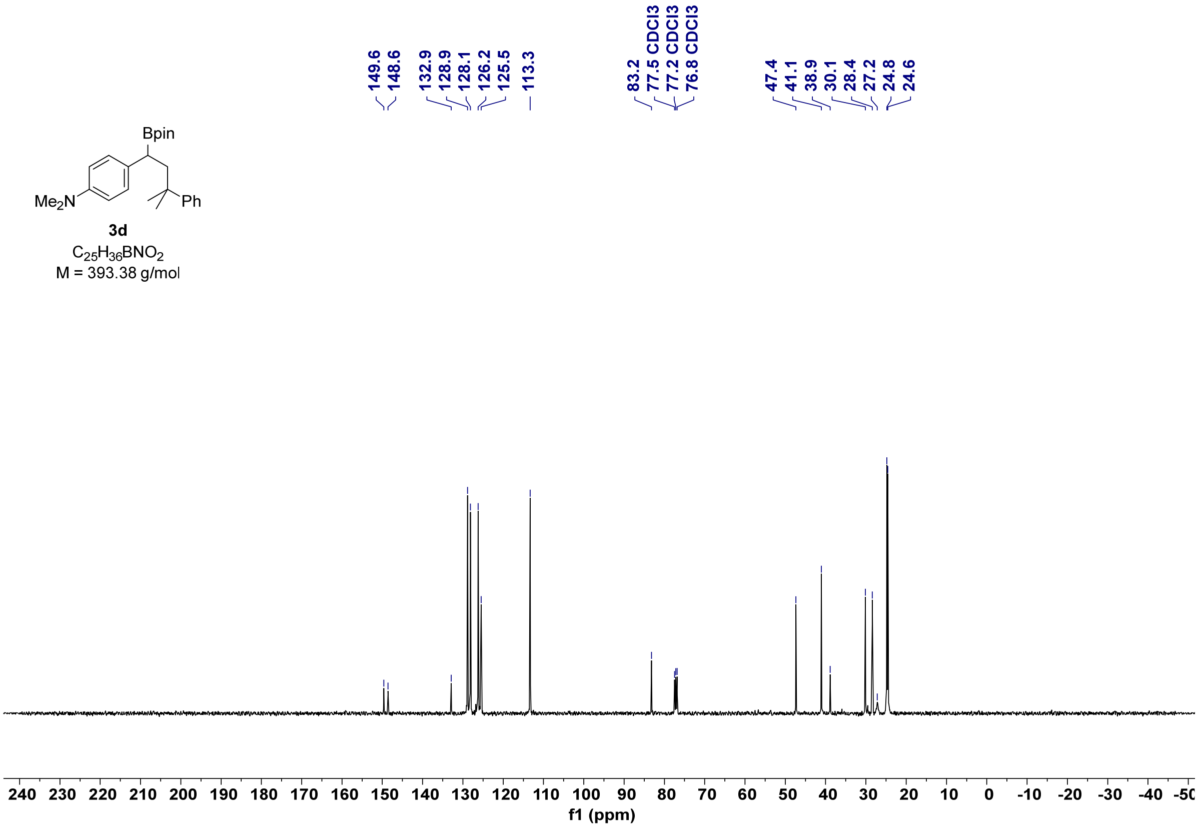

^{13}**C{^{1}H} NMR** spectrum (100 MHz, $CDCl_3$) of compound **3d**.

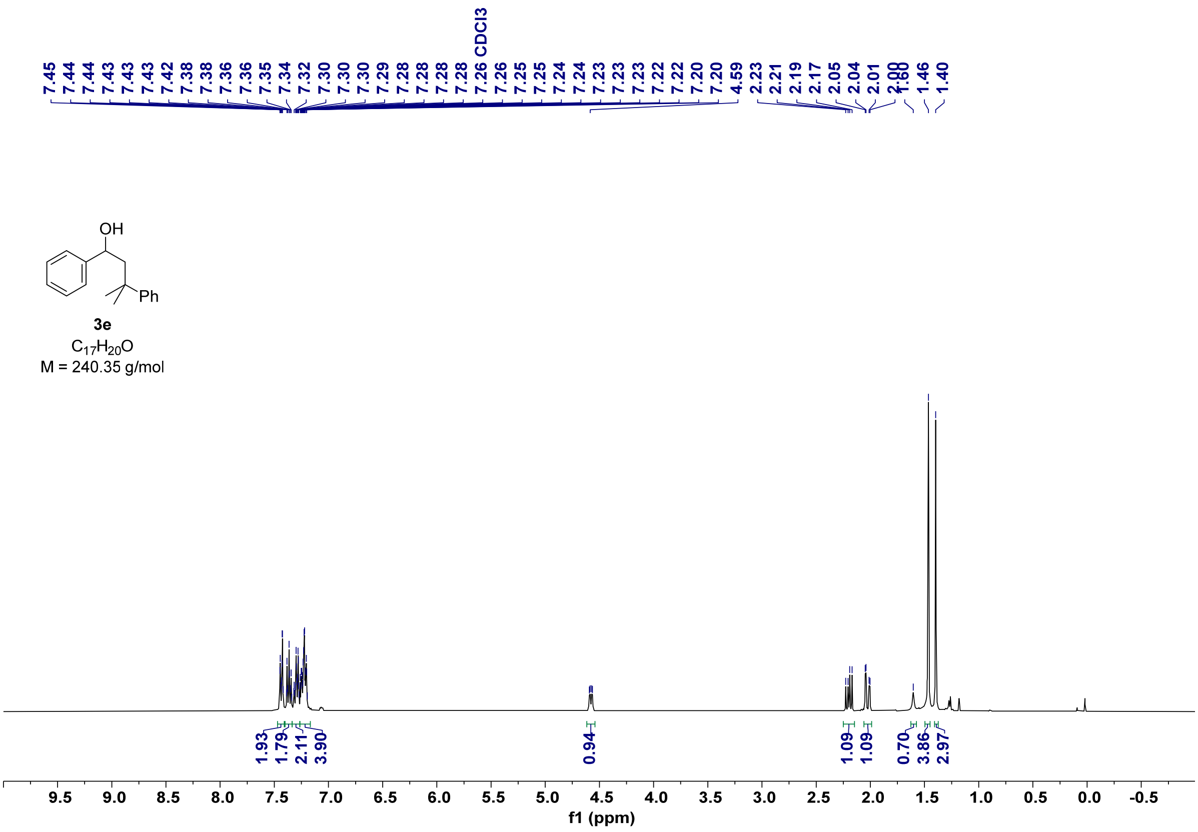

^{1}H NMR spectrum (400 MHz, $CDCl_3$) of compound **3e**.

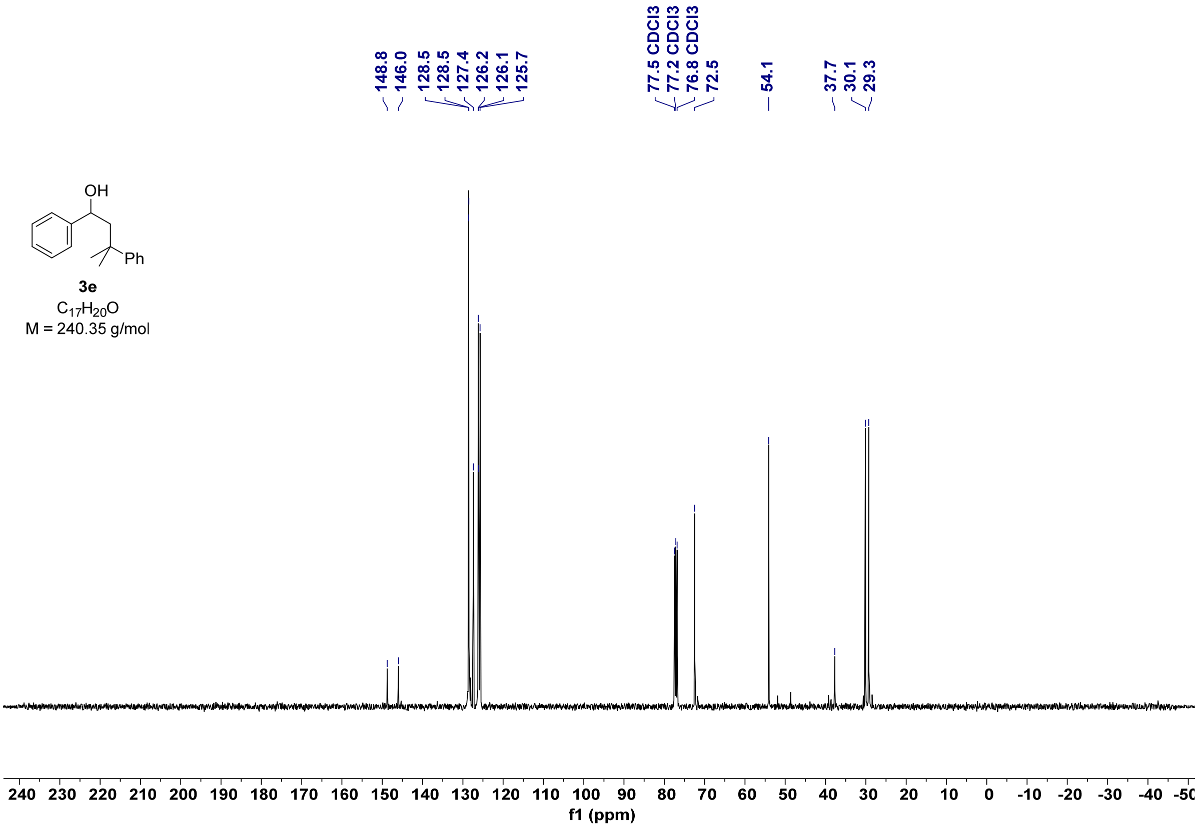

^{13}C{^{1}H} NMR spectrum (100 MHz, $CDCl_3$) of compound **3e**.

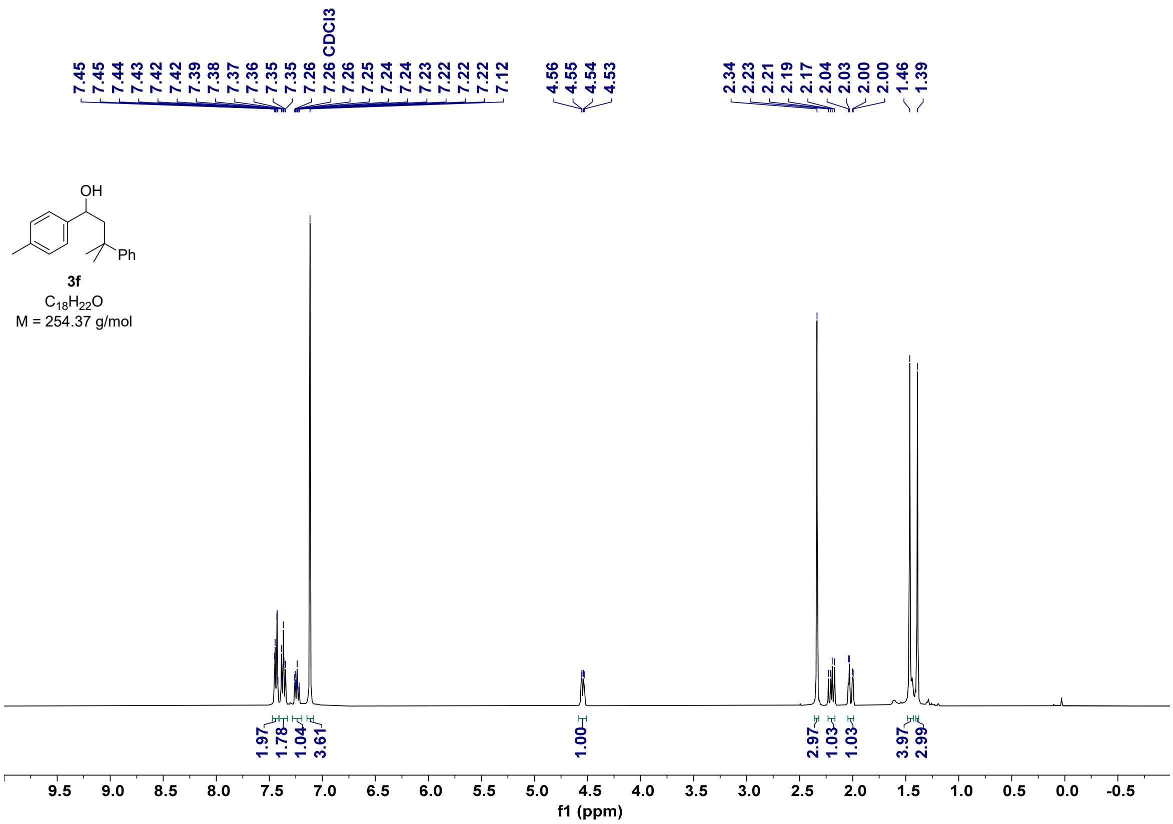

^{1}H NMR spectrum (400 MHz, $CDCl_3$) of compound **3f**.

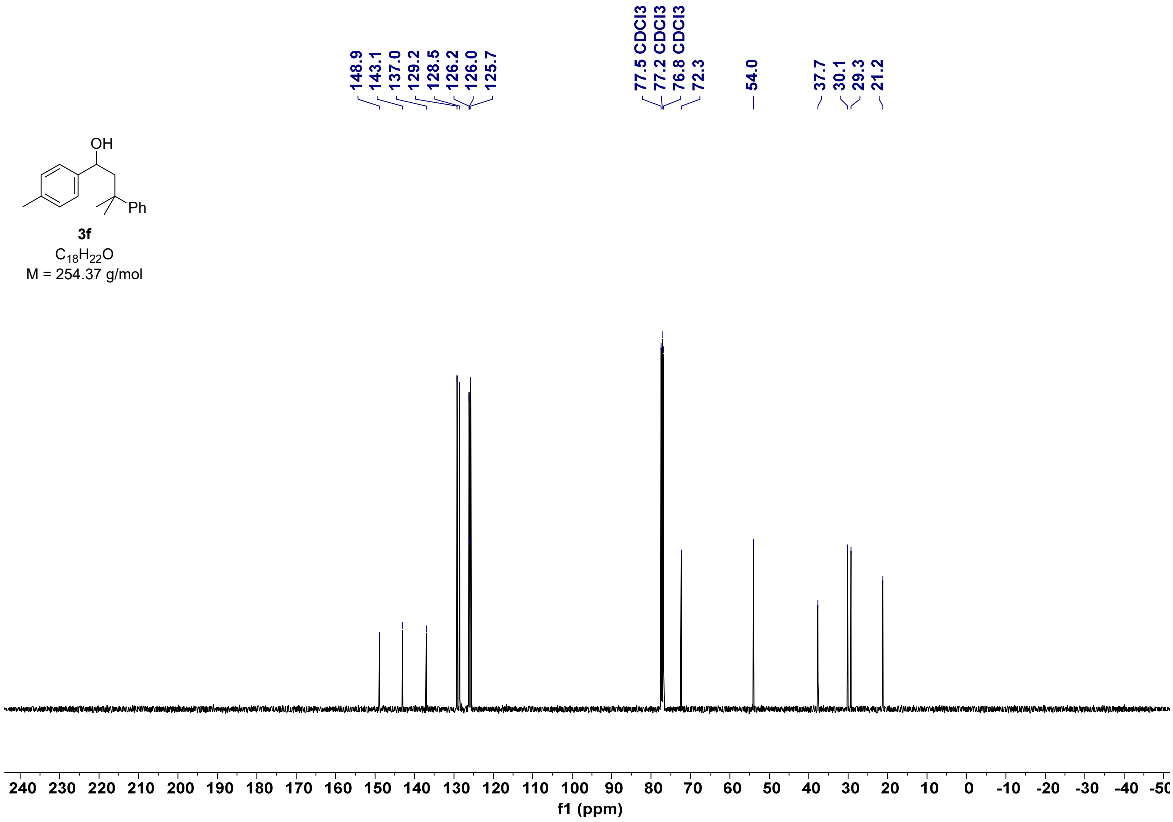

$^{13}C\{^1H\}$ NMR spectrum (100 MHz, $CDCl_3$) of compound **3f**.

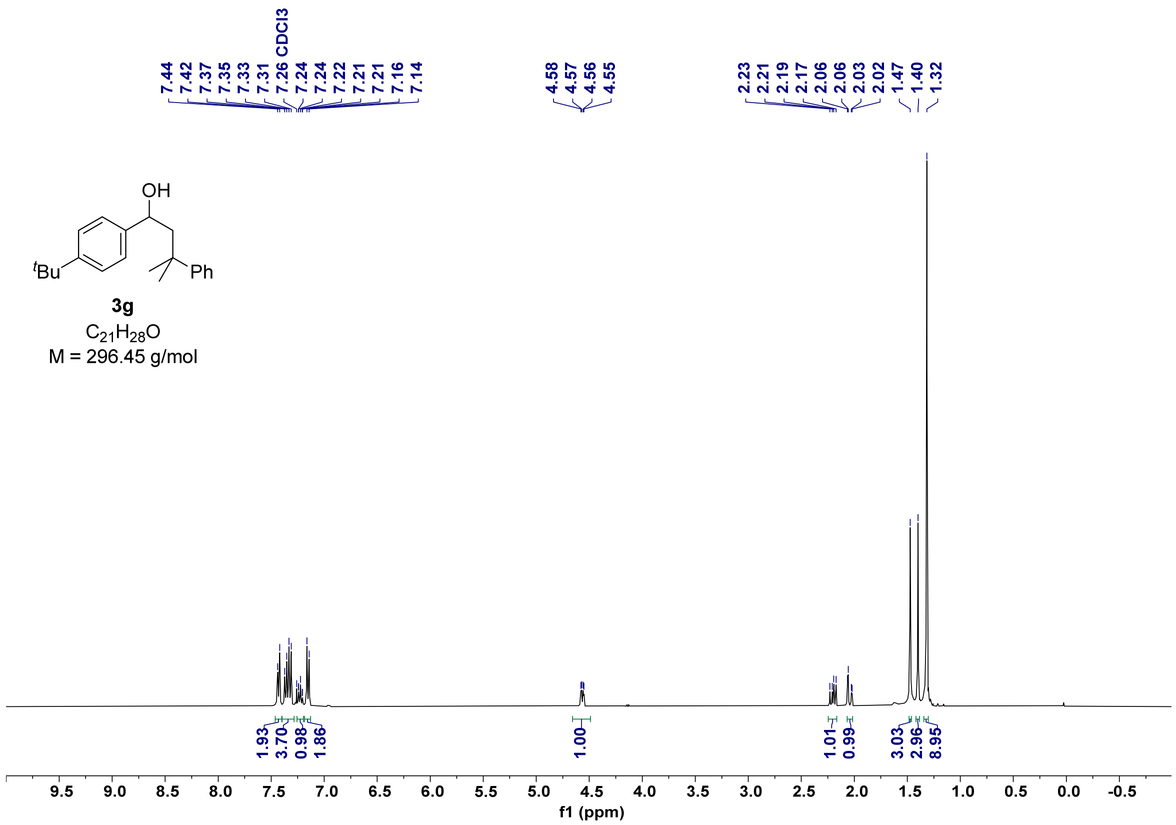

^{1}**H NMR** spectrum (400 MHz, $CDCl_3$) of compound **3g**.

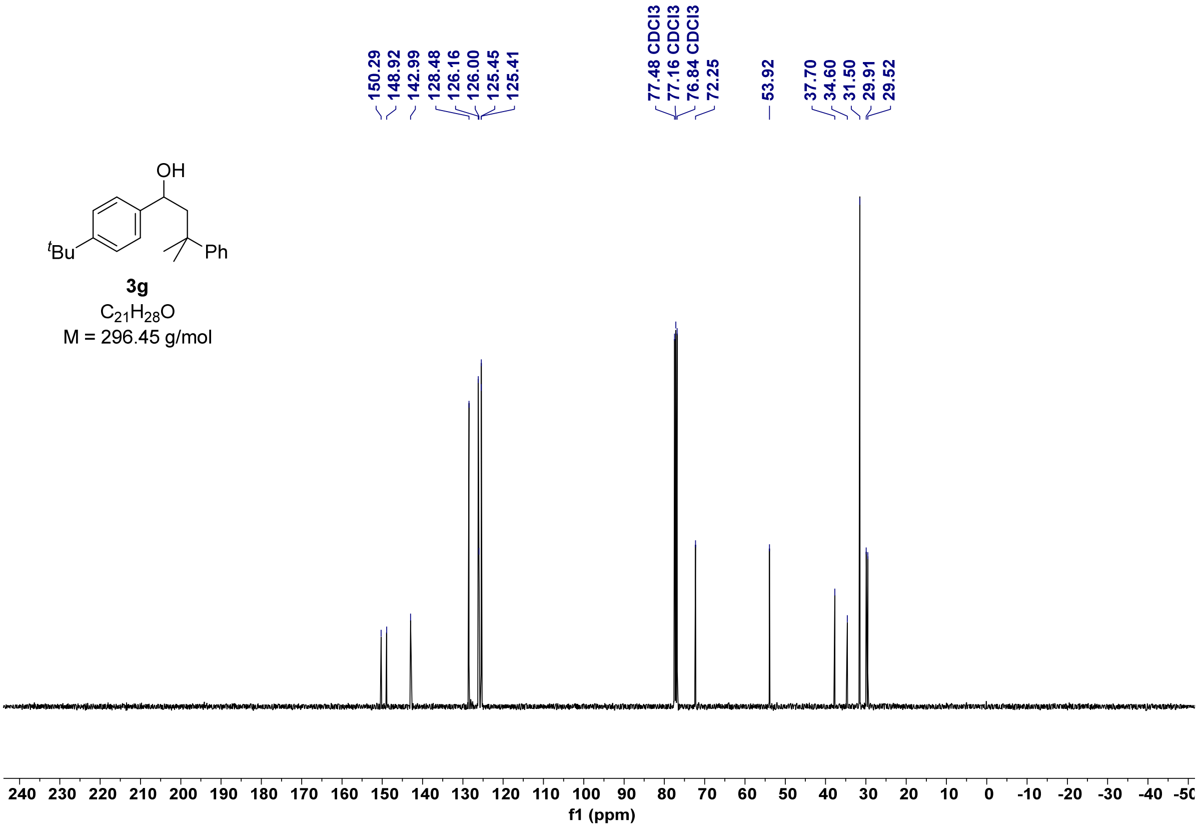

^{13}**C{^{1}H} NMR** spectrum (100 MHz, $CDCl_3$) of compound **3g**.

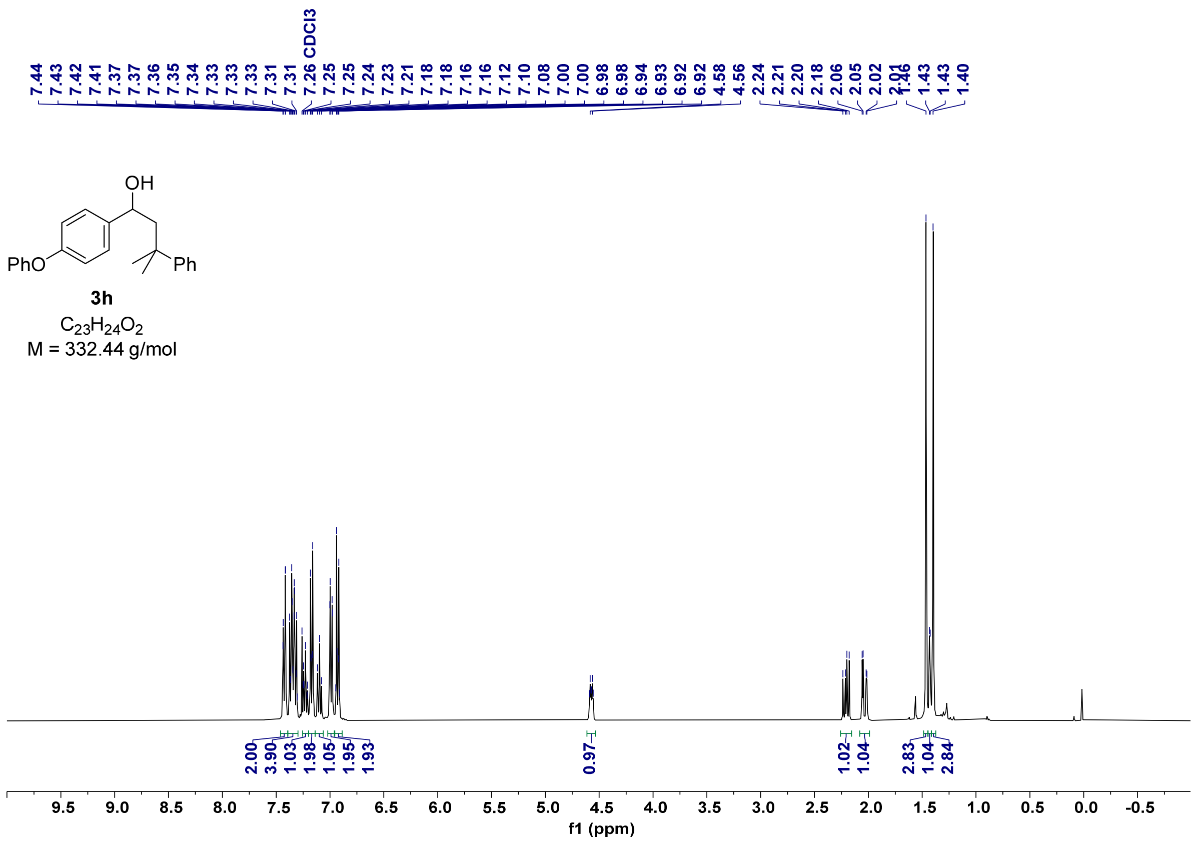

^{1}**H NMR** spectrum (400 MHz, $CDCl_3$) of compound **3h**.

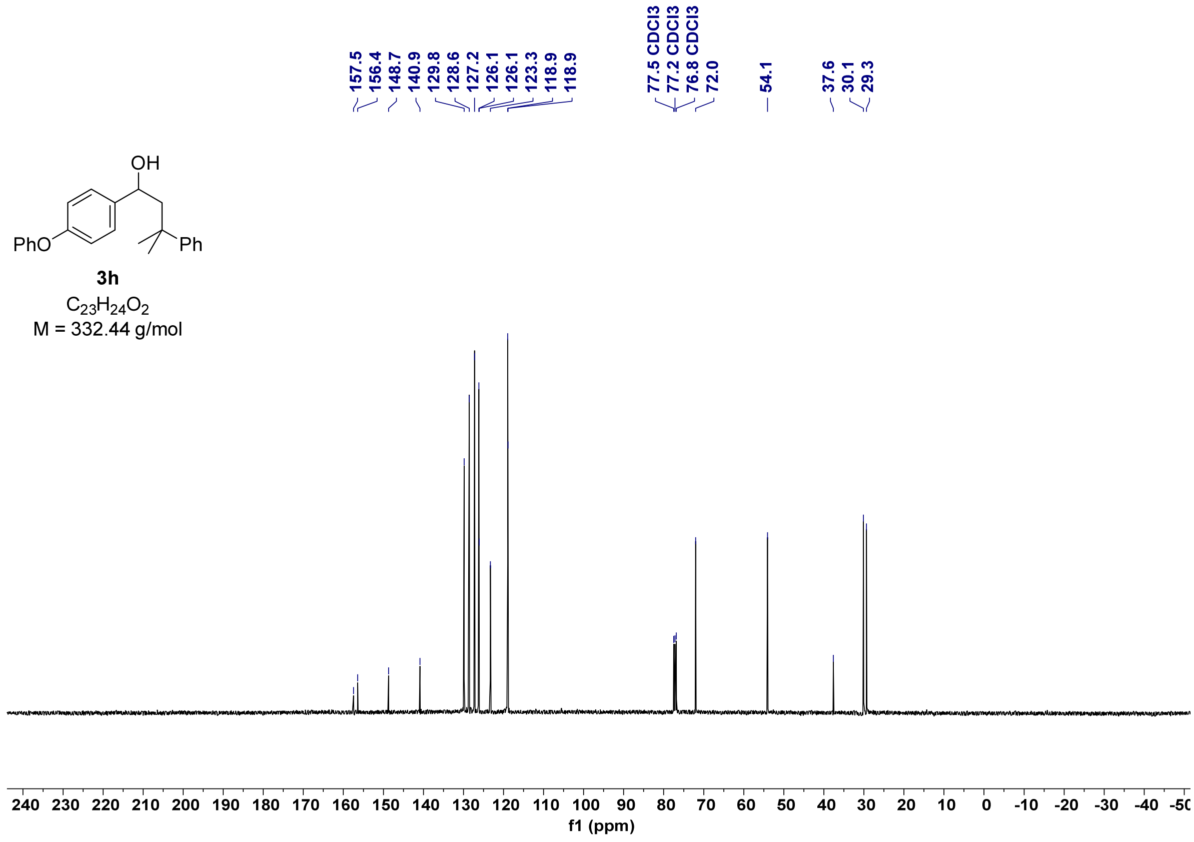

^{13}**C{^{1}H} NMR** spectrum (100 MHz, $CDCl_3$) of compound **3h**.

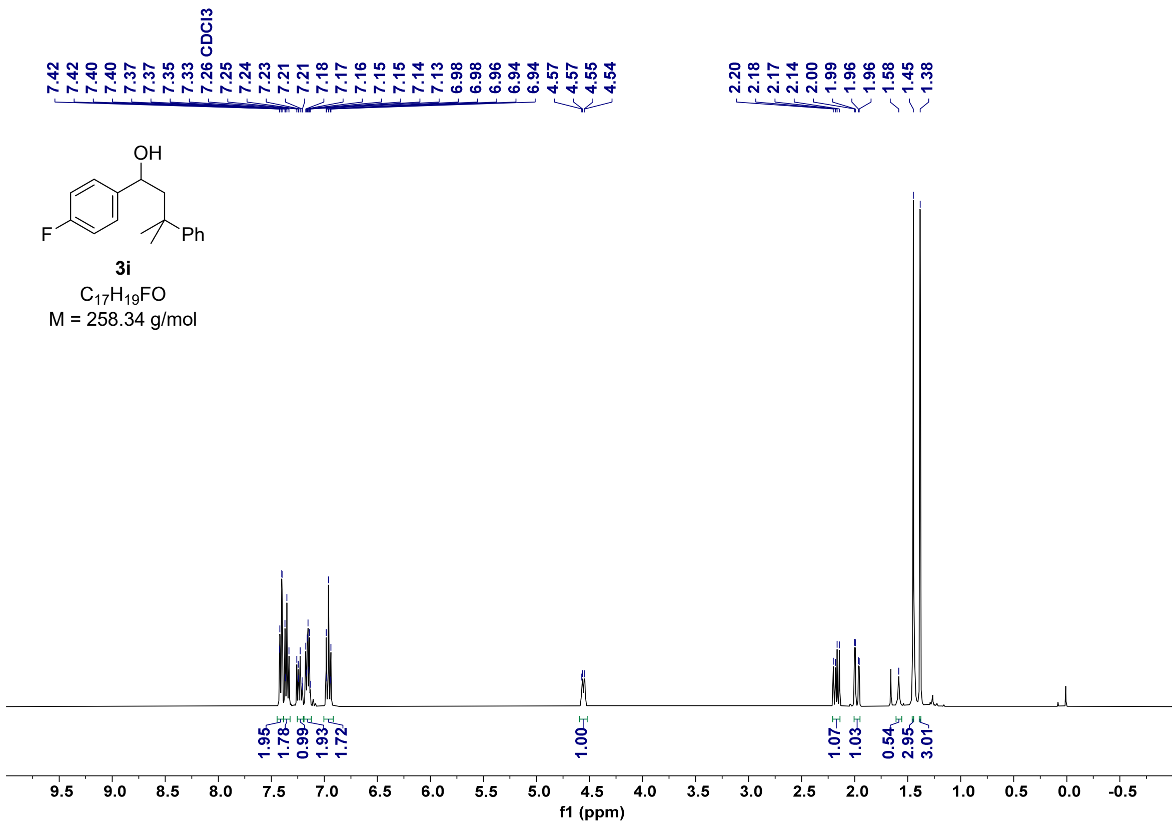

^{1}H NMR spectrum (400 MHz, $CDCl_3$) of compound **3i**.

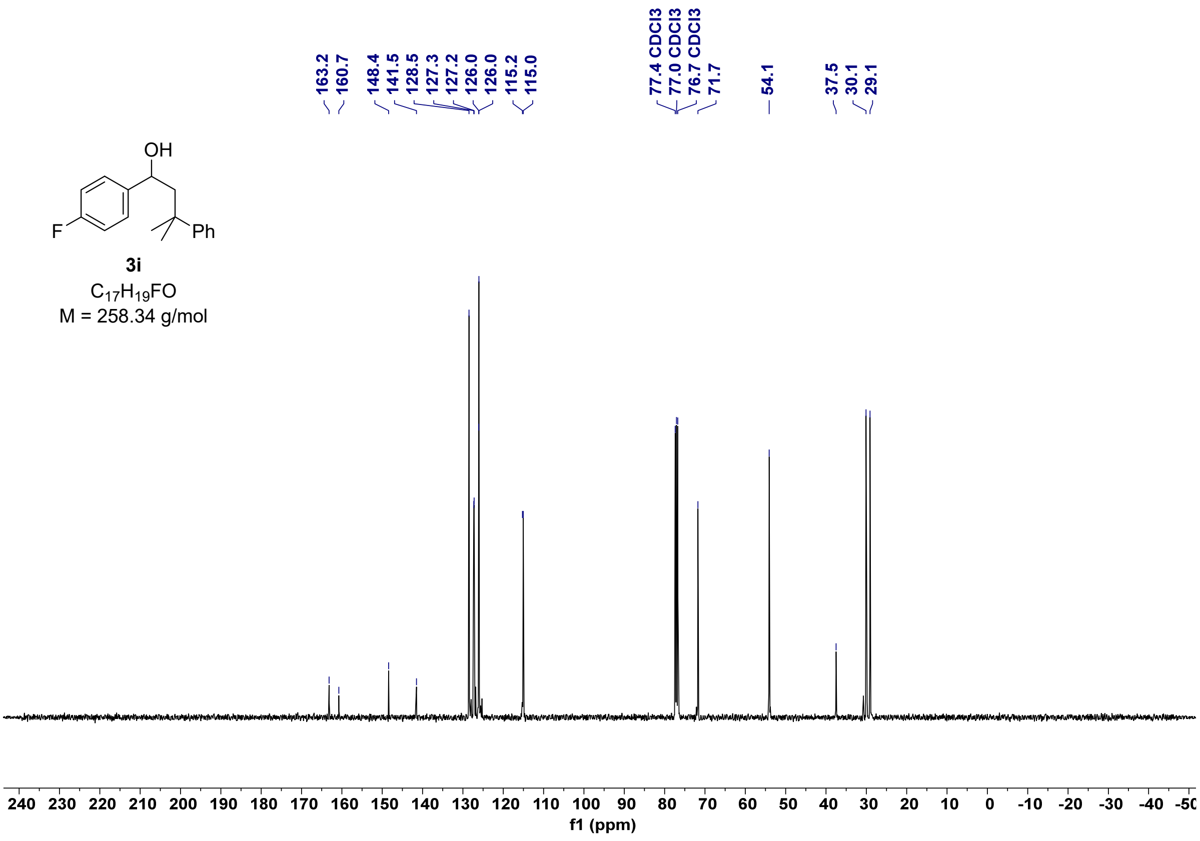

^{13}C{^{1}H} NMR spectrum (100 MHz, $CDCl_3$) of compound **3i**.

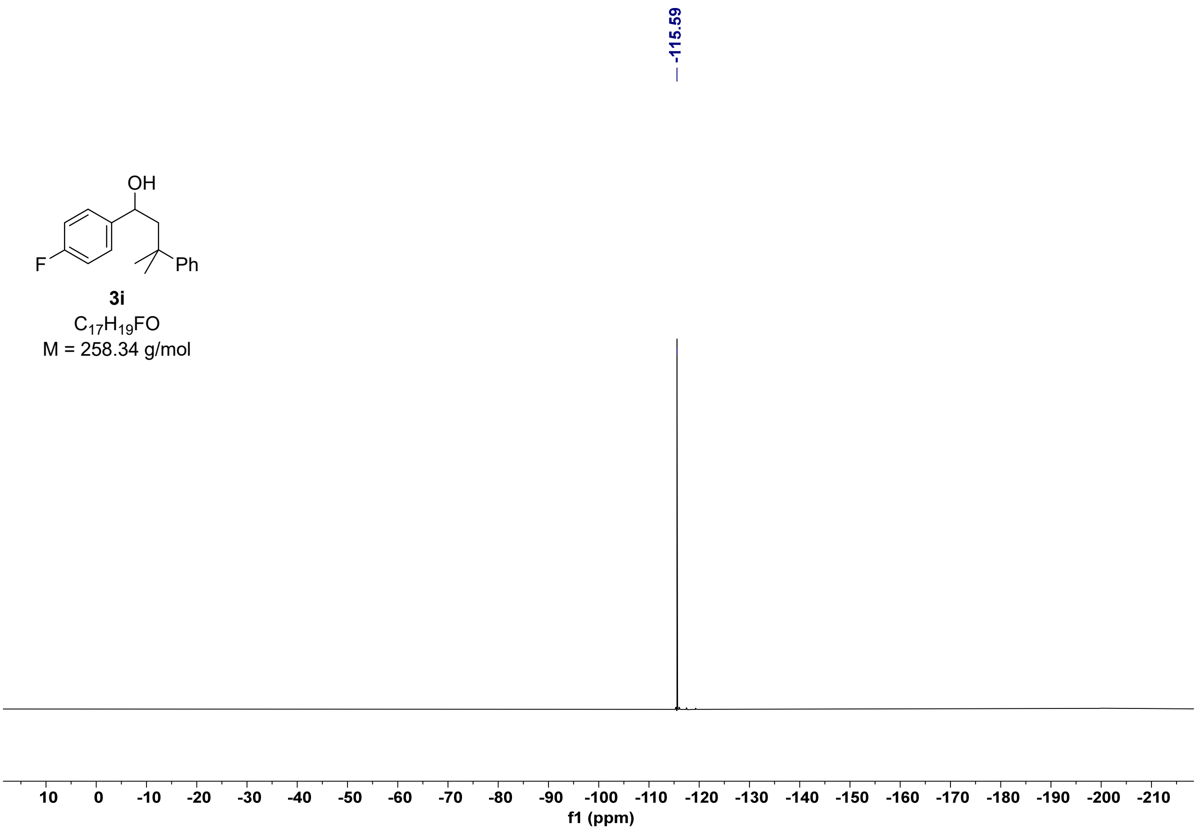

^{19}**F NMR** spectrum (400 MHz, CDCl$_3$) of compound **3i**.

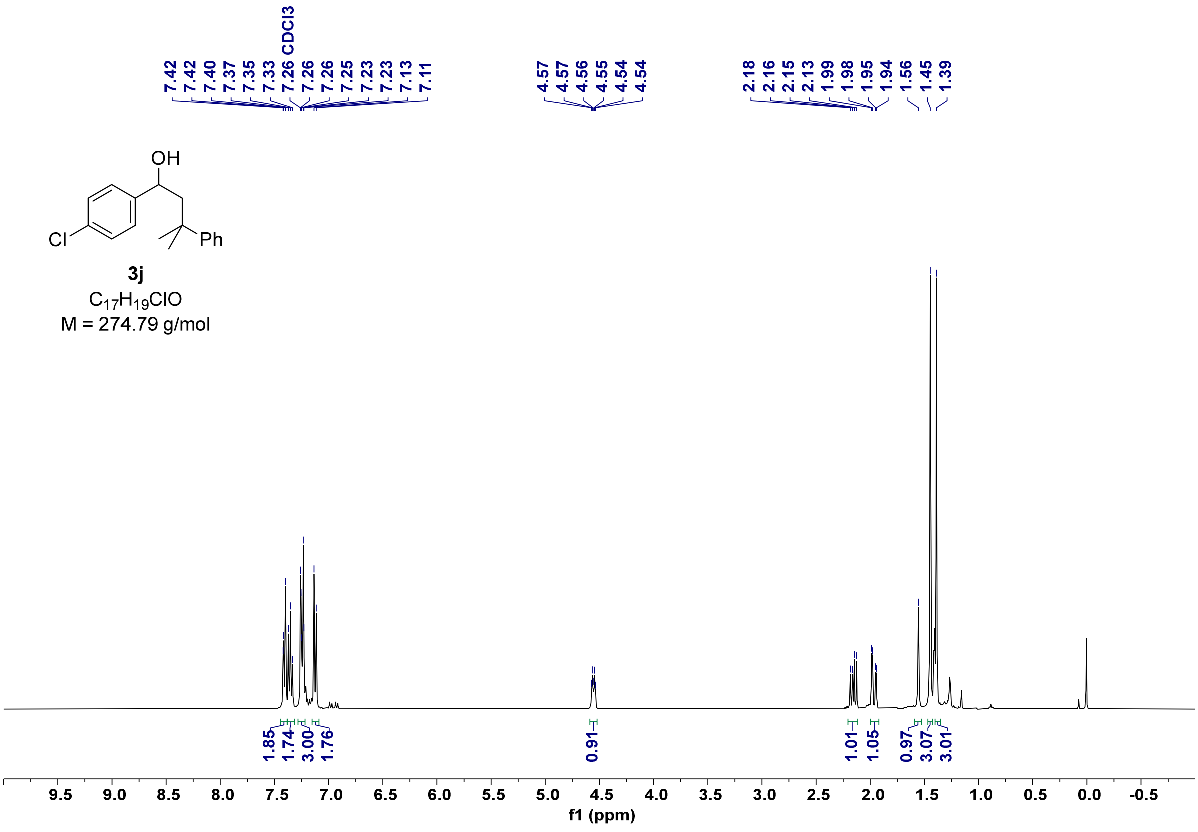

^{1}**H NMR** spectrum (400 MHz, CDCl$_3$) of compound **3j**.

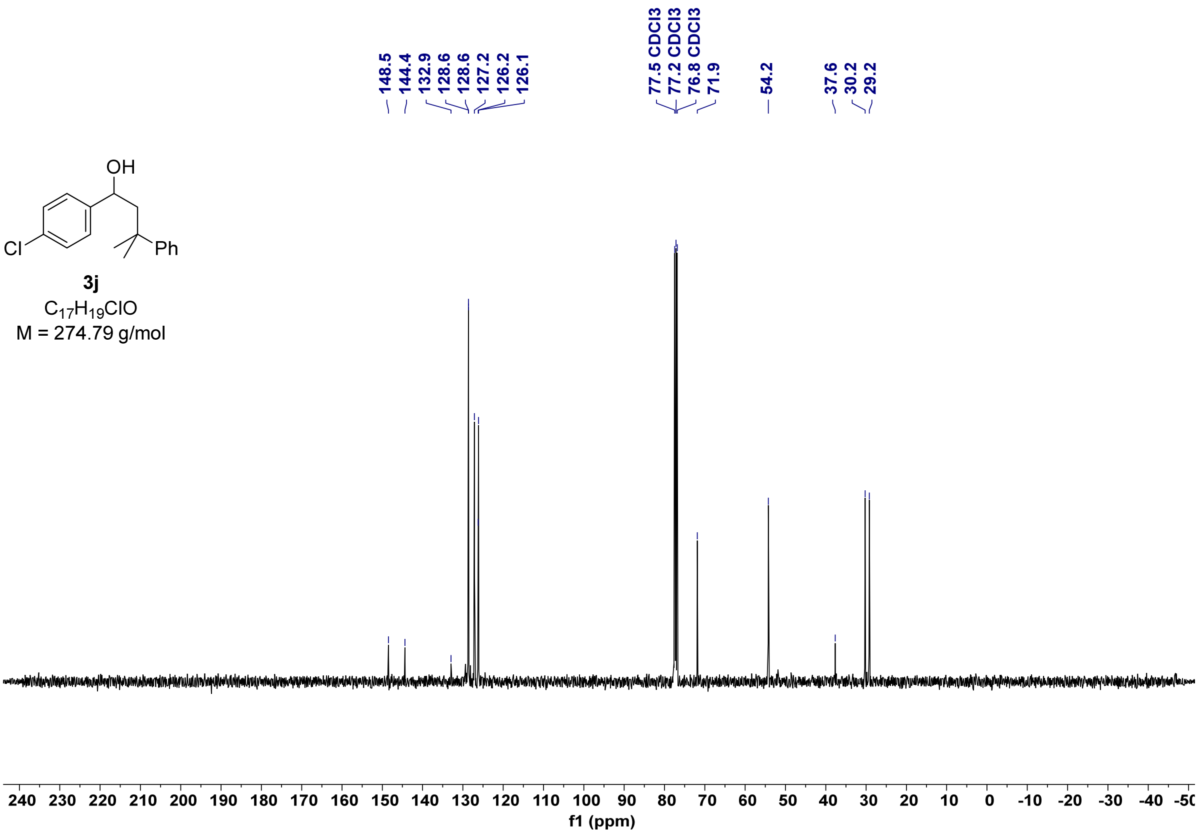

^{13}**C{^{1}H} NMR** spectrum (100 MHz, $CDCl_3$) of compound **3j**.

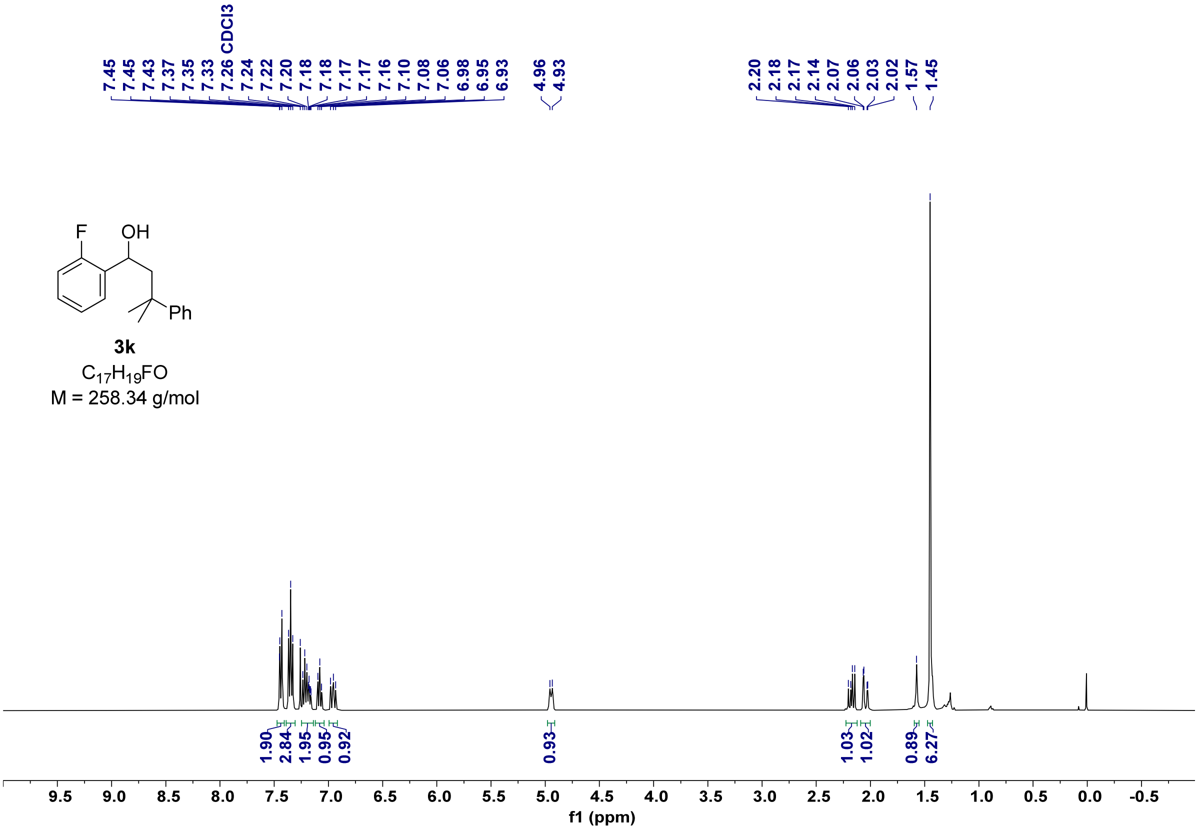

^{1}**H NMR** spectrum (400 MHz, $CDCl_3$) of compound **3k**.

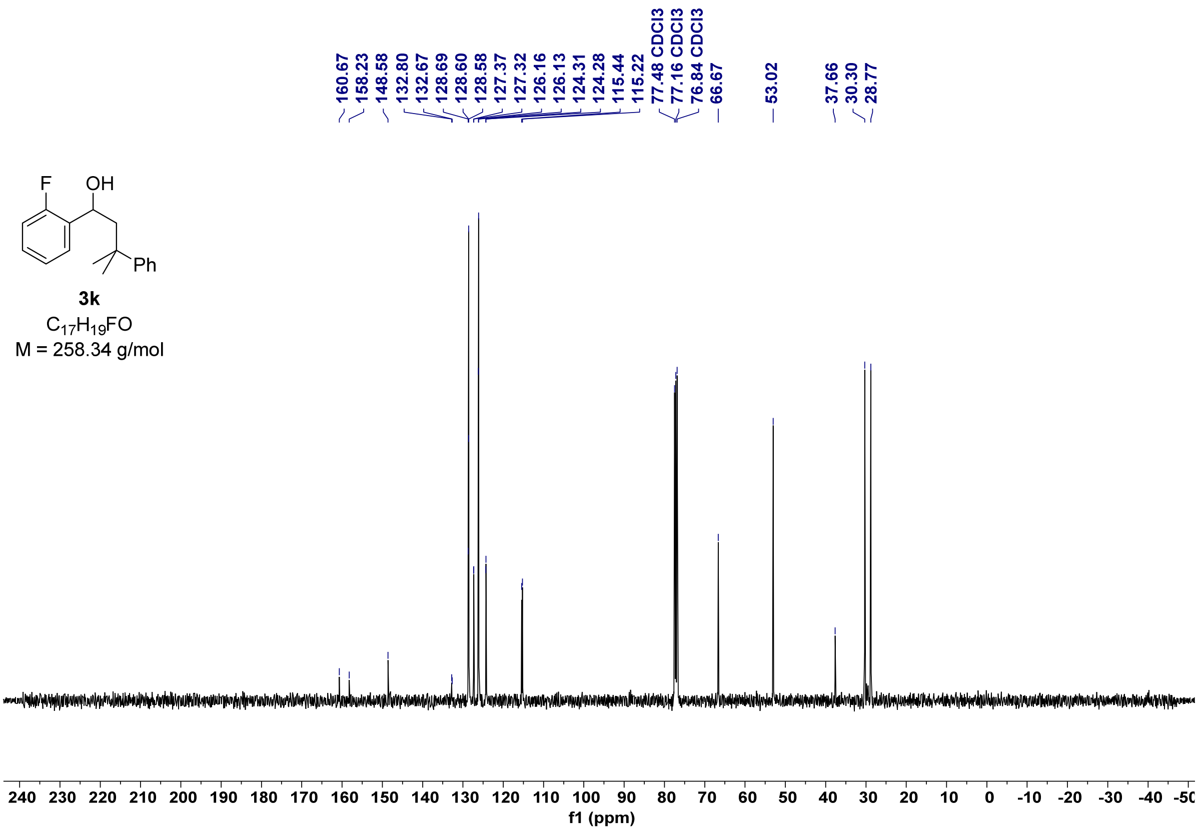

^{13}C{^{1}H} NMR spectrum (100 MHz, $CDCl_3$) of compound **3k**.

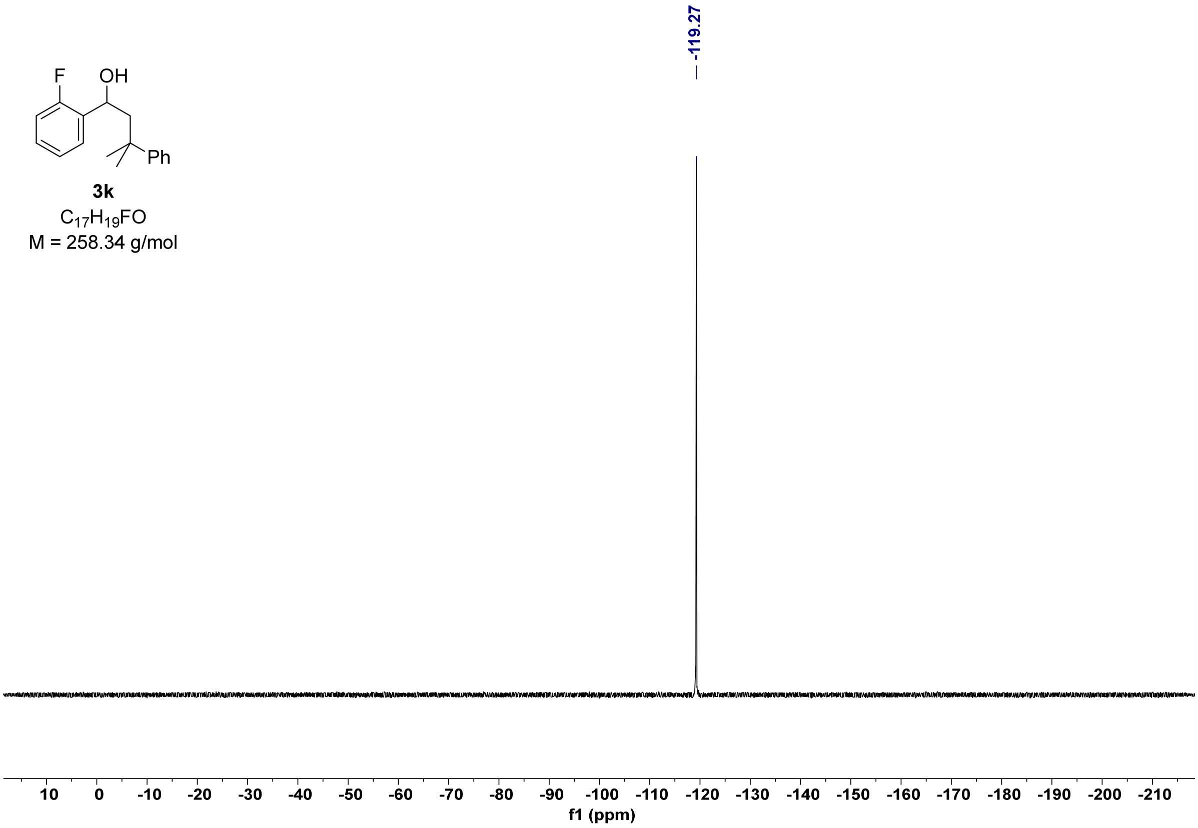

^{19}F NMR spectrum (400 MHz, $CDCl_3$) of compound **3k**.

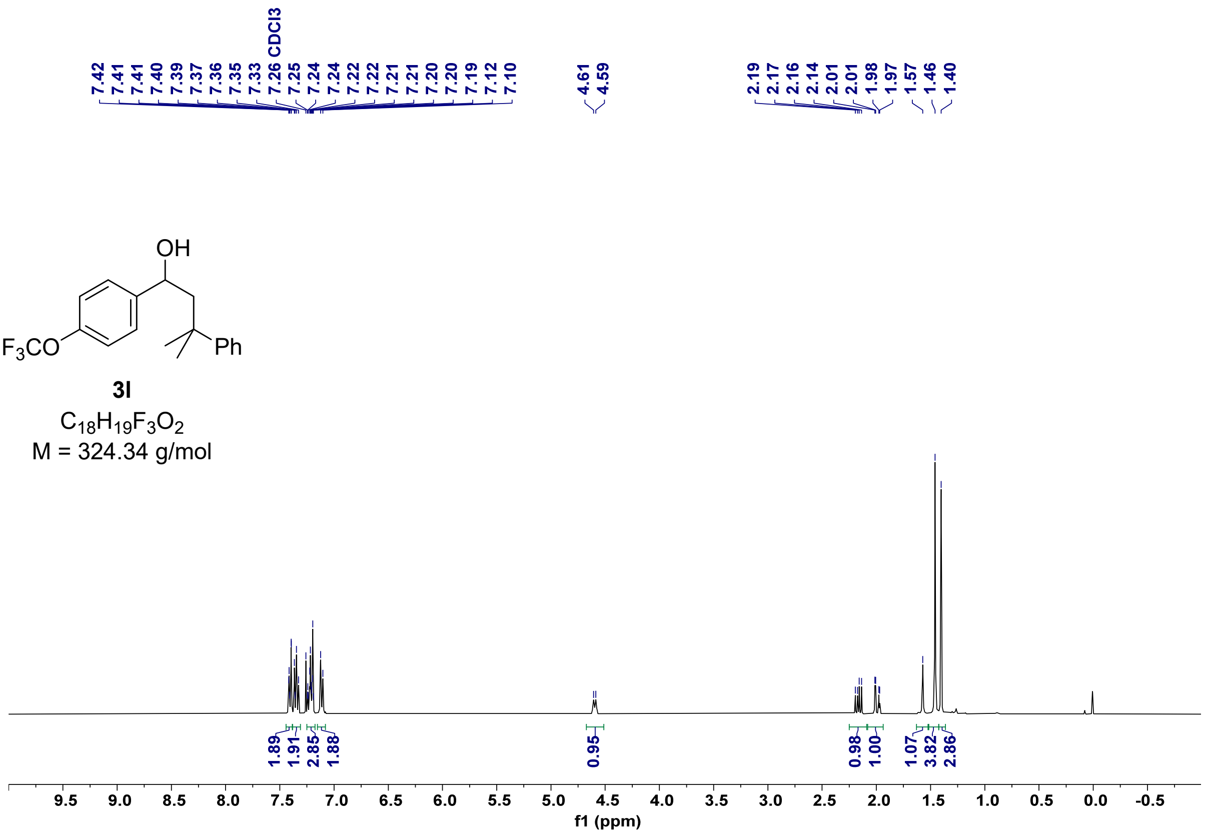

^{1}H NMR spectrum (400 MHz, $CDCl_3$) of compound **3l**.

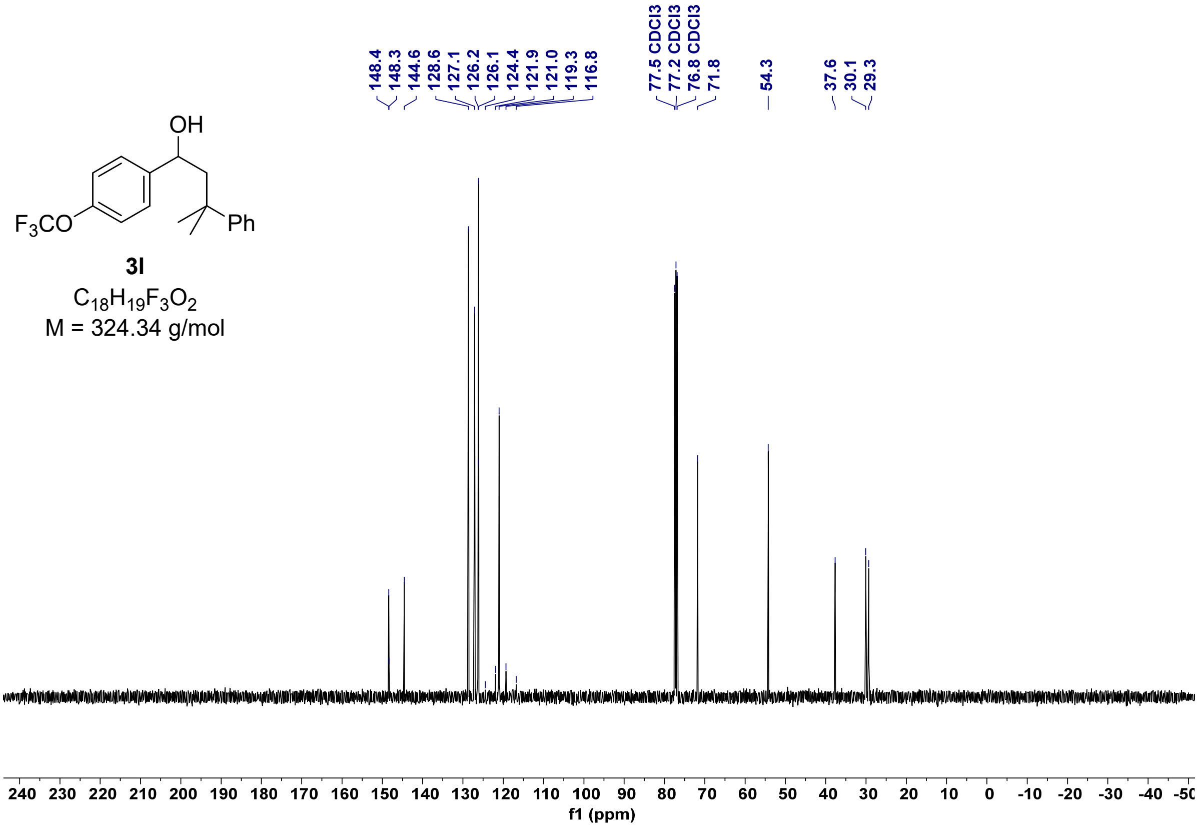

$^{13}C\{^1H\}$ NMR spectrum (100 MHz, $CDCl_3$) of compound **3l**.

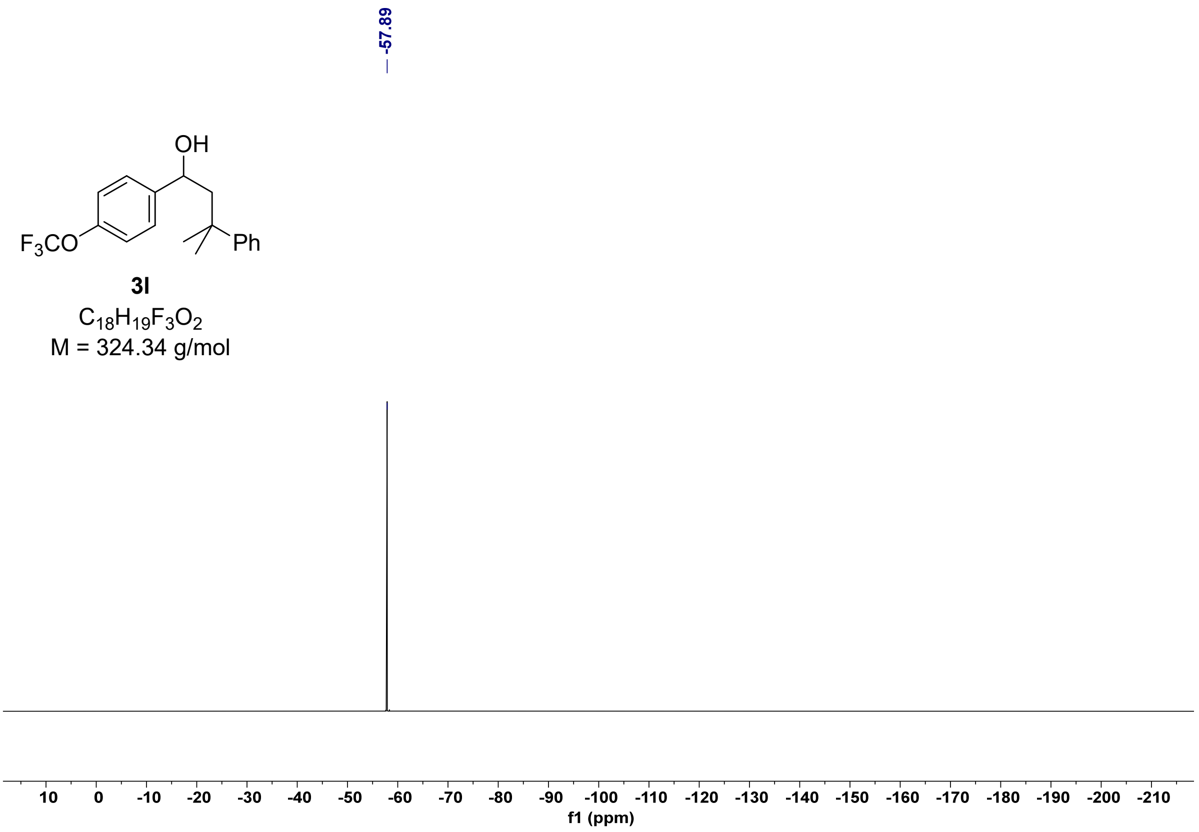

^{19}F NMR spectrum (400 MHz, $CDCl_3$) of compound **3l**.

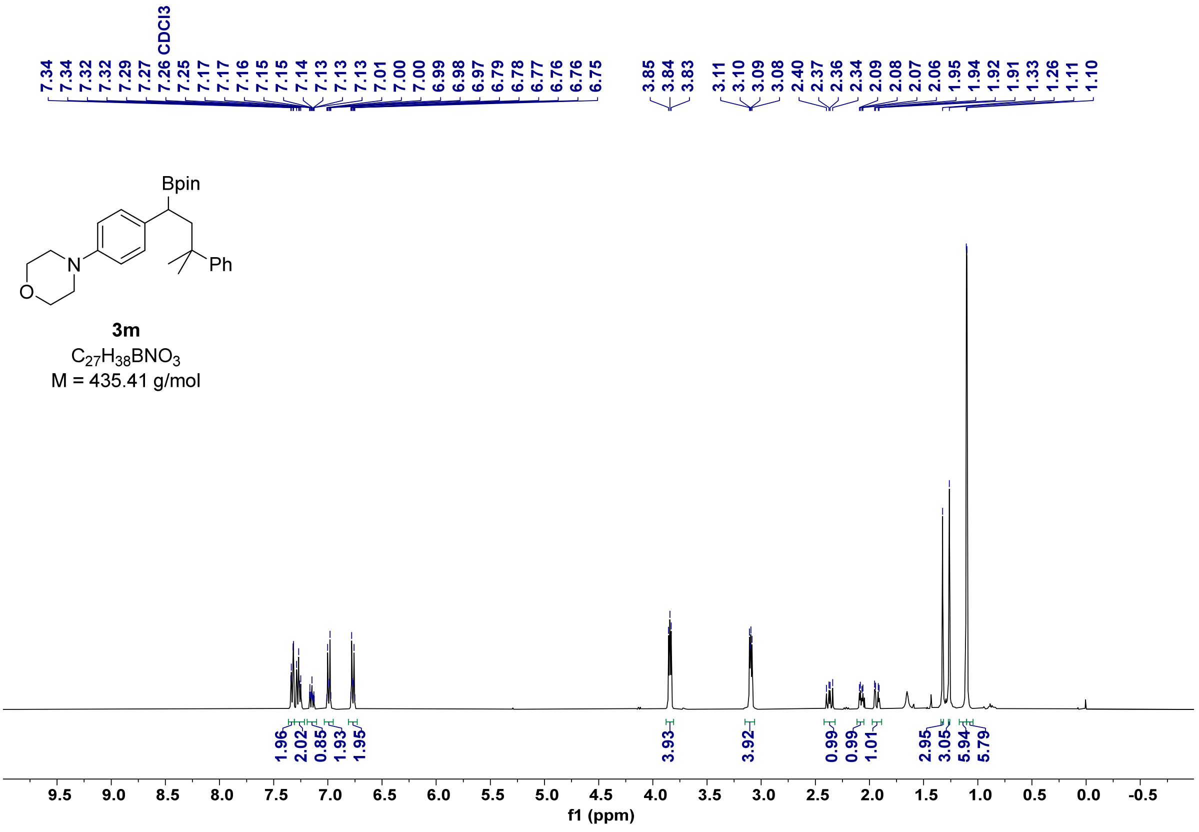

^{1}H NMR spectrum (400 MHz, $CDCl_3$) of compound **3m**.

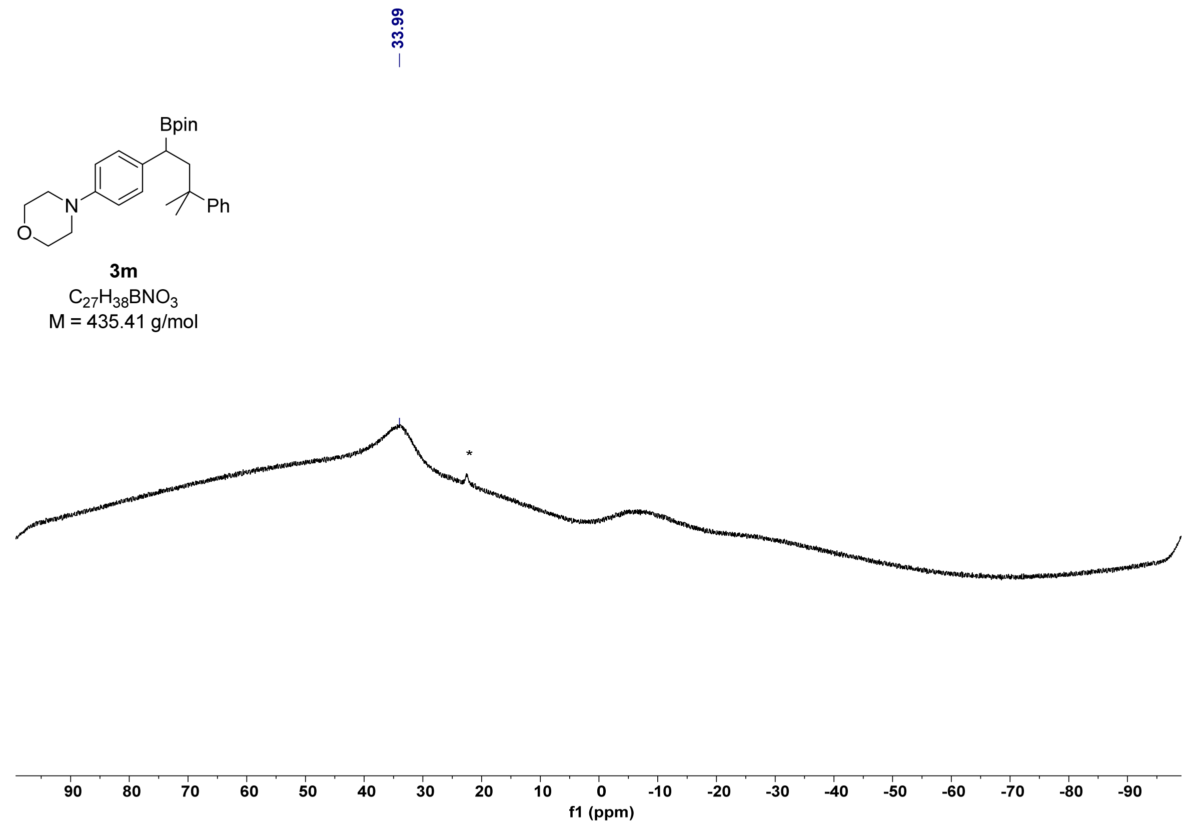

^{11}B NMR spectrum (400 MHz, $CDCl_3$) of compound **3m**. *: Impurity due to decomposition of product after isolation

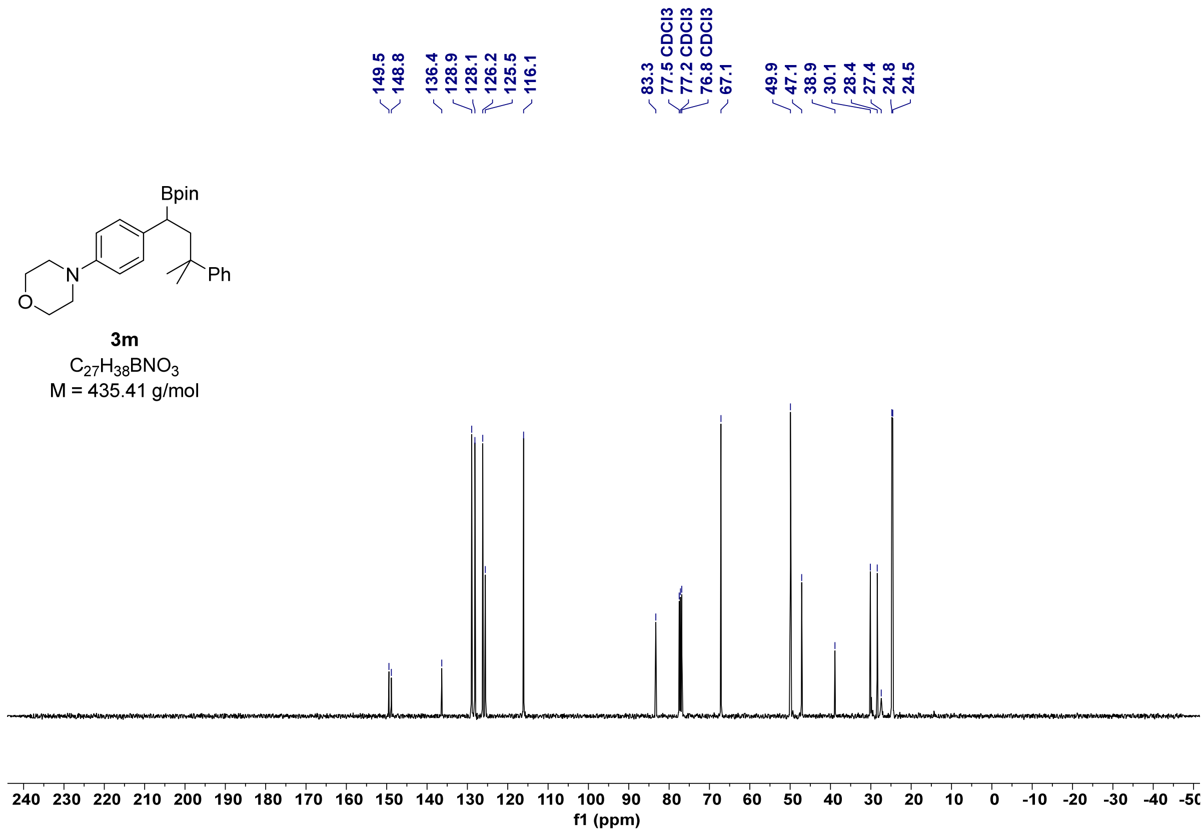

^{13}C{^{1}H} NMR spectrum (100 MHz, $CDCl_3$) of compound **3m**.

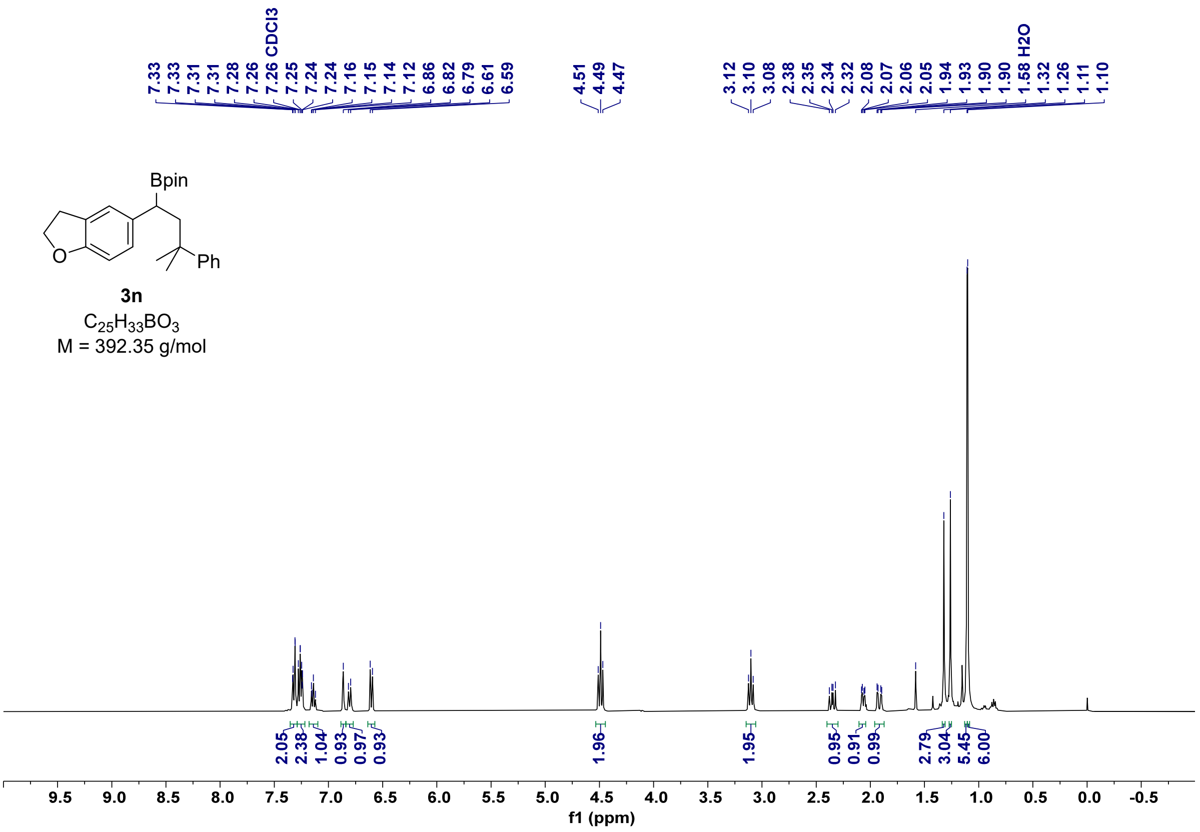

^{1}H NMR spectrum (400 MHz, $CDCl_3$) of compound **3n**.

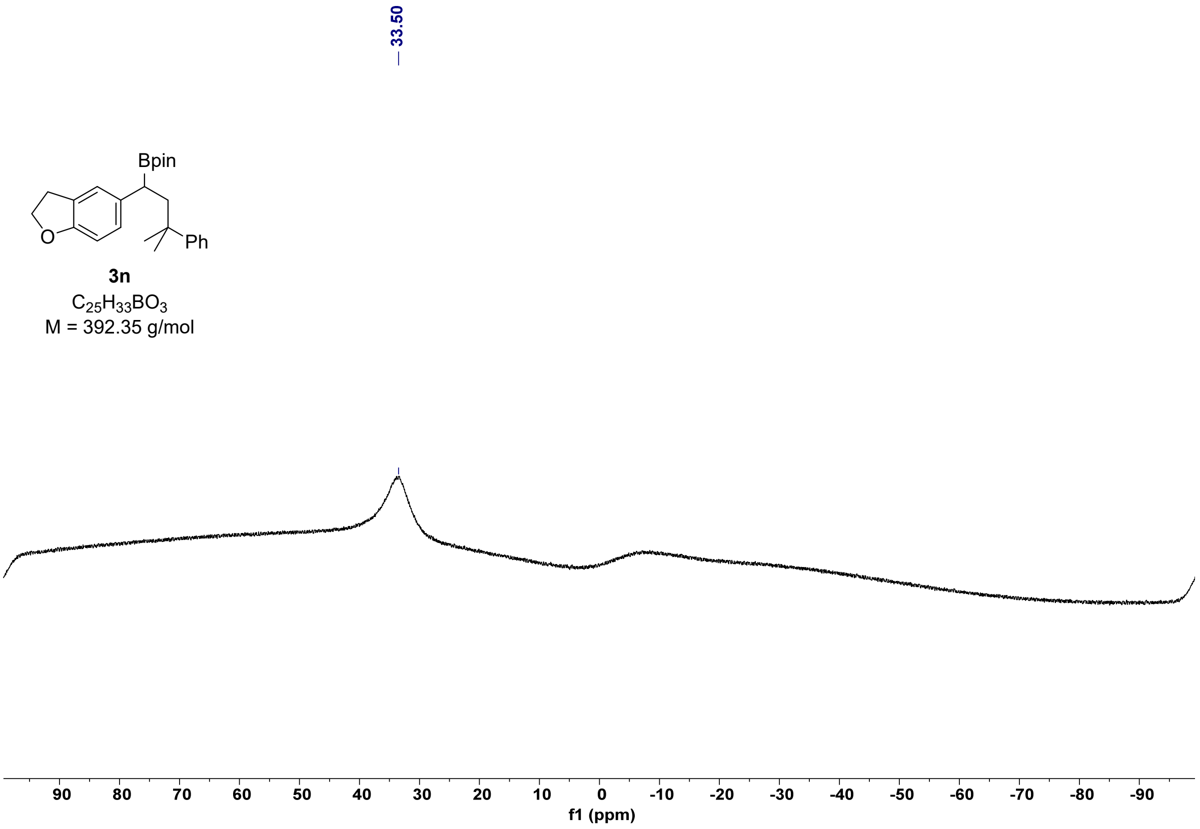

^{11}B NMR spectrum (400 MHz, $CDCl_3$) of compound **3n**.

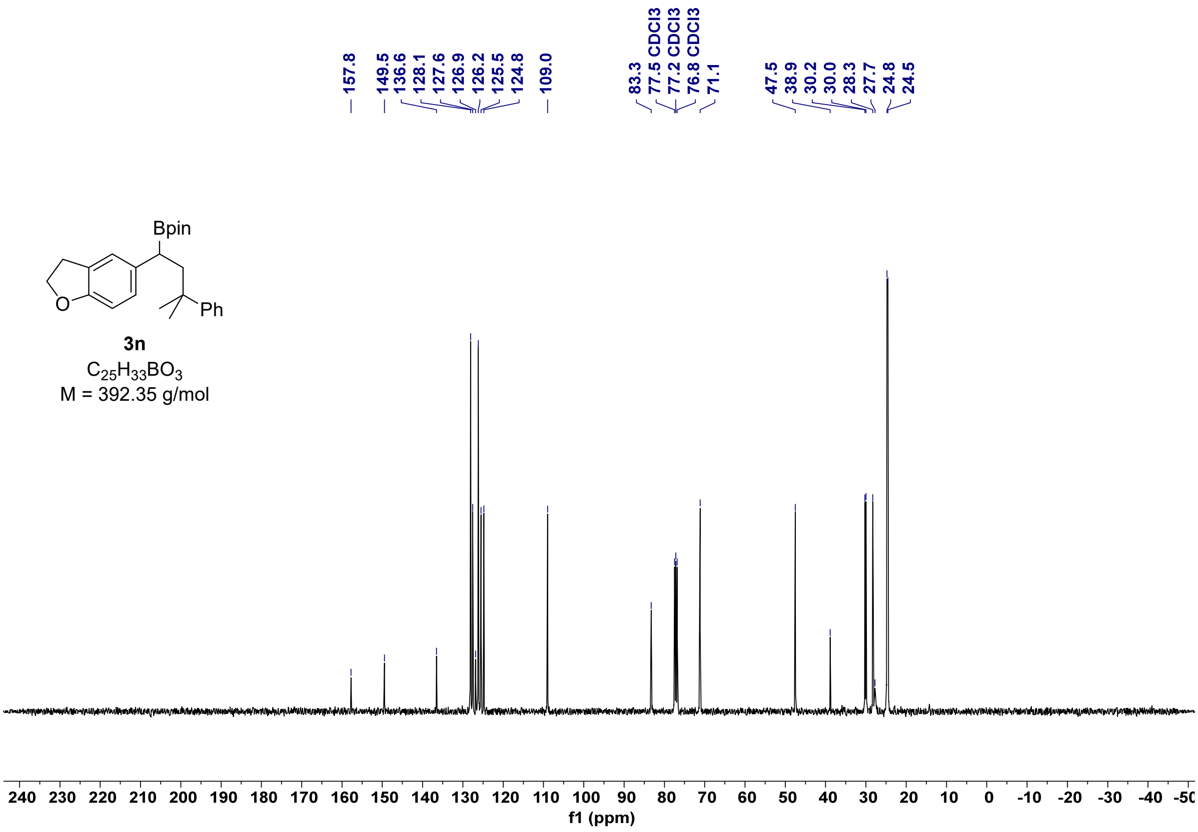

^{13}**C{^{1}H} NMR** spectrum (100 MHz, $CDCl_3$) of compound **3n**.

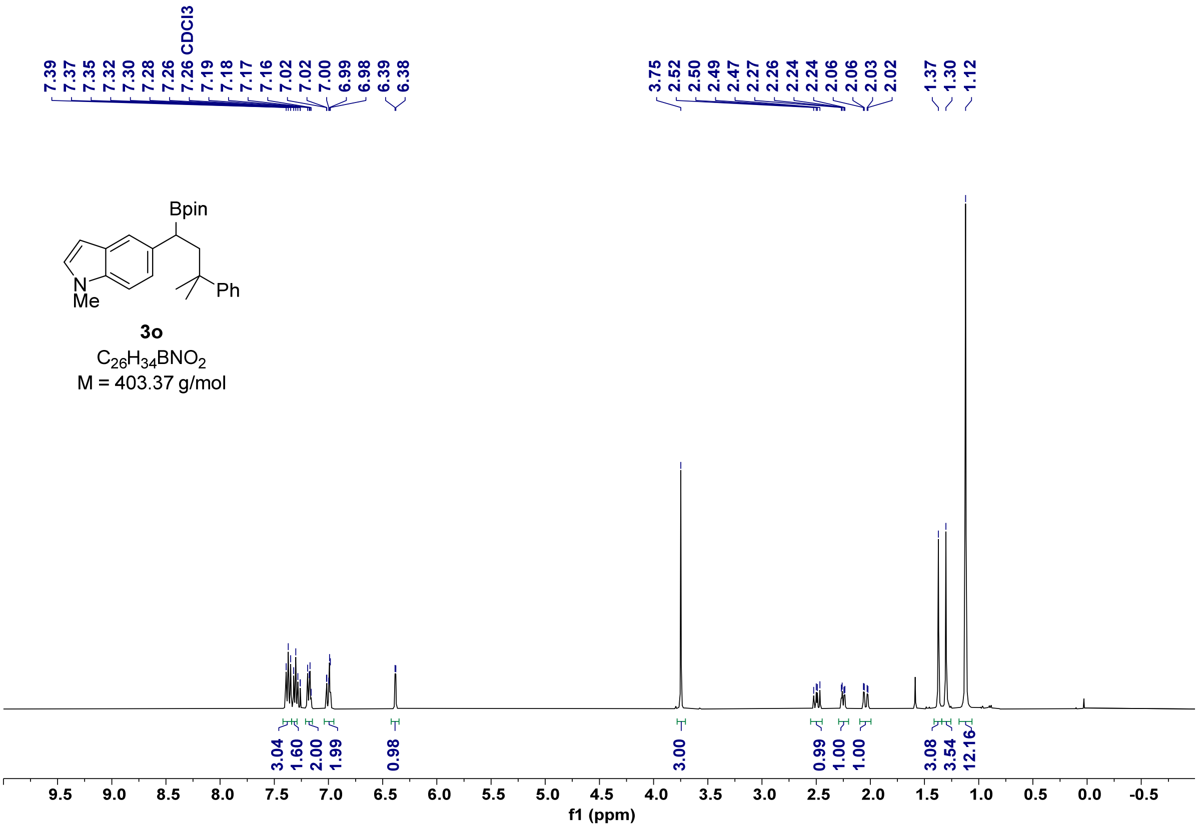

^{1}**H NMR** spectrum (400 MHz, $CDCl_3$) of compound **3o**.

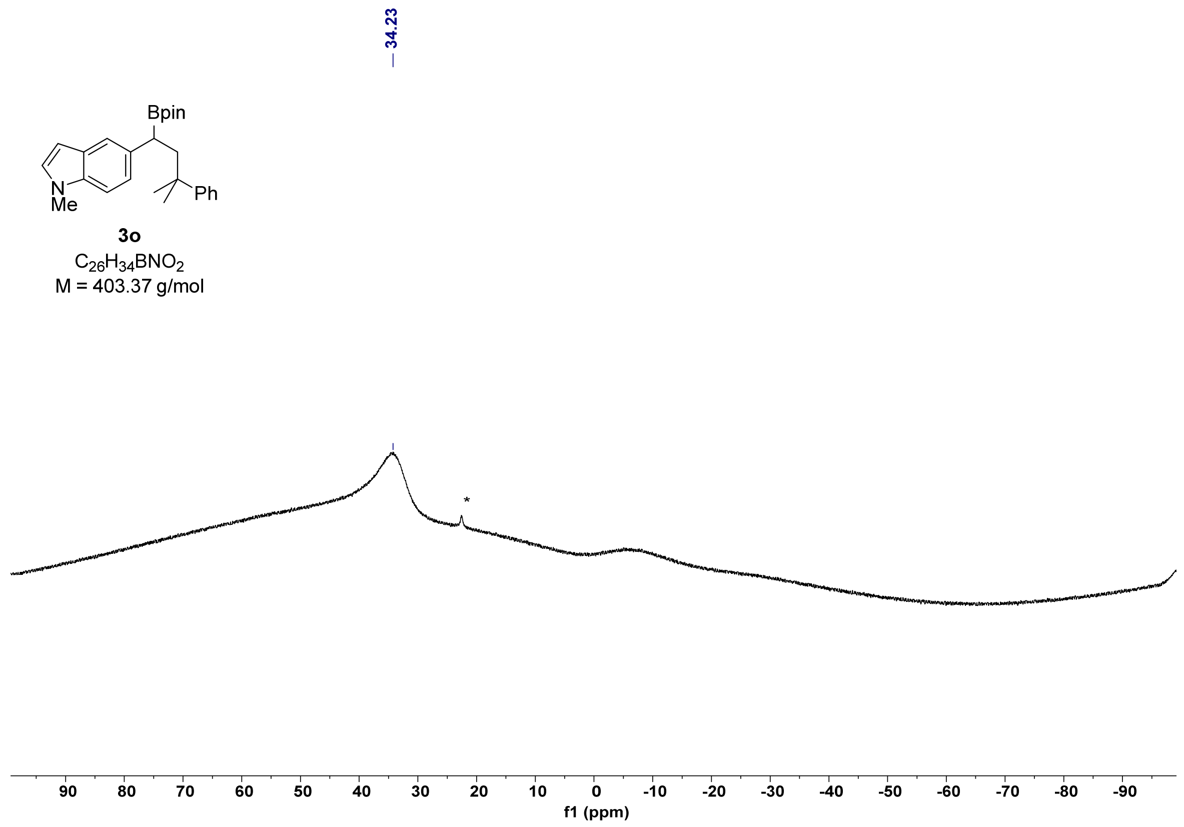

[11]**B NMR** spectrum (400 MHz, $CDCl_3$) of compound **3o**.*: Impurity due to decomposition of product after isolation

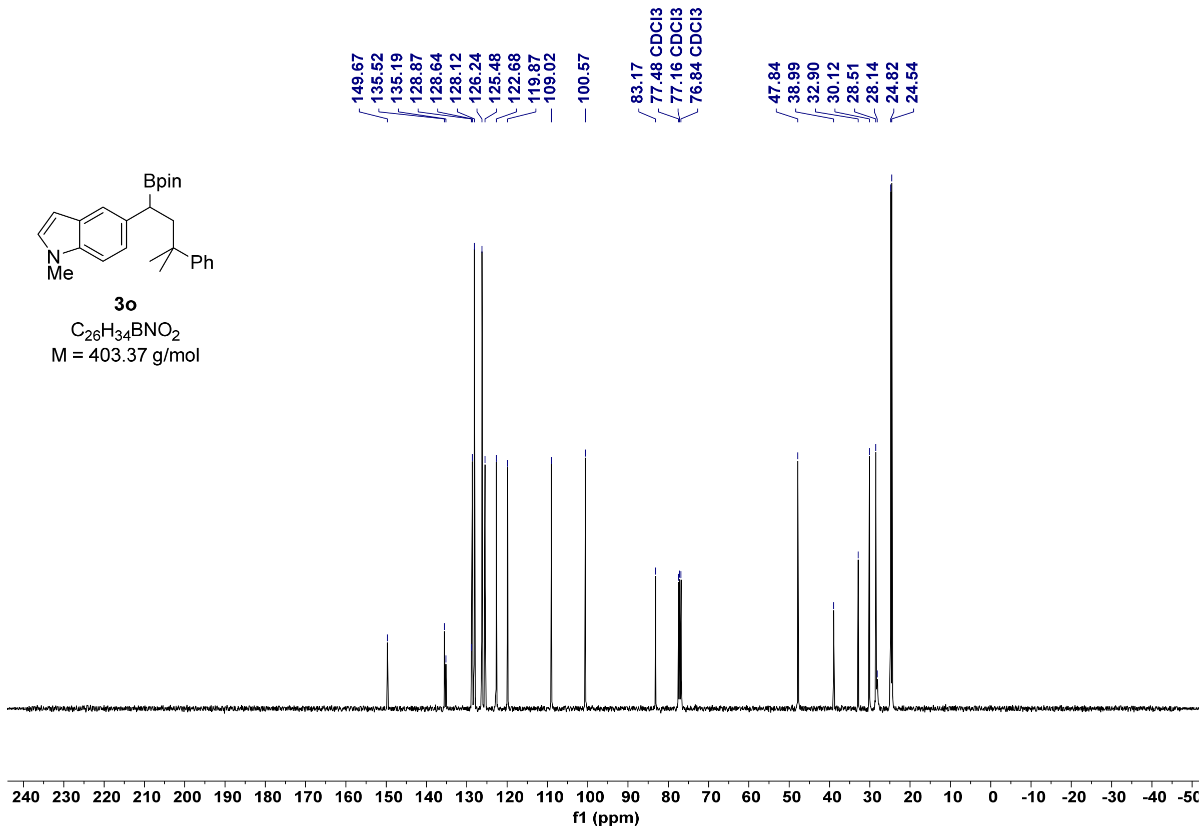

[13]**C{^{1}H} NMR** spectrum (100 MHz, $CDCl_3$) of compound **3o**.

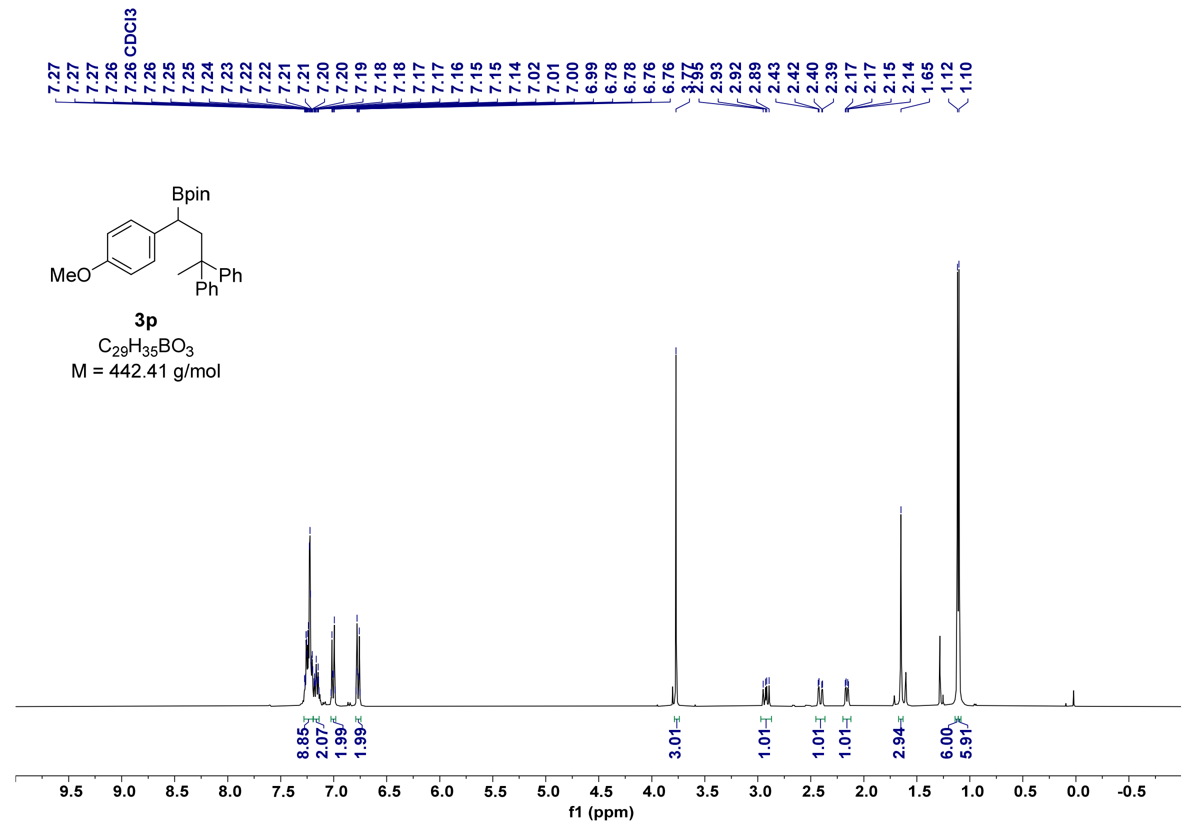

^{1}H NMR spectrum (400 MHz, $CDCl_3$) of compound **3p**.

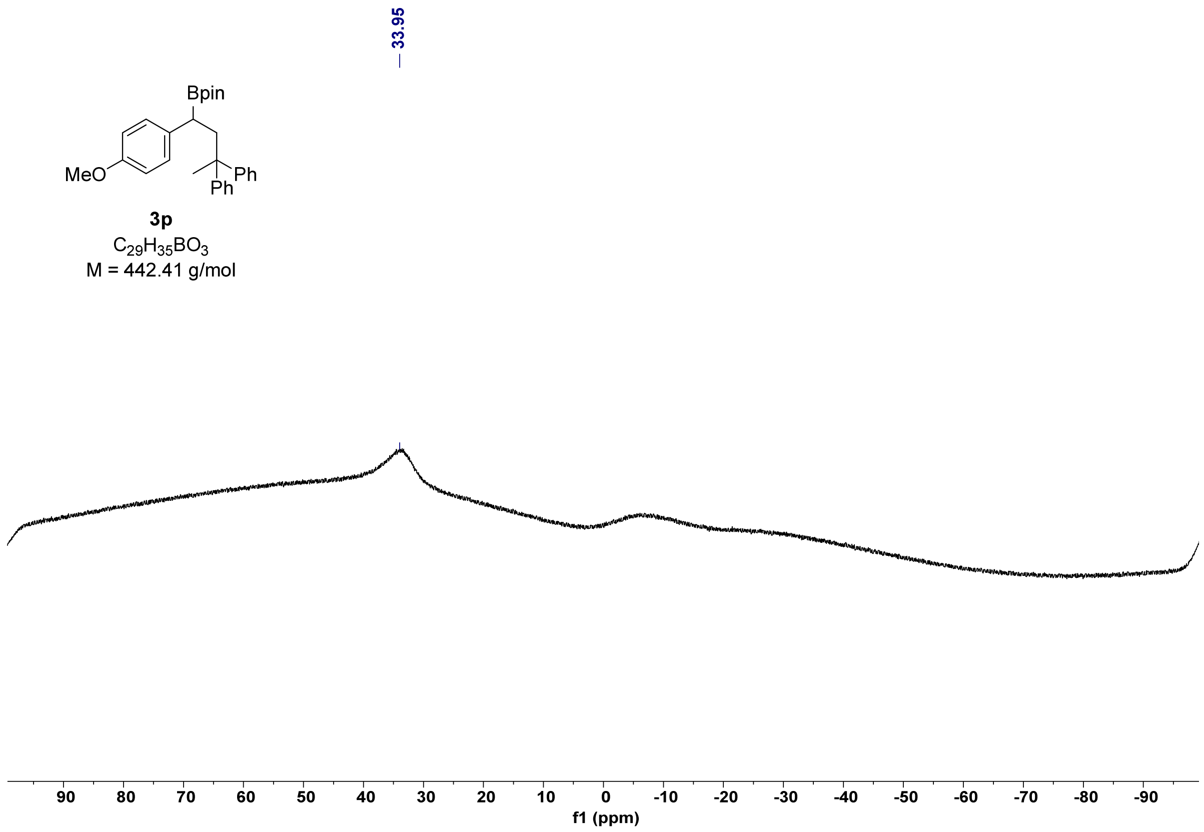

^{11}B NMR spectrum (400 MHz, $CDCl_3$) of compound **3p**.

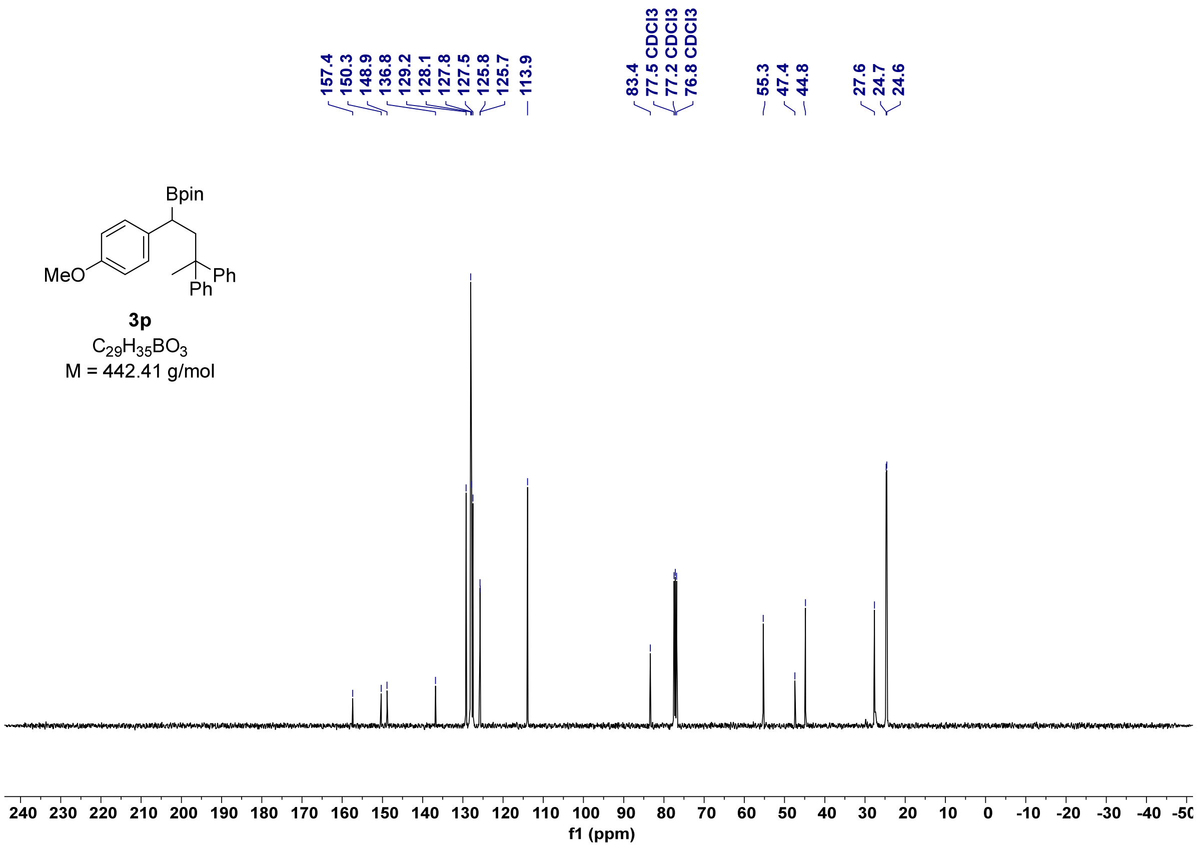

^{13}C{^{1}H} **NMR** spectrum (100 MHz, $CDCl_3$) of compound **3p**.

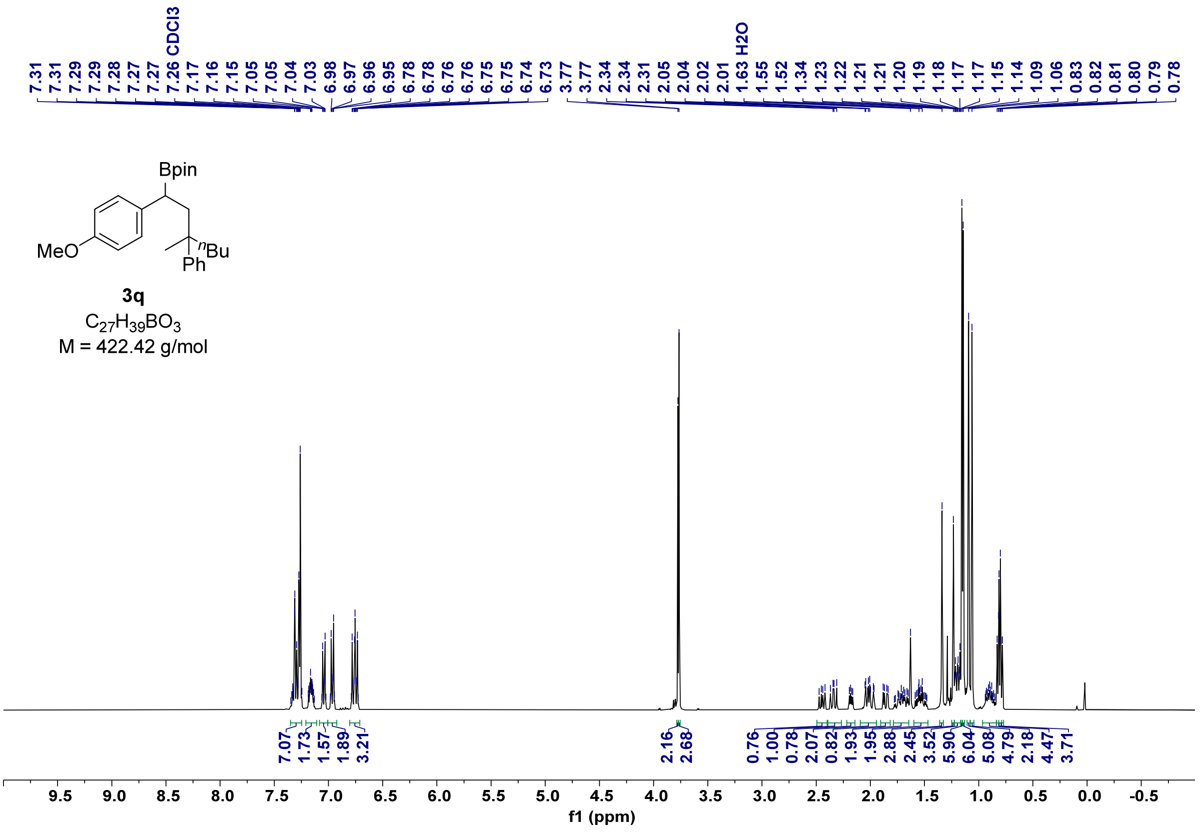

^{1}H **NMR** spectrum (400 MHz, $CDCl_3$) of compound **3q**.

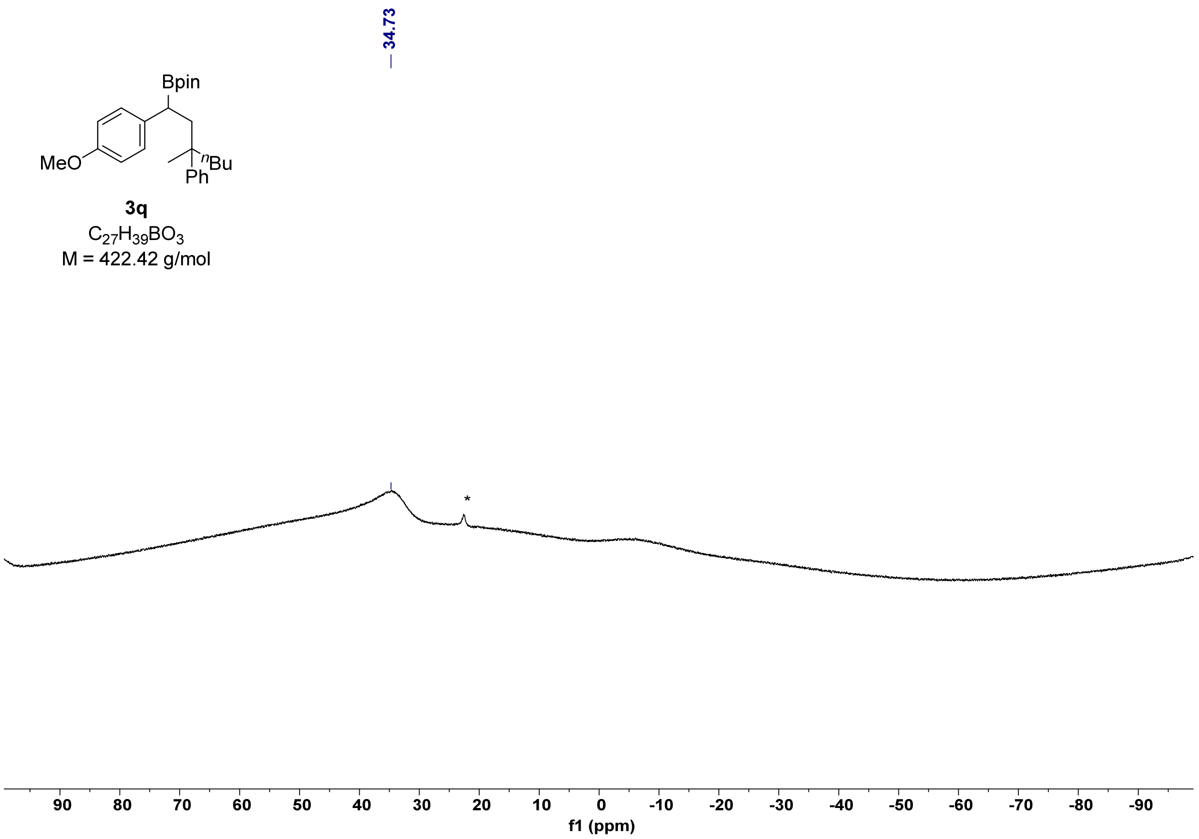

^{11}B NMR spectrum (400 MHz, $CDCl_3$) of compound **3q**. *: Impurity due to decomposition of product after isolation

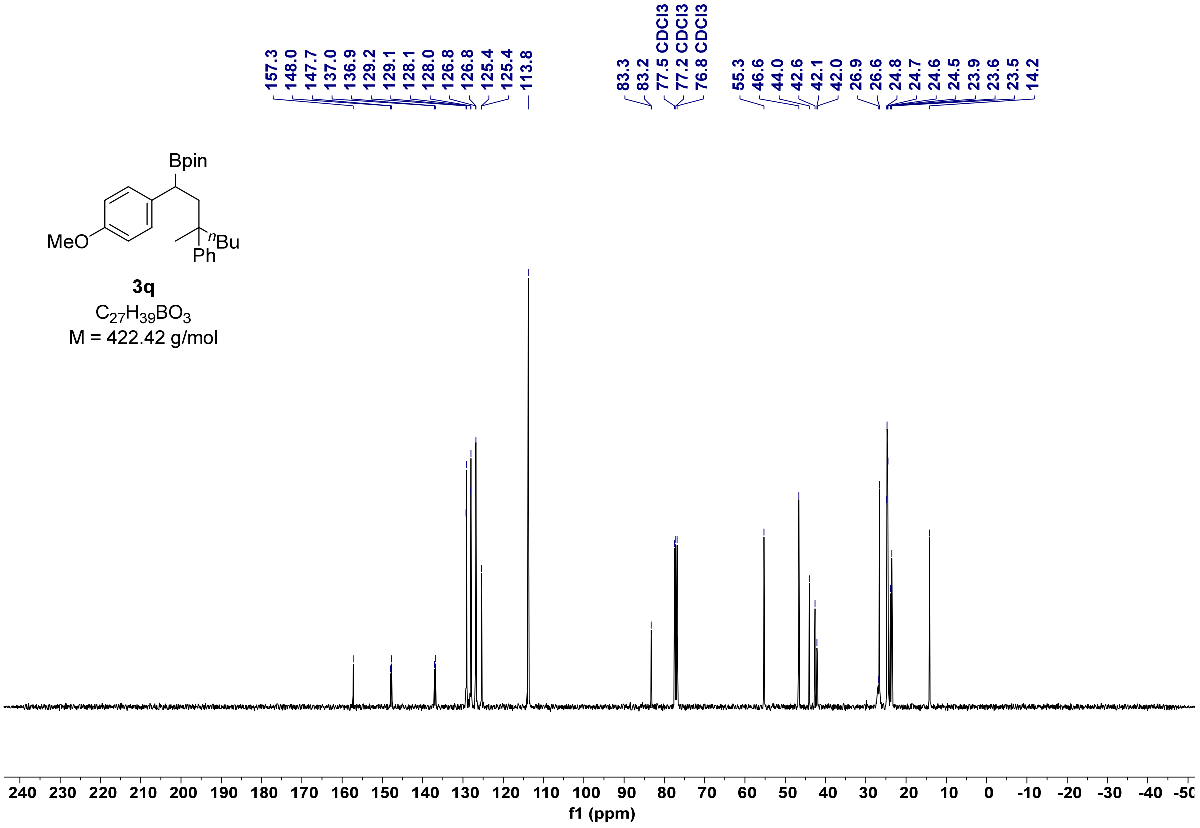

^{13}C{^{1}H} NMR spectrum (100 MHz, $CDCl_3$) of compound **3q**.

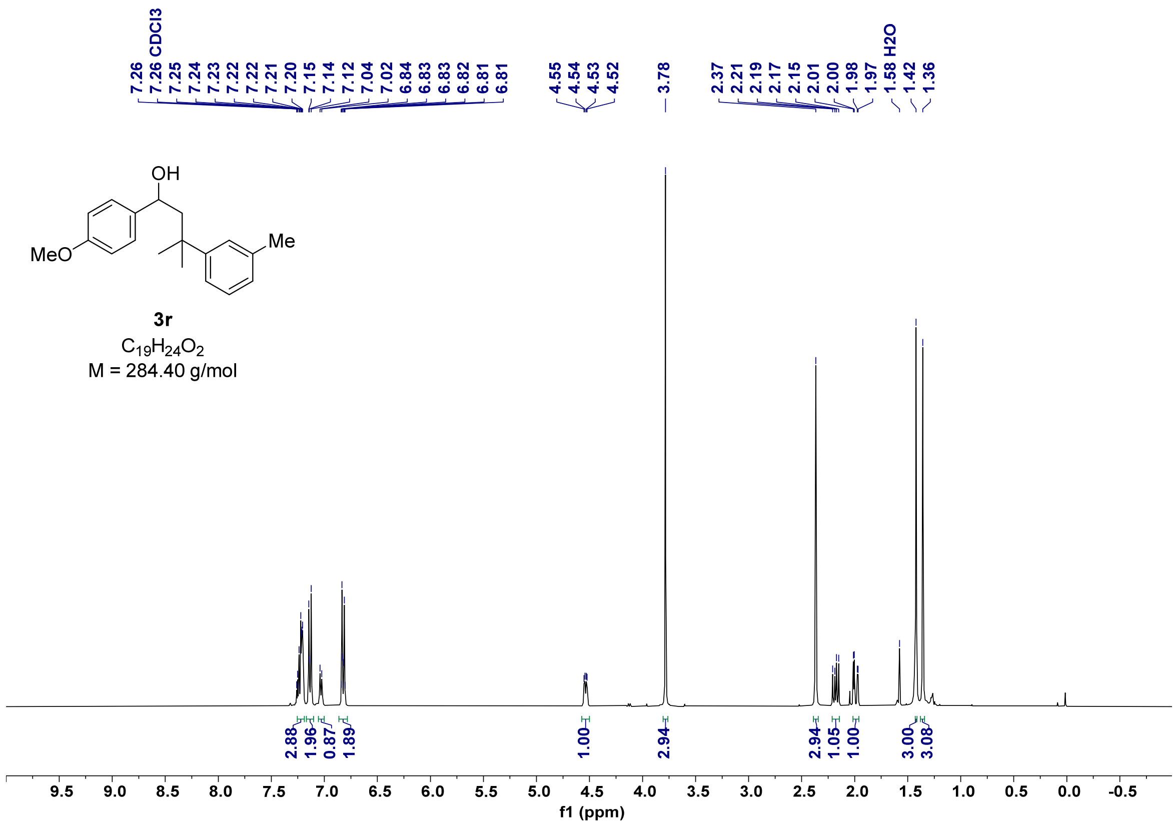

¹H NMR spectrum (400 MHz, $CDCl_3$) of compound **3r**.

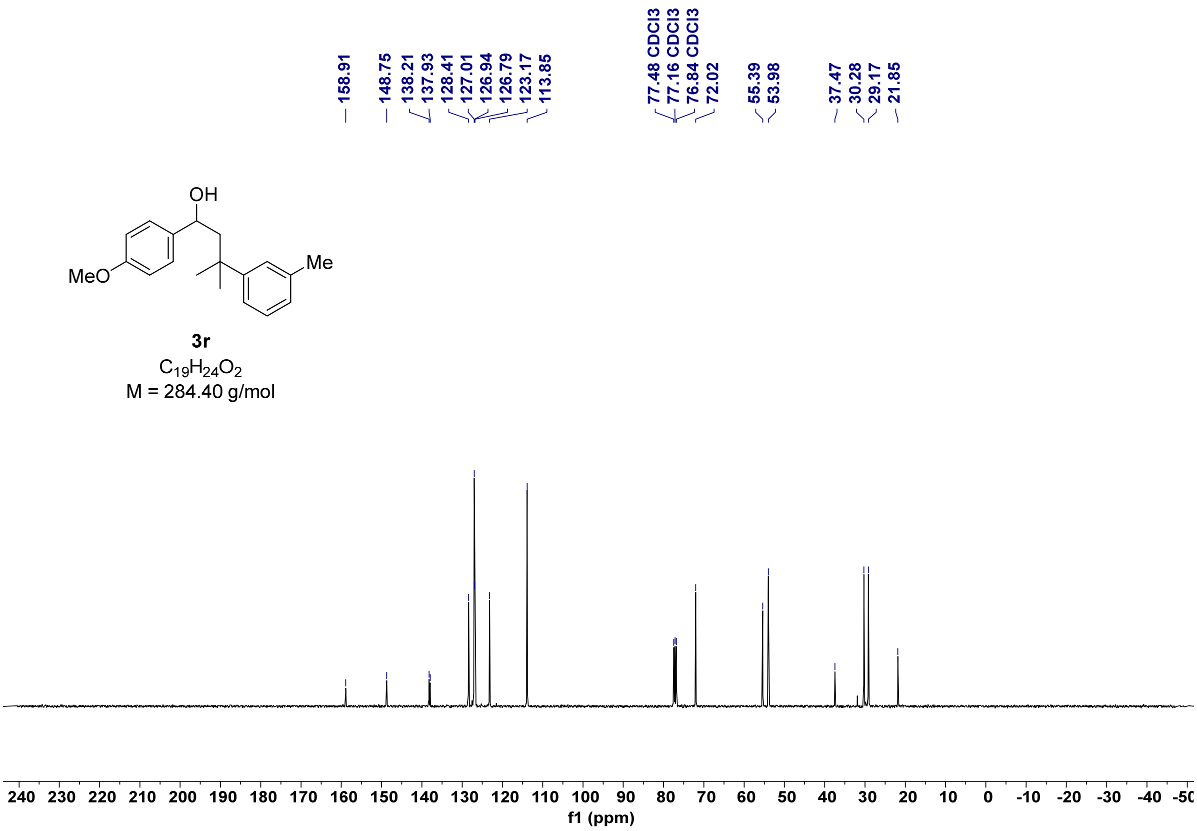

¹³C{¹H} NMR spectrum (100 MHz, $CDCl_3$) of compound **3r**.

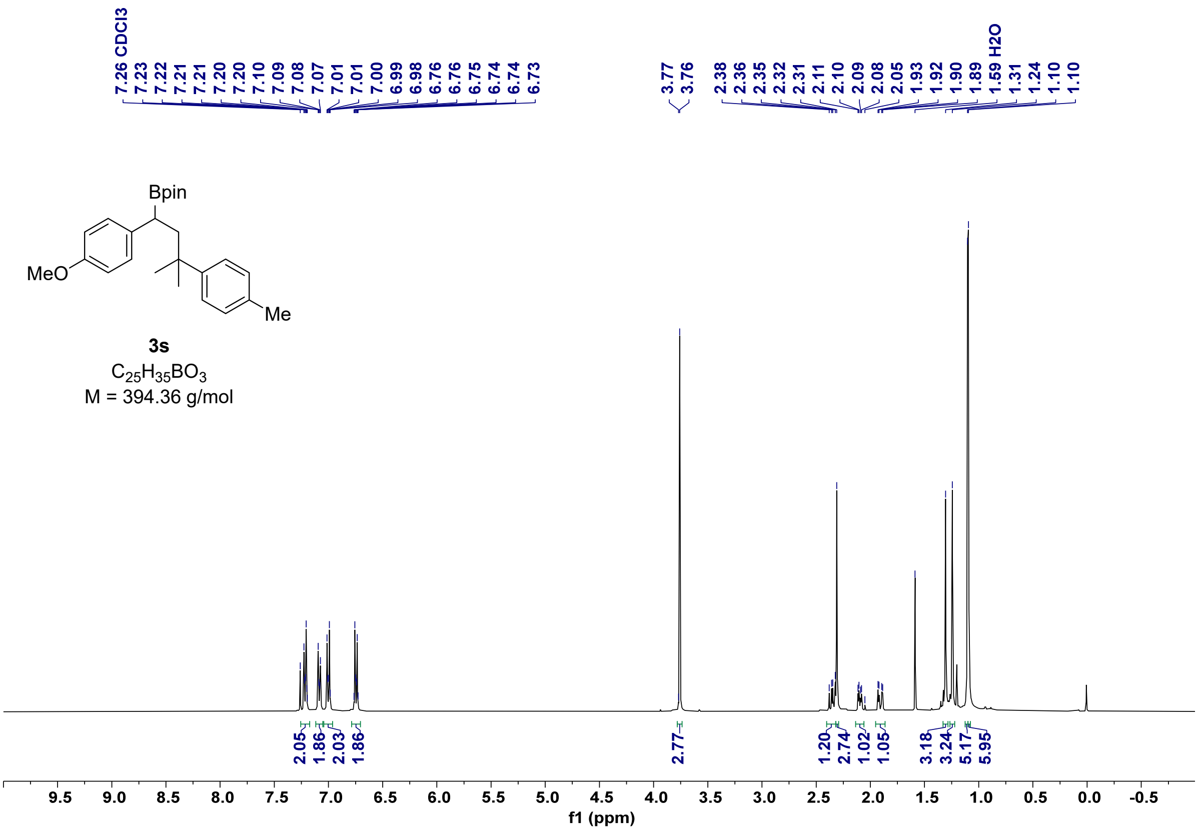

^{1}H NMR spectrum (400 MHz, $CDCl_3$) of compound **3s**.

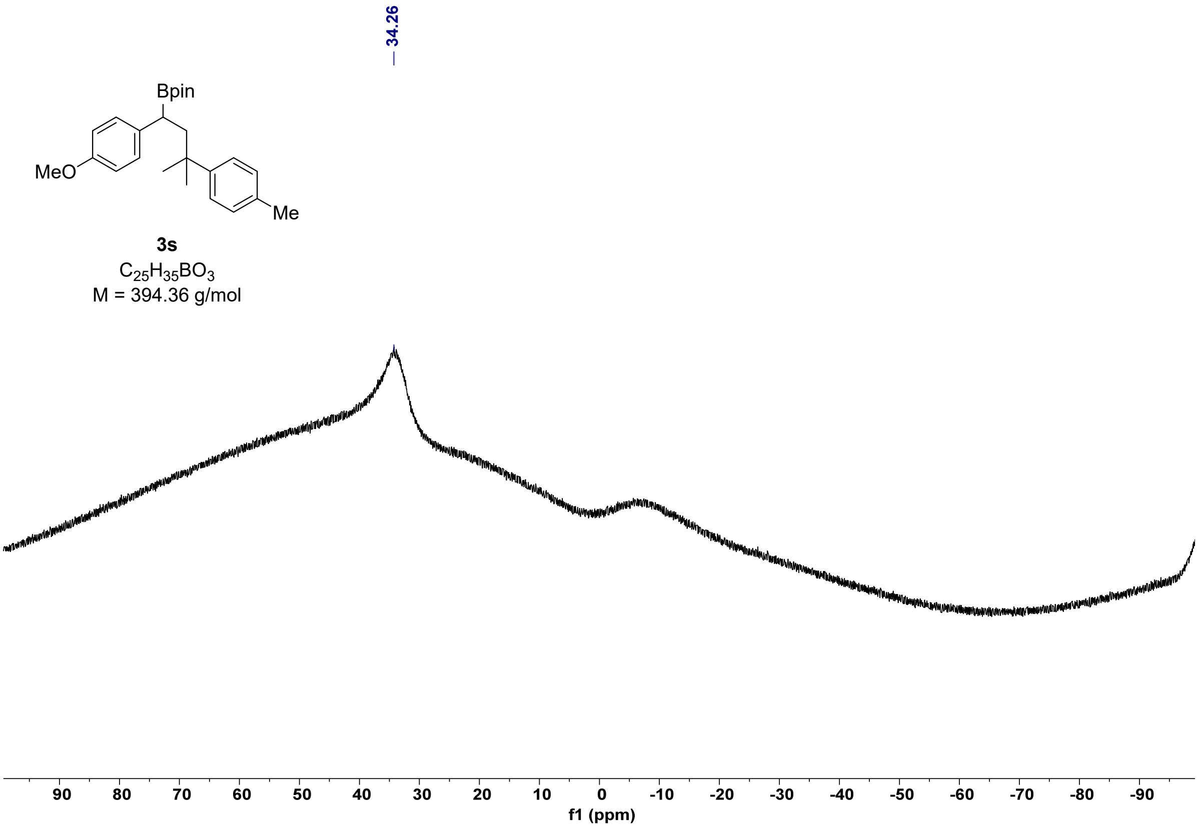

^{11}B NMR spectrum (400 MHz, $CDCl_3$) of compound **3s**.

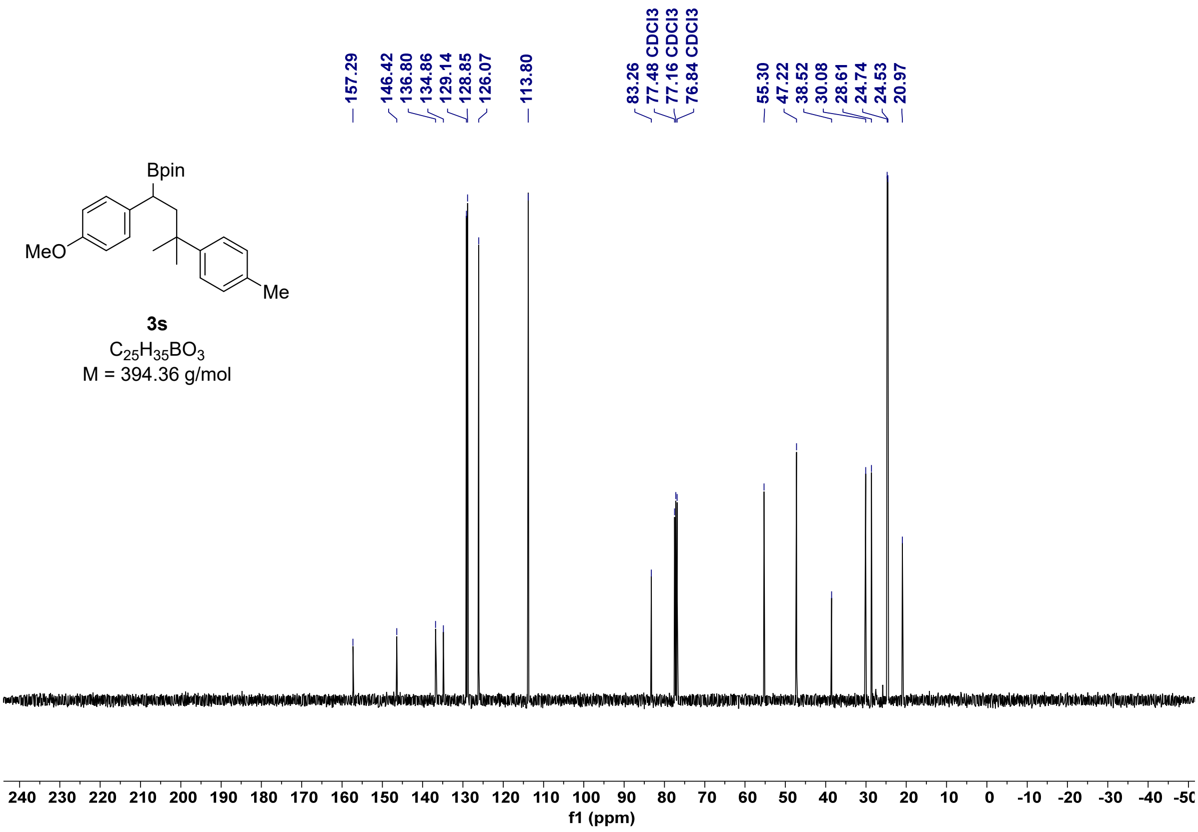

$^{13}C\{^1H\}$ NMR spectrum (100 MHz, $CDCl_3$) of compound **3s**.

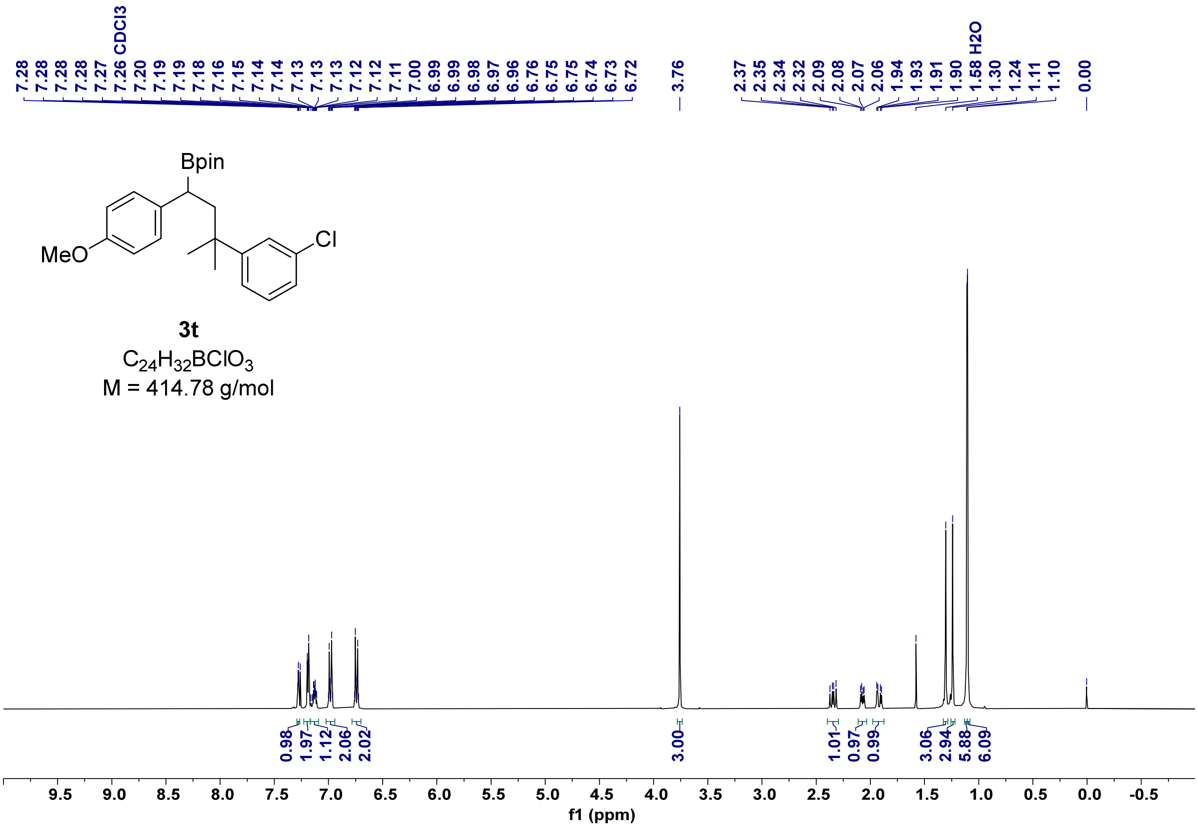

^1H NMR spectrum (400 MHz, $CDCl_3$) of compound **3t**.

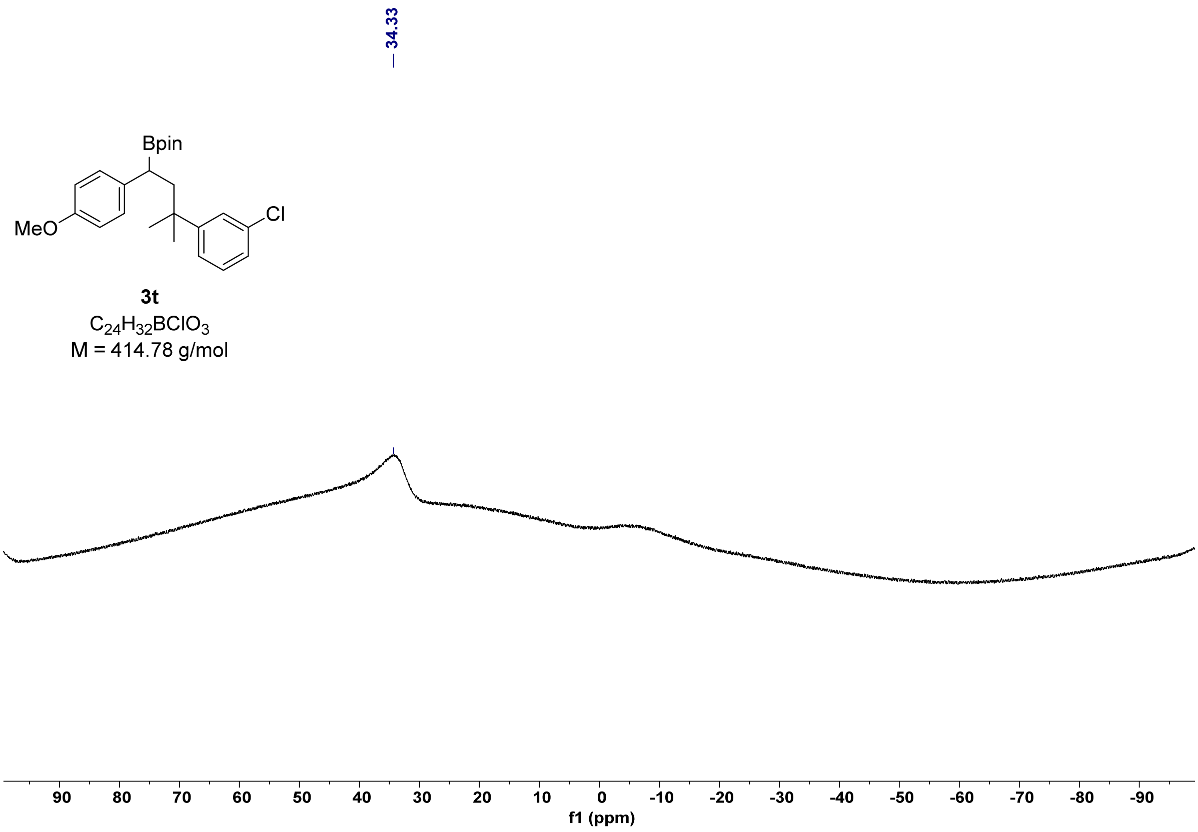

^{11}B NMR spectrum (400 MHz, $CDCl_3$) of compound **3t**.

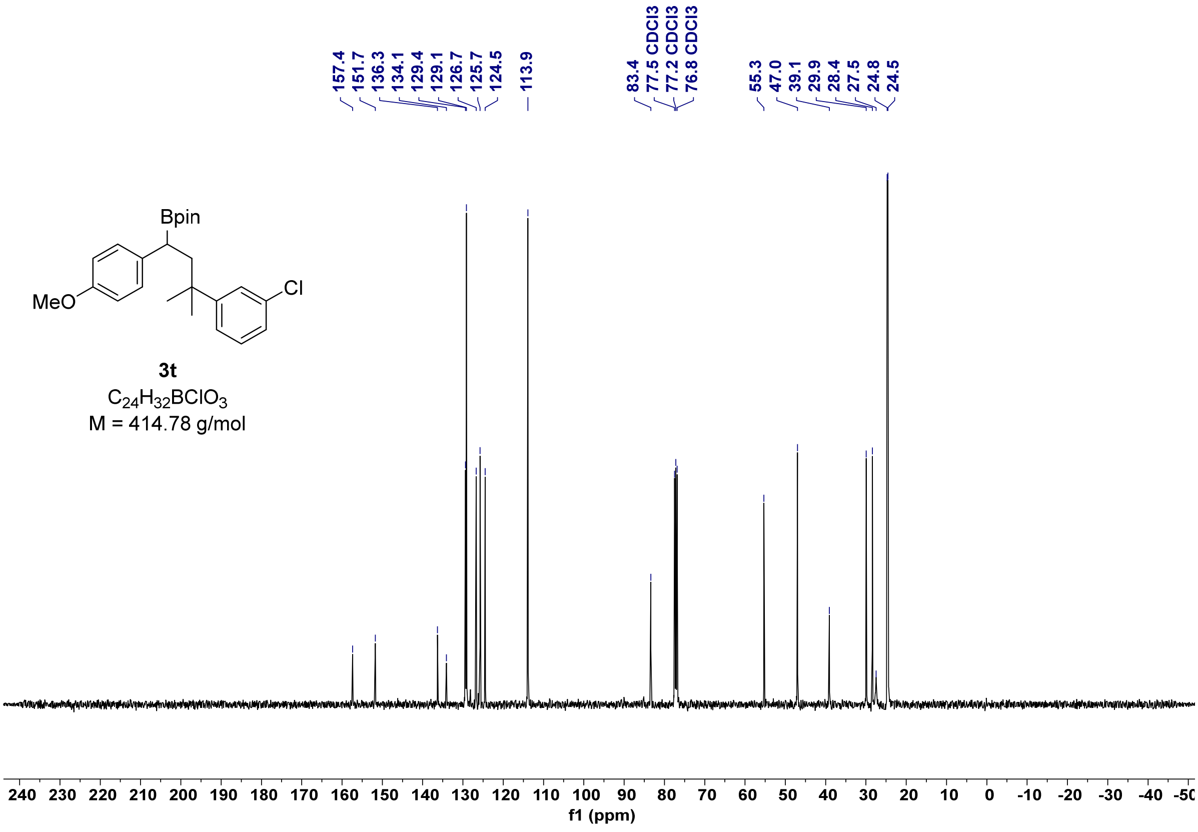

^{13}C{^{1}H} NMR spectrum (100 MHz, $CDCl_3$) of compound **3t**.

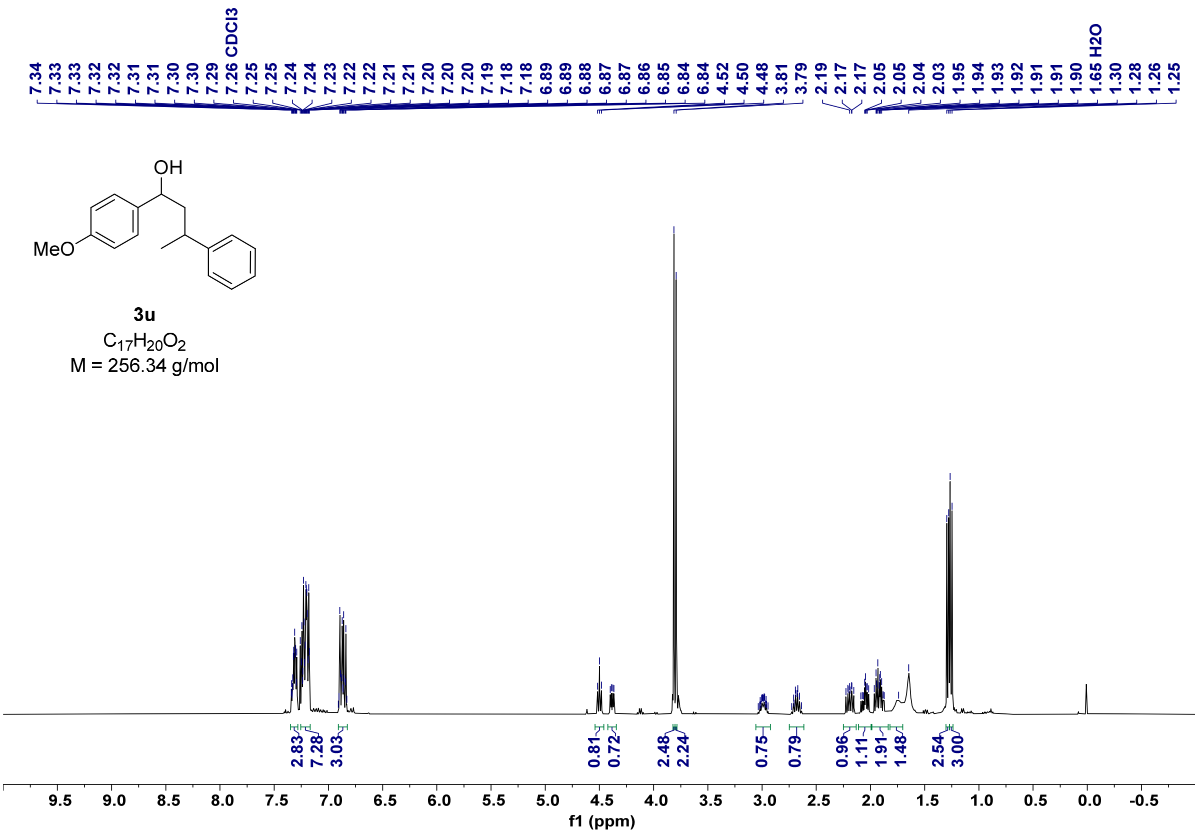

^{1}H NMR spectrum (400 MHz, $CDCl_3$) of compound **3u**.

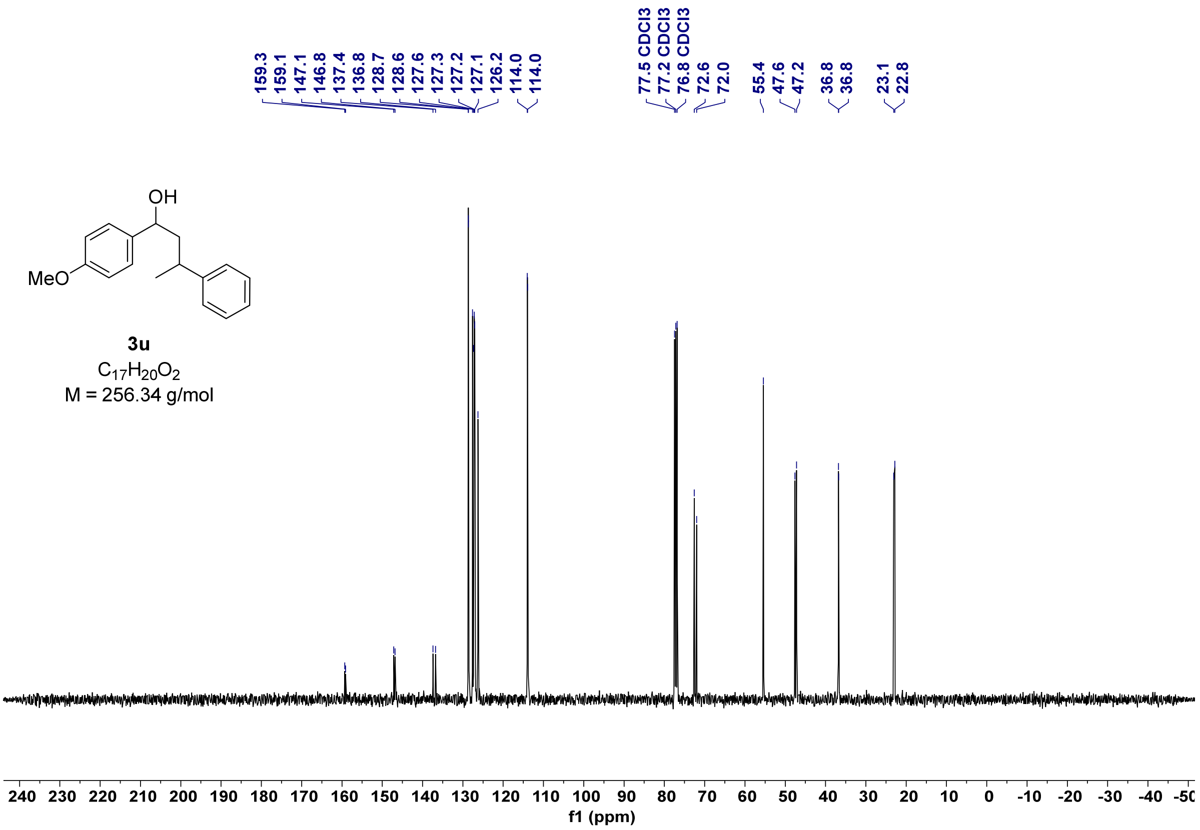

^{13}C{^{1}H} NMR spectrum (100 MHz, $CDCl_3$) of compound **3u**.

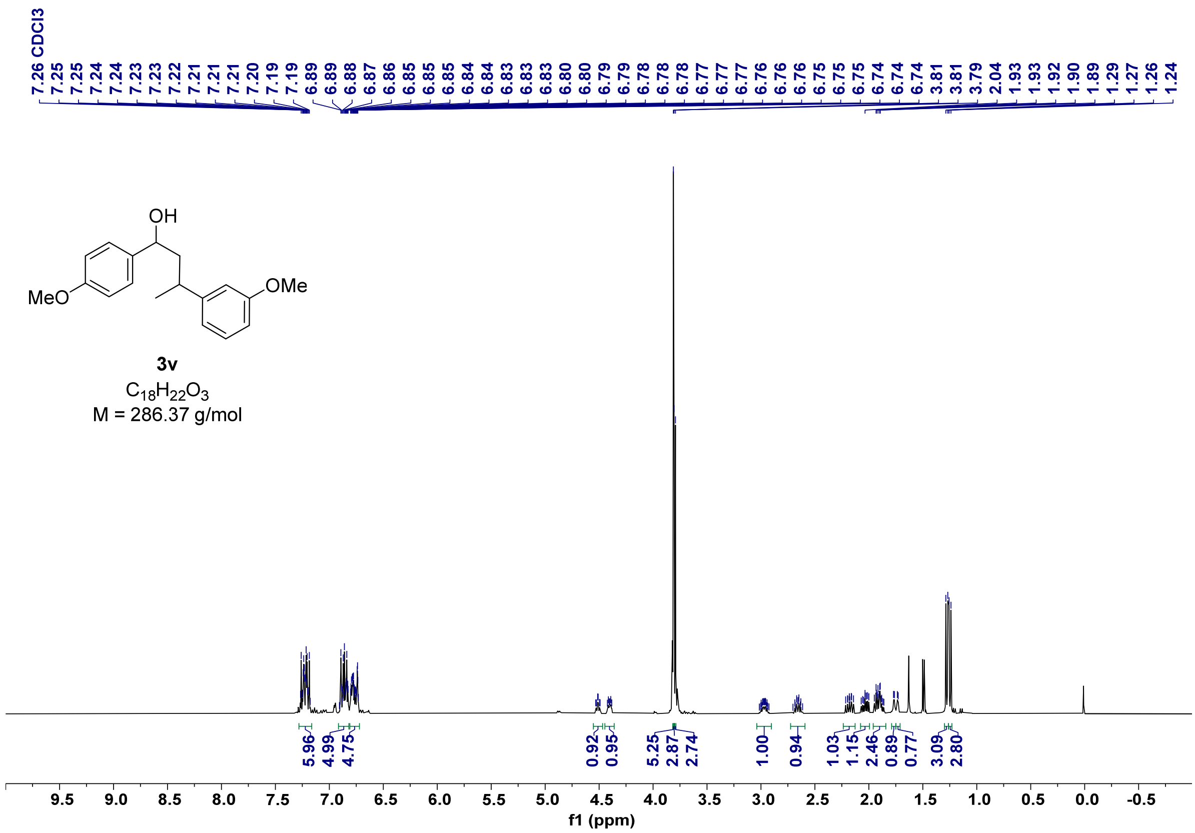

^{1}H NMR spectrum (400 MHz, $CDCl_3$) of compound **3v**.

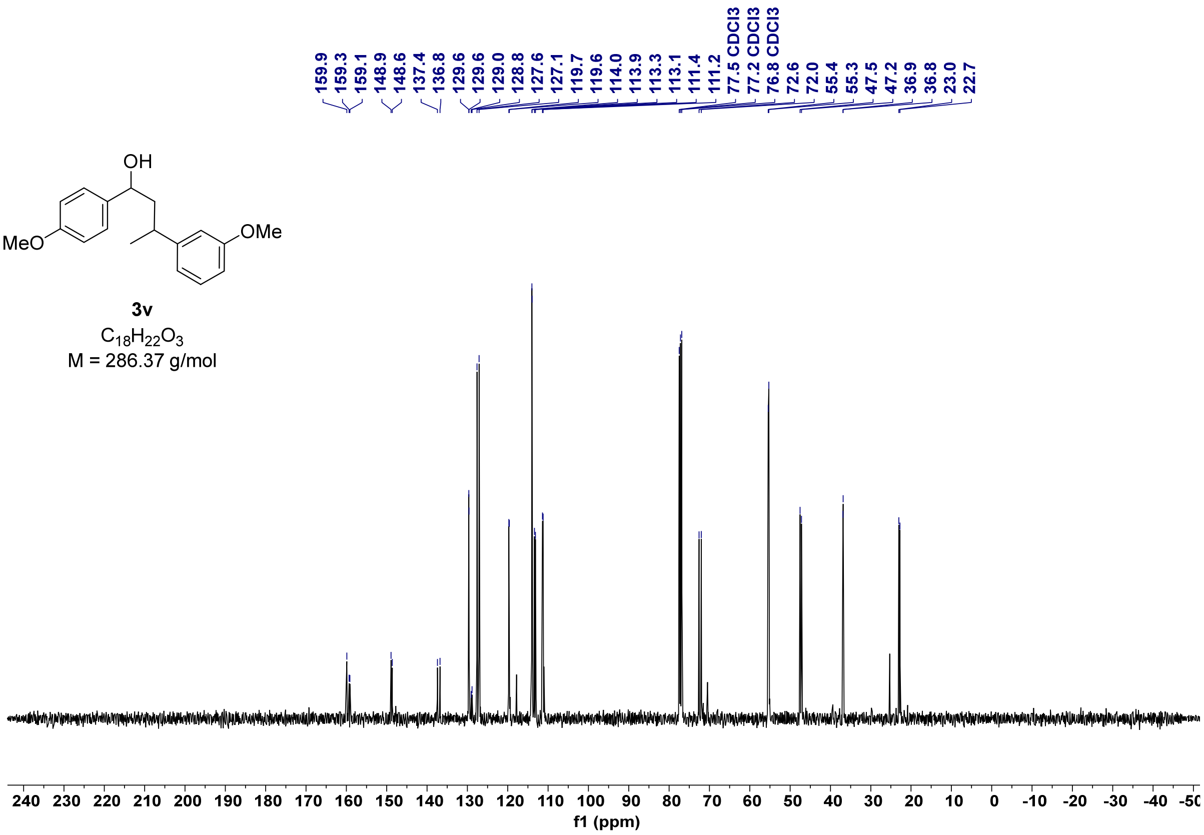

$^{13}C\{^1H\}$ NMR spectrum (100 MHz, $CDCl_3$) of compound **3v**.

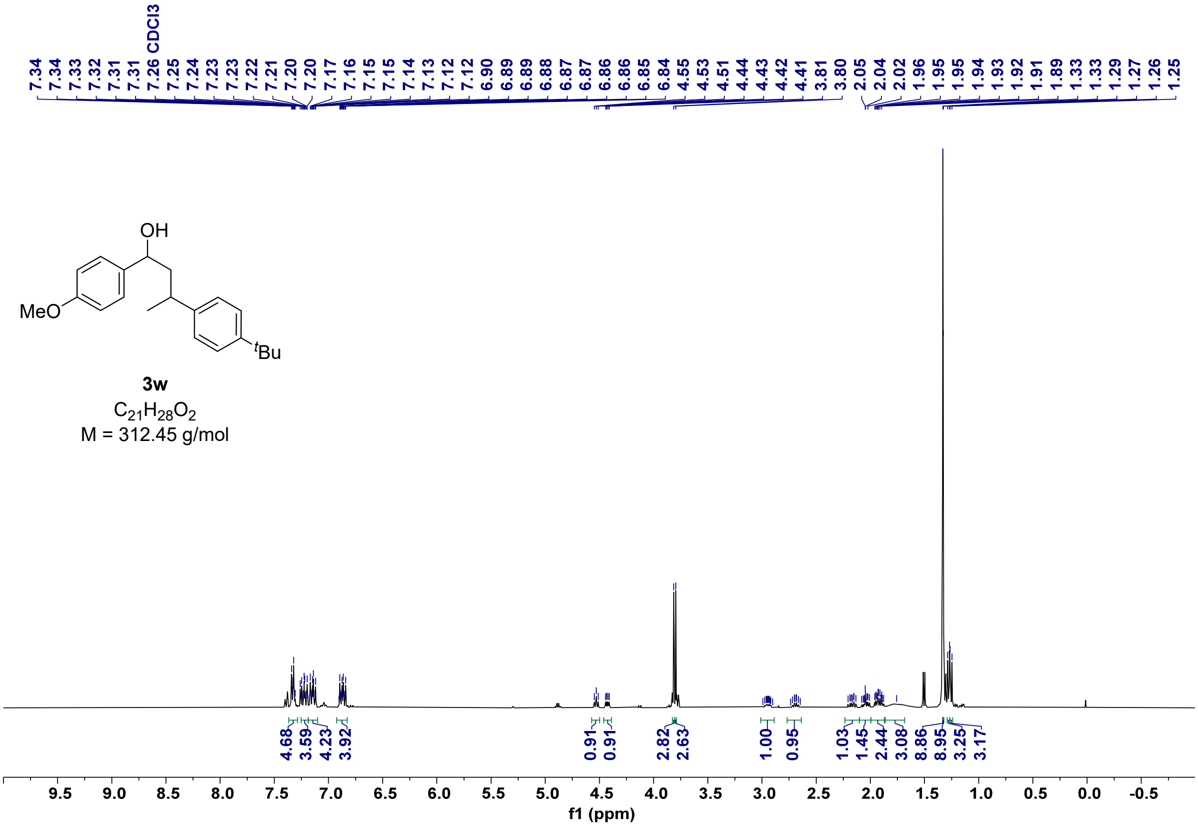

[1]**H NMR** spectrum (400 MHz, $CDCl_3$) of compound **3w**.

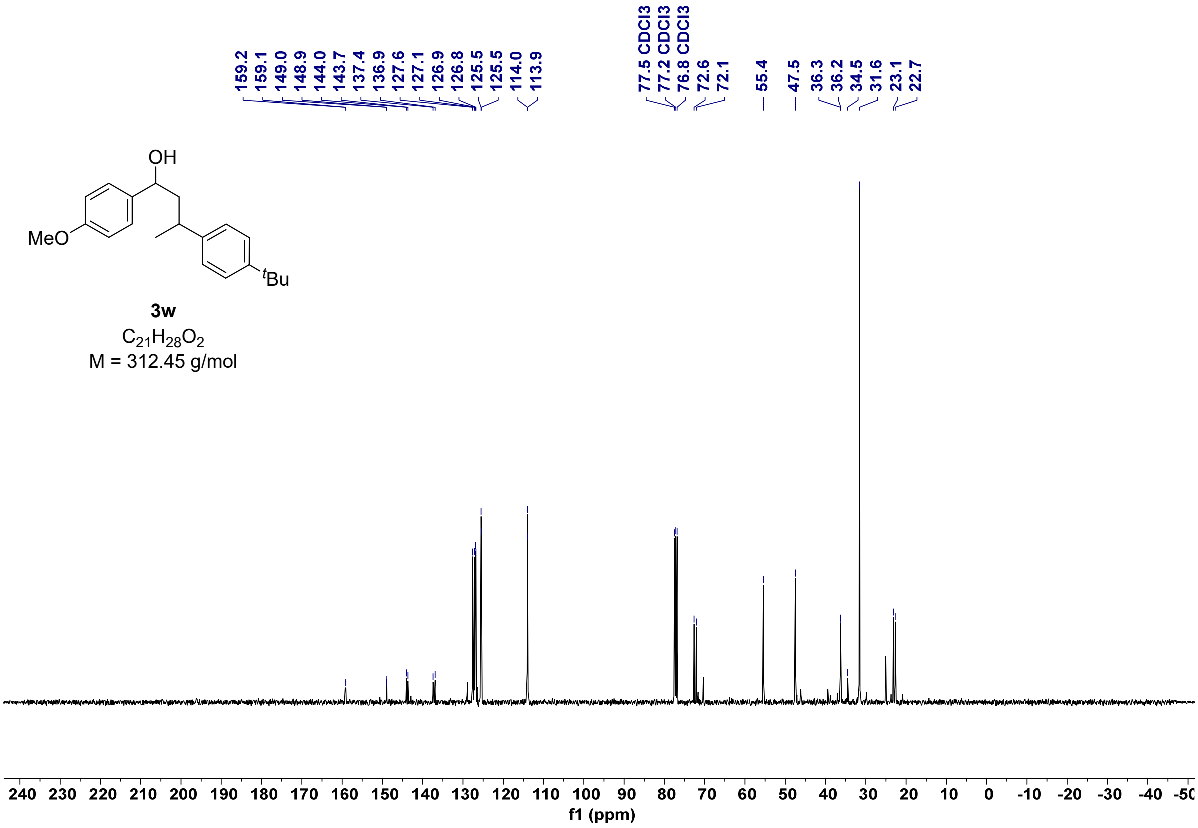

[13]**C{^{1}H} NMR** spectrum (100 MHz, $CDCl_3$) of compound **3w**.

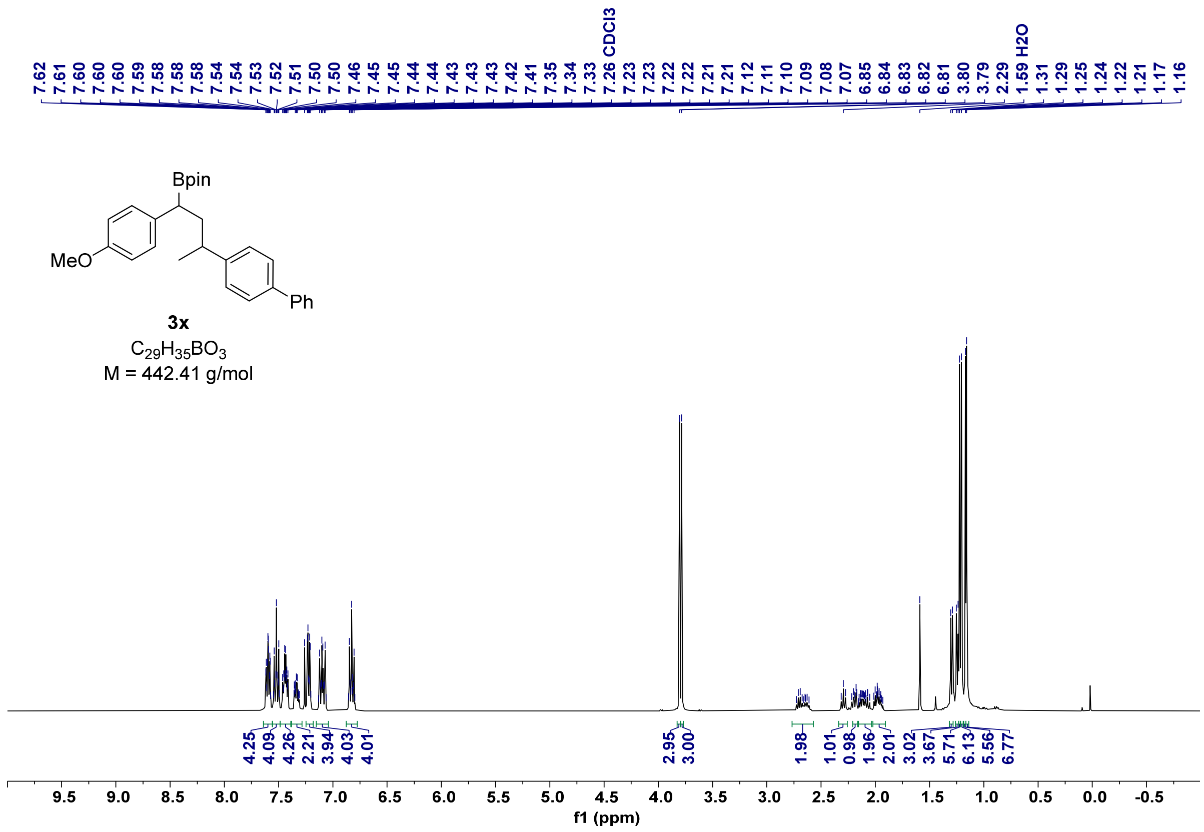

^{1}H NMR spectrum (400 MHz, $CDCl_3$) of compound **3x**.

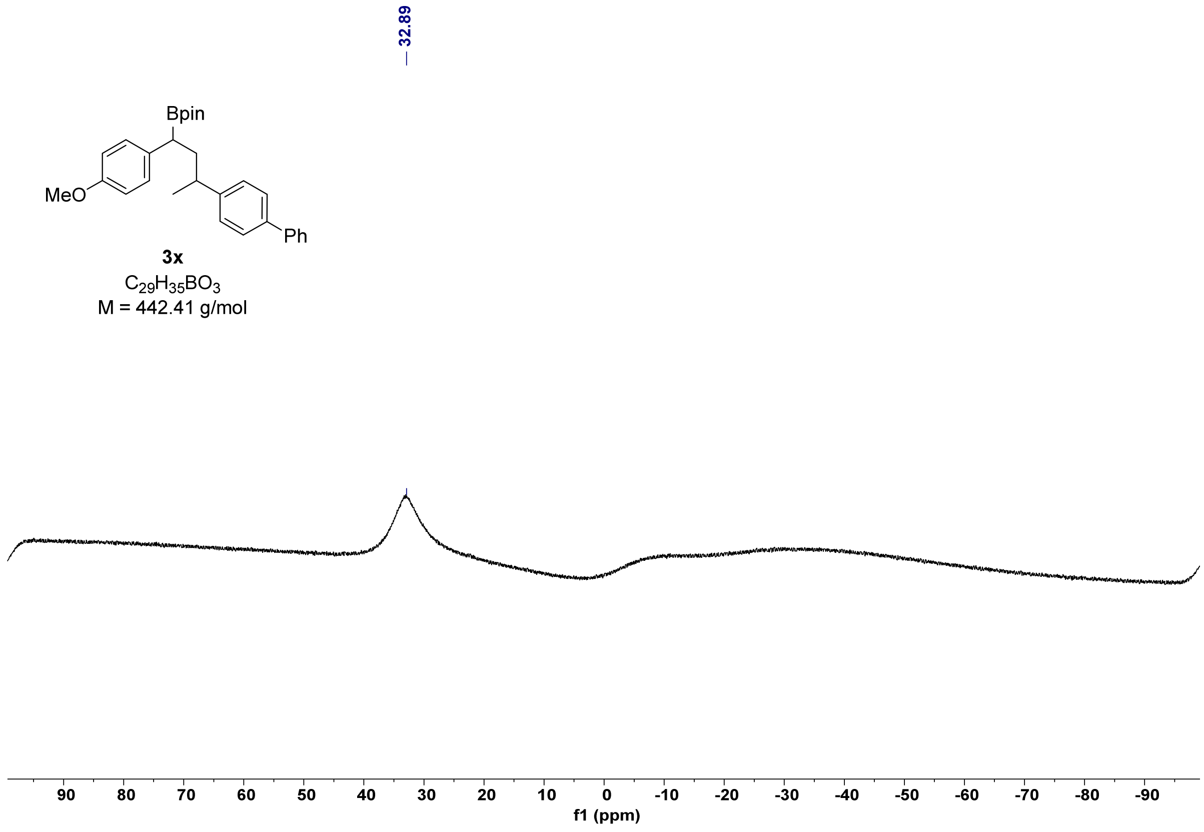

^{11}B NMR spectrum (400 MHz, $CDCl_3$) of compound **3x**.

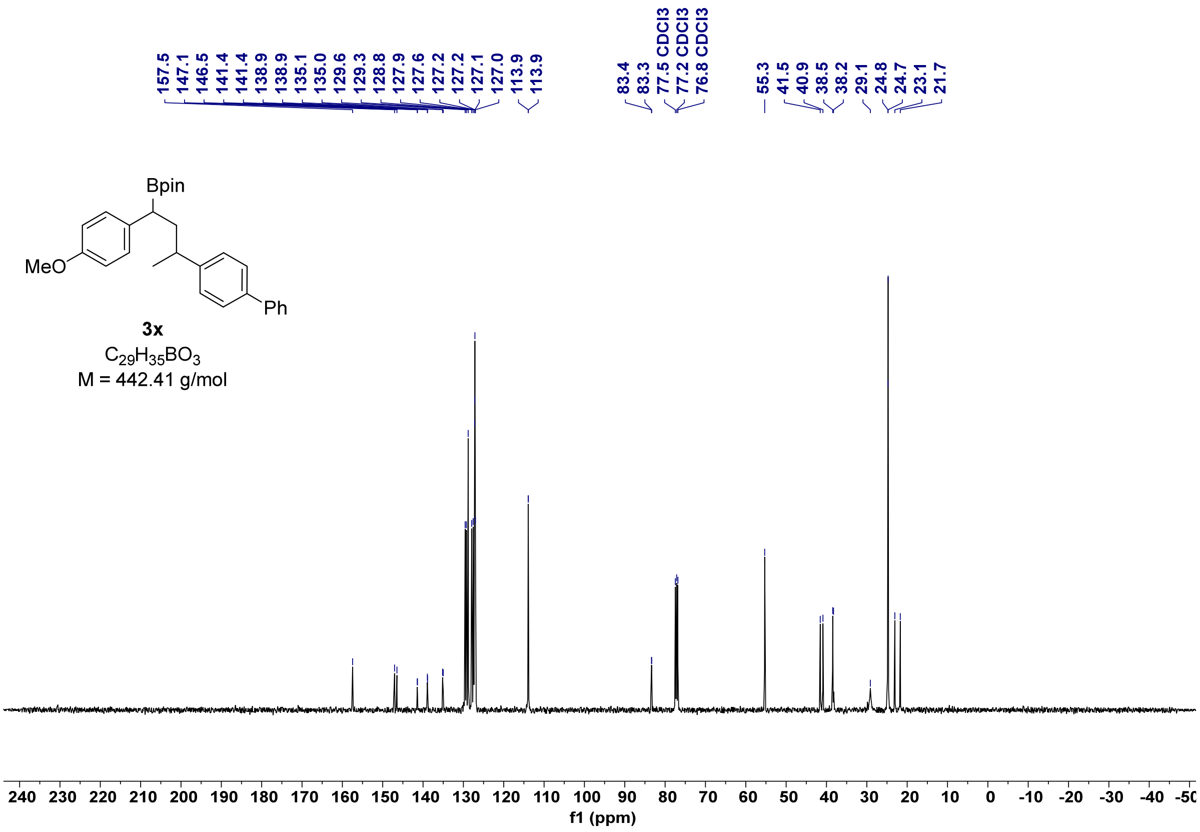

^{13}**C{^{1}H} NMR** spectrum (100 MHz, $CDCl_3$) of compound **3x**.

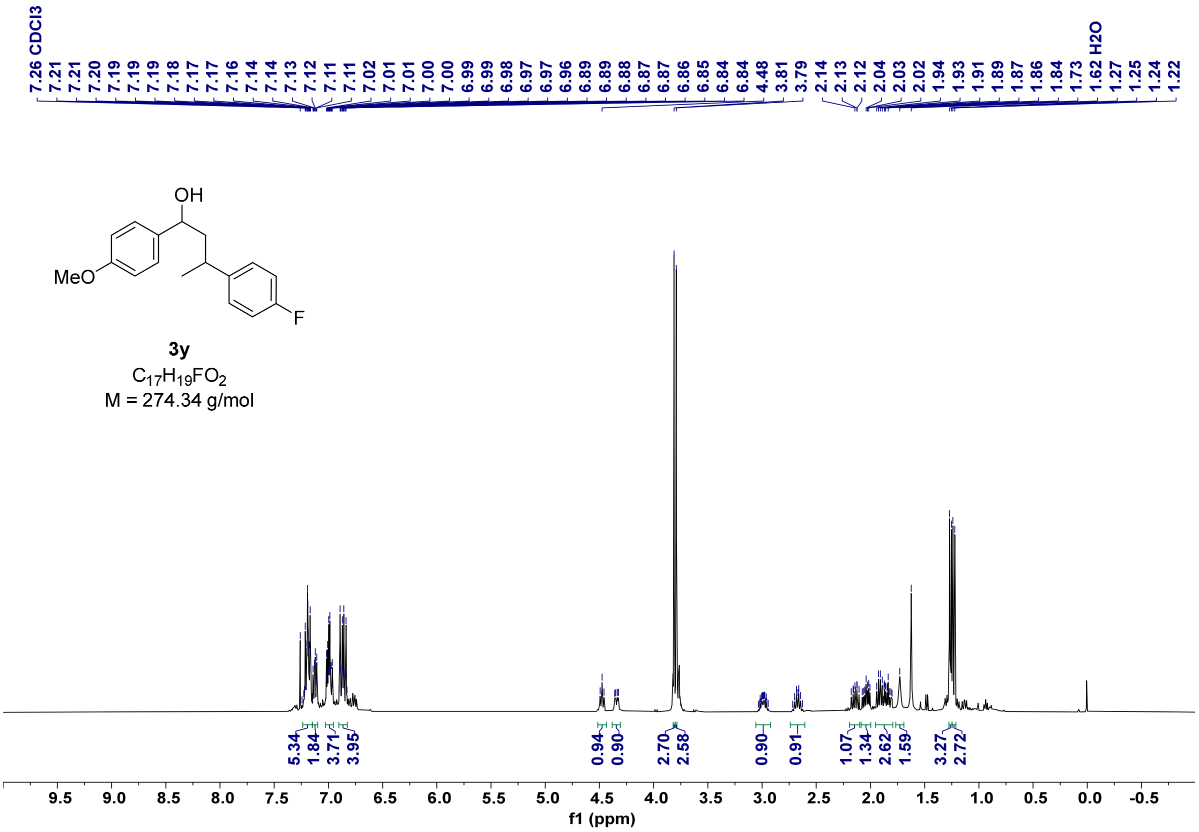

^{1}**H NMR** spectrum (400 MHz, $CDCl_3$) of compound **3y**.

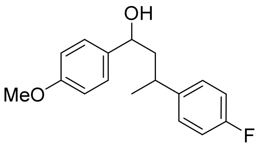

3y
$C_{17}H_{19}FO_2$
M = 274.34 g/mol

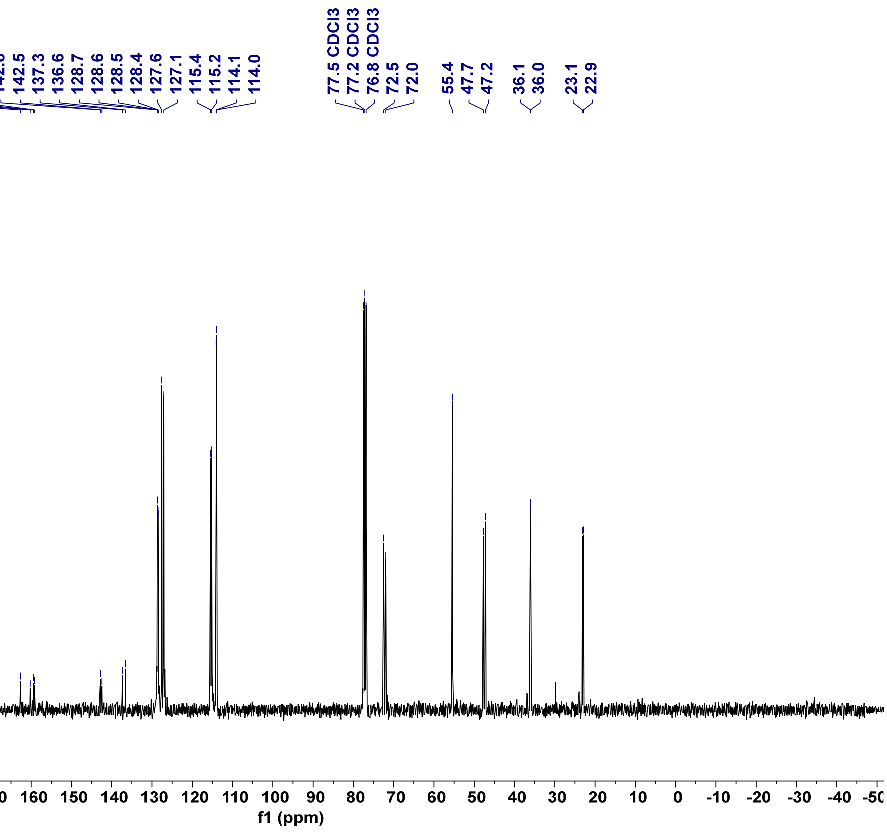

$^{13}C\{^1H\}$ NMR spectrum (100 MHz, $CDCl_3$) of compound **3y**.

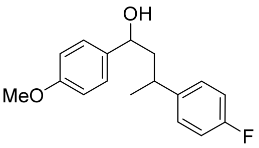

3y
$C_{17}H_{19}FO_2$
M = 274.34 g/mol

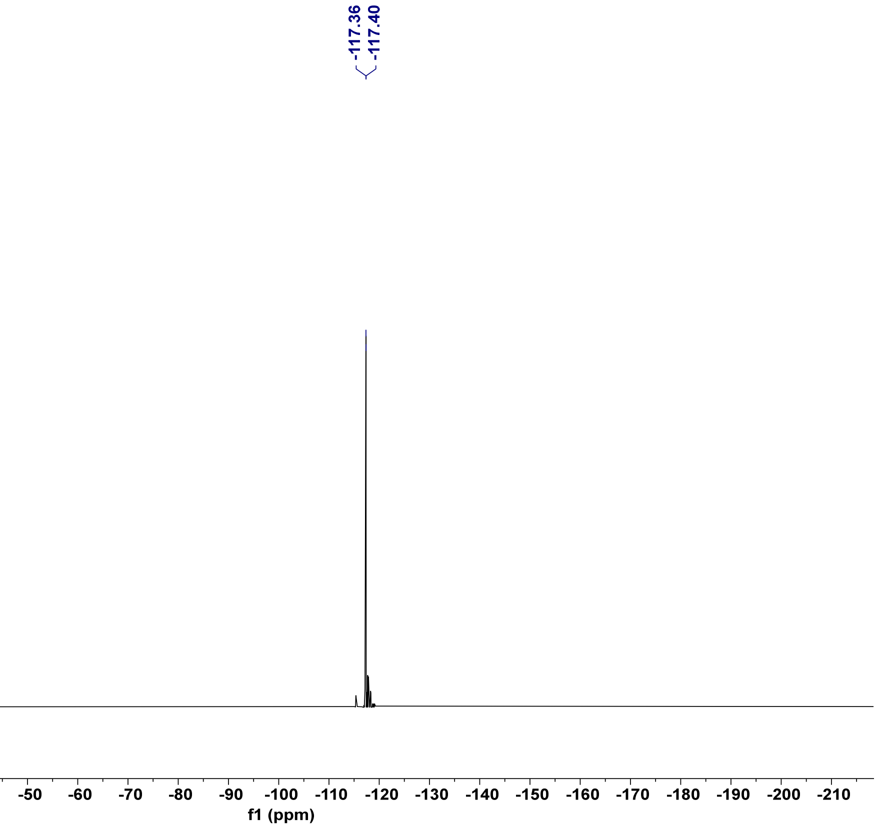

^{19}F NMR spectrum (400 MHz, $CDCl_3$) of compound **3y**.

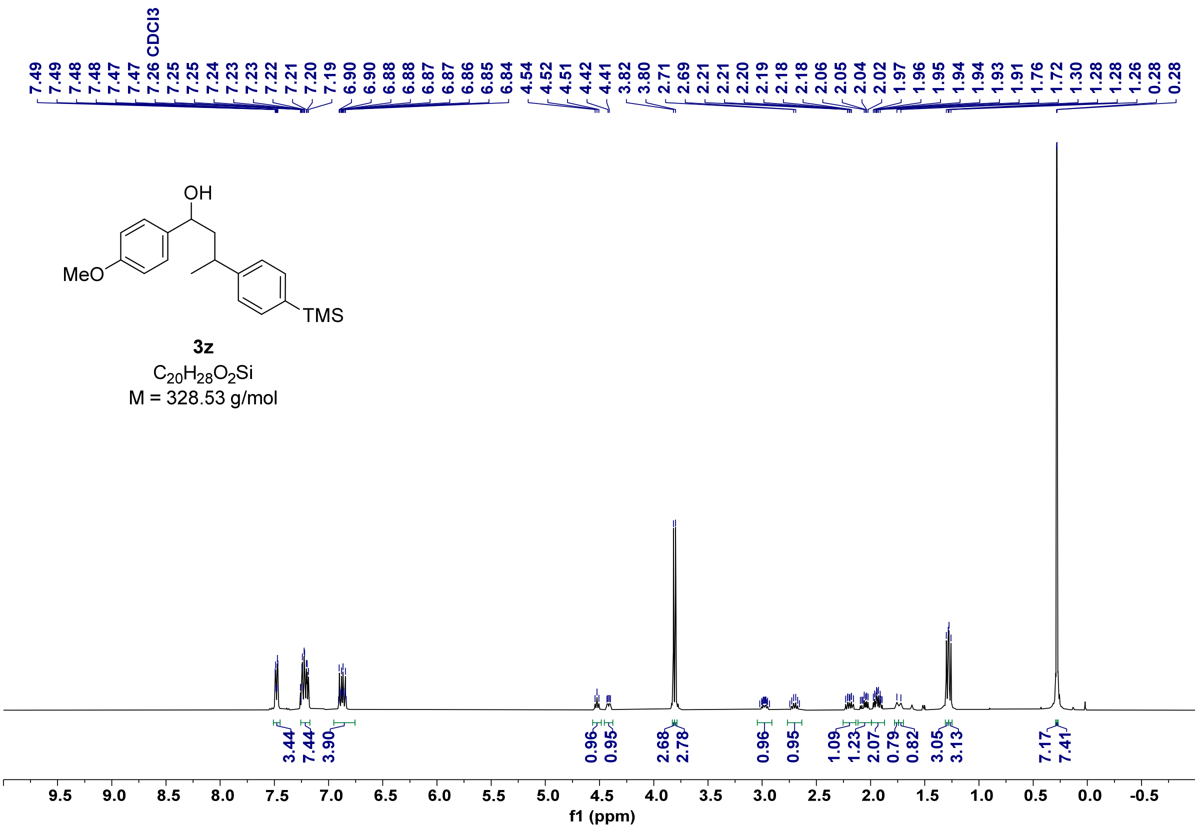

^{1}H NMR spectrum (400 MHz, $CDCl_3$) of compound **3z**.

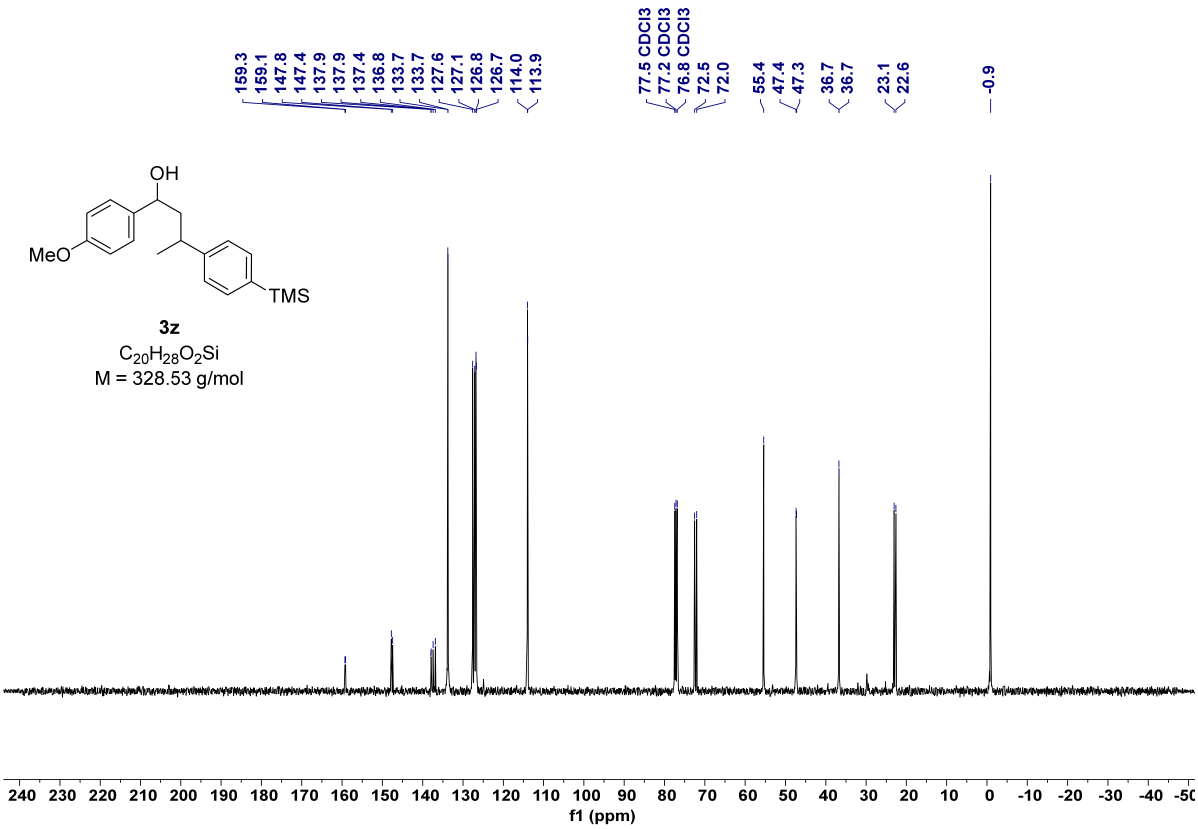

^{13}C{^{1}H} NMR spectrum (100 MHz, $CDCl_3$) of compound **3z**.

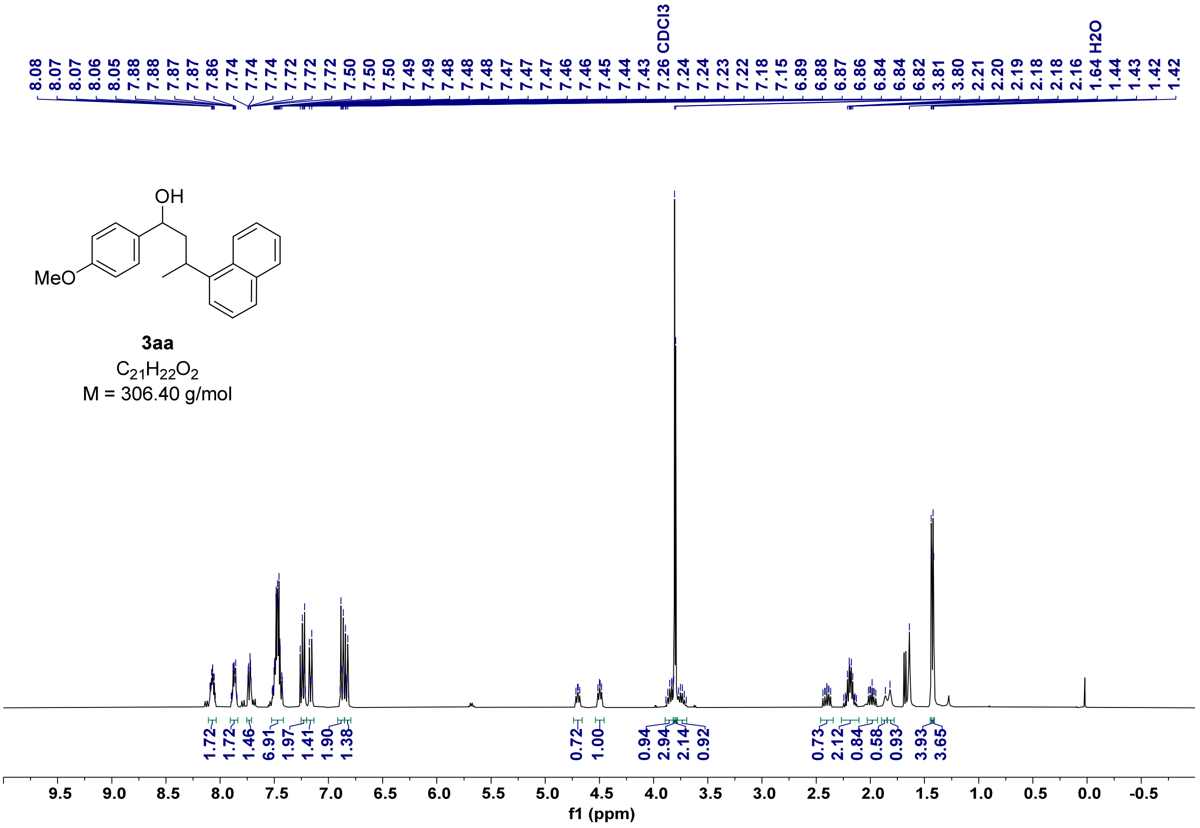

^{1}H NMR spectrum (400 MHz, $CDCl_3$) of compound **3aa**.

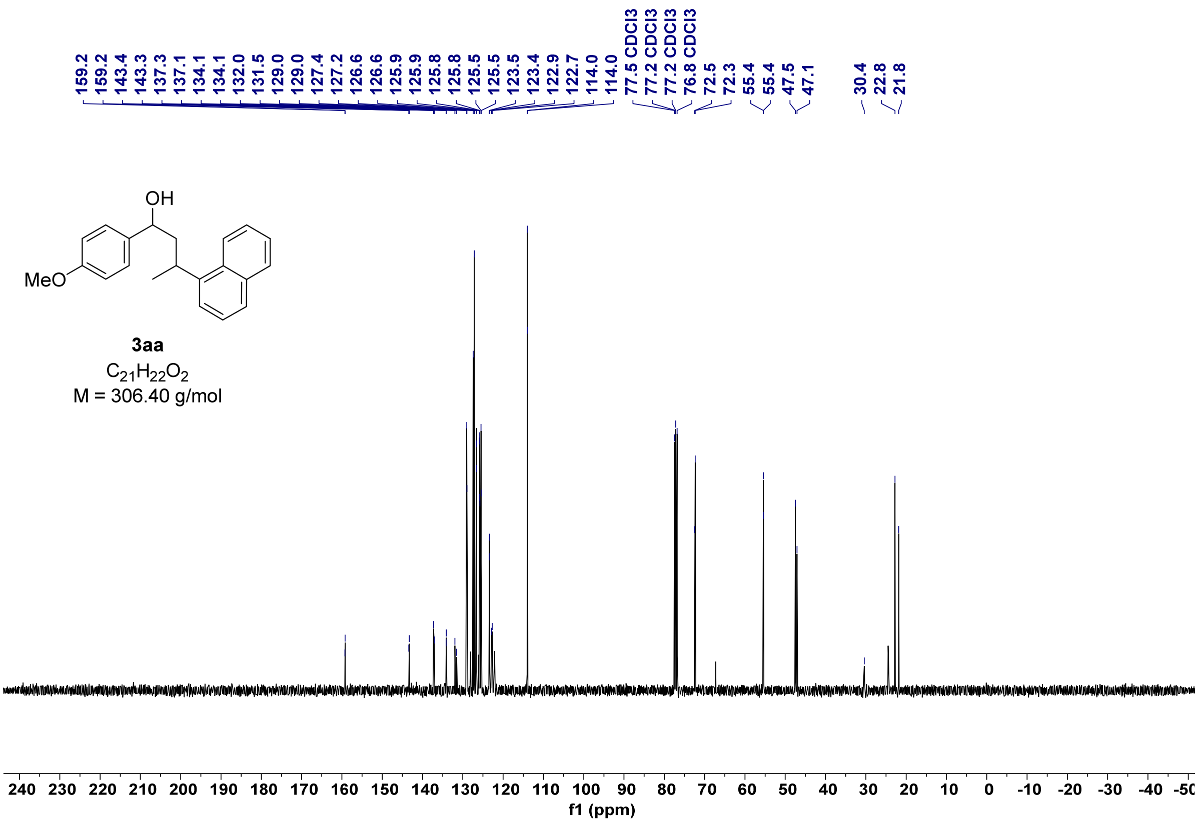

$^{13}C\{^1H\}$ NMR spectrum (100 MHz, $CDCl_3$) of compound **3aa.**

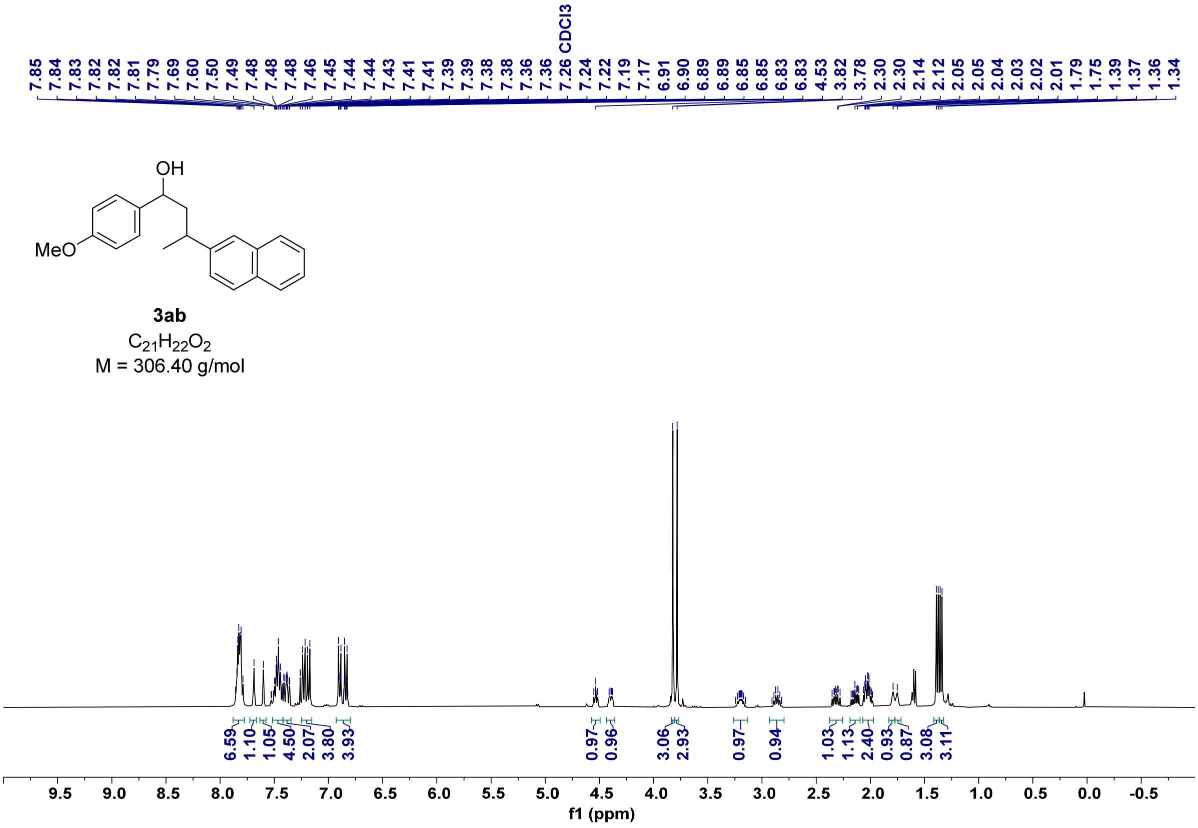

^{1}**H NMR** spectrum (400 MHz, $CDCl_3$) of compound **3ab**.

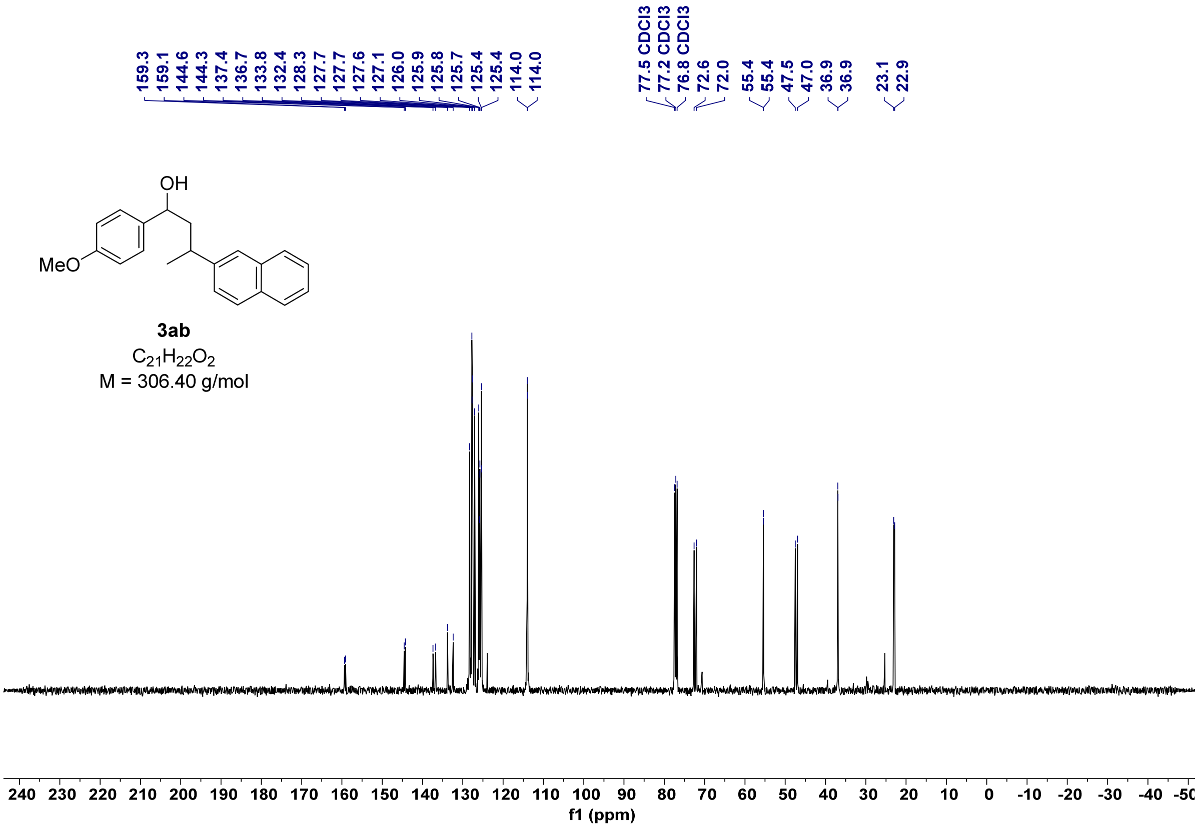

^{13}**C{^{1}H} NMR** spectrum (100 MHz, $CDCl_3$) of compound **3ab**.

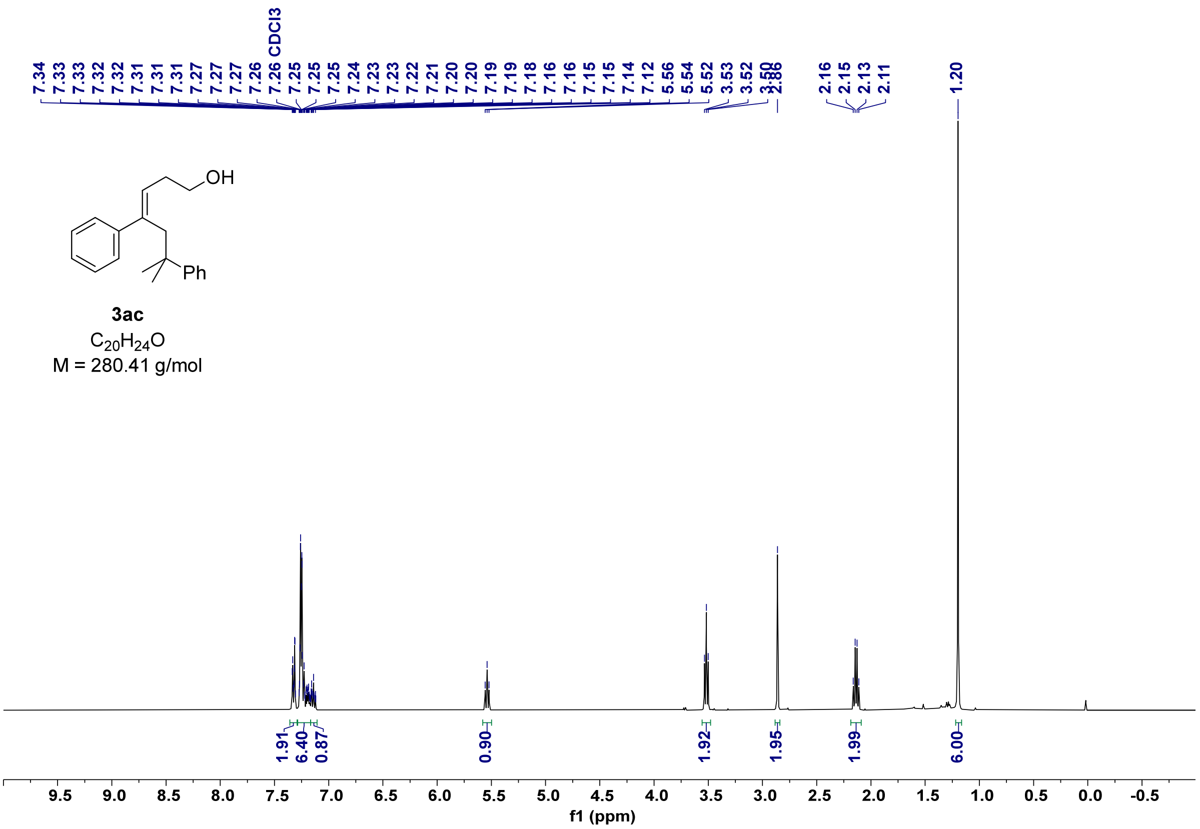

^{1}H NMR spectrum (400 MHz, $CDCl_3$) of compound **3ac**.

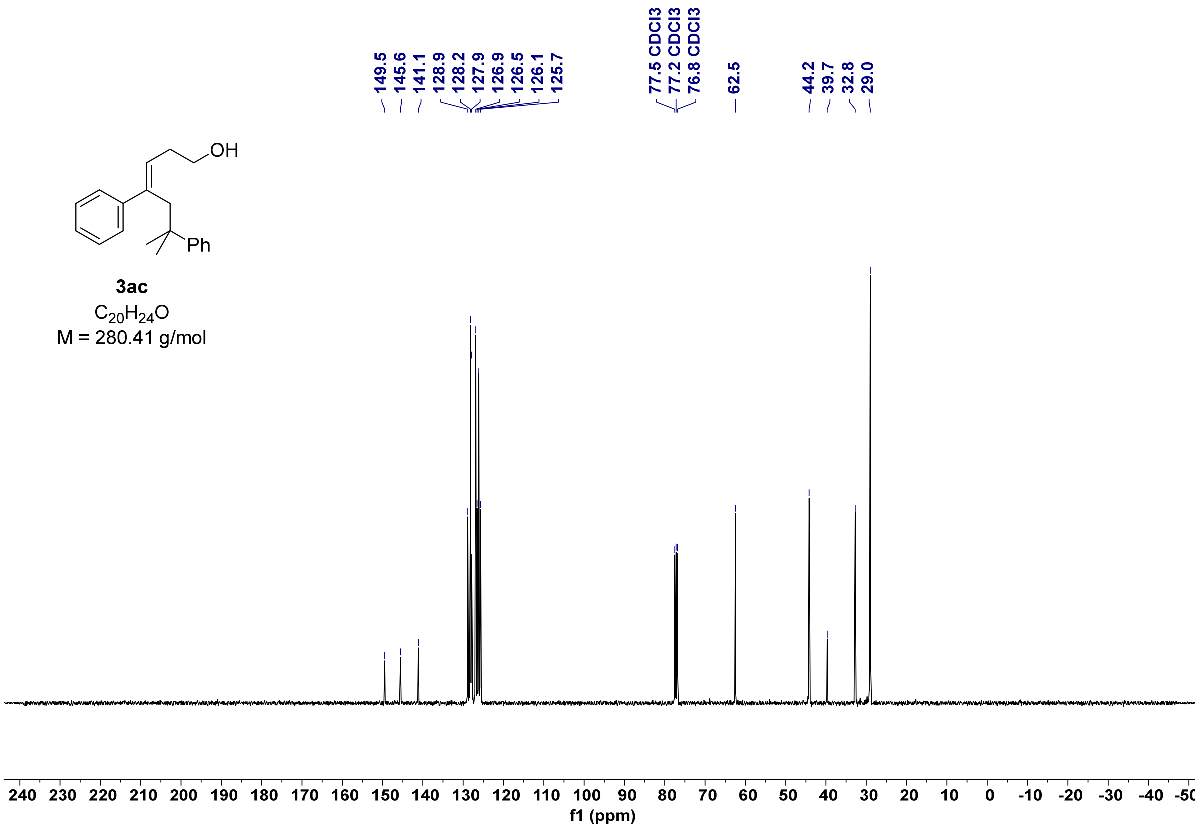

$^{13}C\{^{1}H\}$ NMR spectrum (100 MHz, $CDCl_3$) of compound **3ac**.

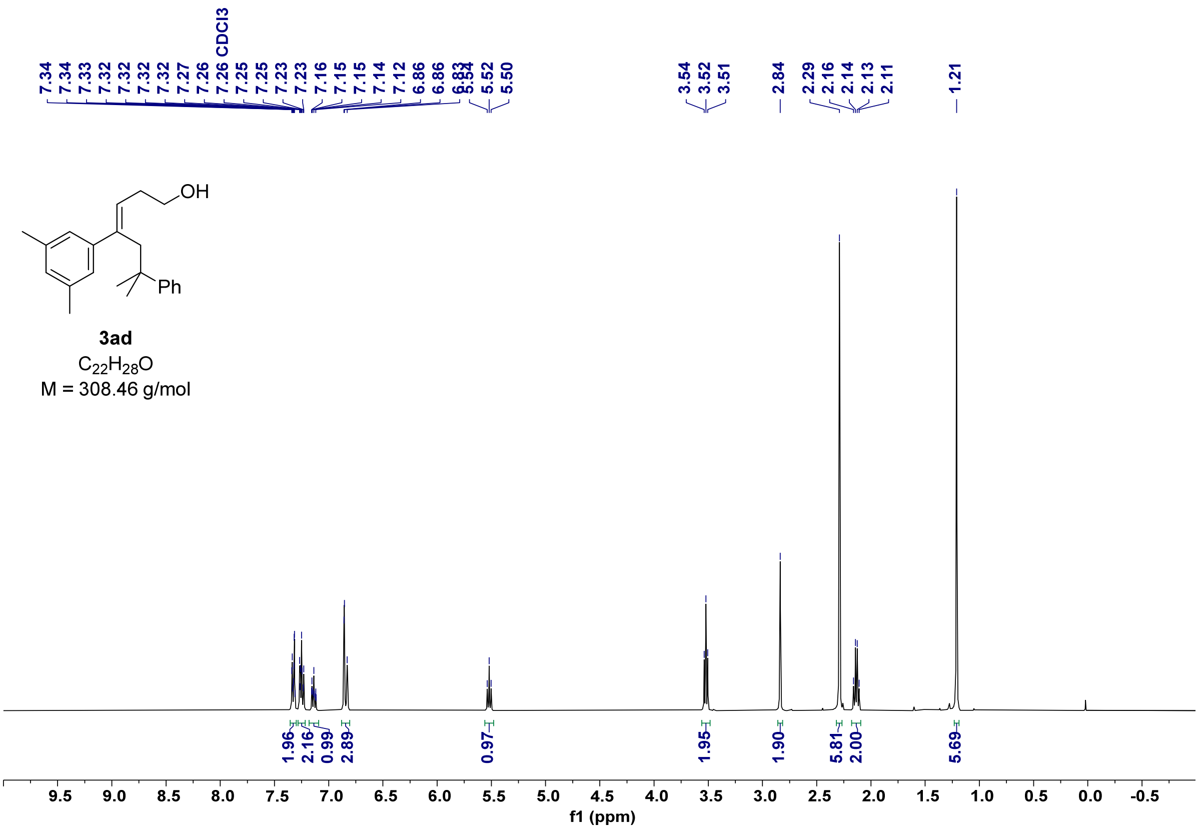

^{1}**H NMR** spectrum (400 MHz, $CDCl_3$) of compound **3ad**.

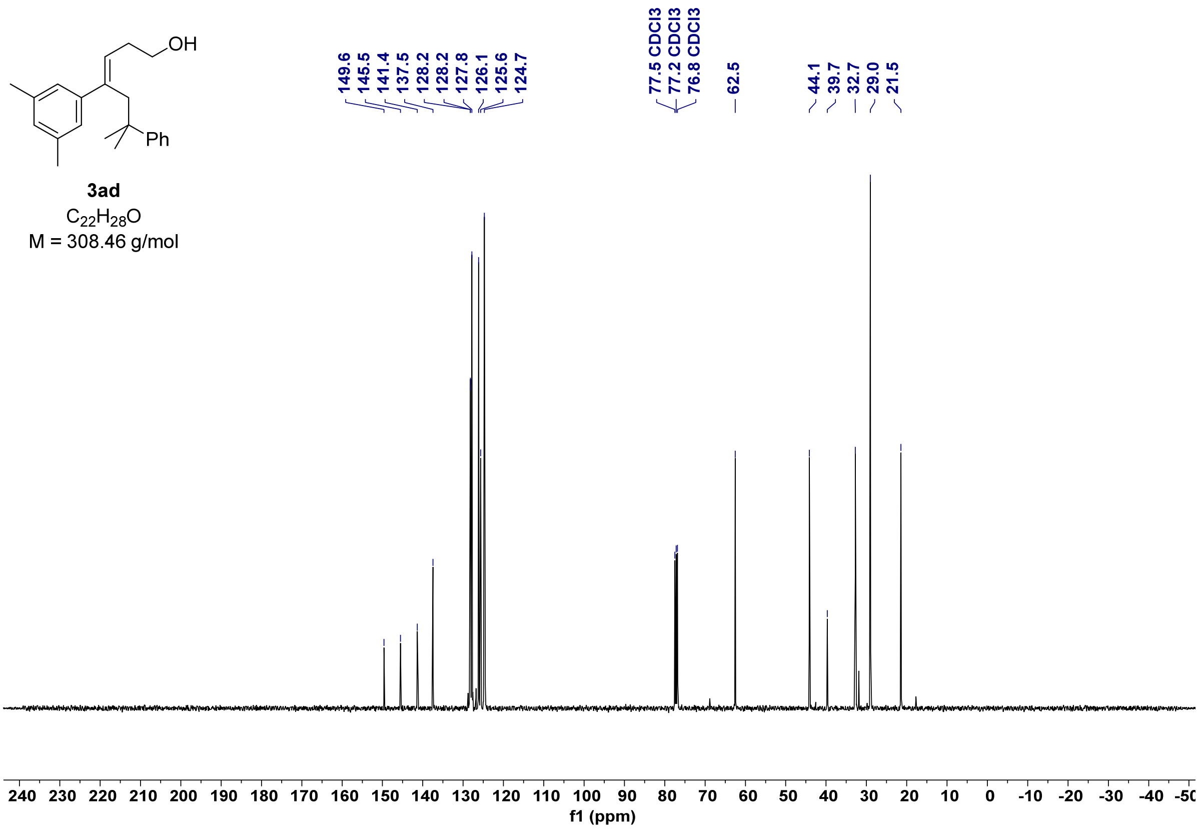

^{13}**C{^{1}H} NMR** spectrum (100 MHz, $CDCl_3$) of compound **3ad**.

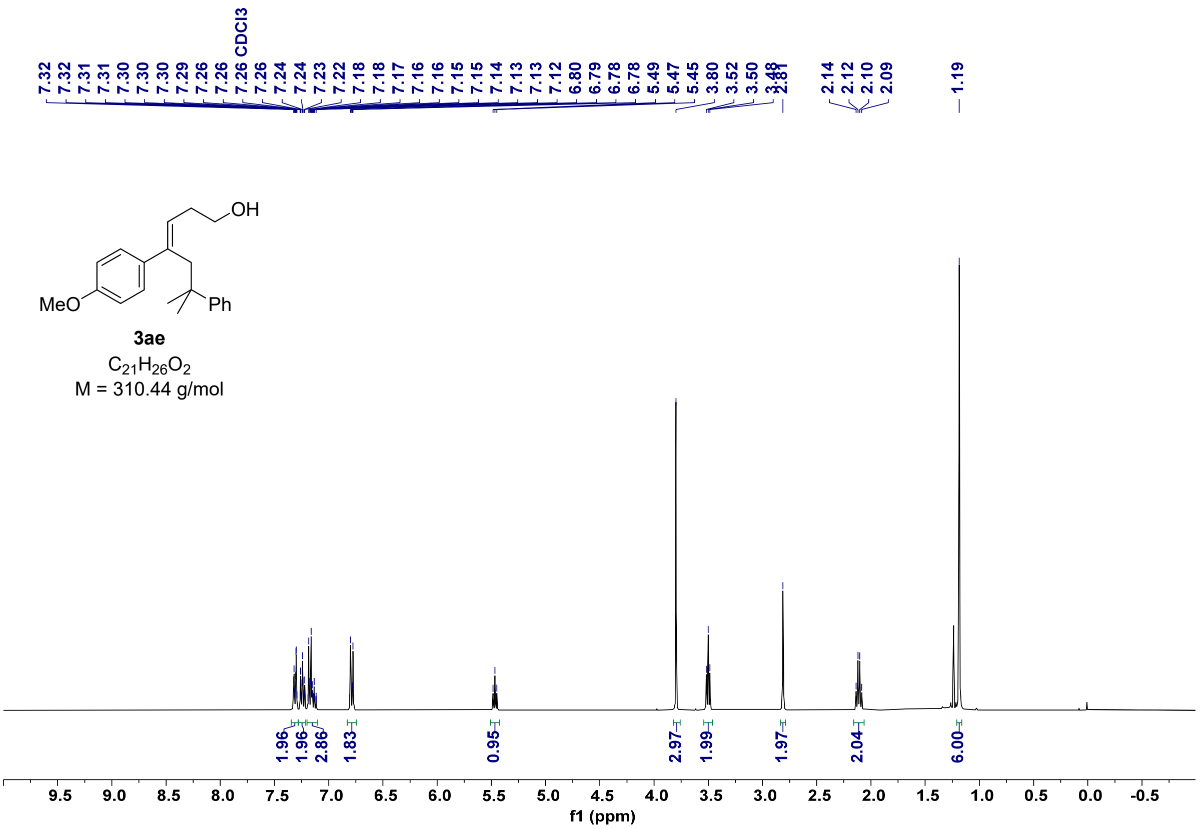

^{1}**H NMR** spectrum (400 MHz, $CDCl_3$) of compound **3ae**.

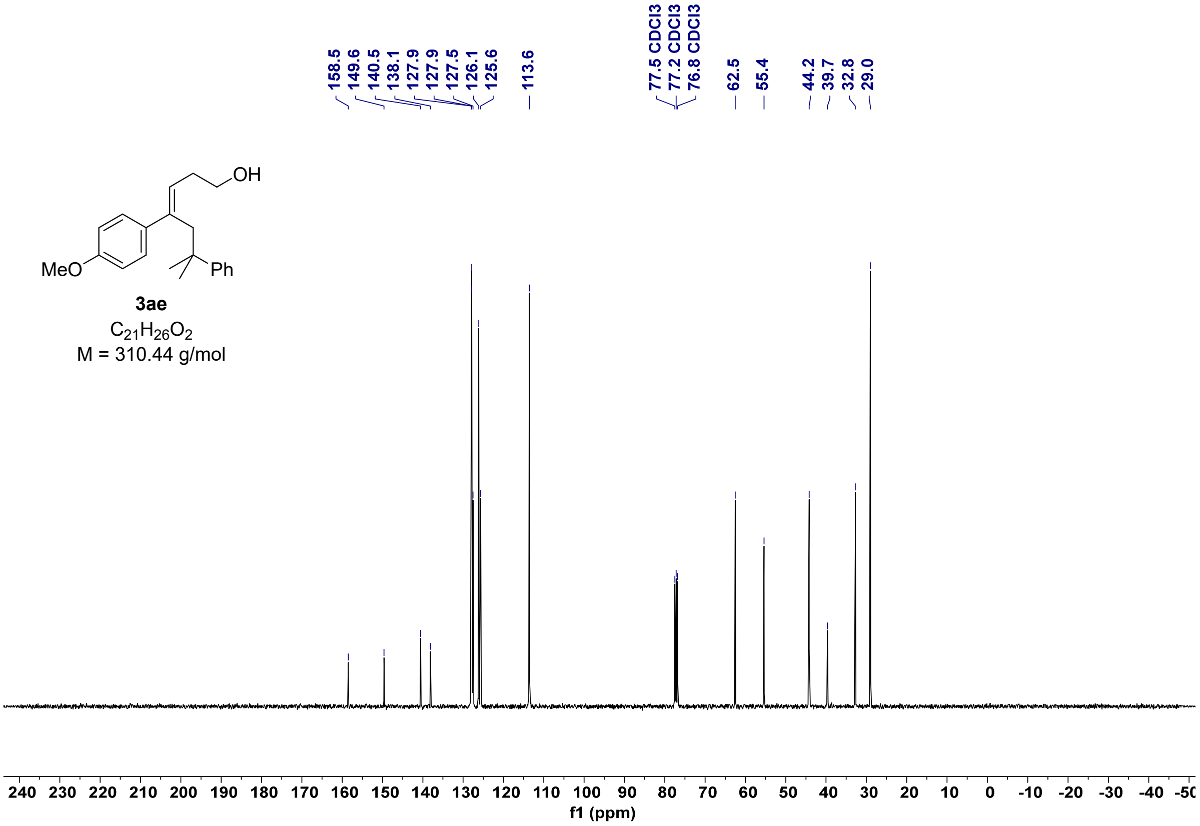

^{13}**C{^{1}H} NMR** spectrum (100 MHz, $CDCl_3$) of compound **3ae**.

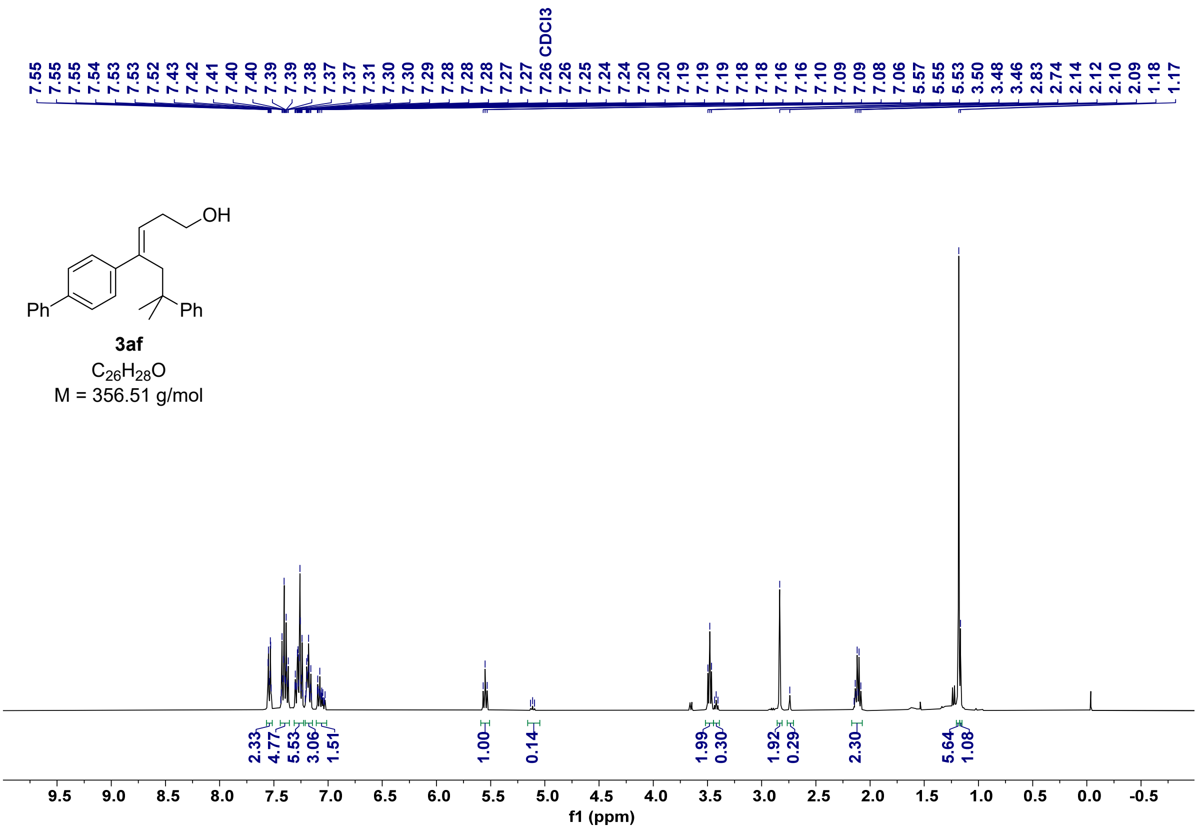

^{1}H NMR spectrum (400 MHz, $CDCl_3$) of compound **3af**.

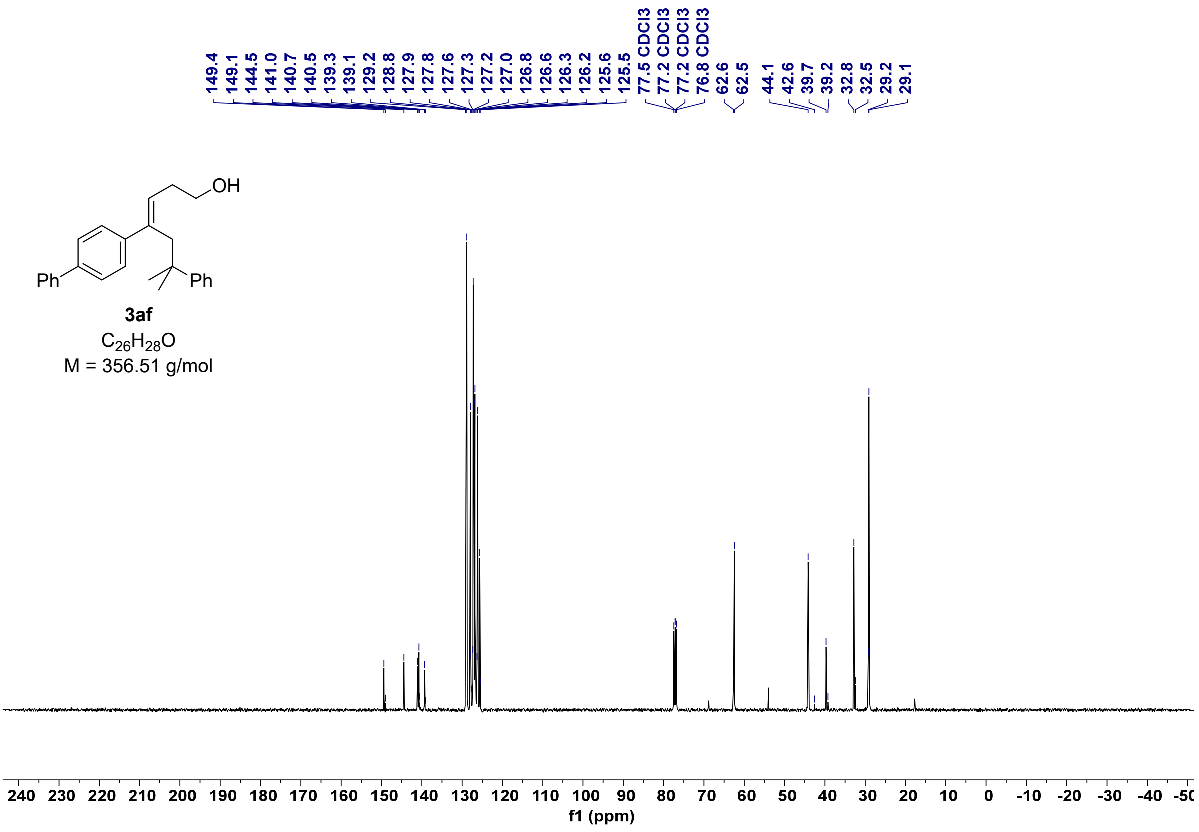

^{13}C{^{1}H} NMR spectrum (100 MHz, $CDCl_3$) of compound **3af**.

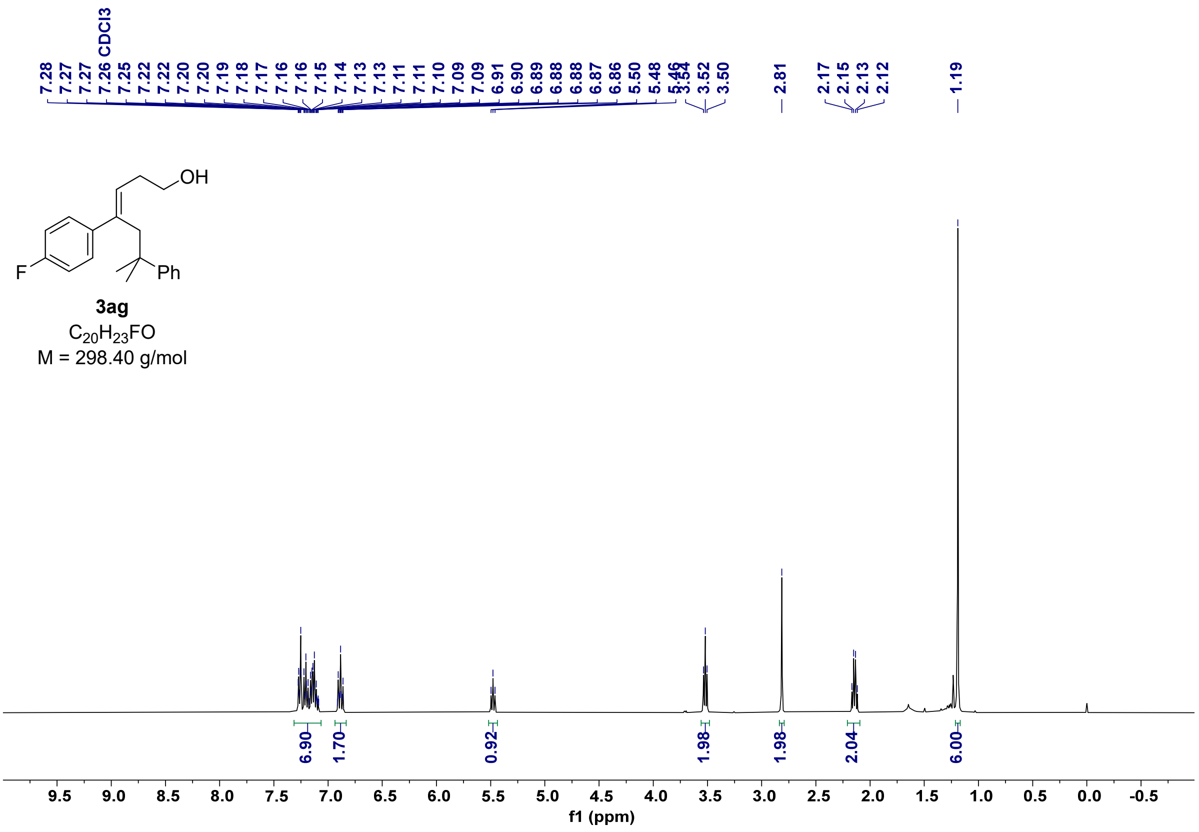

^{1}H NMR spectrum (400 MHz, $CDCl_3$) of compound **3ag**.

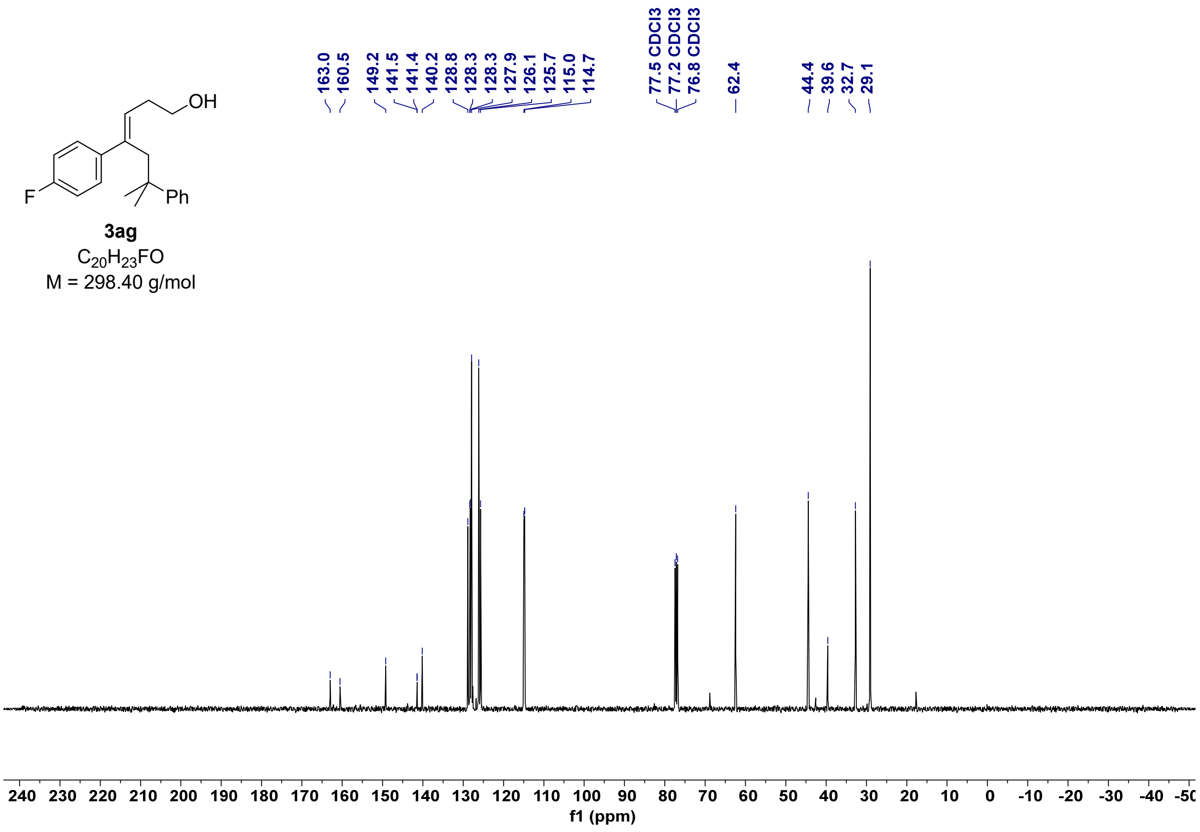

$^{13}C\{^1H\}$ NMR spectrum (100 MHz, $CDCl_3$) of compound **3ag**.

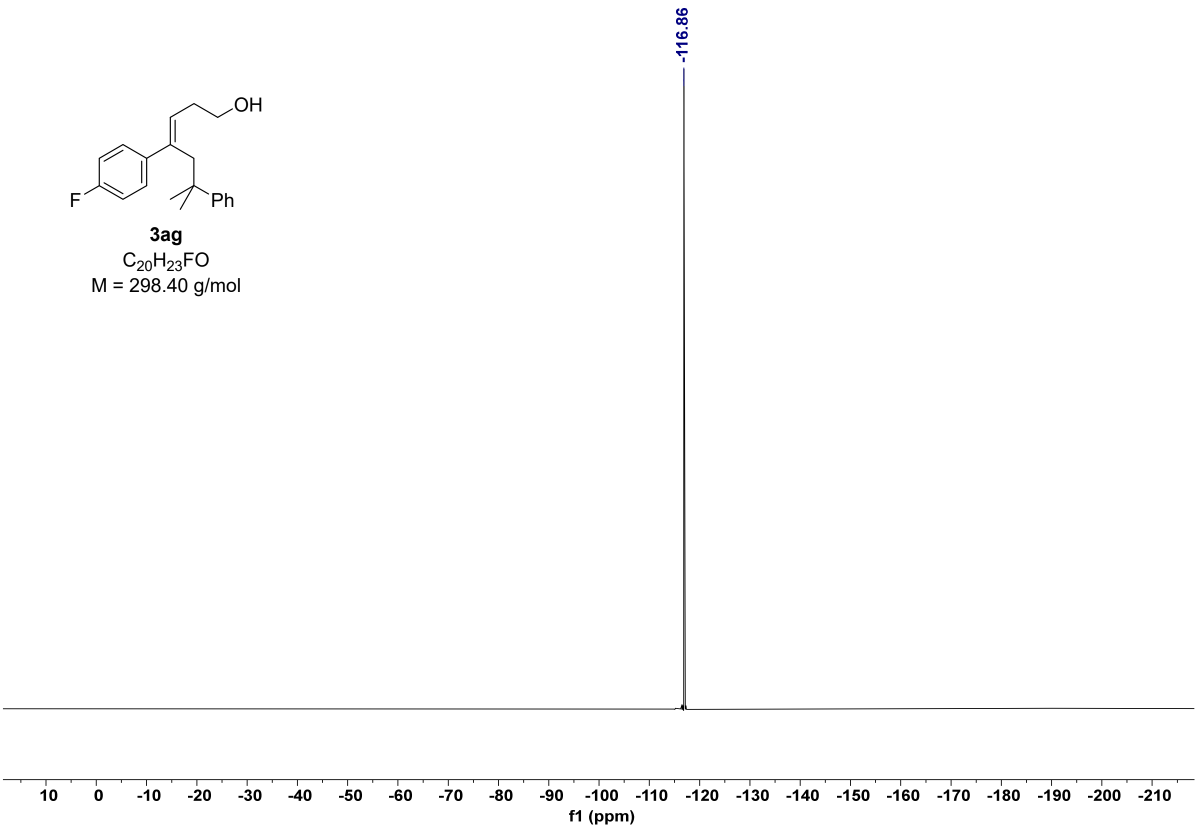

^{19}F NMR spectrum (400 MHz, $CDCl_3$) of compound **3ag**.

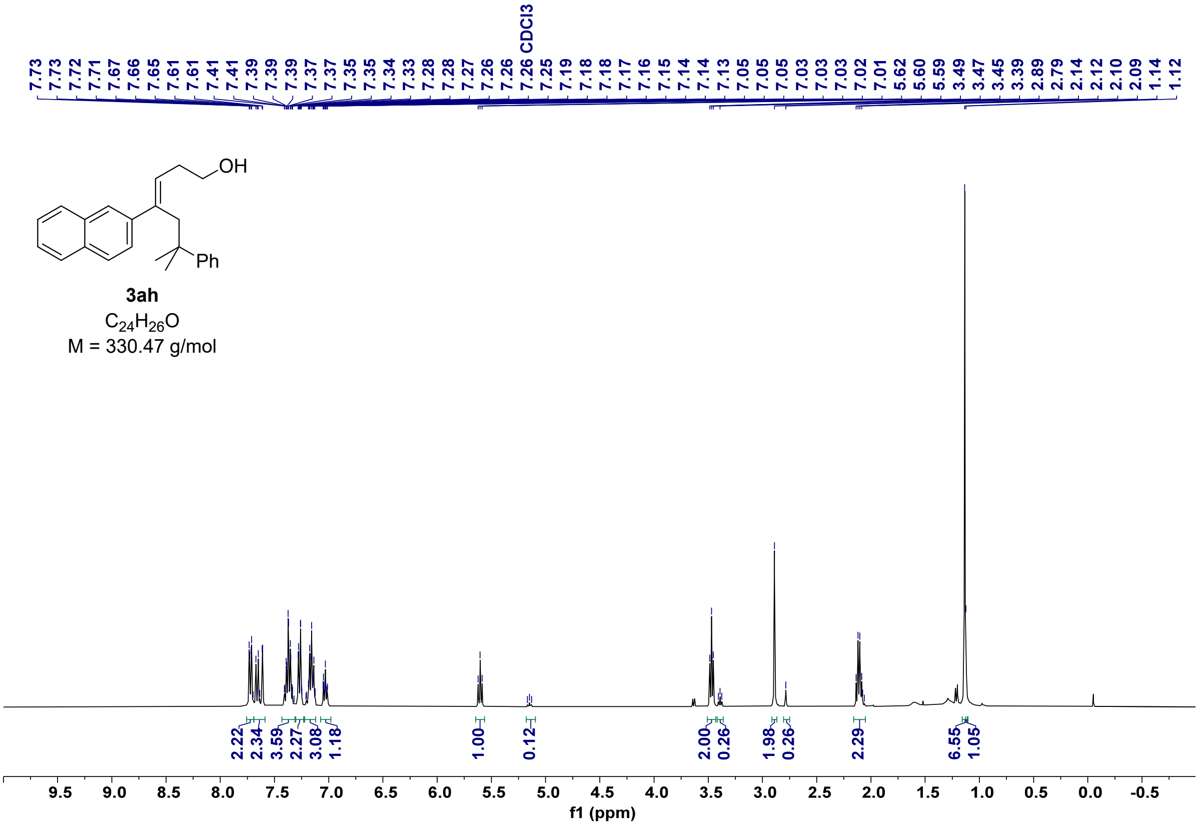

^{1}H NMR spectrum (400 MHz, $CDCl_3$) of compound **3ah**.

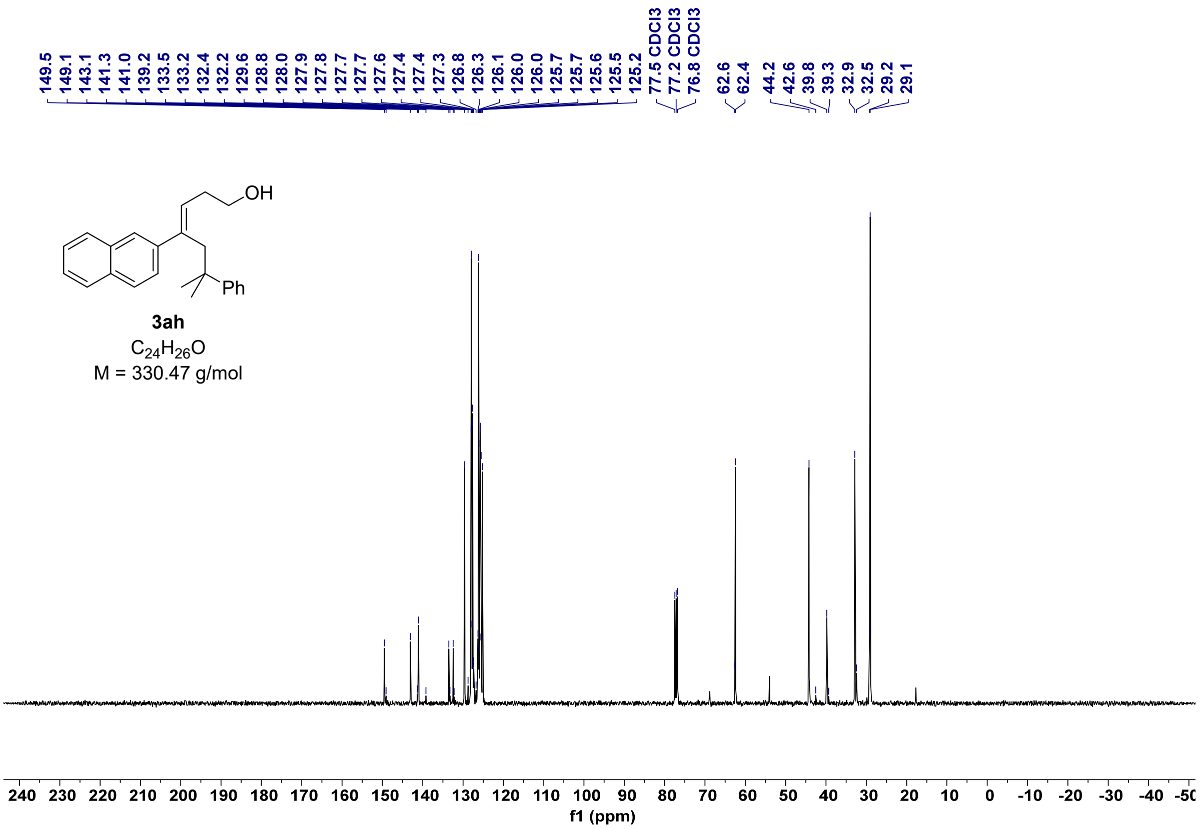

^{13}C{^{1}H} NMR spectrum (100 MHz, $CDCl_3$) of compound **3ah**.

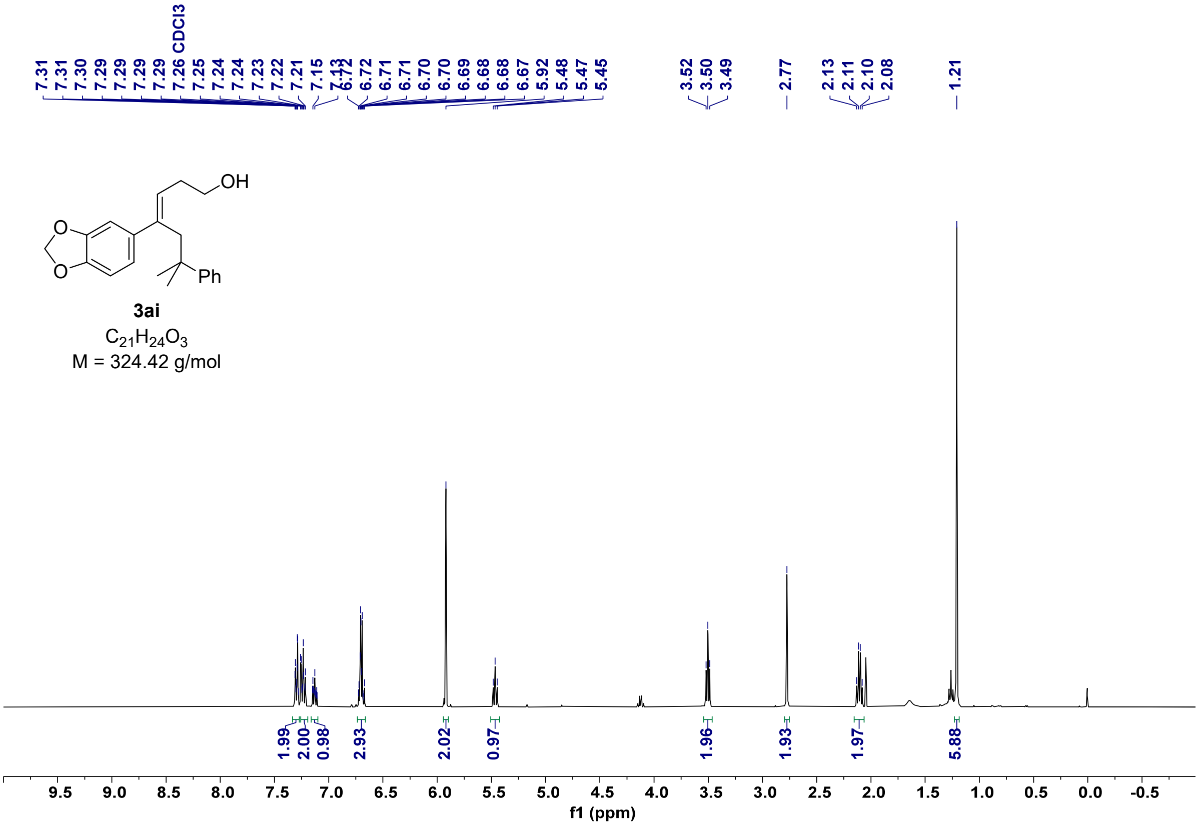

^{1}H NMR spectrum (400 MHz, $CDCl_3$) of compound **3ai**.

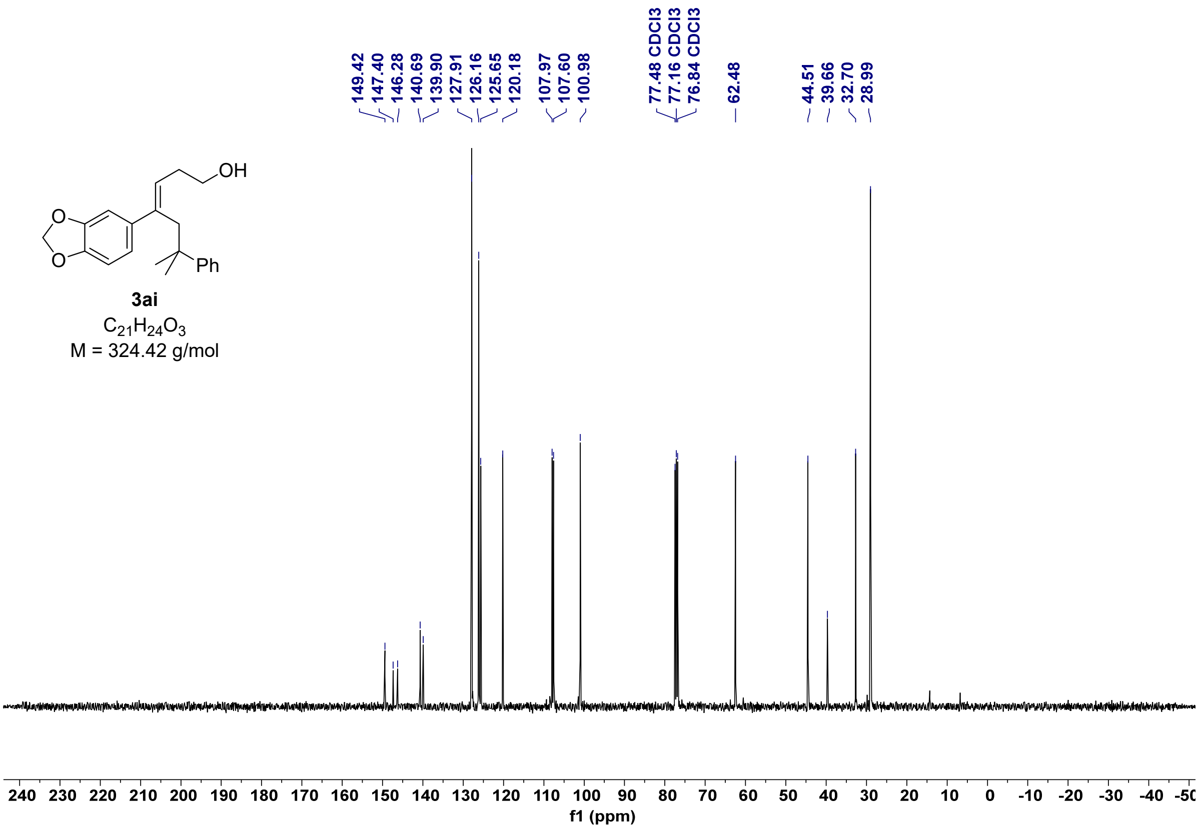

^{13}C{^{1}H} **NMR** spectrum (100 MHz, $CDCl_3$) of compound **3ai**.

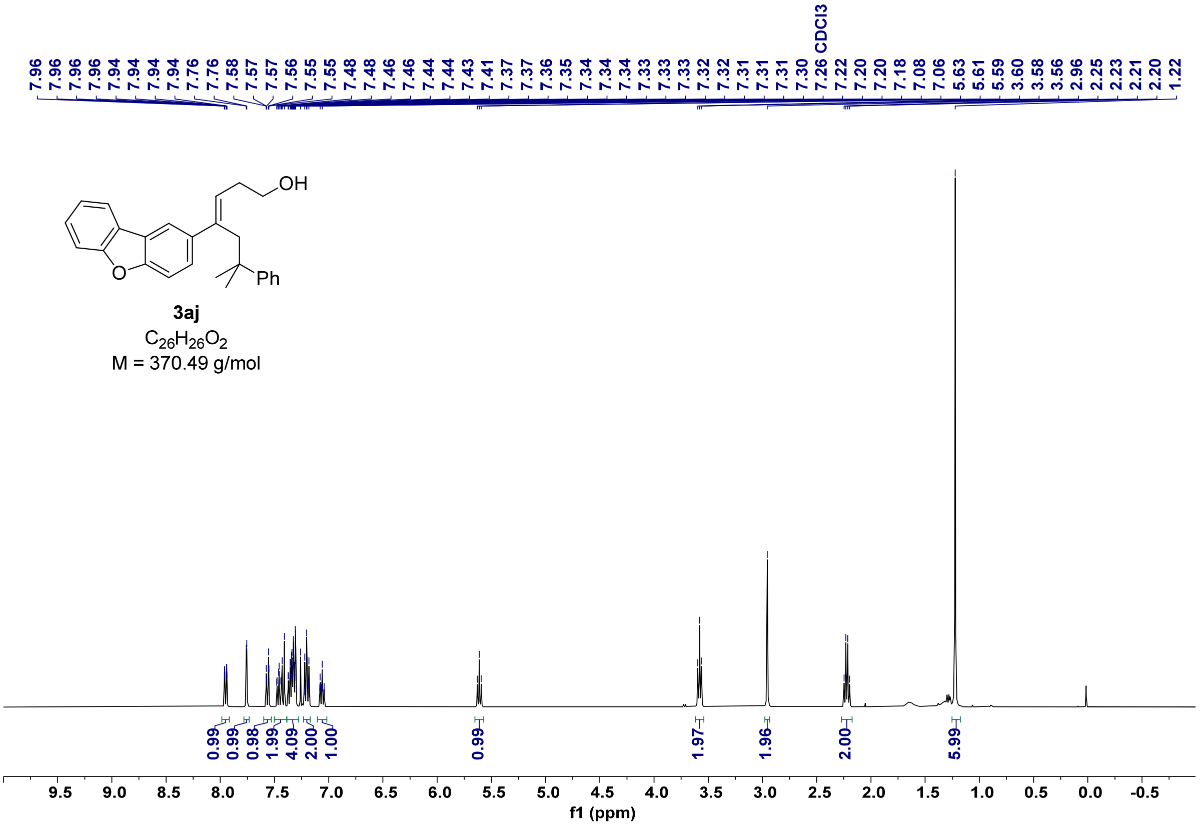

^{1}H **NMR** spectrum (400 MHz, $CDCl_3$) of compound **3aj**.

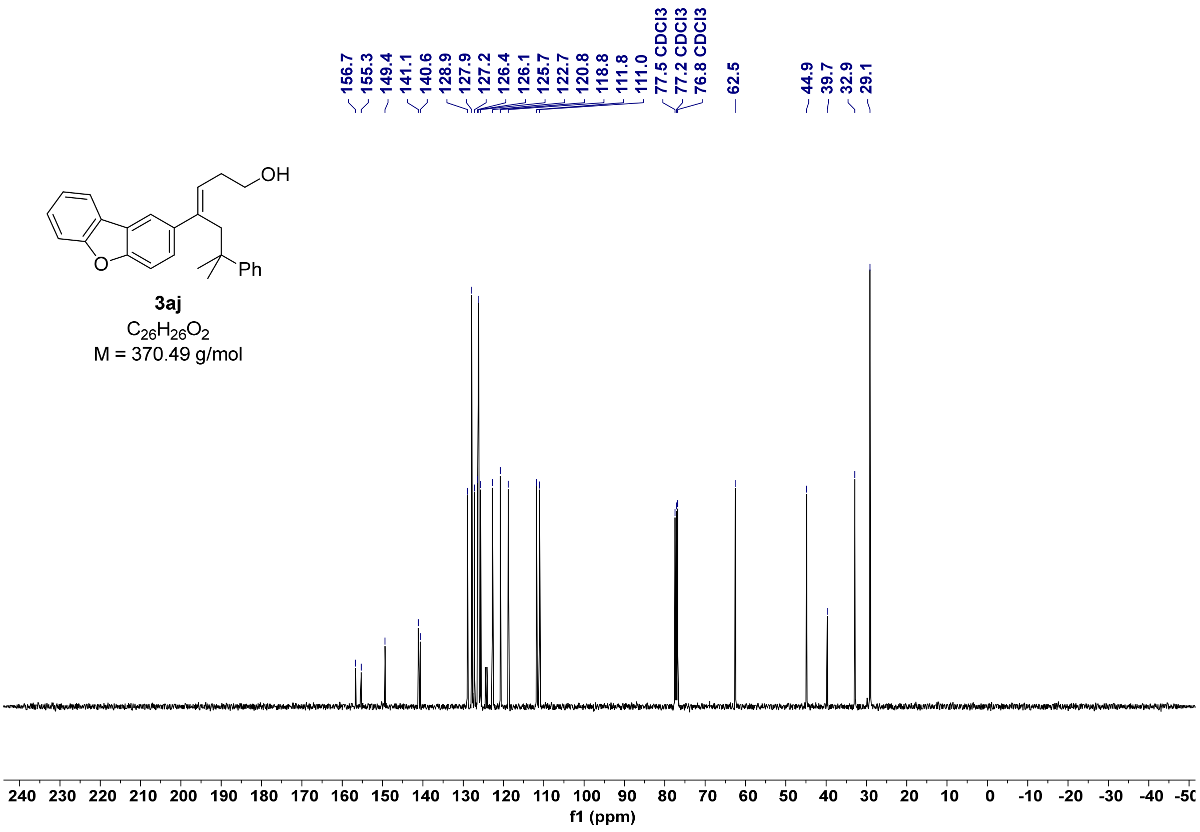

$^{13}C\{^1H\}$ **NMR** spectrum (100 MHz, $CDCl_3$) of compound **3aj**.

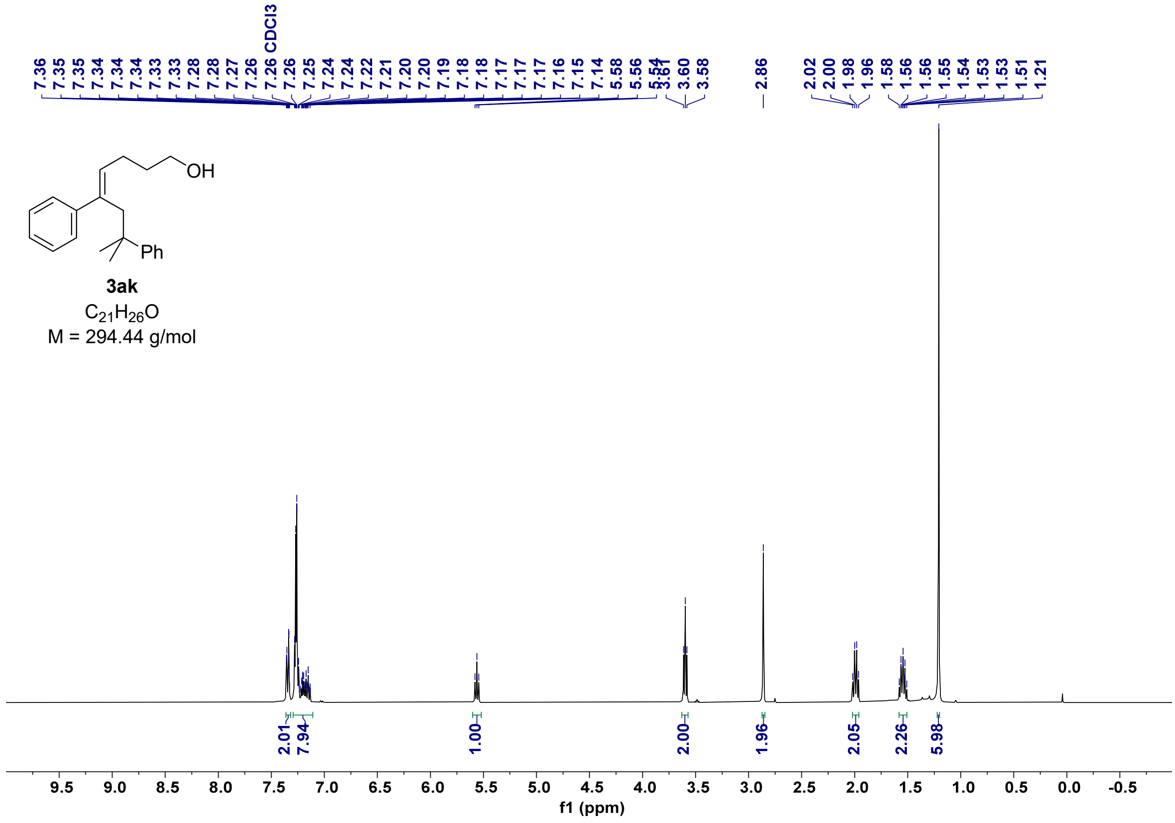

^1H **NMR** spectrum (400 MHz, $CDCl_3$) of compound **3ak**.

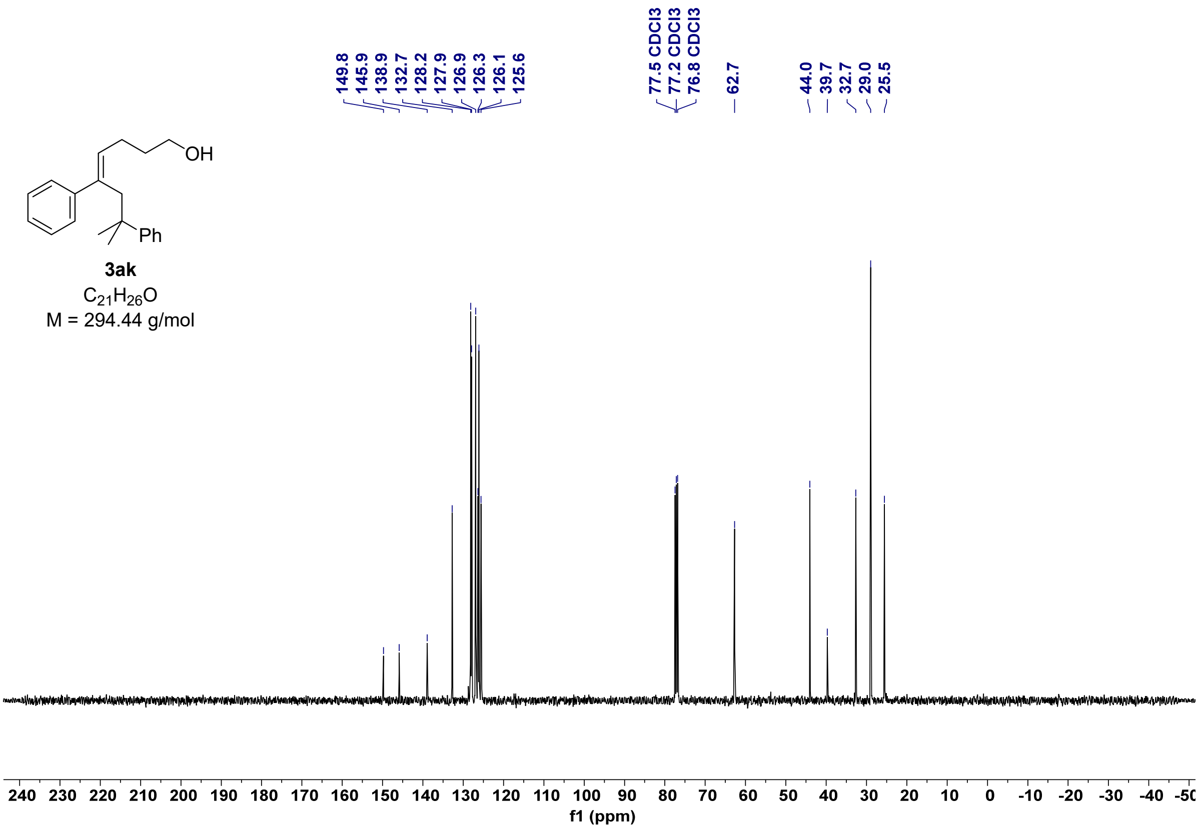

$^{13}C\{^1H\}$ NMR spectrum (100 MHz, $CDCl_3$) of compound **3ak**.

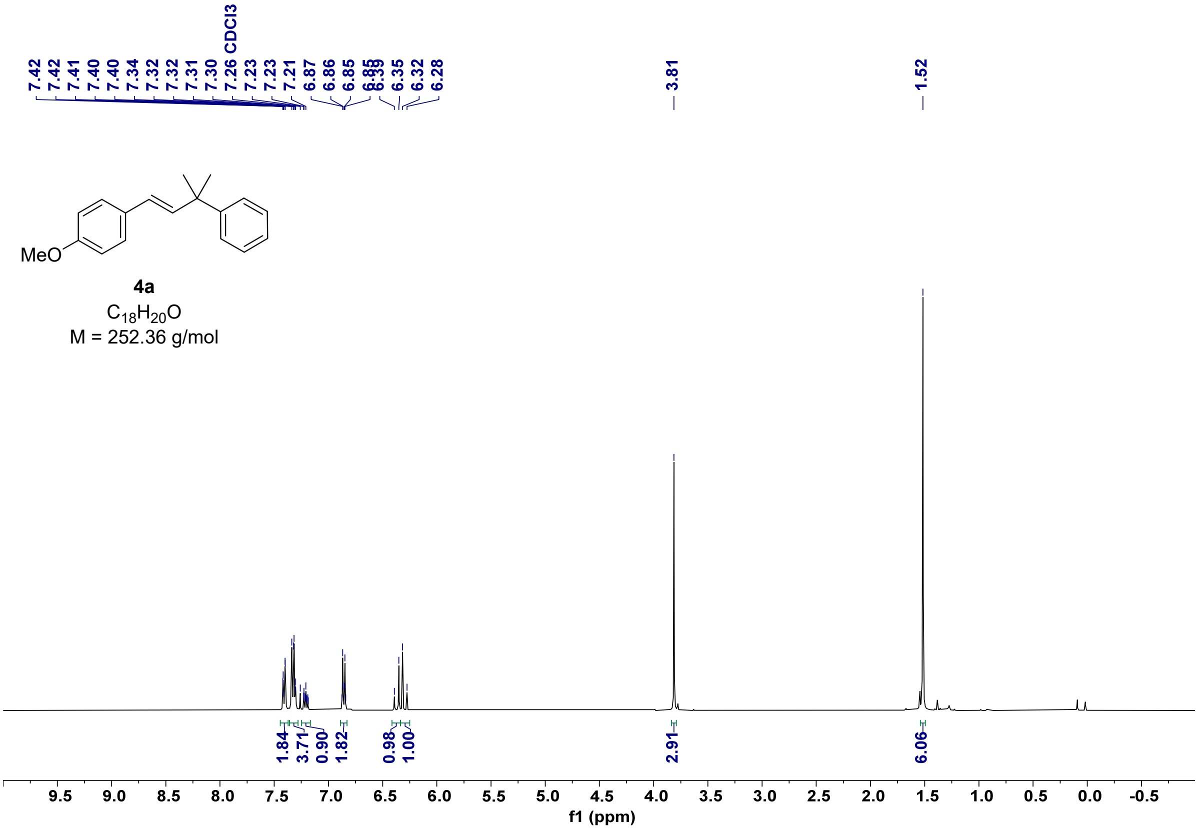

^1H NMR spectrum (400 MHz, $CDCl_3$) of compound **4a**.

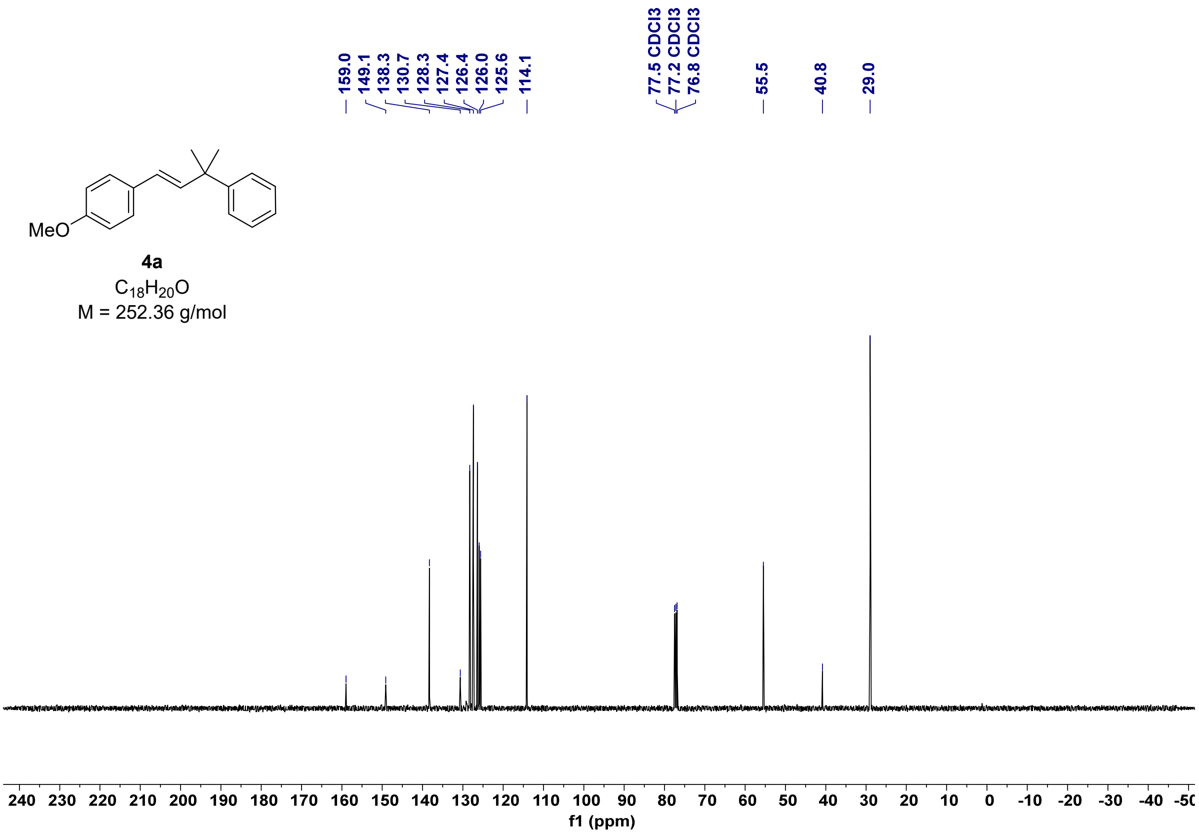

^{13}**C{^{1}H} NMR** spectrum (100 MHz, $CDCl_3$) of compound **4a**.

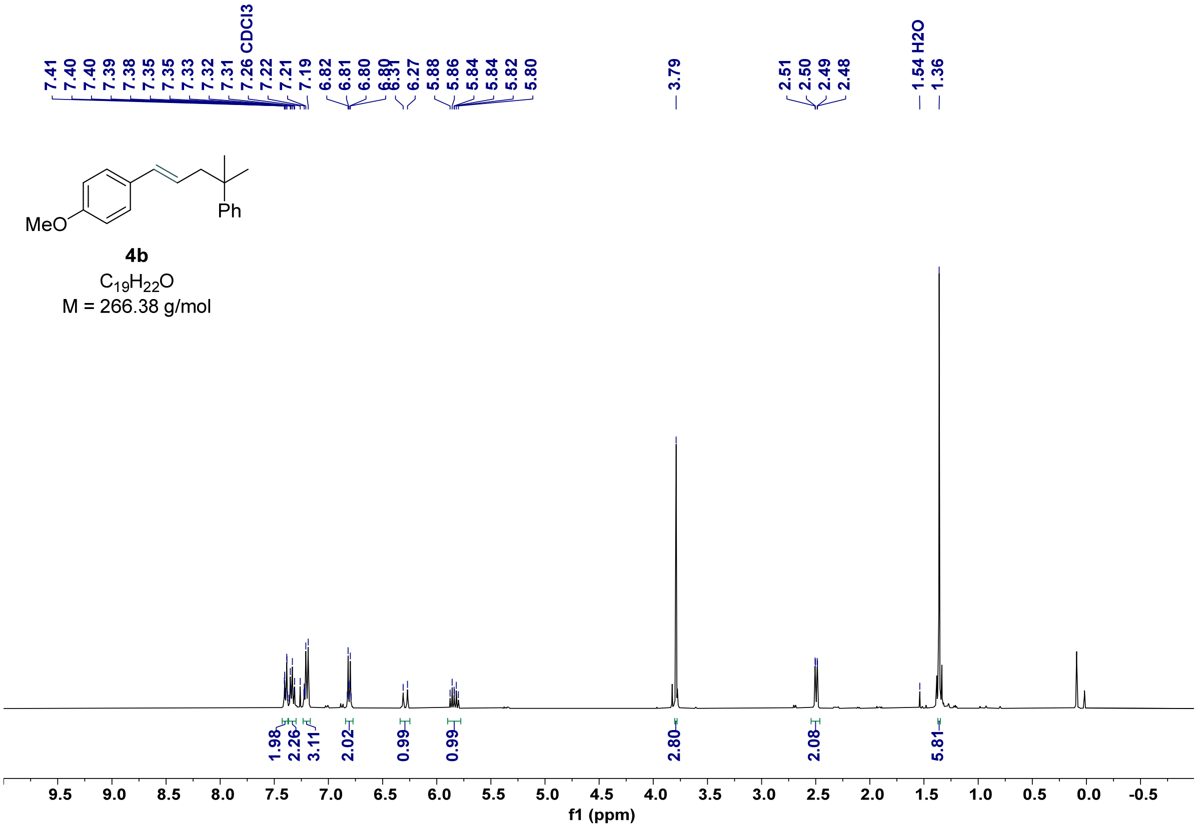

^{1}**H NMR** spectrum (400 MHz, $CDCl_3$) of compound **4b**.

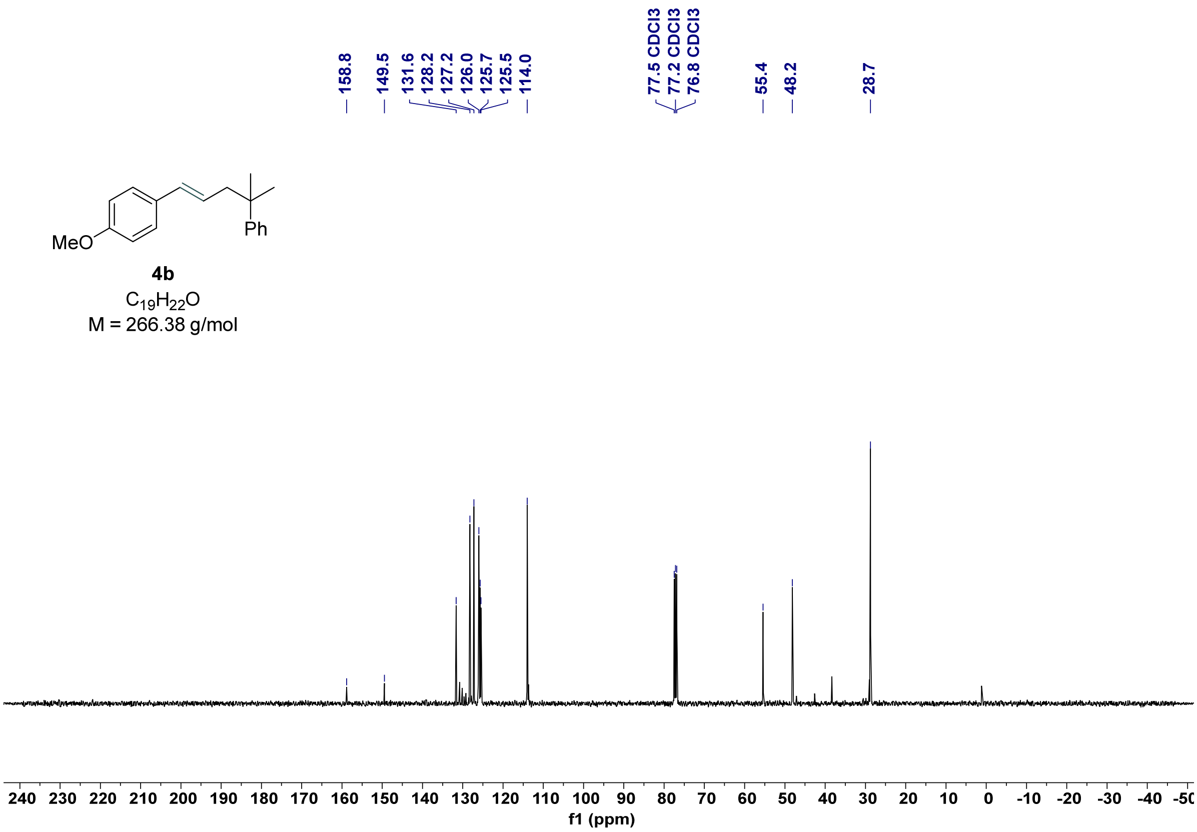

$^{13}C\{^1H\}$ NMR spectrum (100 MHz, $CDCl_3$) of compound **4b**.

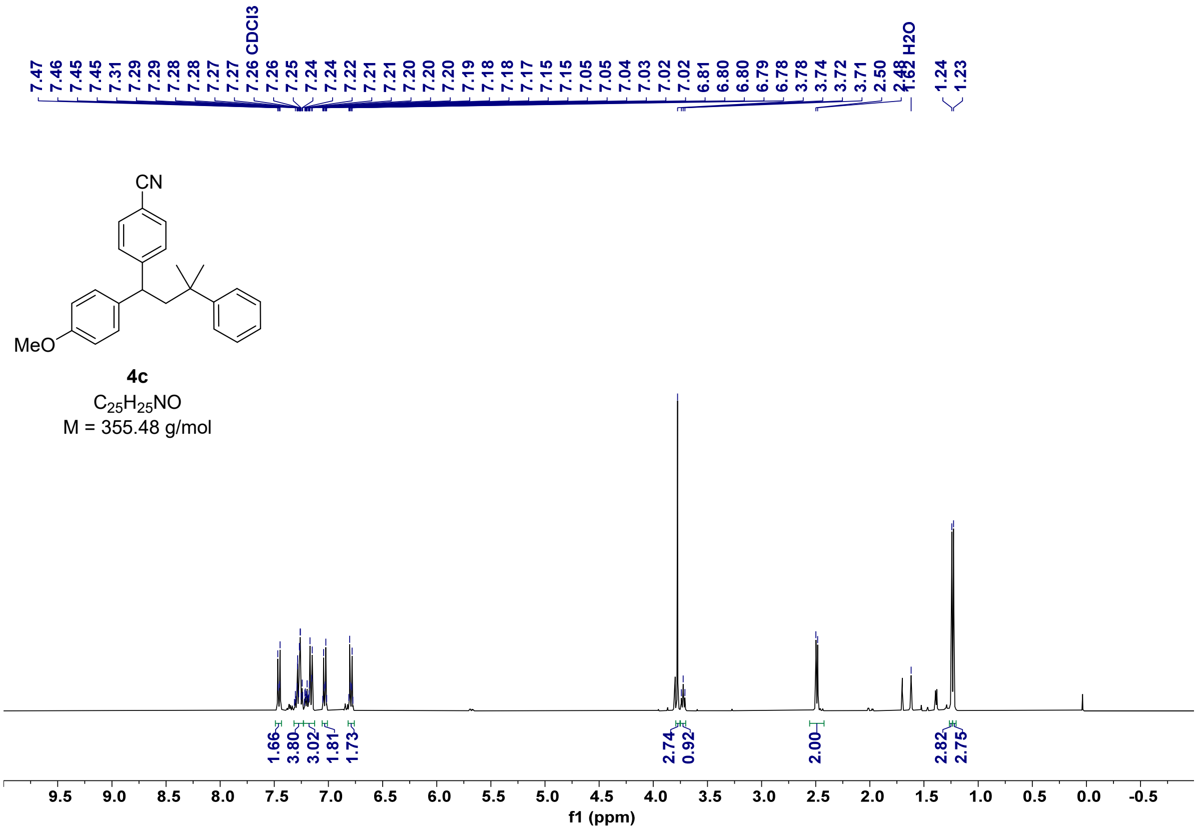

^1H NMR spectrum (400 MHz, $CDCl_3$) of compound **4c**.

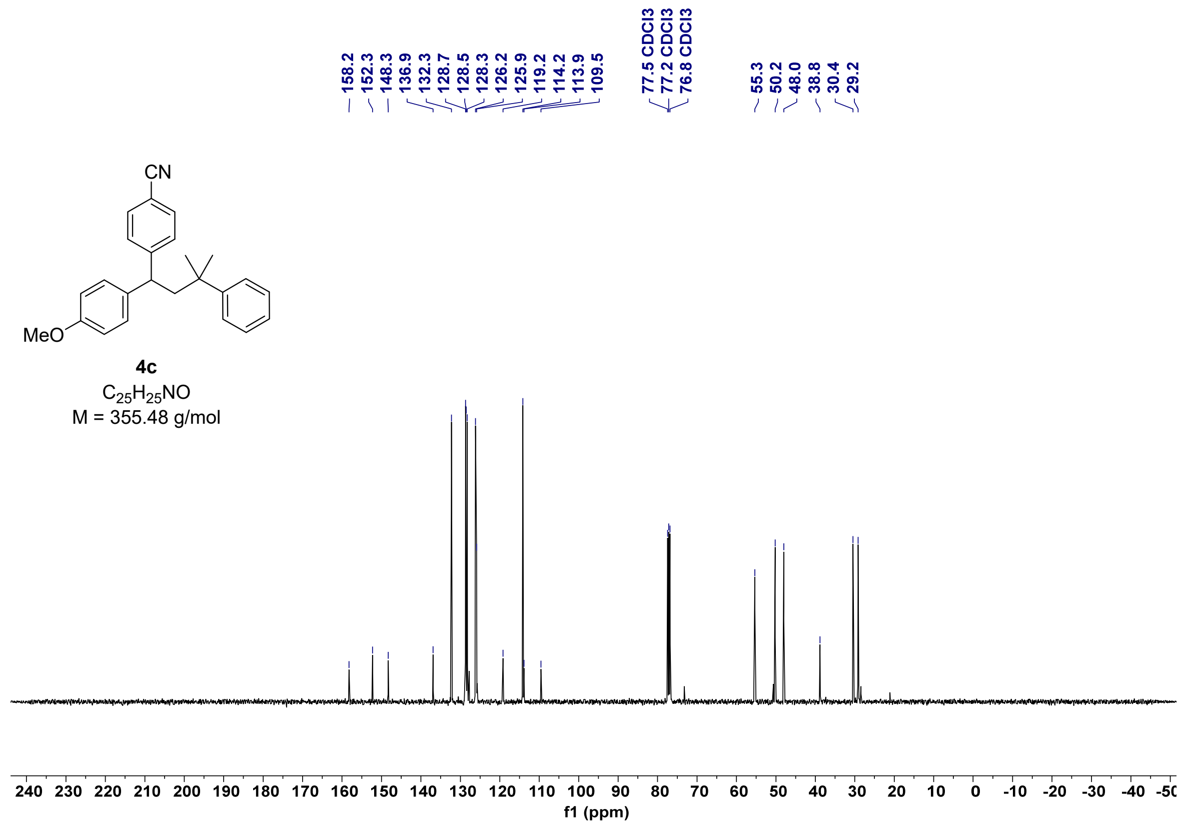

^{13}**C{^{1}H} NMR** spectrum (100 MHz, $CDCl_3$) of compound **4c**.

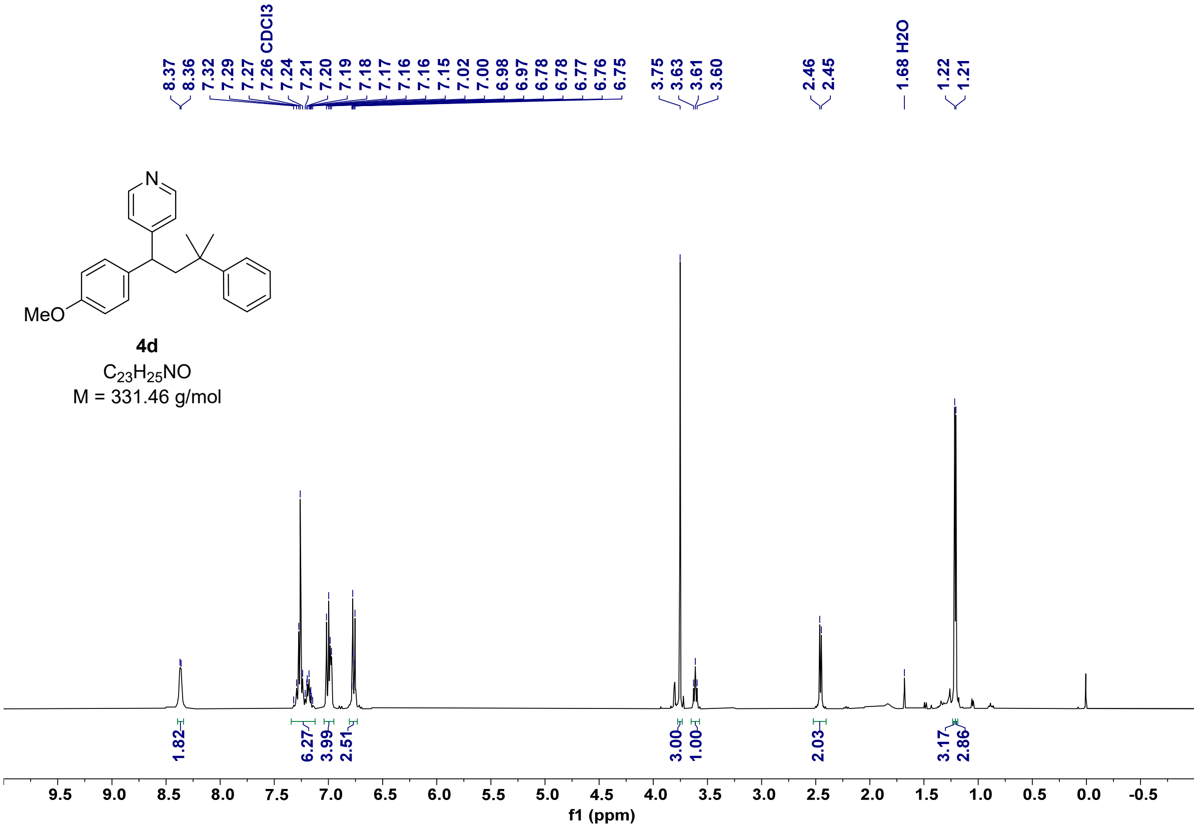

^{1}**H NMR** spectrum (400 MHz, $CDCl_3$) of compound **4d**.

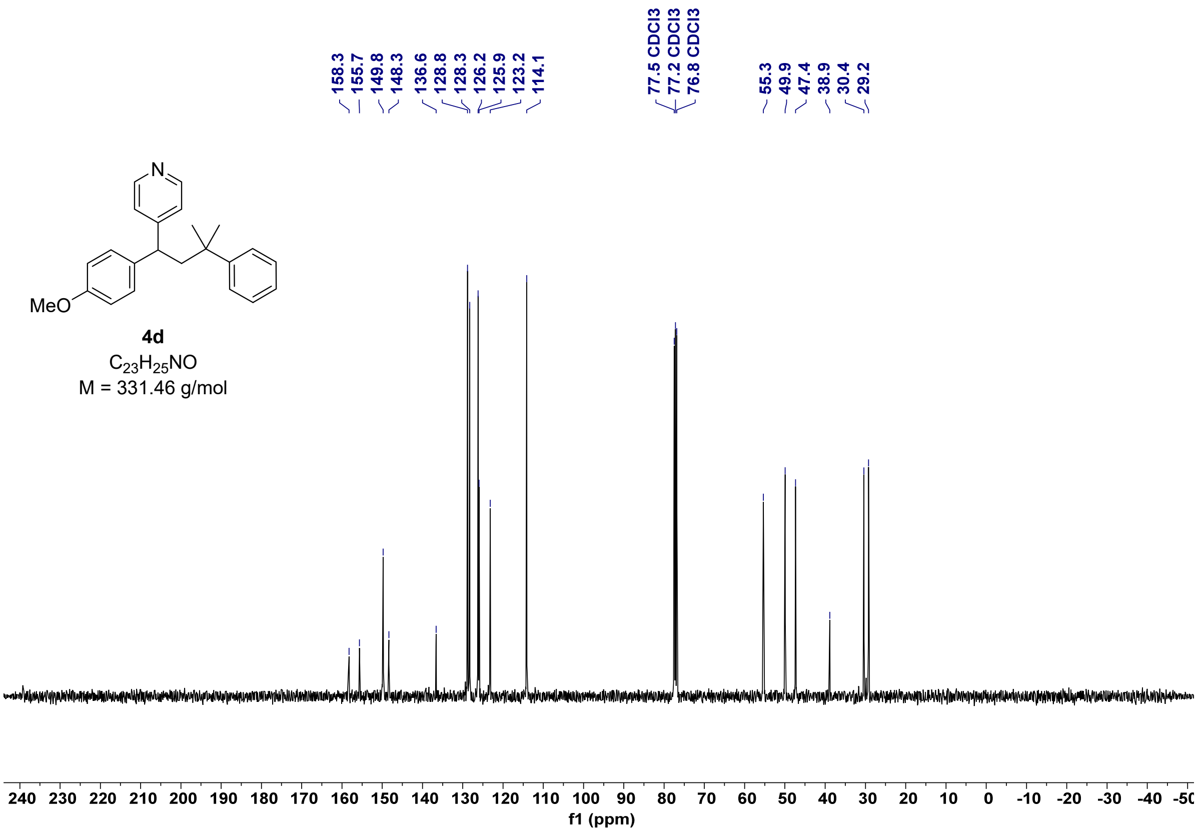

$^{13}C\{^{1}H\}$ NMR spectrum (100 MHz, $CDCl_3$) of compound **4d**.

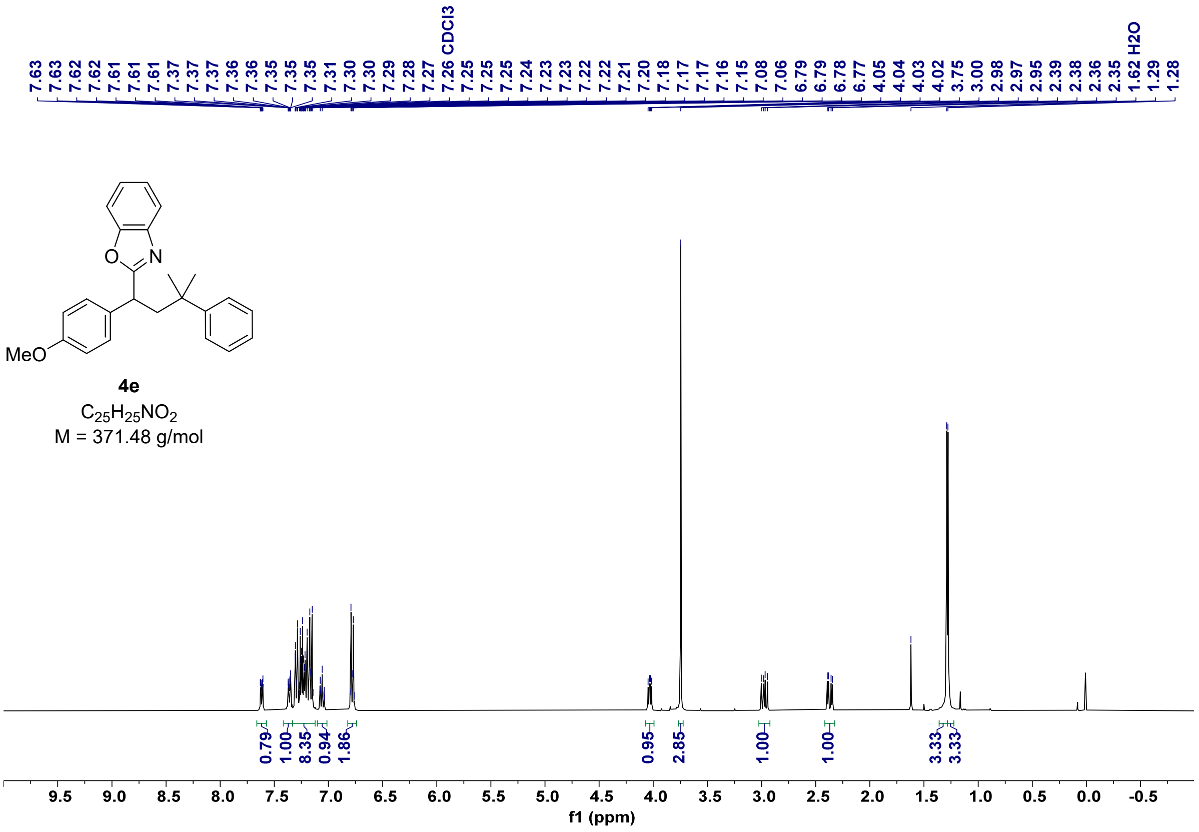

^{1}H NMR spectrum (400 MHz, $CDCl_3$) of compound **4e**.

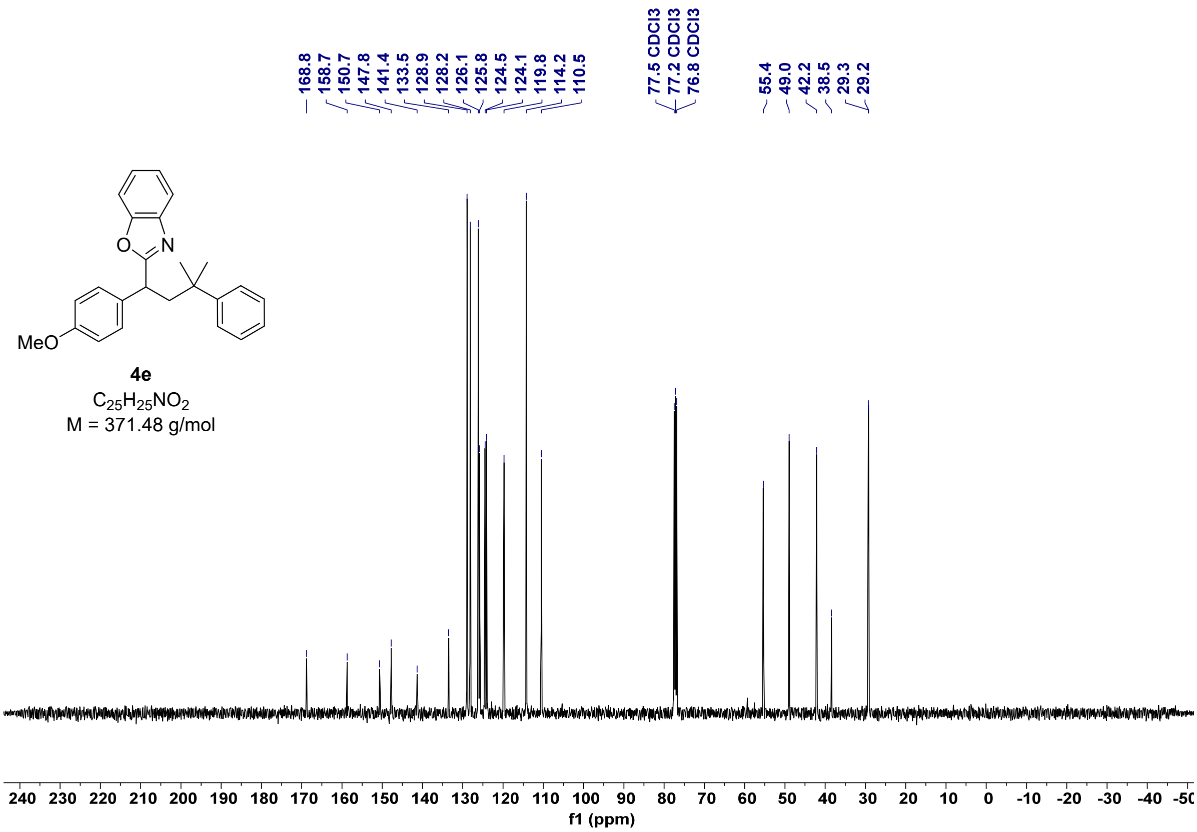

$^{13}C\{^{1}H\}$ NMR spectrum (100 MHz, $CDCl_3$) of compound **4e**.

9. Energies and Cartesian Coordinates of the Optimized Structures

Styrene
M06-2X/def2SVP Electronic E: -309.284391 a.u.
M06-2X/def2SVP Gibbs free E: -309.181006 a.u.
M06-2X/def2TZVPP Electronic E: -309.628578 a.u.

C	-1.7827010000	-1.0418740000	0.0000000000
C	-0.4093510000	-1.2847870000	0.0000000000
C	0.5121720000	-0.2271430000	0.0000000000
C	0.0178050000	1.0882380000	0.0000000000
C	-1.3522660000	1.3319070000	0.0000000000
C	-2.2592730000	0.2682320000	0.0000000000
H	-2.4820660000	-1.8799260000	0.0000000000
H	-0.0380920000	-2.3123550000	0.0000000000
H	0.7119260000	1.9306680000	0.0000000000
H	-1.7178690000	2.3604090000	0.0000000000
H	-3.3331040000	0.4631950000	0.0000000000
C	1.9560130000	-0.5384570000	0.0000000000
H	2.1935990000	-1.6074270000	0.0000010000
C	2.9624200000	0.3413330000	0.0000000000
H	2.7975050000	1.4218860000	-0.0000010000
H	3.9991860000	-0.0011440000	0.0000000000

2a
M06-2X/def2SVP Electronic E: -759.986658 a.u.
M06-2X/def2SVP Gibbs free E: -759.674599 a.u.
M06-2X/def2TZVPP Electronic E: -760.846341 a.u.

C	3.9228535723	-0.4068358001	1.1856571784
C	3.9330342392	-1.6177895727	0.4977297419
C	2.9727385025	-1.8482908272	-0.4896913369
C	2.0210462057	-0.8745364296	-0.7824094923
C	1.9996031561	0.3558492948	-0.1005981050
C	2.9657068412	0.5680148855	0.8916682107
H	4.6657674005	-0.2129593185	1.9620171586
H	4.6806805280	-2.3784926208	0.7290970822
H	2.9638172653	-2.7940571813	-1.0352220183
H	1.2693275918	-1.0718308782	-1.5506349048
H	2.9817090189	1.5045004584	1.4495536530
C	0.9361181383	1.3971331722	-0.4591771877
C	1.0247739262	2.6532489240	0.4107451301
H	1.9871970744	3.1715802505	0.2703294216
H	0.2249467792	3.3577562945	0.1364121860
H	0.9092124570	2.4221002220	1.4792178926
C	1.0692563835	1.7879317864	-1.9413063242
H	0.9612816433	0.9197783033	-2.6060996668
H	0.2921356564	2.5164929854	-2.2199788729
H	2.0523460153	2.2480559922	-2.1319476775
B	-0.4503762738	0.6710497686	-0.2108723490
O	-1.1120961501	-0.0467814722	-1.1709299428
O	-1.0923346027	0.6545932688	0.9964506676
C	-2.1436558641	-0.8068610465	-0.5030125953
C	-2.3831825112	0.0386081683	0.7950717549
C	-1.5592847435	-2.1840967661	-0.2064318212
C	-3.3502597589	-0.9245217527	-1.4155077822
C	-2.7466620158	-0.7753812749	2.0234846393
C	-3.3878025371	1.1668617647	0.5860790675
H	-1.2297433920	-2.6417041304	-1.1502865743
H	-0.6891370070	-2.1105742556	0.4633664095
H	-2.3052519877	-2.8444952551	0.2572908455
H	-3.0902813301	-1.5347042982	-2.2922463301
H	-4.1822074757	-1.4162684184	-0.8899841053
H	-3.6855141076	0.0588388789	-1.7682411390
H	-2.9029128849	-0.1015923775	2.8780191026
H	-3.6789307502	-1.3335679942	1.8518651896
H	-1.9514541402	-1.4841434900	2.2862550698
H	-4.4113682961	0.7793588594	0.4875678055
H	-3.3516908088	1.8389876240	1.4550360563
H	-3.1451707576	1.7526082581	-0.3129260375

KOtBu
M06-2X/def2SVP Electronic E: -832.656714 a.u.
M06-2X/def2SVP Gibbs free E: -832.566799 a.u.
M06-2X/def2TZVPP Electronic E: -833.010635 a.u.

C	1.0745425311	0.0005942291	0.0008195543
O	-0.2861797263	0.0008116761	0.0003566052
C	1.6225142467	-1.1197996982	0.9124467129
H	2.7246528770	-1.1617178320	0.9431358964
H	1.2513256558	-0.9694210751	1.9391584370
H	1.2465577317	-2.0934975612	0.5588389996
C	1.6221515140	-0.2295556647	-1.4247289429
H	1.2477280058	0.5640755152	-2.0914278553
H	2.7242160592	-0.2362393063	-1.4756167306
H	1.2499435283	-1.1935494033	-1.8079003867
C	1.6226558594	1.3506310882	0.5126494532
H	1.2489485077	2.1614239699	-0.1331872395
H	1.2495621295	1.5349149281	1.5331311072
H	2.7246092411	1.3990442401	0.5302165093
K	-2.5787871611	-0.0059541059	-0.0004321200

tBuOBpin-KOtBu
M06-2X/def2SVP Electronic E: -1476.369125 a.u.
M06-2X/def2SVP Gibbs free E: -1475.984348 a.u.
M06-2X/def2TZVPP Electronic E: -1477.452836 a.u.

B	0.1093800173	-0.0365981562	-0.0266608236
O	0.6978567046	-1.3493935222	0.3617222134
O	-0.7516358400	0.3955578730	1.1068227973
O	-0.7984071127	-0.1133971810	-1.1600483348
C	1.2807305198	-2.2429632751	-0.5647216300
C	-2.0747734157	-0.0115517443	0.8296869070
C	-2.1411908418	0.0599473131	-0.7489237723
C	-2.6419370715	1.4159509892	-1.2561128501
H	-2.5109135109	1.4523461029	-2.3482250438
H	-3.7079809745	1.5708435941	-1.0338256830
H	-2.0713720229	2.2457610315	-0.8184763798
C	-3.0142152474	-1.0278911049	-1.3710098337
H	-2.9936477703	-0.9273799020	-2.4668386939
H	-2.6551242626	-2.0324813336	-1.1152315376
H	-4.0604132912	-0.9351300371	-1.0398110789
C	-2.2877947405	-1.4337325118	1.3605556022
H	-3.3233889965	-1.7768076283	1.2198165292
H	-1.5998516786	-2.1345198391	0.8711854778
H	-2.0702529847	-1.4399041896	2.4403541082
C	-3.0470310026	0.9291498400	1.5326497190
H	-2.8404264458	1.9785414005	1.2840386291
H	-4.0890942881	0.7011634757	1.2606074800
H	-2.9490033764	0.8120423688	2.6228129618
C	2.3036004124	-3.0701837056	0.2164153164
H	1.8139481418	-3.5708022565	1.0661972460
H	2.7694405024	-3.8403359729	-0.4168720317
H	3.1035169773	-2.4196622395	0.6065886554
C	0.1973478755	-3.1663557466	-1.1324600786
H	0.6257363451	-3.9084337752	-1.8242174152
H	-0.3081616404	-3.7070537520	-0.3167913542
H	-0.5461588102	-2.5626576193	-1.6703454278
C	1.9834036908	-1.5229553273	-1.7210242826
H	1.2601936743	-0.9156178191	-2.2833762259
H	2.7685177441	-0.8540410692	-1.3436190224
H	2.4346908585	-2.2602474055	-2.4026621037
O	1.2813200520	0.8510729157	-0.1177395643
C	1.3335776766	2.1931517836	-0.5486791729
C	0.5109319018	3.1075607765	0.3663310510

C	2.8070968681	2.5979800296	-0.4585613853
C	0.8708831728	2.3282034144	-2.0020374313
H	0.8962343885	3.0638481509	1.3990685925
H	0.5705131948	4.1538134047	0.0292714888
H	-0.5396872850	2.7948737586	0.3938264743
H	2.9566052635	3.6410828041	-0.7753389882
H	3.1702556923	2.4981029887	0.5772870991
H	3.4179134499	1.9455252032	-1.1003467473
H	1.4709993631	1.6646108333	-2.6432245928
H	-0.1795457246	2.0350774480	-2.1074337145
H	0.9982299735	3.3629383790	-2.3558629329
K	1.4716068740	0.0201862347	2.4091497846

tBuOBpin
M06-2X/def2SVP Electronic E: -643.648098 a.u.
M06-2X/def2SVP Gibbs free E: -643.380607 a.u.
M06-2X/def2TZVPP Electronic E: -644.392348 a.u.

B	0.2770216902	-0.4817263026	-0.1601234355
O	-0.1640337685	0.7107022197	0.3701914200
O	-0.7554054653	-1.2967133605	-0.5578103672
C	-1.5726974350	0.8013360990	0.0797529018
C	-1.9717153155	-0.7082577918	-0.0636015092
C	-2.2719607890	1.5378740609	1.2072835244
C	-1.7143414147	1.5698662092	-1.2303840960
C	-2.2783549973	-1.3592035041	1.2817461685
C	-3.0949334896	-0.9728783412	-1.0497086619
H	-2.0447736950	1.0885644567	2.1821838411
H	-1.9391746609	2.5856184741	1.2254444890
H	-3.3614595704	1.5269183834	1.0555938707
H	-2.7696582624	1.7288299498	-1.4922593212
H	-1.2317866901	2.5515870500	-1.1208945147
H	-1.2238647628	1.0344010208	-2.0571325514
H	-3.2347127671	-1.0065620212	1.6924276520
H	-2.3381137125	-2.4477655976	1.1421154859
H	-1.4825859309	-1.1491088914	2.0118452803
H	-3.3170219637	-2.0494118230	-1.0739544220
H	-4.0085699760	-0.4409644324	-0.7454692994
H	-2.8206429571	-0.6569642071	-2.0640999362
O	1.5644471012	-0.8667602009	-0.3001427460
C	2.7016091810	-0.0420226860	0.0034425245
C	2.6976584254	0.3119095032	1.4882266671
H	3.6056860217	0.8758634886	1.7462675087
H	1.8212356809	0.9239500484	1.7402918372
H	2.6761596103	-0.6059154391	2.0947005941
C	2.6711083964	1.2125188913	-0.8660929585
H	3.5755892652	1.8144895014	-0.6970852929
H	2.6342102804	0.9334264951	-1.9298412528
H	1.7917934934	1.8270694387	-0.6300536973
C	3.9160368900	-0.8961386312	-0.3370250073
H	4.8455510719	-0.3449281045	-0.1361925753
H	3.9142428465	-1.8157881192	0.2659090989
H	3.8957216696	-1.1781098365	-1.3997152196

Prd
M06-2X/def2SVP Electronic E: -1069.311113 a.u.
M06-2X/def2SVP Gibbs free E: -1068.869061 a.u.
M06-2X/def2TZVPP Electronic E: -1070.509789 a.u.

C	-1.6521112102	3.5769595594	-1.5432737364
C	-2.6945597873	4.0925307027	-0.7740371359
C	-2.9296496064	3.5680142073	0.4982925555
C	-2.1287237693	2.5396443817	0.9910170609
C	-1.0727670108	2.0144060295	0.2296801667
C	-0.8504130841	2.5468386355	-1.0479474014
H	-1.4586451052	3.9779231581	-2.5402505501
H	-3.3197176936	4.8986190539	-1.1620813140
H	-3.7411613536	3.9636908197	1.1122993829
H	-2.3207498860	2.1299360758	1.9858384966
H	-0.0438182294	2.1525672551	-1.6686380615
C	-0.2661637555	0.8427066692	0.7655864934
H	-0.2595993637	0.9300675805	1.8629832321
C	1.1662642514	0.7713305498	0.2240858313
H	1.5984857187	1.7856839724	0.1995372993
H	1.1470957722	0.4135305193	-0.8186085484
C	2.1275285120	-0.1458488243	1.0233819719
C	3.4597698703	-0.1748221629	0.2635189677
C	1.5284161694	-1.5485909818	1.1596284579
C	2.3674798818	0.4150495581	2.4351953429
C	4.2273973365	0.9973658428	0.1579325048
C	3.9491932638	-1.3282358005	-0.3625638033
H	2.2331561969	-2.2347703765	1.6529869609
H	0.6223740120	-1.5147233827	1.7848502641
H	1.2542629834	-1.9719203797	0.1826810576
H	2.7137121329	1.4584127624	2.4081194577
H	1.4456752746	0.3822493580	3.0343480946
H	3.1273023970	-0.1835463225	2.9605155232
C	5.4327200974	1.0161983838	-0.5398235451
H	3.8777591156	1.9180523074	0.6298231404
C	5.1581837356	-1.3139192226	-1.0635609982
H	3.3884730686	-2.2614630039	-0.3115946295
C	5.9067791122	-0.1436555477	-1.1556262449
H	6.0062019798	1.9431679643	-0.6021238476
H	5.5134225150	-2.2299009042	-1.5399003169
H	6.8513827209	-0.1326545687	-1.7022641363
B	-1.1685578122	-0.3935513210	0.3767540485
O	-1.1188799756	-1.0070462356	-0.8446957274
O	-2.1798717295	-0.8680652819	1.1685591857
C	-2.0830474148	-2.0827672529	-0.8335151496
C	-3.0538477063	-1.6394530786	0.3152071308
C	-1.3314203815	-3.3680387820	-0.5048215588
C	-2.7298047347	-2.1835611628	-2.2032673525
C	-3.6586447949	-2.7799158526	1.1121214851
C	-4.1416769948	-0.6871087760	-0.1723260135
H	-0.5264145175	-3.5079321117	-1.2405525790
H	-0.8816545767	-3.3234054966	0.4977313948
H	-1.9981897547	-4.2405247823	-0.5507931153
H	-1.9814534781	-2.5090930509	-2.9399456312
H	-3.5434738246	-2.9237930564	-2.1908272512
H	-3.1338150228	-1.2167782644	-2.5286482221
H	-4.3250334531	-2.3742140587	1.8866873069
H	-4.2532373719	-3.4334149767	0.4567686389
H	-2.8847733863	-3.3815488781	1.6051755912
H	-4.8900713227	-1.2090877687	-0.7847422817
H	-4.6471262150	-0.2506358356	0.7010059897
H	-3.7118297954	0.1353671548	-0.7647838825

Int1
M06-2X/def2SVP Electronic E: -1592.712847 a.u.
M06-2X/def2SVP Gibbs free E: -1592.280665 a.u.
M06-2X/def2TZVPP Electronic E: -1593.912896 a.u.

C	-3.9956249116	0.2410760217	-0.0837864708
C	-2.8689993148	0.4107647167	0.7246556840
C	-1.9520138787	-0.6347115692	0.9569186854
C	-2.2624833092	-1.8806588211	0.3719901359
C	-3.3875198725	-2.0573518192	-0.4360012667
C	-4.2594904421	-0.9930905101	-0.6833094124
H	-4.6731395773	1.0826061281	-0.2449497577
H	-2.6875751003	1.3908969869	1.1633395052
H	-1.5916440606	-2.7252105749	0.5248014725
H	-3.5832806348	-3.0370358395	-0.8775278852
H	-5.1387005080	-1.1287748640	-1.3154614724
C	-0.6502893194	-0.4065899267	1.6860800947
B	0.4772540152	0.0268788923	0.5114303180
O	1.8388586129	0.1087993963	1.0068564532
O	0.5180108035	-1.0761736000	-0.4910220559
C	1.8484935935	-1.2827124947	-0.9155053330
C	2.7151491718	-0.7367109418	0.2942594851
C	2.0757266122	-0.5057759022	-2.2188030812
H	3.0992867499	-0.6168012931	-2.6047173133

H	1.3948990080	-0.8996529830	-2.9927138551
H	1.8658388192	0.5614790321	-2.0769326654
C	2.0529438144	-2.7680281946	-1.2009158522
H	1.7829178060	-3.3837644914	-0.3334042138
H	1.4123193552	-3.0706610540	-2.0435532150
H	3.0969949472	-2.9837065697	-1.4750022124
C	3.9374700211	0.0686108581	-0.1464358046
H	4.6344151198	-0.5435755975	-0.7396816585
H	3.6460877255	0.9448037459	-0.7412743621
H	4.4735022476	0.4274816605	0.7452702727
C	3.1899161794	-1.8480850532	1.2357048759
H	2.3534196263	-2.4767711026	1.5649385620
H	3.9510422302	-2.4905272145	0.7686390707
H	3.6323794204	-1.3791195522	2.1274195533
O	0.0160597931	1.2639655779	-0.2099539022
C	0.3896034391	2.6242542221	-0.1234858965
K	-1.3601888061	-0.0239751291	-1.9646395398
C	1.1195681234	3.0198200004	-1.4142774240
H	0.5219288146	2.7447162200	-2.2991201010
H	1.3031994453	4.1043589883	-1.4511222600
H	2.0922033658	2.5108186914	-1.4790331152
C	-0.9000198795	3.4485157530	-0.0220794306
H	-0.6896972383	4.5276727753	-0.0686380689
H	-1.5822422371	3.1956782771	-0.8507331874
H	-1.4209902162	3.2436302785	0.9249591012
C	1.2964424052	2.9631577485	1.0634435435
H	0.8292680544	2.6754521511	2.0142588012
H	2.2547043259	2.4367947020	0.9894000421
H	1.4765729292	4.0491416305	1.0826094899
C	-0.1612450977	-1.6842553039	2.3733639599
H	0.1129581842	-2.4604158892	1.6449626459
H	0.7423005614	-1.4601667773	2.9588250634
H	-0.9183536975	-2.1050004494	3.0595805203
C	-0.7754345911	0.6892203205	2.7461427417
H	0.1877082084	0.8130695704	3.2648186958
H	-1.0365839529	1.6652867314	2.3164179092
H	-1.5382688836	0.4419954412	3.5070221306

Int2
M06-2X/def2SVP Electronic E: -1592.690893 a.u.
M06-2X/def2SVP Gibbs free E: -1592.268276 a.u.
M06-2X/def2TZVPP Electronic E: -1593.894967 a.u.

C	-3.7779055008	1.1347998784	-0.0026081721
C	-2.9946385619	0.4081620336	0.8776624213
C	-2.6398573213	-0.9899672163	0.6374498391
C	-3.2915093381	-1.5616716000	-0.5400282511
C	-4.0725077155	-0.8036042055	-1.3953248085
C	-4.3058823091	0.5734701650	-1.1860283881
H	-3.9951390697	2.1812326716	0.2352482443
H	-2.6320559851	0.8969569225	1.7843061224
H	-3.1712794332	-2.6265456438	-0.7470618072
H	-4.5260133548	-1.2968319526	-2.2611101881
H	-4.9417064409	1.1518418048	-1.8561385319
C	-1.7763449810	-1.6980016765	1.4529713833
B	1.4813930574	0.6471709746	-0.3180205508
O	2.5534076872	0.4225150655	0.4934312711
O	1.1923183452	-0.4186051813	-1.1481793895
C	2.2850347432	-1.3592358430	-1.0154497761
C	2.8449629653	-0.9956302070	0.4036718069
C	3.2759081518	-1.0592742076	-2.1333766137
H	4.1088606162	-1.7755420644	-2.1274880424
H	2.7586430973	-1.1371044643	-3.1001301209
H	3.6863351668	-0.0428219662	-2.0402373079
C	1.7440553828	-2.7708112314	-1.1338099390
H	0.9090754402	-2.9370393854	-0.4414518426
H	1.3882354233	-2.9471872060	-2.1591903460
H	2.5370801632	-3.5016782310	-0.9176818606
C	4.3365943425	-1.2079122719	0.5690005067
H	4.5914243665	-2.2640117176	0.3977471030
H	4.9122581686	-0.5855593227	-0.1276701500
H	4.6336353975	-0.9455224744	1.5942910920
C	2.0626542908	-1.6785421416	1.5188677221
H	0.9788526703	-1.5597197148	1.3628705693
H	2.2947255051	-2.7515727820	1.5703234251
H	2.3309710559	-1.2163943406	2.4796867219
O	0.6848989613	1.7428824603	-0.3929198470
C	0.8059193318	2.9270842001	0.4300876603
K	-1.3037010976	0.4036550674	-1.6301933360
C	2.1779250991	3.5507568751	0.2026283086
H	2.3269429467	3.7631766094	-0.8664565290
H	2.2571903213	4.4957264716	0.7584444664
H	2.9748522755	2.8771001710	0.5470696912
C	-0.3021510512	3.8539189307	-0.0495102156
H	-0.2941623093	4.7897105507	0.5264361306
H	-0.1668918347	4.0976201571	-1.1136865515
H	-1.2844732556	3.3748869901	0.0862533470
C	0.5948417272	2.5458198789	1.8900425217
H	-0.3992686620	2.0932407638	2.0206819147
H	1.3598194410	1.8298027793	2.2219239972
H	0.6549670620	3.4414060559	2.5246519188
C	-1.4959092721	-3.1570941677	1.1973913822
H	-1.3147423105	-3.3747852583	0.1298976593
H	-0.6009791065	-3.4912931458	1.7473942583
H	-2.3203060105	-3.8357682870	1.5031119236
C	-1.2589221682	-1.0758615440	2.7267730871
H	-0.5582637848	-1.7516671690	3.2410908254
H	-0.7169257207	-0.1257181156	2.5593566464
H	-2.0596286076	-0.8367427421	3.4563765985

Int3
M06-2X/def2SVP Electronic E: -949.014456 a.u.
M06-2X/def2SVP Gibbs free E: -948.880294 a.u.
M06-2X/def2TZVPP Electronic E: -949.479789 a.u.

C	1.6805827538	0.8886922155	1.1610674940
C	0.2983994177	0.8229043602	1.1844233077
C	-0.4860133277	0.5953900128	-0.0289729114
C	0.2984509344	0.7035698180	-1.2588009488
C	1.6807426517	0.7712560235	-1.2418247417
C	2.4207359173	0.7954548207	-0.0386805929
H	2.2128901078	1.0233010364	2.1080098644
H	-0.2183244366	0.9199741672	2.1408492088
H	-0.2186515581	0.7072110377	-2.2199535740
H	2.2132190583	0.8132244073	-2.1972740781
H	3.5081937793	0.8664121998	-0.0421220408
C	-1.8209455392	0.2408168765	-0.0116039790
K	0.8703321051	-1.8537827401	0.0862089098
C	-2.5764621748	0.0450980708	-1.3021092772
H	-2.0373731555	-0.6060940308	-2.0154174208
H	-3.5559176578	-0.4228793873	-1.1194194757
H	-2.7763550537	0.9861669367	-1.8537844801
C	-2.5773930998	0.1754566296	1.2915049595
H	-3.5575946199	-0.3066748884	1.1555999450
H	-2.0399596861	-0.4025265783	2.0664920660
H	-2.7756534160	1.1672220125	1.7464657652

Int4
M06-2X/def2SVP Electronic E: -1258.354377 a.u.
M06-2X/def2SVP Gibbs free E: -1258.087347 a.u.
M06-2X/def2TZVPP Electronic E: -1259.158607 a.u.

C	2.9020629466	0.0399779982	1.4453663423
C	3.8669249221	0.2545217111	0.4423785788
C	3.7109279364	-0.4822803128	-0.7545112421
C	2.6127264351	-1.2885749526	-0.9811847452
C	1.5070871413	-1.3926147004	-0.0356114058
C	1.7856074814	-0.7575669500	1.2457782029
H	3.0285284099	0.5209989173	2.4205199676
H	4.7440282616	0.8768611660	0.6179694850
H	4.4805612878	-0.4156214333	-1.5297740934

H	2.5275292281	-1.8267279278	-1.9298640793
H	1.0729435779	-0.8709390040	2.0641127451
C	0.3000811914	-1.9676393617	-0.3937621538
H	0.2453011863	-2.4297532549	-1.3853678241
C	-0.9039880616	-2.1020101532	0.4925636130
H	-1.2486523629	-3.1534939512	0.5415829000
H	-0.6519386257	-1.8356350017	1.5310875169
C	-1.7742038564	2.4198047981	-0.9948811093
C	-2.0354887666	1.0448098102	-1.0448197798
C	-1.8295589079	0.2199310651	0.0720561947
C	-1.3403119884	0.8289991324	1.2423346333
C	-1.0762142529	2.1982370954	1.2990231296
C	-1.2943046673	3.0055117319	0.1775565762
H	-1.9539211595	3.0336197999	-1.8798321861
H	-2.4125644798	0.6209592977	-1.9754839039
H	-1.1679949696	0.2224867359	2.1336492905
H	-0.7002725839	2.6375667381	2.2250948783
H	-1.0966420843	4.0780375210	0.2207672615
C	-2.1557948947	-1.2763056610	0.0727989823
K	1.1622169867	1.3019378799	-0.7654953120
C	-3.2781771980	-1.5283756174	1.0924788232
H	-4.1850868784	-0.9594564744	0.8356232762
H	-3.5376655586	-2.5983359274	1.1083319900
H	-2.9712900091	-1.2418230786	2.1097266733
C	-2.6339857583	-1.7543888740	-1.2989153970
H	-2.8301294618	-2.8366995575	-1.2598897919
H	-3.5674664530	-1.2557661067	-1.6017500283
H	-1.8787320140	-1.5761230976	-2.0780810086

Int5
M06-2X/def2SVP Electronic E: -1902.029809 a.u.
M06-2X/def2SVP Gibbs free E: -1901.476145 a.u.
M06-2X/def2TZVPP Electronic E: -1903.572496 a.u.

C	1.0294360494	2.9943689784	1.8354192609
C	-0.1786218886	2.5457410427	2.4015985677
C	-0.1789250812	1.2382442386	2.9393943554
C	0.9152054091	0.4021704196	2.8343336723
C	2.1262544882	0.7774594573	2.1154598163
C	2.1416375861	2.1755910716	1.7066205728
H	1.1011729708	4.0262986071	1.4767297519
H	-1.0406970631	3.2047579217	2.5047921147
H	-1.0691480879	0.8719062706	3.4612165888
H	0.8671074640	-0.6030384315	3.2642576219
H	3.0420006756	2.5879610783	1.2476944010
C	3.1057544550	-0.1544050910	1.8165881000
H	2.9712900713	-1.1624470000	2.2241222985
C	4.3696522951	0.1117240791	1.0531842609
H	4.5162970580	1.1943339543	0.9120789500
H	5.2588058571	-0.2272422148	1.6204692895
C	2.2857678973	1.7767744037	-2.4295608795
C	3.2499316847	1.2916889292	-1.5450411202
C	3.3752777127	-0.0829445297	-1.2731691945
C	2.4835181948	-0.9509276164	-1.9211394463
C	1.5117973336	-0.4714241197	-2.8076342754
C	1.4087116033	0.8949528274	-3.0697682915
H	2.2189996772	2.8498521147	-2.6199268975
H	3.9261810423	1.9998512756	-1.0614860145
H	2.5410418642	-2.0246871111	-1.7437412887
H	0.8322987886	-1.1751588178	-3.2928962953
H	0.6544490592	1.2707524205	-3.7634882639
C	4.4849424849	-0.5759162648	-0.3400407935
K	0.3634570517	0.7075835500	-0.1310098472
C	4.4377255794	-2.0933352415	-0.1542210863
H	4.6038022164	-2.6250053665	-1.1037911486
H	5.2303833837	-2.4006406943	0.5450555309
H	3.4739899259	-2.4223785258	0.2616747235
C	5.8414000341	-0.2052868789	-0.9608792015
H	6.6603147254	-0.5578187728	-0.3145982610
H	5.9651492164	-0.6664143245	-1.9530116473
H	5.9477822144	0.8841239975	-1.0746175035
B	-2.4003990501	-0.7048682635	-0.2643958147
O	-3.6965065458	-1.0559477377	-0.0215182649
O	-2.2669602004	0.5771716768	-0.7590383457
C	-4.4752231641	0.1634014094	-0.1017080234
C	-3.6001872703	1.0543132643	-1.0525279086
C	-5.8560932829	-0.1615313563	-0.6366842265
C	-4.5598857146	0.7285058684	1.3116019087
C	-3.8638910006	0.7738175449	-2.5271046712
C	-3.6713830780	2.5420298733	-0.7652921527
H	-5.8016675405	-0.7185925383	-1.5802947489
H	-6.3992580795	-0.7747341118	0.0962304749
H	-6.4279185623	0.7633628637	-0.8015862126
H	-5.1874815684	1.6295569177	1.3449818548
H	-5.0039736275	-0.0295534747	1.9720485892
H	-3.5606550142	0.9824813067	1.6976727317
H	-4.8417078372	1.1663270455	-2.8381379978
H	-3.0852354627	1.2651032134	-3.1277351242
H	-3.8352126482	-0.3054664274	-2.7384761661
H	-3.0504120829	3.0884913817	-1.4896661940
H	-4.7065400005	2.9002614271	-0.8647227812
H	-3.3104650075	2.7742538722	0.2446329295
O	-1.2736761423	-1.4287457990	-0.0614436897
C	-1.1909497320	-2.6883927154	0.6419619811
C	-1.9608028531	-3.7451380029	-0.1404730854
H	-1.8491163498	-4.7266401240	0.3418316057
H	-3.0307050585	-3.4963227389	-0.1804324613
H	-1.5729632276	-3.8160913446	-1.1674097841
C	-1.7506899018	-2.5125014863	2.0490049167
H	-1.6322217431	-3.4439108141	2.6205528263
H	-1.2079975422	-1.7108741599	2.5719043208
H	-2.8191176034	-2.2574753907	2.0151633344
C	0.2970028597	-3.0086394236	0.6852086020
H	0.4758550000	-3.9432254004	1.2351841324
H	0.6908287656	-3.1220359500	-0.3357403540
H	0.8488293175	-2.1977670422	1.1886923779

Int5'
M06-2X/def2SVP Electronic E: -2018.374246 a.u.
M06-2X/def2SVP Gibbs free E: -2017.762660 a.u.
M06-2X/def2TZVPP Electronic E: -2020.029111 a.u.

C	1.7161434134	-2.8908665909	-2.4110607216
C	2.3130976415	-3.6676351598	-1.4133114813
C	1.8473150112	-3.5404707345	-0.1019204426
C	0.8276269793	-2.6355359596	0.2014973732
C	0.2411696851	-1.8032587191	-0.7741181182
C	0.7030367818	-1.9828652754	-2.0950622462
H	2.0418320740	-2.9916004484	-3.4488911609
H	3.1068321452	-4.3755524725	-1.6574101309
H	2.2730446859	-4.1571244322	0.6928252809
H	0.4691862090	-2.5778348482	1.2298686788
H	0.2638728379	-1.3957471647	-2.8992565699
C	-0.7744287886	-0.7605617085	-0.3960960235
H	-1.2020416747	-1.0967733867	0.5556083900
C	-1.9592919949	-0.6551560192	-1.3774711176
H	-2.1808238723	0.4024926812	-1.5747321085
H	-1.7209195091	-1.1075917112	-2.3548444170
C	-4.1460274430	1.2028641286	1.8439537563
C	-3.7537309002	0.6357681587	0.6333821698
C	-3.6751043787	-0.7578829382	0.4716938236
C	-3.9924058985	-1.5581223411	1.5764916470
C	-4.3822017153	-0.9927364707	2.7946471455
C	-4.4643038539	0.3901296222	2.9345227424
H	-4.1912477244	2.2898289617	1.9401090648
H	-3.4657250864	1.2928695960	-0.1887798895
H	-3.9348461231	-2.6443634124	1.5014901644
H	-4.6205399032	-1.6438820813	3.6382454874
H	-4.7664770063	0.8332085535	3.8852485559
C	-3.2555372436	-1.3420891504	-0.8823985491

C	-3.0308111028	-2.8547331353	-0.8087352704
H	-3.9556497886	-3.3942942395	-0.5534613201
H	-2.6891173142	-3.2234157927	-1.7882799834
H	-2.2642066443	-3.1196001751	-0.0644310164
C	-4.3742628895	-1.0631513952	-1.8987965925
H	-4.1015034306	-1.4642753410	-2.8880514527
H	-5.3202503647	-1.5326845583	-1.5870638391
H	-4.5501807963	0.0179948080	-2.0066805238
B	-0.0386353145	0.7563756304	-0.0280676704
O	1.1594809023	1.0079484335	-0.8921991447
O	-0.9318088235	1.8651691306	-0.3902820216
C	0.8573624006	1.9348497706	-1.9184618655
C	-0.3601101108	2.7462989677	-1.3303538829
C	2.1017275287	2.7746936502	-2.2034453037
C	0.4842900107	1.1960379750	-3.2059746191
C	0.0869930793	4.0495661269	-0.6553326903
C	-1.4228612723	3.1167168478	-2.3668163395
H	2.5069664729	3.2254309225	-1.2888228574
H	2.8848259800	2.1372510315	-2.6464282559
H	1.8861259320	3.5775355088	-2.9247302742
H	0.3070541430	1.8977672963	-4.0340211294
H	1.3117897227	0.5308212601	-3.4971592542
H	-0.4199432714	0.5897906855	-3.0673794141
H	0.3465435748	4.8143784425	-1.4026617259
H	-0.7399182726	4.4327301372	-0.0395507253
H	0.9599527070	3.9064044586	-0.0079959946
H	-2.2216629203	3.6886266060	-1.8701834990
H	-1.0049808781	3.7456505339	-3.1679031321
H	-1.8772952138	2.2268645603	-2.8212704792
C	0.3846332467	0.8388018744	1.6606480140
C	1.8654055169	0.5782205483	1.8428897890
C	-0.4574869605	-0.0890724133	2.5505289610
C	0.0599794233	2.2511150448	2.1830026449
C	2.8192307974	1.5376653610	1.4325821924
C	2.3800940482	-0.6120865125	2.3902552094
H	-0.2305268916	0.0791729975	3.6189185227
H	-1.5290030079	0.1224037916	2.4033735520
H	-0.3198600681	-1.1596916307	2.3583935677
H	0.6007483182	3.0464830925	1.6632868921
H	-1.0116192953	2.4573567460	2.0514540848
H	0.3023367029	2.3316387488	3.2581998634
C	4.1906624414	1.3073664492	1.5248231677
H	2.4723593153	2.4778868838	1.0046120916
C	3.7560492340	-0.8559710426	2.4774973807
H	1.6984825813	-1.3796492253	2.7551486719
C	4.6745511729	0.0975439463	2.0381154663
H	4.8898115396	2.0769582017	1.1900246461
H	4.1075551272	-1.7989152539	2.9021038505
H	5.7477136728	-0.0872816124	2.1100947111
K	3.1133256914	-0.6491098191	-0.6812383053

Int5''

M06-2X/def2SVP Electronic E: -1567.679517 a.u.
M06-2X/def2SVP Gibbs free E: -1567.281283 a.u.
M06-2X/def2TZVPP Electronic E: -1568.821739 a.u.

C	-0.1499549845	2.0533783467	0.4450167993
C	-0.1205860553	2.6147056693	-0.8373443973
C	-0.1228132407	1.7740537746	-1.9529793202
C	-0.1715783943	0.3857191909	-1.7834196624
C	-0.2297258044	-0.1904337715	-0.5068754089
C	-0.1957730668	0.6669776469	0.6049016255
H	-0.1460782581	2.7003528315	1.3246237534
H	-0.0980503630	3.6987892951	-0.9631640498
H	-0.0938723802	2.1976262815	-2.9590153046
H	-0.1812031174	-0.2655327841	-2.6612433281
H	-0.2324741285	0.2442094327	1.6121728200
C	-0.3589202609	-1.6906004505	-0.3319084402
H	-0.3816754608	-2.1296450618	-1.3428586764
C	-1.6428194560	-2.1093456799	0.4058443906
H	-1.6510251909	-3.2125290590	0.4178203898
H	-1.5658809267	-1.7999780025	1.4624689115
C	-3.7101740812	2.1473938762	-0.3732583309
C	-3.4743630182	0.8213796400	-0.7471363579
C	-3.2881071989	-0.1804911978	0.2125496703
C	-3.3677716532	0.1925319982	1.5645019828
C	-3.6050919037	1.5114505993	1.9429084417
C	-3.7736552412	2.5010658104	0.9723466793
H	-3.8412027985	2.9077683640	-1.1461681706
H	-3.4241720143	0.5799689123	-1.8092713409
H	-3.2310572357	-0.5616554785	2.3430340020
H	-3.6578471280	1.7687893792	3.0027802124
H	-3.9565613962	3.5368120927	1.2643038800
C	-3.0125081197	-1.6456630673	-0.1523761523
C	-4.0997801380	-2.5158900155	0.5047325470
H	-5.1038557835	-2.1784974208	0.2056998032
H	-3.9822765309	-3.5659577362	0.1957262485
H	-4.0403033632	-2.4810209715	1.6024276642
C	-3.0719583707	-1.8865958259	-1.6625989392
H	-2.8412311962	-2.9414662116	-1.8770131862
H	-4.0775128893	-1.6738645644	-2.0565048954
H	-2.3530802511	-1.2665158195	-2.2159427156
C	3.2283186561	-1.3000457645	0.2233146470
C	3.1940381633	-0.5581770711	1.4786933541
C	4.4621707983	-1.1000933467	-0.5300219856
C	4.1585692340	0.3828919095	1.8048119631
H	2.3635805690	-0.7133759679	2.1695813190
C	5.4013560953	-0.1501818041	-0.1799043047
H	4.6110717646	-1.6949689010	-1.4359387231
C	5.2571669421	0.6622401318	0.9689864015
H	4.0513863221	0.9295094239	2.7471751902
H	6.2830933707	-0.0271850988	-0.8165386067
H	6.0128835033	1.3968510463	1.2457173734
C	2.1867251691	-2.0579596532	-0.2772744445
C	0.8679227925	-2.2909427767	0.3988023769
H	0.6604910115	-3.3721071010	0.5128683398
H	0.8780201350	-1.8897737009	1.4253989327
H	2.3443404839	-2.5619220106	-1.2380118377
K	2.7130463891	1.3023016618	-0.7415261399

Int6

M06-2X/def2SVP Electronic E: -1902.042491 a.u.
M06-2X/def2SVP Gibbs free E: -1901.482772 a.u.
M06-2X/def2TZVPP Electronic E: -1903.580916 a.u.

C	-2.1940152730	-3.5478445506	-0.3878553608
C	-2.3158956688	-3.6002711714	-1.7805855362
C	-1.5211921775	-2.7523397759	-2.5583124458
C	-0.6392529128	-1.8574927443	-1.9490243617
C	-0.5215589729	-1.7658065820	-0.5471201748
C	-1.3129573343	-2.6484808262	0.2156370520
H	-2.7977706082	-4.2105647352	0.2362740495
H	-3.0063613064	-4.3018342685	-2.2517242665
H	-1.5884448737	-2.7876866537	-3.6479197529
H	-0.0360251734	-1.1890358062	-2.5681907439
H	-1.2602222442	-2.6060259850	1.3041420799
C	0.2848929551	-0.6669033873	0.0659401439
H	1.0051052071	-0.3238534804	-0.6930541645
C	1.0319083438	-0.9927436350	1.3578126604
H	1.2605262563	-0.0383772778	1.8623465371
H	0.3751183020	-1.5481968280	2.0494106804
C	4.9154690753	0.9682641476	0.3766186661
C	4.0438525570	0.1381529473	1.0805334819
C	3.3675557446	-0.9195988271	0.4517887683
C	3.6018540362	-1.1079224038	-0.9186752365
C	4.4662623538	-0.2734953945	-1.6309868459
C	5.1292112074	0.7700098004	-0.9873840850
H	5.4254117776	1.7807528204	0.8984006629
H	3.8848343010	0.3294468732	2.1428772565
H	3.1005704524	-1.9154394657	-1.4531019423

H	4.6226938519	-0.4462992669	-2.6977993260
H	5.8078547170	1.4205916186	-1.5420326626
C	2.3680368182	-1.7780039106	1.2346636178
K	-3.2681801196	-0.5331541628	-0.9541910529
C	2.1471941304	-3.1367885719	0.5607705469
H	3.0948088514	-3.6940831770	0.4847558864
H	1.4455755133	-3.7370935893	1.1603011855
H	1.7193688964	-3.0512242267	-0.4461121804
C	2.8890286517	-2.0479029440	2.6550334967
H	2.2132146194	-2.7483483606	3.1690980296
H	3.8948546924	-2.4960085804	2.6357431200
H	2.9307038467	-1.1331328166	3.2629967845
B	-0.7439179090	0.6441357393	0.3070064384
O	-0.0662194298	1.8323200666	0.8052233855
O	-1.2921023483	1.0842881135	-1.0154471900
C	0.2973556868	2.6850389003	-0.2561669458
C	-0.6884868772	2.2847477072	-1.4459360473
C	0.1386100639	4.1336392162	0.2072330625
C	1.7816185265	2.4709090605	-0.5848048820
C	-1.8026610279	3.3075147606	-1.6848408267
C	0.0341919522	2.0397212340	-2.7717800223
H	-0.8878631755	4.3543634582	0.5265426852
H	0.8046070267	4.3075074596	1.0654361232
H	0.4156619606	4.8416091503	-0.5884059330
H	2.1271811175	3.1293233078	-1.3954733882
H	2.3671437560	2.7020357641	0.3173607767
H	2.0018974633	1.4315859048	-0.8625825566
H	-1.4151557920	4.2732135731	-2.0407708048
H	-2.4856463503	2.9123028312	-2.4532079155
H	-2.3835022333	3.4772449633	-0.7686767604
H	-0.6974971961	1.7150382845	-3.5277388702
H	0.5235210132	2.9533482746	-3.1421718604
H	0.7955830977	1.2537212038	-2.6750866972
O	-1.8408384297	0.1504418722	1.1604008090
C	-2.4612009115	0.8457104589	2.2218674527
C	-3.0221130136	2.1937369312	1.7600979415
H	-3.5682805138	2.6913454047	2.5762753623
H	-2.2031405936	2.8442638707	1.4291874880
H	-3.7222572941	2.0556140538	0.9192047228
C	-1.4745324530	1.0498656714	3.3751916757
H	-1.9659990717	1.5335243480	4.2336156701
H	-1.0817004438	0.0755487922	3.7045035418
H	-0.6352724412	1.6726238838	3.0404499426
C	-3.6094896160	-0.0486032274	2.6956681683
H	-4.1207800491	0.3851768158	3.5678086029
H	-4.3640708775	-0.1795316952	1.9013346594
H	-3.2247281100	-1.0415169558	2.9726686245

TS1_2
M06-2X/def2SVP Electronic E: -1592.678833 a.u.
M06-2X/def2SVP Gibbs free E: -1592.251210 a.u.
M06-2X/def2TZVPP Electronic E: -1593.881001 a.u.

C	-3.9794531184	0.2578376292	-0.0897172884
C	-2.9391172858	0.1746815600	0.8221166737
C	-2.0393532369	-0.9586566597	0.8746032231
C	-2.3820114620	-2.0233624271	-0.0474490988
C	-3.4351801566	-1.9205620623	-0.9468045752
C	-4.2439339649	-0.7713256289	-1.0169917839
H	-4.6167450993	1.1474147443	-0.0789028245
H	-2.7948858606	0.9953463231	1.5257522910
H	-1.7930210766	-2.9414761756	-0.0400929505
H	-3.6374527128	-2.7621066953	-1.6163953178
H	-5.0801381732	-0.7064412008	-1.7137014809
C	-0.9393288090	-1.0382915674	1.7407880288
B	0.7576926881	0.2753571613	0.2011437895
O	1.9812257732	0.3597873763	0.8325227640
O	0.7672253204	-0.7137619716	-0.7936202183
C	2.1341207837	-1.0814991261	-1.0421416925
C	2.8834321731	-0.6045199851	0.2688731550
C	2.5758396570	-0.3220949935	-2.2923017008
H	3.6116745948	-0.5610324434	-2.5702223490
H	1.9229424231	-0.6087754874	-3.1305460212
H	2.4949527895	0.7645727102	-2.1467268382
C	2.2173211060	-2.5770010200	-1.2979270739
H	1.7930677375	-3.1547217620	-0.4675046653
H	1.6580384110	-2.8247761241	-2.2119647702
H	3.2631962329	-2.8848121415	-1.4436665366
C	4.2136033346	0.0878785736	0.0014044275
H	4.9166736066	-0.6005597244	-0.4900428980
H	4.0951101617	0.9783620955	-0.6289229193
H	4.6554582663	0.4013617341	0.9581699590
C	3.0987307916	-1.7157180197	1.2876796337
H	2.1682047934	-2.2538773378	1.4920461318
H	3.8550602949	-2.4330872857	0.9394753981
H	3.4522893326	-1.2669143381	2.2272353311
O	-0.1212234125	1.3374902261	0.1011650914
C	0.2369269942	2.7238335655	0.2408239047
K	-1.4877118454	0.1988003418	-1.8684425673
C	1.3575826559	3.0586357179	-0.7425403389
H	1.0618042752	2.7887225280	-1.7688511404
H	1.5753312248	4.1362388224	-0.7223504560
H	2.2758940876	2.5138695184	-0.4813241877
C	-1.0261825407	3.5004261914	-0.1135307075
H	-0.8667386713	4.5798077821	0.0204387016
H	-1.3120807472	3.3304360362	-1.1643708968
H	-1.8581003720	3.1840009141	0.5321425932
C	0.6661441541	3.0437100671	1.6701648386
H	-0.1698804532	2.9088182045	2.3701682111
H	1.4977785399	2.3961821379	1.9776036815
H	0.9942310380	4.0918146844	1.7304991383
C	-0.3066233823	-2.3936734401	1.9326205328
H	0.1626906006	-2.7782115950	1.0088472358
H	0.4726854171	-2.3599625344	2.7084972474
H	-1.0345115790	-3.1708429278	2.2495621703
C	-0.8106469555	-0.0572078781	2.8771466083
H	0.1973347470	-0.0954411498	3.3223412034
H	-0.9669376549	0.9771448598	2.5391184334
H	-1.5329424365	-0.2327618024	3.7043848998

TS3_4
M06-2X/def2SVP Electronic E: -1258.307253 a.u.
M06-2X/def2SVP Gibbs free E: -1258.046675 a.u.
M06-2X/def2TZVPP Electronic E: -1259.113167 a.u.

C	3.1449335532	-0.1980621156	1.4323707287
C	3.9818054651	0.3499780155	0.4514452558
C	3.7313548487	0.0394436463	-0.8915124634
C	2.6557242323	-0.7722357613	-1.2450171462
C	1.7708683948	-1.3040176626	-0.2733787216
C	2.0650649981	-1.0059121736	1.0827632809
H	3.3360121028	0.0102733889	2.4874578875
H	4.8233884521	0.9857695810	0.7289160385
H	4.3810158150	0.4395331263	-1.6729647729
H	2.4692219566	-0.9929115480	-2.2989859740
H	1.4321212094	-1.4120057826	1.8735659992
C	0.6260744053	-2.0999658004	-0.6797822443
H	0.5970401720	-2.3859336463	-1.7357794182
C	-0.3998820615	-2.4807405375	0.1421034351
H	-1.0888765778	-3.2602105340	-0.1855532465
H	-0.3465502866	-2.3235465459	1.2222995323
C	-1.5816411233	2.1696778698	-1.3112992962
C	-1.7952201174	0.7965553119	-1.2235870097
C	-2.0475813075	0.1351257758	0.0343830540
C	-1.9098303382	0.9875864498	1.1906447750
C	-1.6841291980	2.3520457643	1.0838135343
C	-1.5302601526	2.9830083197	-0.1684695403
H	-1.4540977061	2.6194318846	-2.3003862187
H	-1.8560187142	0.2223731255	-2.1486672131
H	-2.0549419430	0.5640488462	2.1859056659

H	-1.6394141119	2.9508278866	1.9978964538
H	-1.3940061236	4.0623096934	-0.2452695293
C	-2.4159155716	-1.2239200032	0.0993040958
K	1.0368440657	1.5698046723	-0.0310288221
C	-2.9361780048	-1.8005254889	1.3899609953
H	-3.9748052226	-1.4911637508	1.6238481281
H	-2.9372607340	-2.9027362381	1.3480799886
H	-2.3208340678	-1.5215137415	2.2603655148
C	-2.9056170018	-1.8769863628	-1.1702992444
H	-3.2013042548	-2.9212637990	-0.9821108809
H	-3.7852510324	-1.3659029243	-1.6093784312
H	-2.1326380197	-1.9030279414	-1.9597251910

TS4_5'

M06-2X/def2SVP Electronic E: -2018.348377 a.u.

M06-2X/def2SVP Gibbs free E: -2017.742521 a.u.

M06-2X/def2TZVPP Electronic E: -2020.006199 a.u.

C	2.0863187741	-2.9107303842	-2.4235533778
C	2.8128832856	-3.5419773434	-1.4007032278
C	2.2815812458	-3.4700208927	-0.0970277443
C	1.1296814640	-2.7517435110	0.1760340991
C	0.4048337918	-2.0141500063	-0.8326152586
C	0.9277227559	-2.1855587865	-2.1664600153
H	2.4374474236	-2.9864454658	-3.4569994392
H	3.7147804871	-4.1155208278	-1.6154944898
H	2.7827465139	-3.9964558676	0.7207125219
H	0.7409285524	-2.7347121618	1.1974078509
H	0.4131261491	-1.7148211842	-3.0055264544
C	-0.6890127837	-1.1910998756	-0.4918603355
H	-1.0266070394	-1.3076249101	0.5391547361
C	-1.8094446829	-0.8724449523	-1.4479547545
H	-1.9861614037	0.2135055541	-1.5229661971
H	-1.5705458961	-1.2201804566	-2.4688719569
C	-4.2551717776	0.9428030251	1.6697821875
C	-3.8514700324	0.4055401756	0.4497136524
C	-3.6193782391	-0.9718512612	0.2955685945
C	-3.7917937368	-1.7863547457	1.4219103953
C	-4.1895622787	-1.2511843692	2.6512285669
C	-4.4287426248	0.1144194416	2.7810743116
H	-4.4266803063	2.0178816001	1.7563531113
H	-3.6931572467	1.0763119155	-0.3966922378
H	-3.6140863449	-2.8599203801	1.3527724506
H	-4.3140853014	-1.9132259219	3.5106835273
H	-4.7419757715	0.5328059568	3.7394033843
C	-3.1737841175	-1.5166008797	-1.0651860833
C	-3.0408380394	-3.0399919699	-1.0623958180
H	-3.9994602511	-3.5310727190	-0.8324355929
H	-2.7162877477	-3.3802850725	-2.0580875614
H	-2.2905246528	-3.3837427204	-0.3362500918
C	-4.2126024497	-1.1243221150	-2.1275483051
H	-3.9210790001	-1.5297666891	-3.1093087318
H	-5.2090808984	-1.5203821394	-1.8764321668
H	-4.2967289793	-0.0317443907	-2.2284675961
B	0.1896231960	1.2258332308	0.1936774387
O	1.2712907029	1.4491439671	-0.6732788572
O	-0.9286423582	1.9028544892	-0.2648889383
C	0.8135978908	2.1749980127	-1.8279414596
C	-0.5409066858	2.7937145321	-1.3216178587
C	1.8729759326	3.2002803824	-2.2051143940
C	0.6236772958	1.1890431578	-2.9730610480
C	-0.3614927827	4.1817266895	-0.7084087979
C	-1.6361895697	2.8577585360	-2.3754651492
H	2.1474653453	3.8354877914	-1.3536496393
H	2.7794913506	2.6836577721	-2.5557603344
H	1.5147348974	3.8423825198	-3.0232911153
H	0.3215351874	1.7054286923	-3.8952329094
H	1.5749075168	0.6717236843	-3.1740857587
H	-0.1330645741	0.4386837948	-2.7175324110
H	-0.1425443876	4.9353065254	-1.4779513890
H	-1.2940864575	4.4645462644	-0.1992371495
H	0.4494538722	4.1942413704	0.0317017425
H	-2.5464862311	3.2831370505	-1.9278467189
H	-1.3292648000	3.5121974416	-3.2050575674
H	-1.8801530134	1.8696196157	-2.7834513177
C	0.4732732602	1.0506121297	1.7857954803
C	1.9225185739	0.6310315826	2.0194417796
C	-0.5282529044	0.1500652880	2.5160563230
C	0.2668637370	2.4748540133	2.3588888425
C	2.9803836548	1.4996293682	1.6765282230
C	2.2764357869	-0.6190660272	2.5484375077
H	-0.4435463340	0.2884133642	3.6074755837
H	-1.5552935448	0.4100271785	2.2166690669
H	-0.3904444944	-0.9161145066	2.3047037037
H	0.8921787634	3.2335033396	1.8689254855
H	-0.7819567465	2.7793053860	2.2322719216
H	0.5068187562	2.4869323189	3.4347006988
C	4.3149918655	1.1196382831	1.8081323436
H	2.7553449521	2.4846628535	1.2679303083
C	3.6142982731	-1.0110759336	2.6747864973
H	1.5022634659	-1.3174441479	2.8619695254
C	4.6426646783	-0.1503524677	2.2960055898
H	5.1051147863	1.8179130497	1.5245297014
H	3.8469994098	-1.9992329389	3.0766704825
H	5.6860215591	-0.4548616813	2.3937741166
K	3.0212273330	-0.5566886416	-0.6129185023

TS4_5''

M06-2X/def2SVP Electronic E: -1567.632863 a.u.

M06-2X/def2SVP Gibbs free E: -1567.242048 a.u.

M06-2X/def2TZVPP Electronic E: -1568.777210 a.u.

C	-0.2230196711	2.3239773736	0.4992634593
C	0.2143275440	3.0255200206	-0.6430069937
C	0.4573272518	2.2807069110	-1.8084667562
C	0.2453400273	0.9056718299	-1.8472898094
C	-0.3100606839	0.1832495591	-0.7344992827
C	-0.4424922441	0.9538689775	0.4736420567
H	-0.4058114658	2.8684502917	1.4301422115
H	0.3502123262	4.1074794408	-0.6239062531
H	0.8196399551	2.7862495182	-2.7082535335
H	0.4134934105	0.3643127116	-2.7823933135
H	-0.8135562429	0.4687020332	1.3783111589
C	-0.6719008683	-1.1738152937	-0.8683315911
H	-0.7690787577	-1.5137792026	-1.9027071263
C	-1.4733214701	-1.9277585285	0.1504261340
H	-1.2327469192	-3.0070335260	0.0835734428
H	-1.1791737655	-1.6235794608	1.1709672503
C	-3.7911025848	1.8574072316	-0.6509954465
C	-3.5095839316	0.5037718037	-0.8298543235
C	-3.4626724933	-0.3840285511	0.2564723473
C	-3.7415152002	0.1374527713	1.5284455762
C	-4.0285339311	1.4910567275	1.7132782377
C	-4.0474709861	2.3612885673	0.6245012885
H	-3.8040623561	2.5238751198	-1.5158970034
H	-3.2980808974	0.1432400278	-1.8365111860
H	-3.7260533812	-0.5149003488	2.4024805978
H	-4.2373558875	1.8654473171	2.7176943529
H	-4.2655740199	3.4214178810	0.7669062893
C	-3.0335002763	-1.8394253785	0.0685395759
C	-3.6080026674	-2.7406874623	1.1687251903
H	-4.7041090084	-2.6548588733	1.2302338537
H	-3.3602857975	-3.7908532899	0.9521917587
H	-3.1885534598	-2.5069090249	2.1580043614
C	-3.5061796133	-2.3902552003	-1.2821782914
H	-3.2270823237	-3.4521235111	-1.3667545230
H	-4.6009306242	-2.3164099616	-1.3771459010
H	-3.0529826911	-1.8647092475	-2.1329234693
C	3.3802528854	-1.2923689477	-0.2311029018
C	3.1286954453	-1.1798180653	1.1625998380

C	4.5796558456	-0.7013257788	-0.7068631649
C	4.0038648175	-0.5000414163	2.0066820238
H	2.2215404687	-1.6162192592	1.5843788384
C	5.4478045554	-0.0176007037	0.1406619836
H	4.8125422628	-0.7756582646	-1.7721318757
C	5.1657670158	0.1039250215	1.5079824387
H	3.7709883466	-0.4309844284	3.0716955440
H	6.3552636738	0.4307616331	-0.2698183108
H	5.8445734201	0.6399389236	2.1722687609
C	2.4681092974	-1.9399837131	-1.1497464460
C	1.2646308542	-2.4943569792	-0.7931073790
H	0.7367515993	-3.1305291867	-1.5048769540
H	1.0210053839	-2.6746528784	0.2558262840
H	2.7707482356	-1.9459153226	-2.2009249676
K	2.6817645970	1.5861651125	0.0847159487

TS5_6
M06-2X/def2SVP Electronic E: -1902.011536 a.u.
M06-2X/def2SVP Gibbs free E: -1901.455084 a.u.
M06-2X/def2TZVPP Electronic E: -1903.552916 a.u.

C	-2.2999269258	-3.5884516867	-0.4756637980
C	-2.5899008297	-3.5609019775	-1.8532642892
C	-1.7928419896	-2.7206714874	-2.6591065486
C	-0.8086081938	-1.9119909151	-2.1170056737
C	-0.5181749349	-1.8723950111	-0.6964526525
C	-1.3108748126	-2.7951518149	0.0890351393
H	-2.8704301065	-4.2556048872	0.1778548926
H	-3.3538490851	-4.2068075134	-2.2863317310
H	-1.9540190150	-2.7009024104	-3.7412776504
H	-0.2150298522	-1.2736584954	-2.7770699848
H	-1.1357433056	-2.8642291637	1.1645411458
C	0.4065595929	-0.9674415432	-0.1751967128
H	0.9480551368	-0.3790959287	-0.9188421207
C	1.0025262281	-1.0195101634	1.1961690211
H	1.1284032341	0.0032000624	1.5983236725
H	0.3188500782	-1.5365974374	1.8909457978
C	4.9892973345	0.9161435167	0.2573011806
C	4.1435128086	0.1333435184	1.0455524097
C	3.4051019367	-0.9274584483	0.4995105604
C	3.5601891705	-1.1792968355	-0.8741743230
C	4.4003310353	-0.3970089613	-1.6672672265
C	5.1187225382	0.6593838391	-1.1065518302
H	5.5487211929	1.7340173561	0.7163963607
H	4.0591680068	0.3669673321	2.1074789395
H	3.0076843673	-1.9949784932	-1.3409140675
H	4.4943283263	-0.6183616666	-2.7325033296
H	5.7777184050	1.2710547963	-1.7255888154
C	2.3994320095	-1.7156690662	1.3380534909
K	-3.2996409153	-0.7153978621	-0.9943842378
C	2.3064452093	-3.1738142749	0.8768478248
H	3.2921692344	-3.6653859510	0.9054768097
H	1.6288528307	-3.7307594098	1.5427463245
H	1.9031445257	-3.2601159118	-0.1410814070
C	2.7767603908	-1.7041923493	2.8240642732
H	2.0911579476	-2.3596798627	3.3824343778
H	3.8037333277	-2.0669618694	2.9885691155
H	2.6919711537	-0.6992115727	3.2634023613
B	-1.1469468699	1.0552315442	0.2603377815
O	-0.2414776295	1.9781435395	0.7221365087
O	-1.4460588571	1.2336025733	-1.0878210082
C	0.2599722038	2.7527766492	-0.3762041781
C	-0.6666056112	2.3234100230	-1.6045543747
C	0.1347544592	4.2245801598	0.0018832065
C	1.7396541986	2.4189523954	-0.5516959585
C	-1.6591878575	3.4021617784	-2.0293362609
C	0.1128790723	1.8463324839	-2.8224244856
H	-0.9009230588	4.5010895268	0.2357914442
H	0.7492549317	4.4158285126	0.8930621826
H	0.4977529508	4.8710676220	-0.8097968288
H	2.1822491819	2.9906006603	-1.3796241807
H	2.2681770636	2.6900463947	0.3737003736
H	1.9052863358	1.3493866987	-0.7323963732
H	-1.1481549139	4.2930552632	-2.4189890206
H	-2.3011583009	2.9990111054	-2.8262240034
H	-2.3028947874	3.7018136593	-1.1910391026
H	-0.5931647124	1.4953209313	-3.5893049075
H	0.7016117555	2.6702901791	-3.2509283992
H	0.7940712959	1.0223969709	-2.5758780817
O	-2.0452567123	0.3633490104	1.0447438074
C	-2.4584912835	0.7566745145	2.3642401397
C	-3.0044517662	2.1825748343	2.3142574046
H	-3.4130663007	2.4716742312	3.2932896496
H	-2.2079011941	2.8915938786	2.0475311417
H	-3.8103582882	2.2571810280	1.5677094763
C	-1.2955458622	0.6540771420	3.3452308160
H	-1.6289129336	0.9492180909	4.3510008068
H	-0.9222417585	-0.3785468416	3.3918012461
H	-0.4735253593	1.3144730266	3.0377527502
C	-3.5618572757	-0.2237471466	2.7448482941
H	-3.9142688008	-0.0336356258	3.7684859597
H	-4.4244588344	-0.1228149778	2.0666551248
H	-3.1861225364	-1.2563582864	2.6900057504

TS5'_6'
M06-2X/def2SVP Electronic E: -2018.347116 a.u.
M06-2X/def2SVP Gibbs free E: -2017.740680 a.u.
M06-2X/def2TZVPP Electronic E: -2020.004057 a.u.

C	1.5256264209	-2.7961446895	-2.6152490418
C	1.9143792220	-3.7533678498	-1.6715243804
C	1.3663682037	-3.7022537403	-0.3888162111
C	0.4540387442	-2.6972873296	-0.0520907186
C	0.0723085194	-1.7116266006	-0.9742614621
C	0.6194531510	-1.7934329833	-2.2679164568
H	1.9351072429	-2.8283442659	-3.6269963219
H	2.6263836639	-4.5360426140	-1.9387556223
H	1.6473196433	-4.4478348831	0.3577198388
H	0.0330124642	-2.6762797934	0.9541323436
H	0.3442747660	-1.0433497827	-3.0114396793
C	-0.8356229628	-0.5661588703	-0.5815691967
H	-1.2300820540	-0.7786904831	0.4193561765
C	-2.0536277806	-0.3903311554	-1.5237250590
H	-2.2934037670	0.6777770114	-1.6140217438
H	-1.8047438965	-0.7319829352	-2.5428572674
C	-4.2640798048	1.1916091306	1.8254391007
C	-3.8789133754	0.7179002034	0.5733021657
C	-3.7487951925	-0.6580614596	0.3195975574
C	-4.0167726658	-1.5395876390	1.3741568603
C	-4.4054061326	-1.0688673635	2.6320671974
C	-4.5311693008	0.2979124652	2.8649041680
H	-4.3500788029	2.2671541382	1.9924144831
H	-3.6643683348	1.4385275091	-0.2183757278
H	-3.9227773197	-2.6157439217	1.2272305318
H	-4.6056658821	-1.7818828744	3.4343644382
H	-4.8289157192	0.6668727358	3.8479693330
C	-3.3302555583	-1.1389581709	-1.0756382603
C	-3.0859451684	-2.6495181248	-1.1103477777
H	-4.0115416258	-3.2110871877	-0.9121759874
H	-2.7218940576	-2.9425629658	-2.1070923291
H	-2.3335071637	-2.9631030126	-0.3722887467
C	-4.4557709111	-0.8037944901	-2.0674914625
H	-4.1841340938	-1.1375057965	-3.0815046306
H	-5.3933556465	-1.3042567939	-1.7809683870
H	-4.6447912929	0.2796441993	-2.1043237772
B	-0.0054256906	0.8156578951	-0.5024882076
O	1.2923830829	0.9269902553	-1.0349163987
O	-0.6534145892	2.0348078019	-0.6097554522
C	1.3391022310	2.1096942548	-1.8509176341

C	0.2111965055	3.0050923694	-1.2194636094
C	2.7364350554	2.7048683160	-1.7942688487
C	1.0133732034	1.6824336662	-3.2808150259
C	0.7493840036	3.9612176454	-0.1636479793
C	-0.6037298110	3.7928951965	-2.2363411467
H	3.0636699867	2.8682736556	-0.7598257400
H	3.4509466536	2.0242901391	-2.2831331276
H	2.7709352701	3.6636105966	-2.3323423155
H	1.0664103710	2.5309758437	-3.9769595044
H	1.7419687973	0.9242949195	-3.6047575910
H	0.0058946464	1.2430175801	-3.3419832804
H	1.3212516608	4.7733784599	-0.6349222174
H	-0.0904332705	4.4032290442	0.3902381505
H	1.3998252114	3.4435784776	0.5487413622
H	-1.3561188107	4.3952986258	-1.7070892746
H	0.0412994687	4.4769123653	-2.8075458927
H	-1.1278659145	3.1313820878	-2.9375885835
C	0.4120370658	0.6708538165	2.1508297860
C	1.7670448068	0.2662416246	2.1647614107
C	-0.6354834665	-0.2767419649	2.6893783469
C	0.0338590842	2.1020912413	2.4658320074
C	2.8708640840	1.1498586042	1.8626844591
C	2.1817587628	-1.0812534339	2.4906417566
H	-0.6129591930	-0.3361485627	3.7994143568
H	-1.6470956280	0.0698693960	2.4174236892
H	-0.5488321949	-1.3091829284	2.3232156093
H	0.8793579063	2.8001783558	2.4215317277
H	-0.7535614383	2.4941550345	1.8003688642
H	-0.3645376632	2.1674115909	3.4997320101
C	4.1931393541	0.7304152001	1.8618165170
H	2.6625051902	2.1930692178	1.6215145451
C	3.5110753341	-1.4829441960	2.4853400616
H	1.4256377759	-1.8118518548	2.7801539980
C	4.5534817784	-0.5990848618	2.1535293333
H	4.9728700748	1.4600522881	1.6232876076
H	3.7463305126	-2.5164731238	2.7570836684
H	5.5964847094	-0.9162434999	2.1685397463
K	3.0206205510	-0.9024417558	-0.4415231314

Int1_3sol

M06-2X/def2SVP Electronic E: -2289.271032 a.u.

M06-2X/def2SVP Gibbs free E: -2288.512860 a.u.

M06-2X/def2TZVPP Electronic E: -2291.272569 a.u.

C	-0.5250421384	3.5698601778	1.7195128518
C	-0.0881722009	3.8177304980	0.4175827498
C	1.0883676362	3.2366239595	-0.0596378465
C	1.8720940997	2.3766017651	0.7353937066
C	1.4257289735	2.1682185993	2.0563541088
C	0.2503109215	2.7462573644	2.5384009081
H	-1.4461376834	4.0199653629	2.0949184659
H	-0.6667379982	4.4731309989	-0.2371250955
H	1.3910789781	3.4463870921	-1.0848820035
H	1.9955053600	1.5125743098	2.7139912789
H	-0.0605912235	2.5526144211	3.5675154752
C	3.0677025013	1.6365420046	0.1811232270
C	2.0980559822	-1.8827414905	0.8132958068
O	3.5127646469	-0.8042065443	-0.7496961318
O	1.8398200794	-0.4987620837	0.8345665396
C	3.4468036358	-1.9990600903	-0.0052043520
C	4.6845694310	-2.0783129311	0.8955533460
H	4.7557274990	-3.0399745988	1.4250265381
H	5.5777087596	-1.9652240394	0.2626059156
H	4.6907406376	-1.2683505500	1.6354140228
C	3.4740462546	-3.1939180326	-0.9577195456
H	4.4327018975	-3.2083107599	-1.4985482772
H	3.3757871261	-4.1462741777	-0.4132989620
H	2.6673835944	-3.1305907744	-1.7000211097
C	2.2127982820	-2.4100013769	2.2420002909
H	1.2307186455	-2.3612034671	2.7363018483
H	2.5401293728	-3.4611798004	2.2529334717
H	2.9217494707	-1.8150671010	2.8329660488
C	0.9038966110	-2.5739618581	0.1462912414
H	0.9927859091	-3.6712162941	0.1251860308
H	0.0059483396	-2.3162334293	0.7324108376
H	0.7561950764	-2.2029106960	-0.8747898783
B	2.4831322625	0.1478035679	-0.3446363189
C	1.4310389739	0.2170066649	-2.7988666519
C	2.8144037962	0.1680117294	-3.4568676030
C	0.6785861939	-1.0727621242	-3.1575658026
C	0.6465759012	1.4093138376	-3.3610174047
H	3.3726405864	1.0973468944	-3.2836701499
H	3.4037299142	-0.6628077988	-3.0517882136
H	2.6975614878	0.0388499852	-4.5442400365
H	-0.2782621095	-1.1106915184	-2.6137515255
H	0.4766546670	-1.1292291404	-4.2386067852
H	1.2726485552	-1.9554122647	-2.8784873168
H	0.5263239249	1.3389048157	-4.4529247712
H	-0.3569796135	1.4520383733	-2.9044918743
H	1.1672957704	2.3507545265	-3.1289520543
O	1.4479500556	0.3780883728	-1.3992484204
K	-0.6223808712	0.5017603663	0.1836711869
C	3.7225311231	2.3989696323	-0.9707826041
H	3.0383927599	2.5449137912	-1.8172164667
H	4.0850575990	3.3952526343	-0.6558940006
H	4.5870980695	1.8311164820	-1.3489869723
C	4.1268814865	1.3964755996	1.2599947758
H	5.0102609144	0.9222042210	0.8069784716
H	4.4532895859	2.3360543935	1.7422846275
H	3.7639424270	0.7158317613	2.0429996987
O	-1.8516154288	-0.5674099991	2.2702584243
C	-2.7535392433	-1.6615085392	2.3311355354
C	-0.9655178299	-0.6020376143	3.3917504102
C	-1.9841736166	-2.7504088177	3.0650522457
H	-3.6644791321	-1.3778234979	2.8911867932
H	-3.0421635432	-1.9223026602	1.3027621413
C	-1.2393909697	-1.9268038246	4.1193468743
H	0.0669483138	-0.5308276997	3.0089580287
H	-1.1542558739	0.2673439103	4.0407237767
H	-1.2739196812	-3.2372073207	2.3779025143
H	-2.6367307227	-3.5208175604	3.4959937170
H	-0.3183910232	-2.4067488300	4.4758091963
H	-1.8904105027	-1.7554439824	4.9887785753
O	-2.8757845987	1.8427849870	-0.3282153316
C	-3.3407774180	3.1058322411	-0.7660807442
C	-3.9539934355	1.2365497296	0.3679151432
C	-4.7656625156	2.8433458249	-1.2603209798
H	-3.3407889202	3.8194034477	0.0790045254
H	-2.6592123869	3.4772608454	-1.5422023515
C	-5.2253426459	1.6459357549	-0.3985977089
H	-3.7715321945	0.1527352062	0.3919750342
H	-3.9788004584	1.6098672629	1.4072436108
H	-4.7510165295	2.5729917306	-2.3252731791
H	-5.4076663732	3.7259651830	-1.1453797030
H	-5.5899582774	0.8243548834	-1.0302199485
H	-6.0320989418	1.9159154159	0.2949806306
O	-2.2014467511	-1.3407422326	-1.0880482689
C	-2.2120503419	-2.7608627290	-1.1434987683
C	-3.0885504494	-0.9106166142	-2.1103801508
C	-3.6557423007	-3.1603340799	-1.5122031099
H	-1.4983684059	-3.1013130589	-1.9154285391
H	-1.8801284715	-3.1492072687	-0.1720948566
C	-4.2968526109	-1.8306147436	-1.9608020264
H	-3.2955456137	0.1565965687	-1.9610108918
H	-2.6106127449	-1.0465689775	-3.0994222366
H	-4.1863618459	-3.5924891672	-0.6536522570
H	-3.6576513924	-3.9086139353	-2.3154180293
H	-4.9644717071	-1.4389476493	-1.1789931751
H	-4.8737943531	-1.9190994497	-2.8902542170

Int1_4sol
M06-2X/def2SVP Electronic E: -2521.452915 a.u.
M06-2X/def2SVP Gibbs free E: -2520.579349 a.u.
M06-2X/def2TZVPP Electronic E: -2523.722892 a.u.

C	-6.3668949773	1.5990571845	-1.8973249182
C	-6.2678075582	1.2743954282	-0.5403937755
C	-5.2299142285	0.4688777402	-0.0828198646
C	-4.2466501522	-0.0404619002	-0.9542381047
C	-4.3747830022	0.2842999773	-2.3142444722
C	-5.4148178324	1.0956550858	-2.7799538925
H	-7.1787282701	2.2334854345	-2.2583894379
H	-7.0040550975	1.6624585355	0.1674467642
H	-5.1369164795	0.2581986176	0.9840515963
H	-3.6454063632	-0.0885799760	-3.0341682709
H	-5.4772011154	1.3328159714	-3.8446770649
C	-3.1173981239	-0.8833750584	-0.4028574969
C	-0.9112707070	-0.6750618445	2.5621956087
O	-2.7596281445	0.5906746396	1.8203308769
O	-1.1427046776	-0.9685567538	1.2042493807
C	-2.2414786376	0.0442292931	3.0127012868
C	-3.2618160150	-0.9371925251	3.6007967656
H	-2.9650825903	-1.2944903151	4.5983748797
H	-4.2289998264	-0.4199501796	3.6945760363
H	-3.4002107874	-1.8062046740	2.9468297217
C	-2.0205105771	1.1602718899	4.0320589552
H	-2.9888002369	1.6169150155	4.2877014448
H	-1.5723949985	0.7709041077	4.9597668249
H	-1.3688004227	1.9488933693	3.6351318886
C	-0.6260111451	-1.9686773772	3.3220423041
H	0.3310676232	-2.3948105856	2.9818763542
H	-0.5449353827	-1.7864832794	4.4051361964
H	-1.4103514507	-2.7173059336	3.1493231992
C	0.3242749004	0.2287951162	2.6716890782
H	0.5800661903	0.4756205181	3.7133915518
H	1.1880389726	-0.3022985237	2.2376493225
H	0.1744745184	1.1592283047	2.1101645009
B	-2.1288794356	0.0034658945	0.6317230724
C	-1.5233945237	2.4025133382	-0.3146812015
C	-2.9731564771	2.8912411386	-0.2442543294
C	-0.7136368855	3.1236227019	0.7695086371
C	-0.9387023011	2.7445275522	-1.6895871433
H	-3.5672943791	2.4998168622	-1.0809081466
H	-3.4337362778	2.5634279530	0.6968282867
H	-2.9951863260	3.9915164706	-0.2925799264
H	0.3317614092	2.7824212795	0.7386593965
H	-0.7347149567	4.2152683100	0.6240971519
H	-1.1364648047	2.8952087156	1.7582189539
H	-0.9382875423	3.8299740706	-1.8761123672
H	0.0976048170	2.3734364163	-1.7600848457
H	-1.5344391024	2.2549968745	-2.4762892991
O	-1.3801158418	1.0077819669	-0.1797981266
K	0.8596520870	-0.3323349441	-0.5017771845
C	-3.7211492450	-2.0962211085	0.3212235365
H	-4.4264016307	-1.7988573890	1.1095851034
H	-4.2704106516	-2.7497400083	-0.3814298635
H	-2.9216427884	-2.6900115399	0.7913671329
C	-2.2140796131	-1.4191119287	-1.5183029682
H	-1.4174957533	-2.0401285427	-1.0815000786
H	-2.7646270660	-2.0502230042	-2.2401269763
H	-1.7350892703	-0.6024911273	-2.0801740100
O	3.2589235194	-1.1624466535	0.4295110830
C	4.6065466922	-0.7206992841	0.4530266704
C	3.1385992668	-2.1647009156	1.4225557345
C	5.0836750460	-0.8591617259	1.9152011765
H	5.2135718283	-1.3508761589	-0.2212017552
H	4.6302636040	0.3115251669	0.0765357350
C	3.9297364061	-1.6147562392	2.6052133493
H	2.0699302934	-2.3269525313	1.6178200935
H	3.5760895171	-3.1158059575	1.0608999640
H	5.2623330373	0.1200617339	2.3803109832
H	6.0229030556	-1.4256567099	1.9632368195
H	3.2963749889	-0.9223486247	3.1801616733
H	4.2760193468	-2.4043495203	3.2842078174
O	1.7693460812	0.4274879737	-2.8829193276
C	1.3558455314	0.8024681270	-4.1843854243
C	3.1859775052	0.3256460188	-2.9062700482
C	2.3732549757	1.8580994654	-4.6056837629
H	1.3892791265	-0.0729701384	-4.8597513866
H	0.3218139402	1.1665144932	-4.1279260159
C	3.6749732328	1.3537362125	-3.9503950635
H	3.5476208347	0.5185958687	-1.8860204155
H	3.4809881606	-0.6988607379	-3.1940693512
H	2.0806517260	2.8353384320	-4.1943717268
H	2.4509252799	1.9569835884	-5.6959811565
H	4.2380073797	2.1742726462	-3.4849707978
H	4.3348496426	0.8730141541	-4.6844815586
O	0.9686290777	-3.0053448929	-0.7089595646
C	2.0082500816	-3.7653729778	-1.3105497630
C	0.0989353410	-3.8792665224	-0.0046420672
C	2.0318113374	-5.1299508267	-0.5901709404
H	1.8095955558	-3.8862246080	-2.3888591129
H	2.9431089657	-3.1962276474	-1.1953241245
C	1.0209689996	-4.9557582120	0.5532248150
H	-0.4349545496	-3.2779915196	0.7449688411
H	-0.6420068172	-4.3226240744	-0.6973385432
H	3.0352647190	-5.3941251324	-0.2307523370
H	1.7019947978	-5.9256629092	-1.2725628647
H	1.5157758840	-4.5925997329	1.4659808045
H	0.4877544236	-5.8834794401	0.7985168064
O	2.4084174400	1.8095073363	0.0488231589
C	2.9858624444	2.0530124498	1.3225985783
C	2.7406424674	2.9221397309	-0.7620229245
C	4.2904034796	2.8367866758	1.0595963660
H	2.2873543090	2.6498028900	1.9366060866
H	3.1346205422	1.0820618081	1.8143220307
C	4.2098873030	3.1805760562	-0.4419314730
H	2.5341069713	2.6588610758	-1.8075408448
H	2.1143385407	3.7924827282	-0.4864056744
H	5.1811229313	2.2365619344	1.2907088356
H	4.3291781339	3.7419094534	1.6796388415
H	4.8398025113	2.4970266536	-1.0320395863
H	4.5179962446	4.2094487650	-0.6676386966

Int2_3sol
M06-2X/def2SVP Electronic E: -2289.246440 a.u.
M06-2X/def2SVP Gibbs free E: -2288.491418 a.u.
M06-2X/def2TZVPP Electronic E: -2291.252623 a.u.

C	1.8957947790	3.4402372884	-1.3081167960
C	1.2138252575	3.7512407890	-0.1132012290
C	-0.1585414589	3.6197285233	0.0120459670
C	-1.0040145176	3.1666397587	-1.0880015345
C	-0.2626526821	2.8642692581	-2.3079870016
C	1.1121941404	3.0034419018	-2.3952659968
H	2.9763140667	3.5580075429	-1.3957618728
H	1.7854693329	4.1003843940	0.7537305049
H	-0.6262235753	3.8734501071	0.9663721946
H	-0.8122949355	2.5325843854	-3.1911045554
H	1.6016902209	2.7597947340	-3.3443610831
C	-2.3782675341	3.0436079849	-0.9900308053
C	-3.0709096058	-1.6440975376	-0.4312061448
O	-3.0779340443	-0.7763397489	1.7449114669
O	-1.9007366414	-0.8225310109	-0.2229715812
C	-4.0197425804	-1.1466838098	0.7124491271
C	-4.7686651939	0.1219652255	0.3191434923
H	-5.5643233903	-0.0892583075	-0.4089027770
H	-5.2239216194	0.5614858369	1.2181956424
H	-4.0770530014	0.8594608033	-0.1164600888

C	-4.9696574215	-2.1965622472	1.2548596682
H	-5.6007412412	-1.7515502145	2.0373810199
H	-5.6270490244	-2.5681094665	0.4550901322
H	-4.4266379861	-3.0444788170	1.6912840187
C	-3.6161781639	-1.4221656129	-1.8296338678
H	-2.9218659374	-1.8381250840	-2.5742443070
H	-4.5826240432	-1.9353633882	-1.9444920473
H	-3.7539563729	-0.3546916136	-2.0413529965
C	-2.6506610516	-3.0987201514	-0.2491680975
H	-3.4881765631	-3.7808744886	-0.4514385416
H	-1.8402645522	-3.3293545284	-0.9565399599
H	-2.2852440781	-3.2839738995	0.7719273546
B	-1.9152150747	-0.4395687150	1.1021140664
C	-0.5721688368	0.4292935770	3.0229921943
C	-1.6281843344	1.3576586396	3.6100845663
C	-0.5418072019	-0.9154271543	3.7407953449
C	0.8046428627	1.0829130894	3.0504358641
H	-1.6270478514	2.3183661707	3.0744588203
H	-2.6284298775	0.9094850576	3.5329210114
H	-1.4092623934	1.5517884371	4.6700766630
H	0.2199118495	-1.5602657977	3.2776663606
H	-0.2872846179	-0.7785809893	4.8014752767
H	-1.5208673056	-1.4130771819	3.6791906493
H	1.1170628117	1.2790257086	4.0861551467
H	1.5448556800	0.4267914766	2.5663809214
H	0.7778394077	2.0404996243	2.5067571639
O	-0.8443985404	0.1976759639	1.6284069673
K	0.8320091965	0.7145919616	-0.3363817733
C	-3.0867074488	3.4063106066	0.2905090577
H	-2.8186815580	2.7537122755	1.1471956118
H	-2.8751409116	4.4400350861	0.6311044846
H	-4.1793022083	3.3327412487	0.1746612374
C	-3.1780726347	2.5910842774	-2.1847725248
H	-4.2437131417	2.4718579552	-1.9327898015
H	-3.1352206922	3.2948394470	-3.0425033488
H	-2.8346743317	1.6194745527	-2.5935458698
O	1.2445797816	-0.8809330370	-2.4093311094
C	1.9641255052	-2.0903039224	-2.6098938940
C	0.0515752277	-0.8688712026	-3.2013589205
C	0.9436122733	-3.0755139056	-3.1631600980
H	2.7857744272	-1.9255685638	-3.3300766848
H	2.4017136900	-2.3968903710	-1.6483432731
C	0.0807762450	-2.1554680136	-4.0276527977
H	-0.8158810915	-0.8334854882	-2.5202466494
H	0.0381629734	0.0366033819	-3.8258288929
H	0.3469334794	-3.5028393080	-2.3412109723
H	1.4065516784	-3.8993238548	-3.7218292670
H	-0.9245675618	-2.5511185168	-4.2238049268
H	0.5737762001	-1.9770821405	-4.9947365024
O	3.3013455279	0.9125807108	0.6812832476
C	4.1580373197	1.8775115001	1.2594540206
C	4.0333377526	0.3185032177	-0.3778584384
C	5.5164745171	1.1778903960	1.3379782650
H	4.2121867882	2.7709889357	0.6098056510
H	3.7440560647	2.1724720459	2.2330403133
C	5.4908265021	0.2308314367	0.1172974695
H	3.5740587048	-0.6555683483	-0.5992654999
H	3.9611384078	0.9560735194	-1.2788042432
H	5.5894726591	0.6047502121	2.2728423932
H	6.3506717645	1.8905931456	1.3114341512
H	5.7550701755	-0.7958730090	0.4043219483
H	6.1898763093	0.5470135850	-0.6677114156
O	1.6538143427	-1.5546993759	0.8638429569
C	0.7695176565	-2.6159840805	0.5429732488
C	2.6948717898	-2.1012779508	1.6500474720
C	1.6320560648	-3.8970919095	0.4823042225
H	-0.0065764757	-2.7023363441	1.3246776751
H	0.2697200405	-2.3581937141	-0.4015314597
C	3.0189281253	-3.4245757893	0.9632512987
H	3.5171795131	-1.3745135723	1.6783885486
H	2.3409788187	-2.2743810634	2.6849367571
H	1.6726722779	-4.3188557100	-0.5314573642
H	1.2217989419	-4.6696447678	1.1462016337
H	3.6896112945	-3.2423874419	0.1096318167
H	3.5094618605	-4.1416976093	1.6338819247

Int2_4sol

M06-2X/def2SVP Electronic E: -2521.425906 a.u.
M06-2X/def2SVP Gibbs free E: -2520.559007 a.u.
M06-2X/def2TZVPP Electronic E: -2523.699153 a.u.

C	-0.9572211608	-4.4524177054	0.3087309102
C	-2.3544275567	-4.2686905919	0.3322245262
C	-2.9553202366	-3.2475143821	1.0464577640
C	-2.1896828362	-2.2901408297	1.8290106741
C	-0.7556971604	-2.5267797555	1.8009535443
C	-0.1844078813	-3.5560900190	1.0693003729
H	-0.4987888087	-5.2708924590	-0.2481924206
H	-2.9933797495	-4.9560270014	-0.2324260189
H	-4.0446292104	-3.1625319405	1.0271261723
H	-0.0988495146	-1.8857185836	2.3914149526
H	0.9061161470	-3.6695062331	1.0998390010
C	-2.7631323196	-1.2548930035	2.5546709661
C	-1.0018622488	3.2666807180	1.2859085393
O	-2.5164562203	2.4840446203	-0.3256585769
O	-0.6156451544	1.9474844824	0.8444249820
C	-2.5077766094	3.3276011644	0.8469645766
C	-3.4344727026	2.6685329644	1.8632267329
H	-3.5226758913	3.2657615103	2.7814150813
H	-4.4338098181	2.5639467160	1.4170433773
H	-3.0687538284	1.6628920409	2.1235428143
C	-3.0017275546	4.7116796635	0.4739393610
H	-4.0627028192	4.6593914198	0.1911442202
H	-2.9090290197	5.3951106166	1.3308288614
H	-2.4396437098	5.1252339389	-0.3729990497
C	-0.7901734465	3.3864101564	2.7843272701
H	0.2857066826	3.3902836766	3.0134903033
H	-1.2181502158	4.3301442358	3.1539680435
H	-1.2559957543	2.5500078725	3.3203425383
C	-0.1289540865	4.2701403371	0.5401182987
H	-0.3258069307	5.2981145762	0.8753256744
H	0.9276428669	4.0370491893	0.7417569885
H	-0.2961937238	4.2155778519	-0.5462127728
B	-1.4959138737	1.5869970362	-0.1569679972
C	-2.1583452961	-0.1952065790	-1.7761543280
C	-3.5182985554	-0.3890045635	-1.1143863642
C	-2.2616900299	0.6512378237	-3.0385649739
C	-1.4916636255	-1.5354171990	-2.0607553950
H	-3.3800489387	-0.8637506459	-0.1317331904
H	-4.0274968045	0.5752416785	-0.9757451464
H	-4.1528534818	-1.0372475011	-1.7360003484
H	-1.2696990568	0.7656477383	-3.5003434362
H	-2.9308498846	0.1685669027	-3.7655547981
H	-2.6638231085	1.6476681645	-2.8028269962
H	-2.0830789875	-2.1228496115	-2.7776075696
H	-0.4876059226	-1.3669813394	-2.4832452328
H	-1.3999419518	-2.1155908466	-1.1286627500
O	-1.2662080194	0.4548713036	-0.8524803347
K	0.9872476074	-0.4731559216	0.1891769769
C	-4.2620606004	-1.1124292703	2.6298472003
H	-4.7456438745	-1.0065559481	1.6380272770
H	-4.7723458245	-1.9754924297	3.1094719798
H	-4.5478338540	-0.2208325036	3.2106439122
C	-1.8872274531	-0.3924989938	3.4286441215
H	-2.4787250473	0.3776482095	3.9497137379
H	-1.3514344590	-0.9620558389	4.2190258800
H	-1.0941677388	0.1428675829	2.8655340152
O	3.0507246433	1.1992497630	0.5532449797
C	3.9855651582	1.8779263956	-0.2703352796

C	2.8356491517	1.9347062022	1.7597301365
C	3.8357800844	3.3472535011	0.0985807452
H	5.0113891954	1.5211455107	-0.0602553132
H	3.7454284380	1.6502160383	-1.3186605192
C	3.6039888770	3.2551492029	1.6084038607
H	1.7492634765	2.0831289528	1.8709067644
H	3.1903566543	1.3472208552	2.6206384926
H	2.9524150250	3.7720886012	-0.4037658286
H	4.7128116842	3.9488507526	-0.1737679977
H	3.0486145284	4.1110431304	2.0155585219
H	4.5682663085	3.1970603910	2.1337467848
O	2.6050137775	-2.3152318067	-0.8422885732
C	2.3524777885	-3.5528149119	-1.4971549626
C	3.7535141070	-1.6813713068	-1.4079082854
C	2.9265956154	-3.3736658564	-2.8941432571
H	2.8613728634	-4.3755697532	-0.9618664840
H	1.2698677712	-3.7397649149	-1.4760794960
C	4.1817816827	-2.5463985355	-2.6022077930
H	3.4624591223	-0.6615240669	-1.7060263494
H	4.5467043083	-1.6027846431	-0.6466838185
H	2.2170950133	-2.8090776852	-3.5181790281
H	3.1415087767	-4.3281903575	-3.3918125521
H	4.5142363015	-1.9413629236	-3.4566496977
H	5.0092871046	-3.2113640329	-2.3152289503
O	2.1653628754	-0.9531161003	2.5390318597
C	3.4761558688	-1.4741648910	2.3246028481
C	1.9037479755	-0.8121397278	3.9346891829
C	3.8996344598	-2.0545329365	3.6673330286
H	3.4286383648	-2.2101645699	1.5092046074
H	4.1462412580	-0.6522110506	2.0121520810
C	3.2348269026	-1.0750406482	4.6345149099
H	1.4998678178	0.1939261988	4.1281459272
H	1.1436071162	-1.5533205507	4.2351735095
H	4.9900903102	-2.1149073330	3.7787359599
H	3.4766370894	-3.0625734328	3.7958240489
H	3.8255877318	-0.1478830632	4.6991317727
H	3.1033386664	-1.4728277813	5.6490230891
O	1.4635302075	0.6574157154	-2.2588301580
C	1.1760151026	2.0418264222	-2.1267122929
C	1.8005034461	0.4345541431	-3.6152301381
C	2.0652915474	2.7700853875	-3.1593271995
H	0.1077416104	2.2202095180	-2.3419760902
H	1.3699137553	2.3231623333	-1.0813423411
C	2.6863937720	1.6221030809	-3.9801912053
H	2.2969570377	-0.5418419539	-3.6952807782
H	0.8870076133	0.4168981843	-4.2392304824
H	2.8364872471	3.3870510249	-2.6785967153
H	1.4574928592	3.4316158194	-3.7905744046
H	3.7192435566	1.4232829860	-3.6569194481
H	2.7004859141	1.8218580888	-5.0592382791

Int3_3sol

M06-2X/def2SVP Electronic E: -2598.573919 a.u.

M06-2X/def2SVP Gibbs free E: -2597.688371 a.u.

M06-2X/def2TZVPP Electronic E: -2600.919047 a.u.

C	5.4696126997	-3.7264776680	-0.7202940663
C	5.3201878974	-2.7987960587	-1.7512204097
C	5.1873276025	-1.4403986686	-1.4655335039
C	5.1812707601	-0.9694654772	-0.1427625603
C	5.3526765498	-1.9156045529	0.8798599662
C	5.4958046832	-3.2751390303	0.5983812092
H	5.5710811028	-4.7901611879	-0.9437231634
H	5.3014419729	-3.1343932900	-2.7901071898
H	5.0563404875	-0.7406492204	-2.2903163731
H	5.3634985233	-1.5973529360	1.9229514592
H	5.6238611276	-3.9851439142	1.4182360206
C	4.8842214081	0.4937574695	0.1679960770
C	-0.4947243078	3.5613657667	-0.8986046352
O	0.5196408111	3.4323150368	1.2216081776
O	-0.4308985354	2.1909107448	-0.4532155697
C	0.5390471350	4.2742101741	0.0494188608
C	1.9591698357	4.2422446045	-0.5037007030
H	2.0712994658	4.9206172078	-1.3607617807
H	2.6547349116	4.5595313173	0.2865228404
H	2.2300524834	3.2244459551	-0.8186150693
C	0.1562875127	5.6879483745	0.4483502831
H	0.9368768516	6.1083640782	1.0983226443
H	0.0710563747	6.3278093084	-0.4422293164
H	-0.7952082008	5.7105644541	0.9943401933
C	-0.1341307227	3.6315785505	-2.3724166849
H	-0.9225811735	3.1570380448	-2.9748945667
H	-0.0460954829	4.6798117062	-2.6944432004
H	0.8126661488	3.1154480770	-2.5754158146
C	-1.9246440190	4.0438928347	-0.6777602878
H	-2.0556704057	5.0772388988	-1.0276603052
H	-2.6115081278	3.3990476003	-1.2463679370
H	-2.2009125713	3.9943462174	0.3861510497
B	0.0845339891	2.1945930956	0.8262630820
C	0.1753358181	0.9657893111	2.9937350668
C	1.5318906108	1.4821026018	3.4602503430
C	-0.9661778268	1.7652434397	3.6148873758
C	0.0206135280	-0.5192163059	3.2985138690
H	2.3428672379	0.9126447431	2.9848128104
H	1.6528565550	2.5443053966	3.2074092324
H	1.6182303472	1.3676553979	4.5506084929
H	-1.9243694447	1.3884684446	3.2285344361
H	-0.9567170583	1.6630598741	4.7096193814
H	-0.8703617234	2.8329259858	3.3663956494
H	0.0838609046	-0.7014000810	4.3808437796
H	-0.9545489555	-0.8767779949	2.9329261277
H	0.8171619538	-1.0934624833	2.7998808899
O	0.0677385239	1.0593103706	1.5623435678
K	-0.9326468718	-0.7159817040	-0.1691011247
C	5.4772282533	1.4279814387	-0.8914498929
H	6.5675178497	1.2952879425	-0.9787293238
H	5.0270329537	1.2663566254	-1.8801922250
H	5.2820429562	2.4757211906	-0.6111920888
C	5.4404617030	0.9067070190	1.5352887848
H	5.2782955463	1.9847439286	1.6888610597
H	4.9363644897	0.3839275389	2.3616266269
H	6.5214791934	0.7101620208	1.6139922918
O	-2.9399772524	-0.5080570479	-1.9499116602
C	-4.0196390946	-1.0987355150	-2.6572490310
C	-2.5901181135	0.6676825555	-2.6629085297
C	-4.8200781592	0.0702390239	-3.2746226957
H	-3.6236834409	-1.7652566187	-3.4431515506
H	-4.6038685666	-1.7055182104	-1.9516376400
C	-3.9352809355	1.3026220678	-2.9991988986
H	-1.9365569958	1.2795663274	-2.0255047186
H	-2.0356013651	0.4032924480	-3.5834146063
H	-5.8130742591	0.1730881795	-2.8174311056
H	-4.9640208629	-0.0884281987	-4.3514356246
H	-4.3076131085	1.8618711048	-2.1270026832
H	-3.8761600366	1.9929904013	-3.8506522299
O	-1.9685726282	-2.8858062061	0.9869695946
C	-1.6206625628	-4.1855790923	1.4238690534
C	-3.1265864636	-3.0489074106	0.1864691175
C	-2.9542233948	-4.8289222174	1.8194209842
H	-1.1482641046	-4.7432642769	0.5940524269
H	-0.8983883255	-4.0956121032	2.2456427305
C	-3.9769501902	-4.1057260524	0.9119051732
H	-3.6082627296	-2.0662829341	0.0832146467
H	-2.8309571104	-3.4009625743	-0.8199795492
H	-3.1699434751	-4.6361583400	2.8792541895
H	-2.9427394669	-5.9160219792	1.6692548634
H	-4.7665058166	-3.6301039118	1.5103855040
H	-4.4608924579	-4.7843334902	0.1977130125
O	-2.9502659283	-0.0074422752	1.4563473970

C	-3.6069019038	1.1023286888	0.8741798976
C	-3.8859427239	-0.6182866084	2.3284150246
C	-5.0032296439	0.5807817161	0.5381357409
H	-3.6562700795	1.9357348997	1.6020467527
H	-3.0237415099	1.4334180644	0.0029501821
C	-5.2633180528	-0.4566511285	1.6526107922
H	-3.5722111096	-1.6603661094	2.4759939036
H	-3.8685767137	-0.1026492883	3.3060400277
H	-4.9739314699	0.0937254796	-0.4460675626
H	-5.7545385089	1.3807181844	0.5069399668
H	-5.6206453842	-1.4081866609	1.2348788169
H	-6.0137167457	-0.1079173855	2.3739727814
C	1.0712230184	-3.0047812896	-0.8581748894
C	0.2088628109	-3.0320470093	-1.9700445662
C	0.2151723045	-1.8964408482	-2.8081770205
C	0.9890943290	-0.7846672430	-2.5319189701
C	1.8447181605	-0.6930613695	-1.3590021793
C	1.8593291510	-1.9056397553	-0.5550864438
H	1.1329748900	-3.8810508368	-0.2045257388
H	-0.3946800790	-3.9097915919	-2.2041514343
H	-0.4178990020	-1.8855788092	-3.7016443727
H	0.9497735878	0.0771682366	-3.2059937560
H	2.5293770401	-1.9620935518	0.3052617050
C	2.5405536666	0.4642676635	-1.0440259361
H	2.4498997555	1.3039313261	-1.7414891738
C	3.3258788089	0.6806303746	0.2099271853
H	3.1672545187	1.7124312019	0.5838545630
H	2.9500383985	0.0182577830	1.0105976773

Int3_4sol
M06-2X/def2SVP Electronic E: -2830.748082 a.u.
M06-2X/def2SVP Gibbs free E: -2829.751521 a.u.
M06-2X/def2TZVPP Electronic E: -2833.361165 a.u.

C	-6.8454760339	-0.7188893908	0.0461245303
C	-5.9772922473	-1.8006985221	0.1911168808
C	-4.8956682918	-1.7255057103	1.0678084652
C	-4.6345105536	-0.5614762531	1.8083056588
C	-5.5340414965	0.5069266639	1.6681062814
C	-6.6248885987	0.4323310645	0.8007183271
H	-7.6897398340	-0.7755963024	-0.6435530495
H	-6.1378370752	-2.7119207411	-0.3887448945
H	-4.2270621021	-2.5820124702	1.1495392849
H	-5.3801112352	1.4288888949	2.2301734471
H	-7.3016083112	1.2847318174	0.7105559758
C	-3.3635838077	-0.4290626909	2.6403434468
C	-0.0274942853	3.6357792638	1.1585348214
O	-0.2744269888	3.8984803381	-1.1523113967
O	0.1525627603	2.3674922107	0.4959702410
C	-0.7993066241	4.4579002263	0.0709024406
C	-2.2982613346	4.1775020925	0.0881988125
H	-2.7818764670	4.6312533309	0.9645823280
H	-2.7501598630	4.6025965566	-0.8192071924
H	-2.4954793529	3.0940811911	0.0928012352
C	-0.5289988205	5.9505280772	0.0933949974
H	-1.1204455205	6.4432831081	-0.6914510124
H	-0.8225780286	6.3788281070	1.0631386466
H	0.5310297401	6.1704578939	-0.0860428486
C	-0.7888045405	3.4342344745	2.4554483193
H	-0.1753706741	2.8533657908	3.1611375204
H	-1.0121728739	4.4064933531	2.9197014578
H	-1.7277052312	2.8927614930	2.2911954809
C	1.3595159495	4.2056177440	1.4408403572
H	1.2976443066	5.1492467905	2.0006293993
H	1.9262164980	3.4820696594	2.0453560794
H	1.9125650576	4.3872188636	0.5070649396
B	0.1299255174	2.6190793038	-0.8599120401
C	0.6538488756	1.8419662806	-3.1602431924
C	-0.6835806108	2.2671529366	-3.7538876615
C	1.7493647147	2.8704451708	-3.4183899991
C	1.0632768877	0.4759192809	-3.6993159057
H	-1.4619543907	1.5461541568	-3.4619821595
H	-0.9714168434	3.2662117052	-3.4004339016
H	-0.6175487449	2.2863197859	-4.8516429414
H	2.6877663208	2.5276335706	-2.9577961068
H	1.9105647592	2.9951219789	-4.4990006295
H	1.4736673593	3.8463711694	-2.9931473910
H	1.2123025880	0.5246119976	-4.7873295003
H	2.0038345628	0.1523674823	-3.2282907974
H	0.2716061181	-0.2613771975	-3.4922873881
O	0.5191992394	1.6659917102	-1.7361326614
K	1.0595568682	-0.7053676033	-0.5477644365
C	-2.9651697488	-1.7646274986	3.2758669201
H	-3.7743520895	-2.1709826589	3.9035961465
H	-2.7032066770	-2.5139894721	2.5163475361
H	-2.0771323589	-1.6211833813	3.9127811390
C	-3.5256262697	0.6046938381	3.7603896968
H	-2.6168050970	0.6212889632	4.3820435586
H	-3.6650118659	1.6223273746	3.3662294182
H	-4.3809279219	0.3710333016	4.4142659054
O	2.3264402789	-0.6545416954	1.8470538244
C	3.3016070874	-1.2598543781	2.6813719557
C	1.5741394278	0.2168030624	2.6787690446
C	3.6637188961	-0.2110803154	3.7564423905
H	2.8775219412	-2.1662472224	3.1496241714
H	4.1515463690	-1.5595087209	2.0545203968
C	2.6362866214	0.9135111867	3.5234726562
H	0.9796825171	0.8851477076	2.0388404332
H	0.8875235582	-0.3720017305	3.3183965486
H	4.6943700936	0.1509500784	3.6455923792
H	3.5680471583	-0.6415861625	4.7620195654
H	3.0876539096	1.7319524798	2.9423355623
H	2.2321817364	1.3328670353	4.4540954257
O	3.0257549347	-2.4119405542	-1.1346948913
C	2.8280467323	-3.5666907186	-1.9276191290
C	4.3189179579	-2.5395502518	-0.5793122569
C	4.1526290793	-3.7390061505	-2.6756347862
H	2.6189713750	-4.4357713122	-1.2752796245
H	1.9573032405	-3.4023532881	-2.5762153893
C	5.1912151999	-3.1578983864	-1.6886019812
H	4.6418565799	-1.5467967264	-0.2375612041
H	4.2823414568	-3.2152978016	0.2964332204
H	4.1340863943	-3.1553693717	-3.6064757631
H	4.3471212336	-4.7872380474	-2.9360690752
H	5.8207854893	-2.3991160307	-2.1732776835
H	5.8564116455	-3.9297460011	-1.2801884025
O	3.3000555909	0.6989271870	-1.1989961136
C	3.4278207129	1.7013779109	-0.2105969165
C	4.5586314796	0.5868363815	-1.8383715342
C	4.7773197823	1.4016327672	0.4340919727
H	3.4234128600	2.7038798926	-0.6833080315
H	2.5696398046	1.6336234727	0.4721445400
C	5.6199373059	0.9263599774	-0.7669255678
H	4.6371773784	-0.4299335667	-2.2472892003
H	4.6145634488	1.3014371594	-2.6797560584
H	4.6430450564	0.5895320814	1.1637975080
H	5.2091311894	2.2684393556	0.9518545695
H	6.2422433639	0.0574157811	-0.5125649026
H	6.2898432277	1.7190166524	-1.1256699699
C	-3.6750837003	0.3272299252	-2.4788115420
C	-3.2002583178	-0.5293641410	-3.4881196112
C	-2.2143877926	-1.4572511504	-3.1170184110
C	-1.7325499969	-1.5424232783	-1.8193075760
C	-2.2203263488	-0.7010433424	-0.7437032701
C	-3.2121253028	0.2651253374	-1.1749155506
H	-4.4444033559	1.0683485921	-2.7202778994
H	-3.5771786648	-0.4732595557	-4.5099366159
H	-1.8051855605	-2.1398279258	-3.8697434285
H	-0.9779620091	-2.3003140380	-1.5885084588

H	-3.6593564386	0.9295230574	-0.4327301219
C	-1.7710145489	-0.8236072905	0.5732983125
H	-1.0758095154	-1.6388414489	0.8008622696
C	-2.2014233495	0.0596766740	1.7016206573
H	-1.3481060765	0.2580698964	2.3788669673
H	-2.4994698622	1.0514545793	1.3172769413
O	0.4316295548	-3.3718955151	0.0922087366
C	-0.6272342676	-4.3295435342	0.0293540129
C	1.2046487880	-3.5756460126	1.2673319783
C	-0.4880386835	-5.2049716126	1.2774960304
H	-1.5948311671	-3.8048074154	-0.0125562277
H	-0.5108075788	-4.9147558066	-0.8981406426
C	0.9900326903	-5.0375589101	1.6297138131
H	2.2452938770	-3.3064505768	1.0403712435
H	0.8446859540	-2.9111975168	2.0766427103
H	-0.7850347387	-6.2454375931	1.0933786298
H	-1.1126278995	-4.8077608112	2.0914854173
H	1.6160107278	-5.6808935630	0.9916054706
H	1.2201241564	-5.2562128806	2.6808228927

Int4_3sol

M06-2X/def2SVP Electronic E: -2598.601657 a.u.

M06-2X/def2SVP Gibbs free E: -2597.710008 a.u.

M06-2X/def2TZVPP Electronic E: -2600.941236 a.u.

C	4.3354185176	-4.0768688271	-1.1697899785
C	4.2532901173	-3.1020282547	-2.1617843539
C	4.4680857347	-1.7575918326	-1.8512518307
C	4.7480580319	-1.3470338883	-0.5410693343
C	4.8399555024	-2.3455401665	0.4416656939
C	4.6387564723	-3.6903863704	0.1363603483
H	4.1680100929	-5.1282015454	-1.4113871239
H	4.0193647718	-3.3853988192	-3.1901489730
H	4.3895060888	-1.0209411957	-2.6509886568
H	5.0521585143	-2.0694600870	1.4763586539
H	4.7131627328	-4.4407308388	0.9263822965
C	4.8832988826	0.1285461421	-0.1502246922
C	0.1361695079	3.3541259281	-0.5155552509
O	1.6569450161	2.8372775185	1.2422553953
O	0.0215531432	1.9913828784	-0.1707964164
C	1.3507107864	3.8984709787	0.3744204416
C	2.6039917352	4.2538428655	-0.4405262710
H	2.4358691551	5.1047576699	-1.1166933175
H	3.4035491607	4.5299047497	0.2636631447
H	2.9624892909	3.4036259448	-1.0346190853
C	0.9927426869	5.1271643063	1.2131547332
H	1.8521597699	5.3827114825	1.8510027137
H	0.7674585634	5.9978224128	0.5790841077
H	0.1312043954	4.9425235246	1.8673098630
C	0.3970421683	3.4938319564	-2.0197175119
H	-0.4584616697	3.0905794605	-2.5826968484
H	0.5210977300	4.5476974534	-2.3114310958
H	1.2953394758	2.9420768237	-2.3270085930
C	-1.2024323359	4.0313738525	-0.2059346030
H	-1.1862890577	5.1089181817	-0.4258773430
H	-1.9834332354	3.5720464735	-0.8332313927
H	-1.4841969652	3.8860977544	0.8454154120
B	1.1561483916	1.5682885905	0.7150722598
C	0.2437830687	0.9483429058	3.0328938116
C	1.4298934993	1.3394744472	3.9195118954
C	-0.7967845615	2.0717984721	3.0125379009
C	-0.4080255216	-0.3255565395	3.5798780800
H	2.1592510538	0.5149303318	3.9512402776
H	1.9219953392	2.2272594480	3.5010979643
H	1.1050624083	1.5546995839	4.9496511882
H	-1.6401379485	1.7936150928	2.3623137786
H	-1.1880650609	2.2518873103	4.0264872418
H	-0.3430468809	2.9964889481	2.6362516282
H	-0.7105786066	-0.1998085292	4.6306360503
H	-1.3099002042	-0.5829432671	2.9998360875
H	0.2997629066	-1.1664076078	3.5171336638
O	0.7035079783	0.6242564998	1.7381189817
K	-1.2203358952	-0.3912731593	0.1580352520
C	5.1409472388	1.0174693052	-1.3709365916
H	6.0690344036	0.7227023035	-1.8860591490
H	4.3223068071	0.9846567600	-2.1024500727
H	5.2506575590	2.0636303569	-1.0450975577
C	6.0721299621	0.3081956390	0.8085470152
H	6.2294632032	1.3789873411	1.0092458438
H	5.8997072618	-0.1816763135	1.7776784027
H	6.9995473956	-0.1003204684	0.3772664086
O	-2.8537795602	-0.2090815194	-1.9696616931
C	-3.8514981914	-0.6693672897	-2.8680638224
C	-2.3067757710	0.9663168015	-2.5477916929
C	-4.4529515048	0.5923027638	-3.5269559621
H	-3.3897619377	-1.3274462000	-3.6248621118
H	-4.5834479713	-1.2585288746	-2.2984241970
C	-3.5285192691	1.7298224882	-3.0479404017
H	-1.7064429228	1.4863323919	-1.7862037696
H	-1.6404986900	0.6989970799	-3.3908133202
H	-5.4935962815	0.7581867154	-3.2186101851
H	-4.4408278749	0.4976817005	-4.6207505787
H	-3.9889995440	2.2809382680	-2.2139371763
H	-3.2811690911	2.4486924341	-3.8396607819
O	-2.4371910261	-2.6206732835	0.9127826405
C	-2.2055370807	-3.9212401653	1.4193550395
C	-3.5457252050	-2.7336263223	0.0364109721
C	-3.6021383567	-4.4616570447	1.7367804009
H	-1.7115392457	-4.5413520185	0.6478981506
H	-1.5407880362	-3.8428596264	2.2893602006
C	-4.5002410929	-3.7398930602	0.7058540390
H	-3.9679740025	-1.7283719153	-0.1061016409
H	-3.2026704061	-3.1093410772	-0.9456988978
H	-3.8883402577	-4.1871256439	2.7614201611
H	-3.6479008905	-5.5550414831	1.6542065139
H	-5.3345973558	-3.2252550632	1.2017448091
H	-4.9262149533	-4.4300637481	-0.0337708296
O	-3.4223958770	0.3417989547	1.4108622603
C	-3.9816281515	1.4511518488	0.7325571218
C	-4.4764442048	-0.2744945120	2.1324006423
C	-5.3034099057	0.9199954330	0.1863378405
H	-4.1439402050	2.2875762326	1.4389196235
H	-3.2713826012	1.7804491979	-0.0397687701
C	-5.7494459927	-0.0713223706	1.2822252800
H	-4.2002039356	-1.3264886352	2.2879272095
H	-4.5802290017	0.2112022378	3.1184181263
H	-5.1082196354	0.3939190319	-0.7588875270
H	-6.0354385671	1.7159355056	-0.0028284898
H	-6.1047549867	-1.0162212520	0.8485196083
H	-6.5629573170	0.3394774305	1.8943818774
C	0.9236914807	-2.8129260235	-0.3947663103
C	0.2824731340	-2.8080785718	-1.6393137658
C	0.3494721297	-1.6577191846	-2.4269802113
C	1.0456675290	-0.5330837757	-1.9751951448
C	1.6868862367	-0.5127546177	-0.7232625484
C	1.6032376104	-1.6837870916	0.0562482084
H	0.8863607509	-3.7052133958	0.2349512170
H	-0.2547090216	-3.6913057494	-1.9906219851
H	-0.1428772736	-1.6341875231	-3.4021500460
H	1.0862329506	0.3638363050	-2.5994440486
H	2.0720151998	-1.6977751761	1.0403136803
C	2.3035717403	0.7477665854	-0.2051914976
H	2.4821200701	1.4049233687	-1.0700145633
C	3.6001212382	0.6031007087	0.5913658744
H	3.8233884951	1.5964138048	1.0180955574
H	3.4354580521	-0.0596000578	1.4592564222

Int4'_3sol

M06-2X/def2SVP Electronic E: -2907.883850 a.u.

M06-2X/def2SVP Gibbs free E: -2906.865682 a.u.
M06-2X/def2TZVPP Electronic E: -2910.569778 a.u.

C	-6.6076964471	3.8585085987	0.3188523383
C	-5.2964117045	4.3182829028	0.2397143700
C	-4.2261780739	3.4422358666	0.4481999609
C	-4.4413627629	2.0885936228	0.7339764670
C	-5.7715176077	1.6414183107	0.8029809558
C	-6.8405296113	2.5108235252	0.6036845227
H	-7.4438640358	4.5413713161	0.1573754817
H	-5.0963808494	5.3679807743	0.0148783705
H	-3.2116269831	3.8357071004	0.3804202439
H	-5.9846879165	0.5876515189	1.0008918741
H	-7.8611451857	2.1270724557	0.6599372320
C	-3.2974225354	1.0994980119	0.9850314421
C	-3.4595661428	0.5115225055	2.3970085156
H	-3.4651447580	1.3080560339	3.1572702625
H	-4.3958851622	-0.0581551407	2.4906797525
H	-2.6265232237	-0.1736073190	2.6226764163
C	-1.9304520776	1.7774660437	0.9022781483
H	-1.1325912220	1.0395956809	1.0730353665
H	-1.7562303581	2.2371248280	-0.0816304334
H	-1.8242107042	2.5636324121	1.6674245038
C	0.0094820191	-1.7248736974	-2.4945279242
C	0.8318928225	-0.7535381223	-3.0736926533
C	0.4239884442	0.5824041488	-3.0574985681
C	-0.8102450395	0.9344910960	-2.5026272533
C	-1.6576336584	-0.0301735488	-1.9370769154
C	-1.2092631280	-1.3626611951	-1.9199910372
H	0.3177395517	-2.7728169084	-2.4914261808
H	1.7861331481	-1.0335735572	-3.5240891087
H	1.0614145477	1.3537960113	-3.4947422788
H	-1.1357362200	1.9778683576	-2.5280340209
H	-1.8436014196	-2.1361399822	-1.4775143441
C	-3.0695814120	0.3031481306	-1.4966146329
H	-3.2133552228	1.3900833645	-1.6197787308
C	-3.3686120307	-0.0652380262	-0.0313391843
H	-4.3744487872	-0.5054256442	0.0260389889
H	-2.6630893402	-0.8442946570	0.3063131631
C	-8.7924561522	-1.0611281108	-0.7827427589
C	-7.8299620344	-0.3285202296	-1.4507962823
C	-6.4613842866	-0.7856339242	-1.6161810745
C	-6.2100640631	-2.0763938909	-1.0015207254
C	-7.1983744557	-2.7919734534	-0.3405721501
C	-8.5123281720	-2.3172837804	-0.2083793327
H	-9.8030611049	-0.6464504889	-0.7033106653
H	-8.0984792249	0.6412752750	-1.8822470926
H	-5.2064778913	-2.5061201885	-1.0569082460
H	-6.9329992060	-3.7618099452	0.0941973138
H	-9.2777223534	-2.8896050190	0.3167624472
C	-5.5153782156	-0.0249349846	-2.2929735045
H	-5.8334767177	0.9539622634	-2.6705479538
C	-4.0751207947	-0.3859024018	-2.4790503546
H	-3.7317105130	-0.1292134127	-3.4988337084
H	-3.9290414589	-1.4770224511	-2.3894903376
C	4.7654684641	2.2941039192	-0.9900814883
O	5.7855360055	0.2677907707	-1.5922110369
O	3.7967578850	1.2191394718	-0.9538532979
C	5.9000388385	1.6797192425	-1.8834938737
C	5.6252575466	1.8511528190	-3.3736489057
H	5.7559665948	2.8958444262	-3.6870064776
H	6.3312853040	1.2268287789	-3.9389318112
H	4.6026190315	1.5335518590	-3.6282374141
C	7.3005739202	2.1492889340	-1.5397898205
H	8.0226064778	1.6814472730	-2.2239272465
H	7.3767994703	3.2404065929	-1.6549133569
H	7.5753572921	1.8799277595	-0.5123593460
C	4.1264709311	3.5410321455	-1.5733405566
H	3.3926345853	3.9553824452	-0.8673331171
H	4.8929070991	4.3093774782	-1.7523802385
H	3.6140942056	3.3257005807	-2.5193797863
C	5.2274091246	2.5462570018	0.4408291505
H	5.9313617740	3.3885959179	0.4858298233
H	4.3557005045	2.7931591909	1.0645741450
H	5.7171394089	1.6567564234	0.8638078234
B	4.4897806767	0.0539445600	-1.2112391870
C	4.4313680516	-2.4425569921	-1.2118231164
C	4.9421985107	-2.6360058499	-2.6345377596
C	5.5499217167	-2.5863012099	-0.1870300312
C	3.2910928476	-3.4021869837	-0.8967288053
H	4.1263000416	-2.4856481627	-3.3571203077
H	5.7493169141	-1.9245730958	-2.8576002637
H	5.3308034886	-3.6567344313	-2.7595165700
H	5.1456051050	-2.4145174177	0.8211899555
H	5.9745556679	-3.5994522242	-0.2267730697
H	6.3545920258	-1.8627477884	-0.3808476088
H	3.6381678322	-4.4442869990	-0.9393242628
H	2.8978346899	-3.1956191740	0.1109209467
H	2.4747241042	-3.2775082796	-1.6250646542
O	3.8487075931	-1.1305132117	-1.0861785508
K	1.4931537138	-0.4503553073	-0.0938521944
O	1.1561429979	1.4237051916	1.6638850629
C	1.0257407407	1.3934803538	3.0783058480
C	1.3260710936	2.7693412888	1.2110734703
C	1.7912883558	2.6176903957	3.5537792484
H	-0.0412207105	1.4565682337	3.3637655350
H	1.4267478057	0.4339523760	3.4346270521
C	1.4227174547	3.6362175638	2.4719108738
H	2.2418997969	2.8017254357	0.6020967292
H	0.4775695503	3.0550053501	0.5700353965
H	2.8738034715	2.4119042760	3.5428860050
H	1.5073767992	2.9340774014	4.5657676210
H	2.1565220606	4.4458667053	2.3659668389
H	0.4465449027	4.0880053131	2.7004611103
O	0.3382246034	-2.6209134006	0.8825540677
C	-0.0733486238	-3.9440090253	0.5406357575
C	-0.4763728382	-2.0984618768	1.9324976460
C	-0.9775257347	-4.3940152096	1.6816891023
H	-0.6231538868	-3.9222033200	-0.4166443167
H	0.8202952241	-4.5724951521	0.4144022856
C	-1.6383448861	-3.0747726943	2.0820941967
H	0.1203485831	-2.0356080435	2.8602323391
H	-0.8016026385	-1.0790516670	1.6657652455
H	-0.3736538536	-4.7863365612	2.5144954118
H	-1.6916525415	-5.1682156015	1.3738400159
H	-2.0564519344	-3.0782204531	3.0968742023
H	-2.4452043486	-2.8227532362	1.3761557603
O	2.9315757126	-1.2783740764	1.9784433392
C	3.8675714468	-0.3333379186	2.4739906058
C	2.9849228488	-2.4012735602	2.8399604833
C	3.9003692026	-0.5208019338	4.0078609536
H	4.8625562402	-0.5320373748	2.0362621008
H	3.5377591952	0.6637528279	2.1473635049
C	3.0560295623	-1.7915444279	4.2371949385
H	2.1003175009	-3.0219809301	2.6450957728
H	3.8918753031	-2.9992548281	2.6278997481
H	3.4833498705	0.3453280563	4.5392026594
H	4.9325275116	-0.6566423221	4.3565023507
H	2.0434104140	-1.5324198387	4.5803863963
H	3.4993010369	-2.4749768561	4.9725392682

Int4_4sol
M06-2X/def2SVP Electronic E: -2830.774370 a.u.
M06-2X/def2SVP Gibbs free E: -2829.773580 a.u.
M06-2X/def2TZVPP Electronic E: -2833.381401 a.u.

C	-6.8143392414	-1.7046550519	-0.6656312996
C	-5.6980743997	-2.5079739880	-0.4434458116
C	-4.6812189916	-2.0834650864	0.4152922043
C	-4.7415037725	-0.8406157062	1.0576474932

C	-5.8826970209	-0.0531051071	0.8347786583
C	-6.9050767342	-0.4747997821	-0.0120909425
H	-7.6079432786	-2.0339124551	-1.3388988368
H	-5.6108822826	-3.4748423574	-0.9435344604
H	-3.8181347561	-2.7336235547	0.5635712768
H	-5.9680315346	0.9245072672	1.3136417705
H	-7.7752006375	0.1659684426	-0.1697837009
C	-3.6027545186	-0.3034119471	1.9289993476
C	0.1776028645	3.2025549206	1.2457932261
O	-1.3153964022	3.0896535600	-0.5825905177
O	-0.0688604803	1.8475978221	0.9461764424
C	-0.9494904917	3.9752267598	0.4510784376
C	-2.1860987819	4.2799452899	1.3067398483
H	-1.9942779940	5.0681112017	2.0498128192
H	-2.9877765039	4.6251687627	0.6364263606
H	-2.5451702936	3.3864644628	1.8302166994
C	-0.4707064416	5.2968335719	-0.1493056574
H	-1.2966950408	5.7636814056	-0.7071472554
H	-0.1538498124	5.9979732173	0.6385767809
H	0.3683670001	5.1539111461	-0.8413172893
C	0.1004433641	3.4277121937	2.7552400501
H	0.9475528274	2.9337947151	3.2540468549
H	0.1522008608	4.5006033867	2.9974903036
H	-0.8290661054	3.0196957772	3.1747840267
C	1.5954784745	3.5477047213	0.7804422023
H	1.8922105544	4.5751539770	1.0414018400
H	2.2908740845	2.8506831704	1.2750678854
H	1.6930952364	3.4091507858	-0.3026616338
B	-0.8522585854	1.7264743716	-0.3255045609
C	0.2179325007	1.6402185368	-2.7333683064
C	-1.0828056868	2.0372442137	-3.4411984672
C	1.1855807762	2.8275550766	-2.7576237308
C	0.8602628458	0.4654425450	-3.4782614807
H	-1.7552601910	1.1739652388	-3.5404128361
H	-1.5965929099	2.8199195803	-2.8679066040
H	-0.8599372171	2.4185382609	-4.4504017097
H	2.1226222978	2.5546721953	-2.2464697185
H	1.4236944867	3.1277682106	-3.7901015835
H	0.7326071127	3.6826180751	-2.2384272175
H	1.0823185893	0.7227107333	-4.5254628144
H	1.8044110910	0.1770182719	-2.9865469353
H	0.1816889208	-0.4007523162	-3.4662207771
O	0.0193036470	1.1944341509	-1.4126995406
K	1.4474455787	-0.2921389614	0.1198994918
C	-2.6030974725	-1.4000872674	2.3098694012
H	-3.0954033886	-2.2029972440	2.8820982836
H	-2.1089452796	-1.8467221801	1.4367623637
H	-1.8129000502	-0.9656339990	2.9435428125
C	-4.1807356800	0.2666236585	3.2358295151
H	-3.3609346829	0.5757891107	3.9027822700
H	-4.8083226326	1.1519219209	3.0576058207
H	-4.7891607708	-0.4836186881	3.7650569446
O	2.7635733928	-0.4477301977	2.3936220387
C	4.0398089646	-0.7604737236	2.9227273848
C	2.0587090251	0.2644384013	3.3966018323
C	4.4039295814	0.3948720895	3.8804804425
H	3.9912985975	-1.7213772183	3.4655707958
H	4.7366255910	-0.8730160613	2.0807084777
C	3.1024225605	1.2184233285	3.9724229679
H	1.1969581710	0.7598851131	2.9239137797
H	1.6922832579	-0.4345371371	4.1723659865
H	5.2365793485	0.9977117021	3.4931406914
H	4.7056448172	0.0043681026	4.8611816255
H	3.1658778797	2.1189468413	3.3430520702
H	2.8633614383	1.5360761375	4.9955746216
O	2.9830624512	-2.2604061562	-0.7711116731
C	2.5088242664	-3.3575678218	-1.5271893745
C	4.3132931279	-2.5749580612	-0.4073259556
C	3.6801309135	-3.7289553336	-2.4422399573
H	2.2524597746	-4.1949329095	-0.8497666788
H	1.5997404019	-3.0476572208	-2.0593511352
C	4.9194717855	-3.2833109363	-1.6320782566
H	4.8161535857	-1.6392361405	-0.1286962204
H	4.3084524597	-3.2482216884	0.4708358251
H	3.6113045894	-3.1703579155	-3.3858177896
H	3.6879759771	-4.7995364741	-2.6836239149
H	5.5449336620	-2.5943958140	-2.2163274761
H	5.5494584466	-4.1295055088	-1.3287634162
O	3.8466236236	0.6863664407	-0.5657730309
C	4.6466887026	1.5334903287	0.2356335074
C	4.5527806719	0.4717745377	-1.7765654223
C	6.0695614513	1.0091585775	0.0435344745
H	4.5612955786	2.5802715693	-0.1127709134
H	4.2765179888	1.4736442937	1.2686905589
C	6.0515483769	0.4835152850	-1.4074977946
H	4.2053168623	-0.4814319116	-2.1992156477
H	4.3201752564	1.2801157019	-2.4921488270
H	6.2668326888	0.1908493420	0.7518344225
H	6.8254030655	1.7876603063	0.2087424478
H	6.4927203670	-0.5203592243	-1.4761270904
H	6.6134035946	1.1389937039	-2.0855101691
C	-4.4932902240	0.3487331786	-2.9009780401
C	-3.9900711542	-0.7183027796	-3.6492684292
C	-2.8446575344	-1.3773250735	-3.1991093983
C	-2.2222722546	-0.9728937252	-2.0180263030
C	-2.7162849159	0.0929903890	-1.2470350523
C	-3.8630422018	0.7486862127	-1.7231102246
H	-5.3886540395	0.8761033864	-3.2389448174
H	-4.4863979662	-1.0325626198	-4.5694599242
H	-2.4326893978	-2.2119536263	-3.7720272487
H	-1.3191375802	-1.4844445056	-1.6739782414
H	-4.2730417912	1.5893443655	-1.1586993507
C	-1.9888764951	0.5296760332	-0.0120390613
H	-1.3286336608	-0.3105857014	0.2708665650
C	-2.8641851664	0.8549077155	1.1990521081
H	-2.2017385929	1.3164139378	1.9491820228
H	-3.6133598496	1.6225082642	0.9385362264
O	0.1203231928	-2.6875730905	0.4153549304
C	-0.7855040038	-3.6961560421	-0.0260990590
C	0.8414456830	-3.1341377785	1.5656835860
C	-0.8882552583	-4.6873863127	1.1262549445
H	-1.7446185320	-3.2249974695	-0.2858147663
H	-0.3834288828	-4.1811384450	-0.9339859102
C	0.5234854617	-4.6197732957	1.7072948448
H	1.9116636590	-2.9238908011	1.4165132465
H	0.4965667003	-2.5679351581	2.4490245389
H	-1.1838926343	-5.6912732980	0.7951069596
H	-1.6220389690	-4.3321697556	1.8666276910
H	1.2135896123	-5.2205583775	1.0950490581
H	0.5917904508	-4.9662539914	2.7465961544

Int4'_4sol

M06-2X/def2SVP Electronic E: -3140.064002 a.u.

M06-2X/def2SVP Gibbs free E: -3138.936368 a.u.

M06-2X/def2TZVPP Electronic E: -3143.017470 a.u.

C	-7.2835203242	3.4468679284	-1.2797982910
C	-5.9763105099	3.8218544585	-1.5765785259
C	-4.8974606536	3.1551527337	-0.9866384528
C	-5.0992247353	2.0993719927	-0.0891953972
C	-6.4263803182	1.7358497020	0.1988163467
C	-7.5038944471	2.3969728009	-0.3849727423
H	-8.1259404237	3.9645738559	-1.7422308361
H	-5.7859899980	4.6392055964	-2.2752781564
H	-3.8865826056	3.4729349515	-1.2435035494
H	-6.6314686130	0.9069367992	0.8814144045
H	-8.5208762522	2.0808225453	-0.1439850963
C	-3.9441911631	1.3428490209	0.5798330460
C	-4.0583775169	1.5141886491	2.1038619413

H	-4.0379130242	2.5785334074	2.3848510599
H	-4.9909505806	1.0753625694	2.4883999747
H	-3.2165140911	1.0094951877	2.6054664714
C	-2.5808937987	1.8828645741	0.1394548461
H	-1.7744887727	1.2911477711	0.6004825595
H	-2.4463047832	1.8317868619	-0.9501277541
H	-2.4551366061	2.9324579102	0.4497623401
C	-0.4417397785	-2.3726489259	-1.0702856123
C	0.2674453912	-1.7325468633	-2.0895387959
C	-0.3439429388	-0.7033984951	-2.8076978762
C	-1.6525869082	-0.3227600941	-2.5111278963
C	-2.3767719947	-0.9466318269	-1.4863314681
C	-1.7485801409	-1.9815530956	-0.7762892558
H	0.0249929473	-3.1843332345	-0.5056550573
H	1.2887066173	-2.0402771216	-2.3304246909
H	0.1994329929	-0.1955431792	-3.6067215469
H	-2.1264162380	0.4789845419	-3.0839218627
H	-2.2955234387	-2.4932290486	0.0204846479
C	-3.8175601336	-0.5736103736	-1.2014263888
H	-4.0731560615	0.2841254252	-1.8474288091
C	-4.0639728556	-0.1659996895	0.2609588931
H	-5.0740177531	-0.4904147508	0.5462204799
H	-3.3680508257	-0.7112340226	0.9217212343
C	-9.4376753524	-1.4408138181	0.3194110147
C	-8.5072639121	-1.1799860901	-0.6682083169
C	-7.1367686347	-1.6571898657	-0.6086900544
C	-6.8439119095	-2.4136469810	0.5950747788
C	-7.8006937847	-2.6633479198	1.5682995825
C	-9.1194387859	-2.1935047476	1.4679131862
H	-10.4524962209	-1.0468290736	0.1984961029
H	-8.8037548687	-0.5905651895	-1.5420451781
H	-5.8344117079	-2.8056120257	0.7436196156
H	-7.5061130150	-3.2478457100	2.4467778736
H	-9.8595973201	-2.3951055981	2.2429571059
C	-6.2265312271	-1.3786851105	-1.6203671233
H	-6.5783869917	-0.7582305886	-2.4533428247
C	-4.7714141831	-1.7349059255	-1.6240419717
H	-4.4461706429	-2.0571586024	-2.6308515483
H	-4.5672490731	-2.5951925046	-0.9611354328
C	1.8831795473	3.2654470553	0.8174469930
O	2.4149005149	3.5700067851	-1.4443024598
O	1.8097960879	2.0037891839	0.1157019137
C	1.7788207265	4.2908155333	-0.3621517246
C	0.3348213859	4.5467444216	-0.7795685164
H	-0.1963456126	5.1510115603	-0.0311160297
H	0.3310304125	5.0919564463	-1.7338339980
H	-0.2098128062	3.6002772630	-0.9178666010
C	2.5154570463	5.5956965916	-0.1309207994
H	2.3754326232	6.2564002665	-0.9981977715
H	2.1164518040	6.1071166824	0.7572391009
H	3.5915331072	5.4312445818	0.0070678663
C	0.7573964690	3.3567817331	1.8291664961
H	0.9333660298	2.6446091593	2.6486150765
H	0.7182144456	4.3675022605	2.2614647931
H	-0.2129448566	3.1276440466	1.3718757386
C	3.2396079322	3.3104509674	1.5131140781
H	3.3398218682	4.2083713588	2.1385949927
H	3.3330332227	2.4238540682	2.1577969282
H	4.0623408896	3.2971678769	0.7824135216
B	2.2756583213	2.2387208431	-1.1645264564
C	3.2286235203	1.2557405687	-3.2383078584
C	2.3994805697	2.0777558223	-4.2183903516
C	4.6099032328	1.8645598966	-3.0234067249
C	3.3562825439	-0.1882959397	-3.7043843920
H	1.3853565981	1.6608299387	-4.3041998328
H	2.3244031187	3.1222046149	-3.8870131169
H	2.8677273433	2.0580450295	-5.2128522025
H	5.1690271496	1.2499588358	-2.3032659952
H	5.1658497266	1.8933963599	-3.9712722613
H	4.5295251293	2.8906009051	-2.6362194055
H	3.9298390916	-0.2393079418	-4.6406322343
H	3.8782505916	-0.7827780340	-2.9383814108
H	2.3648390390	-0.6291516995	-3.8816612703
O	2.5525683368	1.1841733202	-1.9659400247
K	2.3388796150	-0.6699589732	0.0429835435
O	3.4377267139	-0.6813539229	2.4956826402
C	4.6114277542	-1.1282296188	3.1486320113
C	2.6409720053	-0.0436449065	3.4849731946
C	4.9958607036	0.0246898663	4.0733478326
H	4.3963391215	-2.0467740464	3.7270467792
H	5.3670623741	-1.3629453681	2.3860374101
C	3.6275372329	0.6168342055	4.4696008837
H	1.9720974011	0.6635415377	2.9760686594
H	2.0158713768	-0.7954489592	3.9977547736
H	5.5938377735	0.7630326874	3.5184421034
H	5.5867068512	-0.3111977388	4.9349550445
H	3.6221980616	1.7111840982	4.3787781669
H	3.3615062255	0.3696885629	5.5055480320
O	2.9165876276	-3.2361420579	-0.4114145049
C	2.6582300302	-4.4308037184	-1.1323894556
C	3.9265027678	-3.5501750455	0.5424116081
C	4.0443721603	-5.0121375435	-1.3898282552
H	2.0484991868	-5.1171834611	-0.5148647161
H	2.0959270657	-4.1798699272	-2.0411442345
C	4.8146056392	-4.6306206748	-0.1095571421
H	4.4552050477	-2.6180026312	0.7845488758
H	3.4555627422	-3.9334388793	1.4648930304
H	4.4876320641	-4.5269755384	-2.2714635417
H	4.0187107452	-6.0939815560	-1.5713063221
H	5.8188641268	-4.2489964738	-0.3390181491
H	4.9307441160	-5.4901151388	0.5633880937
O	0.5744695405	-1.4148961222	1.8578456030
C	0.7821631058	-2.7602668039	2.2695965252
C	-0.6550961088	-0.9131279438	2.3768215292
C	-0.5972739970	-3.2747960893	2.6574547765
H	1.2470447756	-3.3104629204	1.4377419217
H	1.4748164883	-2.7843939163	3.1321803499
C	-1.2229725579	-2.0191050146	3.2673013924
H	-0.4652660289	0.0234789727	2.9256482903
H	-1.3235732754	-0.6909728542	1.5298352395
H	-0.5546727489	-4.1249175399	3.3504975453
H	-1.1512712245	-3.5846677615	1.7580455801
H	-0.8782517666	-1.8927825690	4.3048435537
H	-2.3212314218	-2.0313573354	3.2692864857
O	4.9788519599	-0.4501740059	-0.3826027801
C	5.6980455244	0.5199193718	0.3718408102
C	5.9241336095	-1.2705175838	-1.0492502463
C	7.0828012826	-0.0961065644	0.6706174555
H	5.8000356136	1.4484678478	-0.2156718182
H	5.1059389167	0.7412747347	1.2708631746
C	7.0297175694	-1.4703550382	-0.0201085749
H	5.4155334122	-2.1929276682	-1.3642159209
H	6.3106089715	-0.7555145884	-1.9494454436
H	7.2736883725	-0.1819931986	1.7490101378
H	7.8786615567	0.5264843272	0.2408254242
H	6.7337517883	-2.2577251346	0.6908758171
H	7.9854879280	-1.7616315530	-0.4740252440

TS1_2_3sol

M06-2X/def2SVP Electronic E: -2289.235919 a.u.

M06-2X/def2SVP Gibbs free E: -2288.478064 a.u.

M06-2X/def2TZVPP Electronic E: -2291.240245 a.u.

C	-0.8478765275	2.6084850227	2.7803425623
C	-0.3966870949	3.3648568332	1.6826994406
C	0.8646626139	3.1845649898	1.1362054685
C	1.8023745842	2.2103994228	1.6524425982
C	1.3162418010	1.4767458997	2.8022332829
C	0.0469389094	1.6716126916	3.3270382623

H	-1.8369441803	2.7666317305	3.2116717305
H	-1.0584275707	4.1156701628	1.2401350011
H	1.1642095192	3.8091417174	0.2931046988
H	1.9665710618	0.7365397236	3.2714134278
H	-0.2596922646	1.0780791942	4.1943476725
C	3.0786989532	2.0034198957	1.1119174058
C	2.5646504379	-2.0065008404	0.1367715329
O	3.3817319568	-0.3592795599	-1.3538105855
O	1.9089040722	-0.7514883673	0.3617188759
C	3.7757108628	-1.6268305743	-0.8109518310
C	5.0975231205	-1.4457971386	-0.0754866499
H	5.4841669066	-2.4069437716	0.2915668249
H	5.8322605559	-1.0203786181	-0.7744430278
H	4.9915083734	-0.7595803570	0.7702961668
C	3.9832721987	-2.5992796131	-1.9657175724
H	4.8250573728	-2.2527221747	-2.5824871634
H	4.2273480253	-3.6031960096	-1.5882340507
H	3.0964676534	-2.6716911851	-2.6079363572
C	2.9814357125	-2.6128134126	1.4669919841
H	2.0845566480	-2.8356846083	2.0641491420
H	3.5269145164	-3.5546528454	1.3073756123
H	3.6181061007	-1.9312751750	2.0445094055
C	1.5407849451	-2.9182935003	-0.5360727221
H	1.9416498068	-3.9248237149	-0.7212382246
H	0.6692814862	-3.0071682915	0.1298365523
H	1.1991259000	-2.4920326849	-1.4899557600
B	2.2958244118	0.1284595677	-0.6513796851
C	1.1131925801	1.1823753897	-2.5769626330
C	2.2671767234	1.8712979067	-3.2981646035
C	0.8264867677	-0.1891276961	-3.1892040125
C	-0.1473572207	2.0405828427	-2.6237177107
H	2.4200396416	2.8860691834	-2.9037953176
H	3.1946219464	1.2969733974	-3.1669384681
H	2.0481881669	1.9492818650	-4.3735083743
H	0.0432123741	-0.6971863836	-2.6039113265
H	0.4739139887	-0.0806445660	-4.2256358649
H	1.7325707492	-0.8112211415	-3.1938408642
H	-0.4355020664	2.2566273131	-3.6630486521
H	-0.9839445055	1.5178543258	-2.1326626521
H	0.0213167049	2.9918106692	-2.0969319023
O	1.4082540370	1.0373242384	-1.1811274645
K	-0.5863567156	0.5286820188	0.5059880179
C	3.6025995802	2.9494840295	0.0642718026
H	2.8864633504	3.0704665670	-0.7641234592
H	3.8198724012	3.9743772014	0.4416009570
H	4.5387479138	2.5715177541	-0.3796649507
C	4.0837986439	1.2672772282	1.9594304675
H	5.0711012758	1.2396743495	1.4732824880
H	4.2365741269	1.7302122307	2.9592586006
H	3.7866802056	0.2218166633	2.1583164728
O	-1.7954997479	-1.3394498688	1.9180897114
C	-3.0741795776	-1.9489562508	1.8516637534
C	-1.0416498149	-2.0653067064	2.8731660586
C	-2.8425065364	-3.4543270152	2.0990226809
H	-3.7295104704	-1.5208781396	2.6309843430
H	-3.5021398723	-1.7139717247	0.8665946930
C	-1.3768741940	-3.5264507837	2.5793401443
H	0.0190108204	-1.8041641551	2.7475125705
H	-1.3560418270	-1.7850153498	3.8956505718
H	-2.9946628834	-4.0433517325	1.1841790676
H	-3.5391522992	-3.8338475160	2.8576672044
H	-0.7234924693	-3.9056739127	1.7798384491
H	-1.2438871634	-4.1696569715	3.4584813961
O	-2.7595493066	1.9422909091	-0.2213691117
C	-3.5011275611	2.7736372358	-1.0974357920
C	-3.6536286337	1.5692273195	0.8115051853
C	-4.9354124749	2.1999025992	-1.1126452092
H	-3.4983020378	3.8092397637	-0.7142943296
H	-3.0074260337	2.7674448791	-2.0790325874
C	-4.9425800933	1.2138312037	0.0751670084
H	-3.2129848351	0.7394822304	1.3830398906
H	-3.8127886062	2.4214637127	1.4986732991
H	-5.1598001174	1.6900400007	-2.0596578244
H	-5.6750024567	3.0009691604	-0.9844319355
H	-4.8855901780	0.1744702497	-0.2807304402
H	-5.8309471358	1.3104554765	0.7123715847
O	-2.0480252513	-0.9944545194	-1.2160021706
C	-1.9398783151	-2.4061568473	-1.2825108092
C	-2.8269929256	-0.6120377546	-2.3371182884
C	-3.3263163258	-2.8987140862	-1.7171874700
H	-1.1762318452	-2.6856125094	-2.0324308322
H	-1.6151585273	-2.7691136662	-0.2980460924
C	-3.9232448489	-1.6806685283	-2.4587222087
H	-3.2014002655	0.4024963265	-2.1571854607
H	-2.1918880678	-0.6007076547	-3.2424004972
H	-3.9363939390	-3.1623163719	-0.8419319088
H	-3.2531697402	-3.7897085329	-2.3537141902
H	-4.8528604332	-1.3457164771	-1.9786532467
H	-4.1516415473	-1.8971907880	-3.5100947755

TS1_2_4sol

M06-2X/def2SVP Electronic E: -2521.410786 a.u.

M06-2X/def2SVP Gibbs free E: -2520.544865 a.u.

M06-2X/def2TZVPP Electronic E: -2523.682945 a.u.

C	-5.6859649964	-2.5560439103	1.3330363662
C	-5.9951982962	-1.1932796128	1.1592759461
C	-5.0506034707	-0.1979603184	1.3378108861
C	-3.6779784078	-0.4797912423	1.6847456445
C	-3.4069754436	-1.8840236275	1.8844135134
C	-4.3742006596	-2.8669230139	1.7097914134
H	-6.4401416578	-3.3320150295	1.1954936844
H	-7.0122469406	-0.9047468139	0.8752588542
H	-5.3509039719	0.8394604287	1.1698318755
H	-2.4091800769	-2.1905798505	2.2035375735
H	-4.0951103072	-3.9122005373	1.8801741710
C	-2.6759452116	0.5034172934	1.8085729499
C	-1.3990290449	2.7781678708	-1.4203544809
O	-2.9627115561	1.0456759648	-1.6772284361
O	-1.1419190760	1.7408260684	-0.4649912154
C	-2.8673461758	2.4596229249	-1.8895477077
C	-3.9299544969	3.1391706760	-1.0353688958
H	-3.9630561897	4.2227461352	-1.2182570394
H	-4.9100964378	2.7112807648	-1.2904579772
H	-3.7433329289	2.9616666102	0.0301451616
C	-3.1258397207	2.7490628602	-3.3605169880
H	-4.1706809152	2.5063484189	-3.6020346358
H	-2.9628490431	3.8148013294	-3.5802022105
H	-2.4766253645	2.1500292879	-4.0121244749
C	-1.2314005062	4.1350530043	-0.7595352454
H	-0.1832674742	4.2689633131	-0.4530199013
H	-1.4877333777	4.9415567644	-1.4627498903
H	-1.8651156697	4.2291362032	0.1313603514
C	-0.3740907230	2.6125603938	-2.5440115792
H	-0.4795342988	3.3875600003	-3.3161305920
H	0.6385848678	2.6942006062	-2.1187555251
H	-0.4697473517	1.6260021350	-3.0234143432
B	-1.9709758039	0.6560380291	-0.7944598928
C	-1.8044059908	-1.6952148640	-1.6135342982
C	-3.3112849022	-1.9085617505	-1.7291245430
C	-1.1875369408	-1.3730873205	-2.9743376162
C	-1.1407556546	-2.9253102378	-1.0034507452
H	-3.7536195208	-2.0069791124	-0.7275762278
H	-3.7927239554	-1.0695099592	-2.2461620942
H	-3.5078606779	-2.8332833271	-2.2928371216
H	-0.1016277267	-1.2194757055	-2.8662772437
H	-1.3586796258	-2.1955134038	-3.6849775067
H	-1.6424715042	-0.4600799693	-3.3872356521
H	-1.1919179461	-3.7823366185	-1.6911800531

H	-0.0853091565	-2.7045632831	-0.7791315635
H	-1.6481475249	-3.1958088245	-0.0654433192
O	-1.4857017517	-0.6241553332	-0.7140495887
K	1.0195301144	0.1853328804	-0.0097674267
C	-2.9945751460	1.9249655904	2.1903022810
H	-4.0381005561	2.2097925979	1.9933545278
H	-2.8264586945	2.1087742490	3.2756293558
H	-2.3467598089	2.6456990616	1.6565189406
C	-1.3032375608	0.0741813733	2.2571900118
H	-0.5855436610	0.9058286279	2.1481443609
H	-1.2589728642	-0.2413080817	3.3272785107
H	-0.9231327472	-0.7794684836	1.6707275322
O	3.5755853025	0.9511287171	-0.1638260337
C	4.8120877827	0.3124619465	-0.4264774227
C	3.8718776833	2.3261939947	0.0132354235
C	5.6124392814	1.2927110774	-1.3019527612
H	5.3366872212	0.1163111429	0.5265834919
H	4.6032171430	-0.6493243358	-0.9140619623
C	4.8967637766	2.6438896598	-1.0788764147
H	2.9384076231	2.8973736149	-0.0615791876
H	4.2963253558	2.4901018347	1.0216130757
H	5.5764533982	0.9965452636	-2.3595125882
H	6.6671675722	1.3210059522	-0.9995906257
H	4.3829416364	2.9670858266	-1.9949079419
H	5.5812403365	3.4465150883	-0.7759675707
O	1.5765083288	-2.0729100895	1.2350669098
C	0.8868465202	-3.0583370811	1.9865511722
C	2.9541479267	-2.4216299541	1.2522749452
C	1.5340965241	-4.3664728317	1.5474116078
H	1.0343477907	-2.8803707287	3.0686715815
H	-0.1841530325	-2.9828598968	1.7581927999
C	3.0084005596	-3.9621762046	1.3514505216
H	3.4098298035	-2.0179973796	0.3373488328
H	3.4452537382	-1.9523652474	2.1232751237
H	1.0868439472	-4.6914488639	0.5956671810
H	1.4014780319	-5.1720184992	2.2807395430
H	3.4400216843	-4.4168556733	0.4492455787
H	3.6246215484	-4.2708042654	2.2061556472
O	1.6670052243	1.8490532724	1.9767396315
C	2.2507381603	0.9605190180	2.9272345217
C	1.2506078004	3.0578576958	2.6061502729
C	1.8932948321	1.5349836787	4.2932448645
H	1.8513504200	-0.0539805540	2.7632377501
H	3.3447979914	0.9319978936	2.7727761841
C	1.8801951598	3.0339634163	3.9950472355
H	1.5792233263	3.9115543327	1.9946035983
H	0.1480051751	3.0759281937	2.6666143275
H	2.6067638890	1.2438318829	5.0749031322
H	0.8882613846	1.1994219626	4.5913812483
H	2.9097087923	3.4222192255	3.9541220127
H	1.3128598066	3.6251673601	4.7252557358
O	2.1911434559	-1.3240809169	-1.8796455692
C	2.8360855689	-0.6762989310	-2.9692311878
C	2.3219844836	-2.7187409708	-2.0961611096
C	3.9919650675	-1.6053693586	-3.3997707234
H	2.1155793191	-0.5369529769	-3.7944186971
H	3.1657577493	0.3129929147	-2.6208904435
C	3.7631416136	-2.8824399699	-2.5668070915
H	2.0847710200	-3.2376392345	-1.1578023119
H	1.6069981697	-3.0507910430	-2.8732382599
H	4.9739326443	-1.1574516637	-3.1953322686
H	3.9388332977	-1.8132588336	-4.4763734072
H	4.4331111064	-2.9048608228	-1.6933345136
H	3.9183895872	-3.8052888715	-3.1398807397

TS2_3_3sol
M06-2X/def2SVP Electronic E: -2598.534094 a.u.
M06-2X/def2SVP Gibbs free E: -2597.653709 a.u.
M06-2X/def2TZVPP Electronic E: -2600.882171 a.u.

C	-2.3001116323	-1.0827599546	1.6727081617
C	-2.6985000345	-1.4266151915	0.3716531856
C	-2.7247604722	-0.4862673982	-0.6521348169
C	-2.3583143898	0.8840805047	-0.4270330131
C	-1.8923489710	1.1900205395	0.8973001988
C	-1.8890129592	0.2409278977	1.9097114511
H	-2.2931487074	-1.8248660006	2.4725853594
H	-3.0015716938	-2.4548235900	0.1555391676
H	-3.0410320494	-0.8000229837	-1.6482838989
H	-1.5520613513	2.2019819903	1.1233898424
H	-1.5531288993	0.5333756338	2.9083193244
C	-2.5329463916	1.8871047130	-1.4130425764
C	2.6018608035	2.7434643650	1.4121142563
O	2.7841079144	3.0028795576	-0.9197882173
O	1.7674185124	1.7807290054	0.7343553273
C	2.8727178718	3.8020702739	0.2818852491
C	1.7768485278	4.8587620761	0.1956209247
H	1.8189752822	5.5511126232	1.0475253117
H	1.9125622143	5.4354769056	-0.7302346040
H	0.7796120484	4.3946354712	0.1685886617
C	4.2401280890	4.4557539074	0.3463182163
H	4.3372300123	5.1854953131	-0.4700369437
H	4.3638776516	4.9898706322	1.2997858022
H	5.0453304509	3.7172583482	0.2458035405
C	1.8610142652	3.2979045700	2.6161099066
H	1.7514314379	2.5145976328	3.3804722754
H	2.4267110353	4.1293400643	3.0617786559
H	0.8604284523	3.6562962927	2.3438931298
C	3.8668647603	2.0189718535	1.8586662986
H	4.5201300587	2.6809888600	2.4439289997
H	3.5796957733	1.1666536380	2.4931139069
H	4.4344631141	1.6361746418	0.9978661848
B	1.9950592291	1.9259753587	-0.6180710495
C	1.5552621371	0.9841291549	-2.9023547851
C	0.9084992977	2.2300523714	-3.4945701362
C	3.0245591868	0.8704951720	-3.2888733669
C	0.7931732571	-0.2740335568	-3.3019105181
H	-0.1491098773	2.2809811048	-3.1971424419
H	1.4220074090	3.1369294238	-3.1427047127
H	0.9619520445	2.2006402089	-4.5923999743
H	3.4570573310	-0.0300696349	-2.8288771468
H	3.1244466806	0.7865738836	-4.3804840274
H	3.5876368243	1.7529107851	-2.9526112518
H	0.7965182505	-0.4013958189	-4.3938297894
H	1.2529791543	-1.1582342554	-2.8336557032
H	-0.2524824334	-0.2007447288	-2.9630296718
O	1.4449648954	1.0330484209	-1.4693122944
K	0.3615480541	-0.7865743535	0.0858099098
C	-2.8753640489	1.4664826279	-2.8165727821
H	-2.1289152138	0.7980820920	-3.2883622322
H	-3.8386549394	0.9211972996	-2.8452219742
H	-2.9828721767	2.3444121513	-3.4735612603
C	-1.8600269477	3.2191473777	-1.2023452395
H	-2.0040530886	3.8768875943	-2.0737761334
H	-2.2613966656	3.7623325781	-0.3242323751
H	-0.7672368239	3.1326361376	-1.0368848014
O	1.5019445344	-1.6126013236	2.3274809808
C	2.5348288333	-2.5100106682	2.7006188092
C	1.3133869972	-0.6381150852	3.3556572569
C	3.4758147307	-1.6759381077	3.5578347462
H	2.1192006661	-3.3572634286	3.2769847144
H	2.9926839505	-2.9034252703	1.7819170280
C	2.4864304813	-0.8031172138	4.3365923470
H	1.2924465052	0.3528820479	2.8740978646
H	0.3438738265	-0.8034547476	3.8514578777
H	4.1195791645	-1.0577971031	2.9123213331
H	4.1183170604	-2.2867822047	4.2052030068
H	2.9123657142	0.1617275409	4.6435189672
H	2.1532478848	-1.3278036233	5.2430609127

O	0.0395745771	-3.0939639096	-1.1599822298
C	-0.7928506460	-3.7610808867	-2.0895869521
C	0.4285291873	-4.0583529392	-0.1943267915
C	-0.0896156258	-5.0965354712	-2.3265431293
H	-1.7992032837	-3.9145178611	-1.6570281227
H	-0.8863148090	-3.1328027812	-2.9852703448
C	0.5536262554	-5.3952663256	-0.9553159761
H	1.3678280690	-3.7135336282	0.2623298528
H	-0.3419540235	-4.1251175851	0.5954591568
H	0.6817369448	-4.9772864949	-3.1007259251
H	-0.7830937533	-5.8807665299	-2.6558908460
H	1.6023311823	-5.7036108731	-1.0626263682
H	0.0252887932	-6.1958139390	-0.4215019975
O	2.7198697987	-1.7150233486	-0.7517692130
C	3.7759180629	-1.0294079548	-0.0973193541
C	3.2996827448	-2.7260925466	-1.5536331747
C	4.9259404471	-2.0507110736	0.0600664361
H	4.0976081063	-0.1664276481	-0.7084956266
H	3.3807432434	-0.6465524401	0.8546288174
C	4.4062814692	-3.3011199169	-0.6761669462
H	2.5079058410	-3.4345540979	-1.8317892738
H	3.7212375383	-2.2866471106	-2.4782990578
H	5.1506069701	-2.2591489898	1.1151592303
H	5.8444193926	-1.6703516088	-0.4062334740
H	3.9757106066	-4.0248836835	0.0327842469
H	5.1850805210	-3.8138487043	-1.2550142221
C	-6.0371796470	-0.2992952247	2.3312321540
C	-7.1758173032	-1.0678106975	2.0681595487
C	-7.7845870219	-0.9496099934	0.8148291374
C	-7.2720977450	-0.0827231563	-0.1445038908
C	-6.1215733719	0.7068285579	0.0994227110
C	-5.5175638654	0.5648599018	1.3729906010
H	-5.5351807206	-0.3822405570	3.2986615180
H	-7.5762425091	-1.7484937493	2.8212126640
H	-8.6738721365	-1.5409999179	0.5829183454
H	-7.7667865900	-0.0004005760	-1.1161663402
H	-4.6081962381	1.1260726207	1.5997140629
C	-5.6457730596	1.6333237880	-0.9145056249
H	-6.1933702341	1.6133610428	-1.8623189575
C	-4.6087265616	2.5266818023	-0.7733221109
H	-4.5062749336	3.3294486040	-1.5089456916
H	-4.1928852810	2.7493635039	0.2154338031

TS2_3_4sol
M06-2X/def2SVP Electronic E: -2830.709975 a.u.
M06-2X/def2SVP Gibbs free E: -2829.718904 a.u.
M06-2X/def2TZVPP Electronic E: -2833.325102 a.u.

C	-1.1355021975	-3.5564914147	-1.7166041001
C	-2.1983761088	-2.9478076638	-2.4061640843
C	-2.8405066000	-1.8217647152	-1.9150300253
C	-2.4726837251	-1.2107458176	-0.6655334553
C	-1.3851365039	-1.8603939978	0.0121963165
C	-0.7477695116	-2.9872361684	-0.4970133987
H	-0.6421555045	-4.4463168908	-2.1102922133
H	-2.5306378671	-3.3668361927	-3.3604470778
H	-3.6563481437	-1.3932500980	-2.5013692967
H	-1.0380283306	-1.4592073722	0.9647364361
H	0.0776737954	-3.4225681524	0.0785118310
C	-3.1715615603	-0.1318934190	-0.0730090282
C	0.5419126641	3.5696192284	1.1950898104
O	-0.0038335050	3.5904528832	-1.0945334368
O	0.5931634841	2.2322343777	0.6547732991
C	-0.2954719171	4.3379454450	0.1077337853
C	-1.7983865669	4.2334898250	0.3420923932
H	-2.1126701107	4.8321538012	1.2081402357
H	-2.3227891997	4.6065958928	-0.5491283664
H	-2.1016135613	3.1891917048	0.5073156456
C	0.1129054839	5.7850144667	-0.0946004150
H	-0.5300883050	6.2435415778	-0.8592037354
H	-0.0096635385	6.3512289982	0.8403244506
H	1.1554854755	5.8684368528	-0.4260612964
C	-0.1084586429	3.5297738592	2.5667192145
H	0.5515789637	3.0078112447	3.2754205790
H	-0.2713181794	4.5507525438	2.9423737166
H	-1.0714885754	3.0038475869	2.5396067141
C	1.9758828862	4.0759225650	1.3036241603
H	2.0127054570	5.0712139365	1.7680504807
H	2.5494571248	3.3789338036	1.9329665302
H	2.4578220680	4.1312983618	0.3163251175
B	0.3453620623	2.3277682285	-0.6988543726
C	0.1964336616	1.0886801652	-2.8698787451
C	-1.2677104867	1.4241795589	-3.1229285255
C	1.1309214493	2.0037998407	-3.6512400595
C	0.4838370884	-0.3754748071	-3.1819000841
H	-1.9070587402	0.7755644894	-2.5070064056
H	-1.4746398076	2.4747876672	-2.8722944303
H	-1.5166742908	1.2606211940	-4.1814351267
H	2.1760848111	1.7377384883	-3.4352975415
H	0.9574524368	1.8888535447	-4.7308655110
H	0.9633330364	3.0564450152	-3.3808689776
H	0.2802960153	-0.5927539036	-4.2401917440
H	1.5429805963	-0.6000086239	-2.9751431627
H	-0.1556626122	-1.0281401409	-2.5658577195
O	0.4738139282	1.2221068754	-1.4628799206
K	1.5115057291	-0.6585155734	0.0740473967
C	-4.0770270875	0.7800014612	-0.8486195693
H	-3.6073320363	1.7619429894	-1.0584835725
H	-4.3970372455	0.3663984080	-1.8161259575
H	-4.9976602991	0.9943014217	-0.2684089871
C	-2.6238876984	0.4832154580	1.1898225882
H	-3.3417118948	1.2156220238	1.5975566099
H	-2.4469618365	-0.2598284247	1.9874944349
H	-1.6602007197	1.0142437104	1.0429191901
O	3.3528809800	-0.0586784034	1.8944229563
C	4.7404219198	0.2371061346	1.9302508227
C	2.6886995902	0.5593105289	3.0001189113
C	4.8347621767	1.5731273704	2.6526288053
H	5.2860524158	-0.5511141227	2.4817565458
H	5.1132234267	0.2599942375	0.8961691372
C	3.7418887314	1.4187066864	3.7131324541
H	1.8536390462	1.1578779539	2.5996277009
H	2.2690842299	-0.2173754955	3.6584736037
H	4.5892968694	2.3911360851	1.9567094455
H	5.8301296907	1.7625337714	3.0750216379
H	3.3331994097	2.3778781251	4.0590931818
H	4.1406436413	0.8854792277	4.5881871818
O	2.8372916037	-2.8389410006	-0.6133110764
C	2.6532522293	-3.8235055054	-1.6221940996
C	4.2286164229	-2.6695093622	-0.3398963741
C	3.9157141718	-3.7458495803	-2.4671058036
H	2.5434478787	-4.8232170801	-1.1623884065
H	1.7302807897	-3.5861295629	-2.1687827305
C	4.9830762663	-3.4901583270	-1.3981889121
H	4.4515095859	-1.5922236614	-0.3959822466
H	4.4512082606	-3.0124665279	0.6838230490
H	3.8438055397	-2.8983257120	-3.1656911509
H	4.0957597189	-4.6589257842	-3.0491232943
H	5.8653939764	-2.9615424715	-1.7838685000
H	5.3213684332	-4.4448960333	-0.9706596517
O	0.9659148995	-1.9822170737	2.3322542084
C	2.0038175579	-2.9147315751	2.6361777741
C	-0.0116421012	-1.9645069432	3.3724915266
C	1.4579159463	-3.7700412009	3.7726287969
H	2.2431121559	-3.4793385231	1.7234826235
H	2.9064429672	-2.3580668599	2.9470706331
C	0.5999234456	-2.7536415570	4.5256754858
H	-0.2439669938	-0.9193124277	3.6286857485
H	-0.9362348973	-2.4464151830	3.0101931032

H	2.2520133051	-4.2183654592	4.3837411768
H	0.8256326735	-4.5771514701	3.3722145799
H	1.2374035853	-2.1017666638	5.1431870161
H	-0.1612917363	-3.2080144879	5.1727524539
O	3.4874473719	0.4629232978	-1.4183575840
C	3.6484253812	1.7802236717	-0.9088998326
C	4.4871185292	0.2592905563	-2.4010510668
C	5.1415284861	2.1387710125	-1.0848635259
H	3.0095196453	2.4798863003	-1.4769317860
H	3.3080936441	1.7754844598	0.1371863755
C	5.7292505667	0.9125734682	-1.8075880998
H	4.5824258558	-0.8202204702	-2.5801369798
H	4.1978307085	0.7492703165	-3.3500646630
H	5.6386052970	2.3265165095	-0.1235005633
H	5.2477930113	3.0444386518	-1.6966434379
H	6.2008694300	0.2213340615	-1.0921124687
H	6.4745500941	1.1770512499	-2.5684606589
C	-8.2680711747	-1.0914928111	-1.7591381289
C	-9.4400630834	-0.5169700711	-1.2567788006
C	-9.4459887923	-0.0528896259	0.0614718148
C	-8.3103352462	-0.1658076362	0.8577704541
C	-7.1154896737	-0.7464106639	0.3727713540
C	-7.1286640441	-1.2006560590	-0.9684644144
H	-8.2394632008	-1.4539279133	-2.7895555037
H	-10.3293222706	-0.4284161054	-1.8829351619
H	-10.3492191574	0.4018012396	0.4753860417
H	-8.3352438216	0.1979881036	1.8885129069
H	-6.2226980506	-1.6347255859	-1.3975354611
C	-5.9547136263	-0.8727461939	1.2417271632
H	-6.0396790554	-0.3922631966	2.2220847883
C	-4.7903797294	-1.5127356760	0.9092336155
H	-4.0412763261	-1.7244031467	1.6777342005
H	-4.7461648880	-2.1626729286	0.0303375153

TS3_4_3sol
M06-2X/def2SVP Electronic E: -2598.570017 a.u.
M06-2X/def2SVP Gibbs free E: -2597.682976 a.u.
M06-2X/def2TZVPP Electronic E: -2600.912936 a.u.

C	-4.7734166893	-4.0996495378	0.9755740509
C	-4.6842702971	-3.0884654490	1.9322389051
C	-4.8097928901	-1.7507949234	1.5586291007
C	-5.0115396379	-1.3804701341	0.2196564908
C	-5.1195606468	-2.4134480909	-0.7245112394
C	-5.0024507504	-3.7541449403	-0.3554887956
H	-4.6715552176	-5.1469240098	1.2663367258
H	-4.5073839946	-3.3402597454	2.9799541244
H	-4.7175217810	-0.9820330092	2.3257726097
H	-5.2844741229	-2.1761540047	-1.7763458677
H	-5.0865800021	-4.5324678952	-1.1169984423
C	-5.0108357205	0.0859755276	-0.2043672464
C	-0.0967509099	3.3682591940	0.8325000279
O	-1.3919868821	2.8842598323	-1.1116747359
O	0.0999081123	2.0146235164	0.4032413393
C	-1.2339986664	3.9234249617	-0.1415175020
C	-2.5847955726	4.1460026962	0.5349595798
H	-2.5304761903	4.9485189566	1.2836767428
H	-3.3148480850	4.4414715537	-0.2327046147
H	-2.9524876290	3.2333706123	1.0188544115
C	-0.8439688487	5.1975258147	-0.8828909850
H	-1.6537307378	5.4670407194	-1.5759520812
H	-0.6993394418	6.0328238192	-0.1828609379
H	0.0748227715	5.0649786177	-1.4678015284
C	-0.4964559619	3.3743014853	2.3029746172
H	0.3165006159	2.9437363324	2.9063021135
H	-0.6717558888	4.4016461527	2.6538194744
H	-1.4031597111	2.7826436535	2.4779033858
C	1.2430467731	4.0833357191	0.6737156335
H	1.1725412247	5.1426566999	0.9570860069
H	1.9836845317	3.6033713234	1.3316747234
H	1.6097027620	4.0163855924	-0.3597242078
B	-0.7003897452	1.7625940663	-0.7085948850
C	-0.2872923438	0.7331007373	-2.9406158809
C	-1.6488185572	0.9220972014	-3.6023332984
C	0.6688480399	1.8633408194	-3.3206304816
C	0.3160996632	-0.6153016418	-3.3210138203
H	-2.3084592319	0.0719354132	-3.3752730591
H	-2.1243844828	1.8447087604	-3.2409783179
H	-1.5328149946	0.9874041124	-4.6944540399
H	1.6204119629	1.7305957357	-2.7836475940
H	0.8684727991	1.8498930146	-4.4022999863
H	0.2367563892	2.8404376072	-3.0603685942
H	0.4151592699	-0.7051668162	-4.4126044056
H	1.3134338663	-0.7261348612	-2.8668854210
H	-0.3287591163	-1.4300439967	-2.9575833112
O	-0.4302841494	0.6853440757	-1.5154565149
K	1.1822255267	-0.6165220838	0.1336317388
C	-5.6084582125	0.9852839672	0.8829999491
H	-6.6328716704	0.6744245327	1.1438586151
H	-5.0028575236	0.9830480111	1.7994205611
H	-5.6494808402	2.0249473511	0.5206985288
C	-5.8159390606	0.2997603695	-1.4919884325
H	-5.8727944951	1.3758890601	-1.7163314571
H	-5.3439971100	-0.1841917215	-2.3599344266
H	-6.8444275322	-0.0843366438	-1.4008882930
O	3.0076605641	-0.2327137668	2.0569913851
C	4.1229415730	-0.6531505119	2.8276401403
C	2.4746815806	0.9075869718	2.7121215675
C	4.7374265768	0.6299939472	3.4302577943
H	3.7827944521	-1.3389823661	3.6229517818
H	4.8099741261	-1.2017595462	2.1684556885
C	3.7076802372	1.7248664491	3.0844830197
H	1.7743273674	1.4076545815	2.0270826275
H	1.9176945145	0.6001037917	3.6175702771
H	5.7235053102	0.8515311689	3.0011599923
H	4.8629008596	0.5234379805	4.5157331031
H	4.0408256155	2.3109201460	2.2144058424
H	3.5181796241	2.4185210519	3.9138240832
O	2.5152857570	-2.6459336641	-0.9433146728
C	2.3397597509	-3.9700567409	-1.4111027145
C	3.6653145877	-2.6774559760	-0.1156256554
C	3.7526918989	-4.4464341179	-1.7674958864
H	1.9061112099	-4.5945234677	-0.6082928547
H	1.6421779125	-3.9499161168	-2.2583178845
C	4.6570327222	-3.6064545171	-0.8350923132
H	4.0192067741	-1.6443999975	0.0148148846
H	3.3940172931	-3.0816044013	0.8779975073
H	3.9733134872	-4.2299043887	-2.8215435211
H	3.8675623458	-5.5269897790	-1.6138332515
H	5.3864231224	-3.0246998478	-1.4155766451
H	5.2145942435	-4.2235191770	-0.1188384745
O	3.1217923124	0.4085941524	-1.3622471160
C	3.6699774848	1.5424238718	-0.7159585980
C	4.1278602642	-0.0992599607	-2.2247958885
C	5.0852096874	1.1083084350	-0.3478201163
H	3.6851103808	2.4044083885	-1.4108259204
H	3.0304914298	1.7953890901	0.1413511510
C	5.4747577046	0.1929353800	-1.5276153683
H	3.9230546470	-1.1673594413	-2.3793403913
H	4.0697974809	0.4163810730	-3.1999947088
H	5.0469281341	0.5391210294	0.5919645774
H	5.7690067836	1.9566457556	-0.2130372955
H	5.9600571793	-0.7286703510	-1.1783151758
H	6.1679898621	0.6923789469	-2.2170637904
C	-0.8627289371	-2.8824540573	0.6112204068
C	-0.1206198457	-2.8601716134	1.8065521360
C	-0.2369719603	-1.7117328262	2.6124615322
C	-1.0254198735	-0.6353928003	2.2381900082
C	-1.7926520751	-0.6175852716	1.0127661268

C	-1.6621003784	-1.8184264276	0.2198758883
H	-0.8146297230	-3.7642644947	-0.0357687254
H	0.4935416594	-3.7085743257	2.1112546346
H	0.3146596192	-1.6587840275	3.5565026137
H	-1.0846997470	0.2393199584	2.8931766049
H	-2.2268523144	-1.9006873671	-0.7104341257
C	-2.5497272817	0.4979768679	0.6388494963
H	-2.6148052238	1.2944851565	1.3854325164
C	-3.5325902170	0.5297674330	-0.4884940178
H	-3.6000477973	1.5582269117	-0.8925552824
H	-3.1642407279	-0.0898493025	-1.3240900456

TS3_4'_3sol
M06-2X/def2SVP Electronic E: -2907.856292 a.u.
M06-2X/def2SVP Gibbs free E: -2906.847084 a.u.
M06-2X/def2TZVPP Electronic E: -2910.542934 a.u.

C	-4.9227371376	4.9192741317	-0.3778865723
C	-3.6867718145	4.8239249141	0.2571490501
C	-3.3120660906	3.6397993560	0.8979331113
C	-4.1599714226	2.5242133709	0.9218884317
C	-5.4016801144	2.6389213015	0.2759279375
C	-5.7798637964	3.8169861514	-0.3652718955
H	-5.2173580362	5.8425695239	-0.8800025201
H	-3.0025330863	5.6752027319	0.2552204365
H	-2.3345888586	3.5930457824	1.3801430448
H	-6.0826158072	1.7857116236	0.2613113511
H	-6.7509430074	3.8732301235	-0.8617056716
C	-3.7613455998	1.1997744326	1.5765301459
C	-4.8614185643	0.7533829753	2.5515009927
H	-5.0061753095	1.4951700094	3.3525835685
H	-5.8246910742	0.6037695260	2.0433439440
H	-4.5814254177	-0.2045835636	3.0178516433
C	-2.4572174114	1.3283550671	2.3631648909
H	-2.2725445204	0.3951586845	2.9168762610
H	-1.5947030916	1.4978671795	1.7016129510
H	-2.5098222611	2.1454247204	3.1003257880
C	-0.5999730682	-2.5908727920	-1.5037596115
C	0.2906979045	-2.0702033017	-2.4531179631
C	0.2113042747	-0.6987264240	-2.7426140526
C	-0.7126550200	0.1185557232	-2.1038119178
C	-1.6478027267	-0.3853974974	-1.1421424427
C	-1.5386995897	-1.7843785399	-0.8659095339
H	-0.5650353268	-3.6560602862	-1.2589705338
H	1.0112896522	-2.7119583626	-2.9613492028
H	0.8920592059	-0.2596785334	-3.4763192610
H	-0.7487060845	1.1830640496	-2.3537708855
H	-2.2258051287	-2.2431197502	-0.1504593311
C	-2.7038113912	0.4501612793	-0.6698920181
H	-2.5262524285	1.5202776680	-0.8187758641
C	-3.6154700052	0.1117813982	0.4731085897
H	-4.6403129590	-0.0719940472	0.0847121453
H	-3.3087878076	-0.8243259473	0.9746303645
C	-8.0276162092	-2.3650992928	-1.4673307999
C	-7.3849213421	-1.1694182985	-1.7647509682
C	-5.9739498825	-1.0896828696	-1.8977389990
C	-5.2541631046	-2.2985067697	-1.7003293845
C	-5.9060541758	-3.4933380011	-1.4110924834
C	-7.2981998010	-3.5465165720	-1.2888006347
H	-9.1165841099	-2.3791600118	-1.3745346963
H	-7.9752940597	-0.2596625961	-1.9062080109
H	-4.1629301454	-2.2844607489	-1.7574779202
H	-5.3141100993	-4.4010195478	-1.2670699823
H	-7.8031859114	-4.4847928836	-1.0538970184
C	-5.3259885432	0.1622644762	-2.2089219035
H	-5.9558557951	1.0563287452	-2.1861430225
C	-3.9689965505	0.2931907324	-2.4580280821
H	-3.6026837114	1.2416285284	-2.8615003972
H	-3.4031193634	-0.5782013231	-2.8017530320
C	4.0223276711	1.9536494870	-1.9029034463
O	5.3820831950	0.0334892206	-1.8544558379
O	3.2671027301	0.8162874913	-1.4317712427
C	5.2532966268	1.2669947202	-2.5985640087
C	4.9675252522	0.8825104872	-4.0464082590
H	4.9106527438	1.7685523266	-4.6932633199
H	5.7807265994	0.2379076477	-4.4087459870
H	4.0224135408	0.3248185163	-4.1305483704
C	6.5499844165	2.0473839152	-2.4991376510
H	7.3485209713	1.5006881360	-3.0204527349
H	6.4408718864	3.0323397722	-2.9762357056
H	6.8551523322	2.1902625248	-1.4550248058
C	3.1605523802	2.7794868793	-2.8412044132
H	2.3476150199	3.2590651224	-2.2769380136
H	3.7613298483	3.5724027917	-3.3105348568
H	2.7139885182	2.1608281071	-3.6294601030
C	4.4184855249	2.7792467938	-0.6843399485
H	4.9439908179	3.6977927568	-0.9804230331
H	3.5060190954	3.0628310591	-0.1383723856
H	5.0671380321	2.2062220934	-0.0056648444
B	4.1425762875	-0.2456224394	-1.3469888193
C	4.4281080568	-2.6404176462	-0.6674627583
C	4.8338630124	-3.1445049674	-2.0475486700
C	5.6416817596	-2.4105926257	0.2249083300
C	3.4412234749	-3.5885129741	0.0038935352
H	3.9462534140	-3.2493213530	-2.6889810790
H	5.5346355418	-2.4447032847	-2.5245207430
H	5.3214733023	-4.1263055126	-1.9651014317
H	5.3132975255	-2.0512285150	1.2112361738
H	6.1953166833	-3.3503819317	0.3625031216
H	6.3191137268	-1.6682470232	-0.2212741431
H	3.9074305335	-4.5663486980	0.1903856709
H	3.1111650967	-3.1593128867	0.9628406869
H	2.5578038157	-3.7395394125	-0.6362306665
O	3.7049385725	-1.4057501695	-0.8103131428
K	1.3242156560	-0.7532178888	0.1203875123
O	0.7647239655	1.5170829736	1.3079696919
C	1.0309228936	2.0673864450	2.5897305745
C	0.5858047419	2.5626262757	0.3489833470
C	1.6944745963	3.4064439074	2.3032948433
H	0.0871746569	2.2073745089	3.1481509842
H	1.6592900835	1.3542481559	3.1399101949
C	0.9030140285	3.8754280698	1.0783646723
H	1.2730938089	2.3684252947	-0.4907658873
H	-0.4477645317	2.5357646532	-0.0352594738
H	2.7554504828	3.2539140416	2.0489970100
H	1.6368296982	4.1017662953	3.1507949050
H	1.4567888429	4.5812138341	0.4448401303
H	-0.0272402775	4.3676098913	1.3994017857
O	0.4735580674	-2.5137543420	1.9088909665
C	0.1149979677	-3.8749958154	2.1183693841
C	-0.4925571828	-1.6452083340	2.5027007151
C	-0.8436851984	-3.8545266732	3.3008159113
H	-0.3807024443	-4.2762767053	1.2163561565
H	1.0311254444	-4.4554449357	2.2994404978
C	-1.5994723163	-2.5499130035	3.0439874508
H	-0.0036641574	-1.0668810377	3.3071007995
H	-0.8655004896	-0.9342386127	1.7463137090
H	-0.2798799179	-3.7924551716	4.2445141016
H	-1.4929388343	-4.7386806772	3.3380152731
H	-2.0822369940	-2.1354032394	3.9385711719
H	-2.3725878613	-2.7072263294	2.2757374632
O	3.0284147313	-0.7137624449	2.1367233776
C	3.9168614304	0.3878805919	2.0468867148
C	3.3102530271	-1.4060663905	3.3378198518
C	4.2704198861	0.7828599978	3.4986533839
H	4.8240398462	0.0963345277	1.4870763307
H	3.4049161411	1.1792095500	1.4794772138
C	3.5809737170	-0.2996243911	4.3535414251
H	2.4466008913	-2.0450981535	3.5663073701

H	4.2036766416	-2.0472214889	3.2095471090
H	3.9107235596	1.7905615656	3.7487488748
H	5.3584708891	0.7732419472	3.6456988665
H	2.6293667771	0.0697608462	4.7629380966
H	4.2006434523	-0.6407933930	5.1927113624

TS3_4_4sol

M06-2X/def2SVP Electronic E: -2830.736523 a.u.

M06-2X/def2SVP Gibbs free E: -2829.738332 a.u.

M06-2X/def2TZVPP Electronic E: -2833.347341 a.u.

C	-6.1882262673	-3.2586778825	-0.3512342400
C	-4.9317888401	-3.7298379942	0.0208285152
C	-4.0871331101	-2.9447207980	0.8091697194
C	-4.4652746025	-1.6637701849	1.2325546396
C	-5.7408230675	-1.2105068919	0.8551445012
C	-6.5916175412	-1.9930386734	0.0777067624
H	-6.8482607936	-3.8700803102	-0.9692233208
H	-4.5992434788	-4.7180365267	-0.3038005014
H	-3.1112919308	-3.3430221569	1.0869415100
H	-6.0740617529	-0.2155436132	1.1567227752
H	-7.5740618614	-1.6076001711	-0.2029537665
C	-3.5186917068	-0.7443282550	2.0003953437
C	-0.4895539420	3.5032662470	1.1420660330
O	-1.4830011464	3.1943571770	-0.9782847915
O	-0.2311585156	2.1755583911	0.6564789763
C	-1.6165846894	4.0384504948	0.1689051481
C	-3.0301731259	3.8847178556	0.7188296565
H	-3.2137673255	4.5793034384	1.5506860489
H	-3.7436569655	4.1107342883	-0.0867133706
H	-3.2156073366	2.8615998397	1.0618439350
C	-1.4145592062	5.4822903515	-0.2745166990
H	-2.2236223146	5.7630461076	-0.9641316872
H	-1.4495285705	6.1637154710	0.5884060443
H	-0.4584547636	5.6212500063	-0.7948119983
C	-0.9165372516	3.4537167101	2.6011557126
H	-0.0866500709	3.0903122396	3.2249485576
H	-1.1852065568	4.4611639504	2.9522603805
H	-1.7777929815	2.7907040700	2.7516147769
C	0.8175291296	4.2860592106	1.0222494943
H	0.7048376883	5.3240935622	1.3646704263
H	1.5798253733	3.8008556334	1.6500053351
H	1.1811437036	4.2931323251	-0.0162556642
B	-0.6801508820	2.1112948563	-0.6735739888
C	0.2699460066	1.8515872001	-2.9654338874
C	-1.0393973375	1.9762492454	-3.7399831775
C	1.0237140552	3.1808242511	-2.9239703867
C	1.1564734566	0.7744944939	-3.5829824753
H	-1.5812724824	1.0195133798	-3.7270062486
H	-1.6789690441	2.7503246505	-3.2966829685
H	-0.8289324981	2.2475287155	-4.7856930417
H	1.9425968428	3.0579958442	-2.3296086061
H	1.2979400192	3.5003504049	-3.9404544359
H	0.3973480619	3.9635921497	-2.4724522822
H	1.4108984316	1.0299178508	-4.6219109067
H	2.0905592785	0.6832054187	-3.0065523714
H	0.6302195497	-0.1909278383	-3.5780874851
O	0.0286487415	1.4126279157	-1.6209573760
K	1.4361094492	-0.1147653037	-0.0378654164
C	-2.3470833280	-1.5080512138	2.6228473302
H	-2.6962709054	-2.2934987868	3.3129681615
H	-1.7054836900	-1.9683378157	1.8588392647
H	-1.7175588001	-0.8068725901	3.1950247108
C	-4.2672219052	-0.0209824430	3.1294209811
H	-3.5592994882	0.5870457604	3.7146525248
H	-5.0389628594	0.6615696291	2.7439887339
H	-4.7522507984	-0.7330096495	3.8159732909
O	2.5966480634	-0.0581585205	2.3298494420
C	3.5954642826	-0.6743698002	3.1247968732
C	2.0034069787	0.9455520800	3.1374695384
C	4.1827157859	0.4449800982	4.0105657927
H	3.1374554180	-1.4645481373	3.7458988880
H	4.3270618799	-1.1418494211	2.4516007165
C	3.1881469694	1.6127930263	3.8327958686
H	1.4155034137	1.6072537478	2.4850013185
H	1.3192205897	0.4819140602	3.8730676996
H	5.1933836418	0.7276938719	3.6881145555
H	4.2459702912	0.1175627974	5.0564278569
H	3.6146511953	2.3891363738	3.1802030656
H	2.9028003120	2.0839435847	4.7820020111
O	3.3601589620	-1.8686866380	-0.5580330536
C	2.9166810612	-2.9086806087	-1.4123877154
C	4.6996542672	-2.1819916372	-0.2240434266
C	4.0941631421	-3.1528549960	-2.3596574153
H	2.6901473135	-3.8114207189	-0.8135767517
H	1.9941085065	-2.5855592039	-1.9143175767
C	5.3228189809	-2.7652265613	-1.5054474852
H	5.1877240331	-1.2657304146	0.1357835938
H	4.7114913031	-2.9300281654	0.5906759375
H	4.0093218364	-2.4985255599	-3.2382507771
H	4.1272733589	-4.1911619648	-2.7131087574
H	5.9424931080	-2.0180561048	-2.0196593992
H	5.9622156573	-3.6271046689	-1.2752093833
O	3.4140808311	1.4635220254	-0.9483790333
C	3.7782137652	2.2552388545	0.1704829638
C	4.5909783961	1.0510063668	-1.6410937659
C	5.1024123352	1.6652821422	0.6295254715
H	3.8916323313	3.3156834098	-0.1244156818
H	2.9738583643	2.1851018142	0.9164295106
C	5.7786392716	1.3717096796	-0.7146916554
H	4.4909652438	-0.0241333326	-1.8523476633
H	4.6658161543	1.5874795683	-2.6010308964
H	4.9029441429	0.7354510010	1.1852765338
H	5.6823409069	2.3435189697	1.2697759564
H	6.5030622877	0.5474361516	-0.6639087850
H	6.3107161414	2.2653686250	-1.0703494906
C	-4.0613972959	-0.6342044178	-3.2470949485
C	-3.1714435142	-1.3662347900	-4.0488913948
C	-1.9185711029	-1.6765260216	-3.4965322578
C	-1.5697888911	-1.2699220158	-2.2186258530
C	-2.4561320573	-0.5228558110	-1.3655679788
C	-3.7306381922	-0.2269713088	-1.9624305272
H	-5.0502660096	-0.3758154546	-3.6389307128
H	-3.4462416057	-1.6893608796	-5.0538360424
H	-1.1937552024	-2.2486103197	-4.0853746922
H	-0.5818046135	-1.5301075871	-1.8244132128
H	-4.4682826862	0.3360111716	-1.3849101277
C	-2.0473214418	-0.1010499242	-0.0847195567
H	-1.1003322425	-0.5340991717	0.2568533241
C	-2.9578151407	0.3416898591	1.0176061549
H	-2.4170594637	1.0584582589	1.6640079558
H	-3.8296180954	0.8870858316	0.6161565214
O	0.5718305978	-2.5527462071	0.6002837553
C	-0.3406593953	-3.5390868475	0.1213754881
C	1.3233075232	-3.0548552460	1.7052770725
C	-0.4145965881	-4.5889102220	1.2229108115
H	-1.2999842444	-3.0480930947	-0.0990688703
H	0.0479315482	-3.9757574333	-0.8166760213
C	1.0111853401	-4.5475611837	1.7739288442
H	2.3882040768	-2.8316883033	1.5363987572
H	1.0009552042	-2.5356312076	2.6250163788
H	-0.7168647246	-5.5750883843	0.8472344626
H	-1.1303990331	-4.2752916561	1.9994834081
H	1.6878438348	-5.1124184821	1.1141603084
H	1.1049298388	-4.9494599821	2.7911638763

TS3_4'_4sol

M06-2X/def2SVP Electronic E: -3140.029720 a.u.

M06-2X/def2SVP Gibbs free E: -3138.911328 a.u.

M06-2X/def2TZVPP Electronic E: -3142.984800 a.u.

C	-7.0869629955	4.1738290024	-0.1410146971
C	-5.8323589584	4.4294663885	0.4069602585
C	-5.1351456392	3.4210160931	1.0779918414
C	-5.6702974842	2.1332836411	1.2174351650
C	-6.9363027593	1.8945990878	0.6566088291
C	-7.6362529543	2.8969067143	-0.0120407163
H	-7.6331814981	4.9604886809	-0.6647408571
H	-5.3863393634	5.4218961008	0.3144072150
H	-4.1546385275	3.6533841759	1.4944812705
H	-7.3824140653	0.9009620549	0.7331644965
H	-8.6174366251	2.6771334511	-0.4383316562
C	-4.9260392393	1.0000224804	1.9299769249
C	-5.8507352651	0.3631145488	2.9794580223
H	-6.1954796646	1.1100404457	3.7117040307
H	-6.7353495519	-0.0993261141	2.5186761399
H	-5.3111769780	-0.4273194299	3.5246075940
C	-3.6774719235	1.5090144853	2.6536808611
H	-3.2175597180	0.6792918538	3.2111791024
H	-2.9241053488	1.8997048010	1.9544544308
H	-3.9276413927	2.3017775210	3.3764399434
C	-1.1176886633	-2.3829341583	-0.8844440601
C	-0.1942015399	-1.7331776641	-1.7150089143
C	-0.4145788767	-0.3756882600	-1.9989039432
C	-1.5060137960	0.3027782115	-1.4755775255
C	-2.4702086904	-0.3328637015	-0.6307398265
C	-2.2238072375	-1.7181200518	-0.3680448632
H	-0.9803870772	-3.4431648314	-0.6491014879
H	0.6569204176	-2.2687244768	-2.1414040692
H	0.2925261215	0.1672294633	-2.6320323006
H	-1.6419825594	1.3623360633	-1.7130450395
H	-2.9323241944	-2.2760981947	0.2492339141
C	-3.6356306108	0.3652246705	-0.2116575626
H	-3.5961775072	1.4491363410	-0.3630970601
C	-4.5375757151	-0.0937539500	0.8929525668
H	-5.4933288073	-0.4880386644	0.4807760323
H	-4.0851431679	-0.9313251364	1.4570684910
C	-8.3656172003	-3.3663906264	-1.3141786806
C	-7.9472802316	-2.0616337614	-1.5502585920
C	-6.5736105292	-1.7211602269	-1.6316520001
C	-5.6474590399	-2.7798075785	-1.4487352439
C	-6.0731126897	-4.0848696339	-1.2209378486
C	-7.4342996182	-4.3976911368	-1.1491952620
H	-9.4349900985	-3.5851598413	-1.2595214577
H	-8.6913051716	-1.2710662022	-1.6829258737
H	-4.5774743047	-2.5612407586	-1.4698096485
H	-5.3272501455	-4.8724031960	-1.0858250915
H	-7.7628320152	-5.4212870761	-0.9620982252
C	-6.1593084896	-0.3575917968	-1.8854953394
H	-6.9459483900	0.4015936150	-1.8493630091
C	-4.8558009586	0.0339104847	-2.1049426225
H	-4.6616184707	1.0559132715	-2.4406082127
H	-4.1103221297	-0.6983873375	-2.4270292007
C	2.4632160936	3.4447315357	0.3217880507
O	3.0779510338	3.3700031740	-1.9446400510
O	2.2478164674	2.1207883505	-0.2106015844
C	2.5449299871	4.3115800988	-0.9844603681
C	1.1699342199	4.7328664500	-1.4922449358
H	0.7195120692	5.4971422566	-0.8444149468
H	1.2782742694	5.1525692266	-2.5022983629
H	0.4856634483	3.8725843930	-1.5476980875
C	3.4716954952	5.5083048265	-0.8896324962
H	3.4573067869	6.0610470273	-1.8396554216
H	3.1364859000	6.1880744629	-0.0926349662
H	4.5051051589	5.2022347873	-0.6843281257
C	1.3134379637	3.8117111082	1.2421922526
H	1.3580443120	3.2024200074	2.1566134424
H	1.3823436954	4.8696198651	1.5354526171
H	0.3431054995	3.6377643739	0.7601131996
C	3.7788967709	3.4152982157	1.0912499818
H	3.9642680655	4.3696549013	1.6037463940
H	3.7244667226	2.6171082926	1.8461917780
H	4.6267491283	3.2032081485	0.4235466294
B	2.7147740584	2.1240624555	-1.5096226601
C	3.1883682703	0.7651910776	-3.5479457837
C	2.3240102370	1.6034712557	-4.4841504107
C	4.6593215101	1.1482671478	-3.6586895284
C	3.0007398490	-0.7228518998	-3.8145533797
H	1.2606981667	1.3516864011	-4.3557451841
H	2.4604115192	2.6755276377	-4.2859025325
H	2.6002761868	1.4032651422	-5.5292626966
H	5.2577777328	0.5188480178	-2.9845029229
H	5.0152781648	0.9953093918	-4.6875190484
H	4.8079009244	2.2048139239	-3.3922142083
H	3.3709669281	-0.9846081113	-4.8158673881
H	3.5568184789	-1.3066063483	-3.0646295521
H	1.9359740352	-0.9944026718	-3.7570774973
O	2.7534577837	0.9566510867	-2.1886228457
K	2.1624327664	-0.6442238146	-0.0430191299
O	3.3486934235	-0.4415462468	2.3757193861
C	4.5897708151	-0.7575049171	2.9785090964
C	2.6186116737	0.3802946073	3.2821652897
C	5.0048169618	0.5324004431	3.6731815687
H	4.4651388010	-1.5811650747	3.7077037883
H	5.2826040664	-1.0873072727	2.1927449592
C	3.6598628266	1.0542572492	4.2019749326
H	2.0284979922	1.0894024410	2.6847224492
H	1.9142315663	-0.2392796651	3.8624696510
H	5.4293426677	1.2266941279	2.9307673105
H	5.7499962602	0.3738337104	4.4632995863
H	3.5959598182	2.1502939733	4.1698883600
H	3.5057179745	0.7432400523	5.2441053484
O	2.7924157745	-3.2490084260	-0.1875341078
C	2.3825580783	-4.4720666867	-0.7730315566
C	3.6983232768	-3.5813558904	0.8547112107
C	3.6703503443	-5.2860552355	-0.8716169401
H	1.6452829851	-4.9763193663	-0.1193484654
H	1.9094723333	-4.2577278701	-1.7401036253
C	4.4474613485	-4.8472605183	0.3866427723
H	4.3465514963	-2.7106623993	1.0205935204
H	3.1355560047	-3.7812498698	1.7840285515
H	4.2146188936	-5.0051424210	-1.7847814653
H	3.4814041188	-6.3665164735	-0.9065370102
H	5.5013197124	-4.6371957299	0.1580525014
H	4.4253587557	-5.6194950058	1.1666728787
O	0.3972613874	-0.9877254197	1.8960396244
C	0.6046219455	-2.3318138549	2.3125961777
C	-0.7324964721	-0.4213627677	2.5553489070
C	-0.6885186194	-2.7492461755	3.0011104162
H	0.8521541826	-2.9419787820	1.4293232894
H	1.4624210935	-2.3678800112	3.0110392254
C	-1.1312779287	-1.4289242076	3.6322831923
H	-0.4528618444	0.5622915224	2.9665814895
H	-1.5382349179	-0.2738596940	1.8187546502
H	-0.5400523640	-3.5585734718	3.7279700730
H	-1.4250236002	-3.0772283567	2.2523579044
H	-0.5687344832	-1.2454146076	4.5611441059
H	-2.2038840985	-1.3937235753	3.8667588164
O	4.7759188047	-0.7752440564	-0.6228313843
C	5.4581995574	0.3617855714	-0.1108428434
C	5.7387813406	-1.6879882285	-1.1159921609
C	6.8855529216	-0.1097139030	0.2490017260
H	5.4857770105	1.1596319553	-0.8737638276
H	4.8828860479	0.7309723481	0.7508146361
C	6.8765628662	-1.6072955869	-0.1058314096
H	5.2601710177	-2.6736777334	-1.1981518387
H	6.0846787776	-1.3802385281	-2.1214087738
H	7.1250422372	0.0659034670	1.3068072541

H	7.6298395502	0.4273815571	-0.3542232428
H	6.6319596292	-2.2209949704	0.7754076780
H	7.8339146288	-1.9586680620	-0.5115091484

alpha-cyclopropyl styrene
M06-2X/def2SVP Electronic E: -425.853922 a.u.
M06-2X/def2SVP Gibbs free E: -425.691758 a.u.
M06-2X/def2TZVPP Electronic E: -426.326700 a.u.

C	-1.3714252357	-2.5297144822	1.3247687733
C	-2.4549373029	-2.9686077735	0.5614347912
C	-3.1600061979	-2.0520948449	-0.2175921687
C	-2.7888027628	-0.7076929858	-0.2310693017
C	-1.7119590168	-0.2495317732	0.5442221284
C	-1.0025574892	-1.1869815331	1.3128344565
H	-0.8017772257	-3.2410405481	1.9257476375
H	-2.7421327894	-4.0214996129	0.5681571428
H	-4.0066549252	-2.3841177325	-0.8213769650
H	-3.3531127041	-0.0006462964	-0.8411389582
H	-0.1366292324	-0.8621566143	1.8926017319
C	-1.3301415775	1.1930886371	0.5400063403
C	-0.7332854820	1.7599381689	1.5983292771
H	-0.4211501065	2.8057706004	1.5828912853
H	-0.5481692278	1.1993461458	2.5170469487
C	-1.6821247899	1.9600465983	-0.6948734297
C	-2.9841470775	2.7237095323	-0.7497520738
C	-1.6777416138	3.4569148701	-0.7408124300
H	-1.4136084024	1.4498158011	-1.6248483579
H	-3.5763746024	2.7447579394	0.1680289264
H	-3.5611953557	2.6881575973	-1.6759465385
H	-1.3459356267	3.9238125668	-1.6694944309
H	-1.4113702555	4.0044487392	0.1643912151

PrdE
M06-2X/def2SVP Electronic E: -1185.887179 a.u.
M06-2X/def2SVP Gibbs free E: -1185.385873 a.u.
M06-2X/def2TZVPP Electronic E: -1187.216702 a.u.

C	2.7899116050	2.2957572977	-2.6798552290
C	2.4906281533	3.2994078132	-1.7569661491
C	1.6918994529	2.9995762712	-0.6533718451
C	1.2001397176	1.7067750785	-0.4720950448
C	1.5046525907	0.6856415612	-1.3836763515
C	2.3068573185	1.0024302648	-2.4910161940
H	3.4052515636	2.5214310622	-3.5530789091
H	2.8788573958	4.3098337402	-1.8973124119
H	1.4574068008	3.7744449085	0.0793347527
H	0.5952515985	1.4709174435	0.4065050746
H	2.5507553086	0.2273362847	-3.2211959456
C	0.9904598100	-0.7044562903	-1.1819013262
C	2.0039332700	-1.8286050874	-1.2547497790
H	1.5292444916	-2.7579519305	-1.6037816080
H	2.7673828567	-1.5685869107	-2.0044417525
C	3.3621052997	0.8886628153	2.3712365877
C	2.8687165112	-0.3289102248	1.8923269647
C	3.3118694894	-0.8608172707	0.6762971572
C	4.2791821656	-0.1364316087	-0.0392457450
C	4.7683104441	1.0798097715	0.4287337996
C	4.3083900399	1.6012442296	1.6405443613
H	2.9946270009	1.2811917169	3.3215633134
H	2.1174643266	-0.8544575025	2.4818484093
H	4.6378913542	-0.5156608463	-0.9991808119
H	5.5076866529	1.6281439877	-0.1586419735
H	4.6867985099	2.5566145280	2.0088305235
C	2.7384007594	-2.1508046677	0.0822786052
C	3.8768551400	-3.1340303420	-0.2273059993
H	4.4068379275	-3.4166036529	0.6949780233
H	3.4723936670	-4.0500652148	-0.6852085578
H	4.6101576841	-2.7050976858	-0.9249724521
C	1.7680152560	-2.8365673679	1.0445018436
H	1.4214480393	-3.7853949966	0.6079171971

H	2.2580037357	-3.0665223271	2.0026291945
H	0.8858680905	-2.2139997038	1.2503326185
C	-0.3256529361	-0.8789083698	-0.9637159003
C	-1.0771632356	-2.1623848538	-0.7562027019
C	-1.9367032121	-2.1264983906	0.5171468542
H	-0.9554618804	0.0200026538	-0.9638284322
H	-0.3922048537	-3.0206016923	-0.7363284933
H	-1.7495182966	-2.3084125498	-1.6196157717
H	-2.4647638717	-3.0898161869	0.6337787442
H	-1.3071999102	-2.0193398014	1.4153009139
B	-3.0319334630	-0.9994987903	0.5123944832
O	-3.5887014298	-0.4605307979	1.6420226749
O	-3.5501890245	-0.4577698893	-0.6347435518
C	-4.3804804275	0.6768113103	1.2389352600
C	-4.6870610079	0.3497595047	-0.2632327405
C	-5.6065028596	0.7770067493	2.1282705658
C	-3.5004656010	1.9119494838	1.4054389335
C	-5.9277813344	-0.5206326701	-0.4356894166
C	-4.7603331010	1.5623181112	-1.1733495823
H	-6.1676622230	-0.1656069042	2.1484640740
H	-5.2969280739	1.0150296654	3.1559812703
H	-6.2716272532	1.5792813337	1.7759923665
H	-4.0523400993	2.8336108034	1.1741723094
H	-3.1566763569	1.9633870574	2.4481945874
H	-2.6151841741	1.8594090040	0.7540158908
H	-6.8470702757	0.0444456978	-0.2280058262
H	-5.9666325251	-0.8800880240	-1.4736927165
H	-5.8948303895	-1.3954618902	0.2303952111
H	-4.9857408836	1.2389754930	-2.1996302170
H	-5.5604940757	2.2416053602	-0.8440297485
H	-3.8112542530	2.1127764386	-1.1879263810

PrdZ
M06-2X/def2SVP Electronic E: -1185.886462 a.u.
M06-2X/def2SVP Gibbs free E: -1185.388615 a.u.
M06-2X/def2TZVPP Electronic E: -1187.216033 a.u.

C	2.8125439370	2.4035376179	-2.5932236991
C	2.6745803622	3.3493882482	-1.5765165232
C	1.9225615763	3.0366527912	-0.4431469848
C	1.3185369261	1.7851055872	-0.3258814646
C	1.4648501964	0.8193200430	-1.3316852253
C	2.2178247490	1.1488819808	-2.4681150081
H	3.3910322257	2.6424749460	-3.4878074871
H	3.1506760833	4.3274000483	-1.6675858818
H	1.8118567755	3.7682402926	0.3597805666
H	0.7467931763	1.5360965390	0.5711692651
H	2.3406333770	0.4107081361	-3.2644409726
C	0.8834071935	-0.5489864526	-1.1817210457
C	1.8500181255	-1.7145694651	-1.2498851111
H	1.3121240905	-2.6149850829	-1.5861593408
H	2.6221005114	-1.5048631074	-2.0074851204
C	3.6541389526	0.8349695036	2.3645935191
C	3.0039717953	-0.3160766050	1.9075965736
C	3.2874922358	-0.8544766349	0.6479415536
C	4.2586085586	-0.2081684416	-0.1348039164
C	4.9002753238	0.9434552124	0.3096539294
C	4.5988760367	1.4736458147	1.5671093496
H	3.4097893717	1.2346303263	3.3509379253
H	2.2580050869	-0.7835561611	2.5504449596
H	4.5001554593	-0.5979921549	-1.1264686088
H	5.6372283755	1.4338277398	-0.3297279285
H	5.0985030213	2.3783600332	1.9184717209
C	2.5607910221	-2.0800173939	0.0864065364
C	3.5851062007	-3.1850603052	-0.2153972413
H	4.1063210951	-3.4929298881	0.7037320373
H	3.0797650964	-4.0681342914	-0.6366876629
H	4.3409533079	-2.8498924980	-0.9406359894
C	1.5317910401	-2.6383830791	1.0709119230
H	1.0366216929	-3.5167320461	0.6290985932

H	2.0133687450	-2.9602680307	2.0064796433
H	0.7540707021	-1.9026160595	1.3202963274
C	-0.4286459098	-0.7630006286	-0.9837537340
C	-1.5442864527	0.2416503625	-0.9397748062
C	-2.6753795386	-0.1378622686	-1.9083778221
H	-0.7483107198	-1.8079593664	-0.8748136872
H	-1.1645814411	1.2485772781	-1.1634814364
H	-1.9540999576	0.2749583836	0.0847108843
H	-3.4744883185	0.6233143517	-1.8615768840
H	-2.3108761003	-0.1274882273	-2.9482838094
B	-3.3387860731	-1.5269661739	-1.5922224772
O	-3.9956738050	-2.3016022156	-2.5118883523
O	-3.3323757452	-2.0932247137	-0.3437661092
C	-4.2620319406	-3.5798003623	-1.8971539404
C	-4.2257892085	-3.2258210892	-0.3704198302
C	-5.5955227467	-4.1089024360	-2.3933625416
C	-3.1316973828	-4.5167273906	-2.3121556635
C	-5.5742550916	-2.7436283825	0.1544073506
C	-3.6709351673	-4.3219210328	0.5212262745
H	-6.3993018815	-3.3765570174	-2.2472628258
H	-5.5254979740	-4.3328398777	-3.4674253375
H	-5.8606248842	-5.0373155839	-1.8661711326
H	-3.2906563921	-5.5340683220	-1.9285046910
H	-3.0924912203	-4.5596044399	-3.4097542889
H	-2.1597404699	-4.1509826793	-1.9485381525
H	-6.2976892682	-3.5680692361	0.2209192199
H	-5.4329776375	-2.3218577937	1.1595500907
H	-5.9935846082	-1.9588749566	-0.4923863627
H	-3.6919342688	-3.9896495812	1.5689836442
H	-4.2823412270	-5.2325986589	0.4378528217
H	-2.6334496748	-4.5662793943	0.2599352478
H	3.7779204931	2.2091987252	1.0268543920
H	2.4826976312	2.2992336379	-0.1939229289
B	-1.5818416711	0.2415533890	0.0165264997
O	-2.1670676617	-0.1635461319	1.1861898676
O	-2.3758092014	0.0097495910	-1.0742337207
C	-3.5431799532	-0.4921983047	0.8917380482
C	-3.4831487547	-0.8068693052	-0.6426546765
C	-3.9768972829	-1.6568855172	1.7626119154
C	-4.3749867216	0.7472615622	1.2039019348
C	-3.1110963520	-2.2574391333	-0.9327021544
C	-4.7279386606	-0.4156992610	-1.4184609031
H	-3.2887263908	-2.5064597180	1.6709350088
H	-3.9971797673	-1.3420296263	2.8157701063
H	-4.9884625774	-1.9873689148	1.4839738455
H	-5.4483920762	0.5522074416	1.0720130146
H	-4.1994911213	1.0391269461	2.2492193774
H	-4.0882261085	1.5889093632	0.5565914873
H	-3.9428753863	-2.9383404333	-0.7038813756
H	-2.8637817063	-2.3524209865	-1.9995934985
H	-2.2329591701	-2.5689672688	-0.3482146462
H	-4.5989131525	-0.6811355571	-2.4774888866
H	-5.6061153540	-0.9553941171	-1.0341353167
H	-4.9188573766	0.6629001053	-1.3559133113
C	-0.6141028666	2.3748510456	-0.5183039277
C	-1.5833375395	3.1720464579	0.3154578205
C	-0.1905358250	3.6797767478	0.0880782687
H	-0.8296598072	2.4208640936	-1.5885609613
H	-1.8150099693	2.7957211176	1.3164698343
H	-2.4163570125	3.6630602780	-0.1913896883
H	-0.0472250270	4.5301909337	-0.5819963311
H	0.4751555988	3.6611370228	0.9487255092

PrdVCP
M06-2X/def2SVP Electronic E: -1185.865424 a.u.
M06-2X/def2SVP Gibbs free E: -1185.361741 a.u.
M06-2X/def2TZVPP Electronic E: -1187.191129 a.u.

C	1.3730256024	-1.8273017696	-2.1913349573
C	2.0822149790	-1.1195648840	-3.1663096672
C	2.0584142793	0.2726557820	-3.1504191926
C	1.3347357323	0.9563075681	-2.1683924485
C	0.6323622711	0.2641144189	-1.1766690396
C	0.6595875505	-1.1396934272	-1.2148631221
H	1.3786875346	-2.9192679056	-2.1920406696
H	2.6440232762	-1.6533196181	-3.9351395397
H	2.6022295682	0.8375532248	-3.9104172360
H	1.3243218073	2.0482294167	-2.1779081143
H	0.1165330786	-1.7053674571	-0.4510595783
C	-0.1683588783	0.9621886926	-0.0817246603
C	0.5114459756	0.9029002949	1.3203646773
H	0.0663927202	1.7027454103	1.9373146790
H	0.1865177355	-0.0265771842	1.8114769371
C	4.3755314067	-1.4964387703	-0.3369872799
C	3.7536370947	-0.3106119237	0.0622345090
C	2.7419440395	-0.3109676250	1.0302980452
C	2.3837434711	-1.5477245549	1.5919968194
C	2.9987014798	-2.7330586258	1.1958814537
C	4.0020535782	-2.7145236433	0.2253029960
H	5.1544292436	-1.4618446087	-1.1017547230
H	4.0601850497	0.6217085397	-0.4118806122
H	1.6009136164	-1.5931249007	2.3526304170
H	2.6941622044	-3.6777665821	1.6510802496
H	4.4868479587	-3.6411279127	-0.0875145514
C	2.0551281997	0.9762014251	1.5144842760
C	2.2912044966	1.0772517727	3.0352820646
H	3.3684704948	1.0727191565	3.2613146190
H	1.8614375681	2.0129451247	3.4252176695
H	1.8251068454	0.2414773263	3.5768759211
C	2.6872812200	2.2187463333	0.8825585185
H	2.3108345709	3.1248547233	1.3744249364

Int4VCP
M06-2X/def2SVP Electronic E: -1374.919530 a.u.
M06-2X/def2SVP Gibbs free E: -1374.592691 a.u.
M06-2X/def2TZVPP Electronic E: -1375.851438 a.u.

C	2.8016589987	0.0569072765	1.4633698845
C	3.8094434630	0.2958858880	0.5054138063
C	3.7021586271	-0.4108185110	-0.7090589181
C	2.6192979707	-1.2244244961	-1.0011011962
C	1.4881277565	-1.3863389845	-0.0909180126
C	1.7049488049	-0.7455310754	1.2014956938
H	2.8792504099	0.5226403308	2.4508827266
H	4.6706925919	0.9258830240	0.7266478736
H	4.4950506393	-0.3141359364	-1.4574704029
H	2.5924749204	-1.7323414604	-1.9654425843
H	0.9673728802	-0.8762464121	1.9931392371
C	0.3179182766	-2.0378627459	-0.4716236118
C	-0.8921465933	-2.1325561339	0.4247214380
H	-1.2727250752	-3.1711625690	0.4562183963
H	-0.6200441367	-1.9023912074	1.4658685285
C	-1.7320550440	2.4348661825	-0.9882626961
C	-1.9905316926	1.0592304388	-1.0453763128
C	-1.7887022475	0.2289642733	0.0687561232
C	-1.2979487175	0.8331212525	1.2410697515
C	-1.0360067605	2.2021847898	1.3049476098
C	-1.2572116868	3.0157152300	0.1883506402
H	-1.9123988843	3.0526510020	-1.8703701611
H	-2.3690885500	0.6412957888	-1.9780282621
H	-1.1271172278	0.2228623599	2.1299091475
H	-0.6618086426	2.6373891274	2.2337298145
H	-1.0652871425	4.0889946246	0.2387598702
C	-2.1381106139	-1.2621254681	0.0676542338
K	1.1919326932	1.3618324365	-0.7847498117
C	-3.2118686147	-1.5039559593	1.1431117749
H	-4.1146457841	-0.9054863584	0.9455291931
H	-3.5002863857	-2.5665858272	1.1503663445
H	-2.8457305175	-1.2499668535	2.1489074772
C	-2.7216735961	-1.7011204086	-1.2770593997

H	-2.9420942584	-2.7786415727	-1.2457007829
H	-3.6621427641	-1.1706708363	-1.4941050766
H	-2.0277445091	-1.5278691583	-2.1101325674
C	0.2721451390	-2.7062251464	-1.8151649224
C	1.1411237114	-3.9047640700	-2.1190307744
C	-0.3434004059	-4.0672393555	-1.9826574262
H	0.1345022281	-2.0565540543	-2.6950286765
H	1.7693410958	-4.2745706010	-1.3050510685
H	1.5796488489	-3.9936272073	-3.1163573829
H	-0.9262002471	-4.2660765798	-2.8851309322
H	-0.7152628276	-4.5669648870	-1.0850115058

Int5E
M06-2X/def2SVP Electronic E: -1374.883957 a.u.
M06-2X/def2SVP Gibbs free E: -1374.558219 a.u.
M06-2X/def2TZVPP Electronic E: -1375.819488 a.u.

C	3.9570346140	-0.2920508368	1.1800564657
C	4.4462675057	-0.1239445953	-0.1139715085
C	3.6071411090	-0.4068518376	-1.1912554752
C	2.2995656600	-0.8338691087	-0.9702774731
C	1.7732546759	-0.9905398797	0.3265826269
C	2.6455972853	-0.7159088113	1.3953787854
H	4.6022954648	-0.0956913842	2.0384847137
H	5.4694517920	0.2158804005	-0.2815025498
H	3.9686844707	-0.2944852655	-2.2150935363
H	1.6902563064	-1.0717415900	-1.8443835842
H	2.3137133786	-0.8499903416	2.4248725975
C	0.3566861776	-1.4384941097	0.5493299954
C	-0.3362612869	-0.9991737986	1.8348361224
H	-0.7871333779	-1.8811754885	2.3142602730
H	0.4393619433	-0.6601441156	2.5306625958
C	-2.1345305700	2.5800914319	-1.0510425421
C	-2.3261120718	1.5984139463	-0.0712806846
C	-1.2593347887	1.1409945620	0.7175370771
C	0.0066022601	1.7098261956	0.4927658869
C	0.2073935505	2.6752783980	-0.4940765786
C	-0.8663295561	3.1177930318	-1.2739426946
H	-2.9856243232	2.9186985483	-1.6451942430
H	-3.3267096249	1.1889807744	0.0666345953
H	0.8555193635	1.3923325100	1.1014836223
H	1.2061476130	3.0879208883	-0.6491368432
H	-0.7155580141	3.8790317326	-2.0412407358
C	-1.4460228560	0.1145324357	1.8343465477
K	-0.7661404266	0.0471458442	-2.2400793014
C	-1.3209792763	0.8656436386	3.1750421413
H	-2.1098478259	1.6265438747	3.2718625737
H	-1.4200815247	0.1570959890	4.0124374828
H	-0.3452924569	1.3663697053	3.2638387257
C	-2.8438591658	-0.5137544507	1.8009177590
H	-2.9240815278	-1.2650983819	2.6001751247
H	-3.6192048393	0.2447858736	1.9838964655
H	-3.0643876199	-1.0040151563	0.8431452436
C	-0.1985782256	-2.2834293478	-0.3469995109
C	-1.5921469655	-2.8336435172	-0.4093481769
C	-2.3941057838	-2.1424978688	-1.5329960758
H	0.4268053819	-2.5985300128	-1.1923222042
H	-2.0458456746	-2.8010773507	0.6024017396
H	-1.4972327753	-3.9044880206	-0.6620706295
H	-3.2779683661	-2.7670746002	-1.7666672147
H	-2.8239246285	-1.2004059107	-1.1226035985

Int5VCP
M06-2X/def2SVP Electronic E: -2018.583865 a.u.
M06-2X/def2SVP Gibbs free E: -2017.960276 a.u.
M06-2X/def2TZVPP Electronic E: -2020.249125 a.u.

C	-1.7445332917	-0.9725213107	2.7712887458
C	-1.1924865699	-0.4626690328	3.9512418423
C	-0.2913599210	0.6016316116	3.8613528266
C	0.0534695660	1.1409104848	2.6188610587
C	-0.4800872755	0.6359104437	1.4140827984
C	-1.3913534145	-0.4348598058	1.5329684231
H	-2.4553037725	-1.8014905520	2.8115186620
H	-1.4669593998	-0.8803046059	4.9214634562
H	0.1506643322	1.0201431739	4.7683924592
H	0.7726271356	1.9582203608	2.5802392689
H	-1.8085930754	-0.8796699209	0.6320780234
C	0.0056783218	1.1031458662	0.0705890128
C	-1.0132697148	0.9426563436	-1.0806427920
H	-0.5148678898	1.3681237132	-1.9649773291
H	-1.0842638678	-0.1335919481	-1.2898892485
C	-5.2520807451	-0.7636460035	0.4798920280
C	-4.3688906521	0.2999161959	0.2803141615
C	-3.4914036112	0.3249393888	-0.8124047026
C	-3.5486366317	-0.7562217912	-1.7070366749
C	-4.4302556942	-1.8190048720	-1.5161891082
C	-5.2878025515	-1.8308661295	-0.4153045381
H	-5.9155529235	-0.7543675542	1.3471880028
H	-4.3596601363	1.1088402645	1.0104757236
H	-2.8797878686	-0.7775711052	-2.5707686402
H	-4.4432377126	-2.6447753674	-2.2306854862
H	-5.9785451113	-2.6616314300	-0.2602123381
C	-2.5024388207	1.4753367677	-1.0691579641
K	1.3182826876	-1.6951058738	2.2570502687
C	-2.8008801647	2.0178430454	-2.4817774712
H	-3.8557150204	2.3208325390	-2.5745564997
H	-2.1706432590	2.8965479048	-2.6897598905
H	-2.5903893208	1.2684063653	-3.2587509076
C	-2.7550941071	2.6192400357	-0.0876940811
H	-2.1710131728	3.5009566231	-0.3744369827
H	-3.8148415751	2.9162068495	-0.1117223520
H	-2.4979059693	2.3503014405	0.9468878586
B	1.2922867461	0.0094684939	-0.3166542988
O	1.9784526563	0.3686908804	-1.5376319309
O	2.3236582431	0.1519397268	0.7462395748
C	3.3649835262	0.5508917428	-1.3487547911
C	3.6182110213	0.1939093440	0.1821507684
C	4.1266894236	-0.3584554159	-2.3157970588
C	3.7142759008	1.9995179432	-1.7064859831
C	4.2767145647	-1.1752217132	0.3905094947
C	4.4491092152	1.2368680950	0.9276960963
H	3.8623815281	-1.4145594298	-2.1744760647
H	3.8675992308	-0.0796884987	-3.3483048932
H	5.2158576277	-0.2520649034	-2.1978083796
H	4.7959351324	2.1898999294	-1.6483304439
H	3.3853931815	2.1867385747	-2.7396074192
H	3.1949481447	2.7142008729	-1.0570390744
H	5.2877305997	-1.2300234993	-0.0378480904
H	4.3703388423	-1.3624176527	1.4733428945
H	3.6672105777	-1.9743694440	-0.0493435465
H	4.5457438688	0.9373685418	1.9824536974
H	5.4611770396	1.3204488490	0.5039200711
H	3.9774273463	2.2277346972	0.9005290536
O	0.7885648610	-1.3944260592	-0.2712873094
C	0.5792121988	-2.3998775168	-1.2442791506
C	1.6841890001	-3.4581129774	-1.1273811583
H	1.4657398859	-4.3277373073	-1.7653925818
H	2.6541077944	-3.0477935321	-1.4413804361
H	1.7749733833	-3.8181556005	-0.0889214752
C	0.5483342863	-1.8956432947	-2.6908551182
H	0.3065991466	-2.7359687013	-3.3597122934
H	-0.2148293648	-1.1174129957	-2.8280733943
H	1.5139095309	-1.4677495878	-2.9821924930
C	-0.7627343407	-3.0602083613	-0.9118671948
H	-0.9671087682	-3.9134313309	-1.5761367839
H	-0.7626229496	-3.4250849524	0.1287002005
H	-1.5833392859	-2.3366027832	-1.0182217053
C	0.7184557699	2.4620699939	0.1480072291
C	0.4580206169	3.6372608016	-0.7432545377

C	0.2044531341	3.7519506065	0.7347721590
H	1.7770227794	2.3302365296	0.3772663030
H	-0.4104588708	3.5934588675	-1.4044560584
H	1.3120973200	4.1698325773	-1.1692998553
H	0.9098599249	4.3420856331	1.3257302769
H	-0.8247522722	3.8099997422	1.0874540872

Int5Z
M06-2X/def2SVP Electronic E: -1374.890970 a.u.
M06-2X/def2SVP Gibbs free E: -1374.567296 a.u.
M06-2X/def2TZVPP Electronic E: -1375.826319 a.u.

C	3.3212567544	1.4965243651	-0.9946783543
C	3.3778656604	2.8657359359	-0.7365019181
C	2.1901413032	3.5928262298	-0.6490887896
C	0.9604894127	2.9540343299	-0.7974846758
C	0.8819820892	1.5733564707	-1.0447899389
C	2.0893541621	0.8614652586	-1.1522625478
H	4.2409892752	0.9147015387	-1.0797587206
H	4.3404943056	3.3645321089	-0.6115094337
H	2.2197843479	4.6680157471	-0.4622041980
H	0.0458763755	3.5443713803	-0.7231387247
H	2.0685789766	-0.2056058244	-1.3908027949
C	-0.4406127496	0.8889352163	-1.1685631271
C	-1.5640925880	1.3369552922	-0.2499599134
H	-1.6643833637	2.4336549672	-0.3035505539
H	-2.5059115441	0.9408228788	-0.6548587485
C	-0.5853589386	-2.4816670212	2.6879286804
C	-0.5789169761	-1.1027307829	2.4448612182
C	-1.4343327999	-0.5258573318	1.4930901112
C	-2.2974334042	-1.3865977400	0.7902441524
C	-2.3009562879	-2.7622926209	1.0218625826
C	-1.4426179166	-3.3196321804	1.9753645447
H	0.0878720472	-2.8978576624	3.4398712287
H	0.1062924219	-0.4781780869	3.0177676009
H	-2.9865588299	-0.9785418329	0.0489090654
H	-2.9791697173	-3.4028526567	0.4552143731
H	-1.4473900933	-4.3948403744	2.1614260347
C	-1.4969935857	0.9864219752	1.2634622709
K	0.6446268887	-2.2070529581	-0.2028340697
C	-2.8087812018	1.4910715334	1.8938845315
H	-2.8351575581	1.2660691385	2.9705497218
H	-2.8956944587	2.5812301145	1.7661574650
H	-3.6842360944	1.0195907168	1.4228364006
C	-0.3356721616	1.7113473762	1.9511037989
H	-0.3802450190	2.7851873792	1.7190906304
H	-0.4016960166	1.6065575661	3.0441882246
H	0.6470294004	1.3373286242	1.6286610976
C	-0.6913364118	-0.0391381056	-2.1198378289
C	0.1802848104	-0.5692409755	-3.2235615891
C	0.5802741313	-2.0478626968	-3.0049353753
H	-1.7038833483	-0.4685254489	-2.1074707632
H	1.0763536170	0.0642878501	-3.3227131140
H	-0.4024508750	-0.3972333455	-4.1577395906
H	-0.3588102092	-2.6460619130	-3.0775710641
H	1.1558521698	-2.3666554353	-3.8988888992

Int6E
M06-2X/def2SVP Electronic E: -2018.562235 a.u.
M06-2X/def2SVP Gibbs free E: -2017.948984 a.u.
M06-2X/def2TZVPP Electronic E: -2020.237086 a.u.

C	1.7282743778	3.2521005059	-1.5548907553
C	2.8788580665	4.0389848380	-1.5631827161
C	3.8933579987	3.7717833309	-0.6420271665
C	3.7645521647	2.7206275551	0.2628100314
C	2.6161114454	1.9110672539	0.2807528574
C	1.5994521066	2.2048601394	-0.6410605180
H	0.9235242158	3.4497452344	-2.2659513489
H	2.9835927695	4.8562156496	-2.2787259793
H	4.7943299076	4.3881792956	-0.6275527533
H	4.5702934464	2.5294657312	0.9739323461
H	0.6760960166	1.6206569795	-0.6450623990
C	2.4876582554	0.7901025908	1.2635900570
C	3.6982627256	-0.0922968843	1.4822383823
H	4.5661300259	0.5315533160	1.7556799239
H	3.5131498892	-0.7380024295	2.3501840339
C	1.6338739661	-2.9018253324	-1.9106806551
C	2.6113671799	-1.9868019420	-1.5021138233
C	3.0266476931	-1.9151498529	-0.1631951131
C	2.4026451605	-2.7744124189	0.7582094160
C	1.4313680890	-3.6909327933	0.3551118346
C	1.0467423889	-3.7661930369	-0.9869136557
H	1.3395238048	-2.9404486279	-2.9613382249
H	3.0579673491	-1.3348825043	-2.2525064302
H	2.6818953565	-2.7335138997	1.8136328129
H	0.9701660775	-4.3476604773	1.0952625219
H	0.2953824333	-4.4898550097	-1.3067604019
C	4.1594685360	-0.9954048094	0.3010664667
K	-0.1153080079	-0.9307128807	-0.0975554102
C	5.3005310715	-1.8803842430	0.8369915907
H	4.9756022544	-2.4850502565	1.6960876053
H	5.6649536187	-2.5622525363	0.0539798422
H	6.1422679377	-1.2506804377	1.1643204496
C	4.7317462067	-0.1739955146	-0.8592485480
H	5.5181495891	0.4973019807	-0.4841012946
H	5.1881385390	-0.8385361941	-1.6088746529
H	3.9789597292	0.4437564115	-1.3658616043
C	1.3357241872	0.6697145191	1.9600701365
C	0.9305107873	-0.2890238780	3.0497151916
C	-0.3375545404	-1.1081767043	2.7215024994
H	0.5471855143	1.3981934620	1.7230067490
H	0.6967113721	0.3575201322	3.9167068261
H	1.8100965486	-0.8858061760	3.3651498237
H	-0.0193253209	-2.0762845718	2.2724058245
H	-0.8004128460	-1.4085067128	3.6815636057
B	-3.0083535472	0.0880154505	-0.4663637667
O	-1.9957296486	1.0265064851	-0.4691164875
O	-4.2248996707	0.6045635955	-0.1239028839
C	-2.6172074661	2.3132501305	-0.2305943729
C	-3.9653478198	1.9023130731	0.4602699813
C	-1.7165470690	3.1630025686	0.6452009065
C	-2.8260995951	2.9675032474	-1.5907284598
C	-3.8119911550	1.6868562169	1.9624164875
C	-5.1310563491	2.8252065137	0.1617510900
H	-1.4153525972	2.6205741373	1.5506604110
H	-0.8122909012	3.4532558340	0.0917105500
H	-2.2416498573	4.0830901015	0.9422986736
H	-3.2411812701	3.9793863437	-1.4861858637
H	-1.8557516744	3.0417047263	-2.1022854891
H	-3.5038110135	2.3715309829	-2.2196601413
H	-3.6624789972	2.6388407379	2.4901395047
H	-4.7292547231	1.2184826994	2.3469858952
H	-2.9635763808	1.0197016706	2.1849414807
H	-6.0262337721	2.4675061739	0.6899646142
H	-4.9107330429	3.8441120588	0.5125149226
H	-5.3544343422	2.8593781051	-0.9119723599
O	-2.7105898893	-1.1976341568	-0.7802980544
C	-3.5977619570	-2.3202663305	-0.5696786254
C	-4.7601576325	-2.2174725516	-1.5495961474
H	-5.4260495254	-3.0852545778	-1.4393180723
H	-5.3431246974	-1.3052342044	-1.3596387276
H	-4.3870770693	-2.1926576552	-2.5841571754
C	-4.0789512274	-2.3307895332	0.8781739926
H	-4.6356665231	-3.2575104080	1.0785587755
H	-3.2180315409	-2.2800363687	1.5625660521
H	-4.7370453649	-1.4758197233	1.0814647756
C	-2.7459303346	-3.5496860965	-0.8543223075
H	-3.3514115509	-4.4634175095	-0.7756040382
H	-2.3227715183	-3.4953056981	-1.8685282343

H	-1.9234203629	-3.6213928408	-0.1247022840

Int6Z
M06-2X/def2SVP Electronic E: -2018.564034 a.u.
M06-2X/def2SVP Gibbs free E: -2017.950181 a.u.
M06-2X/def2TZVPP Electronic E: -2020.238979 a.u.

C	3.2339288604	0.5288420810	-1.6764535783
C	2.5273972440	1.4598873692	-2.3547465794
C	1.8537183305	1.3577733090	-3.6968617888
C	0.3156754575	1.4110016552	-3.6307494689
H	2.4643162682	2.4545866428	-1.8887398816
H	2.2805967873	0.4867751367	-4.2366074289
H	2.1970933537	2.2463406319	-4.2592538891
H	-0.0558222551	1.5826969190	-4.6611930902
H	-0.0492797202	0.3820779365	-3.3922487862
B	-2.9002644747	0.0331457584	0.3895945895
O	-4.0072950659	-0.3552151582	1.0895532025
O	-2.8610448774	1.3908348561	0.1452997533
C	-4.6661104308	0.8559971810	1.5325168884
C	-4.1720406049	1.9050841412	0.4765547781
C	-6.1656029663	0.6307948210	1.5447661242
C	-4.1516151900	1.1557304793	2.9353882424
C	-4.9995164699	1.8816002874	-0.8034928065
C	-4.0423881477	3.3210019010	1.0035898371
H	-6.5233483732	0.2538123985	0.5785134314
H	-6.4207690684	-0.1060048850	2.3196417745
H	-6.6901510221	1.5691009594	1.7773508987
H	-4.6592905843	2.0275625933	3.3703812724
H	-4.3414951109	0.2848178514	3.5785636440
H	-3.0678924262	1.3493942192	2.9237219148
H	-5.9963261988	2.3141536355	-0.6417273109
H	-4.4834012560	2.4739318809	-1.5721084535
H	-5.1189080231	0.8551500990	-1.1812096019
H	-3.6982236072	3.9849070691	0.1975695491
H	-5.0190837274	3.6864303890	1.3533612164
H	-3.3264024603	3.3816857676	1.8323494132
O	-1.8888565758	-0.7405230828	-0.0732893366
C	-1.7846616647	-2.1726197588	0.0880251068
C	-2.9951005195	-2.8385593156	-0.5568855155
H	-2.8804076604	-3.9314507495	-0.5297153472
H	-3.9187808735	-2.5715495212	-0.0252417888
H	-3.0845406926	-2.5249759094	-1.6074754064
C	-1.6881361701	-2.5090201276	1.5718563610
H	-1.5913138303	-3.5959435656	1.7047042265
H	-0.8054697706	-2.0276950375	2.0179162106
H	-2.5877782088	-2.1719166724	2.1058594333
C	-0.5047165761	-2.5512201251	-0.6435051933
H	-0.3087136696	-3.6291173837	-0.5544262762
H	-0.5899008799	-2.2972765972	-1.7106935826
H	0.3527228147	-2.0057867321	-0.2175949031
K	-0.3239908134	1.3348999093	-0.8741881747
C	3.9861328678	0.9082119041	-0.4149332190
H	5.0241788421	0.5460068026	-0.4965969180
C	3.4587591821	0.3951999378	0.9518754689
C	2.1085984909	1.0243077233	1.3108664241
C	4.4682592533	0.8493435552	2.0231566028
C	3.3989630554	-1.1344216151	0.9758275194
C	1.0428639436	0.2693899701	1.8234108162
C	1.9332780318	2.4179388046	1.2265755510
H	4.1312935324	0.5521488900	3.0276225974
H	5.4507467209	0.3881043063	1.8379855495
H	4.5957142836	1.9422602628	2.0136491261
H	4.3658640417	-1.5486124651	0.6550566708
H	3.1984232674	-1.5043976525	1.9924907996
H	2.6252139020	-1.5342024735	0.3039882253
C	-0.1398232430	0.8795536962	2.2564570273
H	1.1306099510	-0.8144151693	1.9052890802
C	0.7549235231	3.0307199261	1.6530375115
H	2.7370628690	3.0448229546	0.8364117832
C	-0.2882089897	2.2627004206	2.1790643022
H	-0.9480037068	0.2633165533	2.6586311646
H	0.6529090999	4.1149548955	1.5779102017
H	-1.2083927865	2.7402466447	2.5191555395
H	4.0613714520	2.0039793432	-0.3720384739
C	3.3908963729	-0.8665974485	-2.1857182575
C	2.2784076894	-1.5950011356	-2.6365661585
C	4.6426441759	-1.5025615788	-2.2094427458
C	2.4058509823	-2.9091486755	-3.0833649718
H	1.2964117104	-1.1181481365	-2.6327817946
C	4.7748791126	-2.8151264269	-2.6621365585
H	5.5329817332	-0.9641425456	-1.8787334213
C	3.6565354245	-3.5281572418	-3.0948969521
H	1.5211622464	-3.4523508442	-3.4221857666
H	5.7609175726	-3.2836664843	-2.6763138307
H	3.7594782453	-4.5580179549	-3.4413865728

TS4VCP_5E
M06-2X/def2SVP Electronic E: -1374.877543 a.u.
M06-2X/def2SVP Gibbs free E: -1374.551799 a.u.
M06-2X/def2TZVPP Electronic E: -1375.812388 a.u.

C	3.8640282604	0.1722734911	0.8141393265
C	4.4292760742	-0.0366265811	-0.4463600386
C	3.6757499844	-0.7306848491	-1.3916585188
C	2.3947150787	-1.1918384414	-1.0908849647
C	1.7817003406	-0.9848257481	0.1773938253
C	2.5856198539	-0.2844413238	1.1157590364
H	4.4305060492	0.7005835437	1.5845446914
H	5.4307901328	0.3260366584	-0.6812443637
H	4.0863088899	-0.9245620584	-2.3853614960
H	1.8724974385	-1.7499277487	-1.8716695234
H	2.2093070737	-0.0935489476	2.1203936452
C	0.4243638167	-1.4687986866	0.4840410373
C	-0.2658943730	-1.0418826439	1.7716469952
H	-0.7589827054	-1.9208338483	2.2165756899
H	0.5085103837	-0.7717580574	2.5012335465
C	-1.8893498582	2.5786410578	-1.1004461521
C	-2.1214010862	1.5719272921	-0.1548935467
C	-1.1246161536	1.1803508806	0.7523851192
C	0.1103829206	1.8476617565	0.6821425942
C	0.3548693784	2.8397472127	-0.2670987207
C	-0.6485707330	3.2125589454	-1.1681317581
H	-2.6878067618	2.8623807919	-1.7888210255
H	-3.0991276923	1.0912016740	-0.1392408050
H	0.9025698121	1.5819421078	1.3821154242
H	1.3315474674	3.3266772006	-0.2990452716
H	-0.4658613072	3.9941664932	-1.9075043861
C	-1.3485155835	0.1066190173	1.8172751216
K	-0.1098444158	0.1672659614	-2.0221100468
C	-1.2373266672	0.7766835000	3.1998855514
H	-2.0093150732	1.5508052716	3.3275743542
H	-1.3718001347	0.0228321960	3.9915383697
H	-0.2522906749	1.2452277758	3.3440952099
C	-2.7600469416	-0.4829151446	1.7215007553
H	-2.8882152851	-1.2512919143	2.4982804555
H	-3.5230452761	0.2919274827	1.8915313815
H	-2.9610876800	-0.9476205782	0.7469478860
C	-0.1478141858	-2.4108173075	-0.3550392788
C	-1.5273945554	-2.9579700644	-0.3530424107
C	-1.9174781629	-2.0725634273	-1.5245249744
H	0.4789617811	-2.8719663694	-1.1205239134
H	-2.0760016864	-2.8223617107	0.5908558368
H	-1.5370583852	-4.0278647422	-0.6062113084
H	-2.3737675944	-2.5979524175	-2.3756790084
H	-2.4983737633	-1.1872497000	-1.2237383412

TS4VCP_5VCP
M06-2X/def2SVP Electronic E: -2018.564193 a.u.
M06-2X/def2SVP Gibbs free E: -2017.945043 a.u.

M06-2X/def2TZVPP Electronic E: -2020.232063 a.u.

C	-1.7230199456	-0.6731946756	2.9231025241
C	-1.1092010370	-0.1354181484	4.0721563264
C	-0.2097376629	0.9247553622	3.8842928223
C	0.0956495470	1.4099701779	2.6151788533
C	-0.5011146426	0.8717432016	1.4173488880
C	-1.4411338026	-0.1929253895	1.6536235707
H	-2.4430950906	-1.4905244117	3.0268416313
H	-1.3538307194	-0.5025067422	5.0695614413
H	0.2706044453	1.3856733079	4.7524610874
H	0.8050986941	2.2325577181	2.5292087600
H	-1.9306010095	-0.6556994121	0.7974280424
C	-0.1637111620	1.3340956190	0.1201391872
C	-1.0285392786	1.0008877748	-1.0774976272
H	-0.4898457329	1.3552066063	-1.9708949332
H	-1.0892030659	-0.0931257262	-1.1978798300
C	-5.2953526835	-0.5338692565	0.5108021127
C	-4.4201298214	0.5138545922	0.2162192315
C	-3.5269347037	0.4374200265	-0.8612062098
C	-3.5690827380	-0.7246782416	-1.6493213069
C	-4.4372054674	-1.7764024393	-1.3589619657
C	-5.3045097494	-1.6891708859	-0.2687667433
H	-5.9709824721	-0.4443688615	1.3640459387
H	-4.4262954229	1.3921028856	0.8615497639
H	-2.9029096914	-0.8131919228	-2.5098484301
H	-4.4364838188	-2.6678169020	-1.9901206002
H	-5.9854696426	-2.5097630534	-0.0355923749
C	-2.5206796335	1.5447389986	-1.1973889767
K	1.2772967174	-1.3377029747	2.5644590617
C	-2.7470840522	1.9592025857	-2.6630025399
H	-3.7902088689	2.2686010890	-2.8354351041
H	-2.0907379679	2.8061630202	-2.9186718635
H	-2.5140145942	1.1399945967	-3.3592192468
C	-2.7592566711	2.7756610555	-0.3249081162
H	-2.1031105209	3.5960525236	-0.6428933772
H	-3.7977462266	3.1287527777	-0.4245521695
H	-2.5597742704	2.5737056176	0.7371551976
B	1.4877165776	-0.5225257152	-0.3742941670
O	1.9405799533	-0.4164144274	-1.6796777911
O	2.4743178019	-0.0754427607	0.5309282706
C	3.2619683423	0.1374442849	-1.6979698518
C	3.7142934689	0.0647065184	-0.1804449769
C	4.1222141045	-0.7017146082	-2.6353865495
C	3.1784686891	1.5557262875	-2.2473173350
C	4.5526889081	-1.1728654994	0.1397154252
C	4.4517664632	1.3023047334	0.3072221156
H	4.1318587032	-1.7617914434	-2.3527706830
H	3.7232069410	-0.6228766508	-3.6568945361
H	5.1582300255	-0.3320664903	-2.6442228656
H	4.1754621987	2.0083452583	-2.3442141665
H	2.7167483917	1.5174397798	-3.2448341778
H	2.5559082729	2.1891373605	-1.6085120803
H	5.5356250985	-1.1384052980	-0.3502412238
H	4.7168416698	-1.2168252799	1.2272867135
H	4.0400205288	-2.0935641588	-0.1699470480
H	4.6910831584	1.1895259376	1.3748780154
H	5.3965518776	1.4244839958	-0.2427219712
H	3.8567701876	2.2150521663	0.1821228429
O	0.6165531810	-1.5304834211	0.0225078261
C	0.4006145859	-2.7760530201	-0.6590746207
C	1.7327793918	-3.5012603608	-0.8403153306
H	1.5686015881	-4.5023640570	-1.2645341997
H	2.3872464006	-2.9391272455	-1.5207822698
H	2.2437938260	-3.6219776915	0.1284801956
C	-0.2768905964	-2.5579884213	-2.0081312028
H	-0.3695208338	-3.5179481363	-2.5374259573
H	-1.2860075186	-2.1495449145	-1.8643239868
H	0.3078449107	-1.8653827917	-2.6275948021
C	-0.5178307984	-3.5824081346	0.2538848737
H	-0.7840244570	-4.5399227664	-0.2162142103
H	-0.0267625667	-3.8084347383	1.2150569253
H	-1.4418431747	-3.0204337367	0.4541892362
C	0.7404282989	2.5364393228	0.0758270235
C	0.5941855781	3.6560155443	-0.9119809423
C	0.3385304726	3.9204233443	0.5438336777
H	1.7815084509	2.3096375419	0.3418123711
H	-0.2637093124	3.6266347366	-1.5886714278
H	1.4944973086	4.0856514179	-1.3583580883
H	1.0799230637	4.5118985623	1.0876928925
H	-0.6912753989	4.0690054817	0.8733350320

TS4VCP_5Z

M06-2X/def2SVP Electronic E: -1374.873169 a.u.
M06-2X/def2SVP Gibbs free E: -1374.548495 a.u.
M06-2X/def2TZVPP Electronic E: -1375.807708 a.u.

C	4.3105557405	-1.3709884231	-0.1219263455
C	4.4708684593	-0.1144756992	0.4586929789
C	3.3273123537	0.5560720975	0.9093674575
C	2.0680354121	-0.0172605384	0.7948996436
C	1.8659921326	-1.2971346114	0.2110350235
C	3.0456567033	-1.9476067828	-0.2485655404
H	5.1814208620	-1.9189876582	-0.4896545428
H	5.4591594014	0.3375067569	0.5538573358
H	3.4203786767	1.5491817367	1.3554841367
H	1.2065229232	0.5460760057	1.1523796470
H	2.9780756265	-2.9267480540	-0.7256250024
C	0.5471592790	-1.9162412395	0.1232768919
C	-0.5960385388	-1.4354659859	1.0121698143
H	-0.6778666144	-0.3341555502	0.9584760164
H	-1.5321066771	-1.7950430623	0.5587600806
C	0.3609052539	-5.1908164510	4.0120199691
C	0.2425360440	-3.8091386450	3.8192345752
C	-0.5407301304	-3.2817124725	2.7797246046
C	-1.2243517753	-4.1943758436	1.9573252382
C	-1.1033459665	-5.5716079554	2.1391882538
C	-0.3018161569	-6.0796869246	3.1660053948
H	0.9805106370	-5.5688092461	4.8275904226
H	0.7855576514	-3.1441651241	4.4906920794
H	-1.8537007801	-3.8240712922	1.1468982183
H	-1.6332550700	-6.2522647776	1.4704841491
H	-0.2023146021	-7.1570516015	3.3077273777
C	-0.6767794692	-1.7780326424	2.5352558362
K	1.8484242484	-4.3561002412	1.2990239791
C	-2.0752667504	-1.3509817039	3.0182493883
H	-2.1951825695	-1.5426439706	4.0953640111
H	-2.2257753340	-0.2744929832	2.8406101357
H	-2.8654323780	-1.8985905928	2.4825892944
C	0.3559109080	-0.9867820362	3.3428433722
H	0.2477053729	0.0862390755	3.1293995520
H	0.2019571035	-1.1197588498	4.4241538614
H	1.3902238156	-1.2766484232	3.1037384258
C	0.2157104963	-2.8430696882	-0.8770778026
C	0.8842721422	-3.2688421472	-2.1250967318
C	1.1331460027	-4.6121032878	-1.4519466130
H	-0.8173205919	-3.2047118177	-0.8285083320
H	1.7906785886	-2.7054124845	-2.3756586484
H	0.2231854773	-3.2686439294	-3.0065548887
H	0.4475965372	-5.4072666799	-1.7714747437
H	2.1847515552	-4.9447322556	-1.4822979738

TS6E_PrdE

M06-2X/def2SVP Electronic E: -2018.556889 a.u.
M06-2X/def2SVP Gibbs free E: -2017.942439 a.u.
M06-2X/def2TZVPP Electronic E: -2020.231929 a.u.

C	1.9104619316	3.3271837613	-1.8130400630
C	3.0880110834	4.0654030393	-1.7018997899
C	3.9783566823	3.7728801741	-0.6674172203
C	3.7020420098	2.7456579984	0.2322092341

C	2.5261044859	1.9825284979	0.1309773962
C	1.6339982154	2.3034144715	-0.9062598774
H	1.1991011075	3.5448073997	-2.6124481364
H	3.3084629238	4.8642289267	-2.4118868808
H	4.8980077859	4.3510990520	-0.5585118051
H	4.4135370985	2.5372595222	1.0327667050
H	0.6883039586	1.7636402562	-1.0007746404
C	2.2253486110	0.8978023741	1.1162976632
C	3.3684799485	0.0393401567	1.6155888458
H	4.1631733453	0.6831141507	2.0294721073
H	3.0073855810	-0.5598706393	2.4615911347
C	2.0594162959	-2.9621227655	-1.9874923413
C	2.9357394106	-2.0250671921	-1.4277916015
C	3.0505372380	-1.8756686316	-0.0365623890
C	2.2344637728	-2.6843633498	0.7734326479
C	1.3616656441	-3.6216816913	0.2204942453
C	1.2733373216	-3.7710145756	-1.1667695771
H	2.0004841677	-3.0613496945	-3.0731660506
H	3.5425203302	-1.4171700726	-2.0983588345
H	2.2815677199	-2.5855726201	1.8603780578
H	0.7439072064	-4.2358413003	0.8785478146
H	0.5975854488	-4.5092758147	-1.6018507292
C	4.0628180873	-0.9256995268	0.6097922341
K	0.0042150001	-0.8821759869	-0.7359478213
C	5.0556707340	-1.7753255127	1.4251357033
H	4.5475334960	-2.3373606487	2.2222214824
H	5.5784177049	-2.4941183916	0.7763537528
H	5.8089280033	-1.1247022579	1.8957941762
C	4.8745721346	-0.1678769882	-0.4454827147
H	5.5744771558	0.5211029452	0.0493354381
H	5.4689245386	-0.8711631259	-1.0486567159
H	4.2495103418	0.4222096311	-1.1282942730
C	0.9621443457	0.7935220152	1.5870186534
C	0.3343921514	-0.1379995093	2.5889330681
C	-0.8254877388	-0.9396702305	1.9536589938
H	0.2291778954	1.4964414016	1.1696006478
H	-0.0847841812	0.5227098227	3.3720472709
H	1.1147073508	-0.7326342573	3.1041888611
H	-0.4443906898	-1.9533606577	1.6897688807
H	-1.5900888945	-1.1298668722	2.7322426796
B	-2.7674741429	-0.0319831747	-0.1702172526
O	-1.9618560746	1.0012309861	-0.6519932676
O	-3.8294777921	0.4333917300	0.5645480674
C	-2.6908014260	2.2241253333	-0.4221554237
C	-3.6111287021	1.8388173154	0.7884935397
C	-1.7323887282	3.3657619820	-0.1387483549
C	-3.4897512411	2.5111841634	-1.6913474626
C	-2.9079417379	1.9958679595	2.1322292093
C	-4.9536305239	2.5489978692	0.8030897058
H	-1.0432418210	3.1290846881	0.6817375956
H	-1.1399960178	3.5918593336	-1.0373182593
H	-2.2961770061	4.2717573282	0.1303162534
H	-4.0319437408	3.4644945674	-1.6217366368
H	-2.7952019913	2.5674108277	-2.5417817295
H	-4.2144849817	1.7077417698	-1.8917949535
H	-2.7795551708	3.0554583738	2.3954418850
H	-3.5214949011	1.5149642247	2.9080935714
H	-1.9313868348	1.4915739176	2.1120116144
H	-5.5245600954	2.2350518402	1.6887437120
H	-4.8121942564	3.6387399402	0.8564337383
H	-5.5464774224	2.3108744761	-0.0893001828
O	-2.6436316981	-1.2677837281	-0.7394395838
C	-3.5119744218	-2.3849034937	-0.4928792256
C	-4.8720254303	-2.0876055229	-1.1194862428
H	-5.5426680078	-2.9524620263	-1.0118368389
H	-5.3364617649	-1.2208599229	-0.6283557111
H	-4.7559614597	-1.8668146202	-2.1911980114
C	-3.6454884028	-2.6584072616	1.0021894723
H	-4.2972653266	-3.5308829412	1.1582421185
H	-2.6615003764	-2.8639101688	1.4451630325
H	-4.0836585501	-1.7934577321	1.5179400333
C	-2.8451612702	-3.5675797072	-1.1848310267
H	-3.4574369923	-4.4745827258	-1.0806800115
H	-2.7071666348	-3.3588596495	-2.2563715228
H	-1.8602948160	-3.7626052348	-0.7318570836

TS6Z_PrdZ
M06-2X/def2SVP Electronic E: -2018.556737 a.u.
M06-2X/def2SVP Gibbs free E: -2017.942785 a.u.
M06-2X/def2TZVPP Electronic E: -2020.231390 a.u.

C	2.3318803515	0.7187170171	-1.5594995668
C	0.9961989672	0.9124676857	-1.6170824362
C	-0.0351642494	0.2144076034	-2.4637781357
C	-0.9997711384	-0.6674889370	-1.6469835054
H	0.5967046033	1.7185801429	-0.9835823286
H	0.4840067551	-0.2799350313	-3.3114295272
H	-0.6282630338	1.0281577558	-2.9244906047
H	-1.8544538019	-0.9344467018	-2.3001330332
H	-0.4872193767	-1.6414826245	-1.4470950516
B	-2.9307129473	-0.4371875326	0.4500057428
O	-4.0559262797	-0.1803041963	-0.2991432276
O	-2.4708061159	0.6995297071	1.1137525315
C	-4.4833467310	1.1581821123	0.0032700985
C	-3.1784335131	1.8281633696	0.5772663784
C	-5.0148430573	1.8085864498	-1.2618432401
C	-5.5920462430	1.0455213810	1.0469153237
C	-2.3134772875	2.4674044878	-0.5038633508
C	-3.4339881592	2.8254910263	1.6958885745
H	-4.2795076465	1.7559595097	-2.0745313747
H	-5.9261739312	1.2867340833	-1.5878007113
H	-5.2698388352	2.8629376656	-1.0781883926
H	-6.0153190449	2.0280235496	1.2988486233
H	-6.3967065438	0.4154602243	0.6412597228
H	-5.2189737610	0.5757410842	1.9693054468
H	-2.7958967571	3.3605574458	-0.9258879927
H	-1.3585348143	2.7756161518	-0.0499712334
H	-2.0959452750	1.7414587775	-1.2983368522
H	-2.4782787596	3.2547194527	2.0307046223
H	-4.0702821276	3.6495162060	1.3401072608
H	-3.9180970219	2.3508987156	2.5586641741
O	-2.4926781240	-1.6489884217	0.9146570471
C	-3.1210630736	-2.9106074596	0.6494001041
C	-3.2801198764	-3.1402150315	-0.8507223930
H	-3.7329085836	-4.1271509604	-1.0272086283
H	-3.9277119539	-2.3716202227	-1.2938322774
H	-2.3036826368	-3.1043883579	-1.3531825955
C	-4.4781633914	-2.9349395288	1.3494480699
H	-4.9602099036	-3.9154772901	1.2244019821
H	-4.3540084207	-2.7410241656	2.4254888974
H	-5.1374165805	-2.1642638496	0.9252823966
C	-2.1881200547	-3.9566869213	1.2480167019
H	-2.5994841347	-4.9671517434	1.1132249630
H	-1.2048068325	-3.9148387358	0.7542090332
H	-2.0505434223	-3.7758108581	2.3245197817
K	-0.0484337790	-0.4809874936	0.9816798240
C	3.2128745262	1.6584098504	-0.7579876865
H	4.0346753337	2.0101658009	-1.4040026678
C	3.8832124034	1.1331711385	0.5406729440
C	2.8457522917	0.8455120087	1.6309694159
C	4.7961343126	2.2583696509	1.0637921771
C	4.7608796977	-0.0872531811	0.2524873761
C	2.8252478733	-0.3573395696	2.3528115807
C	1.9217042926	1.8403866359	2.0023359989
H	5.2669418339	1.9678542840	2.0149866615
H	5.5934161922	2.4671623721	0.3339330153
H	4.2337443961	3.1893478316	1.2295870784
H	5.4608232245	0.1444225138	-0.5636796372
H	5.3558875500	-0.3577515519	1.1378882423

H	4.1751947056	-0.9666048583	-0.0502034671
C	1.9345884398	-0.5523851619	3.4158692999
H	3.5186409871	-1.1597697487	2.1019271237
C	1.0305527569	1.6497996377	3.0583594615
H	1.9063962833	2.7924627241	1.4678730805
C	1.0355215372	0.4501274369	3.7774227418
H	1.9507324607	-1.4967547308	3.9634522197
H	0.3302440269	2.4437570380	3.3242503771
H	0.3425753576	0.3003318055	4.6069402095
H	2.6272438554	2.5551253794	-0.5083972721
C	3.0042073864	-0.3403218421	-2.3708586630
C	2.5399364235	-1.6654697981	-2.3584166507
C	4.1283746671	-0.0453628628	-3.1581526240
C	3.1755134673	-2.6589307628	-3.1015653068
H	1.6644784599	-1.9171432637	-1.7550566814
C	4.7643242087	-1.0367577005	-3.9049082764
H	4.5078467279	0.9776989469	-3.1986275292
C	4.2940775881	-2.3498225180	-3.8766499513
H	2.7950467157	-3.6817693926	-3.0723926958
H	5.6328509334	-0.7798552664	-4.5145640796
H	4.7951676264	-3.1271623870	-4.4562006560

KHMDS
M06-2X/def2SVP Electronic E: -1472.620350 a.u.
M06-2X/def2SVP Gibbs free E: -1472.442239 a.u.
M06-2X/def2TZVPP Electronic E: -1473.255260 a.u.

N	0.0015959222	0.1845771295	-0.1003298332
Si	1.5505353129	-0.4872000041	-0.0696027436
Si	-1.5483220355	-0.4828184042	-0.0376412420
C	1.9427827105	-1.6739979216	-1.5017775474
H	3.0027480320	-1.9786063329	-1.5062336595
H	1.3352505744	-2.5911473172	-1.4260760814
H	1.7185801474	-1.2085463372	-2.4756397478
C	1.9783130319	-1.4440847705	1.5173711304
H	3.0329486422	-1.7665948619	1.5313377533
H	1.8020220773	-0.8229140011	2.4111250156
H	1.3532556993	-2.3469706385	1.6147891363
C	2.8407238702	0.9144801101	-0.1813807685
H	3.8697511693	0.5191552343	-0.1823992941
H	2.7267381535	1.5014528216	-1.1102789599
H	2.7716804884	1.6014725387	0.6815436449
C	-2.0370457526	-1.5084853615	-1.5623071448
H	-1.9115810911	-0.9224751143	-2.4877214173
H	-1.4017494824	-2.4051495479	-1.6521005662
H	-3.0857708204	-1.8465171424	-1.5143444115
C	-1.8865528682	-1.6041837461	1.4600762925
H	-1.2978793005	-2.5341949920	1.3936248593
H	-1.6095074656	-1.1033881319	2.4023638243
H	-2.9496167372	-1.8903295746	1.5266436860
C	-2.8319150206	0.9260366580	0.0605311178
H	-2.7820854196	1.5842687056	-0.8258300538
H	-3.8614552079	0.5339253803	0.0995514256
H	-2.6947849518	1.5427945161	0.9668331850
K	0.0046863220	2.7450441058	-0.1109006000

2KHMDS
M06-2X/def2SVP Electronic E: -2945.292461 a.u.
M06-2X/def2SVP Gibbs free E: -2944.907297 a.u.
M06-2X/def2TZVPP Electronic E: -2946.556397 a.u.

N	1.9599214997	0.0011352060	0.0034162821
Si	2.6271418381	-1.5553499989	-0.0438497269
Si	2.6347730632	1.5543496951	0.0501858170
C	4.4662172197	-1.7089963506	0.3939779458
H	4.7637477958	-2.7696073961	0.4446929075
H	5.1070805264	-1.2187652854	-0.3557139718
H	4.6833267471	-1.2530532144	1.3740135916
C	2.4523080162	-2.3722496107	-1.7590670036
H	2.9497990456	-3.3560044154	-1.7827123548
H	1.4039107355	-2.5527424220	-2.0546812617
H	2.9251911937	-1.7468319746	-2.5355063453
C	1.7144773177	-2.7145260004	1.1620474082
H	2.0826232277	-3.7502750919	1.0794278021
H	1.8635744289	-2.4075998822	2.2128264419
H	0.6306482516	-2.7499320111	0.9548564618
C	2.4527261934	2.3769042213	1.7618658409
H	1.4032898931	2.5628743564	2.0502721937
H	2.9178671589	1.7514765571	2.5429587282
H	2.9545384430	3.3584456256	1.7860111437
C	4.4774892322	1.6968761354	-0.3757619195
H	5.1104572777	1.2043072693	0.3791046219
H	4.6985308421	1.2378958721	-1.3534938468
H	4.7814686398	2.7556589875	-0.4263806101
C	1.7366299376	2.7148270952	-1.1654750707
H	0.6516158372	2.7576676075	-0.9658117839
H	2.1104802922	3.7486217863	-1.0840471321
H	1.8910882872	2.4032395973	-2.2141196685
K	0.0061549953	0.0138734811	-1.8870893003
N	-1.9557124768	0.0106748533	-0.0053939370
Si	-2.6295577474	-1.5433476101	0.0237457900
Si	-2.6228024833	1.5674173884	-0.0424239490
C	-4.4698908499	-1.6836705822	-0.4130979027
H	-4.7729522810	-2.7421996456	-0.4736430119
H	-5.1071625746	-1.1974493869	0.3422674346
H	-4.6861177491	-1.2171985524	-1.3883543258
C	-2.4557517920	-2.3813755848	1.7286749534
H	-2.9535579975	-3.3651855160	1.7401484685
H	-1.4075678910	-2.5656126956	2.0226898085
H	-2.9288284476	-1.7652044960	2.5123390647
C	-1.7236527013	-2.6915982564	-1.1979355370
H	-2.0970520735	-3.7264344219	-1.1286933476
H	-1.8721732911	-2.3700370441	-2.2444613412
H	-0.6397968778	-2.7354414481	-0.9922554413
C	-2.4379637073	2.3997647729	-1.7491230055
H	-1.3877957719	2.5822671507	-2.0371444092
H	-2.9068622096	1.7817194246	-2.5338399169
H	-2.9347093153	3.3840221208	-1.7666341301
C	-4.4644016543	1.7169705410	0.3859289635
H	-5.1005350044	1.2328251426	-0.3717190695
H	-4.6872652006	1.2527501083	1.3607590228
H	-4.7626671562	2.7770131307	0.4437173778
C	-1.7168236823	2.7156059745	1.1790194263
H	-0.6320483575	2.7537864243	0.9772072144
H	-2.0853336060	3.7518505204	1.1047140992
H	-1.8705964695	2.3984215406	2.2260872191
K	-0.0022725680	-0.0009516923	1.8842302914

HMDSBpin
M06-2X/def2SVP Electronic E: -1283.603124 a.u.
M06-2X/def2SVP Gibbs free E: -1283.244393 a.u.
M06-2X/def2TZVPP Electronic E: -1284.626952 a.u.

B	0.4190355857	-0.6085461455	-0.1808623843
O	-0.3209079526	-0.5282488716	0.9837344739
O	-0.3250151058	-0.2136441294	-1.2766148646
C	-1.5330515844	0.1819990108	0.6800879327
C	-1.6936518642	-0.0973209408	-0.8532304353
C	-2.6597172234	-0.3552290379	1.5440135347
C	-1.2882436702	1.6580758028	0.9803491941
C	-2.3763274007	-1.4331592552	-1.1331666805
C	-2.3677653267	1.0166425976	-1.6336244692
H	-2.7426907276	-1.4462291969	1.4612309404
H	-2.4680899906	-0.1045396697	2.5973500548
H	-3.6192401078	0.0977692297	1.2533785655
H	-2.1974365872	2.2563920995	0.8271025540
H	-0.9766608731	1.7621934030	2.0295147717
H	-0.4901492348	2.0640016183	0.3407097329
H	-3.4471888121	-1.3973140033	-0.8880734577
H	-2.2722923954	-1.6703980115	-2.2016081271
H	-1.9111982600	-2.2436497527	-0.5521093596

H	-2.4463101931	0.7297132547	-2.6922022128
H	-3.3836073483	1.1963205361	-1.2513403563
H	-1.7954585379	1.9509204600	-1.5735680453
N	1.7764689158	-1.0433308703	-0.2438481035
Si	2.3824554090	-1.7580491216	-1.7531153386
Si	2.8087873523	-0.8530453184	1.1893422212
C	3.5140963600	-3.2074689074	-1.3442607325
C	3.3005843574	-0.4756809113	-2.7738229219
C	0.9738710746	-2.4662901483	-2.7739362907
C	2.8949779990	-2.4570026613	2.1635777932
C	4.5319691267	-0.3181010215	0.6479570482
C	2.1637147333	0.5131496680	2.3052156215
H	3.9049819026	-3.6281228049	-2.2851114419
H	4.3777111632	-2.9504079701	-0.7146990309
H	2.9441559042	-4.0023708353	-0.8373157966
H	3.6298211926	-0.9071201456	-3.7327280378
H	2.6379986021	0.3762623738	-2.9940872030
H	4.1886971275	-0.0903965549	-2.2510826986
H	1.3962905743	-3.0852883028	-3.5821984131
H	0.3233653987	-3.1106394521	-2.1614881436
H	0.3551162776	-1.6770324672	-3.2203997124
H	1.8825807840	-2.7718721909	2.4623456900
H	3.3463847883	-3.2718778817	1.5782002467
H	3.4933284343	-2.3228004756	3.0789930424
H	5.0299130466	-1.0114209004	-0.0445876893
H	4.4884922541	0.6731796411	0.1692457936
H	5.1716418242	-0.2273571673	1.5410005858
H	1.2420103032	0.2174025914	2.8230096940
H	2.9327302744	0.7504760397	3.0583277497
H	1.9589565501	1.4314834368	1.7323504367

TS1N
M06-2X/def2SVP Electronic E: -2232.621279 a.u.
M06-2X/def2SVP Gibbs free E: -2232.097294 a.u.
M06-2X/def2TZVPP Electronic E: -2234.104333 a.u.

B	0.4477751363	0.5172786427	-0.1057842925
C	-0.8590993270	-0.8994715140	-1.6878873301
O	-0.4577973566	0.7826696224	0.9873028583
O	0.5875451431	1.7096773393	-0.8307933476
C	-0.3701559201	2.1940791050	1.2397456380
C	-0.1129382322	2.7712929203	-0.1949469445
C	0.7596406914	4.0194272236	-0.2138264104
H	0.9044617599	4.3458428221	-1.2541604164
H	0.2786992410	4.8410589662	0.3386030010
H	1.7474022438	3.8303976824	0.2248662068
C	-1.4070606662	3.0473168224	-0.9518152669
H	-1.1691689093	3.2462751196	-2.0068522215
H	-2.0754973305	2.1779312342	-0.9074146753
H	-1.9337980530	3.9231849454	-0.5459650884
C	-1.6514762334	2.6905748004	1.8953253408
H	-1.5988466996	3.7772865723	2.0555168803
H	-2.5435622542	2.5027290259	1.2792822789
H	-1.7774159332	2.2264882611	2.8874401555
C	0.8048371170	2.4295232935	2.1875493279
H	1.7444682202	2.0658867997	1.7448537787
H	0.9188595406	3.4933632039	2.4378762457
H	0.6260074840	1.8741459840	3.1199211171
N	1.6105428810	-0.3599691378	0.0661903237
Si	1.5846572837	-1.5603031269	1.3614983428
C	2.4891132932	-3.1344077590	0.8479679203
H	3.5479225622	-2.9643770770	0.6023439982
H	2.4586231468	-3.8448359857	1.6904011676
H	2.0111914456	-3.6181834829	-0.0173687757
C	-0.1777814846	-2.0407849931	1.8448620588
H	-0.5159254435	-1.4019499687	2.6759710862
H	-0.8522539854	-1.9448988415	0.9835781220
H	-0.2051726500	-3.0869984160	2.1880885290
C	2.3930492420	-0.9425660754	2.9497180904
H	3.4684706972	-0.7438377402	2.8413432155
H	1.9074488760	-0.0208653672	3.3035403731
H	2.2696964647	-1.7086314739	3.7337049151
Si	3.1211854060	-0.1075879309	-0.8289084383
C	3.4618335322	-1.4361494344	-2.1282108316
H	2.8227741717	-1.3037513767	-3.0149201876
H	4.5089069976	-1.3366705897	-2.4597517658
H	3.3225461410	-2.4612833610	-1.7561230215
C	4.5778292938	-0.1224443228	0.3778919693
H	4.4683369223	0.6715342418	1.1340274593
H	4.7212528843	-1.0777482378	0.9038372889
H	5.5011219304	0.0827733720	-0.1883080540
C	3.2498495919	1.5340856730	-1.7329318761
H	4.2413778315	1.5671014582	-2.2152201482
H	2.4761250413	1.6494838718	-2.5032375512
H	3.1654450384	2.3875952990	-1.0456586898
C	-2.2279253583	-1.0511682500	-1.3097191559
C	-2.7368840889	-2.2440972019	-0.6791520119
C	-3.2327430028	-0.0474471927	-1.5433734536
C	-4.0472242622	-2.3537173105	-0.2243521808
H	-2.0797449504	-3.1049129185	-0.5569341663
C	-4.5346815467	-0.1638756386	-1.0716694730
H	-2.9728704974	0.8346874656	-2.1273536261
C	-4.9665074177	-1.3035342688	-0.3712331104
H	-4.3631429214	-3.2869980956	0.2502936704
H	-5.2413023228	0.6464432576	-1.2717360833
H	-5.9939070576	-1.3954935025	-0.0177956120
C	-0.0642255441	-2.1858713393	-1.7578155270
H	0.9825494719	-1.9815356059	-2.0128629089
H	-0.0469647650	-2.7463728639	-0.8105898944
H	-0.4605840753	-2.8852708598	-2.5258023712
K	-2.7792721683	-0.2339392911	1.5146954872
C	-0.5683328954	0.0090759386	-2.8661705444
H	-0.9593815918	-0.4050996075	-3.8209528998
H	-0.9793666070	1.0202410915	-2.7499846922
H	0.5188828279	0.1424601039	-2.9929888021

Int2N
M06-2X/def2SVP Electronic E: -2232.639403 a.u.
M06-2X/def2SVP Gibbs free E: -2232.119547 a.u.
M06-2X/def2TZVPP Electronic E: -2234.124501 a.u.

B	0.9830372613	0.9074577585	0.2841973945
C	-1.0598268889	-1.5905737053	-1.9599976416
O	-0.1246205278	0.9502376702	1.1489372126
O	1.2361263366	2.1596785317	-0.2277906229
C	-0.3491972632	2.3470715144	1.4677323134
C	0.1991111583	3.0590308873	0.1880954479
C	0.8038226537	4.4284619512	0.4440181331
H	1.1445547822	4.8605872802	-0.5076718287
H	0.0552841754	5.1067793011	0.8791873525
H	1.6653648245	4.3666389444	1.1206387457
C	-0.8338065608	3.1378400324	-0.9311828999
H	-0.3204312968	3.4073719754	-1.8651713033
H	-1.3371304663	2.1714351524	-1.0855389310
H	-1.5967088867	3.9004723847	-0.7200543029
C	-1.8234870072	2.6003631290	1.7360089190
H	-1.9861421110	3.6713959252	1.9235190484
H	-2.4638586568	2.3238501761	0.8852853442
H	-2.1464474741	2.0661975716	2.6443558280
C	0.4685147349	2.6628211711	2.7150056849
H	1.5426290238	2.5074425331	2.5338549059
H	0.3153267526	3.7010421944	3.0394332858
H	0.1537048843	1.9958158098	3.5306768886
N	1.7835490534	-0.2481246938	0.0553735606
Si	1.7523986360	-1.4802842032	1.3443101051
C	2.8344821078	-2.9618875209	0.9435849146
H	3.8761522493	-2.7089275934	0.6998512450
H	2.8505153289	-3.5884663907	1.8507459759
H	2.4177667771	-3.5727662284	0.1291298185
C	0.0124116393	-2.1427726683	1.6430515812

H	-0.4779815986	-1.5688085788	2.4445692775
H	-0.5714873288	-2.1200784180	0.7089137510
H	0.0654316646	-3.1903249593	1.9779583688
C	2.3877861783	-0.7016328905	2.9322013020
H	3.4142095535	-0.3227025351	2.8064589329
H	1.7469985009	0.1401995014	3.2351363077
H	2.3930334857	-1.4372367834	3.7525007423
Si	3.1506235844	-0.1475225816	-1.0961331256
C	3.3491826714	-1.7045589896	-2.1355098130
H	2.5222993642	-1.8265814775	-2.8508493357
H	4.2739409288	-1.5800355183	-2.7239295298
H	3.4464770536	-2.6343920169	-1.5619273454
C	4.7251157234	0.1377717527	-0.1018381536
H	4.6327078221	1.0599699321	0.4943907295
H	4.9544140935	-0.6907346712	0.5858189833
H	5.5875000336	0.2589855697	-0.7771052199
C	2.9740828745	1.2656696546	-2.3193808791
H	3.7263400459	1.1169544574	-3.1115830915
H	1.9804447997	1.2880359206	-2.7897675696
H	3.1466182232	2.2407934405	-1.8463303618
C	-2.3739485110	-1.4630714398	-1.5550280267
C	-3.1428191746	-2.4936034906	-0.8544842643
C	-3.1294247283	-0.2241063010	-1.7634936678
C	-4.3883285840	-2.2427923579	-0.3087657829
H	-2.7221830058	-3.4950833759	-0.7487568502
C	-4.3772962175	-0.0119488558	-1.2024928891
H	-2.6908397718	0.5638370716	-2.3781921419
C	-5.0263425485	-0.9848300846	-0.4129311934
H	-4.8958888750	-3.0563565039	0.2193854282
H	-4.8728627942	0.9473636068	-1.3846126205
H	-6.0181828114	-0.8127727413	0.0044329474
C	-0.2483558644	-2.8476175456	-1.7987411077
H	0.7500186482	-2.6216179733	-1.3789012999
H	-0.7173089034	-3.5845264823	-1.1308981075
H	-0.0589604816	-3.3648966572	-2.7608721200
K	-2.4520814948	-0.3863324345	1.1133121476
C	-0.3937241337	-0.4678742887	-2.7158944907
H	-0.8822675209	-0.2397405306	-3.6840899282
H	-0.3756511226	0.4881671179	-2.1579375209
H	0.6523049813	-0.7193934325	-2.9428156562

TS4_5N

M06-2X/def2SVP Electronic E: -2541.948766 a.u.
M06-2X/def2SVP Gibbs free E: -2541.296169 a.u.
M06-2X/def2TZVPP Electronic E: -2543.770148 a.u.

C	-0.4155348195	4.0058623306	-2.2354740073
C	-0.6619291430	4.9165545052	-1.1954217112
C	-0.2952618235	4.5211527239	0.1013892632
C	0.2224046633	3.2566267616	0.3538894220
C	0.4055448147	2.2621728307	-0.6675819111
C	0.1077783779	2.7406444541	-1.9919598584
H	-0.6316097713	4.2939086282	-3.2680273360
H	-1.0608048484	5.9114839727	-1.3949781557
H	-0.4087481601	5.2203694292	0.9346176778
H	0.5235906698	3.0188208574	1.3732766664
H	0.2919559047	2.0912734247	-2.8482263993
C	0.8035139673	0.9254958331	-0.3651705064
H	1.2308730238	0.8039624097	0.6346927635
C	1.4260396126	0.0587744156	-1.4303817193
H	1.2967415726	-1.0037435276	-1.1750196771
H	0.8847571587	0.1819582003	-2.3866023987
C	4.9777178453	-1.9076718009	0.7430321641
C	4.3072570619	-1.4970742116	-0.4110498876
C	3.7528587931	-0.2128905110	-0.5194018842
C	3.9102867565	0.6507071035	0.5784676598
C	4.5741954445	0.2432738782	1.7354820285
C	5.1117335632	-1.0412098784	1.8256855135
H	5.3953766726	-2.9154441455	0.7928050257
H	4.2125194133	-2.2052143018	-1.2347866635
H	3.5013933178	1.6607375149	0.5349157044
H	4.6744640371	0.9385009070	2.5716680381
H	5.6334254034	-1.3606896174	2.7297164553
C	2.9521166557	0.2212563332	-1.7484501026
K	-2.5994988519	2.6096973347	-0.5217304827
C	3.3005287171	1.6640267221	-2.1348292191
H	4.3814031799	1.7630309021	-2.3256270105
H	2.7665221131	1.9486343615	-3.0542932772
H	3.0203527668	2.3881885293	-1.3585977626
C	3.2561316578	-0.6754771123	-2.9544802242
H	2.7389805644	-0.2832167662	-3.8432875118
H	4.3342029693	-0.7115593040	-3.1769640539
H	2.9021760947	-1.7058641482	-2.7992468314
B	-0.8999499278	-0.3954148603	0.6337290710
O	-0.2959201112	-0.8103312799	1.8350963467
O	-1.8551401282	0.6257198516	0.9537013920
C	-0.7803987606	-0.0673897737	2.9410669437
C	-2.0918869436	0.5957019504	2.3717321662
C	-1.0268977292	-1.0298285066	4.0985363839
C	0.2855916637	0.9372942067	3.3689243094
C	-3.3307748299	-0.2604353359	2.6266796201
C	-2.3380008422	2.0126701918	2.8690126986
H	-1.6735548562	-1.8647419494	3.8009622078
H	-0.0641923367	-1.4452753811	4.4300507791
H	-1.4861261166	-0.5109989503	4.9534627616
H	-0.0055423227	1.4796877956	4.2798757516
H	1.2177044146	0.3891355557	3.5695211228
H	0.4890920874	1.6614555962	2.5754429931
H	-3.5585159355	-0.3279940250	3.6995607058
H	-4.1985417893	0.1905652462	2.1244530549
H	-3.1931561397	-1.2766133669	2.2292319215
H	-3.2941849177	2.3945445311	2.4734665910
H	-2.4179796995	2.0296571887	3.9659976459
H	-1.5279466461	2.6915428956	2.5693339582
N	-1.1606859313	-1.4342618476	-0.3792503310
Si	-0.3057345165	-2.9824924938	-0.2368948335
Si	-2.4931149627	-1.2189173687	-1.5035655198
C	0.2561517568	-3.5913494657	-1.9444628954
C	-1.4498736867	-4.2781743453	0.5212253051
C	1.2301843441	-2.9245082119	0.8535649039
C	-2.1554083939	0.2551844227	-2.6363720092
C	-2.7625645614	-2.7028525087	-2.6317646906
C	-4.1543365611	-0.9362184515	-0.6540272612
H	-0.4492786982	-4.3115037320	-2.3848126448
H	0.3839931040	-2.7611163494	-2.6564954607
H	1.2287332768	-4.1004625062	-1.8482761263
H	-2.3717538681	-4.4253749428	-0.0621716136
H	-0.9381602420	-5.2519553837	0.5948989543
H	-1.7390912596	-3.9730810783	1.5399057227
H	1.8803841798	-3.7756430105	0.5909842220
H	1.8078279393	-1.9961035623	0.7417220324
H	0.9513856054	-3.0055681891	1.9127468456
H	-3.0777560334	0.7600346906	-2.9709007117
H	-1.4811999411	0.9729520044	-2.1515398423
H	-1.6251185152	-0.0804547429	-3.5421839524
H	-1.9433244583	-2.8429662286	-3.3500910285
H	-2.9225675506	-3.6492615486	-2.0941201385
H	-3.6776846277	-2.4961149723	-3.2114505375
H	-4.1862442730	-0.0063136535	-0.0699763342
H	-4.9466320267	-0.8942203555	-1.4194297907
H	-4.3949196069	-1.7633667878	0.0327535205

Int5N

M06-2X/def2SVP Electronic E: -2541.979096 a.u.
M06-2X/def2SVP Gibbs free E: -2541.323584 a.u.
M06-2X/def2TZVPP Electronic E: -2543.800095 a.u.

C	-0.7532880223	-3.4983552630	-2.1258920454
C	-1.4613154171	-4.3555408848	-1.2757088588
C	-1.9935947660	-3.8414687252	-0.0886445935

C	-1.8056873331	-2.4969266353	0.2443905916
C	-1.0857234674	-1.6184206585	-0.5905983205
C	-0.5731850615	-2.1557833039	-1.7868738281
H	-0.3402862768	-3.8794771043	-3.0625283447
H	-1.6106105184	-5.4029245409	-1.5431273590
H	-2.5657637292	-4.4898224643	0.5786461355
H	-2.2454057415	-2.1047085102	1.1651864954
H	-0.0265918751	-1.5016579853	-2.4698391838
C	-0.8722825927	-0.1880678227	-0.2070410024
H	-1.4970178247	-0.0077643989	0.6807284820
C	-1.3115854295	0.8057668872	-1.2855706255
H	-0.9809444298	1.7998956644	-0.9607705794
H	-0.7680586746	0.6186173976	-2.2292267647
C	-3.9815203486	2.2885068367	1.7690200840
C	-3.3139530216	2.1305343662	0.5573628769
C	-3.6158468829	1.0685625340	-0.3128182745
C	-4.6165187072	0.1725042089	0.0811133911
C	-5.2900737946	0.3269454636	1.2973848652
C	-4.9762965031	1.3831385695	2.1479095377
H	-3.7205518863	3.1218334151	2.4246262519
H	-2.5299694194	2.8422819435	0.2883879289
H	-4.8826393045	-0.6691004346	-0.5589407476
H	-6.0659788084	-0.3888254235	1.5768719749
H	-5.5000614909	1.5034483327	3.0979684082
C	-2.8268060904	0.9162926926	-1.6152256666
C	-3.3159405043	-0.2756846369	-2.4455984426
H	-4.3589284372	-0.1261912551	-2.7672104946
H	-2.6965456696	-0.3716297072	-3.3507503807
H	-3.2630850736	-1.2287243645	-1.9036961188
C	-3.0225277281	2.1799530573	-2.4712328646
H	-2.4318886295	2.1023322362	-3.3980258446
H	-4.0800515195	2.3119903775	-2.7483851028
H	-2.6932022869	3.0846913944	-1.9390063768
B	0.6821244449	0.1544262602	0.3847547920
O	0.5793464662	1.0767616108	1.5472533916
O	1.2325862949	-1.0983522469	1.0121557716
C	0.8009372501	0.3764182981	2.7465972845
C	1.7524382648	-0.7772889519	2.2917718958
C	1.4069963092	1.3124179671	3.7847788686
C	-0.5269819648	-0.1735448896	3.2830570401
C	3.2008236350	-0.3030509949	2.1527206535
C	1.7094036666	-2.0090995214	3.1923347296
H	2.3064522330	1.8086830673	3.3989892368
H	0.6752748608	2.0908834556	4.0492251065
H	1.6723634423	0.7682582748	4.7047419619
H	-0.4228232548	-0.5958199705	4.2941167160
H	-1.2569962431	0.6487612175	3.3225156442
H	-0.9261333302	-0.9509182911	2.6167708823
H	3.6681336196	-0.1134397914	3.1306665704
H	3.7874735698	-1.0821159227	1.6410752404
H	3.2408232990	0.6120667300	1.5479240398
H	2.4618698533	-2.7514941893	2.8727361546
H	1.9587670943	-1.7436238532	4.2306997885
H	0.7124026819	-2.4761436981	3.1998077765
N	1.6379513881	0.8203118892	-0.6668876033
Si	2.7490820200	-0.0384686817	-1.6960482472
Si	1.8451679476	2.5631473511	-0.5545550132
C	4.5376096896	0.5634052485	-1.4994581955
C	2.3195034769	0.0907082640	-3.5382942972
C	2.9063509847	-1.9013676456	-1.3827996043
C	3.2169708886	3.0885774857	0.6423930313
C	2.2979320097	3.2923640978	-2.2509458797
C	0.3422859948	3.6042582467	-0.0625500129
H	5.1843449885	0.0202066904	-2.2083603670
H	4.6744926944	1.6384925377	-1.6846557903
H	4.9039018246	0.3482187500	-0.4822117560
H	2.9954789907	-0.5469357380	-4.1319681280
H	1.2873064717	-0.2382645648	-3.7401489082
H	2.4148838247	1.1189901469	-3.9155388133
H	3.6949606546	-2.2822345113	-2.0518011693
H	3.2045377418	-2.1042210842	-0.3444699919
H	1.9796420106	-2.4323372727	-1.6430970571
H	2.8838140163	2.9399012685	1.6803765291
H	4.1536128800	2.5302876799	0.4970402459
H	3.4383946990	4.1605695667	0.5094283193
H	3.2444720194	2.9374555208	-2.6838636928
H	1.4943192309	3.0925964423	-2.9788425499
H	2.3756343733	4.3871423360	-2.1412193342
H	-0.2070998358	3.1492195906	0.7712343906
H	0.7207202643	4.5878690592	0.2640233785
H	-0.3409651021	3.7841780119	-0.9070030187
K	1.0426049267	-3.5813675043	0.4849807866

3ClPhMe2CBpin-KOtBu

M06-2X/def2SVP Electronic E: -2052.162755 a.u.

M06-2X/def2SVP Gibbs free E: -2051.742845 a.u.

M06-2X/def2TZVPP Electronic E: -2053.518396 a.u.

C	-3.9996922034	0.2423889511	-0.0550452422
C	-2.8793963181	0.4098447371	0.7600385091
C	-1.9475347091	-0.6280753883	0.9653693652
C	-2.2285254600	-1.8634481281	0.3458451249
C	-3.3447009539	-2.0121483944	-0.4728973513
C	-4.2462351825	-0.9723369666	-0.6986128451
H	-4.6944016546	1.0728799902	-0.1962679352
H	-2.7172692180	1.3793789428	1.2276545036
H	-1.5479110933	-2.7049688985	0.4599381948
H	-5.1139240522	-1.1163908284	-1.3424811298
C	-0.6493972036	-0.4088352310	1.6965560892
B	0.4756396631	0.0243460400	0.5148678081
O	1.8350526106	0.1077458312	1.0097758302
O	0.5136915342	-1.0809566810	-0.4836694200
C	1.8444264441	-1.2913690885	-0.9082751440
C	2.7127719692	-0.7370937351	0.2969761513
C	2.0697020171	-0.5233657297	-2.2170459876
H	3.0930794764	-0.6366220248	-2.6025211211
H	1.3895328834	-0.9240177901	-2.9881251211
H	1.8604173441	0.5452853428	-2.0832296750
C	2.0463849885	-2.7787428471	-1.1835925493
H	1.7782952200	-3.3878095150	-0.3108220349
H	1.4023494175	-3.0868530475	-2.0215666796
H	3.0893489978	-2.9973220481	-1.4593004330
C	3.9297092243	0.0725725783	-0.1504547233
H	4.6276152735	-0.5385212575	-0.7435643382
H	3.6330653714	0.9456757081	-0.7474864795
H	4.4666171794	0.4370748644	0.7383643102
C	3.1944691313	-1.8423763073	1.2414439007
H	2.3610428145	-2.4720863274	1.5765130707
H	3.9558970768	-2.4844901122	0.7746465703
H	3.6385896128	-1.3681169816	2.1294388821
O	0.0079235165	1.2570810370	-0.2060567570
C	0.3786182000	2.6192403503	-0.1242120000
K	-1.3373498669	-0.0262236679	-1.9870309598
C	1.0916460946	3.0142264279	-1.4242419472
H	0.4831783128	2.7385413122	-2.3016392567
H	1.2741059146	4.0987491168	-1.4647212881
H	2.0637055517	2.5056295035	-1.5006413022
C	-0.9126544819	3.4380522659	-0.0072290744
H	-0.7079239449	4.5180947803	-0.0564038016
H	-1.6040779796	3.1817640474	-0.8270419749
H	-1.4200287507	3.2295067375	0.9462170855
C	1.2983364983	2.9607446526	1.0517221680
H	0.8446727299	2.6698304014	2.0080432243
H	2.2584601995	2.4396351035	0.9659006090
H	1.4732398752	4.0475554912	1.0696550427
C	-0.1633402292	-1.6905075002	2.3785075017
H	0.1065705992	-2.4666918868	1.6484316386
H	0.7427297074	-1.4705029626	2.9612987693
H	-0.9198518237	-2.1091046118	3.0662254125

C	-0.7675944323	0.6875843855	2.7559476360
H	0.1988185680	0.8087347721	3.2688111815
H	-1.0279826752	1.6636171798	2.3257047913
H	-1.5271681256	0.4430342525	3.5206544977
Cl	-3.6003971894	-3.5445760959	-1.2791693261

4MeOstyrene-3ClPhMe2CK-add-ts
M06-2X/def2SVP Electronic E: -1832.149137 a.u.
M06-2X/def2SVP Gibbs free E: -1831.870426 a.u.
M06-2X/def2TZVPP Electronic E: -1833.244571 a.u.

C	2.9923653468	-0.5382261242	-1.3932789056
C	3.9569648778	-0.8646885636	-0.4227495815
C	4.0047197213	-0.0988581006	0.7504289487
C	3.1028344042	0.9532416636	0.9401984559
C	2.1102764614	1.2937152646	-0.0083892507
C	2.0998922957	0.5065948462	-1.1942199096
H	2.9612298968	-1.1298282264	-2.3105032573
H	4.7433301903	-0.3070849322	1.5248911328
H	3.1614167816	1.5296694163	1.8670483963
H	1.3602865864	0.7082915773	-1.9713726778
C	1.1744183477	2.3715350500	0.2410287237
H	1.3139661572	2.9186499345	1.1782263679
C	0.1565542617	2.7438670277	-0.6147042804
H	-0.2838949567	3.7363788936	-0.4954008270
H	0.1529651207	2.3696532870	-1.6425605979
C	-1.7353054258	-0.8338392958	2.1571993357
C	-1.7035650701	0.4502827901	1.6232950128
C	-1.8160063519	0.6942285665	0.2106843705
C	-1.7924582404	-0.4861759666	-0.6087640313
C	-1.7964355723	-1.7474895695	-0.0422720603
C	-1.7996737392	-1.9749152996	1.3440938525
H	-1.7120489980	-0.9579750876	3.2430956507
H	-1.6791542821	1.2951355108	2.3108190193
H	-1.8169205765	-0.4067660696	-1.6954807907
C	-1.9149014297	2.0076141711	-0.3160972481
K	1.0053517772	-1.2634363295	0.9243021473
C	-2.3802419236	2.1936723956	-1.7389077240
H	-3.4670927828	2.0231424542	-1.8636757812
H	-2.1773659325	3.2244924270	-2.0746102134
H	-1.8651981378	1.5267797416	-2.4477906813
C	-2.3523253672	3.0920880466	0.6436758279
H	-2.4405363322	4.0564131282	0.1190936108
H	-3.3344186912	2.8825508209	1.1093182487
H	-1.6321785127	3.2428859315	1.4661771336
H	-1.8489339300	-2.9836337829	1.7523832665
Cl	-1.7433978853	-3.1460806221	-1.1059197300
O	4.7783475810	-1.9050355128	-0.7005829553
C	5.7611779810	-2.2373983334	0.2483452426
H	5.3126141807	-2.5341103306	1.2117117947
H	6.3206731995	-3.0873576863	-0.1592841484
H	6.4573902391	-1.4001719717	0.4217070531

4MeOstyrene
M06-2X/def2SVP Electronic E: -423.678041 a.u.
M06-2X/def2SVP Gibbs free E: -423.545059 a.u.
M06-2X/def2TZVPP Electronic E: -424.156999 a.u.

C	1.4404097639	0.2448959000	0.0002925663
C	0.9572467347	-1.0782082420	0.0005934146
C	0.4983302961	1.2783278498	-0.0001505318
C	-0.4008194285	-1.3448647895	0.0003702090
H	1.6588998158	-1.9145056626	0.0011374931
C	-0.8756633332	1.0284755139	-0.0004139047
H	0.8461549123	2.3141867569	-0.0003130305
C	-1.3340530284	-0.2927121838	-0.0001548268
H	-0.7761111320	-2.3693114650	0.0006835575
H	-1.5722604577	1.8657546893	-0.0008127687
C	2.8771083469	0.5745279464	0.0004443559
C	3.8988014517	-0.2884713675	-0.0012901475
H	4.9298176894	0.0707911765	-0.0009437041
H	3.7512449478	-1.3716737407	-0.0031158920
H	3.0990288440	1.6471769007	0.0020612428
O	-2.6340051063	-0.6453993505	-0.0003045275
C	-3.6033237354	0.3769722703	-0.0002401302
H	-3.5209013945	1.0109128413	-0.8979639676
H	-4.5814071209	-0.1174293053	-0.0001103306
H	-3.5206580656	1.0109762619	0.8974229227

4MeOstyrene-PhMe2CK-add-ts
M06-2X/def2SVP Electronic E: -1372.697054 a.u.
M06-2X/def2SVP Gibbs free E: -1372.405964 a.u.
M06-2X/def2TZVPP Electronic E: -1373.637435 a.u.

C	2.4653506977	-0.5489677004	1.0944310308
C	3.4027937900	-0.3030896556	0.0744648757
C	3.0986639728	-0.7279307310	-1.2259044096
C	1.8819434570	-1.3661509163	-1.4891845361
C	0.9107316904	-1.6020081740	-0.4908624494
C	1.2602085038	-1.1808994538	0.8213529189
H	2.7091718568	-0.2277639270	2.1089808304
H	3.8015933160	-0.5708501993	-2.0441545525
H	1.6676612364	-1.6789898934	-2.5143967681
H	0.5572187413	-1.3312111271	1.6427918647
C	-0.3667615419	-2.2108998132	-0.8169081912
H	-0.5005458532	-2.5093315069	-1.8611044079
C	-1.3841548802	-2.4299741430	0.0826540818
H	-2.1847523813	-3.1211437838	-0.1880375460
H	-1.1958216979	-2.3426144963	1.1560745598
C	-2.1065673598	2.3206088335	-1.3249992715
C	-2.5036358609	0.9939676666	-1.1632573099
C	-2.5699517415	0.3642003405	0.1297974489
C	-2.0601879595	1.1684785478	1.2096043237
C	-1.6476385808	2.4803503837	1.0290767542
C	-1.6774914348	3.0966177745	-0.2393219366
H	-2.1357858332	2.7600841188	-2.3263270057
H	-2.8566675327	0.4509480221	-2.0402719145
H	-2.0440983868	0.7567695346	2.2200739405
H	-1.3077995983	3.0506553667	1.8978853479
H	-1.3930035248	4.1418500623	-0.3664614372
C	-3.1027934052	-0.9382118764	0.3013201604
K	0.6008939710	1.2916859476	-0.7124918838
C	-3.5061442837	-1.3964486230	1.6798809801
H	-4.4445533424	-0.9291719589	2.0390823266
H	-3.6711822165	-2.4872835923	1.6888745567
H	-2.7368971816	-1.1920022156	2.4413322507
C	-3.9301956486	-1.4934578056	-0.8338893150
H	-4.3321886898	-2.4851208090	-0.5723183996
H	-4.7942537255	-0.8514691583	-1.0963058299
H	-3.3403067650	-1.6235620757	-1.7590483746
O	4.5447135656	0.3286491609	0.4327035039
C	5.5025671195	0.5747228776	-0.5673418680
H	5.1006266894	1.2237395821	-1.3636366968
H	6.3422462559	1.0858520216	-0.0823937692
H	5.8662520823	-0.3623205551	-1.0202108425

4MeOstyrene-PhMeCHK-add-ts
M06-2X/def2SVP Electronic E: -1333.432402 a.u.
M06-2X/def2SVP Gibbs free E: -1333.167712 a.u.
M06-2X/def2TZVPP Electronic E: -1334.328423 a.u.

C	-2.9706380225	-0.2920420704	1.2411404442
C	-3.7568876111	-0.6967822635	0.1468718577
C	-3.5175776052	-0.1030410716	-1.0999753171
C	-2.5080771470	0.8549116911	-1.2391580880
C	-1.6846457369	1.2618390126	-0.1645137813
C	-1.9708850057	0.6590725476	1.0916391884
H	-3.1689382807	-0.7434453771	2.2152894429
H	-4.1119228397	-0.3757965769	-1.9722550566
H	-2.3376481238	1.2938048867	-2.2256183399
H	-1.3828374898	0.9332047227	1.9693048377
C	-0.6153418324	2.2215337418	-0.3631326928

H	-0.5551478902	2.6671222709	-1.3608374145
C	0.3094651860	2.5872574863	0.5918177105
H	0.8732898209	3.5103585904	0.4476854896
H	0.1545989031	2.3056339962	1.6362448567
C	2.2660044721	-1.7302774573	-1.1990308419
C	2.2980260025	-0.3491477435	-0.9966487550
C	2.2065979858	0.2255517583	0.3165447052
C	1.9318191428	-0.7108228522	1.3719469738
C	1.8826923014	-2.0775477692	1.1533676012
C	2.0650759714	-2.6218194905	-0.1380674239
H	2.4011485350	-2.1185323556	-2.2127392581
H	2.4877005656	0.3019603388	-1.8515832974
H	1.8104646699	-0.3259322372	2.3887860455
H	1.7099707255	-2.7460125442	2.0010961825
H	2.0627689362	-3.7006104308	-0.2988353504
C	2.3467839692	1.6115557548	0.5458781603
K	-0.6107364824	-1.4388367460	-0.5382342763
C	2.9402097446	2.4776317785	-0.5314442906
H	3.0996176348	3.5055829681	-0.1725506848
H	3.9135739801	2.1078849924	-0.9070696618
H	2.2717041279	2.5471071554	-1.4114456299
H	2.5445593208	1.9015628876	1.5817506557
O	-4.7034523727	-1.6346013845	0.3867039715
C	-5.5129749308	-2.0416498273	-0.6885671714
H	-6.2127004258	-2.7874151777	-0.2938566974
H	-6.0864759345	-1.1980611337	-1.1069768021
H	-4.9163920442	-2.5022431510	-1.4941073826

octene
M06-2X/def2SVP Electronic E: -314.069065 a.u.
M06-2X/def2SVP Gibbs free E: -313.883194 a.u.
M06-2X/def2TZVPP Electronic E: -314.431319 a.u.

C	-1.8118102100	-1.5817773249	0.0931036680
C	-0.9765626192	-2.4275950348	-0.8715744897
C	0.3448181194	-2.8351747782	-0.2899265719
H	0.3000259932	-3.4152975876	0.6407039387
C	1.5369452239	-2.5339989396	-0.8055107669
H	2.4648957195	-2.8559750341	-0.3265132465
H	1.6252777089	-1.9543410270	-1.7300233178
H	-1.5527487443	-3.3335744247	-1.1300810420
H	-0.8134334850	-1.8739599684	-1.8105878613
H	-1.9549323242	-2.1390289414	1.0351916007
H	-1.2478526884	-0.6707548557	0.3564828484
C	-3.1720216031	-1.1937594394	-0.4769589789
H	-3.7269764951	-2.1091338575	-0.7478528839
H	-3.0265910541	-0.6345471710	-1.4178327323
C	-4.0144052006	-0.3582334878	0.4812854084
H	-3.4575897938	0.5551295305	0.7557733892
H	-4.1620958786	-0.9193745028	1.4209939244
C	-5.3742643996	0.0343629838	-0.0886339679
H	-5.2237175292	0.5981292709	-1.0248003365
H	-5.9256405411	-0.8794379465	-0.3677554374
C	-6.2095095233	0.8618964384	0.8802996617
H	-5.6909376057	1.7944213197	1.1520430409
H	-7.1830646435	1.1341759035	0.4473172480
H	-6.4005294260	0.3058548747	1.8112689047

octene-PhMe2CK-add-int
M06-2X/def2SVP Electronic E: -1263.098661 a.u.
M06-2X/def2SVP Gibbs free E: -1262.748919 a.u.
M06-2X/def2TZVPP Electronic E: -1263.920393 a.u.

C	-2.4737673653	-0.5726028839	-1.9553109701
C	-1.4240277807	-1.5615936057	-2.4917990711
C	0.0306319858	-1.3266586062	-2.0653394357
H	0.6635747324	-1.6873124825	-2.9000132248
C	0.3749541075	-2.1961903239	-0.8557680338
H	-0.3652480433	-2.0397380425	-0.0492391110
H	0.2982148571	-3.2926655028	-1.0741249546
C	1.0070928160	1.1017359127	1.8978153965
C	0.9769348186	-0.1872841244	1.3646204529
C	1.8837328819	-0.5955317114	0.3683615131
C	2.8460698720	0.3376482948	-0.0453640654
C	2.8839167507	1.6319674750	0.4868283538
C	1.9587708430	2.0264017103	1.4538145918
H	0.2860185460	1.3866494118	2.6667352526
H	0.2195255046	-0.8883663439	1.7221934138
H	3.5761098948	0.0664623855	-0.8079237653
H	3.6422465791	2.3352839898	0.1368472951
H	1.9858944547	3.0355071075	1.8683827072
C	1.7770401466	-2.0035895180	-0.2195064367
C	2.8556408591	-2.2632471062	-1.2717954426
H	3.8680782242	-2.2089004054	-0.8410200853
H	2.7204262034	-3.2735938742	-1.6881418468
H	2.7926134272	-1.5472446158	-2.1032703544
C	1.9616601333	-3.0216144376	0.9188544213
H	1.9227445958	-4.0460370373	0.5176397051
H	2.9326717475	-2.8884481760	1.4216295744
H	1.1689925176	-2.9301549802	1.6769365394
H	-1.7895885347	-2.5853412068	-2.2225104782
H	-1.4974642556	-1.5175343434	-3.5935053782
H	-2.2551980485	0.4353767624	-2.3626318743
H	-3.4736736753	-0.8308576683	-2.3499809502
C	-2.5707435870	-0.4891452329	-0.4344672839
H	-1.5724315909	-0.2911566478	-0.0020831970
H	-2.8592327111	-1.4787459501	-0.0368522132
C	-3.5529634289	0.5650083868	0.0674041977
H	-4.5553047179	0.3609197260	-0.3483231119
H	-3.2636767272	1.5576925491	-0.3272536249
C	-3.6406326380	0.6395364607	1.5888625423
H	-3.9400721271	-0.3472359848	1.9799876717
H	-2.6343763863	0.8364495736	1.9980907572
C	-4.6126716307	1.7042735600	2.0813055297
H	-5.6307151572	1.5104964281	1.7096375162
H	-4.6579438340	1.7366468257	3.1795951622
H	-4.3163941592	2.7044749836	1.7283868503
K	0.1385409000	1.4207262684	-1.2127735347

octene-PhMe2CK-add-ts
M06-2X/def2SVP Electronic E: -1263.063898 a.u.
M06-2X/def2SVP Gibbs free E: -1262.719139 a.u.
M06-2X/def2TZVPP Electronic E: -1263.887928 a.u.

C	-2.0278583127	-1.2073207169	0.2411043944
C	-1.2458321402	-2.2190776961	-0.6211312620
C	0.0813999216	-2.7162454390	-0.1151022151
H	0.0250225809	-3.5120018561	0.6360923320
C	1.3376290275	-2.4732816977	-0.7014679894
H	2.0473591980	-3.3159231811	-0.7032169699
H	1.3411886456	-1.9745830572	-1.6910117556
C	0.8816060450	1.8548699129	0.9370243579
C	1.3550403977	0.5457523251	1.0430456683
C	2.2891632745	0.0064683413	0.1070749544
C	2.7012059505	0.8887982156	-0.9380270954
C	2.2290902094	2.1952724963	-1.0293378450
C	1.3042942723	2.6981697582	-0.0977624474
H	0.1730326593	2.2253753219	1.6823299866
H	1.0208466032	-0.0658655998	1.8806032488
H	3.4301872454	0.5470123974	-1.6740104890
H	2.5925991929	2.8387293459	-1.8342832336
H	0.9460458971	3.7262300832	-0.1643165757
C	2.7067652220	-1.3765367275	0.1717147159
K	-0.2300862220	0.4939673516	-1.5553639434
C	3.9272192997	-1.7273443959	-0.6544398555
H	4.8177065009	-1.1270319391	-0.3953410923
H	4.1920985852	-2.7862097871	-0.5085631559
H	3.7458683507	-1.5932051075	-1.7353723189
C	2.7006374586	-1.9843947192	1.5577248423
H	3.1144743500	-3.0055884015	1.5277741315
H	3.2903662286	-1.4037760759	2.2897620691

H	1.6701128942	-2.0717671418	1.9439059435
H	-1.9385979665	-3.0711890002	-0.7687695892
H	-1.1342336884	-1.8268506901	-1.6563717740
H	-2.2764818962	-1.6976792072	1.1971673773
H	-1.3740663227	-0.3666652505	0.5461322987
C	-3.3082844603	-0.6848323335	-0.4061492045
H	-3.9717301732	-1.5368282908	-0.6327275466
H	-3.0845655592	-0.2292660477	-1.3927610760
C	-4.0553165711	0.3323568402	0.4506236430
H	-3.3899690678	1.1889276576	0.6636528871
H	-4.2868123740	-0.1221717518	1.4295203146
C	-5.3442958499	0.8420828676	-0.1866955995
H	-5.1102521506	1.2943409741	-1.1655847361
H	-6.0052891324	-0.0155015372	-0.3966130830
C	-6.0775155413	1.8545472758	0.6842287373
H	-5.4472187197	2.7351828251	0.8832708716
H	-7.0031145911	2.2062666619	0.2059518938
H	-6.3486612717	1.4149319969	1.6564801852

PhMeCHBpin-KOtBu
M06-2X/def2SVP Electronic E: -1553.457648 a.u.
M06-2X/def2SVP Gibbs free E: -1553.052480 a.u.
M06-2X/def2TZVPP Electronic E: -1554.613444 a.u.

C	-0.5984380905	-0.3708945206	1.7372065901
C	-1.9216444877	-0.6299242550	1.0846832418
C	-2.8294166341	0.4233829292	0.8577626607
C	-2.2627286063	-1.8898087107	0.5562235248
C	-3.9896532631	0.2457464922	0.1036818619
H	-2.5999627084	1.4086503949	1.2690570562
C	-3.4241334319	-2.0754388930	-0.1991124090
H	-1.5922587459	-2.7355030388	0.7141655049
C	-4.2902473222	-1.0063459732	-0.4441002395
H	-4.6651325036	1.0888626090	-0.0577242919
H	-3.6521030154	-3.0646174215	-0.6023110924
H	-5.1962633849	-1.1502672115	-1.0352470551
C	-0.0779101073	-1.5501659959	2.5519708214
H	0.1713689744	-2.4022407514	1.9016131582
H	0.8464349754	-1.2659852915	3.0747724328
H	-0.8045059708	-1.9024229707	3.3041339002
H	-0.7173323267	0.4994629851	2.4001226735
B	0.4819050092	0.0665524601	0.5339661759
O	1.8573152878	0.2514122702	0.9893188867
O	0.5628950096	-1.0549496003	-0.4411216516
O	-0.0451266829	1.2562689577	-0.2166049857
K	-1.4273037793	-0.1098329615	-1.8727976616
C	2.7394391453	-0.6290778151	0.3221715684
C	1.8966485508	-1.1587333377	-0.9009138154
C	0.1892443780	2.6120069323	0.1023481672
C	4.0053112792	0.1222595658	-0.0860419820
C	3.1442861882	-1.7532550779	1.2810599441
C	2.0671479086	-0.2832454434	-2.1487832823
C	2.1861776177	-2.6083411952	-1.2736114873
C	1.5529539836	3.0590068156	-0.4363901229
C	-0.9140328868	3.4146001584	-0.5917620763
C	0.1438538719	2.8792644081	1.6104744876
H	4.6946427824	-0.5332995726	-0.6410106736
H	3.7788092718	0.9962040538	-0.7094126325
H	4.5249753099	0.4743579658	0.8180214259
H	2.2838819460	-2.3765235391	1.5525596659
H	3.9249011651	-2.3994210648	0.8524874705
H	3.5394527887	-1.2976858527	2.2015586385
H	3.0787914903	-0.3562830102	-2.5738150015
H	1.3648063612	-0.6267497016	-2.9275615152
H	1.8509447895	0.7674671633	-1.9160245572
H	1.9357714916	-3.2882970211	-0.4491972609
H	1.5795359484	-2.8953077117	-2.1465023460
H	3.2456839067	-2.7479113120	-1.5380436997
H	1.6005777034	2.9143883949	-1.5270959711
H	1.7287905607	4.1255742347	-0.2269897543
H	2.3447455708	2.4642558836	0.0368075489
H	-0.8002524853	4.4943876050	-0.4125057377
H	-0.8776121175	3.2465746356	-1.6807850929
H	-1.9042720414	3.1041205636	-0.2238613905
H	-0.8476229717	2.6477549053	2.0263712998
H	0.8962774492	2.2672507303	2.1282909993
H	0.3564488485	3.9399421361	1.8120700809

styrene-cychex-Bpin-CPhMe2
M06-2X/def2SVP Electronic E: -1303.672145 a.u.
M06-2X/def2SVP Gibbs free E: -1303.095453 a.u.
M06-2X/def2TZVPP Electronic E: -1305.132466 a.u.

C	0.6811930873	-2.9114276354	-1.8319942079
C	1.4634924817	-2.4666879332	-2.8999290543
C	1.6453927249	-1.0978364772	-3.0830053026
C	1.0561659179	-0.1808505216	-2.2082132793
C	0.2769944824	-0.6086000233	-1.1273159147
C	0.0982532021	-1.9916090134	-0.9651910927
H	0.5244296873	-3.9804226212	-1.6738399760
H	1.9249795963	-3.1821894386	-3.5830308949
H	2.2570432114	-0.7320982660	-3.9104459281
H	1.2325846788	0.8824573876	-2.3740429344
H	-0.5015047092	-2.3569945395	-0.1262085120
C	-0.3510244960	0.3289080024	-0.0903284146
C	0.3052099627	0.1408815881	1.2780098470
H	2.2798584432	4.0906290891	2.0501418556
H	-0.3852369521	0.2043038524	2.1256915533
C	4.2639115113	0.9821622072	-0.8793323653
C	3.2121527348	1.0228375141	0.0376068009
C	2.7041341844	-0.1578033951	0.5938682174
C	3.2950982604	-1.3791122562	0.2408639314
C	4.3381670226	-1.4218142770	-0.6822877402
C	4.8228610621	-0.2406268156	-1.2489602652
H	4.6456013689	1.9107091562	-1.3084175948
H	2.7737113725	1.9808319775	0.3272518167
H	2.9134189861	-2.3059366869	0.6761285631
H	4.7727309142	-2.3826167286	-0.9653635443
H	5.6399636250	-0.2746922612	-1.9719849555
C	1.5891040213	-0.1082434350	1.5873768333
C	2.0167713186	-0.3529018277	3.0130187054
H	2.8093043300	0.3516237991	3.3111490351
H	1.1730914307	-0.2553922965	3.7105363960
H	2.4409373368	-1.3653275247	3.1176211936
C	2.5035011176	3.3798349409	2.8567765302
H	1.8329438009	2.5137923117	2.7822986638
H	3.5461571856	3.0465868348	2.7718146305
H	2.3564145088	3.8741484690	3.8261360648
B	-1.8902529440	-0.0630289809	0.0771625599
O	-2.5764519142	0.0835945325	1.2543011494
O	-2.6768796903	-0.5552993499	-0.9251082659
C	-3.9786242557	-0.1202535872	0.9736507748
C	-3.9256561917	-0.9773159250	-0.3377199335
C	-4.6304861648	-0.8113849411	2.1573807772
C	-4.5977282152	1.2566338973	0.7566891047
C	-3.8021496516	-2.4720469141	-0.0602512988
C	-5.0547786137	-0.7098807616	-1.3159956010
H	-4.0978242947	-1.7302494872	2.4322018547
H	-4.6246370659	-0.1371868632	3.0256842502
H	-5.6760294683	-1.0625322250	1.9254771403
H	-5.6815388124	1.1855512250	0.5899599445
H	-4.4229135998	1.8703546427	1.6518943368
H	-4.1432887706	1.7668206507	-0.1058813629
H	-4.7453062063	-2.8893837271	0.3192231754
H	-3.5439507461	-2.9852558315	-0.9974929487
H	-3.0086004873	-2.6759015894	0.6739696593
H	-4.9394390126	-1.3583570505	-2.1961335396
H	-6.0266780660	-0.9316212571	-0.8509159132
H	-5.0537012947	0.3334821278	-1.6558768275
C	-0.3127937875	1.8345583368	-0.5194519387

C	-1.1820079131	2.1386794634	-1.7503537958
C	-0.7020623253	2.7988604787	0.6101720361
H	0.7365262716	2.0633488310	-0.7788810388
C	-1.0184035928	3.5876440336	-2.2079185578
H	-2.2411336020	1.9684693731	-1.4885268540
H	-0.9585699409	1.4439743048	-2.5725992664
C	-0.5570323962	4.2570804691	0.1721868765
H	-1.7468292588	2.6033408649	0.9116930394
H	-0.0783476733	2.6177394702	1.4974215825
C	-1.3745921553	4.5575068877	-1.0826837881
H	-1.6446236010	3.7777633516	-3.0931662739
H	0.0282647219	3.7557984491	-2.5177278808
H	-0.8514146002	4.9281739894	0.9940571438
H	0.5079609491	4.4619959438	-0.0371295036
H	-1.2195301517	5.5991536320	-1.4038111969
H	-2.4484908901	4.4530983787	-0.8469092807

styrene-cychex
M06-2X/def2SVP Electronic E: -543.682113 a.u.
M06-2X/def2SVP Gibbs free E: -543.435140 a.u.
M06-2X/def2TZVPP Electronic E: -544.286633 a.u.

C	2.9153934400	0.6730521992	-0.0206002586
C	3.1413416542	-0.6631135598	0.3136967819
C	2.0552919327	-1.5271430039	0.4475922569
C	0.7557009201	-1.0603769301	0.2519311760
C	0.5105636232	0.2852333275	-0.0670658856
C	1.6168818648	1.1396229501	-0.2099574364
H	3.7578092719	1.3558579716	-0.1462215034
H	4.1586762838	-1.0302054913	0.4599664426
H	2.2180810180	-2.5752548203	0.7054177623
H	-0.0784546461	-1.7552692499	0.3635786956
H	1.4571354594	2.1803785709	-0.4973798722
C	-0.8795922329	0.7951961753	-0.2532511664
C	-1.1855080467	2.0676688861	0.0395808489
H	-2.1963808743	2.4576463084	-0.1000391993
H	-0.4443005674	2.7570490329	0.4501070382
C	-1.9388684918	-0.1833914499	-0.7359693239
C	-2.7612321935	-0.7470606711	0.4377391964
C	-2.8846390706	0.4073556859	-1.7902407433
H	-1.4213441361	-1.0322440751	-1.2144736594
C	-3.7731772686	-1.7892508704	-0.0367816619
H	-3.2898445653	0.0951968771	0.9179675955
H	-2.0942221444	-1.1745156593	1.2027224362
C	-3.8869740953	-0.6386572231	-2.2783558262
H	-3.4390820774	1.2543601711	-1.3521783628
H	-2.2993668187	0.8080159858	-2.6325048123
C	-4.6927131049	-1.2190779974	-1.1163427033
H	-4.3638391635	-2.1578361886	0.8159964330
H	-3.2290815371	-2.6587763769	-0.4455280937
H	-4.5603738873	-0.1953304010	-3.0278643202
H	-3.3412138691	-1.4538920768	-2.7846866279
H	-5.3853408303	-1.9942688494	-1.4786132410
H	-5.3127978269	-0.4195007476	-0.6745083857

styrene-cycpen-Bpin-CPhMe2
M06-2X/def2SVP Electronic E: -1264.419499 a.u.
M06-2X/def2SVP Gibbs free E: -1263.857484 a.u.
M06-2X/def2TZVPP Electronic E: -1265.832852 a.u.

C	1.7033798712	-0.9685138776	-2.8143524103
C	2.1895788761	-0.0965484008	-3.7896079981
C	1.9400623646	1.2674637923	-3.6578960822
C	1.2396336473	1.7567867931	-2.5525703857
C	0.7796822252	0.9036134079	-1.5387534652
C	1.0023213036	-0.4725555981	-1.7179258963
H	1.8648314911	-2.0446033496	-2.9076977824
H	2.7402705962	-0.4792371864	-4.6508835383
H	2.2904817357	1.9648264975	-4.4215208360
H	1.0644215625	2.8288746226	-2.4932502095
H	0.6218759130	-1.1802745213	-0.9789403201
C	0.0446232948	1.3933572951	-0.2873195790
C	0.9235077778	1.4958349257	1.0100270498
H	1.1788170096	2.5552503864	1.1648007289
H	0.2787367322	1.2335483584	1.8614293353
C	2.9886312953	-2.9598209759	0.4792137751
C	3.1026494023	-1.5685761850	0.4990721442
C	2.1532601595	-0.7706272930	1.1514045978
C	1.0980219514	-1.4195151129	1.8107374227
C	0.9821068113	-2.8097252005	1.7951169982
C	1.9237809483	-3.5893145105	1.1215968644
H	3.7385347670	-3.5535471653	-0.0476112258
H	3.9382335831	-1.1065697544	-0.0271086805
H	0.3385287637	-0.8331357906	2.3304564555
H	0.1482269995	-3.2880537037	2.3140221195
H	1.8312851351	-4.6767152305	1.1042240661
C	2.2742542047	0.7541376886	1.2080684667
C	2.7309926603	1.1070734782	2.6400542543
H	3.6860072813	0.6129772601	2.8737455150
H	2.8714938978	2.1949456387	2.7418070975
H	1.9879885384	0.7849285980	3.3849478920
C	3.3496074070	1.2882033337	0.2515637992
H	3.3295013730	2.3892443665	0.2540947742
H	4.3530533161	0.9794568514	0.5818841311
H	3.2150632673	0.9463038376	-0.7828416303
B	-1.1906543982	0.4155655936	-0.0369397264
O	-1.6536716461	0.0551595726	1.2015393032
O	-1.9542309489	-0.1117215213	-1.0439934268
C	-2.8958857278	-0.6606796345	1.0316601573
C	-2.8077968780	-1.1204028422	-0.4655703980
C	-2.9495543475	-1.7951419722	2.0396256070
C	-4.0342207238	0.3215204202	1.2835070338
C	-2.0887384141	-2.4561912086	-0.6281599716
C	-4.1342112257	-1.1417950694	-1.2030804542
H	-2.0517765586	-2.4236488773	1.9820369854
H	-3.0175941582	-1.3814904665	3.0561720245
H	-3.8351965259	-2.4237993566	1.8650727277
H	-5.0065697640	-0.1906393320	1.2733584820
H	-3.8940766939	0.7843709881	2.2709224717
H	-4.0538431093	1.1177538530	0.5268719908
H	-2.7114684350	-3.2922450919	-0.2801475378
H	-1.8608244174	-2.6085622222	-1.6928475149
H	-1.1415980208	-2.4677578528	-0.0674114393
H	-3.9736178930	-1.4813846815	-2.2362448572
H	-4.8321277103	-1.8396286047	-0.7173513604
H	-4.5943082806	-0.1464830653	-1.2367435985
C	-0.6266780737	2.7894308732	-0.5299884981
C	-1.6704035074	3.1694792705	0.5512019200
C	0.3285918923	4.0261548604	-0.5876221410
H	-1.1519864734	2.7170457463	-1.4955201014
C	-1.6284954981	4.6947259548	0.6292282872
H	-1.3761782227	2.7506420694	1.5263726549
H	-2.6691343317	2.7741360923	0.3191094239
C	-0.1318254476	4.9812370851	0.5215359508
H	0.2168888906	4.5262052251	-1.5616054369
H	1.3884155391	3.7503046592	-0.4930032306
H	-2.1546781639	5.1338722913	-0.2348050607
H	-2.0909063176	5.0943230317	1.5432255700
H	0.1035069152	6.0329286593	0.3037800247
H	0.3555626327	4.7220077972	1.4761906110

styrene-cycpen
M06-2X/def2SVP Electronic E: -504.406108 a.u.
M06-2X/def2SVP Gibbs free E: -504.187187 a.u.
M06-2X/def2TZVPP Electronic E: -504.967272 a.u.

C	2.8624334806	0.7108728646	0.1712467966
C	3.0491406202	-0.6198541100	0.5505890700
C	1.9556362655	-1.4832363080	0.5676635328
C	0.6870416848	-1.0214075298	0.2149585876
C	0.4780504795	0.3184342032	-0.1492162811

C	1.5956631702	1.1713818451	-0.1763293543
H	3.7131773629	1.3939657597	0.1347777137
H	4.0425250244	-0.9820051424	0.8208662369
H	2.0867051561	-2.5278558340	0.8559699906
H	-0.1510672527	-1.7180262901	0.2370710021
H	1.4749978406	2.2070709153	-0.4979308032
C	-0.8809355359	0.8209930959	-0.5111527949
C	-1.1918698456	2.1162859402	-0.3563639409
H	-2.1745965143	2.5063800369	-0.6286569807
H	-0.4826549075	2.8305087532	0.0667096453
C	-1.8937142208	-0.2036105694	-1.0017565751
C	-2.6132398602	-0.9202510009	0.1928373753
C	-3.0491061855	0.3821372271	-1.8490569321
H	-1.3445826809	-0.9459691255	-1.5990013478
C	-4.1192633554	-0.8074920375	-0.0896045293
H	-2.3674911685	-0.4030683389	1.1329332658
H	-2.2962798377	-1.9664797358	0.3060530821
C	-4.2442262483	0.4819612423	-0.8988818800
H	-3.2965434660	-0.3350683039	-2.6467036378
H	-2.7823412148	1.3291011882	-2.3374026297
H	-4.4504755733	-1.6566767296	-0.7088740965
H	-4.7225188645	-0.8131221450	0.8293835352
H	-5.2056969869	0.5770545970	-1.4237430179
H	-4.1367336659	1.3536803120	-0.2325325127

tBuBpin-KOtBu-deBpin-rea
M06-2X/def2SVP Electronic E: -1401.168414 a.u.
M06-2X/def2SVP Gibbs free E: -1400.787038 a.u.
M06-2X/def2TZVPP Electronic E: -1402.165179 a.u.

C	-0.7628049690	-0.6977686560	1.7099801196
B	0.3666731898	-0.1275086070	0.6402184212
O	1.7370963169	0.0288867042	1.1269699129
O	0.5003279973	-1.1524295673	-0.4528226075
C	1.8559554618	-1.3209341396	-0.8096417427
C	2.6406771095	-0.8344443382	0.4726479314
C	2.1575120073	-0.4675923718	-2.0483231543
H	3.1983461530	-0.5722747740	-2.3872359676
H	1.5169431245	-0.8020584468	-2.8827980659
H	1.9616758737	0.5934263612	-1.8496056598
C	2.1008480679	-2.7847539398	-1.1659039234
H	1.7726719045	-3.4523808077	-0.3591841332
H	1.5315818159	-3.0439068674	-2.0720720609
H	3.1658115730	-2.9743982586	-1.3704029252
C	3.9271538968	-0.0738051947	0.1525746221
H	4.6422651018	-0.7102036426	-0.3915244763
H	3.7345291747	0.8237954782	-0.4481850403
H	4.4020111722	0.2434899547	1.0932661155
C	3.0101964980	-1.9898849647	1.4105148370
H	2.1472416083	-2.6300544457	1.6241820678
H	3.8149128535	-2.6142293439	0.9944975969
H	3.3608644640	-1.5621702313	2.3619037473
O	-0.1709828065	1.1414677829	0.0100355062
C	0.2105426807	2.4903500324	0.1881648503
K	-1.2001139200	-0.0097944166	-1.9974646901
C	1.3428465865	2.8498300438	-0.7798725725
H	1.0542577955	2.6193852676	-1.8184919425
H	1.5878307235	3.9215608885	-0.7252648075
H	2.2428346589	2.2730113947	-0.5266236797
C	-1.0242500386	3.3314496857	-0.1500719398
H	-0.8248432773	4.4070885403	-0.0296936671
H	-1.3373011577	3.1630273209	-1.1945138679
H	-1.8601629443	3.0549577534	0.5104575334
C	0.6686765983	2.8280367400	1.6120235280
H	-0.1618370827	2.7631157827	2.3258213741
H	1.4633113035	2.1394049688	1.9254024118
H	1.0545340903	3.8587400632	1.6394866889
C	-0.3002609767	-2.0113846301	2.3400528085
H	-0.0473916139	-2.7520254066	1.5644411659
H	0.5934098913	-1.8567442051	2.9641978871
H	-1.0842750032	-2.4503380381	2.9852842847
C	-1.0697783138	0.2915225108	2.8337485939
H	-0.1576335909	0.5813393485	3.3802542680
H	-1.5322683301	1.2089693512	2.4387327252
H	-1.7743666546	-0.1419850494	3.5685607121
C	-2.0714932226	-0.9690732437	0.9552776056
H	-2.4691608541	-0.0413458459	0.5062015991
H	-1.9279652251	-1.7293257130	0.1671425300
H	-2.8597856717	-1.3550966582	1.6273458796

tBuBpin-KOtBu-deBpin-ts
M06-2X/def2SVP Electronic E: -1401.107053 a.u.
M06-2X/def2SVP Gibbs free E: -1400.729502 a.u.
M06-2X/def2TZVPP Electronic E: -1402.106723 a.u.

C	-0.8652489804	-0.7434791242	1.6976221978
B	0.9825308950	0.3659214578	-0.2347631867
O	2.1673414597	0.5677740075	0.4364929092
O	1.0275379820	-0.7961390418	-1.0219558246
C	2.4083289636	-1.1790592114	-1.1191921843
C	3.0215452049	-0.5646406430	0.1915655246
C	2.9674817376	-0.5309583659	-2.3839217436
H	4.0145805781	-0.8160158950	-2.5573367854
H	2.3701787828	-0.8614644641	-3.2457973879
H	2.9070033455	0.5662735047	-2.3247324861
C	2.5086093658	-2.6912720430	-1.2131566287
H	1.9681910188	-3.1799067140	-0.3925206625
H	2.0750107273	-3.0318042773	-2.1648709279
H	3.5608237797	-3.0110599080	-1.1849483804
C	4.4505969879	-0.0693198512	0.0395957885
H	5.1166932342	-0.8957878011	-0.2497203409
H	4.5259757145	0.7264791789	-0.7122936489
H	4.8016999508	0.3320209374	1.0011648315
C	2.9231309855	-1.5142682459	1.3773133717
H	1.9023226725	-1.9042776987	1.4775484381
H	3.6208199865	-2.3565882708	1.2682335671
H	3.1788667906	-0.9672140180	2.2957747937
O	0.0874512505	1.3404904750	-0.6130076298
C	0.1464877249	2.7172445602	-0.2155490230
K	-1.5559823810	-0.6946015903	-1.0535428078
C	1.3610868157	3.3659116650	-0.8765662161
H	1.3093659027	3.2417860170	-1.9687649237
H	1.3934341951	4.4415656928	-0.6495504952
H	2.2878339475	2.9026339789	-0.5101769105
C	-1.1425858592	3.3430911052	-0.7348756946
H	-1.1780203239	4.4149484707	-0.4935806560
H	-1.2104311641	3.2293565612	-1.8272025855
H	-2.0155535235	2.8559125079	-0.2733706112
C	0.2206582315	2.8390833142	1.3026217209
H	-0.6510611014	2.3550520987	1.7644023417
H	1.1317559830	2.3607724702	1.6868697953
H	0.2284977605	3.9001957909	1.5922680230
C	-0.9819433814	-2.2596358320	1.6413274902
H	-1.5629452811	-2.6439430318	0.7754647770
H	-0.0005556834	-2.7646605357	1.5970243724
H	-1.5060967707	-2.6889608823	2.5381015216
C	-0.1254667777	-0.3897851107	2.9745984179
H	0.9037315918	-0.7813429972	3.0003648424
H	-0.0532349713	0.6996671442	3.1366627363
H	-0.6365975881	-0.7990818973	3.8896111298
C	-2.2752241947	-0.1841045638	1.8544890543
H	-2.2976072904	0.9190321680	1.9212467887
H	-2.9857376676	-0.4634002501	1.0471202090
H	-2.7793896465	-0.5551342418	2.7872577289

tBuK
M06-2X/def2SVP Electronic E: -757.442714 a.u.
M06-2X/def2SVP Gibbs free E: -757.359619 a.u.
M06-2X/def2TZVPP Electronic E: -757.703599 a.u.

C	-0.2534154919	-0.7105057162	1.3557573266

C	-0.0597691204	-2.1088665175	1.9120451530
H	0.0135497723	-2.8768689332	1.1216328545
H	0.8509603288	-2.1999910303	2.5303211961
H	-0.9134004491	-2.4269953807	2.5724763302
C	-0.4087395528	0.2408978925	2.5351849100
H	0.4772789128	0.2564017982	3.1943753945
H	-0.6026182860	1.2933282097	2.2391803609
H	-1.2796640819	-0.0310133848	3.1934945528
C	-1.5682405665	-0.6951857043	0.5865578913
H	-1.8338057144	0.3005753302	0.1730654848
H	-1.5784075700	-1.4025538191	-0.2616403208
H	-2.4461857224	-0.9739505222	1.2324558840
K	0.8274109316	1.2570258976	-0.2518020878
